# Association of internet use and health service utilization with self-rated health in middle-aged and older adults: findings from a nationally representative longitudinal survey

**DOI:** 10.3389/fpubh.2024.1429983

**Published:** 2024-10-03

**Authors:** Ximin Ma, Qi Hu, Jiahui He, Wenlong Wang, Kexin Chen, Hui Qiao

**Affiliations:** ^1^School of Public Health, Ningxia Medical University, Yinchuan, China; ^2^Key Laboratory of Environmental Factors and Chronic Disease Control, Yinchuan, China; ^3^School of Humanities and Management, Ningxia Medical University, Yinchuan, China

**Keywords:** internet use, self-rated health, middle-aged and older adults, propensity score matching, instrumental variable, mediating effect

## Abstract

**Objective:**

This study aims to explore the relationship between internet use and self-rated health among middle-aged and older adults and further investigates the mediating role of health service utilization between internet use and self-rated health, providing a reference for more effectively utilizing the internet to improve the health status of middle-aged and older adults.

**Method:**

We analyzed data from the 2018 and 2020 waves of the China Health and Retirement Longitudinal Study (CHARLS), this includes 10,011 in 2018 and 7,080 in 2020 over the age of 45. To explore the relationship between internet use and self-rated health, we employed propensity score matching (PSM) and instrumental variable regression analysis, accounting for a wide range of potential covariates. Additionally, the Sobel test was used to examine the mediating effect of health service utilization on this relationship.

**Results:**

According to the fully adjusted propensity score matching and instrumental variable regression model, internet use significantly enhanced self-rated health among middle-aged and older adults (β = 0.348, *P* < 0.01; β = 0.293, *P* < 0.1). However, subgroup analyses revealed that this positive effect was not significant among middle-aged and older adults who were divorced (β = 0.190, *P* > 0.05), lived in special zone (β = −1.379, *P* > 0.05), and lacked medical insurance (β = 0.314, *P* > 0.05). Furthermore, mediation analysis revealed that inpatient services (β = 0.0215, *P* < 0.01) acted as a mediator in the relationship between internet use and self-rated health.

**Conclusion:**

Internet use positively correlates with self-rated health among middle-aged and older adults. Additionally, the utilization of health services can significantly influence this relationship. These findings underscore the importance of developing targeted government strategies to promote internet access and create a supportive online environment, thereby enhancing the health outcomes of this demographic group.

## 1 Introduction

Since the 1990s, the aging process in China has accelerated, characterized by its fast pace and large scale. According to the seventh national census data, the population aged 60 and above accounts for 18.7% of the total population, with those aged 65 and above accounting for 13.5% (1, 2), approaching the 14% threshold of a deeply aging society (3). The aging trend is becoming increasingly severe. Moreover, the rapid development of the internet has enabled more older adult people in China to enjoy convenient lives, with the internet penetration rate among the older adult population increasing steadily. As of December 2021, the number of older adult internet users in China aged 60 years and above reached 119 million, with an internet penetration rate of 43.2% (4). On the one hand, internet technology has facilitated people's daily lives, especially through the use of mobile internet via smartphones, which has brought great convenience (5). On the other hand, the advent of the internet has also had some negative impacts on people, particularly manifested as internet addiction (6). Health ecology theory posits that the environment's impact on individuals is multilayered and involves complex influencing factors (7). The proficiency in internet use among middle-aged and older groups is relatively low, resulting in a more challenging learning process for them. The digital anxiety they experience can lead to greater psychological resistance to using the internet, thereby causing disparities in internet usage among older adult individuals. Therefore, against the backdrop of population aging and the digital divide among older adult in China, studying the impact of internet use on the health of middle-aged and older Chinese people holds practical significance.

In recent years, scholars have explored the relationship between internet use and health. Studies have shown that internet technology overcomes the limitations of time and space, providing abundant information resources (8). By providing health knowledge, information, resources, and services to older adult people through the internet, the health status of older adult users can be effectively improved (9). The internet offers a platform for information sharing and doctor–patient interactions (10, 11). This online platform allows users to influence their health behaviors through information sharing and learning (12). Compared with non-users, older adult internet users show improvements in indicators of wellbeing, mental and physical health, and health behaviors. Additionally, internet use is significantly associated with better self-rated health (13–15), higher life satisfaction (16, 17), lower levels of depression and/or anxiety (18–20), and more favorable health behaviors (21, 22). However, other studies have reported opposite results. In the United States, Matusitz reported that internet use negatively impacts residents' health, specifically because it leads to sedentary and inactive behaviors, thereby increasing the risk of weight gain and obesity (23). Additionally, Hökby noted that both the frequency and accessibility of internet use have negative effects on health (24). Choi's survey in South Korea revealed that excessive internet use can have negative health impacts (25). Other studies have indicated that the internet compresses face-to-face communication among groups, thereby affecting the maintenance of interpersonal relationships (26), leading to self-isolation, inhibiting the expression of personal emotions (27), and increasing the tendency of internet users to suffer from depression (28).

Owing to differences in demographic characteristics and socioeconomic development levels across various countries and regions, the results of existing studies on the relationship between internet use and health vary. Overall, the current research has the following shortcomings: some studies on the relationship between internet use and health are from developed Western countries, while related research in developing countries such as China is still lacking; some studies focus on the health impact of internet use on middle-aged and older people but rarely consider the differences in self-rated health between internet users and non-users. Self-rated health (SRH) is considered a comprehensive and sensitive indicator for assessing health status (29, 30). In some cases, SRH is regarded as a more accurate predictor of mortality than many objective health indicators (31). Additionally, research on the potential mechanisms of the relationship between internet use and health is insufficient, often overlooking the important role of healthcare service utilization as a potential mediating variable and rarely considering the endogeneity issues that may lead to self-selection bias in samples.

With respect to the relationship between the internet and healthcare service utilization, research indicates that internet use provides a more convenient avenue for health management and medical services. Through the internet, people can access medical information, make doctor appointments, conduct online consultations, and purchase medications, effectively improving the efficiency and quality of medical resource utilization and services (32). Additionally, the internet can enhance self-health management, access to health information, and disease prevention (33, 34) by providing information resources and establishing interactive communication platforms (12). During the COVID-19 pandemic, the role of the internet in providing health-related information and healthcare services has increased significantly, leading some to recognize it as one of the key determinants of health (6).

In summary, the literature has not reached a consensus on the relationship between the internet and health. Previous studies have investigated the relationship between internet use and self-rated health among middle-aged and older individuals, but there is still a lack of research examining healthcare service utilization as a key mediating variable in the relationship between internet use and self-rated health among middle-aged and older adults individuals. Therefore, this study focuses on the health status disparities among older adults in China under the digital divide, explores the association between internet use and self-rated health among middle-aged and older adults self-rated health and the mediating role of healthcare service utilization. This study utilizes data from the China Health and Retirement Longitudinal Study (CHARLS) for 2018 and 2020. The sample includes a nationally representative group of individuals aged 45 and above, providing a broader and more comprehensive perspective. This study explores the relationship between internet use and self-rated health, aiming to provide new empirical evidence for a deeper understanding of technological advancements and their impact on population health.

## 2 Methods

### 2.1 Sample and data collection

**Figure 1:**
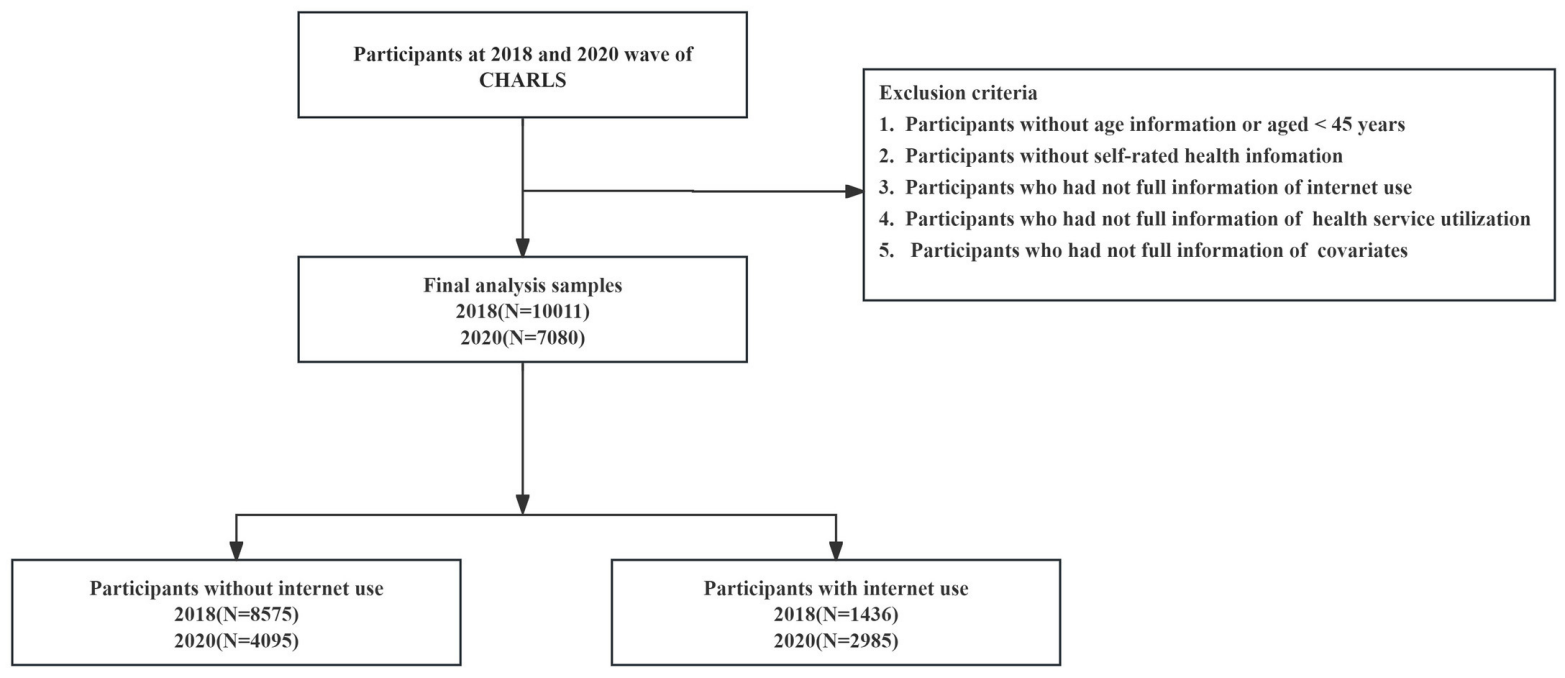
Flow diagram of the inclusion and exclusion criteria.

The China Health and Retirement Longitudinal Study (CHARLS) is an extensive interdisciplinary survey initiative spearheaded by the National Development Institute of Peking University and executed by the China Social Science Survey Center of Peking University. This project aims to gather high-quality microdata that accurately represent the households and individuals of Chinese adults aged 45 years and above. CHARLS has conducted extensive surveys and interviews across 150 counties and 450 communities (villages) within 28 provinces (including autonomous regions and municipalities) during the years 2011, 2013, 2015, 2018, and 2020 (35). The project kicked off with the CHARLS National Baseline Survey in 2011, a comprehensive effort that spanned 2 years and involved 23,000 respondents across 12,400 households. Our current analysis draws from the data collected in the 2018 and 2020 cycles, specifically targeting adults aged 45 years and above. From these two cycles, a total of 17,091 subjects were screened, and the detailed process of participant selection is illustrated in Figure 1.

### 2.2 Survey instrument and variable selection

#### 2.2.1 Explained variable (Y): self-rated health

Self-rated health (SRH) serves as a pivotal metric for evaluating aging and public health, functioning as the dependent variable in our study. Research has established a significant link between SRH and critical outcomes such as mortality, objective health indicators, and mental health (36, 37). Consequently, employing SRH as a measure to gauge the overall health status of middle-aged and older adults is both reliable and validated (38). The CHARLS questionnaire probes participants' perceptions of their current physical health, a method widely recognized for assessing SRH. This survey instrument addresses both objective and subjective health aspects, offering a comprehensive view of an individual's health. Health is classified into five levels: “very bad,” “bad,” “fair,” “good,” and “very good,” which correspond to numeric scales ranging from 1 to 5. This scale facilitates the evaluation of self-rated health in individuals aged 45 and older, providing a structured approach to health assessment.

#### 2.2.2 Explanatory variables (X): internet use

The independent variable, internet use among middle-aged and older adults, was assessed through the CHARLS questionnaire. This questionnaire featured a specific question inquiring about participants' internet activities in the previous month, asking, “Did you go online in the past month?” The range of online activities considered included chatting, reading news, watching videos, playing games, and managing finances via mobile phone networks. The responses were categorized simply as yes (1) or no (0) to indicate whether the participants had engaged in any online activities.

#### 2.2.3 Mediating variable (M): health service utilization

The mediating variable is healthcare service utilization among middle-aged and older individuals. According to previous research, outpatient and inpatient service utilization are often used as indicators to measure healthcare service utilization (39–41). This study uses “outpatient” and “inpatient” as two measurement indicators to assess healthcare service utilization among middle-aged and older individuals. The CHARLS questionnaire includes the following questions: “In the past month, have you visited an outpatient clinic or received home medical services?” and “Have you been hospitalized in the past year?” The answers to “outpatient” and “inpatient” are “yes” or “no” and are binary variables.

#### 2.2.4 Control variable

Research has shown that a combination of individual characteristics, family characteristics, and social insurance policies significantly influences self-rated health (42–44). Specifically, individual characteristics encompass demographics such as sex, age, marital status, education, drinking, exercise, and sleep duration. Family characteristics consider factors such as the current address and the duration of residence of children. Additionally, social insurance coverage, comprising medical insurance and endowment insurance, (Table 1).

**Table 1:** Variable definitions and descriptive statistics (*N* = 17,091).

**Variables**	**Explanation**	**Code**	**2018**		**2020**	
			**Mean**	**SD**	**Mean**	**SD**
Explained variable (Y)	Self-rated health	Very bad = 1, Bad = 2, Fair = 3, Good = 4, Very good = 5	3.050	1.030	3.054	1.027
Explanatory variable (X)	Internet use	Yes = 1 and No = 0	0.143	0.351	0.422	0.494
Control variable (C)	Sex	Male = 1 and Female = 0	0.475	0.499	0.444	0.497
	Age	45–59 = 1,60-74 = 2, ≥75 = 3	1.700	0.680	1.646	0.648
	Marital status	Married = 1, Divorced = 2, Widowed = 3	1.397	0.786	1.421	0.803
	Education	Primary school and below = 1, Middle school = 2, High school and above = 3	1.500	0.725	1.478	0.708
	Current address	City Center or Town Center = 1, Combination Zone Between Urban and Rural Areas/ZhenXiang Area = 2, Village = 3, Special Zone = 4	2.520	0.815	2.419	0.839
	Medical insurance	Yes = 1 and No = 0	0.970	0.170	0.956	0.204
	Endowment insurance	Yes = 1 and No = 0	0.208	0.406	0.856	0.351
	Duration of residence of children	< 6 months = 0, ≥6 months = 1	0.283	0.451	0.258	0.438
	Drinking	Drink More than Once a Month = 1, Drink but, Less than Once a Month = 2, None of These = 3	2.401	0.873	2.364	0.878
	Exercise	Yes = 1 and No = 0	0.507	0.500	0.581	0.493
	Sleep duration	< 7 h = 1, 7–8 h = 2, ≥8 h = 3	1.671	0.851	1.613	0.820
Mediator variable	Outpatient	Yes = 1 and No = 0	0.173	0.378	0.200	0.400
	Inpatients	Yes = 1 and No = 0	0.170	0.375	0.190	0.392

**Figure 2:**
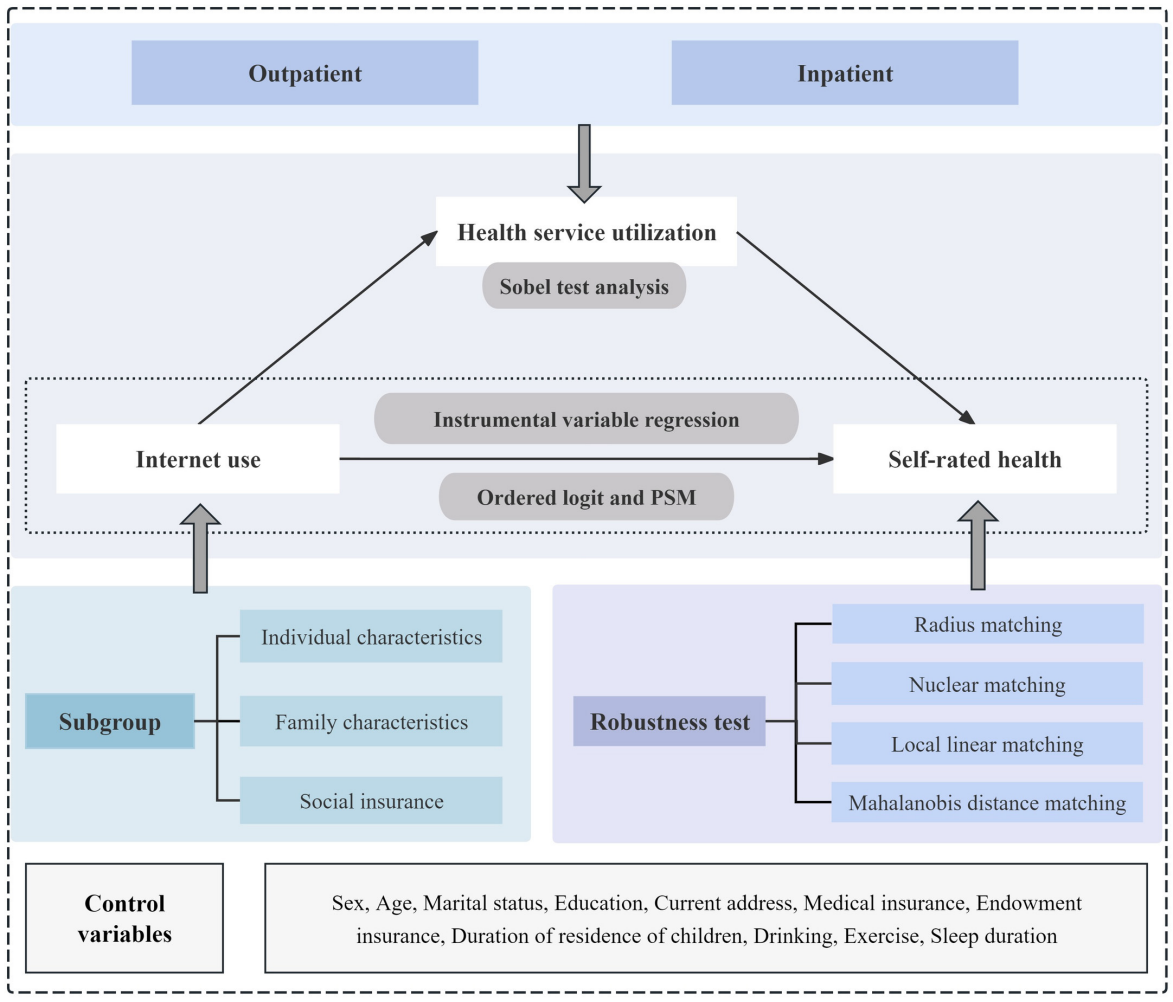
Diagram of the associations between internet use and self-rated health, including potential confounders.

All the variables are defined and assigned in Supplementary Table S1. A flowchart depicting the relationship between internet use and self-rated health is shown in Figure 2.

### 2.3 Statistical analysis

In our study, before conducting the analysis, we rigorously examined all survey data for missing values and outliers. The statistical analysis was performed via econometric software, specifically SPSS 26.0 and STATA version 17.0. Initially, the study approached mixed factor issues via the propensity score matching (PSM) method. This involved balance testing and assessing the common support hypothesis. PSM regression and ordered logit regression analyses were subsequently performed to evaluate the association between internet use and self-rated health. To ensure robust findings, we used various propensity score matching methods and instrumental variable regression. Finally, a mediating analysis was performed to explore the pathway linking internet use and self-rated health. All the statistical tests in this study were performed at a significance level of *P* < 0.05 via a two-tailed approach.

## 3 Results

### 3.1 Variable definitions and descriptive statistics

This study is based on longitudinal data examining the relationship between health service utilization internet use and self-rated health, with 10,011 people included in 2018 and 7,080 people included in 2020. Table 1 shows the variable assignments and basic characteristics of the study population for both surveys in 2018 and 2020.

### 3.2 The propensity score matches the hypothesis of common support

**Figure 3:**
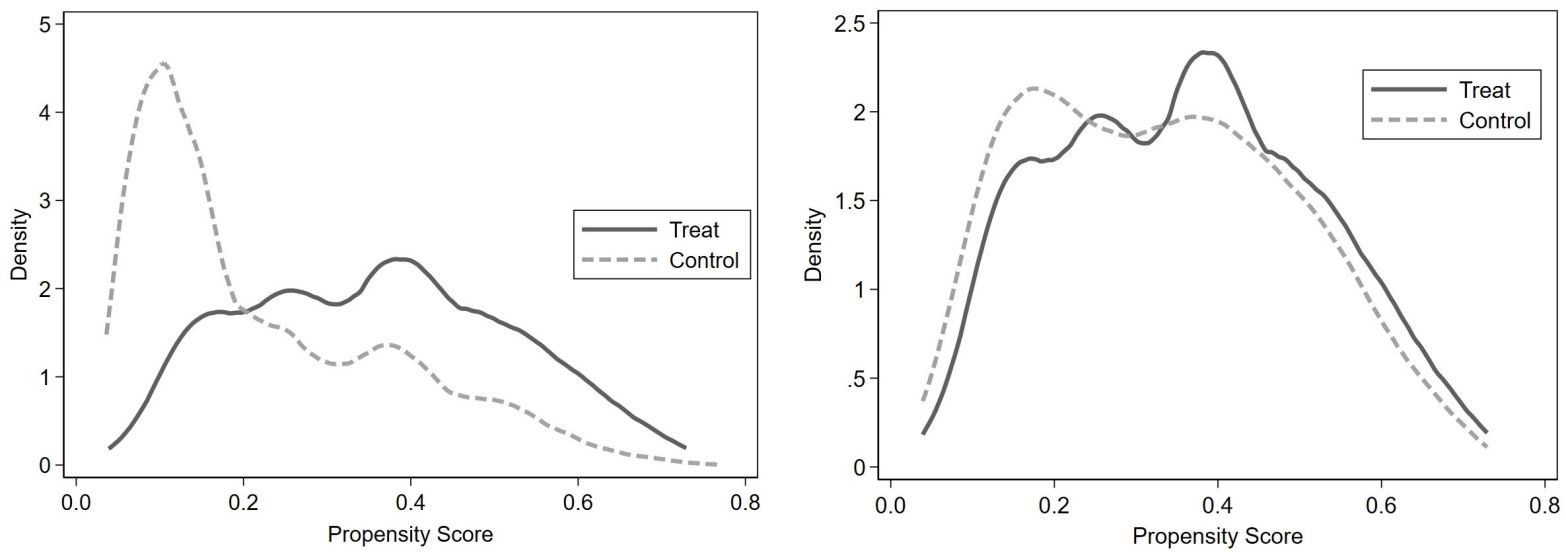
Balance test before and after matching between the treatment group and the control group.

For the effective application of the propensity score matching (PSM) method, it is essential to fulfill two critical conditions: the common support hypothesis and the parallel hypothesis. In our study, kernel density function plots were employed to assess the common support assumption (45–47). As illustrated in Figure 3, there was a noticeable disparity in the propensity score kernel density distributions between the experimental group (Internet users) and the control group (non-Internet users) prior to matching. However, after matching, a significant convergence in the kernel density distribution curves of both groups was observed. This convergence serves as evidence of effective matching, thereby validating the common support hypothesis.

### 3.3 Processing the self-selection problem

To assess the association between internet use and self-rated health among middle-aged and older adults while minimizing potential self-selection bias, we employed the propensity score matching technique. Table 2 demonstrates that the application of nearest-neighbor matching significantly reduced the standard deviations in the samples to < 10% (48, 49), indicating a more uniform variation in the observed characteristics within the sample and effectively managing confounding variables.

**Table 2:** Sample balance test of propensity score matching.

**Variable**	**Sample**	**Mean**		**Standard deviation%**	**Deviation reduction %**	***T*** **test**	
		**Processing group**	**Control group**			**t**	***P***
Sex	Before	0.476	0.458	3.6	73.7	2.07	0.038
	After	0.476	0.480	−1.0		−0.45	0.655
Age	Before	1.517	1.734	−33.5	96.4	−18.84	< 0.001
	After	1.517	1.525	−1.2		−0.60	0.549
Marital status	Before	1.326	1.436	−14.3	73.1	−7.96	< 0.001
	After	1.326	1.296	3.9		1.97	0.049
Education	Before	1.690	1.422	36.4	96	21.73	< 0.001
	After	1.690	1.680	1.5		0.64	0.522
Current address	Before	2.236	2.563	−38.7	96.3	−22.98	< 0.001
	After	2.236	2.248	−1.4		−0.64	0.525
Medical insurance	Before	0.969	0.963	2.9	−5.9	1.65	0.098
	After	0.969	0.974	−3.1		−1.59	0.111
Endowment insurance	Before	0.737	0.386	75.6	96.1	42.25	< 0.001
	After	0.737	0.751	−3.0		−1.49	0.137
Duration of residence of children	Before	0.266	0.275	−2.0	0.2	−1.17	0.244
	After	0.266	0.257	2.0		0.97	0.333
Drinking	Before	2.210	2.447	−26.9	84.5	−15.62	< 0.001
	After	2.210	2.247	−4.2		−1.91	0.057
Exercise	Before	0.603	0.515	17.8	99.2	10.15	< 0.001
	After	0.603	0.602	0.1		0.07	0.948
Sleep duration	Before	1.600	1.663	−7.7	84	−4.34	< 0.001
	After	1.600	1.590	1.2		0.6	0.550

### 3.4 Comprehensive evaluation of the effect of internet use on self-rated health among middle-aged and older adults

Upon detailed analysis of Table 3, which includes columns (1) through (4), we delved into how internet use impacts the self-rated health of middle-aged and older adults. Ordered logit regression analyses were conducted for columns (1) and (2), whereas propensity score matching (PSM) methodologies were implemented in columns (3) and (4). Column (1) initially revealed a significant positive correlation between internet use and self-rated health without adjusting for control variables. The findings across columns (2) and (4) consistently demonstrated a significant positive relationship between internet use and self-rated health, even when additional variables were accounted for. Notably, in column (4), control variables such as current address, alcohol consumption, and sleep duration were found to be statistically significant at the 1% level, reinforcing the robustness of the association.

**Table 3:** Results of the ordered logit model and PSM.

**Variable**	**(1)**	**(2)**	**(3)**	**(4)**
**Internet use (reference:No)**				
Yes	0.540^***^	0.356^***^	0.375^***^	0.348^***^
	(0.034)	(0.036)	(0.069)	(0.069)
**Sex (reference:Female)**				
Male		0.020		−0.002
		(0.031)		(0.071)
**Age (reference: 45–59)**				
60–74		−0.227^***^		−0.112
		(0.032)		(0.076)
≥75		−0.234^***^		−0.107
		(0.053)		(0.121)
**Marital status (reference: Married)**				
Divorced		−0.143		−0.329^*^
		(0.103)		(0.182)
Widowed		−0.015		0.066
		(0.040)		(0.092)
**Education (reference: Primary school and below)**				
Middle school		0.152^***^		0.040
		(0.036)		(0.084)
High school and above		0.168^***^		0.175^*^
		(0.046)		(0.092)
Current address (reference: City Center or Town Center)				
Combination Zone Between Urban and Rural Areas/ZhenXiang Area		−0.093^*^		−0.067
		(0.055)		(0.101)
Village		−0.230^***^		−0.197^***^
		(0.038)		(0.080)
Special Zone		0.171		0.115
		(0.269)		(0.690)
**Medical insurance (reference: No)**				
Yes		−0.226^***^		−0.287^*^
		(0.079)		(0.158)
**Endowment insurance (reference: No)**				
Yes		0.147^***^		0.009
		(0.041)		(0.085)
**Duration of residence of children (reference:**<**6 months)**				
≥6 months		0.075^**^		0.130^*^
		(0.032)		(0.073)
**Drinking (reference: Drink more than once a month)**				
Drink, but less than once a month		−0.187^***^		−0.183^*^
		(0.056)		(0.104)
None of these		−0.474^***^		−0.569^***^
		(0.034)		(0.078)
**Exercise (reference: No)**				
Yes		0.072^**^		−0.060
		(0.029)		(0.069)
**Sleep duration (reference:**<**7 h)**				
7–8 h		0.572^***^		0.610^***^
		(0.039)		(0.086)
≥8 h		0.558^***^		0.590^***^
		(0.035)		(0.084)
Time constant	Yes			
N	1,7091	1,7091	3,082	3,082
Pseudo R^2^	0.0054	0.0232	0.0056	0.0231
Prob>F	< 0.001	< 0.001	< 0.001	< 0.001

^***^P < 0.01, ^**^P < 0.05, ^*^P < 0.1. The numbers in the brackets are the standard errors of coefficient robustness.

### 3.5 Robustness test based on the matching approach

This study adopted a variety of propensity score matching methods—including radius matching, nuclear matching, local linear matching, and Mahalanobis distance matching—to confirm the robustness of its findings (50–52). Table 4 shows that, regardless of the matching technique utilized, internet use consistently had a significant and positive influence on self-rated health (*P* < 0.001), underscoring the stability of our results across different methodologies.

**Table 4:** Robustness test based on the matching approach.

**Variable**	**Radius matching**	**Nuclear matching**	**Local linear matching**	**Mahalanobis distance matching**
Internet use (reference: No)	0.368^***^	0.356^***^	0.367^***^	0.312^***^
Yes	(0.039)	(0.036)	(0.070)	(0.042)
Control variable	Yes	Yes	Yes	Yes
N	12,998	17,091	3,082	8,607
Pseudo R^2^	0.0242	0.0232	0.0224	0.023
Prob>F	< 0.001	< 0.001	< 0.001	< 0.001
Time constant	Yes			

^***^P < 0.01, ^**^P < 0.05, ^*^P < 0.1. The numbers in the brackets are the standard errors of coefficient robustness.

### 3.6 Robustness tests: instrumental variable regression

Although this paper controls for variables such as individual and family characteristics that may affect self-rated health in the empirical model and the propensity score matching (PSM) method can alleviate biases caused by sample self-selection, there may still be endogeneity issues caused by omitted variables or reverse causality. Therefore, to avoid endogeneity issues, whether a family has broadband access is used as an instrumental variable, and a regression is conducted via an instrumental variable model to complete the endogeneity test. The instrumental variable method is an effective tool for addressing endogeneity issues, allowing for a more accurate estimation of the true effect of the explained variable by introducing an instrumental variable. The rationale for selecting this variable as an instrumental variable is 2-fold. First, the instrumental variable is closely related to the endogenous variable of internet use. Second, the instrumental variable is uncorrelated with the error term (53, 54). The estimation results of the instrumental variable are shown in Table 5. The results indicate that after controlling for other variables, the estimation results of the instrumental variable are not significant, suggesting that the instrumental variable does not affect self-rated health and has exogeneity. The first-stage estimation results of the impact of internet use on self-rated health show that family broadband coverage has a significant positive effect on internet use, confirming the validity of the instrumental variable. The second-stage estimation results reveal that internet use can significantly and positively promote self-rated health. This suggests that internet use plays a role in enhancing self-rated health, and this conclusion holds even after the endogeneity issue is addressed.

**Table 5:** Robustness and endogeneity tests: instrumental variable regressions.

**Model**	**(1)**	**(2) First Stage**	**(3) 2SLS Second Stage**
Variable	Self-rated health	Internet use	Self-rated health
Internet use (reference: No)	0.338^***^		0.293^*^
Yes	(0.035)		(0.170)
Instrumental variable (Home broadband coverage)	0.022	0.099^***^	
	(0.031)	(0.007)	
Control variable	Yes	Yes	Yes
R^2^/Pseudo R^2^	0.021	0.148	0.049
Prob>F	< 0.001	< 0.001	< 0.001

^***^P < 0.01, ^**^P < 0.05, ^*^P < 0.1. The numbers in the brackets are the standard errors of coefficient robustness.

### 3.7 Subgroup and sensitivity analyses

Table 6 presents the subgroup analysis outcomes, exploring the relationship between internet use and self-rated health. This analysis demonstrated a stable connection across various demographic subgroups, such as sex, age, education, endowment insurance, duration of residence of children, drinking, exercise, and sleep duration. However, differences were observed based on marital status, current address and the presence of medical insurance. Notably, divorced individuals, lived in special zone and those without medical insurance exhibited no significant difference in the association between internet use and self-rated health (*P* > 0.05).

**Table 6:** Subgroup analyses of associations between internet use and self-rated health (*N* = 17,091).

**Subgroup**	**Self-rated health**				
**Associations between internet use and self-rated health**	**Coefficient**	**SD**	**Z**	**P**	**95%CI**
**By sex**					
Male	0.370	0.052	7.150	< 0.001	(0.269, 0.471)
Female	0.345	0.049	6.980	< 0.001	(0.249, 0.442)
**By age**					
45–59	0.316	0.049	6.440	< 0.001	(0.220, 0.413)
60–74	0.371	0.057	6.460	< 0.001	(0.258, 0.483)
≥75	0.275	0.134	2.060	0.040	(0.013, 0.536)
**By marital status**					
Married	0.360	0.040	9.090	< 0.001	(0.282, 0.437)
Divorced	0.190	0.234	0.810	0.418	(−0.269, 0.649)
Widowed	0.319	0.090	3.550	< 0.001	(0.143,0.495)
**By education**					
Primary school and below	0.354	0.049	7.170	< 0.001	(0.258, 0.451)
Middle school	0.310	0.069	4.510	< 0.001	(0.175, 0.445)
High school and above	0.270	0.084	3.210	0.001	(0.105, 0.434)
**By current address**					
City center or town center	0.305	0.068	4.470	< 0.001	(0.171, 0.439)
Combination Zone Between Urban and Rural Areas/ZhenXiang Area	0.238	0.102	2.340	0.019	(0.039, 0.437)
Village	0.400	0.047	8.570	< 0.001	(0.308, 0.491)
Special Zone	–1.379	1.120	–1.23	0.218	(–3.576, 0.817)
**By medical insurance**					
Yes	0.353	0.036	9.700	< 0.001	(0.281, 0.424)
No	0.314	0.194	1.610	0.107	(−0.067, 0.695)
**By endowment insurance**					
Yes	0.339	0.044	7.750	< 0.001	(0.253, 0.424)
No	0.363	0.064	5.700	< 0.001	(0.239,0.488)
**By duration of residence of children**					
< 6 months	0.333	0.069	4.810	< 0.001	(0.197, 0.468)
≥6 months	0.362	0.042	8.670	< 0.001	(0.280, 0.444)
**By drinking**					
Drink more than once a month	0.339	0.066	5.130	< 0.001	(0.210, 0.469)
Drink, but less than once a month	0.472	0.113	4.170	< 0.001	(0.250,0.695)
None of these	0.340	0.046	7.350	< 0.001	(0.249, 0.430)
**By exercise**					
Yes	0.320	0.047	6.830	< 0.001	(0.228,0.412)
No	0.393	0.056	7.080	< 0.001	(0.284, 0.502)
**By sleep duration**					
< 7 h	0.356	0.046	7.660	< 0.001	(0.265, 0.447)
7–8 h	0.301	0.083	3.640	< 0.001	(0.139,0.463)
≥8 h	0.404	0.077	5.280	< 0.001	(0.254, 0.554)
Time constant	Yes				

^***^P < 0.01, ^**^P < 0.05, ^*^P < 0.1.

### 3.8 Analysis of the mediating effect of the action path

**Figure 4:**
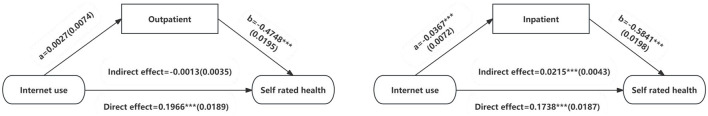
Mediating effects of internet use and health service utilization on self-rated health.

Our study included a mediation analysis to examine the relationships between self-rated health, internet use, health service utilization, and various covariates. The regression analysis revealed a significant positive correlation between the utilization of inpatient services and self-rated health (β = 0.0215, *P* < 0.01; BootSE = 0.0043), which accounted for 10.99% of the total effect observed. Conversely, the use of outpatient services did not demonstrate a significant mediating role, as indicated in Table 7 and Figure 4.The moderating effect results are shown in Supplementary Table S2 and Supplementary Figure S1.

**Table 7:** Mediating role of health service utilization on internet use—self-rated health (*N* = 17,091).

	**Outpatient**		**Inpatients**	
	**Coefficient**	**S.E**.	**Coefficient**	**S.E**.
Sobel	−0.0013	0.0035	0.0215^***^	0.0043
Goodman-1 (Aronian)	−0.0013	0.0035	0.0215^***^	0.0043
Goodman-2	−0.0013	0.0035	0.0215^***^	0.0043
a coefficient	0.0027	0.0074	−0.0367^***^	0.0072
b coefficient	−0.4748^***^	0.0195	−0.5841^***^	0.0198
Indirect effect	−0.0013	0.0035	0.0215^***^	0.0043
Direct effect	0.1966^***^	0.0189	0.1738^***^	0.0187
Total effect	0.1953^***^	0.0192	0.1953^***^	0.0192
Mediating effect (%)	−0.0065		0.1099	
The ratio of indirect to direct effect (%)	−0.0065		0.1234	
The ratio of total to direct effect (%)	0.9935		1.1234	
Control variables	Yes		Yes	
N	17,091		17,091	
Pseudo R^2^	0.0846		0.0982	
Prob>F	< 0.001		< 0.001	
Time constant	Yes		Yes	

^***^P < 0.01, ^**^P < 0.05, ^*^P < 0.1.

## 4 Discussion

Investigating the association between internet use and self-rated health among middle-aged and older adults is crucial for societal and public health, particularly in the context of an aging population. This study focuses on understanding how the internet can support health promotion strategies tailored to middle-aged and older adults. We analyzed data from a nationally representative database of Chinese adults aged 45 years and older. Our findings reveal that internet use significantly enhances the health of middle-aged and older adults. This aligns with prior studies, underscoring the internet's role in improving health (55–57). The positive impact observed can be attributed to comprehensive health information (58), telemedicine services, and social support networks available online. These resources empower middle-aged and older adults in managing their health, increasing their quality of life, and fostering better health conditions.

Subgroup analyses highlighted the nuanced relationship between internet use and self-rated health in middle-aged and older adults. Notably, divorced individuals did not experience significant improvements in self-rated health associated with internet use. This lack of improvement could stem from increased feelings of loneliness and insufficient social support among this group (59). Despite having access to the internet, they may encounter obstacles in obtaining psychological support and substantial health services. In a similar vein, middle-aged and older adults lacking medical insurance, reported no significant health benefits from internet use. This absence of positive outcomes suggests that, while online health resources are accessible, uninsured individuals struggle to utilize these resources effectively and access medical services, primarily because of financial constraints (60).

This study revealed that the utilization of health services, especially inpatient services, plays a crucial role in mediating the relationship between internet use and self-rated health among middle-aged and older adults. The effect of internet use on improving self-rated health is less pronounced among individuals who regularly use outpatient services. This is likely because these services provide immediate access to professional medical advice and treatment, alleviating the uncertainties that come from solely relying on online health information. However, the impact of internet use is mediated by inpatient services, as older adults with serious health conditions benefit from the direct and comprehensive care offered by hospitals and healthcare institutions. Internet use can facilitate the booking of inpatient services and the inquiry of inpatient information, increasing personal utilization of these services. The utilization of inpatient services may, in turn, improve individual health conditions, thereby enhancing the self-assessed health levels of middle-aged and older adults.

## 5 Research strengths and limitations

Our study has several notable strengths. First, it utilized data from an extensive survey that covered 150 counties and districts across China, including major provinces and cities. To our knowledge, no other study has explored the association between health service utilization related to internet use and self-rated health in such a large and diverse Chinese population. Moreover, our analysis meticulously accounted for a wide range of potential confounding factors, ensuring the robustness of our findings. Additionally, the substantial sample size of our study facilitated a detailed evaluation of the heterogeneity in the relationship between internet use and self-rated health, allowing for nuanced insights into how this relationship varies across different segments of the population.

Our study has several limitations that merit consideration. Our research focuses primarily on middle-aged and older adults in China, which may limit the applicability of our findings to younger demographic data or populations from other countries. This focus inherently restricts the generalization of our results beyond the specified group. Additionally, the data for middle-aged and older adults were derived from the 2018 and 2020 CHARLS (China Health and Retirement Longitudinal Study) databases. These databases, while comprehensive, may not perfectly mirror the original populations owing to their screening processes. Moreover, our reliance on self-reported data could lead to an overestimation of the link between internet use and self-rated health.

## 6 Conclusion

Our study, which draws on data from the 2018 and 2020 China Health and Retirement Longitudinal Study (CHARLS), revealed a significant association between internet use and self-rated health among Chinese adults aged 45 years and older. Through subgroup analyses, we found that this association was not significant for individuals who were divorced, lived in special zone and without medical insurance. Furthermore, mediation analyses revealed that the use of inpatient services acted as a bridge connecting internet use to self-rated health. On the basis of these findings, we suggest that policymakers must devise targeted strategies that encourage internet adoption among middle-aged and older individuals. Such initiatives should aim to foster a supportive online environment, thereby improving the overall health and wellbeing of these population segments.

## Data Availability

The datasets presented in this study can be found in online repositories. The names of the repository/repositories and accession number(s) can be found below: http://charls.pku.edu.cn/index.htm.
