# Supplementary material for: Association of internet use and health service utilization with self-rated health in middle-aged and older adults: findings from a nationally representative longitudinal survey
Source: Front Public Health. 2024 Oct 3;12:1429983. doi: 10.3389/fpubh.2024.1429983 (PMC11483889; doi:10.3389/fpubh.2024.1429983)
Supplement: Supplementary file 5 [file Data_Sheet_5.PDF]

China Health and Retirement Longitudinal Study

Wave 5 (2020)

CodeBook

VersionID: 20231106

Nov. 2023

National School of Development
Institute of Social Science Survey
Peking University

This page intentionally left blank

Contents

B	Demographic Backgrounds	1
C	Family Information	9
D	Health Status and Functioning	115
F	Work and Retirement	237
G1	Household Income	259
G2	Individual Income	371
V	COVID Module	397
EX	Exit Module	413
AUX1	Sample Information	559
AUX2	Sampling Weights	561

MODULE B

Demographic Backgrounds

ID: Individual ID

A String Variable	
Obs:	19,395

householdID: Household ID

A String Variable	
Obs:	19,395

communityID: Community ID

A String Variable	
Obs:	19,395

proxy_2: If Demographic Background Moduel Use Proxy Pattern

	Freq.	%
1 Yes	1,420	98.89
2 No	16	1.11
Total	1,436	100.00

ba001: Interviewer record R's gender

	Freq.	%
1 Male	9,090	46.87
2 Female	10,305	53.13
Total	19,395	100.00

ba002: Reconfirm Gender

	Freq.	%

	Freq.	%
1 Male	24	55.81
2 Female	19	44.19
Total	43	100.00

ba003_1: What's your actual date of birth? Year

Mean	SD	Min	Max	Obs
1,964.62	8.60	1,935.00	1,988.00	157

ba003_2: What's your actual date of birth? Month

Mean	SD	Min	Max	Obs
5.76	4.03	-1.00	12.00	157

ba003_3: What's your actual date of birth? Day

Mean	SD	Min	Max	Obs
13.62	10.00	-1.00	31.00	157

ba005: Type of Interview Address

	Freq.	%
1 Family Housing	17,305	89.22
2 Workplace	1,214	6.26
3 Other, Please Specify	876	4.52
Total	19,395	100.00

ba006: What's Your Residential Address Now

	Freq.	%
1 Address of Interview	17,931	92.45
2 Not Address of Interview, Chinese Mainland	1,454	7.50
3 Hong Kong, China	2	0.01
4 Macao, China	1	0.01
6 Abroad	7	0.04
Total	19,395	100.00

ba007: What's the Type of the Residential Address

	Freq.	%
1 Family Housing	2,017	76.06
2 Nursing home	49	1.85
3 Hospital	27	1.02
4 Other, Please Specify	559	21.08
Total	2,652	100.00

ba008: Was It Village or City/Town?

	Freq.	%
1 City Center or Town Center	4,714	24.32
2 Combination Zone Between Urban and Rural Areas / ZhenXiang Area	2,377	12.26
3 Village	12,270	63.30
4 Special Area	24	0.12
Total	19,385	100.00

ba009: Type of HuKou

	Freq.	%
1 Agricultural HuKou	14,431	74.41
2 Non-Agricultural HuKou	2,913	15.02
3 Unified Residence HuKou	2,040	10.52
4 Do not Have HuKou	11	0.06
Total	19,395	100.00

ba010: What's the Highest Level of Education Now? Not Including Adult Education

	Freq.	%
1 No formal education (illiterate)	269	15.75
2 Did not finish primary school	334	19.56
3 Sishu/home school	4	0.23
4 Elementary school	353	20.67
5 Middle school	403	23.59
6 High school	189	11.07
7 Vocational school	54	3.16
8 Two-/Three-Year College/Associate degree	63	3.69
9 Four-Year College/Bachelor's degree	35	2.05
10 Master's degree	3	0.18
11 Doctoral degree/Ph.D.	1	0.06
Total	1,708	100.00

ba010_1: Are You Literate

	Freq.	%
1 Yes	242	39.87
2 No	365	60.13
Total	607	100.00

ba011: What's Your Marital Status

	Freq.	%
1 Married with Spouse Present	14,594	75.25
2 Married but Not Living with Spouse Temporarily for Reasons Such as Work	1,662	8.57
3 Separated, no longer living together as a spouse	96	0.49
4 Divorced	267	1.38
5 Widowed	2,662	13.73
6 Never Married	114	0.59
Total	19,395	100.00

ba012: Do You Have Anyone Living Together as A Spouse?

	Freq.	%
1 Yes	20	0.64
2 No	3,119	99.36
Total	3,139	100.00

ba013: In the Past Year, How Many Months Have You Lived with Your Spouse

Mean	SD	Min	Max	Obs
10.78	3.02	0.00	12.00	16,276

ba014: How Ofen are Social Pensions Paid

	Freq.	%
1 One Month	7,934	40.91
2 One Quarter	653	3.37
3 Half a Year	531	2.74
4 One Year	1,311	6.76
5 More Than One Year	12	0.06
Participate in the Endowment Insurance But Haven't Reached the Age of Receiving It	6,038	31.13
7 Didn't Participate in Any Social Endowment Insurance	2,916	15.03
Total	19,395	100.00

ba014_1: Number of Years

Mean	SD	Min	Max	Obs
3.33	1.78	2.00	7.00	12

ba015: What Type of Social Endowment Insurance Do You Participate in

	Freq.	%
1 Pension Insurance for Employees of State Organs or Public Institutions	938	5.69
2 Worker's Basic Endownment Insurace	2,398	14.55
3 Social Endowment Insurance for Non-working Urban Residents	728	4.42
4 New Social Endowment Insurance for Rural Residents	9,277	56.30
5 Social Endownment Insurance for Urban and Rural Residents	2,430	14.75
6 Other, Please Specify	708	4.30
Total	16,479	100.00

ba016: How Much Does Social Medical Insurance Cost Per Year

	Freq.	%
1 Cost Per Year	15,166	78.20
2 No Need to Pay	3,295	16.99
3 No Social Medical Insurance	934	4.82
Total	19,395	100.00

ba016_1: How Much Does Social Medical Insurance Cost Per Year

Mean	SD	Min	Max	Obs
428.35	1,731.49	-1.00	99,220.00	15,172

ba017: Type of Social Medical Insurance

Mean	SD	Min	Max	Obs
3.36	1.19	1.00	6.00	18,460

ba018: How Many Days Do You Live Alone in the First Half of This Year

Mean	SD	Min	Max	Obs
22.18	52.54	0.00	182.00	19,395

ba019: In the First Half of This Year, How Many Days Do You Live with Your Spouse Only

Mean	SD	Min	Max	Obs
82.42	82.75	0.00	182.00	16,276

ba020: In the First Half of This Year, To What Extent Does Not Live with Others Result

Mean	SD	Min	Max	Obs
8.07	24.59	0.00	100.00	13,441

xr18gender: XR18Gender

A String Variable	
Obs:	19,242

xr20gender: XR20Gender

A String Variable	
Obs:	19,395

daynumber: DayNumber

Mean	SD	Min	Max	Obs
91.35	84.44	0.00	364.00	19,395

xrlivecovid: XRLiveCovid

A String Variable	
Obs:	19,395

xrage: XRAge

Mean	SD	Min	Max	Obs
61.57	10.08	18.00	108.00	19,395

xrgender: XRGender

Mean	SD	Min	Max	Obs
1.53	0.50	1.00	2.00	19,395

xrpartner: XRPartner

Mean	SD	Min	Max	Obs
1.16	0.37	1.00	2.00	19,395

xrresidencesurveyhome: XRResidenceSurveyHome

Mean	SD	Min	Max	Obs
1.14	0.34	1.00	2.00	19,395

xrtype: XRType

Mean	SD	Min	Max	Obs
1.01	0.08	1.00	2.00	19,395

ziwtime: ZIWTime

A String Variable	
Obs:	19,264

zrbirthyear: ZRBirthYear

Mean	SD	Min	Max	Obs
1,958.38	10.07	1,912.00	2,002.00	19,238

zredu: ZREdu

Mean	SD	Min	Max	Obs
3.43	1.92	1.00	11.00	17,687

zrgender: ZRGender

Mean	SD	Min	Max	Obs
1.53	0.50	1.00	2.00	19,242

versionID: Version ID

A String Variable	
Obs:	19,395

This page intentionally left blank

MODULE C

Family Information

householdID: Household ID

A String Variable	
Obs:	11,406

communityID: Community ID

A String Variable	
Obs:	11,406

ca001: Family Respondent

	Freq.	%
1 Main Respondent	4,332	52.24
2 Spouse	3,961	47.76
Total	8,293	100.00

ca002_1_: Is ZChildName[1] Still Alive?

	Freq.	%
1 Yes	10,725	99.45
2 No	59	0.55
Total	10,784	100.00

ca002_2_: Is ZChildName[2] Still Alive?

	Freq.	%
1 Yes	8,833	99.48
2 No	46	0.52
Total	8,879	100.00

ca002_3_: Is ZChildName[3] Still Alive?

	Freq.	%
1 Yes	4,836	99.10
2 No	44	0.90
Total	4,880	100.00

ca002_4_: Is ZChildName[4] Still Alive?

	Freq.	%
1 Yes	2,500	98.58
2 No	36	1.42
Total	2,536	100.00

ca002_5_: Is ZChildName[5] Still Alive?

	Freq.	%
1 Yes	1,161	98.64
2 No	16	1.36
Total	1,177	100.00

ca002_6_: Is ZChildName[6] Still Alive?

	Freq.	%
1 Yes	491	97.23
2 No	14	2.77
Total	505	100.00

ca002_7_: Is ZChildName[7] Still Alive?

	Freq.	%
1 Yes	224	97.82
2 No	5	2.18
Total	229	100.00

ca002_8_: Is ZChildName[8] Still Alive?

	Freq.	%
1 Yes	91	93.81
2 No	6	6.19
Total	97	100.00

ca002_9_: Is ZChildName[9] Still Alive?

	Freq.	%
1 Yes	36	94.74
2 No	2	5.26
Total	38	100.00

ca002_10_: Is ZChildName[10] Still Alive?

	Freq.	%
1 Yes	19	90.48
2 No	2	9.52
Total	21	100.00

ca002_11_: Is ZChildName[11] Still Alive?

	Freq.	%
1 Yes	8	88.89
2 No	1	11.11
Total	9	100.00

ca002_12_: Is ZChildName[12] Still Alive?

	Freq.	%
1 Yes	3	100.00
Total	3	100.00

ca002_13_: Is ZChildName[13] Still Alive?

	Freq.	%
1 Yes	1	100.00
Total	1	100.00

ca002_14_: Is ZChildName[14] Still Alive?

No Observations

ca002_15_: Is ZChildName[15] Still Alive?

No Observations

ca002_16_: Is ZChildName[16] Still Alive?

No Observations

ca002_17_: Is ZChildName[17] Still Alive?

	Freq.	%
1 Yes	1	100.00
Total	1	100.00

ca003_1_1_: When Did ZChildName[1] Pass Away (Year)

Mean	SD	Min	Max	Obs
2,018.41	1.69	2,011.00	2,020.00	59

ca003_1_2_: When Did ZChildName[2] Pass Away (Year)

Mean	SD	Min	Max	Obs
2,018.33	2.11	2,011.00	2,020.00	46

ca003_1_3_: When Did ZChildName[3] Pass Away (Year)

Mean	SD	Min	Max	Obs
2,018.32	2.13	2,011.00	2,020.00	44

ca003_1_4_: When Did ZChildName[4] Pass Away (Year)

Mean	SD	Min	Max	Obs
2,018.22	2.38	2,011.00	2,020.00	36

ca003_1_5_: When Did ZChildName[5] Pass Away (Year)

Mean	SD	Min	Max	Obs
2,018.25	2.32	2,012.00	2,020.00	16

ca003_1_6_: When Did ZChildName[6] Pass Away (Year)

Mean	SD	Min	Max	Obs
2,018.57	1.45	2,015.00	2,020.00	14

ca003_1_7_: When Did ZChildName[7] Pass Away (Year)

Mean	SD	Min	Max	Obs
2,019.60	0.55	2,019.00	2,020.00	5

ca003_1_8_: When Did ZChildName[8] Pass Away (Year)

Mean	SD	Min	Max	Obs
2,017.67	2.88	2,012.00	2,020.00	6

ca003_1_9_: When Did ZChildName[9] Pass Away (Year)

Mean	SD	Min	Max	Obs
2,019.50	0.71	2,019.00	2,020.00	2

ca003_1_10_: When Did ZChildName[10] Pass Away (Year)

Mean	SD	Min	Max	Obs
2,018.50	0.71	2,018.00	2,019.00	2

ca003_1_11_: When Did ZChildName[11] Pass Away (Year)

Mean	SD	Min	Max	Obs
2,018.00	.	2,018.00	2,018.00	1

ca003_2_1_: When Did ZChildName[1] Pass Away (Month)

Mean	SD	Min	Max	Obs
5.88	3.70	1.00	12.00	59

ca003_2_2_: When Did ZChildName[2] Pass Away (Month)

Mean	SD	Min	Max	Obs
6.43	3.84	1.00	12.00	46

ca003_2_3_: When Did ZChildName[3] Pass Away (Month)

Mean	SD	Min	Max	Obs
6.52	3.39	1.00	12.00	44

ca003_2_4_: When Did ZChildName[4] Pass Away (Month)

Mean	SD	Min	Max	Obs
5.64	3.31	1.00	12.00	36

ca003_2_5_: When Did ZChildName[5] Pass Away (Month)

Mean	SD	Min	Max	Obs
3.75	3.57	1.00	12.00	16

ca003_2_6_: When Did ZChildName[6] Pass Away (Month)

Mean	SD	Min	Max	Obs
4.29	3.31	1.00	10.00	14

ca003_2_7_: When Did ZChildName[7] Pass Away (Month)

Mean	SD	Min	Max	Obs
5.40	4.72	1.00	12.00	5

ca003_2_8_: When Did ZChildName[8] Pass Away (Month)

Mean	SD	Min	Max	Obs
7.33	3.83	1.00	12.00	6

ca003_2_9_: When Did ZChildName[9] Pass Away (Month)

Mean	SD	Min	Max	Obs
6.50	7.78	1.00	12.00	2

ca003_2_10_: When Did ZChildName[10] Pass Away (Month)

Mean	SD	Min	Max	Obs
8.50	4.95	5.00	12.00	2

ca003_2_11_: When Did ZChildName[11] Pass Away (Month)

Mean	SD	Min	Max	Obs
1.00	.	1.00	1.00	1

ca003_3_1_: When Did ZChildName[1] Pass Away (Day)

Mean	SD	Min	Max	Obs
13.67	10.03	1.00	29.00	45

ca003_3_2_: When Did ZChildName[2] Pass Away (Day)

Mean	SD	Min	Max	Obs
11.45	9.00	1.00	27.00	31

ca003_3_3_: When Did ZChildName[3] Pass Away (Day)

Mean	SD	Min	Max	Obs
13.65	9.15	1.00	30.00	31

ca003_3_4_: When Did ZChildName[4] Pass Away (Day)

Mean	SD	Min	Max	Obs
10.08	9.29	1.00	30.00	24

ca003_3_5_: When Did ZChildName[5] Pass Away (Day)

Mean	SD	Min	Max	Obs
9.14	10.59	1.00	30.00	14

ca003_3_6_: When Did ZChildName[6] Pass Away (Day)

Mean	SD	Min	Max	Obs
7.50	7.25	1.00	19.00	8

ca003_3_7_: When Did ZChildName[7] Pass Away (Day)

Mean	SD	Min	Max	Obs
6.67	4.93	1.00	10.00	3

ca003_3_8_: When Did ZChildName[8] Pass Away (Day)

Mean	SD	Min	Max	Obs
5.75	4.57	1.00	12.00	4

ca003_3_9_: When Did ZChildName[9] Pass Away (Day)

Mean	SD	Min	Max	Obs
1.00	.	1.00	1.00	1

ca003_3_10_: When Did ZChildName[10] Pass Away (Day)

Mean	SD	Min	Max	Obs
1.00	.	1.00	1.00	1

ca003_3_11_: When Did ZChildName[11] Pass Away (Day)

Mean	SD	Min	Max	Obs
3.00	.	3.00	3.00	1

ca005_1_: XChildPanAliveName[1]'s Birth Year

Mean	SD	Min	Max	Obs
1,982.40	15.05	1,957.00	2,000.00	10

ca005_2_: XChildPanAliveName[2]'s Birth Year

Mean	SD	Min	Max	Obs
1,974.27	12.37	1,959.00	1,996.00	11

ca005_3_: XChildPanAliveName[3]'s Birth Year

Mean	SD	Min	Max	Obs
1,969.25	5.12	1,962.00	1,974.00	4

ca005_4_: XChildPanAliveName[4]'s Birth Year

Mean	SD	Min	Max	Obs
1,973.20	9.15	1,964.00	1,988.00	5

ca005_5_: XChildPanAliveName[5]'s Birth Year

Mean	SD	Min	Max	Obs
1,978.17	8.38	1,967.00	1,989.00	6

ca005_6_: XChildPanAliveName[6]'s Birth Year

Mean	SD	Min	Max	Obs
1,972.00	4.24	1,969.00	1,975.00	2

ca005_7_: XChildPanAliveName[7]'s Birth Year

No Observations

ca006_1_: XChildPanAliveName[1]'s Gender

	Freq.	%
1 Male	26	53.06
2 Female	23	46.94
Total	49	100.00

ca006_2_: XChildPanAliveName[2]'s Gender

	Freq.	%
1 Male	23	62.16
2 Female	14	37.84
Total	37	100.00

ca006_3_: XChildPanAliveName[3]'s Gender

	Freq.	%
1 Male	10	58.82
2 Female	7	41.18
Total	17	100.00

ca006_4_: XChildPanAliveName[4]'s Gender

	Freq.	%
1 Male	3	50.00
2 Female	3	50.00
Total	6	100.00

ca006_5_: XChildPanAliveName[5]'s Gender

	Freq.	%
2 Female	1	100.00
Total	1	100.00

ca006_6_: XChildPanAliveName[6]'s Gender

	Freq.	%
2 Female	1	100.00
Total	1	100.00

ca006_7_: XChildPanAliveName[7]'s Gender

	Freq.	%
1 Male	1	100.00
Total	1	100.00

ca007_1_: XChildPanAliveName[1]'s Highest Degree

	Freq.	%
1 No Formal Education(Illiterate)	14	1.74
2 Did not Finish Primary School	41	5.09
4 Elementary School	94	11.66
5 Middle School	194	24.07
6 High School	97	12.03
7 Vocational School	71	8.81
8 Two-/Three-Year College/Associate Degree	112	13.90
9 Four-Year College/Bachelor's Degree	142	17.62
10 Master's Degree	21	2.61
11 Doctoral Degree/Ph.D.	1	0.12
997 Don't Know	18	2.23
999 Refuse	1	0.12
Total	806	100.00

ca007_2_: XChildPanAliveName[2]'s Highest Degree

	Freq.	%
1 No Formal Education(Illiterate)	17	2.50
2 Did not Finish Primary School	56	8.25
4 Elementary School	101	14.87
5 Middle School	209	30.78
6 High School	73	10.75
7 Vocational School	44	6.48
8 Two-/Three-Year College/Associate Degree	62	9.13
9 Four-Year College/Bachelor's Degree	84	12.37
10 Master's Degree	11	1.62
11 Doctoral Degree/Ph.D.	2	0.29
997 Don't Know	18	2.65
999 Refuse	2	0.29
Total	679	100.00

ca007_3_: XChildPanAliveName[3]'s Highest Degree

	Freq.	%
1 No Formal Education(Illiterate)	26	7.01
2 Did not Finish Primary School	46	12.40
4 Elementary School	72	19.41
5 Middle School	106	28.57
6 High School	44	11.86

	Freq.	%
7 Vocational School	21	5.66
8 Two-/Three-Year College/Associate Degree	25	6.74
9 Four-Year College/Bachelor's Degree	18	4.85
10 Master's Degree	3	0.81
997 Don't Know	9	2.43
999 Refuse	1	0.27
Total	371	100.00

ca007_4_: XChildPanAliveName[4]'s Highest Degree

	Freq.	%
1 No Formal Education(Illiterate)	15	6.52
2 Did not Finish Primary School	30	13.04
3 Sishu/Home School	1	0.43
4 Elementary School	56	24.35
5 Middle School	47	20.43
6 High School	19	8.26
7 Vocational School	13	5.65
8 Two-/Three-Year College/Associate Degree	19	8.26
9 Four-Year College/Bachelor's Degree	13	5.65
11 Doctoral Degree/Ph.D.	1	0.43
997 Don't Know	15	6.52
999 Refuse	1	0.43
Total	230	100.00

ca007_5_: XChildPanAliveName[5]'s Highest Degree

	Freq.	%
1 No Formal Education(Illiterate)	10	8.47
2 Did not Finish Primary School	24	20.34
4 Elementary School	23	19.49
5 Middle School	22	18.64
6 High School	11	9.32
7 Vocational School	3	2.54
8 Two-/Three-Year College/Associate Degree	6	5.08
9 Four-Year College/Bachelor's Degree	6	5.08
10 Master's Degree	1	0.85
11 Doctoral Degree/Ph.D.	1	0.85
997 Don't Know	11	9.32
Total	118	100.00

ca007_6_: XChildPanAliveName[6]'s Highest Degree

	Freq.	%
1 No Formal Education(Illiterate)	12	25.53
2 Did not Finish Primary School	1	2.13
4 Elementary School	8	17.02
5 Middle School	10	21.28
6 High School	3	6.38
7 Vocational School	4	8.51
9 Four-Year College/Bachelor's Degree	1	2.13
997 Don't Know	8	17.02
Total	47	100.00

ca007_7_: XChildPanAliveName[7]'s Highest Degree

	Freq.	%
1 No Formal Education(Illiterate)	9	30.00
2 Did not Finish Primary School	2	6.67
4 Elementary School	4	13.33
5 Middle School	8	26.67
6 High School	3	10.00
7 Vocational School	1	3.33
9 Four-Year College/Bachelor's Degree	1	3.33
997 Don't Know	2	6.67
Total	30	100.00

ca007_8_: XChildPanAliveName[8]'s Highest Degree

	Freq.	%
1 No Formal Education(Illiterate)	1	14.29
4 Elementary School	2	28.57
5 Middle School	1	14.29
8 Two-/Three-Year College/Associate Degree	1	14.29
997 Don't Know	2	28.57
Total	7	100.00

ca007_9_: XChildPanAliveName[9]'s Highest Degree

	Freq.	%
1 No Formal Education(Illiterate)	2	50.00
5 Middle School	1	25.00
997 Don't Know	1	25.00
Total	4	100.00

ca007_10_: XChildPanAliveName[10]'s Highest Degree

	Freq.	%
5 Middle School	2	50.00
8 Two-/Three-Year College/Associate Degree	2	50.00
Total	4	100.00

ca007_11_: XChildPanAliveName[11]'s Highest Degree

	Freq.	%
4 Elementary School	2	50.00
5 Middle School	1	25.00
997 Don't Know	1	25.00
Total	4	100.00

ca007_12_: XChildPanAliveName[12]'s Highest Degree

	Freq.	%
5 Middle School	1	100.00
Total	1	100.00

ca007_13_: XChildPanAliveName[13]'s Highest Degree

	Freq.	%
4 Elementary School	1	100.00
Total	1	100.00

ca007_14_: XChildPanAliveName[14]'s Highest Degree

No Observations

ca007_15_: XChildPanAliveName[15]'s Highest Degree

No Observations

ca007_16_: XChildPanAliveName[16]'s Highest Degree

No Observations

ca007_17_: XChildPanAliveName[17]'s Highest Degree

	Freq.	%
1 No Formal Education(Illiterate)	1	100.00
Total	1	100.00

ca008_1_: Is XChildAliveName[1] Working or A Student

	Freq.	%
1 Working	8,899	82.97
2 Student	308	2.87
3 A Student Working Part-time or Full-time	24	0.22
4 Neither A Student Nor Dose She/He Work	1,448	13.50
997 Don't Know	44	0.41
999 Refuse	2	0.02
Total	10,725	100.00

ca008_2_: Is XChildAliveName[2] Working or A Student

	Freq.	%
1 Working	6,842	77.46
2 Student	456	5.16
3 A Student Working Part-time or Full-time	20	0.23
4 Neither A Student Nor Dose She/He Work	1,456	16.48
997 Don't Know	55	0.62
999 Refuse	4	0.05
Total	8,833	100.00

ca008_3_: Is XChildAliveName[3] Working or A Student

	Freq.	%
1 Working	3,719	76.90

	Freq.	%
2 Student	141	2.92
3 A Student Working Part-time or Full-time	2	0.04
4 Neither A Student Nor Dose She/He Work	927	19.17
997 Don't Know	45	0.93
999 Refuse	2	0.04
Total	4,836	100.00

ca008_4_: Is XChildAliveName[4] Working or A Student

	Freq.	%
1 Working	1,922	76.88
2 Student	40	1.60
3 A Student Working Part-time or Full-time	2	0.08
4 Neither A Student Nor Dose She/He Work	505	20.20
997 Don't Know	30	1.20
999 Refuse	1	0.04
Total	2,500	100.00

ca008_5_: Is XChildAliveName[5] Working or A Student

	Freq.	%
1 Working	858	73.90
2 Student	11	0.95
3 A Student Working Part-time or Full-time	2	0.17
4 Neither A Student Nor Dose She/He Work	267	23.00
997 Don't Know	23	1.98
Total	1,161	100.00

ca008_6_: Is XChildAliveName[6] Working or A Student

	Freq.	%
1 Working	362	73.73
2 Student	2	0.41
4 Neither A Student Nor Dose She/He Work	118	24.03
997 Don't Know	9	1.83
Total	491	100.00

ca008_7_: Is XChildAliveName[7] Working or A Student

	Freq.	%
1 Working	163	72.77
2 Student	4	1.79
4 Neither A Student Nor Dose She/He Work	53	23.66
997 Don't Know	4	1.79
Total	224	100.00

ca008_8_: Is XChildAliveName[8] Working or A Student

	Freq.	%
1 Working	63	69.23
2 Student	2	2.20
4 Neither A Student Nor Dose She/He Work	23	25.27

997 Don't Know	3	3.30
Total	91	100.00

ca008_9_: Is XChildAliveName[9] Working or A Student

	Freq.	%
1 Working	27	75.00
4 Neither A Student Nor Dose She/He Work	8	22.22
997 Don't Know	1	2.78
Total	36	100.00

ca008_10_: Is XChildAliveName[10] Working or A Student

	Freq.	%
1 Working	15	78.95
4 Neither A Student Nor Dose She/He Work	3	15.79
997 Don't Know	1	5.26
Total	19	100.00

ca008_11_: Is XChildAliveName[11] Working or A Student

	Freq.	%
1 Working	5	62.50
4 Neither A Student Nor Dose She/He Work	2	25.00
997 Don't Know	1	12.50
Total	8	100.00

ca008_12_: Is XChildAliveName[12] Working or A Student

	Freq.	%
1 Working	1	33.33
4 Neither A Student Nor Dose She/He Work	2	66.67
Total	3	100.00

ca008_13_: Is XChildAliveName[13] Working or A Student

	Freq.	%
1 Working	1	100.00
Total	1	100.00

ca008_14_: Is XChildAliveName[14] Working or A Student

No Observations

ca008_15_: Is XChildAliveName[15] Working or A Student

No Observations

ca008_16_: Is XChildAliveName[16] Working or A Student

No Observations

ca008_17_: Is XChildAliveName[17] Working or A Student

	Freq.	%
1 Working	1	100.00
Total	1	100.00

ca010_1_: XChildPanAliveName[1]'s Marital Status

	Freq.	%
1 Married with Spouse Present	7,307	68.44
2 Married but not Living With Spouse Temporarily for Reasons Such as Work	886	8.30
3 Separated	81	0.76
4 Divorced	438	4.10
5 Widowed	90	0.84
6 Never Married	1,841	17.24
997 Don't Know	32	0.30
999 Refuse	1	0.01
Total	10,676	100.00

ca010_2_: XChildPanAliveName[2]'s Marital Status

	Freq.	%
1 Married with Spouse Present	6,082	70.17
2 Married but not Living With Spouse Temporarily for Reasons Such as Work	794	9.16
3 Separated	51	0.59
4 Divorced	334	3.85
5 Widowed	78	0.90
6 Never Married	1,280	14.77
997 Don't Know	44	0.51
999 Refuse	5	0.06
Total	8,668	100.00

ca010_3_: XChildPanAliveName[3]'s Marital Status

	Freq.	%
1 Married with Spouse Present	3,650	76.46
2 Married but not Living With Spouse Temporarily for Reasons Such as Work	431	9.03
3 Separated	26	0.54
4 Divorced	171	3.58
5 Widowed	60	1.26
6 Never Married	398	8.34
997 Don't Know	35	0.73
999 Refuse	3	0.06
Total	4,774	100.00

ca010_4_: XChildPanAliveName[4]'s Marital Status

	Freq.	%
1 Married with Spouse Present	1,917	77.24
2 Married but not Living With Spouse Temporarily for Reasons Such as Work	241	9.71
3 Separated	15	0.60
4 Divorced	82	3.30
5 Widowed	48	1.93
6 Never Married	149	6.00
997 Don't Know	29	1.17
999 Refuse	1	0.04
Total	2,482	100.00

ca010_5_: XChildPanAliveName[5]'s Marital Status

	Freq.	%
1 Married with Spouse Present	926	79.83
2 Married but not Living With Spouse Temporarily for Reasons Such as Work	101	8.71
3 Separated	6	0.52
4 Divorced	41	3.53
5 Widowed	27	2.33
6 Never Married	50	4.31
997 Don't Know	9	0.78
Total	1,160	100.00

ca010_6_: XChildPanAliveName[6]'s Marital Status

	Freq.	%
1 Married with Spouse Present	394	80.41
2 Married but not Living With Spouse Temporarily for Reasons Such as Work	28	5.71
3 Separated	3	0.61
4 Divorced	20	4.08
5 Widowed	15	3.06
6 Never Married	18	3.67
997 Don't Know	12	2.45
Total	490	100.00

ca010_7_: XChildPanAliveName[7]'s Marital Status

	Freq.	%
1 Married with Spouse Present	182	81.98
2 Married but not Living With Spouse Temporarily for Reasons Such as Work	11	4.95
3 Separated	2	0.90
4 Divorced	7	3.15
5 Widowed	6	2.70
6 Never Married	10	4.50
997 Don't Know	4	1.80
Total	222	100.00

ca010_8_: XChildPanAliveName[8]'s Marital Status

	Freq.	%
1 Married with Spouse Present	73	82.95
2 Married but not Living With Spouse Temporarily for Reasons Such as Work	4	4.55
4 Divorced	3	3.41
5 Widowed	3	3.41
6 Never Married	3	3.41
997 Don't Know	2	2.27
Total	88	100.00

ca010_9_: XChildPanAliveName[9]'s Marital Status

	Freq.	%
1 Married with Spouse Present	28	77.78
2 Married but not Living With Spouse Temporarily for Reasons Such as Work	3	8.33
5 Widowed	3	8.33
6 Never Married	1	2.78
997 Don't Know	1	2.78
Total	36	100.00

ca010_10_: XChildPanAliveName[10]'s Marital Status

	Freq.	%
1 Married with Spouse Present	15	78.95
2 Married but not Living With Spouse Temporarily for Reasons Such as Work	2	10.53
6 Never Married	1	5.26
997 Don't Know	1	5.26
Total	19	100.00

ca010_11_: XChildPanAliveName[11]'s Marital Status

	Freq.	%
1 Married with Spouse Present	6	75.00
2 Married but not Living With Spouse Temporarily for Reasons Such as Work	1	12.50
997 Don't Know	1	12.50
Total	8	100.00

ca010_12_: XChildPanAliveName[12]'s Marital Status

	Freq.	%
1 Married with Spouse Present	3	100.00
Total	3	100.00

ca010_13_: XChildPanAliveName[13]'s Marital Status

	Freq.	%
1 Married with Spouse Present	1	100.00

Total	1	100.00

ca010_14_: XChildPanAliveName[14]'s Marital Status

No Observations

ca010_15_: XChildPanAliveName[15]'s Marital Status

No Observations

ca010_16_: XChildPanAliveName[16]'s Marital Status

No Observations

ca010_17_: XChildPanAliveName[17]'s Marital Status

	Freq.	%
1 Married with Spouse Present	1	100.00
Total	1	100.00

ca012_1_: XChildCoupleDis[1]'s Annual Income

	Freq.	%
1 No Income	649	6.08
2 Less Than 2000 Yuan	85	0.80
3 2000-5000 Yuan	312	2.92
4 5000-10000 Yuan	438	4.10
5 10000-20000 Yuan	1,038	9.72
6 20000-30000 Yuan	1,308	12.25
7 30000-50000 Yuan	1,826	17.10
8 50000-100000 Yuan	1,850	17.33
9 100000-150000 Yuan	592	5.55
10 150000-200000 Yuan	158	1.48
11 200000-300000 Yuan	121	1.13
12 More Than 300000 Yuan	96	0.90
997 Don't Know	2,193	20.54
999 Refuse	10	0.09
Total	10,676	100.00

ca012_2_: XChildCoupleDis[2]'s Annual Income

	Freq.	%
1 No Income	582	6.71
2 Less Than 2000 Yuan	60	0.69
3 2000-5000 Yuan	196	2.26
4 5000-10000 Yuan	302	3.48
5 10000-20000 Yuan	788	9.09
6 20000-30000 Yuan	976	11.26
7 30000-50000 Yuan	1,332	15.37
8 50000-100000 Yuan	1,371	15.82
9 100000-150000 Yuan	486	5.61
10 150000-200000 Yuan	150	1.73

11 200000-300000 Yuan	103	1.19
12 More Than 300000 Yuan	75	0.87
997 Don't Know	2,235	25.78
999 Refuse	12	0.14
Total	8,668	100.00

ca012_3_: XChildCoupleDis[3]'s Annual Income

	Freq.	%
1 No Income	220	4.61
2 Less Than 2000 Yuan	50	1.05
3 2000-5000 Yuan	108	2.26
4 5000-10000 Yuan	182	3.81
5 10000-20000 Yuan	427	8.94
6 20000-30000 Yuan	457	9.57
7 30000-50000 Yuan	725	15.19
8 50000-100000 Yuan	734	15.37
9 100000-150000 Yuan	278	5.82
10 150000-200000 Yuan	74	1.55
11 200000-300000 Yuan	49	1.03
12 More Than 300000 Yuan	36	0.75
997 Don't Know	1,429	29.93
999 Refuse	5	0.10
Total	4,774	100.00

ca012_4_: XChildCoupleDis[4]'s Annual Income

	Freq.	%
1 No Income	76	3.06
2 Less Than 2000 Yuan	18	0.73
3 2000-5000 Yuan	66	2.66
4 5000-10000 Yuan	102	4.11
5 10000-20000 Yuan	220	8.86
6 20000-30000 Yuan	267	10.76
7 30000-50000 Yuan	388	15.63
8 50000-100000 Yuan	320	12.89
9 100000-150000 Yuan	113	4.55
10 150000-200000 Yuan	51	2.05
11 200000-300000 Yuan	25	1.01
12 More Than 300000 Yuan	22	0.89
997 Don't Know	810	32.63
999 Refuse	4	0.16
Total	2,482	100.00

ca012_5_: XChildCoupleDis[5]'s Annual Income

	Freq.	%
1 No Income	51	4.40
2 Less Than 2000 Yuan	10	0.86
3 2000-5000 Yuan	41	3.53
4 5000-10000 Yuan	51	4.40
5 10000-20000 Yuan	114	9.83
6 20000-30000 Yuan	107	9.22
7 30000-50000 Yuan	150	12.93
8 50000-100000 Yuan	140	12.07

	Freq.	%
9 100000-150000 Yuan	55	4.74
10 150000-200000 Yuan	17	1.47
11 200000-300000 Yuan	4	0.34
12 More Than 300000 Yuan	6	0.52
997 Don't Know	414	35.69
Total	1,160	100.00

ca012_6_: XChildCoupleDis[6]'s Annual Income

	Freq.	%
1 No Income	19	3.88
2 Less Than 2000 Yuan	5	1.02
3 2000-5000 Yuan	15	3.06
4 5000-10000 Yuan	19	3.88
5 10000-20000 Yuan	43	8.78
6 20000-30000 Yuan	57	11.63
7 30000-50000 Yuan	64	13.06
8 50000-100000 Yuan	51	10.41
9 100000-150000 Yuan	21	4.29
10 150000-200000 Yuan	7	1.43
11 200000-300000 Yuan	3	0.61
12 More Than 300000 Yuan	2	0.41
997 Don't Know	184	37.55
Total	490	100.00

ca012_7_: XChildCoupleDis[7]'s Annual Income

	Freq.	%
1 No Income	11	4.95
2 Less Than 2000 Yuan	1	0.45
3 2000-5000 Yuan	9	4.05
4 5000-10000 Yuan	12	5.41
5 10000-20000 Yuan	20	9.01
6 20000-30000 Yuan	22	9.91
7 30000-50000 Yuan	25	11.26
8 50000-100000 Yuan	24	10.81
9 100000-150000 Yuan	12	5.41
10 150000-200000 Yuan	4	1.80
11 200000-300000 Yuan	1	0.45
997 Don't Know	81	36.49
Total	222	100.00

ca012_8_: XChildCoupleDis[8]'s Annual Income

	Freq.	%
1 No Income	6	6.82
2 Less Than 2000 Yuan	2	2.27
3 2000-5000 Yuan	3	3.41
4 5000-10000 Yuan	3	3.41
5 10000-20000 Yuan	7	7.95
6 20000-30000 Yuan	6	6.82
7 30000-50000 Yuan	6	6.82
8 50000-100000 Yuan	8	9.09
9 100000-150000 Yuan	5	5.68
10 150000-200000 Yuan	1	1.14

	Freq.	%
997 Don't Know	41	46.59
Total	88	100.00

ca012_9_: XChildCoupleDis[9]'s Annual Income

	Freq.	%
1 No Income	1	2.78
2 Less Than 2000 Yuan	2	5.56
3 2000-5000 Yuan	2	5.56
4 5000-10000 Yuan	3	8.33
5 10000-20000 Yuan	3	8.33
6 20000-30000 Yuan	2	5.56
7 30000-50000 Yuan	1	2.78
8 50000-100000 Yuan	4	11.11
9 100000-150000 Yuan	1	2.78
10 150000-200000 Yuan	1	2.78
997 Don't Know	16	44.44
Total	36	100.00

ca012_10_: XChildCoupleDis[10]'s Annual Income

	Freq.	%
1 No Income	1	5.26
4 5000-10000 Yuan	2	10.53
5 10000-20000 Yuan	1	5.26
7 30000-50000 Yuan	1	5.26
8 50000-100000 Yuan	6	31.58
9 100000-150000 Yuan	1	5.26
997 Don't Know	7	36.84
Total	19	100.00

ca012_11_: XChildCoupleDis[11]'s Annual Income

	Freq.	%
4 5000-10000 Yuan	1	12.50
7 30000-50000 Yuan	1	12.50
8 50000-100000 Yuan	1	12.50
9 100000-150000 Yuan	1	12.50
997 Don't Know	4	50.00
Total	8	100.00

ca012_12_: XChildCoupleDis[12]'s Annual Income

	Freq.	%
7 30000-50000 Yuan	1	33.33
8 50000-100000 Yuan	1	33.33
997 Don't Know	1	33.33
Total	3	100.00

ca012_13_: XChildCoupleDis[13]'s Annual Income

	Freq.	%

997 Don't Know	1	100.00
Total	1	100.00

ca012_14_: XChildCoupleDis[14]'s Annual Income

No Observations

ca012_15_: XChildCoupleDis[15]'s Annual Income

No Observations

ca012_16_: XChildCoupleDis[16]'s Annual Income

No Observations

ca012_17_: XChildCoupleDis[17]'s Annual Income

	Freq.	%
997 Don't Know	1	100.00
Total	1	100.00

ca013_1_: XChildPanAliveName[1]'s Health Status

	Freq.	%
1 Very Good	3,430	31.96
2 Good	2,524	23.52
3 Fair	4,061	37.84
4 Poor	483	4.50
5 Very Poor	151	1.41
997 Don't Know	80	0.75
999 Refuse	4	0.04
Total	10,733	100.00

ca013_2_: XChildPanAliveName[2]'s Health Status

	Freq.	%
1 Very Good	2,866	32.43
2 Good	2,144	24.26
3 Fair	3,174	35.91
4 Poor	451	5.10
5 Very Poor	120	1.36
997 Don't Know	79	0.89
999 Refuse	4	0.05
Total	8,838	100.00

ca013_3_: XChildPanAliveName[3]'s Health Status

	Freq.	%
1 Very Good	1,339	27.65
2 Good	1,204	24.86

3 Fair	1,892	39.07
4 Poor	268	5.53
5 Very Poor	72	1.49
997 Don't Know	65	1.34
999 Refuse	3	0.06
Total	4,843	100.00

ca013_4_: XChildPanAliveName[4]'s Health Status

	Freq.	%
1 Very Good	632	25.21
2 Good	650	25.93
3 Fair	1,002	39.97
4 Poor	151	6.02
5 Very Poor	30	1.20
997 Don't Know	40	1.60
999 Refuse	2	0.08
Total	2,507	100.00

ca013_5_: XChildPanAliveName[5]'s Health Status

	Freq.	%
1 Very Good	268	23.04
2 Good	275	23.65
3 Fair	485	41.70
4 Poor	84	7.22
5 Very Poor	22	1.89
997 Don't Know	29	2.49
Total	1,163	100.00

ca013_6_: XChildPanAliveName[6]'s Health Status

	Freq.	%
1 Very Good	106	21.50
2 Good	129	26.17
3 Fair	191	38.74
4 Poor	38	7.71
5 Very Poor	10	2.03
997 Don't Know	19	3.85
Total	493	100.00

ca013_7_: XChildPanAliveName[7]'s Health Status

	Freq.	%
1 Very Good	54	23.89
2 Good	62	27.43
3 Fair	89	39.38
4 Poor	10	4.42
5 Very Poor	4	1.77
997 Don't Know	7	3.10
Total	226	100.00

ca013_8_: XChildPanAliveName[8]'s Health Status

	Freq.	%
1 Very Good	18	19.78
2 Good	30	32.97
3 Fair	34	37.36
4 Poor	4	4.40
5 Very Poor	2	2.20
997 Don't Know	3	3.30
Total	91	100.00

ca013_9_: XChildPanAliveName[9]'s Health Status

	Freq.	%
1 Very Good	3	8.33
2 Good	12	33.33
3 Fair	13	36.11
4 Poor	3	8.33
5 Very Poor	4	11.11
997 Don't Know	1	2.78
Total	36	100.00

ca013_10_: XChildPanAliveName[10]'s Health Status

	Freq.	%
1 Very Good	5	26.32
2 Good	6	31.58
3 Fair	6	31.58
4 Poor	1	5.26
997 Don't Know	1	5.26
Total	19	100.00

ca013_11_: XChildPanAliveName[11]'s Health Status

	Freq.	%
1 Very Good	1	12.50
2 Good	1	12.50
3 Fair	4	50.00
4 Poor	1	12.50
997 Don't Know	1	12.50
Total	8	100.00

ca013_12_: XChildPanAliveName[12]'s Health Status

	Freq.	%
1 Very Good	1	33.33
3 Fair	2	66.67
Total	3	100.00

ca013_13_: XChildPanAliveName[13]'s Health Status

	Freq.	%

1 Very Good	1	100.00
Total	1	100.00

ca013_14_: XChildPanAliveName[14]'s Health Status

No Observations

ca013_15_: XChildPanAliveName[15]'s Health Status

No Observations

ca013_16_: XChildPanAliveName[16]'s Health Status

No Observations

ca013_17_: XChildPanAliveName[17]'s Health Status

	Freq.	%
1 Very Good	1	100.00
Total	1	100.00

ca014_1_: How Long XFamilyRAndS Lived With XChildPanAliveName[1]

Mean	SD	Min	Max	Obs
3.63	4.94	0.00	12.00	10,733

ca014_2_: How Long XFamilyRAndS Lived With XChildPanAliveName[2]

Mean	SD	Min	Max	Obs
2.23	4.10	0.00	12.00	8,838

ca014_3_: How Long XFamilyRAndS Lived With XChildPanAliveName[3]

Mean	SD	Min	Max	Obs
1.49	3.47	0.00	12.00	4,843

ca014_4_: How Long XFamilyRAndS Lived With XChildPanAliveName[4]

Mean	SD	Min	Max	Obs
1.23	3.17	0.00	12.00	2,507

ca014_5_: How Long XFamilyRAndS Lived With XChildPanAliveName[5]

Mean	SD	Min	Max	Obs
0.90	2.63	0.00	12.00	1,163

ca014_6_: How Long XFamilyRAndS Lived With XChildPanAliveName[6]

Mean	SD	Min	Max	Obs
0.74	2.49	0.00	12.00	493

ca014_7_: How Long XFamilyRAndS Lived With XChildPanAliveName[7]

Mean	SD	Min	Max	Obs
0.96	2.85	0.00	12.00	226

ca014_8_: How Long XFamilyRAndS Lived With XChildPanAliveName[8]

Mean	SD	Min	Max	Obs
0.86	2.67	0.00	12.00	91

ca014_9_: How Long XFamilyRAndS Lived With XChildPanAliveName[9]

Mean	SD	Min	Max	Obs
0.94	2.97	0.00	12.00	36

ca014_10_: How Long XFamilyRAndS Lived With XChildPanAliveName[10]

Mean	SD	Min	Max	Obs
1.21	3.34	0.00	12.00	19

ca014_11_: How Long XFamilyRAndS Lived With XChildPanAliveName[11]

Mean	SD	Min	Max	Obs
0.00	0.00	0.00	0.00	8

ca014_12_: How Long XFamilyRAndS Lived With XChildPanAliveName[12]

Mean	SD	Min	Max	Obs
0.00	0.00	0.00	0.00	3

ca014_13_: How Long XFamilyRAndS Lived With XChildPanAliveName[13]

Mean	SD	Min	Max	Obs
12.00	.	12.00	12.00	1

ca014_14_: How Long XFamilyRAndS Lived With XChildPanAliveName[14]

No Observations

ca014_15_: How Long XFamilyRAndS Lived With XChildPanAliveName[15]

No Observations

ca014_16_: How Long XFamilyRAndS Lived With XChildPanAliveName[16]

No Observations

ca014_17_: How Long XFamilyRAndS Lived With XChildPanAliveName[17]

Mean	SD	Min	Max	Obs
0.00	.	0.00	0.00	1

ca015_1_: Frequency See XChildPanAliveName[1]

	Freq.	%
1 Almost Every Day	955	11.49
2 2-3 Times a Week	484	5.82
3 Once a week	765	9.20
4 Every Two Weeks	750	9.02
5 Once a Month	1,049	12.62
6 Once Every Three Months	946	11.38
7 Once Every Six Months	1,056	12.70
8 Once a Year	1,426	17.16
9 Almost Never	451	5.43
10 Other	430	5.17
Total	8,312	100.00

ca015_2_: Frequency See XChildPanAliveName[2]

	Freq.	%
1 Almost Every Day	815	10.54
2 2-3 Times a Week	407	5.26
3 Once a week	737	9.53
4 Every Two Weeks	735	9.50
5 Once a Month	1,041	13.46
6 Once Every Three Months	839	10.85
7 Once Every Six Months	956	12.36
8 Once a Year	1,365	17.65
9 Almost Never	446	5.77
10 Other	393	5.08
Total	7,734	100.00

ca015_3_: Frequency See XChildPanAliveName[3]

	Freq.	%
1 Almost Every Day	476	10.70
2 2-3 Times a Week	249	5.60
3 Once a week	406	9.13
4 Every Two Weeks	417	9.38
5 Once a Month	587	13.20
6 Once Every Three Months	456	10.25
7 Once Every Six Months	544	12.23
8 Once a Year	791	17.79
9 Almost Never	273	6.14
10 Other	248	5.58
Total	4,447	100.00

ca015_4_: Frequency See XChildPanAliveName[4]

	Freq.	%
1 Almost Every Day	216	9.22
2 2-3 Times a Week	130	5.55
3 Once a week	189	8.07
4 Every Two Weeks	218	9.30
5 Once a Month	335	14.30
6 Once Every Three Months	281	11.99
7 Once Every Six Months	284	12.12
8 Once a Year	378	16.13
9 Almost Never	155	6.62
10 Other	157	6.70
Total	2,343	100.00

ca015_5_: Frequency See XChildPanAliveName[5]

	Freq.	%
1 Almost Every Day	85	7.62
2 2-3 Times a Week	65	5.82
3 Once a week	91	8.15
4 Every Two Weeks	103	9.23
5 Once a Month	158	14.16
6 Once Every Three Months	138	12.37
7 Once Every Six Months	142	12.72
8 Once a Year	175	15.68
9 Almost Never	86	7.71
10 Other	73	6.54
Total	1,116	100.00

ca015_6_: Frequency See XChildPanAliveName[6]

	Freq.	%
1 Almost Every Day	46	9.68
2 2-3 Times a Week	25	5.26
3 Once a week	24	5.05
4 Every Two Weeks	31	6.53
5 Once a Month	54	11.37
6 Once Every Three Months	60	12.63
7 Once Every Six Months	62	13.05
8 Once a Year	89	18.74
9 Almost Never	46	9.68
10 Other	38	8.00
Total	475	100.00

ca015_7_: Frequency See XChildPanAliveName[7]

	Freq.	%
1 Almost Every Day	20	9.35
2 2-3 Times a Week	4	1.87
3 Once a week	14	6.54
4 Every Two Weeks	18	8.41
5 Once a Month	31	14.49
6 Once Every Three Months	16	7.48

7 Once Every Six Months	39	18.22
8 Once a Year	43	20.09
9 Almost Never	18	8.41
10 Other	11	5.14
Total	214	100.00

ca015_8_: Frequency See XChildPanAliveName[8]

	Freq.	%
1 Almost Every Day	2	2.30
2 2-3 Times a Week	10	11.49
3 Once a week	5	5.75
4 Every Two Weeks	9	10.34
5 Once a Month	7	8.05
6 Once Every Three Months	15	17.24
7 Once Every Six Months	13	14.94
8 Once a Year	15	17.24
9 Almost Never	7	8.05
10 Other	4	4.60
Total	87	100.00

ca015_9_: Frequency See XChildPanAliveName[9]

	Freq.	%
1 Almost Every Day	1	2.94
2 2-3 Times a Week	3	8.82
3 Once a week	3	8.82
4 Every Two Weeks	3	8.82
5 Once a Month	2	5.88
6 Once Every Three Months	8	23.53
7 Once Every Six Months	3	8.82
8 Once a Year	8	23.53
9 Almost Never	1	2.94
10 Other	2	5.88
Total	34	100.00

ca015_10_: Frequency See XChildPanAliveName[10]

	Freq.	%
1 Almost Every Day	2	11.11
3 Once a week	1	5.56
4 Every Two Weeks	1	5.56
5 Once a Month	3	16.67
6 Once Every Three Months	3	16.67
7 Once Every Six Months	1	5.56
8 Once a Year	6	33.33
10 Other	1	5.56
Total	18	100.00

ca015_11_: Frequency See XChildPanAliveName[11]

	Freq.	%
1 Almost Every Day	1	12.50

	Freq.	%
2 2-3 Times a Week	1	12.50
3 Once a week	1	12.50
4 Every Two Weeks	1	12.50
5 Once a Month	1	12.50
6 Once Every Three Months	1	12.50
10 Other	2	25.00
Total	8	100.00

ca015_12_: Frequency See XChildPanAliveName[12]

	Freq.	%
3 Once a week	1	33.33
5 Once a Month	1	33.33
8 Once a Year	1	33.33
Total	3	100.00

ca015_13_: Frequency See XChildPanAliveName[13]

No Observations

ca015_14_: Frequency See XChildPanAliveName[14]

No Observations

ca015_15_: Frequency See XChildPanAliveName[15]

No Observations

ca015_16_: Frequency See XChildPanAliveName[16]

No Observations

ca015_17_: Frequency See XChildPanAliveName[17]

	Freq.	%
4 Every Two Weeks	1	100.00
Total	1	100.00

ca016_1_: Contact With XChildPanAliveName[1] By Phone/Message/Wechat/Mail/Email

	Freq.	%
1 Almost Every Day	818	13.39
2 2-3 Times a Week	1,078	17.65
3 Once a week	1,275	20.87
4 Every Two Weeks	945	15.47
5 Once a Month	803	13.15
6 Once Every Three Months	284	4.65
7 Once Every Six Months	105	1.72
8 Once a Year	25	0.41
9 Almost Never	494	8.09

10 Other	281	4.60
Total	6,108	100.00

ca016_2_: Contact With XChildPanAliveName[2] By Phone/Message/Wechat/Mail/Email

	Freq.	%
1 Almost Every Day	578	10.01
2 2-3 Times a Week	954	16.52
3 Once a week	1,164	20.16
4 Every Two Weeks	1,008	17.45
5 Once a Month	842	14.58
6 Once Every Three Months	260	4.50
7 Once Every Six Months	111	1.92
8 Once a Year	36	0.62
9 Almost Never	544	9.42
10 Other	278	4.81
Total	5,775	100.00

ca016_3_: Contact With XChildPanAliveName[3] By Phone/Message/Wechat/Mail/Email

	Freq.	%
1 Almost Every Day	206	6.21
2 2-3 Times a Week	409	12.33
3 Once a week	648	19.54
4 Every Two Weeks	595	17.94
5 Once a Month	572	17.25
6 Once Every Three Months	177	5.34
7 Once Every Six Months	65	1.96
8 Once a Year	27	0.81
9 Almost Never	417	12.58
10 Other	200	6.03
Total	3,316	100.00

ca016_4_: Contact With XChildPanAliveName[4] By Phone/Message/Wechat/Mail/Email

	Freq.	%
1 Almost Every Day	121	6.69
2 2-3 Times a Week	207	11.45
3 Once a week	272	15.04
4 Every Two Weeks	281	15.54
5 Once a Month	308	17.04
6 Once Every Three Months	101	5.59
7 Once Every Six Months	54	2.99
8 Once a Year	19	1.05
9 Almost Never	304	16.81
10 Other	141	7.80
Total	1,808	100.00

ca016_5_: Contact With XChildPanAliveName[5] By Phone/Message/Wechat/Mail/Email

	Freq.	%
1 Almost Every Day	50	5.71
2 2-3 Times a Week	82	9.37
3 Once a week	124	14.17
4 Every Two Weeks	131	14.97
5 Once a Month	159	18.17
6 Once Every Three Months	42	4.80
7 Once Every Six Months	25	2.86
8 Once a Year	4	0.46
9 Almost Never	198	22.63
10 Other	60	6.86
Total	875	100.00

ca016_6_: Contact With XChildPanAliveName[6] By Phone/Message/Wechat/Mail/Email

	Freq.	%
1 Almost Every Day	21	5.53
2 2-3 Times a Week	35	9.21
3 Once a week	38	10.00
4 Every Two Weeks	51	13.42
5 Once a Month	72	18.95
6 Once Every Three Months	22	5.79
7 Once Every Six Months	13	3.42
8 Once a Year	7	1.84
9 Almost Never	87	22.89
10 Other	34	8.95
Total	380	100.00

ca016_7_: Contact With XChildPanAliveName[7] By Phone/Message/Wechat/Mail/Email

	Freq.	%
1 Almost Every Day	7	3.98
2 2-3 Times a Week	12	6.82
3 Once a week	21	11.93
4 Every Two Weeks	14	7.95
5 Once a Month	31	17.61
6 Once Every Three Months	6	3.41
7 Once Every Six Months	6	3.41
8 Once a Year	1	0.57
9 Almost Never	63	35.80
10 Other	15	8.52
Total	176	100.00

ca016_8_: Contact With XChildPanAliveName[8] By Phone/Message/Wechat/Mail/Email

	Freq.	%
1 Almost Every Day	1	1.43
2 2-3 Times a Week	5	7.14
3 Once a week	3	4.29
4 Every Two Weeks	12	17.14
5 Once a Month	8	11.43

	Freq.	%
6 Once Every Three Months	4	5.71
7 Once Every Six Months	5	7.14
8 Once a Year	3	4.29
9 Almost Never	23	32.86
10 Other	6	8.57
Total	70	100.00

ca016_9_: Contact With XChildPanAliveName[9] By Phone/Message/Wechat/Mail/Email

	Freq.	%
2 2-3 Times a Week	1	3.70
4 Every Two Weeks	4	14.81
5 Once a Month	5	18.52
6 Once Every Three Months	3	11.11
7 Once Every Six Months	3	11.11
9 Almost Never	9	33.33
10 Other	2	7.41
Total	27	100.00

ca016_10_: Contact With XChildPanAliveName[10] By Phone/Message/Wechat/Mail/Email

	Freq.	%
3 Once a week	1	6.67
4 Every Two Weeks	1	6.67
5 Once a Month	3	20.00
7 Once Every Six Months	1	6.67
9 Almost Never	8	53.33
10 Other	1	6.67
Total	15	100.00

ca016_11_: Contact With XChildPanAliveName[11] By Phone/Message/Wechat/Mail/Email

	Freq.	%
1 Almost Every Day	2	40.00
9 Almost Never	2	40.00
10 Other	1	20.00
Total	5	100.00

ca016_12_: Contact With XChildPanAliveName[12] By Phone/Message/Wechat/Mail/Email

	Freq.	%
1 Almost Every Day	1	50.00
9 Almost Never	1	50.00
Total	2	100.00

ca016_13_: Contact With XChildPanAliveName[13] By Phone/Message/Wechat/Mail/Email

No Observations

ca016_14_: Contact With XChildPanAliveName[14] By Phone/Message/Wechat/Mail/Email

No Observations

ca016_15_: Contact With XChildPanAliveName[15] By Phone/Message/Wechat/Mail/Email

No Observations

ca016_16_: Contact With XChildPanAliveName[16] By Phone/Message/Wechat/Mail/Email

No Observations

ca016_17_: Contact With XChildPanAliveName[17] By Phone/Message/Wechat/Mail/Email

	Freq.	%
9 Almost Never	1	100.00
Total	1	100.00

ca017_1_1_: Total Money Received From XChildPanAliveName[1]

Mean	SD	Min	Max	Obs
2,339.03	7,686.55	0.00	300,000.00	8,136

ca017_1_2_: Total Money Received From XChildPanAliveName[2]

Mean	SD	Min	Max	Obs
1,771.21	5,834.90	0.00	203,000.00	7,569

ca017_1_3_: Total Money Received From XChildPanAliveName[3]

Mean	SD	Min	Max	Obs
1,418.26	5,223.25	0.00	200,000.00	4,349

ca017_1_4_: Total Money Received From XChildPanAliveName[4]

Mean	SD	Min	Max	Obs
1,295.43	5,395.10	0.00	200,000.00	2,281

ca017_1_5_: Total Money Received From XChildPanAliveName[5]

Mean	SD	Min	Max	Obs
969.49	2,698.02	0.00	60,000.00	1,085

ca017_1_6_: Total Money Received From XChildPanAliveName[6]

Mean	SD	Min	Max	Obs
724.95	2,025.85	0.00	24,000.00	461

ca017_1_7_: Total Money Received From XChildPanAliveName[7]

Mean	SD	Min	Max	Obs
976.18	3,422.82	0.00	40,000.00	207

ca017_1_8_: Total Money Received From XChildPanAliveName[8]

Mean	SD	Min	Max	Obs
797.59	2,731.17	0.00	24,000.00	83

ca017_1_9_: Total Money Received From XChildPanAliveName[9]

Mean	SD	Min	Max	Obs
466.67	651.76	0.00	2,000.00	33

ca017_1_10_: Total Money Received From XChildPanAliveName[10]

Mean	SD	Min	Max	Obs
483.33	651.02	0.00	2,000.00	18

ca017_1_11_: Total Money Received From XChildPanAliveName[11]

Mean	SD	Min	Max	Obs
1,228.57	1,402.04	0.00	4,000.00	7

ca017_1_12_: Total Money Received From XChildPanAliveName[12]

Mean	SD	Min	Max	Obs
1,500.00	1,322.88	0.00	2,500.00	3

ca017_1_13_: Total Money Received From XChildPanAliveName[13]

No Observations

ca017_1_14_: Total Money Received From XChildPanAliveName[14]

No Observations

ca017_1_15_: Total Money Received From XChildPanAliveName[15]

No Observations

ca017_1_16_: Total Money Received From XChildPanAliveName[16]

No Observations

ca017_1_17_: Total Money Received From XChildPanAliveName[17]

No Observations

ca017_1_min_1_: Min Bracket of ca017_1_1_

Mean	SD	Min	Max	Obs
2,338.78	4,280.04	-1.00	20,000.00	174

ca017_1_min_2_: Min Bracket of ca017_1_2_

Mean	SD	Min	Max	Obs
1,688.56	3,638.36	-1.00	20,000.00	162

ca017_1_min_3_: Min Bracket of ca017_1_3_

Mean	SD	Min	Max	Obs
1,415.12	3,119.80	-1.00	20,000.00	97

ca017_1_min_4_: Min Bracket of ca017_1_4_

Mean	SD	Min	Max	Obs
570.08	989.57	-1.00	5,000.00	61

ca017_1_min_5_: Min Bracket of ca017_1_5_

Mean	SD	Min	Max	Obs
442.90	576.71	-1.00	1,500.00	30

ca017_1_min_6_: Min Bracket of ca017_1_6_

Mean	SD	Min	Max	Obs
128.00	205.81	-1.00	500.00	14

ca017_1_min_7_: Min Bracket of ca017_1_7_

Mean	SD	Min	Max	Obs
99.43	182.94	-1.00	500.00	7

ca017_1_min_8_: Min Bracket of ca017_1_8_

Mean	SD	Min	Max	Obs
-1.00	0.00	-1.00	-1.00	4

ca017_1_min_9_: Min Bracket of ca017_1_9_

Mean	SD	Min	Max	Obs
-1.00	.	-1.00	-1.00	1

ca017_1_min_10_: Min Bracket of ca017_1_10_

No Observations

ca017_1_min_11_: Min Bracket of ca017_1_11_

Mean	SD	Min	Max	Obs
100.00	.	100.00	100.00	1

ca017_1_min_12_: Min Bracket of ca017_1_12_

No Observations

ca017_1_min_13_: Min Bracket of ca017_1_13_

No Observations

ca017_1_min_14_: Min Bracket of ca017_1_14_

No Observations

ca017_1_min_15_: Min Bracket of ca017_1_15_

No Observations

ca017_1_min_16_: Min Bracket of ca017_1_16_

No Observations

ca017_1_min_17_: Min Bracket of ca017_1_17_

Mean	SD	Min	Max	Obs
-1.00	.	-1.00	-1.00	1

ca017_1_max_1_: Max Bracket of ca017_1_1_

Mean	SD	Min	Max	Obs

4,228.93	6,134.70	-1.00	20,000.00	171

ca017_1_max_2_: Max Bracket of ca017_1_2_

Mean	SD	Min	Max	Obs
3,578.80	6,100.68	-1.00	20,000.00	163

ca017_1_max_3_: Max Bracket of ca017_1_3_

Mean	SD	Min	Max	Obs
3,370.09	6,269.70	-1.00	20,000.00	95

ca017_1_max_4_: Max Bracket of ca017_1_4_

Mean	SD	Min	Max	Obs
1,307.58	2,918.30	-1.00	20,000.00	62

ca017_1_max_5_: Max Bracket of ca017_1_5_

Mean	SD	Min	Max	Obs
1,973.81	3,845.98	-1.00	20,000.00	31

ca017_1_max_6_: Max Bracket of ca017_1_6_

Mean	SD	Min	Max	Obs
213.57	426.20	-1.00	1,500.00	14

ca017_1_max_7_: Max Bracket of ca017_1_7_

Mean	SD	Min	Max	Obs
285.00	567.37	-1.00	1,500.00	7

ca017_1_max_8_: Max Bracket of ca017_1_8_

Mean	SD	Min	Max	Obs
-1.00	0.00	-1.00	-1.00	4

ca017_1_max_9_: Max Bracket of ca017_1_9_

Mean	SD	Min	Max	Obs
-1.00	.	-1.00	-1.00	1

ca017_1_max_10_: Max Bracket of ca017_1_10_

No Observations

ca017_1_max_11_: Max Bracket of ca017_1_11_

Mean	SD	Min	Max	Obs
-1.00	.	-1.00	-1.00	1

ca017_1_max_12_: Max Bracket of ca017_1_12_

No Observations

ca017_1_max_13_: Max Bracket of ca017_1_13_

No Observations

ca017_1_max_14_: Max Bracket of ca017_1_14_

No Observations

ca017_1_max_15_: Max Bracket of ca017_1_15_

No Observations

ca017_1_max_16_: Max Bracket of ca017_1_16_

No Observations

ca017_1_max_17_: Max Bracket of ca017_1_17_

Mean	SD	Min	Max	Obs
-1.00	.	-1.00	-1.00	1

ca017_2_1_: Regular Payment Received from XChildPanAliveName[1]

Mean	SD	Min	Max	Obs
766.56	3,827.10	0.00	120,000.00	8,240

ca017_2_2_: Regular Payment Received from XChildPanAliveName[2]

Mean	SD	Min	Max	Obs
574.82	2,476.02	0.00	60,000.00	7,673

ca017_2_3_: Regular Payment Received from XChildPanAliveName[3]

Mean	SD	Min	Max	Obs
459.83	1,808.81	0.00	40,000.00	4,408

ca017_2_4_: Regular Payment Received from XChildPanAliveName[4]

Mean	SD	Min	Max	Obs
453.01	1,986.81	0.00	36,000.00	2,319

ca017_2_5_: Regular Payment Received from XChildPanAliveName[5]

Mean	SD	Min	Max	Obs
232.94	861.73	0.00	18,000.00	1,105

ca017_2_6_: Regular Payment Received from XChildPanAliveName[6]

Mean	SD	Min	Max	Obs
239.08	1,254.92	0.00	24,000.00	468

ca017_2_7_: Regular Payment Received from XChildPanAliveName[7]

Mean	SD	Min	Max	Obs
539.38	2,994.28	0.00	40,000.00	210

ca017_2_8_: Regular Payment Received from XChildPanAliveName[8]

Mean	SD	Min	Max	Obs
427.59	2,587.52	0.00	24,000.00	87

ca017_2_9_: Regular Payment Received from XChildPanAliveName[9]

Mean	SD	Min	Max	Obs
166.67	409.78	0.00	2,000.00	33

ca017_2_10_: Regular Payment Received from XChildPanAliveName[10]

Mean	SD	Min	Max	Obs
150.00	478.05	0.00	2,000.00	18

ca017_2_11_: Regular Payment Received from XChildPanAliveName[11]

Mean	SD	Min	Max	Obs
185.71	328.78	0.00	800.00	7

ca017_2_12_: Regular Payment Received from XChildPanAliveName[12]

Mean	SD	Min	Max	Obs
266.67	461.88	0.00	800.00	3

ca017_2_13_: Regular Payment Received from XChildPanAliveName[13]

No Observations

ca017_2_14_: Regular Payment Received from XChildPanAliveName[14]

No Observations

ca017_2_15_: Regular Payment Received from XChildPanAliveName[15]

No Observations

ca017_2_16_: Regular Payment Received from XChildPanAliveName[16]

No Observations

ca017_2_17_: Regular Payment Received from XChildPanAliveName[17]

Mean	SD	Min	Max	Obs
0.00	.	0.00	0.00	1

ca017_2_min_1_: Min Bracket of ca017_2_1_

Mean	SD	Min	Max	Obs
693.73	1,482.32	-1.00	10,000.00	70

ca017_2_min_2_: Min Bracket of ca017_2_2_

Mean	SD	Min	Max	Obs
397.68	1,426.63	-1.00	10,000.00	60

ca017_2_min_3_: Min Bracket of ca017_2_3_

Mean	SD	Min	Max	Obs
358.81	841.40	-1.00	3,000.00	37

ca017_2_min_4_: Min Bracket of ca017_2_4_

Mean	SD	Min	Max	Obs
190.88	634.02	-1.00	3,000.00	24

ca017_2_min_5_: Min Bracket of ca017_2_5_

Mean	SD	Min	Max	Obs
281.00	902.30	-1.00	3,000.00	11

ca017_2_min_6_: Min Bracket of ca017_2_6_

Mean	SD	Min	Max	Obs
56.43	98.08	-1.00	200.00	7

ca017_2_min_7_: Min Bracket of ca017_2_7_

Mean	SD	Min	Max	Obs
299.50	476.52	-1.00	1,000.00	4

ca017_2_min_8_: Min Bracket of ca017_2_8_

No Observations

ca017_2_min_9_: Min Bracket of ca017_2_9_

Mean	SD	Min	Max	Obs
-1.00	.	-1.00	-1.00	1

ca017_2_min_10_: Min Bracket of ca017_2_10_

No Observations

ca017_2_min_11_: Min Bracket of ca017_2_11_

Mean	SD	Min	Max	Obs
-1.00	.	-1.00	-1.00	1

ca017_2_max_1_: Max Bracket of ca017_2_1_

Mean	SD	Min	Max	Obs
1,734.22	3,125.56	-1.00	10,000.00	72

ca017_2_max_2_: Max Bracket of ca017_2_2_

Mean	SD	Min	Max	Obs
455.98	1,436.71	-1.00	10,000.00	60

ca017_2_max_3_: Max Bracket of ca017_2_3_

Mean	SD	Min	Max	Obs
1,270.45	2,754.44	-1.00	10,000.00	38

ca017_2_max_4_: Max Bracket of ca017_2_4_

Mean	SD	Min	Max	Obs
624.17	2,102.28	-1.00	10,000.00	24

ca017_2_max_5_: Max Bracket of ca017_2_5_

Mean	SD	Min	Max	Obs
290.09	900.80	-1.00	3,000.00	11

ca017_2_max_6_: Max Bracket of ca017_2_6_

Mean	SD	Min	Max	Obs
142.00	378.34	-1.00	1,000.00	7

ca017_2_max_7_: Max Bracket of ca017_2_7_

Mean	SD	Min	Max	Obs
749.25	1,500.50	-1.00	3,000.00	4

ca017_2_max_8_: Max Bracket of ca017_2_8_

No Observations

ca017_2_max_9_: Max Bracket of ca017_2_9_

Mean	SD	Min	Max	Obs
-1.00	.	-1.00	-1.00	1

ca017_2_max_10_: Max Bracket of ca017_2_10_

No Observations

ca017_2_max_11_: Max Bracket of ca017_2_11_

Mean	SD	Min	Max	Obs
-1.00	.	-1.00	-1.00	1

ca017_3_1_: Total In-kind Payment Received From XChildPanAliveName[1]

Mean	SD	Min	Max	Obs
1,197.86	3,159.09	0.00	130,000.00	7,762

ca017_3_2_: Total In-kind Payment Received From XChildPanAliveName[2]

Mean	SD	Min	Max	Obs
878.75	2,305.63	0.00	102,200.00	7,261

ca017_3_3_: Total In-kind Payment Received From XChildPanAliveName[3]

Mean	SD	Min	Max	Obs

793.70	4,735.50	0.00	260,000.00	4,155

ca017_3_4_: Total In-kind Payment Received From XChildPanAliveName[4]

Mean	SD	Min	Max	Obs
567.75	1,166.38	0.00	20,000.00	2,170

ca017_3_5_: Total In-kind Payment Received From XChildPanAliveName[5]

Mean	SD	Min	Max	Obs
502.84	1,073.11	0.00	10,000.00	1,037

ca017_3_6_: Total In-kind Payment Received From XChildPanAliveName[6]

Mean	SD	Min	Max	Obs
370.70	736.81	0.00	6,000.00	446

ca017_3_7_: Total In-kind Payment Received From XChildPanAliveName[7]

Mean	SD	Min	Max	Obs
318.91	700.44	0.00	5,000.00	198

ca017_3_8_: Total In-kind Payment Received From XChildPanAliveName[8]

Mean	SD	Min	Max	Obs
539.07	2,243.64	0.00	20,000.00	81

ca017_3_9_: Total In-kind Payment Received From XChildPanAliveName[9]

Mean	SD	Min	Max	Obs
160.18	293.15	0.00	1,200.00	28

ca017_3_10_: Total In-kind Payment Received From XChildPanAliveName[10]

Mean	SD	Min	Max	Obs
336.39	553.13	0.00	2,000.00	18

ca017_3_11_: Total In-kind Payment Received From XChildPanAliveName[11]

Mean	SD	Min	Max	Obs
700.00	946.57	0.00	2,500.00	6

ca017_3_12_: Total In-kind Payment Received From XChildPanAliveName[12]

Mean	SD	Min	Max	Obs
433.33	513.16	0.00	1,000.00	3

ca017_3_13_: Total In-kind Payment Received From XChildPanAliveName[13]

No Observations

ca017_3_14_: Total In-kind Payment Received From XChildPanAliveName[14]

No Observations

ca017_3_15_: Total In-kind Payment Received From XChildPanAliveName[15]

No Observations

ca017_3_16_: Total In-kind Payment Received From XChildPanAliveName[16]

No Observations

ca017_3_17_: Total In-kind Payment Received From XChildPanAliveName[17]

No Observations

ca017_3_min_1_: Min Bracket of ca017_3_1_

Mean	SD	Min	Max	Obs
1,452.44	2,982.85	-1.00	20,000.00	528

ca017_3_min_2_: Min Bracket of ca017_3_2_

Mean	SD	Min	Max	Obs
1,012.21	2,497.51	-1.00	20,000.00	464

ca017_3_min_3_: Min Bracket of ca017_3_3_

Mean	SD	Min	Max	Obs
817.58	2,028.50	-1.00	20,000.00	285

ca017_3_min_4_: Min Bracket of ca017_3_4_

Mean	SD	Min	Max	Obs
686.59	1,858.29	-1.00	20,000.00	169

ca017_3_min_5_: Min Bracket of ca017_3_5_

Mean	SD	Min	Max	Obs
352.95	698.52	-1.00	5,000.00	75

ca017_3_min_6_: Min Bracket of ca017_3_6_

Mean	SD	Min	Max	Obs
454.76	987.83	-1.00	5,000.00	29

ca017_3_min_7_: Min Bracket of ca017_3_7_

Mean	SD	Min	Max	Obs
274.63	394.40	-1.00	1,500.00	16

ca017_3_min_8_: Min Bracket of ca017_3_8_

Mean	SD	Min	Max	Obs
349.50	596.17	-1.00	1,500.00	6

ca017_3_min_9_: Min Bracket of ca017_3_9_

Mean	SD	Min	Max	Obs
133.00	186.48	-1.00	500.00	6

ca017_3_min_10_: Min Bracket of ca017_3_10_

No Observations

ca017_3_min_11_: Min Bracket of ca017_3_11_

Mean	SD	Min	Max	Obs
749.50	1,061.37	-1.00	1,500.00	2

ca017_3_min_12_: Min Bracket of ca017_3_12_

No Observations

ca017_3_min_13_: Min Bracket of ca017_3_13_

No Observations

ca017_3_min_14_: Min Bracket of ca017_3_14_

No Observations

ca017_3_min_15_: Min Bracket of ca017_3_15_

No Observations

ca017_3_min_16_: Min Bracket of ca017_3_16_

No Observations

ca017_3_min_17_: Min Bracket of ca017_3_17_

Mean	SD	Min	Max	Obs
-1.00	.	-1.00	-1.00	1

ca017_3_max_1_: Max Bracket of ca017_3_1_

Mean	SD	Min	Max	Obs
2,923.32	5,079.80	-1.00	20,000.00	548

ca017_3_max_2_: Max Bracket of ca017_3_2_

Mean	SD	Min	Max	Obs
2,240.06	4,526.31	-1.00	20,000.00	471

ca017_3_max_3_: Max Bracket of ca017_3_3_

Mean	SD	Min	Max	Obs
2,152.25	4,588.72	-1.00	20,000.00	293

ca017_3_max_4_: Max Bracket of ca017_3_4_

Mean	SD	Min	Max	Obs
1,471.13	3,275.25	-1.00	20,000.00	172

ca017_3_max_5_: Max Bracket of ca017_3_5_

Mean	SD	Min	Max	Obs
792.04	1,320.46	-1.00	5,000.00	79

ca017_3_max_6_: Max Bracket of ca017_3_6_

Mean	SD	Min	Max	Obs
892.62	1,718.43	-1.00	5,000.00	29

ca017_3_max_7_: Max Bracket of ca017_3_7_

Mean	SD	Min	Max	Obs
536.94	1,255.58	-1.00	5,000.00	16

ca017_3_max_8_: Max Bracket of ca017_3_8_

Mean	SD	Min	Max	Obs

416.17	584.95	-1.00	1,500.00	6

ca017_3_max_9_: Max Bracket of ca017_3_9_

Mean	SD	Min	Max	Obs
1,199.83	1,943.32	-1.00	5,000.00	6

ca017_3_max_10_: Max Bracket of ca017_3_10_

No Observations

ca017_3_max_11_: Max Bracket of ca017_3_11_

Mean	SD	Min	Max	Obs
9,999.50	14,142.84	-1.00	20,000.00	2

ca017_3_max_12_: Max Bracket of ca017_3_12_

No Observations

ca017_3_max_13_: Max Bracket of ca017_3_13_

No Observations

ca017_3_max_14_: Max Bracket of ca017_3_14_

No Observations

ca017_3_max_15_: Max Bracket of ca017_3_15_

No Observations

ca017_3_max_16_: Max Bracket of ca017_3_16_

No Observations

ca017_3_max_17_: Max Bracket of ca017_3_17_

Mean	SD	Min	Max	Obs
-1.00	.	-1.00	-1.00	1

ca017_4_1_: Regular In-kind Payment Received From XChildPanAliveName[1]

Mean	SD	Min	Max	Obs
196.41	1,451.40	0.00	95,200.00	8,196

ca017_4_2_: Regular In-kind Payment Received From XChildPanAliveName[2]

Mean	SD	Min	Max	Obs
155.25	1,395.95	0.00	102,200.00	7,634

ca017_4_3_: Regular In-kind Payment Received From XChildPanAliveName[3]

Mean	SD	Min	Max	Obs
140.74	1,094.53	0.00	36,000.00	4,383

ca017_4_4_: Regular In-kind Payment Received From XChildPanAliveName[4]

Mean	SD	Min	Max	Obs
92.14	442.90	0.00	8,000.00	2,294

ca017_4_5_: Regular In-kind Payment Received From XChildPanAliveName[5]

Mean	SD	Min	Max	Obs
66.57	294.80	0.00	4,000.00	1,100

ca017_4_6_: Regular In-kind Payment Received From XChildPanAliveName[6]

Mean	SD	Min	Max	Obs
53.07	243.39	0.00	3,600.00	463

ca017_4_7_: Regular In-kind Payment Received From XChildPanAliveName[7]

Mean	SD	Min	Max	Obs
64.64	364.96	0.00	5,000.00	210

ca017_4_8_: Regular In-kind Payment Received From XChildPanAliveName[8]

Mean	SD	Min	Max	Obs
61.98	253.80	0.00	2,000.00	86

ca017_4_9_: Regular In-kind Payment Received From XChildPanAliveName[9]

Mean	SD	Min	Max	Obs
20.00	59.24	0.00	300.00	32

ca017_4_10_: Regular In-kind Payment Received From XChildPanAliveName[10]

Mean	SD	Min	Max	Obs
30.56	117.75	0.00	500.00	18

ca017_4_11_: Regular In-kind Payment Received From XChildPanAliveName[11]

Mean	SD	Min	Max	Obs
0.00	0.00	0.00	0.00	8

ca017_4_12_: Regular In-kind Payment Received From XChildPanAliveName[12]

Mean	SD	Min	Max	Obs
0.00	0.00	0.00	0.00	3

ca017_4_13_: Regular In-kind Payment Received From XChildPanAliveName[13]

No Observations

ca017_4_14_: Regular In-kind Payment Received From XChildPanAliveName[14]

No Observations

ca017_4_15_: Regular In-kind Payment Received From XChildPanAliveName[15]

No Observations

ca017_4_16_: Regular In-kind Payment Received From XChildPanAliveName[16]

No Observations

ca017_4_17_: Regular In-kind Payment Received From XChildPanAliveName[17]

Mean	SD	Min	Max	Obs
0.00	.	0.00	0.00	1

ca017_4_min_1_: Min Bracket of ca017_4_1_

Mean	SD	Min	Max	Obs
460.91	1,151.46	-1.00	10,000.00	114

ca017_4_min_2_: Min Bracket of ca017_4_2_

Mean	SD	Min	Max	Obs
312.59	752.41	-1.00	3,000.00	98

ca017_4_min_3_: Min Bracket of ca017_4_3_

Mean	SD	Min	Max	Obs
231.63	379.76	-1.00	1,000.00	59

ca017_4_min_4_: Min Bracket of ca017_4_4_

Mean	SD	Min	Max	Obs
677.59	2,113.98	-1.00	10,000.00	46

ca017_4_min_5_: Min Bracket of ca017_4_5_

Mean	SD	Min	Max	Obs
105.56	251.89	-1.00	1,000.00	16

ca017_4_min_6_: Min Bracket of ca017_4_6_

Mean	SD	Min	Max	Obs
382.75	871.89	-1.00	3,000.00	12

ca017_4_min_7_: Min Bracket of ca017_4_7_

Mean	SD	Min	Max	Obs
499.50	577.93	-1.00	1,000.00	4

ca017_4_min_8_: Min Bracket of ca017_4_8_

Mean	SD	Min	Max	Obs
1,000.00	.	1,000.00	1,000.00	1

ca017_4_min_9_: Min Bracket of ca017_4_9_

Mean	SD	Min	Max	Obs
99.50	142.13	-1.00	200.00	2

ca017_4_max_1_: Max Bracket of ca017_4_1_

Mean	SD	Min	Max	Obs
1,156.92	2,179.34	-1.00	10,000.00	115

ca017_4_max_2_: Max Bracket of ca017_4_2_

Mean	SD	Min	Max	Obs
537.71	1,580.51	-1.00	10,000.00	99

ca017_4_max_3_: Max Bracket of ca017_4_3_

Mean	SD	Min	Max	Obs
437.54	785.14	-1.00	3,000.00	63

ca017_4_max_4_: Max Bracket of ca017_4_4_

Mean	SD	Min	Max	Obs
422.72	1,523.24	-1.00	10,000.00	47

ca017_4_max_5_: Max Bracket of ca017_4_5_

Mean	SD	Min	Max	Obs
149.25	338.98	-1.00	1,000.00	16

ca017_4_max_6_: Max Bracket of ca017_4_6_

Mean	SD	Min	Max	Obs
849.42	1,327.40	-1.00	3,000.00	12

ca017_4_max_7_: Max Bracket of ca017_4_7_

Mean	SD	Min	Max	Obs
1,499.50	1,732.63	-1.00	3,000.00	4

ca017_4_max_8_: Max Bracket of ca017_4_8_

Mean	SD	Min	Max	Obs
3,000.00	.	3,000.00	3,000.00	1

ca017_4_max_9_: Max Bracket of ca017_4_9_

Mean	SD	Min	Max	Obs
499.50	707.81	-1.00	1,000.00	2

ca018_1_1_: Total Money Provided to XChildPanAliveName[1]

Mean	SD	Min	Max	Obs
4,227.60	35,136.53	0.00	2,000,000.00	8,217

ca018_1_2_: Total Money Provided to XChildPanAliveName[2]

Mean	SD	Min	Max	Obs
1,691.89	14,171.53	0.00	1,000,000.00	7,652

ca018_1_3_: Total Money Provided to XChildPanAliveName[3]

Mean	SD	Min	Max	Obs
978.89	13,803.91	0.00	801,000.00	4,418

ca018_1_4_: Total Money Provided to XChildPanAliveName[4]

Mean	SD	Min	Max	Obs
534.90	5,843.57	0.00	200,000.00	2,319

ca018_1_5_: Total Money Provided to XChildPanAliveName[5]

Mean	SD	Min	Max	Obs
259.60	1,978.63	0.00	40,000.00	1,110

ca018_1_6_: Total Money Provided to XChildPanAliveName[6]

Mean	SD	Min	Max	Obs
273.17	2,673.98	0.00	50,000.00	472

ca018_1_7_: Total Money Provided to XChildPanAliveName[7]

Mean	SD	Min	Max	Obs
508.28	4,666.25	0.00	65,000.00	211

ca018_1_8_: Total Money Provided to XChildPanAliveName[8]

Mean	SD	Min	Max	Obs
152.86	1,095.66	0.00	10,000.00	84

ca018_1_9_: Total Money Provided to XChildPanAliveName[9]

Mean	SD	Min	Max	Obs
15.15	61.85	0.00	300.00	33

ca018_1_10_: Total Money Provided to XChildPanAliveName[10]

Mean	SD	Min	Max	Obs
38.89	114.48	0.00	400.00	18

ca018_1_11_: Total Money Provided to XChildPanAliveName[11]

Mean	SD	Min	Max	Obs
100.00	173.21	0.00	400.00	7

ca018_1_12_: Total Money Provided to XChildPanAliveName[12]

Mean	SD	Min	Max	Obs
533.33	611.01	0.00	1,200.00	3

ca018_1_13_: Total Money Provided to XChildPanAliveName[13]

No Observations

ca018_1_14_: Total Money Provided to XChildPanAliveName[14]

No Observations

ca018_1_15_: Total Money Provided to XChildPanAliveName[15]

No Observations

ca018_1_16_: Total Money Provided to XChildPanAliveName[16]

No Observations

ca018_1_17_: Total Money Provided to XChildPanAliveName[17]

Mean	SD	Min	Max	Obs
0.00	.	0.00	0.00	1

ca018_1_min_1_: Min Bracket of ca018_1_1_

Mean	SD	Min	Max	Obs
2,610.21	5,840.07	-1.00	20,000.00	94

ca018_1_min_2_: Min Bracket of ca018_1_2_

Mean	SD	Min	Max	Obs
2,350.70	5,261.87	-1.00	20,000.00	82

ca018_1_min_3_: Min Bracket of ca018_1_3_

Mean	SD	Min	Max	Obs
2,503.27	5,455.58	-1.00	20,000.00	26

ca018_1_min_4_: Min Bracket of ca018_1_4_

Mean	SD	Min	Max	Obs
1,115.92	4,156.68	-1.00	20,000.00	24

ca018_1_min_5_: Min Bracket of ca018_1_5_

Mean	SD	Min	Max	Obs
832.50	2,041.65	-1.00	5,000.00	6

ca018_1_min_6_: Min Bracket of ca018_1_6_

Mean	SD	Min	Max	Obs
-1.00	0.00	-1.00	-1.00	3

ca018_1_min_7_: Min Bracket of ca018_1_7_

Mean	SD	Min	Max	Obs
-1.00	0.00	-1.00	-1.00	3

ca018_1_min_8_: Min Bracket of ca018_1_8_

Mean	SD	Min	Max	Obs
32.67	58.31	-1.00	100.00	3

ca018_1_min_9_: Min Bracket of ca018_1_9_

Mean	SD	Min	Max	Obs
-1.00	.	-1.00	-1.00	1

ca018_1_min_10_: Min Bracket of ca018_1_10_

No Observations

ca018_1_min_11_: Min Bracket of ca018_1_11_

Mean	SD	Min	Max	Obs
100.00	.	100.00	100.00	1

ca018_1_max_1_: Max Bracket of ca018_1_1_

Mean	SD	Min	Max	Obs
2,945.52	6,016.17	-1.00	20,000.00	87

ca018_1_max_2_: Max Bracket of ca018_1_2_

Mean	SD	Min	Max	Obs
2,357.84	5,403.07	-1.00	20,000.00	77

ca018_1_max_3_: Max Bracket of ca018_1_3_

Mean	SD	Min	Max	Obs
2,914.22	6,356.30	-1.00	20,000.00	27

ca018_1_max_4_: Max Bracket of ca018_1_4_

Mean	SD	Min	Max	Obs

999.22	4,156.24	-1.00	20,000.00	23

ca018_1_max_5_: Max Bracket of ca018_1_5_

Mean	SD	Min	Max	Obs
832.50	2,041.65	-1.00	5,000.00	6

ca018_1_max_6_: Max Bracket of ca018_1_6_

Mean	SD	Min	Max	Obs
-1.00	0.00	-1.00	-1.00	3

ca018_1_max_7_: Max Bracket of ca018_1_7_

Mean	SD	Min	Max	Obs
-1.00	0.00	-1.00	-1.00	3

ca018_1_max_8_: Max Bracket of ca018_1_8_

Mean	SD	Min	Max	Obs
166.00	289.25	-1.00	500.00	3

ca018_1_max_9_: Max Bracket of ca018_1_9_

Mean	SD	Min	Max	Obs
-1.00	.	-1.00	-1.00	1

ca018_1_max_10_: Max Bracket of ca018_1_10_

No Observations

ca018_1_max_11_: Max Bracket of ca018_1_11_

Mean	SD	Min	Max	Obs
500.00	.	500.00	500.00	1

ca018_2_1_: Regular Payment Provided to XChildPanAliveName[1]

Mean	SD	Min	Max	Obs
853.07	8,521.73	0.00	500,000.00	8,274

ca018_2_2_: Regular Payment Provided to XChildPanAliveName[2]

Mean	SD	Min	Max	Obs
471.15	2,935.21	0.00	126,000.00	7,701

ca018_2_3_: Regular Payment Provided to XChildPanAliveName[3]

Mean	SD	Min	Max	Obs
275.68	4,186.13	0.00	260,000.00	4,432

ca018_2_4_: Regular Payment Provided to XChildPanAliveName[4]

Mean	SD	Min	Max	Obs
97.72	906.52	0.00	30,000.00	2,328

ca018_2_5_: Regular Payment Provided to XChildPanAliveName[5]

Mean	SD	Min	Max	Obs
76.64	616.01	0.00	10,000.00	1,111

ca018_2_6_: Regular Payment Provided to XChildPanAliveName[6]

Mean	SD	Min	Max	Obs
38.81	230.10	0.00	4,000.00	472

ca018_2_7_: Regular Payment Provided to XChildPanAliveName[7]

Mean	SD	Min	Max	Obs
96.23	1,103.80	0.00	16,000.00	212

ca018_2_8_: Regular Payment Provided to XChildPanAliveName[8]

Mean	SD	Min	Max	Obs
29.07	130.04	0.00	1,000.00	86

ca018_2_9_: Regular Payment Provided to XChildPanAliveName[9]

Mean	SD	Min	Max	Obs
14.71	60.96	0.00	300.00	34

ca018_2_10_: Regular Payment Provided to XChildPanAliveName[10]

Mean	SD	Min	Max	Obs
38.89	114.48	0.00	400.00	18

ca018_2_11_: Regular Payment Provided to XChildPanAliveName[11]

Mean	SD	Min	Max	Obs
87.50	164.21	0.00	400.00	8

ca018_2_12_: Regular Payment Provided to XChildPanAliveName[12]

Mean	SD	Min	Max	Obs
133.33	230.94	0.00	400.00	3

ca018_2_13_: Regular Payment Provided to XChildPanAliveName[13]

No Observations

ca018_2_14_: Regular Payment Provided to XChildPanAliveName[14]

No Observations

ca018_2_15_: Regular Payment Provided to XChildPanAliveName[15]

No Observations

ca018_2_16_: Regular Payment Provided to XChildPanAliveName[16]

No Observations

ca018_2_17_: Regular Payment Provided to XChildPanAliveName[17]

Mean	SD	Min	Max	Obs
0.00	.	0.00	0.00	1

ca018_2_min_1_: Min Bracket of ca018_2_1_

Mean	SD	Min	Max	Obs
315.78	1,668.96	-1.00	10,000.00	36

ca018_2_min_2_: Min Bracket of ca018_2_2_

Mean	SD	Min	Max	Obs
308.15	1,740.21	-1.00	10,000.00	33

ca018_2_min_3_: Min Bracket of ca018_2_3_

Mean	SD	Min	Max	Obs
14.46	55.75	-1.00	200.00	13

ca018_2_min_4_: Min Bracket of ca018_2_4_

Mean	SD	Min	Max	Obs
-1.00	0.00	-1.00	-1.00	15

ca018_2_min_5_: Min Bracket of ca018_2_5_

Mean	SD	Min	Max	Obs
-1.00	0.00	-1.00	-1.00	5

ca018_2_min_6_: Min Bracket of ca018_2_6_

Mean	SD	Min	Max	Obs
-1.00	0.00	-1.00	-1.00	3

ca018_2_min_7_: Min Bracket of ca018_2_7_

Mean	SD	Min	Max	Obs
-1.00	0.00	-1.00	-1.00	2

ca018_2_min_8_: Min Bracket of ca018_2_8_

Mean	SD	Min	Max	Obs
-1.00	.	-1.00	-1.00	1

ca018_2_max_1_: Max Bracket of ca018_2_1_

Mean	SD	Min	Max	Obs
412.68	1,705.78	-1.00	10,000.00	37

ca018_2_max_2_: Max Bracket of ca018_2_2_

Mean	SD	Min	Max	Obs
30.28	176.95	-1.00	1,000.00	32

ca018_2_max_3_: Max Bracket of ca018_2_3_

Mean	SD	Min	Max	Obs
77.71	266.81	-1.00	1,000.00	14

ca018_2_max_4_: Max Bracket of ca018_2_4_

Mean	SD	Min	Max	Obs
-1.00	0.00	-1.00	-1.00	15

ca018_2_max_5_: Max Bracket of ca018_2_5_

Mean	SD	Min	Max	Obs
-1.00	0.00	-1.00	-1.00	5

ca018_2_max_6_: Max Bracket of ca018_2_6_

Mean	SD	Min	Max	Obs
-1.00	0.00	-1.00	-1.00	3

ca018_2_max_7_: Max Bracket of ca018_2_7_

Mean	SD	Min	Max	Obs
-1.00	0.00	-1.00	-1.00	2

ca018_2_max_8_: Max Bracket of ca018_2_8_

Mean	SD	Min	Max	Obs
-1.00	.	-1.00	-1.00	1

ca018_3_1_: Total In-kind Payment Provided to XChildPanAliveName[1]

Mean	SD	Min	Max	Obs
485.64	2,755.29	0.00	120,000.00	8,106

ca018_3_2_: Total In-kind Payment Provided to XChildPanAliveName[2]

Mean	SD	Min	Max	Obs
241.09	1,510.65	0.00	60,000.00	7,585

ca018_3_3_: Total In-kind Payment Provided to XChildPanAliveName[3]

Mean	SD	Min	Max	Obs
114.77	677.74	0.00	20,000.00	4,399

ca018_3_4_: Total In-kind Payment Provided to XChildPanAliveName[4]

Mean	SD	Min	Max	Obs
67.70	448.78	0.00	15,000.00	2,315

ca018_3_5_: Total In-kind Payment Provided to XChildPanAliveName[5]

Mean	SD	Min	Max	Obs
36.93	194.16	0.00	3,650.00	1,105

ca018_3_6_: Total In-kind Payment Provided to XChildPanAliveName[6]

Mean	SD	Min	Max	Obs
36.04	220.77	0.00	3,000.00	469

ca018_3_7_: Total In-kind Payment Provided to XChildPanAliveName[7]

Mean	SD	Min	Max	Obs
20.17	105.85	0.00	1,200.00	211

ca018_3_8_: Total In-kind Payment Provided to XChildPanAliveName[8]

Mean	SD	Min	Max	Obs
11.26	44.72	0.00	260.00	87

ca018_3_9_: Total In-kind Payment Provided to XChildPanAliveName[9]

Mean	SD	Min	Max	Obs
6.06	34.82	0.00	200.00	33

ca018_3_10_: Total In-kind Payment Provided to XChildPanAliveName[10]

Mean	SD	Min	Max	Obs
41.67	143.74	0.00	600.00	18

ca018_3_11_: Total In-kind Payment Provided to XChildPanAliveName[11]

Mean	SD	Min	Max	Obs
0.00	0.00	0.00	0.00	8

ca018_3_12_: Total In-kind Payment Provided to XChildPanAliveName[12]

Mean	SD	Min	Max	Obs
0.00	0.00	0.00	0.00	3

ca018_3_13_: Total In-kind Payment Provided to XChildPanAliveName[13]

No Observations

ca018_3_14_: Total In-kind Payment Provided to XChildPanAliveName[14]

No Observations

ca018_3_15_: Total In-kind Payment Provided to XChildPanAliveName[15]

No Observations

ca018_3_16_: Total In-kind Payment Provided to XChildPanAliveName[16]

No Observations

ca018_3_17_: Total In-kind Payment Provided to XChildPanAliveName[17]

Mean	SD	Min	Max	Obs
0.00	.	0.00	0.00	1

ca018_3_min_1_: Min Bracket of ca018_3_1_

Mean	SD	Min	Max	Obs
1,029.76	2,383.98	-1.00	20,000.00	196

ca018_3_min_2_: Min Bracket of ca018_3_2_

Mean	SD	Min	Max	Obs
862.70	2,616.06	-1.00	20,000.00	141

ca018_3_min_3_: Min Bracket of ca018_3_3_

Mean	SD	Min	Max	Obs
415.45	852.30	-1.00	5,000.00	44

ca018_3_min_4_: Min Bracket of ca018_3_4_

Mean	SD	Min	Max	Obs
832.70	3,834.70	-1.00	20,000.00	27

ca018_3_min_5_: Min Bracket of ca018_3_5_

Mean	SD	Min	Max	Obs
926.64	2,014.32	-1.00	5,000.00	11

ca018_3_min_6_: Min Bracket of ca018_3_6_

Mean	SD	Min	Max	Obs
19.20	45.17	-1.00	100.00	5

ca018_3_min_7_: Min Bracket of ca018_3_7_

Mean	SD	Min	Max	Obs
1,666.00	2,887.33	-1.00	5,000.00	3

ca018_3_min_8_: Min Bracket of ca018_3_8_

No Observations

ca018_3_min_9_: Min Bracket of ca018_3_9_

Mean	SD	Min	Max	Obs

-1.00	.	-1.00	-1.00	1

ca018_3_max_1_: Max Bracket of ca018_3_1_

Mean	SD	Min	Max	Obs
1,910.40	4,033.13	-1.00	20,000.00	205

ca018_3_max_2_: Max Bracket of ca018_3_2_

Mean	SD	Min	Max	Obs
1,648.96	4,318.36	-1.00	20,000.00	148

ca018_3_max_3_: Max Bracket of ca018_3_3_

Mean	SD	Min	Max	Obs
801.72	1,524.16	-1.00	5,000.00	47

ca018_3_max_4_: Max Bracket of ca018_3_4_

Mean	SD	Min	Max	Obs
229.00	420.81	-1.00	1,500.00	27

ca018_3_max_5_: Max Bracket of ca018_3_5_

Mean	SD	Min	Max	Obs
1,008.55	1,982.96	-1.00	5,000.00	11

ca018_3_max_6_: Max Bracket of ca018_3_6_

Mean	SD	Min	Max	Obs
15.83	41.23	-1.00	100.00	6

ca018_3_max_7_: Max Bracket of ca018_3_7_

Mean	SD	Min	Max	Obs
1,666.00	2,887.33	-1.00	5,000.00	3

ca018_3_max_8_: Max Bracket of ca018_3_8_

No Observations

ca018_3_max_9_: Max Bracket of ca018_3_9_

Mean	SD	Min	Max	Obs
-1.00	.	-1.00	-1.00	1

ca018_4_1_: Regular In-kind Payment Provided to XChildPanAliveName[1]

Mean	SD	Min	Max	Obs
84.86	1,230.64	0.00	60,000.00	8,259

ca018_4_2_: Regular In-kind Payment Provided to XChildPanAliveName[2]

Mean	SD	Min	Max	Obs
31.32	477.96	0.00	24,000.00	7,691

ca018_4_3_: Regular In-kind Payment Provided to XChildPanAliveName[3]

Mean	SD	Min	Max	Obs
22.46	351.52	0.00	18,000.00	4,433

ca018_4_4_: Regular In-kind Payment Provided to XChildPanAliveName[4]

Mean	SD	Min	Max	Obs
12.47	160.80	0.00	5,000.00	2,328

ca018_4_5_: Regular In-kind Payment Provided to XChildPanAliveName[5]

Mean	SD	Min	Max	Obs
2.37	35.47	0.00	1,000.00	1,110

ca018_4_6_: Regular In-kind Payment Provided to XChildPanAliveName[6]

Mean	SD	Min	Max	Obs
3.68	33.99	0.00	600.00	472

ca018_4_7_: Regular In-kind Payment Provided to XChildPanAliveName[7]

Mean	SD	Min	Max	Obs
0.65	6.49	0.00	80.00	212

ca018_4_8_: Regular In-kind Payment Provided to XChildPanAliveName[8]

Mean	SD	Min	Max	Obs
2.30	21.44	0.00	200.00	87

ca018_4_9_: Regular In-kind Payment Provided to XChildPanAliveName[9]

Mean	SD	Min	Max	Obs
0.00	0.00	0.00	0.00	34

ca018_4_10_: Regular In-kind Payment Provided to XChildPanAliveName[10]

Mean	SD	Min	Max	Obs
0.00	0.00	0.00	0.00	18

ca018_4_11_: Regular In-kind Payment Provided to XChildPanAliveName[11]

Mean	SD	Min	Max	Obs
0.00	0.00	0.00	0.00	8

ca018_4_12_: Regular In-kind Payment Provided to XChildPanAliveName[12]

Mean	SD	Min	Max	Obs
0.00	0.00	0.00	0.00	3

ca018_4_13_: Regular In-kind Payment Provided to XChildPanAliveName[13]

No Observations

ca018_4_14_: Regular In-kind Payment Provided to XChildPanAliveName[14]

No Observations

ca018_4_15_: Regular In-kind Payment Provided to XChildPanAliveName[15]

No Observations

ca018_4_16_: Regular In-kind Payment Provided to XChildPanAliveName[16]

No Observations

ca018_4_17_: Regular In-kind Payment Provided to XChildPanAliveName[17]

Mean	SD	Min	Max	Obs
0.00	.	0.00	0.00	1

ca018_4_min_1_: Min Bracket of ca018_4_1_

Mean	SD	Min	Max	Obs
137.22	477.85	-1.00	3,000.00	50

ca018_4_min_2_: Min Bracket of ca018_4_2_

Mean	SD	Min	Max	Obs
224.13	697.81	-1.00	3,000.00	40

ca018_4_min_3_: Min Bracket of ca018_4_3_

Mean	SD	Min	Max	Obs
245.31	829.54	-1.00	3,000.00	13

ca018_4_min_4_: Min Bracket of ca018_4_4_

Mean	SD	Min	Max	Obs
12.40	51.90	-1.00	200.00	15

ca018_4_min_5_: Min Bracket of ca018_4_5_

Mean	SD	Min	Max	Obs
-1.00	0.00	-1.00	-1.00	6

ca018_4_min_6_: Min Bracket of ca018_4_6_

Mean	SD	Min	Max	Obs
-1.00	0.00	-1.00	-1.00	3

ca018_4_min_7_: Min Bracket of ca018_4_7_

Mean	SD	Min	Max	Obs
-1.00	0.00	-1.00	-1.00	2

ca018_4_max_1_: Max Bracket of ca018_4_1_

Mean	SD	Min	Max	Obs
549.23	2,001.83	-1.00	10,000.00	52

ca018_4_max_2_: Max Bracket of ca018_4_2_

Mean	SD	Min	Max	Obs
378.21	1,632.11	-1.00	10,000.00	43

ca018_4_max_3_: Max Bracket of ca018_4_3_

Mean	SD	Min	Max	Obs
306.85	855.18	-1.00	3,000.00	13

ca018_4_max_4_: Max Bracket of ca018_4_4_

Mean	SD	Min	Max	Obs
65.73	258.46	-1.00	1,000.00	15

ca018_4_max_5_: Max Bracket of ca018_4_5_

Mean	SD	Min	Max	Obs
-1.00	0.00	-1.00	-1.00	6

ca018_4_max_6_: Max Bracket of ca018_4_6_

Mean	SD	Min	Max	Obs
-1.00	0.00	-1.00	-1.00	3

ca018_4_max_7_: Max Bracket of ca018_4_7_

Mean	SD	Min	Max	Obs
-1.00	0.00	-1.00	-1.00	2

ca019_1_: Relationship With XChildPanAliveName[1] Affected By the COVID-19 Pandemic?

	Freq.	%
1 Got Better	497	4.63
2 Got Worse	365	3.40
3 No Influence	9,870	91.97
Total	10,732	100.00

ca019_2_: Relationship With XChildPanAliveName[2] Affected By the COVID-19 Pandemic?

	Freq.	%
1 Got Better	383	4.34
2 Got Worse	364	4.12
3 No Influence	8,088	91.54
Total	8,835	100.00

ca019_3_: Relationship With XChildPanAliveName[3] Affected By the COVID-19 Pandemic?

	Freq.	%
1 Got Better	180	3.72
2 Got Worse	211	4.36
3 No Influence	4,451	91.92
Total	4,842	100.00

ca019_4_: Relationship With XChildPanAliveName[4] Affected By the COVID-19 Pandemic?

	Freq.	%
1 Got Better	95	3.79
2 Got Worse	91	3.63
3 No Influence	2,321	92.58
Total	2,507	100.00

ca019_5_: Relationship With XChildPanAliveName[5] Affected By the COVID-19 Pandemic?

	Freq.	%
1 Got Better	46	3.96
2 Got Worse	44	3.78
3 No Influence	1,073	92.26
Total	1,163	100.00

ca019_6_: Relationship With XChildPanAliveName[6] Affected By the COVID-19 Pandemic?

	Freq.	%
1 Got Better	19	3.85
2 Got Worse	18	3.65
3 No Influence	456	92.49
Total	493	100.00

ca019_7_: Relationship With XChildPanAliveName[7] Affected By the COVID-19 Pandemic?

	Freq.	%
1 Got Better	9	3.98
2 Got Worse	10	4.42
3 No Influence	207	91.59
Total	226	100.00

ca019_8_: Relationship With XChildPanAliveName[8] Affected By the COVID-19 Pandemic?

	Freq.	%
1 Got Better	3	3.30
2 Got Worse	3	3.30
3 No Influence	85	93.41
Total	91	100.00

ca019_9_: Relationship With XChildPanAliveName[9] Affected By the COVID-19 Pandemic?

	Freq.	%
1 Got Better	1	2.78
2 Got Worse	5	13.89
3 No Influence	30	83.33
Total	36	100.00

ca019_10_: Relationship With XChildPanAliveName[10] Affected By the COVID-19 Pandemic?

	Freq.	%
1 Got Better	1	5.26
3 No Influence	18	94.74

Total	19	100.00

ca019_11_: Relationship With XChildPanAliveName[11] Affected By the COVID-19 Pandemic?

	Freq.	%
3 No Influence	8	100.00
Total	8	100.00

ca019_12_: Relationship With XChildPanAliveName[12] Affected By the COVID-19 Pandemic?

	Freq.	%
3 No Influence	3	100.00
Total	3	100.00

ca019_13_: Relationship With XChildPanAliveName[13] Affected By the COVID-19 Pandemic?

	Freq.	%
3 No Influence	1	100.00
Total	1	100.00

ca019_14_: Relationship With XChildPanAliveName[14] Affected By the COVID-19 Pandemic?

No Observations

ca019_15_: Relationship With XChildPanAliveName[15] Affected By the COVID-19 Pandemic?

No Observations

ca019_16_: Relationship With XChildPanAliveName[16] Affected By the COVID-19 Pandemic?

No Observations

ca019_17_: Relationship With XChildPanAliveName[17] Affected By the COVID-19 Pandemic?

	Freq.	%
3 No Influence	1	100.00
Total	1	100.00

ca020_1_: Was XChildCoupleDis[1]'s Income Affected By the COVID-19 Pandemic?

	Freq.	%

	Freq.	%
1 Income Increased	61	0.57
2 Income Decreased	6,097	57.16
3 No Influence	3,768	35.33
997 Don't Know	738	6.92
999 Refuse	2	0.02
Total	10,666	100.00

ca020_2_: Was XChildCoupleDis[2]'s Income Affected By the COVID-19 Pandemic?

	Freq.	%
1 Income Increased	42	0.48
2 Income Decreased	4,637	53.55
3 No Influence	3,220	37.18
997 Don't Know	757	8.74
999 Refuse	4	0.05
Total	8,660	100.00

ca020_3_: Was XChildCoupleDis[3]'s Income Affected By the COVID-19 Pandemic?

	Freq.	%
1 Income Increased	24	0.50
2 Income Decreased	2,399	50.35
3 No Influence	1,874	39.33
997 Don't Know	466	9.78
999 Refuse	2	0.04
Total	4,765	100.00

ca020_4_: Was XChildCoupleDis[4]'s Income Affected By the COVID-19 Pandemic?

	Freq.	%
1 Income Increased	8	0.32
2 Income Decreased	1,147	46.34
3 No Influence	1,036	41.86
997 Don't Know	283	11.43
999 Refuse	1	0.04
Total	2,475	100.00

ca020_5_: Was XChildCoupleDis[5]'s Income Affected By the COVID-19 Pandemic?

	Freq.	%
1 Income Increased	2	0.17
2 Income Decreased	476	41.11
3 No Influence	527	45.51
997 Don't Know	153	13.21
Total	1,158	100.00

ca020_6_: Was XChildCoupleDis[6]'s Income Affected By the COVID-19 Pandemic?

	Freq.	%
1 Income Increased	3	0.61
2 Income Decreased	185	37.91
3 No Influence	214	43.85
997 Don't Know	86	17.62
Total	488	100.00

ca020_7_: Was XChildCoupleDis[7]'s Income Affected By the COVID-19 Pandemic?

	Freq.	%
2 Income Decreased	74	33.64
3 No Influence	107	48.64
997 Don't Know	39	17.73
Total	220	100.00

ca020_8_: Was XChildCoupleDis[8]'s Income Affected By the COVID-19 Pandemic?

	Freq.	%
1 Income Increased	1	1.14
2 Income Decreased	29	32.95
3 No Influence	44	50.00
997 Don't Know	14	15.91
Total	88	100.00

ca020_9_: Was XChildCoupleDis[9]'s Income Affected By the COVID-19 Pandemic?

	Freq.	%
1 Income Increased	1	2.78
2 Income Decreased	11	30.56
3 No Influence	17	47.22
997 Don't Know	7	19.44
Total	36	100.00

ca020_10_: Was XChildCoupleDis[10]'s Income Affected By the COVID-19 Pandemic?

	Freq.	%
2 Income Decreased	6	31.58
3 No Influence	10	52.63
997 Don't Know	3	15.79
Total	19	100.00

ca020_11_: Was XChildCoupleDis[11]'s Income Affected By the COVID-19 Pandemic?

	Freq.	%
2 Income Decreased	2	25.00

	Freq.	%
3 No Influence	3	37.50
997 Don't Know	3	37.50
Total	8	100.00

ca020_12_: Was XChildCoupleDis[12]'s Income Affected By the COVID-19 Pandemic?

	Freq.	%
2 Income Decreased	1	33.33
3 No Influence	2	66.67
Total	3	100.00

ca020_13_: Was XChildCoupleDis[13]'s Income Affected By the COVID-19 Pandemic?

	Freq.	%
997 Don't Know	1	100.00
Total	1	100.00

ca020_14_: Was XChildCoupleDis[14]'s Income Affected By the COVID-19 Pandemic?

No Observations

ca020_15_: Was XChildCoupleDis[15]'s Income Affected By the COVID-19 Pandemic?

No Observations

ca020_16_: Was XChildCoupleDis[16]'s Income Affected By the COVID-19 Pandemic?

No Observations

ca020_17_: Was XChildCoupleDis[17]'s Income Affected By the COVID-19 Pandemic?

	Freq.	%
997 Don't Know	1	100.00
Total	1	100.00

ca020_1_1_: Percent of Increase in Income

Mean	SD	Min	Max	Obs
26.72	28.82	-1.00	100.00	61

ca020_1_2_: Percent of Increase in Income

Mean	SD	Min	Max	Obs

741.81	4,625.07	-1.00	30,000.00	42

ca020_1_3_: Percent of Increase in Income

Mean	SD	Min	Max	Obs
23.88	26.40	-1.00	100.00	24

ca020_1_4_: Percent of Increase in Income

Mean	SD	Min	Max	Obs
13.38	15.19	-1.00	40.00	8

ca020_1_5_: Percent of Increase in Income

Mean	SD	Min	Max	Obs
6.50	4.95	3.00	10.00	2

ca020_1_6_: Percent of Increase in Income

Mean	SD	Min	Max	Obs
26.67	20.82	10.00	50.00	3

ca020_1_7_: Percent of Increase in Income

No Observations

ca020_1_8_: Percent of Increase in Income

Mean	SD	Min	Max	Obs
-1.00	.	-1.00	-1.00	1

ca020_1_9_: Percent of Increase in Income

Mean	SD	Min	Max	Obs
10.00	.	10.00	10.00	1

ca020_2_1_: Percent of Decrease in Income

Mean	SD	Min	Max	Obs
38.42	29.67	-1.00	100.00	6,097

ca020_2_2_: Percent of Decrease in Income

Mean	SD	Min	Max	Obs
37.96	29.71	-1.00	100.00	4,637

ca020_2_3_: Percent of Decrease in Income

Mean	SD	Min	Max	Obs
37.51	29.71	-1.00	100.00	2,399

ca020_2_4_: Percent of Decrease in Income

Mean	SD	Min	Max	Obs
37.87	30.16	-1.00	100.00	1,147

ca020_2_5_: Percent of Decrease in Income

Mean	SD	Min	Max	Obs
37.45	30.10	-1.00	100.00	476

ca020_2_6_: Percent of Decrease in Income

Mean	SD	Min	Max	Obs
36.99	28.51	-1.00	100.00	185

ca020_2_7_: Percent of Decrease in Income

Mean	SD	Min	Max	Obs
35.84	30.71	-1.00	100.00	74

ca020_2_8_: Percent of Decrease in Income

Mean	SD	Min	Max	Obs
32.97	28.35	-1.00	100.00	29

ca020_2_9_: Percent of Decrease in Income

Mean	SD	Min	Max	Obs
34.73	31.56	-1.00	100.00	11

ca020_2_10_: Percent of Decrease in Income

Mean	SD	Min	Max	Obs
37.33	28.51	-1.00	70.00	6

ca020_2_11_: Percent of Decrease in Income

Mean	SD	Min	Max	Obs
14.50	21.92	-1.00	30.00	2

ca020_2_12_: Percent of Decrease in Income

Mean	SD	Min	Max	Obs
50.00	.	50.00	50.00	1

cb001: Select Household Member from List

A String Variable	
Obs:	11,157

cb002: Household Other Members

A String Variable	
Obs:	11,395

cb003_1_: XHHOtherMemberName[1]'s Gender

	Freq.	%
1 Male	1,184	32.85
2 Female	2,420	67.15
Total	3,604	100.00

cb003_2_: XHHOtherMemberName[2]'s Gender

	Freq.	%
1 Male	1,104	48.61
2 Female	1,167	51.39
Total	2,271	100.00

cb003_3_: XHHOtherMemberName[3]'s Gender

	Freq.	%
1 Male	552	46.86
2 Female	626	53.14
Total	1,178	100.00

cb003_4_: XHHOtherMemberName[4]'s Gender

	Freq.	%
1 Male	171	50.00
2 Female	171	50.00
Total	342	100.00

cb003_5_: XHHOtherMemberName[5]'s Gender

	Freq.	%
1 Male	66	48.89
2 Female	69	51.11
Total	135	100.00

cb003_6_: XHHOtherMemberName[6]'s Gender

	Freq.	%
1 Male	25	43.10
2 Female	33	56.90
Total	58	100.00

cb003_7_: XHHOtherMemberName[7]'s Gender

	Freq.	%
1 Male	12	42.86
2 Female	16	57.14
Total	28	100.00

cb003_8_: XHHOtherMemberName[8]'s Gender

	Freq.	%
1 Male	5	29.41
2 Female	12	70.59
Total	17	100.00

cb003_9_: XHHOtherMemberName[9]'s Gender

	Freq.	%
1 Male	5	83.33
2 Female	1	16.67
Total	6	100.00

cb003_10_: XHHOtherMemberName[10]'s Gender

	Freq.	%
2 Female	1	100.00
Total	1	100.00

cb004_1_: XHHOtherMemberName[1]'s Age

Mean	SD	Min	Max	Obs
33.04	23.29	0.00	106.00	3,520

cb004_2_: XHHOtherMemberName[2]'s Age

Mean	SD	Min	Max	Obs
14.76	15.11	0.00	90.00	2,247

cb004_3_: XHHOtherMemberName[3]'s Age

Mean	SD	Min	Max	Obs
12.30	13.25	0.00	98.00	1,163

cb004_4_: XHHOtherMemberName[4]'s Age

Mean	SD	Min	Max	Obs
12.50	14.00	0.00	90.00	335

cb004_5_: XHHOtherMemberName[5]'s Age

Mean	SD	Min	Max	Obs
11.32	13.36	0.00	94.00	131

cb004_6_: XHHOtherMemberName[6]'s Age

Mean	SD	Min	Max	Obs
11.31	15.63	0.00	91.00	58

cb004_7_: XHHOtherMemberName[7]'s Age

Mean	SD	Min	Max	Obs
10.43	9.65	0.00	35.00	28

cb004_8_: XHHOtherMemberName[8]'s Age

Mean	SD	Min	Max	Obs
8.76	9.61	1.00	31.00	17

cb004_9_: XHHOtherMemberName[9]'s Age

Mean	SD	Min	Max	Obs
6.33	7.50	1.00	20.00	6

cb004_10_: XHHOtherMemberName[10]'s Age

Mean	SD	Min	Max	Obs
3.00	.	3.00	3.00	1

cb005_1_: XHHOtherMemberName[1] is XFamilyR's?

	Freq.	%
1 Daughter-in-law/Son-in-law	1,775	49.25
2 Grandchild	1,289	35.77
3 Brother-in-law/Sister-in-law	1	0.03
4 Father	72	2.00
5 Mother	160	4.44
6 Mother-in-law	101	2.80
7 Father-in-law	56	1.55
8 Child	58	1.61
9 Sibling	30	0.83
10 Other Kind of Relative	62	1.72

Total	3,604	100.00

cb005_2_: XHHOtherMemberName[2] is XFamilyR's?

	Freq.	%
1 Daughter-in-law/Son-in-law	171	7.53
2 Grandchild	1,931	85.03
3 Brother-in-law/Sister-in-law	1	0.04
4 Father	5	0.22
5 Mother	35	1.54
6 Mother-in-law	18	0.79
7 Father-in-law	10	0.44
8 Child	10	0.44
9 Sibling	6	0.26
10 Other Kind of Relative	84	3.70
Total	2,271	100.00

cb005_3_: XHHOtherMemberName[3] is XFamilyR's?

	Freq.	%
1 Daughter-in-law/Son-in-law	116	9.85
2 Grandchild	971	82.43
3 Brother-in-law/Sister-in-law	3	0.25
4 Father	1	0.08
5 Mother	1	0.08
6 Mother-in-law	7	0.59
7 Father-in-law	1	0.08
8 Child	4	0.34
9 Sibling	2	0.17
10 Other Kind of Relative	72	6.11
Total	1,178	100.00

cb005_4_: XHHOtherMemberName[4] is XFamilyR's?

	Freq.	%
1 Daughter-in-law/Son-in-law	40	11.70
2 Grandchild	241	70.47
3 Brother-in-law/Sister-in-law	3	0.88
5 Mother	1	0.29
6 Mother-in-law	1	0.29
7 Father-in-law	1	0.29
9 Sibling	3	0.88
10 Other Kind of Relative	52	15.20
Total	342	100.00

cb005_5_: XHHOtherMemberName[5] is XFamilyR's?

	Freq.	%
1 Daughter-in-law/Son-in-law	14	10.37
2 Grandchild	91	67.41
6 Mother-in-law	1	0.74
7 Father-in-law	1	0.74
10 Other Kind of Relative	28	20.74

	Freq.	%
Total	135	100.00

cb005_6_: XHHOtherMemberName[6] is XFamilyR's?

	Freq.	%
1 Daughter-in-law/Son-in-law	5	8.62
2 Grandchild	39	67.24
6 Mother-in-law	1	1.72
8 Child	1	1.72
10 Other Kind of Relative	12	20.69
Total	58	100.00

cb005_7_: XHHOtherMemberName[7] is XFamilyR's?

	Freq.	%
1 Daughter-in-law/Son-in-law	3	10.71
2 Grandchild	18	64.29
10 Other Kind of Relative	7	25.00
Total	28	100.00

cb005_8_: XHHOtherMemberName[8] is XFamilyR's?

	Freq.	%
1 Daughter-in-law/Son-in-law	2	11.76
2 Grandchild	10	58.82
10 Other Kind of Relative	5	29.41
Total	17	100.00

cb005_9_: XHHOtherMemberName[9] is XFamilyR's?

	Freq.	%
2 Grandchild	3	50.00
10 Other Kind of Relative	3	50.00
Total	6	100.00

cb005_10_: XHHOtherMemberName[10] is XFamilyR's?

	Freq.	%
2 Grandchild	1	100.00
Total	1	100.00

cb006_1_: Which Child of XFamilyR has XHHOtherMemberName[1] Been Married With?

Mean	SD	Min	Max	Obs
2.35	8.98	1.00	99.00	1,775

cb006_2_: Which Child of XFamilyR has XHHOtherMemberName[2] Been Married With?

Mean	SD	Min	Max	Obs
11.94	29.96	1.00	99.00	171

cb006_3_: Which Child of XFamilyR has XHHOtherMemberName[3] Been Married With?

Mean	SD	Min	Max	Obs
4.09	15.56	1.00	99.00	116

cb006_4_: Which Child of XFamilyR has XHHOtherMemberName[4] Been Married With?

Mean	SD	Min	Max	Obs
4.33	15.40	1.00	99.00	40

cb006_5_: Which Child of XFamilyR has XHHOtherMemberName[5] Been Married With?

Mean	SD	Min	Max	Obs
9.00	25.93	1.00	99.00	14

cb006_6_: Which Child of XFamilyR has XHHOtherMemberName[6] Been Married With?

Mean	SD	Min	Max	Obs
2.00	0.71	1.00	3.00	5

cb006_7_: Which Child of XFamilyR has XHHOtherMemberName[7] Been Married With?

Mean	SD	Min	Max	Obs
2.67	1.53	1.00	4.00	3

cb006_8_: Which Child of XFamilyR has XHHOtherMemberName[8] Been Married With?

Mean	SD	Min	Max	Obs
1.50	0.71	1.00	2.00	2

cb007_1_: Who is XHHOtherMemberName[1]'s Parent?

Mean	SD	Min	Max	Obs
4.39	16.30	1.00	99.00	1,289

cb007_2_: Who is XHHOtherMemberName[1]'s Parent?

Mean	SD	Min	Max	Obs
2.91	11.69	1.00	99.00	1,931

cb007_3_: Who is XHHOtherMemberName[1]'s Parent?

Mean	SD	Min	Max	Obs
4.31	16.35	1.00	99.00	971

cb007_4_: Who is XHHOtherMemberName[1]'s Parent?

Mean	SD	Min	Max	Obs
6.95	22.05	1.00	99.00	241

cb007_5_: Who is XHHOtherMemberName[1]'s Parent?

Mean	SD	Min	Max	Obs
7.24	22.26	1.00	99.00	91

cb007_6_: Who is XHHOtherMemberName[1]'s Parent?

Mean	SD	Min	Max	Obs
4.54	15.55	1.00	99.00	39

cb007_7_: Who is XHHOtherMemberName[1]'s Parent?

Mean	SD	Min	Max	Obs
7.17	22.93	1.00	99.00	18

cb007_8_: Who is XHHOtherMemberName[1]'s Parent?

Mean	SD	Min	Max	Obs
2.10	0.88	1.00	4.00	10

cb007_9_: Who is XHHOtherMemberName[1]'s Parent?

Mean	SD	Min	Max	Obs
1.33	0.58	1.00	2.00	3

cb007_10_: Who is XHHOtherMemberName[1]'s Parent?

Mean	SD	Min	Max	Obs
1.00	.	1.00	1.00	1

cc001: Children/Grandchildren Were Unable to Return Home Due To the COVID-19 Pandemic

	Freq.	%
1 Yes	2,349	20.61
2 No	9,046	79.39
Total	11,395	100.00

cc001_1: Number of Children/Grandchildren Who Were Unbale to Return Home

Mean	SD	Min	Max	Obs
3.33	2.60	1.00	15.00	2,349

cc002: Relatives/Friends Were Unable to Visit XFamilyR

	Freq.	%
1 Yes	7,282	63.91
2 No	4,113	36.09
Total	11,395	100.00

cc003: XFamilyRAndS Received Less Money in Lunar January(Zhengyue)

	Freq.	%
1 Yes	2,427	31.22
2 No	5,347	68.78
Total	7,774	100.00

cc003_1: Amount of Yuan Less Than Previous Years

Mean	SD	Min	Max	Obs
1,277.87	2,603.46	0.00	100,000.00	2,427

cc004: Spent Less Money Giving Hongbao(Red Envelop)

	Freq.	%
1 Yes	3,193	41.07
2 No	4,581	58.93
Total	7,774	100.00

cc004_1: Amount of Yuan Less Than Previous Years

Mean	SD	Min	Max	Obs
1,053.21	1,206.11	1.00	20,000.00	3,193

cc005: Did XFamilyRAndS Mostly Live Together in Lunar January(Zhengyue)?

	Freq.	%
1 Yes	7,961	96.08
2 No	325	3.92
Total	8,286	100.00

cc006_1_: Who Did XCoupleList[1] Live With in Lunar January(Zhengyue)?

A String Variable	
Obs:	11,237

cc006_2_: Who Did XCoupleList[2] Live With in Lunar January(Zhengyue)?

A String Variable	
Obs:	323

cc007_1_: Who Else Lived With CouLiveList[1] in Lunar January(Zhengyue)?

A String Variable	
Obs:	11,394

cc007_2_: Who Else Lived With CouLiveList[2] in Lunar January(Zhengyue)?

A String Variable	
Obs:	325

cc008_1_: More or Fewer People Lived With XCouLiveList[1] in Lunar January(Zhengyue)?

	Freq.	%
1 More People	842	7.39
2 Fewer People	2,506	22.00
3 No Difference	8,045	70.61
Total	11,393	100.00

cc008_2_: More or Fewer People Lived With XCouLiveList[2] in Lunar January(Zhengyue)?

	Freq.	%
1 More People	19	5.85
2 Fewer People	118	36.31
3 No Difference	188	57.85
Total	325	100.00

xhhmembernum: Number of Household Members

Mean	SD	Min	Max	Obs
1.14	1.55	0.00	14.00	11,395

xhhmemberage_1_: XHHMemberName[1]'s Age

Mean	SD	Min	Max	Obs
34.07	16.99	1.00	117.00	5,586

xhhmemberage_2_: XHHMemberName[2]'s Age

Mean	SD	Min	Max	Obs
29.99	18.62	0.00	99.00	3,319

xhhmemberage_3_: XHHMemberName[3]'s Age

Mean	SD	Min	Max	Obs
16.98	16.14	0.00	95.00	2,058

xhhmemberage_4_: XHHMemberName[4]'s Age

Mean	SD	Min	Max	Obs
14.19	14.93	0.00	98.00	1,148

xhhmemberage_5_: XHHMemberName[5]'s Age

Mean	SD	Min	Max	Obs
13.00	14.76	0.00	95.00	365

xhhmemberage_6_: XHHMemberName[6]'s Age

Mean	SD	Min	Max	Obs
12.82	14.85	0.00	94.00	158

xhhmemberage_7_: XHHMemberName[7]'s Age

Mean	SD	Min	Max	Obs
12.76	15.55	0.00	91.00	85

xhhmemberage_8_: XHHMemberName[8]'s Age

Mean	SD	Min	Max	Obs
12.19	14.68	0.00	59.00	43

xhhmemberage_9_: XHHMemberName[9]'s Age

Mean	SD	Min	Max	Obs
8.14	8.06	0.00	35.00	22

xhhmemberage_10_: XHHMemberName[10]'s Age

Mean	SD	Min	Max	Obs
10.29	10.37	1.00	31.00	14

xhhmemberage_11_: XHHMemberName[11]'s Age

Mean	SD	Min	Max	Obs
11.43	9.93	1.00	28.00	7

xhhmemberage_12_: XHHMemberName[12]'s Age

Mean	SD	Min	Max	Obs
9.67	13.32	1.00	25.00	3

xhhmemberage_13_: XHHMemberName[13]'s Age

Mean	SD	Min	Max	Obs
23.00	.	23.00	23.00	1

xhhmemberage_14_: XHHMemberName[14]'s Age

Mean	SD	Min	Max	Obs
20.00	.	20.00	20.00	1

xhhothermembernum: Number of Household Other Members

Mean	SD	Min	Max	Obs
0.67	1.19	0.00	10.00	11,395

xaffectreunion: Family Reunion Was Affected By the COVID-19 Pandemic

	Freq.	%
0 No	3,621	31.78
1 Yes	7,774	68.22
Total	11,395	100.00

xchildalivenum: Number of Children Alive

Mean	SD	Min	Max	Obs
2.54	1.36	0.00	10.00	11,405

xchildpanalivenum: Number of Children Alive When the COVID Pandemic Started

Mean	SD	Min	Max	Obs
2.54	1.37	0.00	10.00	11,405

xchildnum: Number of Children

Mean	SD	Min	Max	Obs

2.56	1.38	0.00	10.00	11,405

xchildalive_1_: Is ZChildName[1] Still Alive When R is Interviewed

	Freq.	%
0 No	59	0.55
1 Yes	10,725	99.45
Total	10,784	100.00

xchildalive_2_: Is ZChildName[2] Still Alive When R is Interviewed

	Freq.	%
0 No	46	0.52
1 Yes	8,833	99.48
Total	8,879	100.00

xchildalive_3_: Is ZChildName[3] Still Alive When R is Interviewed

	Freq.	%
0 No	44	0.90
1 Yes	4,836	99.10
Total	4,880	100.00

xchildalive_4_: Is ZChildName[4] Still Alive When R is Interviewed

	Freq.	%
0 No	36	1.42
1 Yes	2,500	98.58
Total	2,536	100.00

xchildalive_5_: Is ZChildName[5] Still Alive When R is Interviewed

	Freq.	%
0 No	16	1.36
1 Yes	1,161	98.64
Total	1,177	100.00

xchildalive_6_: Is ZChildName[6] Still Alive When R is Interviewed

	Freq.	%
0 No	14	2.77
1 Yes	491	97.23
Total	505	100.00

xchildalive_7_: Is ZChildName[7] Still Alive When R is Interviewed

	Freq.	%
0 No	5	2.18
1 Yes	224	97.82

Total	229	100.00

xchildalive_8_: Is ZChildName[8] Still Alive When R is Interviewed

	Freq.	%
0 No	6	6.19
1 Yes	91	93.81
Total	97	100.00

xchildalive_9_: Is ZChildName[9] Still Alive When R is Interviewed

	Freq.	%
0 No	2	5.26
1 Yes	36	94.74
Total	38	100.00

xchildalive_10_: Is ZChildName[10] Still Alive When R is Interviewed

	Freq.	%
0 No	2	9.52
1 Yes	19	90.48
Total	21	100.00

xchildalive_11_: Is ZChildName[11] Still Alive When R is Interviewed

	Freq.	%
0 No	1	11.11
1 Yes	8	88.89
Total	9	100.00

xchildalive_12_: Is ZChildName[12] Still Alive When R is Interviewed

	Freq.	%
1 Yes	3	100.00
Total	3	100.00

xchildalive_13_: Is ZChildName[13] Still Alive When R is Interviewed

	Freq.	%
1 Yes	1	100.00
Total	1	100.00

xchildalive_14_: Is ZChildName[14] Still Alive When R is Interviewed

No Observations

xchildalive_15_: Is ZChildName[15] Still Alive When R is Interviewed

No Observations

xchildalive_16_: Is ZChildName[16] Still Alive When R is Interviewed

No Observations

xchildalive_17_: Is ZChildName[17] Still Alive When R is Interviewed

	Freq.	%
1 Yes	1	100.00
Total	1	100.00

xchildpanalive_1_: ZChildName[1] Was Still Alive When the COVID-19 Pandemic Started

	Freq.	%
0 No	50	0.46
1 Yes	10,734	99.54
Total	10,784	100.00

xchildpanalive_2_: ZChildName[2] Was Still Alive When the COVID-19 Pandemic Started

	Freq.	%
0 No	39	0.44
1 Yes	8,840	99.56
Total	8,879	100.00

xchildpanalive_3_: ZChildName[3] Was Still Alive When the COVID-19 Pandemic Started

	Freq.	%
0 No	36	0.74
1 Yes	4,844	99.26
Total	4,880	100.00

xchildpanalive_4_: ZChildName[4] Was Still Alive When the COVID-19 Pandemic Started

	Freq.	%
0 No	29	1.14
1 Yes	2,507	98.86
Total	2,536	100.00

xchildpanalive_5_: ZChildName[5] Was Still Alive When the COVID-19 Pandemic Started

	Freq.	%
0 No	14	1.19

1 Yes	1,163	98.81
Total	1,177	100.00

xchildpanalive_6_: ZChildName[6] Was Still Alive When the COVID-19 Pandemic Started

	Freq.	%
0 No	12	2.38
1 Yes	493	97.62
Total	505	100.00

xchildpanalive_7_: ZChildName[7] Was Still Alive When the COVID-19 Pandemic Started

	Freq.	%
0 No	3	1.31
1 Yes	226	98.69
Total	229	100.00

xchildpanalive_8_: ZChildName[8] Was Still Alive When the COVID-19 Pandemic Started

	Freq.	%
0 No	6	6.19
1 Yes	91	93.81
Total	97	100.00

xchildpanalive_9_: ZChildName[9] Was Still Alive When the COVID-19 Pandemic Started

	Freq.	%
0 No	2	5.26
1 Yes	36	94.74
Total	38	100.00

xchildpanalive_10_: ZChildName[10] Was Still Alive When the COVID-19 Pandemic Started

	Freq.	%
0 No	2	9.52
1 Yes	19	90.48
Total	21	100.00

xchildpanalive_11_: ZChildName[11] Was Still Alive When the COVID-19 Pandemic Started

	Freq.	%
0 No	1	11.11
1 Yes	8	88.89
Total	9	100.00

xchildpanalive_12_: ZChildName[12] Was Still Alive When the COVID-19 Pandemic Started

	Freq.	%
1 Yes	3	100.00
Total	3	100.00

xchildpanalive_13_: ZChildName[13] Was Still Alive When the COVID-19 Pandemic Started

	Freq.	%
1 Yes	1	100.00
Total	1	100.00

xchildpanalive_14_: ZChildName[14] Was Still Alive When the COVID-19 Pandemic Started

No Observations

xchildpanalive_15_: ZChildName[15] Was Still Alive When the COVID-19 Pandemic Started

No Observations

xchildpanalive_16_: ZChildName[16] Was Still Alive When the COVID-19 Pandemic Started

No Observations

xchildpanalive_17_: ZChildName[17] Was Still Alive When the COVID-19 Pandemic Started

	Freq.	%
1 Yes	1	100.00
Total	1	100.00

xchildbirth_1_: ZChildName[1]'s Birth Year (updated after CA005)

Mean	SD	Min	Max	Obs
1,981.56	10.56	1,942.00	2,015.00	10,734

xchildbirth_2_: ZChildName[2]'s Birth Year (updated after CA005)

Mean	SD	Min	Max	Obs
1,981.22	11.32	1,903.00	2,018.00	8,840

xchildbirth_3_: ZChildName[3]'s Birth Year (updated after CA005)

Mean	SD	Min	Max	Obs
1,978.10	10.63	1,900.00	2,018.00	4,844

xchildbirth_4_: ZChildName[4]'s Birth Year (updated after CA005)

Mean	SD	Min	Max	Obs
1,975.81	10.00	1,922.00	2,015.00	2,507

xchildbirth_5_: ZChildName[5]'s Birth Year (updated after CA005)

Mean	SD	Min	Max	Obs
1,974.31	9.66	1,948.00	2,015.00	1,163

xchildbirth_6_: ZChildName[6]'s Birth Year (updated after CA005)

Mean	SD	Min	Max	Obs
1,973.82	9.34	1,947.00	2,015.00	493

xchildbirth_7_: ZChildName[7]'s Birth Year (updated after CA005)

Mean	SD	Min	Max	Obs
1,973.24	10.35	1,948.00	2,015.00	226

xchildbirth_8_: ZChildName[8]'s Birth Year (updated after CA005)

Mean	SD	Min	Max	Obs
1,974.14	11.54	1,950.00	2,015.00	91

xchildbirth_9_: ZChildName[9]'s Birth Year (updated after CA005)

Mean	SD	Min	Max	Obs
1,972.58	8.87	1,955.00	1,991.00	36

xchildbirth_10_: ZChildName[10]'s Birth Year (updated after CA005)

Mean	SD	Min	Max	Obs
1,970.89	4.19	1,961.00	1,977.00	19

xchildbirth_11_: ZChildName[11]'s Birth Year (updated after CA005)

Mean	SD	Min	Max	Obs
1,975.75	6.54	1,964.00	1,986.00	8

xchildbirth_12_: ZChildName[12]'s Birth Year (updated after CA005)

Mean	SD	Min	Max	Obs
1,981.33	5.86	1,977.00	1,988.00	3

xchildbirth_13_: ZChildName[13]'s Birth Year (updated after CA005)

Mean	SD	Min	Max	Obs
1,988.00	.	1,988.00	1,988.00	1

xchildbirth_14_: ZChildName[14]'s Birth Year (updated after CA005)

No Observations

xchildbirth_15_: ZChildName[15]'s Birth Year (updated after CA005)

No Observations

xchildbirth_16_: ZChildName[16]'s Birth Year (updated after CA005)

No Observations

xchildbirth_17_: ZChildName[17]'s Birth Year (updated after CA005)

Mean	SD	Min	Max	Obs
1,981.00	.	1,981.00	1,981.00	1

xchildgender_1_: ZChildName[1]'s Gender (updated after CA006)

	Freq.	%
1 Male	7,070	65.87
2 Female	3,664	34.13
Total	10,734	100.00

xchildgender_2_: ZChildName[2]'s Gender (updated after CA006)

	Freq.	%
1 Male	4,468	50.54
2 Female	4,372	49.46
Total	8,840	100.00

xchildgender_3_: ZChildName[3]'s Gender (updated after CA006)

	Freq.	%
1 Male	2,023	41.76
2 Female	2,821	58.24
Total	4,844	100.00

xchildgender_4_: ZChildName[4]'s Gender (updated after CA006)

	Freq.	%
1 Male	880	35.10
2 Female	1,627	64.90
Total	2,507	100.00

xchildgender_5_: ZChildName[5]'s Gender (updated after CA006)

	Freq.	%
1 Male	356	30.61
2 Female	807	69.39
Total	1,163	100.00

xchildgender_6_: ZChildName[6]'s Gender (updated after CA006)

	Freq.	%
1 Male	138	27.99
2 Female	355	72.01
Total	493	100.00

xchildgender_7_: ZChildName[7]'s Gender (updated after CA006)

	Freq.	%
1 Male	69	30.53
2 Female	157	69.47
Total	226	100.00

xchildgender_8_: ZChildName[8]'s Gender (updated after CA006)

	Freq.	%
1 Male	26	28.57
2 Female	65	71.43
Total	91	100.00

xchildgender_9_: ZChildName[9]'s Gender (updated after CA006)

	Freq.	%
1 Male	8	22.22
2 Female	28	77.78
Total	36	100.00

xchildgender_10_: ZChildName[10]'s Gender (updated after CA006)

	Freq.	%
1 Male	9	47.37
2 Female	10	52.63
Total	19	100.00

xchildgender_11_: ZChildName[11]'s Gender (updated after CA006)

	Freq.	%
1 Male	2	25.00
2 Female	6	75.00
Total	8	100.00

xchildgender_12_: ZChildName[12]'s Gender (updated after CA006)

	Freq.	%
1 Male	1	33.33
2 Female	2	66.67
Total	3	100.00

xchildgender_13_: ZChildName[13]'s Gender (updated after CA006)

	Freq.	%
1 Male	1	100.00
Total	1	100.00

xchildgender_14_: ZChildName[14]'s Gender (updated after CA006)

No Observations

xchildgender_15_: ZChildName[15]'s Gender (updated after CA006)

No Observations

xchildgender_16_: ZChildName[16]'s Gender (updated after CA006)

No Observations

xchildgender_17_: ZChildName[17]'s Gender (updated after CA006)

	Freq.	%
2 Female	1	100.00
Total	1	100.00

xchildedu_1_: ZChildName[1]'s Highest Degree (update after CA007)

	Freq.	%
1 No Formal Education(Illiterate)	272	2.53
2 Did not Finish Primary School	838	7.81
4 Elementary School	1,958	18.24
5 Middle School	3,500	32.61
6 High School	1,383	12.88
7 Vocational School	735	6.85
8 Two-/Three-Year College/Associate Degree	879	8.19
9 Four-Year College/Bachelor's Degree	1,013	9.44

10 Master's Degree	122	1.14
11 Doctoral Degree/Ph.D	15	0.14
997 Don't Know	18	0.17
999 Refuse	1	0.01
Total	10,734	100.00

xchildedu_2_: ZChildName[2]'s Highest Degree (update after CA007)

	Freq.	%
1 No Formal Education(Illiterate)	342	3.87
2 Did not Finish Primary School	980	11.09
3 Sishu/Home School	1	0.01
4 Elementary School	1,895	21.44
5 Middle School	2,954	33.42
6 High School	857	9.69
7 Vocational School	530	6.00
8 Two-/Three-Year College/Associate Degree	558	6.31
9 Four-Year College/Bachelor's Degree	642	7.26
10 Master's Degree	53	0.60
11 Doctoral Degree/Ph.D	8	0.09
997 Don't Know	18	0.20
999 Refuse	2	0.02
Total	8,840	100.00

xchildedu_3_: ZChildName[3]'s Highest Degree (update after CA007)

	Freq.	%
1 No Formal Education(Illiterate)	317	6.54
2 Did not Finish Primary School	642	13.25
4 Elementary School	1,196	24.69
5 Middle School	1,596	32.95
6 High School	410	8.46
7 Vocational School	219	4.52
8 Two-/Three-Year College/Associate Degree	219	4.52
9 Four-Year College/Bachelor's Degree	213	4.40
10 Master's Degree	19	0.39
11 Doctoral Degree/Ph.D	3	0.06
997 Don't Know	9	0.19
999 Refuse	1	0.02
Total	4,844	100.00

xchildedu_4_: ZChildName[4]'s Highest Degree (update after CA007)

	Freq.	%
1 No Formal Education(Illiterate)	211	8.42
2 Did not Finish Primary School	405	16.15
3 Sishu/Home School	1	0.04
4 Elementary School	683	27.24
5 Middle School	733	29.24
6 High School	185	7.38
7 Vocational School	88	3.51
8 Two-/Three-Year College/Associate Degree	83	3.31
9 Four-Year College/Bachelor's Degree	94	3.75
10 Master's Degree	6	0.24
11 Doctoral Degree/Ph.D	2	0.08

997 Don't Know	15	0.60
999 Refuse	1	0.04
Total	2,507	100.00

xchildedu_5_: ZChildName[5]'s Highest Degree (update after CA007)

	Freq.	%
1 No Formal Education(Illiterate)	164	14.10
2 Did not Finish Primary School	199	17.11
4 Elementary School	304	26.14
5 Middle School	312	26.83
6 High School	77	6.62
7 Vocational School	37	3.18
8 Two-/Three-Year College/Associate Degree	19	1.63
9 Four-Year College/Bachelor's Degree	34	2.92
10 Master's Degree	4	0.34
11 Doctoral Degree/Ph.D	2	0.17
997 Don't Know	11	0.95
Total	1,163	100.00

xchildedu_6_: ZChildName[6]'s Highest Degree (update after CA007)

	Freq.	%
1 No Formal Education(Illiterate)	82	16.63
2 Did not Finish Primary School	90	18.26
3 Sishu/Home School	1	0.20
4 Elementary School	118	23.94
5 Middle School	130	26.37
6 High School	28	5.68
7 Vocational School	14	2.84
8 Two-/Three-Year College/Associate Degree	7	1.42
9 Four-Year College/Bachelor's Degree	14	2.84
10 Master's Degree	1	0.20
997 Don't Know	8	1.62
Total	493	100.00

xchildedu_7_: ZChildName[7]'s Highest Degree (update after CA007)

	Freq.	%
1 No Formal Education(Illiterate)	50	22.12
2 Did not Finish Primary School	43	19.03
4 Elementary School	54	23.89
5 Middle School	46	20.35
6 High School	14	6.19
7 Vocational School	8	3.54
8 Two-/Three-Year College/Associate Degree	1	0.44
9 Four-Year College/Bachelor's Degree	7	3.10
10 Master's Degree	1	0.44
997 Don't Know	2	0.88
Total	226	100.00

xchildedu_8_: ZChildName[8]'s Highest Degree (update after CA007)

	Freq.	%
1 No Formal Education(Illiterate)	20	21.98
2 Did not Finish Primary School	18	19.78
4 Elementary School	20	21.98
5 Middle School	19	20.88
6 High School	5	5.49
7 Vocational School	2	2.20
8 Two-/Three-Year College/Associate Degree	4	4.40
9 Four-Year College/Bachelor's Degree	1	1.10
997 Don't Know	2	2.20
Total	91	100.00

xchildedu_9_: ZChildName[9]'s Highest Degree (update after CA007)

	Freq.	%
1 No Formal Education(Illiterate)	9	25.00
2 Did not Finish Primary School	8	22.22
4 Elementary School	5	13.89
5 Middle School	11	30.56
6 High School	1	2.78
8 Two-/Three-Year College/Associate Degree	1	2.78
997 Don't Know	1	2.78
Total	36	100.00

xchildedu_10_: ZChildName[10]'s Highest Degree (update after CA007)

	Freq.	%
1 No Formal Education(Illiterate)	4	21.05
2 Did not Finish Primary School	4	21.05
4 Elementary School	4	21.05
5 Middle School	4	21.05
8 Two-/Three-Year College/Associate Degree	3	15.79
Total	19	100.00

xchildedu_11_: ZChildName[11]'s Highest Degree (update after CA007)

	Freq.	%
1 No Formal Education(Illiterate)	1	12.50
2 Did not Finish Primary School	1	12.50
4 Elementary School	4	50.00
5 Middle School	1	12.50
997 Don't Know	1	12.50
Total	8	100.00

xchildedu_12_: ZChildName[12]'s Highest Degree (update after CA007)

	Freq.	%
2 Did not Finish Primary School	1	33.33
5 Middle School	2	66.67
Total	3	100.00

xchildedu_13_: ZChildName[13]'s Highest Degree (update after CA007)

	Freq.	%
4 Elementary School	1	100.00
Total	1	100.00

xchildedu_14_: ZChildName[14]'s Highest Degree (update after CA007)

No Observations

xchildedu_15_: ZChildName[15]'s Highest Degree (update after CA007)

No Observations

xchildedu_16_: ZChildName[16]'s Highest Degree (update after CA007)

No Observations

xchildedu_17_: ZChildName[17]'s Highest Degree (update after CA007)

	Freq.	%
1 No Formal Education(Illiterate)	1	100.00
Total	1	100.00

zchildnum: Number of Children

Mean	SD	Min	Max	Obs
2.72	1.47	1.00	17.00	11,192

zchildbirth_1_: ZChildName[1]'s Birth Year

Mean	SD	Min	Max	Obs
1,981.53	10.57	1,942.00	2,015.00	10,789

zchildbirth_2_: ZChildName[2]'s Birth Year

Mean	SD	Min	Max	Obs
1,981.19	11.34	1,903.00	2,018.00	8,879

zchildbirth_3_: ZChildName[3]'s Birth Year

Mean	SD	Min	Max	Obs
1,978.10	10.65	1,900.00	2,018.00	4,889

zchildbirth_4_: ZChildName[4]'s Birth Year

Mean	SD	Min	Max	Obs
1,975.91	10.12	1,922.00	2,018.00	2,542

zchildbirth_5_: ZChildName[5]'s Birth Year

Mean	SD	Min	Max	Obs
1,974.29	9.77	1,948.00	2,015.00	1,174

zchildbirth_6_: ZChildName[6]'s Birth Year

Mean	SD	Min	Max	Obs
1,973.91	9.45	1,947.00	2,015.00	505

zchildbirth_7_: ZChildName[7]'s Birth Year

Mean	SD	Min	Max	Obs
1,973.54	10.99	1,948.00	2,015.00	230

zchildbirth_8_: ZChildName[8]'s Birth Year

Mean	SD	Min	Max	Obs
1,974.52	12.77	1,950.00	2,015.00	98

zchildbirth_9_: ZChildName[9]'s Birth Year

Mean	SD	Min	Max	Obs
1,973.63	11.05	1,955.00	2,015.00	38

zchildbirth_10_: ZChildName[10]'s Birth Year

Mean	SD	Min	Max	Obs
1,972.86	10.46	1,961.00	2,015.00	21

zchildbirth_11_: ZChildName[11]'s Birth Year

Mean	SD	Min	Max	Obs
1,974.33	7.45	1,963.00	1,986.00	9

zchildbirth_12_: ZChildName[12]'s Birth Year

Mean	SD	Min	Max	Obs
1,981.33	5.86	1,977.00	1,988.00	3

zchildbirth_13_: ZChildName[13]'s Birth Year

Mean	SD	Min	Max	Obs
1,988.00	.	1,988.00	1,988.00	1

zchildbirth_14_: ZChildName[14]'s Birth Year

No Observations

zchildbirth_15_: ZChildName[15]'s Birth Year

No Observations

zchildbirth_16_: ZChildName[16]'s Birth Year

No Observations

zchildbirth_17_: ZChildName[17]'s Birth Year

Mean	SD	Min	Max	Obs
1,981.00	.	1,981.00	1,981.00	1

zchildgender_1_: ZChildName[1]'s Gender

	Freq.	%
1 Male	7,091	65.97
2 Female	3,658	34.03
Total	10,749	100.00

zchildgender_2_: ZChildName[2]'s Gender

	Freq.	%
1 Male	4,476	50.56
2 Female	4,377	49.44
Total	8,853	100.00

zchildgender_3_: ZChildName[3]'s Gender

	Freq.	%
1 Male	2,036	41.76
2 Female	2,840	58.24
Total	4,876	100.00

zchildgender_4_: ZChildName[4]'s Gender

	Freq.	%
1 Male	900	35.43
2 Female	1,640	64.57
Total	2,540	100.00

zchildgender_5_: ZChildName[5]'s Gender

	Freq.	%
1 Male	363	30.79
2 Female	816	69.21
Total	1,179	100.00

zchildgender_6_: ZChildName[6]'s Gender

	Freq.	%
1 Male	142	28.06
2 Female	364	71.94
Total	506	100.00

zchildgender_7_: ZChildName[7]'s Gender

	Freq.	%
1 Male	69	30.00
2 Female	161	70.00
Total	230	100.00

zchildgender_8_: ZChildName[8]'s Gender

	Freq.	%
1 Male	27	27.27
2 Female	72	72.73
Total	99	100.00

zchildgender_9_: ZChildName[9]'s Gender

	Freq.	%
1 Male	8	20.51
2 Female	31	79.49
Total	39	100.00

zchildgender_10_: ZChildName[10]'s Gender

	Freq.	%
1 Male	9	42.86
2 Female	12	57.14
Total	21	100.00

zchildgender_11_: ZChildName[11]'s Gender

	Freq.	%
1 Male	2	22.22
2 Female	7	77.78
Total	9	100.00

zchildgender_12_: ZChildName[12]'s Gender

	Freq.	%
1 Male	1	33.33
2 Female	2	66.67
Total	3	100.00

zchildgender_13_: ZChildName[13]'s Gender

	Freq.	%
1 Male	1	100.00
Total	1	100.00

zchildgender_14_: ZChildName[14]'s Gender

No Observations

zchildgender_15_: ZChildName[15]'s Gender

No Observations

zchildgender_16_: ZChildName[16]'s Gender

No Observations

zchildgender_17_: ZChildName[17]'s Gender

	Freq.	%
2 Female	1	100.00
Total	1	100.00

zchildedu_1_: ZChildName[1]'s Highest Degree

	Freq.	%
1 No Formal Education(Illiterate)	263	2.64
2 Did not Finish Primary School	802	8.04
4 Elementary School	1,874	18.78
5 Middle School	3,326	33.33
6 High School	1,293	12.96
7 Vocational School	664	6.65
8 Two-/Three-Year College/Associate Degree	769	7.71
9 Four-Year College/Bachelor's Degree	873	8.75
10 Master's Degree	101	1.01
11 Doctoral Degree/Ph.D	14	0.14
Total	9,979	100.00

zchildedu_2_: ZChildName[2]'s Highest Degree

	Freq.	%

	Freq.	%
1 No Formal Education(Illiterate)	325	3.96
2 Did not Finish Primary School	925	11.28
3 Sishu/Home School	1	0.01
4 Elementary School	1,807	22.03
5 Middle School	2,764	33.70
6 High School	786	9.58
7 Vocational School	488	5.95
8 Two-/Three-Year College/Associate Degree	496	6.05
9 Four-Year College/Bachelor's Degree	562	6.85
10 Master's Degree	42	0.51
11 Doctoral Degree/Ph.D	6	0.07
Total	8,202	100.00

zchildedu_3_: ZChildName[3]'s Highest Degree

	Freq.	%
1 No Formal Education(Illiterate)	294	6.51
2 Did not Finish Primary School	599	13.27
4 Elementary School	1,135	25.14
5 Middle School	1,507	33.38
6 High School	371	8.22
7 Vocational School	199	4.41
8 Two-/Three-Year College/Associate Degree	196	4.34
9 Four-Year College/Bachelor's Degree	195	4.32
10 Master's Degree	16	0.35
11 Doctoral Degree/Ph.D	3	0.07
Total	4,515	100.00

zchildedu_4_: ZChildName[4]'s Highest Degree

	Freq.	%
1 No Formal Education(Illiterate)	199	8.64
2 Did not Finish Primary School	380	16.50
4 Elementary School	638	27.70
5 Middle School	690	29.96
6 High School	166	7.21
7 Vocational School	76	3.30
8 Two-/Three-Year College/Associate Degree	66	2.87
9 Four-Year College/Bachelor's Degree	81	3.52
10 Master's Degree	6	0.26
11 Doctoral Degree/Ph.D	1	0.04
Total	2,303	100.00

zchildedu_5_: ZChildName[5]'s Highest Degree

	Freq.	%
1 No Formal Education(Illiterate)	156	14.80
2 Did not Finish Primary School	177	16.79
4 Elementary School	282	26.76
5 Middle School	292	27.70
6 High School	66	6.26
7 Vocational School	34	3.23
8 Two-/Three-Year College/Associate Degree	15	1.42
9 Four-Year College/Bachelor's Degree	28	2.66
10 Master's Degree	3	0.28

	Freq.	%
11 Doctoral Degree/Ph.D	1	0.09
Total	1,054	100.00

zchildedu_6_: ZChildName[6]'s Highest Degree

	Freq.	%
1 No Formal Education(Illiterate)	70	15.45
2 Did not Finish Primary School	91	20.09
3 Sishu/Home School	1	0.22
4 Elementary School	112	24.72
5 Middle School	122	26.93
6 High School	25	5.52
7 Vocational School	10	2.21
8 Two-/Three-Year College/Associate Degree	8	1.77
9 Four-Year College/Bachelor's Degree	13	2.87
10 Master's Degree	1	0.22
Total	453	100.00

zchildedu_7_: ZChildName[7]'s Highest Degree

	Freq.	%
1 No Formal Education(Illiterate)	41	20.92
2 Did not Finish Primary School	41	20.92
4 Elementary School	50	25.51
5 Middle School	38	19.39
6 High School	11	5.61
7 Vocational School	7	3.57
8 Two-/Three-Year College/Associate Degree	1	0.51
9 Four-Year College/Bachelor's Degree	6	3.06
10 Master's Degree	1	0.51
Total	196	100.00

zchildedu_8_: ZChildName[8]'s Highest Degree

	Freq.	%
1 No Formal Education(Illiterate)	21	23.86
2 Did not Finish Primary School	19	21.59
4 Elementary School	19	21.59
5 Middle School	18	20.45
6 High School	5	5.68
7 Vocational School	2	2.27
8 Two-/Three-Year College/Associate Degree	3	3.41
9 Four-Year College/Bachelor's Degree	1	1.14
Total	88	100.00

zchildedu_9_: ZChildName[9]'s Highest Degree

	Freq.	%
1 No Formal Education(Illiterate)	7	20.59
2 Did not Finish Primary School	8	23.53
4 Elementary School	5	14.71
5 Middle School	10	29.41
6 High School	2	5.88

8 Two-/Three-Year College/Associate Degree	2	5.88
Total	34	100.00

zchildedu_10_: ZChildName[10]'s Highest Degree

	Freq.	%
1 No Formal Education(Illiterate)	4	23.53
2 Did not Finish Primary School	5	29.41
4 Elementary School	4	23.53
5 Middle School	2	11.76
8 Two-/Three-Year College/Associate Degree	2	11.76
Total	17	100.00

zchildedu_11_: ZChildName[11]'s Highest Degree

	Freq.	%
1 No Formal Education(Illiterate)	1	25.00
2 Did not Finish Primary School	1	25.00
4 Elementary School	2	50.00
Total	4	100.00

zchildedu_12_: ZChildName[12]'s Highest Degree

	Freq.	%
2 Did not Finish Primary School	1	50.00
5 Middle School	1	50.00
Total	2	100.00

versionID: Version ID

A String Variable	
Obs:	11,406

This page intentionally left blank

MODULE D

Health Status and Functioning

ID: Individual ID

A String Variable	
Obs:	19,367

householdID: Household ID

A String Variable	
Obs:	19,367

communityID: Community ID

A String Variable	
Obs:	19,367

proxy: Proxy

	Freq.	%
1 Yes	1,784	100.00
Total	1,784	100.00

proxy_5: Proxy for Health Module

	Freq.	%
1 Yes	1,774	99.44
2 No	10	0.56
Total	1,784	100.00

da001: Self-Reported Health Status

	Freq.	%

1 Very Good	2,107	11.98
2 Good	2,245	12.76
3 Fair	8,860	50.36
4 Poor	3,194	18.16
5 Very Poor	1,170	6.65
997 Don't Know	16	0.09
Total	17,592	100.00

da002_1_: Doctor Diagnosed Disease [1] Compared to ZIWTime

	Freq.	%
1 Better	1,570	23.93
2 Worse	1,242	18.93
3 Same	3,465	52.80
99 Never Had the Disease	285	4.34
Total	6,562	100.00

da002_2_: Doctor Diagnosed Disease [2] Compared to ZIWTime

	Freq.	%
1 Better	735	19.74
2 Worse	757	20.33
3 Same	1,974	53.01
99 Never Had the Disease	258	6.93
Total	3,724	100.00

da002_3_: Doctor Diagnosed Disease [3] Compared to ZIWTime

	Freq.	%
1 Better	418	19.32
2 Worse	518	23.94
3 Same	1,018	47.04
99 Never Had the Disease	210	9.70
Total	2,164	100.00

da002_4_: Doctor Diagnosed Disease [4] Compared to ZIWTime

	Freq.	%
1 Better	159	47.18
2 Worse	30	8.90
3 Same	88	26.11
99 Never Had the Disease	60	17.80
Total	337	100.00

da002_5_: Doctor Diagnosed Disease [5] Compared to ZIWTime

	Freq.	%
1 Better	570	25.31
2 Worse	565	25.09
3 Same	815	36.19
99 Never Had the Disease	302	13.41
Total	2,252	100.00

da002_6_: Doctor Diagnosed Disease [6] Compared to ZIWTime

	Freq.	%
1 Better	225	19.81
2 Worse	191	16.81
3 Same	534	47.01
99 Never Had the Disease	186	16.37
Total	1,136	100.00

da002_7_: Doctor Diagnosed Disease [7] Compared to ZIWTime

	Freq.	%
1 Better	784	24.00
2 Worse	800	24.49
3 Same	1,415	43.31
99 Never Had the Disease	268	8.20
Total	3,267	100.00

da002_8_: Doctor Diagnosed Disease [8] Compared to ZIWTime

	Freq.	%
1 Better	343	28.85
2 Worse	252	21.19
3 Same	370	31.12
99 Never Had the Disease	224	18.84
Total	1,189	100.00

da002_9_: Doctor Diagnosed Disease [9] Compared to ZIWTime

	Freq.	%
1 Better	317	19.96
2 Worse	396	24.94
3 Same	658	41.44
99 Never Had the Disease	217	13.66
Total	1,588	100.00

da002_10_: Doctor Diagnosed Disease [10] Compared to ZIWTime

	Freq.	%
1 Better	1,419	26.76
2 Worse	1,146	21.61
3 Same	2,413	45.50
99 Never Had the Disease	325	6.13
Total	5,303	100.00

da002_11_: Doctor Diagnosed Disease [11] Compared to ZIWTime

	Freq.	%
1 Better	102	26.56
2 Worse	112	29.17
3 Same	128	33.33

	Freq.	%
99 Never Had the Disease	42	10.94
Total	384	100.00

da002_12_: Doctor Diagnosed Disease [12] Compared to ZIWTime

	Freq.	%
2 Worse	4	23.53
3 Same	4	23.53
99 Never Had the Disease	9	52.94
Total	17	100.00

da002_13_: Doctor Diagnosed Disease [13] Compared to ZIWTime

	Freq.	%
1 Better	24	6.98
2 Worse	28	8.14
3 Same	39	11.34
99 Never Had the Disease	253	73.55
Total	344	100.00

da002_14_: Doctor Diagnosed Disease [14] Compared to ZIWTime

	Freq.	%
1 Better	1,292	19.79
2 Worse	2,393	36.65
3 Same	2,377	36.40
99 Never Had the Disease	468	7.17
Total	6,530	100.00

da002_15_: Doctor Diagnosed Disease [15] Compared to ZIWTime

	Freq.	%
1 Better	213	24.01
2 Worse	291	32.81
3 Same	303	34.16
99 Never Had the Disease	80	9.02
Total	887	100.00

da002_1_1_: Self-know Disease [1] Compared to ZIWTime

	Freq.	%
1 Better	21	28.77
2 Worse	3	4.11
3 Same	14	19.18
99 Never Had the Disease	35	47.95
Total	73	100.00

da002_1_5_: Self-know Disease [5] Compared to ZIWTime

	Freq.	%

	Freq.	%
1 Better	30	19.61
2 Worse	17	11.11
3 Same	45	29.41
99 Never Had the Disease	61	39.87
Total	153	100.00

da002_1_11_: Self-know Disease [11] Compared to ZIWTime

	Freq.	%
1 Better	18	13.43
2 Worse	22	16.42
3 Same	38	28.36
99 Never Had the Disease	56	41.79
Total	134	100.00

da003_1_: Diagnosed Disease [1]

	Freq.	%
1 Yes	1,450	11.08
2 No	11,639	88.92
Total	13,089	100.00

da003_2_: Diagnosed Disease [2]

	Freq.	%
1 Yes	1,637	10.30
2 No	14,261	89.70
Total	15,898	100.00

da003_3_: Diagnosed Disease [3]

	Freq.	%
1 Yes	900	5.17
2 No	16,510	94.83
Total	17,410	100.00

da003_4_: Diagnosed Disease [4]

	Freq.	%
1 Yes	214	1.12
2 No	18,873	98.88
Total	19,087	100.00

da003_5_: Diagnosed Disease [5]

	Freq.	%
1 Yes	809	4.65
2 No	16,605	95.35
Total	17,414	100.00

da003_6_: Diagnosed Disease [6]

	Freq.	%
1 Yes	444	2.41
2 No	17,970	97.59
Total	18,414	100.00

da003_7_: Diagnosed Disease [7]

	Freq.	%
1 Yes	1,017	6.21
2 No	15,347	93.79
Total	16,364	100.00

da003_8_: Diagnosed Disease [8]

	Freq.	%
1 Yes	416	2.26
2 No	17,982	97.74
Total	18,398	100.00

da003_9_: Diagnosed Disease [9]

	Freq.	%
1 Yes	630	3.50
2 No	17,362	96.50
Total	17,992	100.00

da003_10_: Diagnosed Disease [10]

	Freq.	%
1 Yes	1,112	7.73
2 No	13,273	92.27
Total	14,385	100.00

da003_11_: Diagnosed Disease [11]

	Freq.	%
1 Yes	241	1.27
2 No	18,780	98.73
Total	19,021	100.00

da003_12_: Diagnosed Disease [12]

	Freq.	%
1 Yes	799	4.13
2 No	18,556	95.87
Total	19,355	100.00

da003_13_: Diagnosed Disease [13]

	Freq.	%
1 Yes	178	0.92
2 No	19,094	99.08
Total	19,272	100.00

da003_14_: Diagnosed Disease [14]

	Freq.	%
1 Yes	1,350	10.15
2 No	11,951	89.85
Total	13,301	100.00

da003_15_: Diagnosed Disease [15]

	Freq.	%
1 Yes	365	1.97
2 No	18,191	98.03
Total	18,556	100.00

da004_1_: Self-known Disease [1]

	Freq.	%
1 Yes	172	1.48
2 No	10,011	86.29
3 Don't Know	1,418	12.22
Total	11,601	100.00

da004_5_: Self-known Disease [5]

	Freq.	%
1 Yes	286	1.73
2 No	13,216	80.03
3 Don't Know	3,011	18.23
Total	16,513	100.00

da004_11_: Self-known Disease [11]

	Freq.	%
1 Yes	312	1.67
2 No	14,785	79.06
3 Don't Know	3,605	19.28
Total	18,702	100.00

da005: Out-patient Service

	Freq.	%
1 Yes	3,966	20.48
2 No	15,396	79.52

	Freq.	%
Total	19,362	100.00

da006: Out-patient Service Times

Mean	SD	Min	Max	Obs
2.23	2.56	0.00	31.00	3,966

da007: In-patient Service

	Freq.	%
1 Yes	3,736	19.30
2 No	15,625	80.70
Total	19,361	100.00

da008: In-patient Service Times

Mean	SD	Min	Max	Obs
1.71	2.09	1.00	84.00	3,736

da009: Delayed Medical Service

	Freq.	%
1 Yes	2,184	11.28
2 No	17,177	88.72
Total	19,361	100.00

da010_s1: Delayed Medical Service 1: In-patient Service, Big Surgery

	Freq.	%
0 No	2,080	95.24
1 Yes	104	4.76
Total	2,184	100.00

da010_s2: Delayed Medical Service 1: Out-patient Service, Small Surgery

	Freq.	%
0 No	2,106	96.43
2 Yes	78	3.57
Total	2,184	100.00

da010_s3: Delayed Medical Service 1: Out-patient Service

	Freq.	%
0 No	1,054	48.26
3 Yes	1,130	51.74
Total	2,184	100.00

da010_s4: Delayed Medical Service 1: Prescribtion for medicine

	Freq.	%
0 No	1,658	75.92
4 Yes	526	24.08
Total	2,184	100.00

da010_s5: Delayed Medical Service 1: Dental care

	Freq.	%
0 No	1,522	69.69
5 Yes	662	30.31
Total	2,184	100.00

da010_s6: Delayed Medical Service 1: Others, Please Specify

	Freq.	%
0 No	1,909	87.41
6 Yes	275	12.59
Total	2,184	100.00

da011_s1: Reason for Out-patient Service: New Symptoms

	Freq.	%
0 No	840	74.34
1 Yes	290	25.66
Total	1,130	100.00

da011_s2: Reason for Out-patient Service: Prevalent Disease

	Freq.	%
0 No	341	30.18
2 Yes	789	69.82
Total	1,130	100.00

da011_s3: Reason for Out-patient Service: Regular Physical Examination

	Freq.	%
0 No	1,014	89.73
3 Yes	116	10.27
Total	1,130	100.00

da012_s1: Reason for Delayed: Unavailable for Appointment

	Freq.	%
0 No	1,780	81.50
1 Yes	404	18.50
Total	2,184	100.00

da012_s2: Reason for Delayed: Change Scedule of Hospital

	Freq.	%
0 No	2,042	93.50
2 Yes	142	6.50
Total	2,184	100.00

da012_s3: Reason for Delayed: I decided to Visit Later

	Freq.	%
0 No	1,630	74.63
3 Yes	554	25.37
Total	2,184	100.00

da012_s4: Reason for Delayed: I am Afraid to go to Hospital

	Freq.	%
0 No	1,591	72.85
4 Yes	593	27.15
Total	2,184	100.00

da012_s5: Reason for Delayed: Others, Please Specify

	Freq.	%
0 No	1,282	58.70
5 Yes	902	41.30
Total	2,184	100.00

da013: RIW: Time for the Latest Physical Examination

	Freq.	%
1 Year and Month	9,052	47.07
2 No Physical Examination Since ZIWTime	10,178	52.93
Total	19,230	100.00

da013_1: Year

Mean	SD	Min	Max	Obs
2,019.49	0.65	2,008.00	2,020.00	9,052

da013_2: Month

Mean	SD	Min	Max	Obs
5.18	3.24	-1.00	12.00	9,052

da014: NIW: Time for the Latest Physical Examination

	Freq.	%
1 Year and Month	67	51.54

	Freq.	%
2 No Physical Examination Lifetime	63	48.46
Total	130	100.00

da014_1: Year

Mean	SD	Min	Max	Obs
2,018.63	3.96	1,988.00	2,020.00	67

da014_2: Month

Mean	SD	Min	Max	Obs
5.16	3.30	-1.00	12.00	67

da019: RIW: Traffic Accident/Injury and Received Medical Treatment Since ZIW-Time

	Freq.	%
1 Yes	517	2.69
2 No	18,713	97.31
Total	19,230	100.00

da020: NIW: Traffic Accident/Injury and Received Medical Treatment

	Freq.	%
1 Yes	15	11.54
2 No	115	88.46
Total	130	100.00

da021: Traffic Accident/Injury Limit Daily Activities

	Freq.	%
1 Yes	279	52.44
2 No	253	47.56
Total	532	100.00

da022: Fallen Down since ZIWTime

	Freq.	%
1 Yes	3,315	17.24
2 No	15,915	82.76
Total	19,230	100.00

da023: Fallen Down

	Freq.	%
1 Yes	40	30.77
2 No	90	69.23
Total	130	100.00

da024: Times Fallen Down Needing Medical Treatment

Mean	SD	Min	Max	Obs
0.54	0.93	0.00	15.00	3,355

da025: Fractured Hip since ZIWTime

	Freq.	%
1 Yes	164	0.85
2 No	19,066	99.15
Total	19,230	100.00

da026: Fractured Hip

	Freq.	%
1 Yes	3	2.31
2 No	127	97.69
Total	130	100.00

da027: Troubled with Body Pain

	Freq.	%
1 None	8,132	42.01
2 A little	5,697	29.43
3 Somewhat	1,993	10.30
4 Quite a Bit	1,729	8.93
5 Very	1,806	9.33
Total	19,357	100.00

da028_s1: Body Parts Feeling Pain

	Freq.	%
0 No	7,216	64.29
1 Head (Headache)	4,008	35.71
Total	11,224	100.00

da028_s2: Body Parts Feeling Pain

	Freq.	%
0 No	7,222	64.34
2 Shoulder	4,002	35.66
Total	11,224	100.00

da028_s3: Body Parts Feeling Pain

	Freq.	%
0 No	8,151	72.62
3 Arm	3,073	27.38
Total	11,224	100.00

da028_s4: Body Parts Feeling Pain

	Freq.	%
0 No	9,222	82.16
4 Wrist	2,002	17.84
Total	11,224	100.00

da028_s5: Body Parts Feeling Pain

	Freq.	%
0 No	8,921	79.48
5 Fingers	2,303	20.52
Total	11,224	100.00

da028_s6: Body Parts Feeling Pain

	Freq.	%
0 No	9,559	85.17
6 Chest	1,665	14.83
Total	11,224	100.00

da028_s7: Body Parts Feeling Pain

	Freq.	%
0 No	8,500	75.73
7 Stomach (Stomachache)	2,724	24.27
Total	11,224	100.00

da028_s8: Body Parts Feeling Pain

	Freq.	%
0 No	8,505	75.78
8 Back	2,719	24.22
Total	11,224	100.00

da028_s9: Body Parts Feeling Pain

	Freq.	%
0 No	4,938	44.00
9 Waist	6,286	56.00
Total	11,224	100.00

da028_s10: Body Parts Feeling Pain

	Freq.	%
0 No	9,658	86.05
10 Buttocks	1,566	13.95
Total	11,224	100.00

da028_s11: Body Parts Feeling Pain

	Freq.	%
0 No	6,688	59.59
11 Leg	4,536	40.41
Total	11,224	100.00

da028_s12: Body Parts Feeling Pain

	Freq.	%
0 No	6,309	56.21
12 Knees	4,915	43.79
Total	11,224	100.00

da028_s13: Body Parts Feeling Pain

	Freq.	%
0 No	9,080	80.90
13 Ankle	2,144	19.10
Total	11,224	100.00

da028_s14: Body Parts Feeling Pain

	Freq.	%
0 No	9,826	87.54
14 Toes	1,398	12.46
Total	11,224	100.00

da028_s15: Body Parts Feeling Pain

	Freq.	%
0 No	8,387	74.72
15 Neck	2,837	25.28
Total	11,224	100.00

da028_s16: Body Parts Feeling Pain

	Freq.	%
0 No	9,661	86.07
16 Other, Please Specify	1,563	13.93
Total	11,224	100.00

da029: Life Expectancy

	Freq.	%
1 Almost Impossible	2,056	11.64
2 Not Very Likely	2,175	12.31
3 Maybe	6,066	34.34
4 Very Likely	1,944	11.01
5 Almost Certain	2,563	14.51

997 Don't Know	2,858	16.18
Total	17,662	100.00

da030: During Last Month Average Hours of Actual Sleep

Mean	SD	Min	Max	Obs
5.96	2.14	-1.00	24.00	19,355

da031: During Last Month Time for a Nap after Lunch

Mean	SD	Min	Max	Obs
42.20	45.73	-1.00	300.00	19,355

da032_1_: Intensive Physical Activity More than 10 Mins Each Time

	Freq.	%
1 Yes	6,886	35.58
2 No	12,467	64.42
Total	19,353	100.00

da032_2_: Moderate Physical Activity More than 10 Mins Each Time

	Freq.	%
1 Yes	10,759	55.59
2 No	8,594	44.41
Total	19,353	100.00

da032_3_: Light Physical Activity More than 10 Mins Each Time

	Freq.	%
1 Yes	14,879	76.89
2 No	4,472	23.11
Total	19,351	100.00

da033_1_: Days with Intensive Physical Activity

Mean	SD	Min	Max	Obs
4.83	2.17	1.00	7.00	6,886

da033_2_: Days with Moderate Physical Activity

Mean	SD	Min	Max	Obs
5.14	2.17	1.00	7.00	10,759

da033_3_: Days with Light Physical Activity

Mean	SD	Min	Max	Obs

5.94	1.75	1.00	7.00	14,879

da034_1_: Time with Intensive Physical Activity

	Freq.	%
1 <2 Hours	1,826	26.52
2 >=2 Hours	5,060	73.48
Total	6,886	100.00

da034_2_: Time with Moderate Physical Activity

	Freq.	%
1 <2 Hours	6,205	57.67
2 >=2 Hours	4,554	42.33
Total	10,759	100.00

da034_3_: Time with Light Physical Activity

	Freq.	%
1 <2 Hours	9,515	63.95
2 >=2 Hours	5,364	36.05
Total	14,879	100.00

da035_1_: Time with Intensive Physical Activity

	Freq.	%
1 <30 Minutes	332	18.18
2 >=30 Minutes	1,494	81.82
Total	1,826	100.00

da035_2_: Time with Moderate Physical Activity

	Freq.	%
1 <30 Minutes	1,582	25.50
2 >=30 Minutes	4,623	74.50
Total	6,205	100.00

da035_3_: Time with Light Physical Activity

	Freq.	%
1 <30 Minutes	1,814	19.06
2 >=30 Minutes	7,701	80.94
Total	9,515	100.00

da036_1_: Time with Intensive Physical Activity

	Freq.	%
1 <4 Hours	1,775	35.08
2 >=4 Hours	3,285	64.92

Total	5,060	100.00

da036_2_: Time with Moderate Physical Activity

	Freq.	%
1 <4 Hours	2,540	55.79
2 >=4 Hours	2,013	44.21
Total	4,553	100.00

da036_3_: Time with Light Physical Activity

	Freq.	%
1 <4 Hours	3,376	62.94
2 >=4 Hours	1,988	37.06
Total	5,364	100.00

da037_1_: Purpose for Intensive Physical Activity

	Freq.	%
1 Job Demands	5,032	73.08
2 Entertainment	165	2.40
3 Exercise	1,153	16.74
4 Others, Please Specify	536	7.78
Total	6,886	100.00

da037_2_: Purpose for Moderate Physical Activity

	Freq.	%
1 Job Demands	4,867	45.24
2 Entertainment	377	3.50
3 Exercise	2,408	22.38
4 Others, Please Specify	3,107	28.88
Total	10,759	100.00

da037_3_: Purpose for Light Physical Activity

	Freq.	%
1 Job Demands	4,420	29.71
2 Entertainment	1,588	10.67
3 Exercise	7,476	50.25
4 Others, Please Specify	1,395	9.38
Total	14,879	100.00

da038_s1: Activities in Last Month

	Freq.	%
0 No	13,165	68.03
1 Interacted With Friends	6,186	31.97
Total	19,351	100.00

da038_s2: Activities in Last Month

	Freq.	%
0 No	16,517	85.35
2 Played Ma-jong, Played Chess, Played Cards, or Went to Community Club	2,834	14.65
Total	19,351	100.00

da038_s3: Activities in Last Month

	Freq.	%
0 No	16,362	84.55
3 Provided Help to Family, Friends, or Neighbors Who Do Not Live With You	2,989	15.45
Total	19,351	100.00

da038_s4: Activities in Last Month

	Freq.	%
0 No	18,023	93.14
4 Went to a Sport, Social, or Other Kind of Club	1,328	6.86
Total	19,351	100.00

da038_s5: Activities in Last Month

	Freq.	%
0 No	18,891	97.62
5 Took Part in a Community-Related Organization	460	2.38
Total	19,351	100.00

da038_s6: Activities in Last Month

	Freq.	%
0 No	18,743	96.86
6 Done Voluntary or Charity work, or Cared for a Sick or Disabled Adult Who Does Not Live With You	608	3.14
Total	19,351	100.00

da038_s7: Activities in Last Month

	Freq.	%
0 No	19,044	98.41
7 Attended an Educational or Training Course	307	1.59
Total	19,351	100.00

da038_s8: Activities in Last Month

	Freq.	%
0 No	18,988	98.12
8 Others, Please Specify	363	1.88

	Freq.	%
Total	19,351	100.00

da038_s9: Activities in Last Month

	Freq.	%
0 No	9,296	48.04
9 None of These	10,055	51.96
Total	19,351	100.00

da039_1_: Frequency of Activities[1]

	Freq.	%
1 Almost Daily	1,900	30.71
2 Almost Every Week	1,348	21.79
3 Not Regularly	2,938	47.49
Total	6,186	100.00

da039_2_: Frequency of Activities[2]

	Freq.	%
1 Almost Daily	715	25.23
2 Almost Every Week	829	29.25
3 Not Regularly	1,290	45.52
Total	2,834	100.00

da039_3_: Frequency of Activities[3]

	Freq.	%
1 Almost Daily	216	7.23
2 Almost Every Week	522	17.46
3 Not Regularly	2,251	75.31
Total	2,989	100.00

da039_4_: Frequency of Activities[4]

	Freq.	%
1 Almost Daily	782	58.89
2 Almost Every Week	242	18.22
3 Not Regularly	304	22.89
Total	1,328	100.00

da039_5_: Frequency of Activities[5]

	Freq.	%
1 Almost Daily	30	6.52
2 Almost Every Week	105	22.83
3 Not Regularly	325	70.65
Total	460	100.00

da039_6_: Frequency of Activities[6]

	Freq.	%
1 Almost Daily	62	10.20
2 Almost Every Week	109	17.93
3 Not Regularly	437	71.88
Total	608	100.00

da039_7_: Frequency of Activities[7]

	Freq.	%
1 Almost Daily	31	10.10
2 Almost Every Week	54	17.59
3 Not Regularly	222	72.31
Total	307	100.00

da039_8_: Frequency of Activities[8]

	Freq.	%
1 Almost Daily	80	22.04
2 Almost Every Week	109	30.03
3 Not Regularly	174	47.93
Total	363	100.00

da040: Used the Internet

	Freq.	%
1 Yes	7,869	40.67
2 No	11,480	59.33
Total	19,349	100.00

da041_s1: Devices to Use Internet: Desktop Computer

	Freq.	%
0 No	7,299	92.76
1 Yes	570	7.24
Total	7,869	100.00

da041_s2: Devices to Use Internet: Laptop Computer

	Freq.	%
0 No	7,668	97.45
2 Yes	201	2.55
Total	7,869	100.00

da041_s3: Devices to Use Internet: Tablet Computer

	Freq.	%
0 No	7,642	97.12

	Freq.	%
3 Yes	227	2.88
Total	7,869	100.00

da041_s4: Devices to Use Internet: Cellphone

	Freq.	%
0 No	69	0.88
4 Yes	7,800	99.12
Total	7,869	100.00

da041_s5: Devices to Use Internet: Others, Please Specify

	Freq.	%
0 No	7,837	99.59
5 Yes	32	0.41
Total	7,869	100.00

da042_s1: Activities with Internet: Chatting

	Freq.	%
0 No	3,173	40.32
1 Yes	4,696	59.68
Total	7,869	100.00

da042_s2: Activities with Internet: Reading News

	Freq.	%
0 No	2,392	30.40
2 Yes	5,477	69.60
Total	7,869	100.00

da042_s3: Activities with Internet: Watching Videos

	Freq.	%
0 No	2,010	25.54
3 Yes	5,859	74.46
Total	7,869	100.00

da042_s4: Activities with Internet: Playing Games

	Freq.	%
0 No	6,924	87.99
4 Yes	945	12.01
Total	7,869	100.00

da042_s5: Activities with Internet: Money Management

	Freq.	%
0 No	7,575	96.26

	Freq.	%
5 Yes	294	3.74
Total	7,869	100.00

da042_s6: Activities with Internet: Others, Please Specify

	Freq.	%
0 No	6,845	86.99
6 Yes	1,024	13.01
Total	7,869	100.00

da043: Payment with Mobile Phone

	Freq.	%
1 Yes	4,922	62.55
2 No	2,947	37.45
Total	7,869	100.00

da044: Use Wechat

	Freq.	%
1 Yes	7,174	91.17
2 No	695	8.83
Total	7,869	100.00

da045: Use Wechat Moments

	Freq.	%
1 Yes	4,208	58.66
2 No	2,966	41.34
Total	7,174	100.00

da046: Ever Smoked

	Freq.	%
1 Yes	423	3.62
2 No	11,274	96.38
Total	11,697	100.00

da047: Still Smoke or already Quit

	Freq.	%
1 Still smoke	4,911	60.82
2 Quit	2,565	31.76
3 Never Smoked	599	7.42
Total	8,075	100.00

da048: Products Normally Use

	Freq.	%

1 Smoking a Pipe	414	5.54
2 Smoking Self-Rolled Cigarettes	670	8.96
3 Filtered Cigarette	5,674	75.90
4 Unfiltered Cigarette	453	6.06
5 Cigar	2	0.03
6 Water Cigarettes	263	3.52
Total	7,476	100.00

da049: Time Totally Quit Smoking

	Freq.	%
1 Age	702	27.37
2 Year	1,863	72.63
Total	2,565	100.00

da049_1: Age

Mean	SD	Min	Max	Obs
45.59	19.67	-1.00	88.00	702

da049_2: Year

Mean	SD	Min	Max	Obs
1,967.83	291.88	-1.00	2,020.00	1,863

da050_1: Current Cigarettes in One Day

Mean	SD	Min	Max	Obs
16.57	11.27	0.00	100.00	4,911

da050_2: Cigarettes in One Day Before Quit

Mean	SD	Min	Max	Obs
20.46	16.89	-1.00	100.00	2,565

da051: Frequency Drank Alcoholic Beverages in the Past Year

	Freq.	%
1 Drink More than Once a Month	5,093	26.32
2 Drink But Less than Once a Month	1,835	9.48
3 None of These	12,421	64.19
Total	19,349	100.00

da052: Frequncy Drinking Per Month in the Last Year

	Freq.	%
1 Once a Month	415	8.15
2 Two or Three Times a Month	972	19.09
3 Once a Week	506	9.94

4 Two or Three Times a Week	693	13.61
5 Four to six times a Week	230	4.52
6 Once a Day	1,308	25.68
7 Twice a Day	748	14.69
8 More than Twice a Day	221	4.34
Total	5,093	100.00

db001: Difficulty with Dressing

	Freq.	%
1 Don't Have Any Difficulty	17,791	91.95
2 Have Difficulty But Can Still Do It	959	4.96
3 Have Difficulty and Need Help	428	2.21
4 Can Not Do It	171	0.88
Total	19,349	100.00

db002: Anyone Ever Helped

	Freq.	%
1 Yes	738	47.37
2 No	820	52.63
Total	1,558	100.00

db003: Difficulty with Bathing or Showering

	Freq.	%
1 Don't Have Any Difficulty	17,633	91.13
2 Have Difficulty But Can Still Do It	729	3.77
3 Have Difficulty and Need Help	724	3.74
4 Can Not Do It	263	1.36
Total	19,349	100.00

db004: Anyone Ever Helped

	Freq.	%
1 Yes	1,017	59.27
2 No	699	40.73
Total	1,716	100.00

db005: Difficulty with Eating

	Freq.	%
1 Don't Have Any Difficulty	18,645	96.36
2 Have Difficulty But Can Still Do It	413	2.13
3 Have Difficulty and Need Help	177	0.91
4 Can Not Do It	114	0.59
Total	19,349	100.00

db006: Anyone Ever Helped

	Freq.	%
1 Yes	353	50.14
2 No	351	49.86
Total	704	100.00

db007: Difficulty with Getting into or out of Bed

	Freq.	%
1 Don't Have Any Difficulty	17,816	92.08
2 Have Difficulty But Can Still Do It	1,128	5.83
3 Have Difficulty and Need Help	253	1.31
4 Can Not Do It	152	0.79
Total	19,349	100.00

db008: Anyone Ever Helped

	Freq.	%
1 Yes	492	32.11
2 No	1,040	67.89
Total	1,532	100.00

db009: Difficulty with Using the Toilet

	Freq.	%
1 Don't Have Any Difficulty	16,439	84.96
2 Have Difficulty But Can Still Do It	2,399	12.40
3 Have Difficulty and Need Help	300	1.55
4 Can Not Do It	210	1.09
Total	19,348	100.00

db010: Anyone Ever Helped

	Freq.	%
1 Yes	526	18.08
2 No	2,383	81.92
Total	2,909	100.00

db011: Difficulty with Controlling Urination and Defecation

	Freq.	%
1 Don't Have Any Difficulty	18,381	95.00
2 Have Difficulty But Can Still Do It	627	3.24
3 Have Difficulty and Need Help	193	1.00
4 Can Not Do It	147	0.76
Total	19,348	100.00

db012: Difficulty with Household Chores

	Freq.	%

	Freq.	%
1 Don't Have Any Difficulty	16,545	85.51
2 Have Difficulty But Can Still Do It	1,262	6.52
3 Have Difficulty and Need Help	717	3.71
4 Can Not Do It	824	4.26
Total	19,348	100.00

db013: Anyone Ever Helped

	Freq.	%
1 Yes	1,690	60.29
2 No	1,113	39.71
Total	2,803	100.00

db014: Difficulty with Preparing Hot Meals

	Freq.	%
1 Don't Have Any Difficulty	17,273	89.28
2 Have Difficulty But Can Still Do It	662	3.42
3 Have Difficulty and Need Help	550	2.84
4 Can Not Do It	862	4.46
Total	19,347	100.00

db015: Anyone Ever Helped

	Freq.	%
1 Yes	1,455	70.15
2 No	619	29.85
Total	2,074	100.00

db016: Difficulty with Shopping for Groceries

	Freq.	%
1 Don't Have Any Difficulty	17,698	91.48
2 Have Difficulty But Can Still Do It	394	2.04
3 Have Difficulty and Need Help	468	2.42
4 Can Not Do It	787	4.07
Total	19,347	100.00

db017: Anyone Ever Helped

	Freq.	%
1 Yes	1,308	79.32
2 No	341	20.68
Total	1,649	100.00

db018: Difficulty with Making Phone Calls

	Freq.	%
1 Don't Have Any Difficulty	17,532	90.62
2 Have Difficulty But Can Still Do It	333	1.72

	Freq.	%
3 Have Difficulty and Need Help	502	2.59
4 Can Not Do It	980	5.07
Total	19,347	100.00

db019: Anyone Ever Helped

	Freq.	%
1 Yes	1,206	66.45
2 No	609	33.55
Total	1,815	100.00

db020: Difficulty with Taking Medications

	Freq.	%
1 Don't Have Any Difficulty	18,081	93.46
2 Have Difficulty But Can Still Do It	470	2.43
3 Have Difficulty and Need Help	531	2.74
4 Can Not Do It	265	1.37
Total	19,347	100.00

db021: Anyone Ever Helped

	Freq.	%
1 Yes	814	64.30
2 No	452	35.70
Total	1,266	100.00

db022: Difficulty with Managing Money

	Freq.	%
1 Don't Have Any Difficulty	17,414	90.01
2 Have Difficulty But Can Still Do It	493	2.55
3 Have Difficulty and Need Help	617	3.19
4 Can Not Do It	823	4.25
Total	19,347	100.00

db023: Anyone Ever Helped

	Freq.	%
1 Yes	1,273	65.86
2 No	660	34.14
Total	1,933	100.00

db024_s1: Helper[1]

	Freq.	%
0 No	1,372	39.56
1 Spouse	2,096	60.44
Total	3,468	100.00

db024_s2: Helper[2]

	Freq.	%
0 No	3,440	99.19
2 Father, Mother, Father-in-Law, Mother-in-Law	28	0.81
Total	3,468	100.00

db024_s3: Helper[3]

	Freq.	%
0 No	1,501	43.28
3 Children, Children's Spouse, Grandson, Granddaughter	1,967	56.72
Total	3,468	100.00

db024_s4: Helper[4]

	Freq.	%
0 No	3,334	96.14
4 Sibling, Brother-in-Law, Sister-in-Law, Sibling of Spouse, Etc	134	3.86
Total	3,468	100.00

db024_s5: Helper[5]

	Freq.	%
0 No	3,414	98.44
5 Other Relative	54	1.56
Total	3,468	100.00

db024_s6: Helper[6]

	Freq.	%
0 No	3,418	98.56
6 Paid Helper (Such as Nanny)	50	1.44
Total	3,468	100.00

db024_s7: Helper[7]

	Freq.	%
0 No	3,466	99.94
7 Volunteer or Employee of Facility	2	0.06
Total	3,468	100.00

db024_s8: Helper[8]

	Freq.	%
0 No	3,435	99.05
8 Nursing Home	33	0.95
Total	3,468	100.00

db024_s9: Helper[9]

	Freq.	%
0 No	3,465	99.91
9 Staff from Home-based Nursing Service	3	0.09
Total	3,468	100.00

db024_s10: Helper[10]

	Freq.	%
0 No	3,448	99.42
10 Help from the Community	20	0.58
Total	3,468	100.00

db024_s11: Helper[11]

	Freq.	%
0 No	3,244	93.54
11 Other, Please Specify	224	6.46
Total	3,468	100.00

db024_1: Number of Paid Helpers

Mean	SD	Min	Max	Obs
1.02	0.14	1.00	2.00	50

db025_s1: Who Help the Most

	Freq.	%
0 No	20	71.43
1 Father	8	28.57
Total	28	100.00

db025_s2: Who Help the Most

	Freq.	%
0 No	10	35.71
2 Mother	18	64.29
Total	28	100.00

db025_s3: Who Help the Most

	Freq.	%
0 No	25	89.29
3 Father-in-Law	3	10.71
Total	28	100.00

db025_s4: Who Help the Most

	Freq.	%
0 No	19	67.86
4 Mother-in-Law	9	32.14
Total	28	100.00

db026_s1: Which Child's Family Helped

	Freq.	%
0 No	639	32.49
1 Preload XChildPanAliveName[1]	1,328	67.51
Total	1,967	100.00

db026_s2: Which Child's Family Helped

	Freq.	%
0 No	1,035	52.62
2 Preload XChildPanAliveName[2]	932	47.38
Total	1,967	100.00

db026_s3: Which Child's Family Helped

	Freq.	%
0 No	1,363	69.29
3 Preload XChildPanAliveName[3]	604	30.71
Total	1,967	100.00

db026_s4: Which Child's Family Helped

	Freq.	%
0 No	1,602	81.44
4 Preload XChildPanAliveName[4]	365	18.56
Total	1,967	100.00

db026_s5: Which Child's Family Helped

	Freq.	%
0 No	1,774	90.19
5 Preload XChildPanAliveName[5]	193	9.81
Total	1,967	100.00

db026_s6: Which Child's Family Helped

	Freq.	%
0 No	1,894	96.29
6 Preload XChildPanAliveName[6]	73	3.71
Total	1,967	100.00

db026_s7: Which Child's Family Helped

	Freq.	%
0 No	1,934	98.32
7 Preload XChildPanAliveName[7]	33	1.68
Total	1,967	100.00

db026_s8: Which Child's Family Helped

	Freq.	%
0 No	1,956	99.44
8 Preload XChildPanAliveName[8]	11	0.56
Total	1,967	100.00

db026_s9: Which Child's Family Helped

	Freq.	%
0 No	1,966	99.95
9 Preload XChildPanAliveName[9]	1	0.05
Total	1,967	100.00

db026_s10: Which Child's Family Helped

	Freq.	%
0 No	1,966	99.95
10 Preload XChildPanAliveName[10]	1	0.05
Total	1,967	100.00

db026_s11: Which Child's Family Helped

	Freq.	%
0 No	1,966	99.95
11 Preload XChildPanAliveName[11]	1	0.05
Total	1,967	100.00

db026_s12: Which Child's Family Helped

	Freq.	%
0 No	1,967	100.00
Total	1,967	100.00

db026_s13: Which Child's Family Helped

	Freq.	%
0 No	1,966	99.95
13 Preload XChildPanAliveName[13]	1	0.05
Total	1,967	100.00

db026_s14: Which Child's Family Helped

	Freq.	%
0 No	1,967	100.00
Total	1,967	100.00

db026_s15: Which Child's Family Helped

	Freq.	%
0 No	1,967	100.00
Total	1,967	100.00

db026_s16: Which Child's Family Helped

	Freq.	%
0 No	1,967	100.00
Total	1,967	100.00

db026_s17: Which Child's Family Helped

	Freq.	%
0 No	1,967	100.00
Total	1,967	100.00

db026_s18: Which Child's Family Helped

	Freq.	%
0 No	1,967	100.00
Total	1,967	100.00

db026_s19: Which Child's Family Helped

	Freq.	%
0 No	1,967	100.00
Total	1,967	100.00

db026_s20: Which Child's Family Helped

	Freq.	%
0 No	1,967	100.00
Total	1,967	100.00

db026_s21: Which Child's Family Helped

	Freq.	%
0 No	1,967	100.00
Total	1,967	100.00

db026_s22: Which Child's Family Helped

	Freq.	%
0 No	1,967	100.00
Total	1,967	100.00

db026_s23: Which Child's Family Helped

	Freq.	%
0 No	1,967	100.00
Total	1,967	100.00

db026_s24: Which Child's Family Helped

	Freq.	%
0 No	1,967	100.00
Total	1,967	100.00

db026_s25: Which Child's Family Helped

	Freq.	%
0 No	1,967	100.00
Total	1,967	100.00

db026_s26: Which Child's Family Helped

	Freq.	%
0 No	1,896	96.39
26 Other Child[26]	71	3.61
Total	1,967	100.00

db026_s27: Which Child's Family Helped

	Freq.	%
0 No	1,949	99.08
27 Other Child[27]	18	0.92
Total	1,967	100.00

db026_s28: Which Child's Family Helped

	Freq.	%
0 No	1,962	99.75
28 Other Child[28]	5	0.25
Total	1,967	100.00

db026_s29: Which Child's Family Helped

	Freq.	%
0 No	1,964	99.85

	Freq.	%
29 Other Child[29]	3	0.15
Total	1,967	100.00

db026_s30: Which Child's Family Helped

	Freq.	%
0 No	1,967	100.00
Total	1,967	100.00

db026_s31: Which Child's Family Helped

	Freq.	%
0 No	1,967	100.00
Total	1,967	100.00

db026_s32: Which Child's Family Helped

	Freq.	%
0 No	1,967	100.00
Total	1,967	100.00

db026_s33: Which Child's Family Helped

	Freq.	%
0 No	1,967	100.00
Total	1,967	100.00

db026_s34: Which Child's Family Helped

	Freq.	%
0 No	1,967	100.00
Total	1,967	100.00

db026_s35: Which Child's Family Helped

	Freq.	%
0 No	1,967	100.00
Total	1,967	100.00

db027_1__s1: Who in Child[1]'s Family Helped

	Freq.	%
0 No	181	13.63
1 Himself/herself	1,147	86.37
Total	1,328	100.00

db027_1__s2: Who in Child[1]'s Family Helped

	Freq.	%
0 No	605	45.56
2 His/her Spouse	723	54.44
Total	1,328	100.00

db027_1__s3: Who in Child[1]'s Family Helped

	Freq.	%
0 No	757	57.00
3 His/her Children	571	43.00
Total	1,328	100.00

db027_2__s1: Who in Child[2]'s Family Helped

	Freq.	%
0 No	88	9.44
1 Himself/herself	844	90.56
Total	932	100.00

db027_2__s2: Who in Child[2]'s Family Helped

	Freq.	%
0 No	462	49.57
2 His/her Spouse	470	50.43
Total	932	100.00

db027_2__s3: Who in Child[2]'s Family Helped

	Freq.	%
0 No	586	62.88
3 His/her Children	346	37.12
Total	932	100.00

db027_3__s1: Who in Child[3]'s Family Helped

	Freq.	%
0 No	49	8.11
1 Himself/herself	555	91.89
Total	604	100.00

db027_3__s2: Who in Child[3]'s Family Helped

	Freq.	%
0 No	306	50.66
2 His/her Spouse	298	49.34
Total	604	100.00

db027_3__s3: Who in Child[3]'s Family Helped

	Freq.	%
0 No	389	64.40
3 His/her Children	215	35.60
Total	604	100.00

db027_4__s1: Who in Child[4]'s Family Helped

	Freq.	%
0 No	23	6.30
1 Himself/herself	342	93.70
Total	365	100.00

db027_4__s2: Who in Child[4]'s Family Helped

	Freq.	%
0 No	194	53.15
2 His/her Spouse	171	46.85
Total	365	100.00

db027_4__s3: Who in Child[4]'s Family Helped

	Freq.	%
0 No	252	69.04
3 His/her Children	113	30.96
Total	365	100.00

db027_5__s1: Who in Child[5]'s Family Helped

	Freq.	%
0 No	12	6.22
1 Himself/herself	181	93.78
Total	193	100.00

db027_5__s2: Who in Child[5]'s Family Helped

	Freq.	%
0 No	89	46.11
2 His/her Spouse	104	53.89
Total	193	100.00

db027_5__s3: Who in Child[5]'s Family Helped

	Freq.	%
0 No	131	67.88
3 His/her Children	62	32.12
Total	193	100.00

db027_6__s1: Who in Child[6]'s Family Helped

	Freq.	%
0 No	3	4.11
1 Himself/herself	70	95.89
Total	73	100.00

db027_6__s2: Who in Child[6]'s Family Helped

	Freq.	%
0 No	36	49.32
2 His/her Spouse	37	50.68
Total	73	100.00

db027_6__s3: Who in Child[6]'s Family Helped

	Freq.	%
0 No	49	67.12
3 His/her Children	24	32.88
Total	73	100.00

db027_7__s1: Who in Child[7]'s Family Helped

	Freq.	%
1 Himself/herself	33	100.00
Total	33	100.00

db027_7__s2: Who in Child[7]'s Family Helped

	Freq.	%
0 No	15	45.45
2 His/her Spouse	18	54.55
Total	33	100.00

db027_7__s3: Who in Child[7]'s Family Helped

	Freq.	%
0 No	25	75.76
3 His/her Children	8	24.24
Total	33	100.00

db027_8__s1: Who in Child[8]'s Family Helped

	Freq.	%
1 Himself/herself	11	100.00
Total	11	100.00

db027_8__s2: Who in Child[8]'s Family Helped

	Freq.	%
0 No	7	63.64
2 His/her Spouse	4	36.36
Total	11	100.00

db027_8__s3: Who in Child[8]'s Family Helped

	Freq.	%
0 No	9	81.82
3 His/her Children	2	18.18
Total	11	100.00

db027_9__s1: Who in Child[9]'s Family Helped

	Freq.	%
1 Himself/herself	1	100.00
Total	1	100.00

db027_9__s2: Who in Child[9]'s Family Helped

	Freq.	%
2 His/her Spouse	1	100.00
Total	1	100.00

db027_9__s3: Who in Child[9]'s Family Helped

	Freq.	%
3 His/her Children	1	100.00
Total	1	100.00

db027_10__s1: Who in Child[10]'s Family Helped

	Freq.	%
1 Himself/herself	1	100.00
Total	1	100.00

db027_10__s2: Who in Child[10]'s Family Helped

	Freq.	%
0 No	1	100.00
Total	1	100.00

db027_10__s3: Who in Child[10]'s Family Helped

	Freq.	%
0 No	1	100.00
Total	1	100.00

db027_11__s1: Who in Child[11]'s Family Helped

	Freq.	%
1 Himself/herself	1	100.00
Total	1	100.00

db027_11__s2: Who in Child[11]'s Family Helped

	Freq.	%
0 No	1	100.00
Total	1	100.00

db027_11__s3: Who in Child[11]'s Family Helped

	Freq.	%
0 No	1	100.00
Total	1	100.00

db027_12__s1: Who in Child[12]'s Family Helped

No Observations

db027_12__s2: Who in Child[12]'s Family Helped

No Observations

db027_12__s3: Who in Child[12]'s Family Helped

No Observations

db027_13__s1: Who in Child[13]'s Family Helped

	Freq.	%
1 Himself/herself	1	100.00
Total	1	100.00

db027_13__s2: Who in Child[13]'s Family Helped

	Freq.	%
2 His/her Spouse	1	100.00
Total	1	100.00

db027_13__s3: Who in Child[13]'s Family Helped

	Freq.	%
3 His/her Children	1	100.00
Total	1	100.00

db027_14__s1: Who in Child[14]'s Family Helped

No Observations

db027_14__s2: Who in Child[14]'s Family Helped

No Observations

db027_14__s3: Who in Child[14]'s Family Helped

No Observations

db027_15__s1: Who in Child[15]'s Family Helped

No Observations

db027_15__s2: Who in Child[15]'s Family Helped

No Observations

db027_15__s3: Who in Child[15]'s Family Helped

No Observations

db027_16__s1: Who in Child[16]'s Family Helped

No Observations

db027_16__s2: Who in Child[16]'s Family Helped

No Observations

db027_16__s3: Who in Child[16]'s Family Helped

No Observations

db027_17__s1: Who in Child[17]'s Family Helped

No Observations

db027_17__s2: Who in Child[17]'s Family Helped

No Observations

db027_17__s3: Who in Child[17]'s Family Helped

No Observations

db027_18__s1: Who in Child[18]'s Family Helped

No Observations

db027_18__s2: Who in Child[18]'s Family Helped

No Observations

db027_18__s3: Who in Child[18]'s Family Helped

No Observations

db027_19__s1: Who in Child[19]'s Family Helped

No Observations

db027_19__s2: Who in Child[19]'s Family Helped

No Observations

db027_19__s3: Who in Child[19]'s Family Helped

No Observations

db027_20__s1: Who in Child[20]'s Family Helped

No Observations

db027_20__s2: Who in Child[20]'s Family Helped

No Observations

db027_20__s3: Who in Child[20]'s Family Helped

No Observations

db027_21__s1: Who in Child[21]'s Family Helped

No Observations

db027_21__s2: Who in Child[21]'s Family Helped

No Observations

db027_21__s3: Who in Child[21]'s Family Helped

No Observations

db027_22__s1: Who in Child[22]'s Family Helped

No Observations

db027_22__s2: Who in Child[22]'s Family Helped

No Observations

db027_22__s3: Who in Child[22]'s Family Helped

No Observations

db027_23__s1: Who in Child[23]'s Family Helped

No Observations

db027_23__s2: Who in Child[23]'s Family Helped

No Observations

db027_23__s3: Who in Child[23]'s Family Helped

No Observations

db027_24__s1: Who in Child[24]'s Family Helped

No Observations

db027_24__s2: Who in Child[24]'s Family Helped

No Observations

db027_24__s3: Who in Child[24]'s Family Helped

No Observations

db027_25__s1: Who in Child[25]'s Family Helped

No Observations

db027_25__s2: Who in Child[25]'s Family Helped

No Observations

db027_25__s3: Who in Child[25]'s Family Helped

No Observations

db027_26__s1: Who in Child[26]'s Family Helped

	Freq.	%
0 No	13	18.31
1 Himself/herself	58	81.69
Total	71	100.00

db027_26__s2: Who in Child[26]'s Family Helped

	Freq.	%
0 No	48	67.61
2 His/her Spouse	23	32.39
Total	71	100.00

db027_26__s3: Who in Child[26]'s Family Helped

	Freq.	%
0 No	47	66.20
3 His/her Children	24	33.80
Total	71	100.00

db027_27__s1: Who in Child[27]'s Family Helped

	Freq.	%
1 Himself/herself	18	100.00
Total	18	100.00

db027_27__s2: Who in Child[27]'s Family Helped

	Freq.	%
0 No	11	61.11
2 His/her Spouse	7	38.89
Total	18	100.00

db027_27__s3: Who in Child[27]'s Family Helped

	Freq.	%
0 No	15	83.33
3 His/her Children	3	16.67
Total	18	100.00

db027_28__s1: Who in Child[28]'s Family Helped

	Freq.	%
1 Himself/herself	5	100.00
Total	5	100.00

db027_28__s2: Who in Child[28]'s Family Helped

	Freq.	%
0 No	3	60.00
2 His/her Spouse	2	40.00
Total	5	100.00

db027_28__s3: Who in Child[28]'s Family Helped

	Freq.	%
0 No	3	60.00
3 His/her Children	2	40.00
Total	5	100.00

db027_29__s1: Who in Child[29]'s Family Helped

	Freq.	%
1 Himself/herself	3	100.00
Total	3	100.00

db027_29__s2: Who in Child[29]'s Family Helped

	Freq.	%
0 No	3	100.00
Total	3	100.00

db027_29__s3: Who in Child[29]'s Family Helped

	Freq.	%
0 No	2	66.67
3 His/her Children	1	33.33
Total	3	100.00

db027_30__s1: Who in Child[30]'s Family Helped

No Observations

db027_30__s2: Who in Child[30]'s Family Helped

No Observations

db027_30__s3: Who in Child[30]'s Family Helped

No Observations

db027_31__s1: Who in Child[31]'s Family Helped

No Observations

db027_31__s2: Who in Child[31]'s Family Helped

No Observations

db027_31__s3: Who in Child[31]'s Family Helped

No Observations

db027_32__s1: Who in Child[32]'s Family Helped

No Observations

db027_32__s2: Who in Child[32]'s Family Helped

No Observations

db027_32__s3: Who in Child[32]'s Family Helped

No Observations

db027_33__s1: Who in Child[33]'s Family Helped

No Observations

db027_33__s2: Who in Child[33]'s Family Helped

No Observations

db027_33__s3: Who in Child[33]'s Family Helped

No Observations

db027_34__s1: Who in Child[34]'s Family Helped

No Observations

db027_34__s2: Who in Child[34]'s Family Helped

No Observations

db027_34__s3: Who in Child[34]'s Family Helped

No Observations

db027_35__s1: Who in Child[35]'s Family Helped

No Observations

db027_35__s2: Who in Child[35]'s Family Helped

No Observations

db027_35__s3: Who in Child[35]'s Family Helped

No Observations

db027_1_1_: Number of Children Helped

Mean	SD	Min	Max	Obs
1.63	0.74	-1.00	5.00	572

db027_1_2_: Number of Children Helped

Mean	SD	Min	Max	Obs
1.63	0.68	1.00	4.00	346

db027_1_3_: Number of Children Helped

Mean	SD	Min	Max	Obs
1.66	0.74	1.00	5.00	215

db027_1_4_: Number of Children Helped

Mean	SD	Min	Max	Obs
1.72	0.73	1.00	5.00	113

db027_1_5_: Number of Children Helped

Mean	SD	Min	Max	Obs
1.79	1.04	1.00	6.00	62

db027_1_6_: Number of Children Helped

Mean	SD	Min	Max	Obs
1.67	1.01	1.00	5.00	24

db027_1_7_: Number of Children Helped

Mean	SD	Min	Max	Obs
2.13	1.64	1.00	6.00	8

db027_1_8_: Number of Children Helped

Mean	SD	Min	Max	Obs
2.00	0.00	2.00	2.00	2

db027_1_9_: Number of Children Helped

Mean	SD	Min	Max	Obs
2.00	.	2.00	2.00	1

db027_1_10_: Number of Children Helped

No Observations

db027_1_11_: Number of Children Helped

No Observations

db027_1_12_: Number of Children Helped

No Observations

db027_1_13_: Number of Children Helped

Mean	SD	Min	Max	Obs
1.00	.	1.00	1.00	1

db027_1_14_: Number of Children Helped

No Observations

db027_1_15_: Number of Children Helped

No Observations

db027_1_16_: Number of Children Helped

No Observations

db027_1_17_: Number of Children Helped

No Observations

db027_1_18_: Number of Children Helped

No Observations

db027_1_19_: Number of Children Helped

No Observations

db027_1_20_: Number of Children Helped

No Observations

db027_1_21_: Number of Children Helped

No Observations

db027_1_22_: Number of Children Helped

No Observations

db027_1_23_: Number of Children Helped

No Observations

db027_1_24_: Number of Children Helped

No Observations

db027_1_25_: Number of Children Helped

No Observations

db027_1_26_: Number of Children Helped

Mean	SD	Min	Max	Obs
1.71	0.95	1.00	5.00	24

db027_1_27_: Number of Children Helped

Mean	SD	Min	Max	Obs
1.67	0.58	1.00	2.00	3

db027_1_28_: Number of Children Helped

Mean	SD	Min	Max	Obs
2.00	0.00	2.00	2.00	2

db027_1_29_: Number of Children Helped

Mean	SD	Min	Max	Obs

1.00	.	1.00	1.00	1

db027_1_30_: Number of Children Helped

No Observations

db027_1_31_: Number of Children Helped

No Observations

db027_1_32_: Number of Children Helped

No Observations

db027_1_33_: Number of Children Helped

No Observations

db027_1_34_: Number of Children Helped

No Observations

db027_1_35_: Number of Children Helped

No Observations

db028_s1: Which Sibling's Family Helps

	Freq.	%
0 No	83	61.94
1 Preload XSibName[1]	51	38.06
Total	134	100.00

db028_s2: Which Sibling's Family Helps

	Freq.	%
0 No	82	61.19
2 Preload XSibName[2]	52	38.81
Total	134	100.00

db028_s3: Which Sibling's Family Helps

	Freq.	%
0 No	88	65.67
3 Preload XSibName[3]	46	34.33
Total	134	100.00

db028_s4: Which Sibling's Family Helps

	Freq.	%
0 No	94	70.15
4 Preload XSibName[4]	40	29.85
Total	134	100.00

db028_s5: Which Sibling's Family Helps

	Freq.	%
0 No	105	78.36
5 Preload XSibName[5]	29	21.64
Total	134	100.00

db028_s6: Which Sibling's Family Helps

	Freq.	%
0 No	115	85.82
6 Preload XSibName[6]	19	14.18
Total	134	100.00

db028_s7: Which Sibling's Family Helps

	Freq.	%
0 No	127	94.78
7 Preload XSibName[7]	7	5.22
Total	134	100.00

db028_s8: Which Sibling's Family Helps

	Freq.	%
0 No	133	99.25
8 Preload XSibName[8]	1	0.75
Total	134	100.00

db028_s9: Which Sibling's Family Helps

	Freq.	%
0 No	133	99.25
9 Preload XSibName[9]	1	0.75
Total	134	100.00

db028_s10: Which Sibling's Family Helps

	Freq.	%
0 No	134	100.00
Total	134	100.00

db028_s11: Which Sibling's Family Helps

	Freq.	%
0 No	134	100.00
Total	134	100.00

db028_s12: Which Sibling's Family Helps

	Freq.	%
0 No	134	100.00
Total	134	100.00

db028_s13: Which Sibling's Family Helps

	Freq.	%
0 No	134	100.00
Total	134	100.00

db028_s14: Which Sibling's Family Helps

	Freq.	%
0 No	134	100.00
Total	134	100.00

db028_s15: Which Sibling's Family Helps

	Freq.	%
0 No	134	100.00
Total	134	100.00

db028_s16: Which Sibling's Family Helps

	Freq.	%
0 No	108	80.60
16 Preload XSibName[16]	26	19.40
Total	134	100.00

db028_s17: Which Sibling's Family Helps

	Freq.	%
0 No	102	76.12
17 Preload XSibName[17]	32	23.88
Total	134	100.00

db028_s18: Which Sibling's Family Helps

	Freq.	%
0 No	101	75.37
18 Preload XSibName[18]	33	24.63

	Freq.	%
Total	134	100.00

db028_s19: Which Sibling's Family Helps

	Freq.	%
0 No	113	84.33
19 Preload XSibName[19]	21	15.67
Total	134	100.00

db028_s20: Which Sibling's Family Helps

	Freq.	%
0 No	129	96.27
20 Preload XSibName[20]	5	3.73
Total	134	100.00

db028_s21: Which Sibling's Family Helps

	Freq.	%
0 No	130	97.01
21 Preload XSibName[21]	4	2.99
Total	134	100.00

db028_s22: Which Sibling's Family Helps

	Freq.	%
0 No	131	97.76
22 Preload XSibName[22]	3	2.24
Total	134	100.00

db028_s23: Which Sibling's Family Helps

	Freq.	%
0 No	134	100.00
Total	134	100.00

db028_s24: Which Sibling's Family Helps

	Freq.	%
0 No	134	100.00
Total	134	100.00

db028_s25: Which Sibling's Family Helps

	Freq.	%
0 No	134	100.00
Total	134	100.00

db028_s26: Which Sibling's Family Helps

	Freq.	%
0 No	134	100.00
Total	134	100.00

db028_s27: Which Sibling's Family Helps

	Freq.	%
0 No	134	100.00
Total	134	100.00

db028_s28: Which Sibling's Family Helps

	Freq.	%
0 No	134	100.00
Total	134	100.00

db028_s29: Which Sibling's Family Helps

	Freq.	%
0 No	134	100.00
Total	134	100.00

db028_s30: Which Sibling's Family Helps

	Freq.	%
0 No	134	100.00
Total	134	100.00

db028_s31: Which Sibling's Family Helps

	Freq.	%
0 No	114	85.07
31 Other Sibling[31]	20	14.93
Total	134	100.00

db028_s32: Which Sibling's Family Helps

	Freq.	%
0 No	130	97.01
32 Other Sibling[32]	4	2.99
Total	134	100.00

db028_s33: Which Sibling's Family Helps

	Freq.	%
0 No	133	99.25

	Freq.	%
33 Other Sibling[33]	1	0.75
Total	134	100.00

db028_s34: Which Sibling's Family Helps

	Freq.	%
0 No	133	99.25
34 Other Sibling[34]	1	0.75
Total	134	100.00

db028_s35: Which Sibling's Family Helps

	Freq.	%
0 No	134	100.00
Total	134	100.00

db028_s36: Which Sibling's Family Helps

	Freq.	%
0 No	134	100.00
Total	134	100.00

db028_s37: Which Sibling's Family Helps

	Freq.	%
0 No	134	100.00
Total	134	100.00

db028_s38: Which Sibling's Family Helps

	Freq.	%
0 No	134	100.00
Total	134	100.00

db028_s39: Which Sibling's Family Helps

	Freq.	%
0 No	134	100.00
Total	134	100.00

db028_s40: Which Sibling's Family Helps

	Freq.	%
0 No	134	100.00
Total	134	100.00

db029_1__s1: Who Help in Person from Sibling[1]'s Family

	Freq.	%
0 No	6	11.76
1 Himself/herself	45	88.24
Total	51	100.00

db029_1__s2: Who Help in Person from Sibling[1]'s Family

	Freq.	%
0 No	22	43.14
2 His/her Spouse	29	56.86
Total	51	100.00

db029_1__s3: Who Help in Person from Sibling[1]'s Family

	Freq.	%
0 No	24	47.06
3 His/her Children	27	52.94
Total	51	100.00

db029_2__s1: Who Help in Person from Sibling[2]'s Family

	Freq.	%
0 No	6	11.54
1 Himself/herself	46	88.46
Total	52	100.00

db029_2__s2: Who Help in Person from Sibling[2]'s Family

	Freq.	%
0 No	26	50.00
2 His/her Spouse	26	50.00
Total	52	100.00

db029_2__s3: Who Help in Person from Sibling[2]'s Family

	Freq.	%
0 No	28	53.85
3 His/her Children	24	46.15
Total	52	100.00

db029_3__s1: Who Help in Person from Sibling[3]'s Family

	Freq.	%
0 No	3	6.52
1 Himself/herself	43	93.48
Total	46	100.00

db029_3__s2: Who Help in Person from Sibling[3]'s Family

	Freq.	%
0 No	21	45.65
2 His/her Spouse	25	54.35
Total	46	100.00

db029_3__s3: Who Help in Person from Sibling[3]'s Family

	Freq.	%
0 No	27	58.70
3 His/her Children	19	41.30
Total	46	100.00

db029_4__s1: Who Help in Person from Sibling[4]'s Family

	Freq.	%
0 No	1	2.50
1 Himself/herself	39	97.50
Total	40	100.00

db029_4__s2: Who Help in Person from Sibling[4]'s Family

	Freq.	%
0 No	25	62.50
2 His/her Spouse	15	37.50
Total	40	100.00

db029_4__s3: Who Help in Person from Sibling[4]'s Family

	Freq.	%
0 No	27	67.50
3 His/her Children	13	32.50
Total	40	100.00

db029_5__s1: Who Help in Person from Sibling[5]'s Family

	Freq.	%
0 No	1	3.45
1 Himself/herself	28	96.55
Total	29	100.00

db029_5__s2: Who Help in Person from Sibling[5]'s Family

	Freq.	%
0 No	13	44.83
2 His/her Spouse	16	55.17
Total	29	100.00

db029_5__s3: Who Help in Person from Sibling[5]'s Family

	Freq.	%
0 No	18	62.07
3 His/her Children	11	37.93
Total	29	100.00

db029_6__s1: Who Help in Person from Sibling[6]'s Family

	Freq.	%
1 Himself/herself	19	100.00
Total	19	100.00

db029_6__s2: Who Help in Person from Sibling[6]'s Family

	Freq.	%
0 No	7	36.84
2 His/her Spouse	12	63.16
Total	19	100.00

db029_6__s3: Who Help in Person from Sibling[6]'s Family

	Freq.	%
0 No	10	52.63
3 His/her Children	9	47.37
Total	19	100.00

db029_7__s1: Who Help in Person from Sibling[7]'s Family

	Freq.	%
1 Himself/herself	7	100.00
Total	7	100.00

db029_7__s2: Who Help in Person from Sibling[7]'s Family

	Freq.	%
0 No	3	42.86
2 His/her Spouse	4	57.14
Total	7	100.00

db029_7__s3: Who Help in Person from Sibling[7]'s Family

	Freq.	%
0 No	3	42.86
3 His/her Children	4	57.14
Total	7	100.00

db029_8__s1: Who Help in Person from Sibling[8]'s Family

	Freq.	%
1 Himself/herself	1	100.00
Total	1	100.00

db029_8__s2: Who Help in Person from Sibling[8]'s Family

	Freq.	%
2 His/her Spouse	1	100.00
Total	1	100.00

db029_8__s3: Who Help in Person from Sibling[8]'s Family

	Freq.	%
0 No	1	100.00
Total	1	100.00

db029_9__s1: Who Help in Person from Sibling[9]'s Family

	Freq.	%
1 Himself/herself	1	100.00
Total	1	100.00

db029_9__s2: Who Help in Person from Sibling[9]'s Family

	Freq.	%
2 His/her Spouse	1	100.00
Total	1	100.00

db029_9__s3: Who Help in Person from Sibling[9]'s Family

	Freq.	%
0 No	1	100.00
Total	1	100.00

db029_10__s1: Who Help in Person from Sibling[10]'s Family

No Observations

db029_10__s2: Who Help in Person from Sibling[10]'s Family

No Observations

db029_10__s3: Who Help in Person from Sibling[10]'s Family

No Observations

db029_11__s1: Who Help in Person from Sibling[11]'s Family

No Observations

db029_11__s2: Who Help in Person from Sibling[11]'s Family

No Observations

db029_11__s3: Who Help in Person from Sibling[11]'s Family

No Observations

db029_12__s1: Who Help in Person from Sibling[12]'s Family

No Observations

db029_12__s2: Who Help in Person from Sibling[12]'s Family

No Observations

db029_12__s3: Who Help in Person from Sibling[12]'s Family

No Observations

db029_13__s1: Who Help in Person from Sibling[13]'s Family

No Observations

db029_13__s2: Who Help in Person from Sibling[13]'s Family

No Observations

db029_13__s3: Who Help in Person from Sibling[13]'s Family

No Observations

db029_14__s1: Who Help in Person from Sibling[14]'s Family

No Observations

db029_14__s2: Who Help in Person from Sibling[14]'s Family

No Observations

db029_14__s3: Who Help in Person from Sibling[14]'s Family

No Observations

db029_15__s1: Who Help in Person from Sibling[15]'s Family

No Observations

db029_15__s2: Who Help in Person from Sibling[15]'s Family

No Observations

db029_15__s3: Who Help in Person from Sibling[15]'s Family

No Observations

db029_16__s1: Who Help in Person from Sibling[16]'s Family

	Freq.	%
0 No	3	12.00
1 Himself/herself	22	88.00
Total	25	100.00

db029_16__s2: Who Help in Person from Sibling[16]'s Family

	Freq.	%
0 No	14	56.00
2 His/her Spouse	11	44.00
Total	25	100.00

db029_16__s3: Who Help in Person from Sibling[16]'s Family

	Freq.	%
0 No	12	48.00
3 His/her Children	13	52.00
Total	25	100.00

db029_17__s1: Who Help in Person from Sibling[17]'s Family

	Freq.	%
0 No	7	22.58
1 Himself/herself	24	77.42
Total	31	100.00

db029_17__s2: Who Help in Person from Sibling[17]'s Family

	Freq.	%
0 No	20	64.52
2 His/her Spouse	11	35.48
Total	31	100.00

db029_17__s3: Who Help in Person from Sibling[17]'s Family

	Freq.	%
0 No	15	48.39
3 His/her Children	16	51.61
Total	31	100.00

db029_18__s1: Who Help in Person from Sibling[18]'s Family

	Freq.	%
0 No	1	3.13
1 Himself/herself	31	96.88
Total	32	100.00

db029_18__s2: Who Help in Person from Sibling[18]'s Family

	Freq.	%
0 No	14	43.75
2 His/her Spouse	18	56.25
Total	32	100.00

db029_18__s3: Who Help in Person from Sibling[18]'s Family

	Freq.	%
0 No	16	50.00
3 His/her Children	16	50.00
Total	32	100.00

db029_19__s1: Who Help in Person from Sibling[19]'s Family

	Freq.	%
0 No	1	5.00
1 Himself/herself	19	95.00
Total	20	100.00

db029_19__s2: Who Help in Person from Sibling[19]'s Family

	Freq.	%
0 No	8	40.00
2 His/her Spouse	12	60.00
Total	20	100.00

db029_19__s3: Who Help in Person from Sibling[19]'s Family

	Freq.	%
0 No	11	55.00
3 His/her Children	9	45.00
Total	20	100.00

db029_20__s1: Who Help in Person from Sibling[20]'s Family

	Freq.	%
1 Himself/herself	5	100.00
Total	5	100.00

db029_20__s2: Who Help in Person from Sibling[20]'s Family

	Freq.	%
2 His/her Spouse	5	100.00
Total	5	100.00

db029_20__s3: Who Help in Person from Sibling[20]'s Family

	Freq.	%
0 No	2	40.00
3 His/her Children	3	60.00
Total	5	100.00

db029_21__s1: Who Help in Person from Sibling[21]'s Family

	Freq.	%
0 No	1	25.00
1 Himself/herself	3	75.00
Total	4	100.00

db029_21__s2: Who Help in Person from Sibling[21]'s Family

	Freq.	%
0 No	1	25.00
2 His/her Spouse	3	75.00
Total	4	100.00

db029_21__s3: Who Help in Person from Sibling[21]'s Family

	Freq.	%
0 No	2	50.00
3 His/her Children	2	50.00
Total	4	100.00

db029_22__s1: Who Help in Person from Sibling[22]'s Family

	Freq.	%
1 Himself/herself	3	100.00
Total	3	100.00

db029_22__s2: Who Help in Person from Sibling[22]'s Family

	Freq.	%

	Freq.	%
0 No	1	33.33
2 His/her Spouse	2	66.67
Total	3	100.00

db029_22__s3: Who Help in Person from Sibling[22]'s Family

	Freq.	%
0 No	1	33.33
3 His/her Children	2	66.67
Total	3	100.00

db029_23__s1: Who Help in Person from Sibling[23]'s Family

No Observations

db029_23__s2: Who Help in Person from Sibling[23]'s Family

No Observations

db029_23__s3: Who Help in Person from Sibling[23]'s Family

No Observations

db029_24__s1: Who Help in Person from Sibling[24]'s Family

No Observations

db029_24__s2: Who Help in Person from Sibling[24]'s Family

No Observations

db029_24__s3: Who Help in Person from Sibling[24]'s Family

No Observations

db029_25__s1: Who Help in Person from Sibling[25]'s Family

No Observations

db029_25__s2: Who Help in Person from Sibling[25]'s Family

No Observations

db029_25__s3: Who Help in Person from Sibling[25]'s Family

No Observations

db029_26__s1: Who Help in Person from Sibling[26]'s Family

No Observations

db029_26__s2: Who Help in Person from Sibling[26]'s Family

No Observations

db029_26__s3: Who Help in Person from Sibling[26]'s Family

No Observations

db029_27__s1: Who Help in Person from Sibling[27]'s Family

No Observations

db029_27__s2: Who Help in Person from Sibling[27]'s Family

No Observations

db029_27__s3: Who Help in Person from Sibling[27]'s Family

No Observations

db029_28__s1: Who Help in Person from Sibling[28]'s Family

No Observations

db029_28__s2: Who Help in Person from Sibling[28]'s Family

No Observations

db029_28__s3: Who Help in Person from Sibling[28]'s Family

No Observations

db029_29__s1: Who Help in Person from Sibling[29]'s Family

No Observations

db029_29__s2: Who Help in Person from Sibling[29]'s Family

No Observations

db029_29__s3: Who Help in Person from Sibling[29]'s Family

No Observations

db029_30__s1: Who Help in Person from Sibling[30]'s Family

No Observations

db029_30__s2: Who Help in Person from Sibling[30]'s Family

No Observations

db029_30__s3: Who Help in Person from Sibling[30]'s Family

No Observations

db029_31__s1: Who Help in Person from Sibling[31]'s Family

	Freq.	%
0 No	5	25.00
1 Himself/herself	15	75.00
Total	20	100.00

db029_31__s2: Who Help in Person from Sibling[31]'s Family

	Freq.	%
0 No	13	65.00
2 His/her Spouse	7	35.00
Total	20	100.00

db029_31__s3: Who Help in Person from Sibling[31]'s Family

	Freq.	%
0 No	13	65.00
3 His/her Children	7	35.00
Total	20	100.00

db029_32__s1: Who Help in Person from Sibling[32]'s Family

	Freq.	%
0 No	2	50.00
1 Himself/herself	2	50.00
Total	4	100.00

db029_32__s2: Who Help in Person from Sibling[32]'s Family

	Freq.	%
0 No	3	75.00
2 His/her Spouse	1	25.00
Total	4	100.00

db029_32__s3: Who Help in Person from Sibling[32]'s Family

	Freq.	%
0 No	3	75.00
3 His/her Children	1	25.00
Total	4	100.00

db029_33__s1: Who Help in Person from Sibling[33]'s Family

	Freq.	%
1 Himself/herself	1	100.00
Total	1	100.00

db029_33__s2: Who Help in Person from Sibling[33]'s Family

	Freq.	%
2 His/her Spouse	1	100.00
Total	1	100.00

db029_33__s3: Who Help in Person from Sibling[33]'s Family

	Freq.	%
3 His/her Children	1	100.00
Total	1	100.00

db029_34__s1: Who Help in Person from Sibling[34]'s Family

	Freq.	%
1 Himself/herself	1	100.00
Total	1	100.00

db029_34__s2: Who Help in Person from Sibling[34]'s Family

	Freq.	%
2 His/her Spouse	1	100.00
Total	1	100.00

db029_34__s3: Who Help in Person from Sibling[34]'s Family

	Freq.	%
3 His/her Children	1	100.00
Total	1	100.00

db029_35__s1: Who Help in Person from Sibling[35]'s Family

No Observations

db029_35__s2: Who Help in Person from Sibling[35]'s Family

No Observations

db029_35__s3: Who Help in Person from Sibling[35]'s Family

No Observations

db029_36__s1: Who Help in Person from Sibling[36]'s Family

No Observations

db029_36__s2: Who Help in Person from Sibling[36]'s Family

No Observations

db029_36__s3: Who Help in Person from Sibling[36]'s Family

No Observations

db029_37__s1: Who Help in Person from Sibling[37]'s Family

No Observations

db029_37__s2: Who Help in Person from Sibling[37]'s Family

No Observations

db029_37__s3: Who Help in Person from Sibling[37]'s Family

No Observations

db029_38__s1: Who Help in Person from Sibling[38]'s Family

No Observations

db029_38__s2: Who Help in Person from Sibling[38]'s Family

No Observations

db029_38__s3: Who Help in Person from Sibling[38]'s Family

No Observations

db029_39__s1: Who Help in Person from Sibling[39]'s Family

No Observations

db029_39__s2: Who Help in Person from Sibling[39]'s Family

No Observations

db029_39__s3: Who Help in Person from Sibling[39]'s Family

No Observations

db029_40__s1: Who Help in Person from Sibling[40]'s Family

No Observations

db029_40__s2: Who Help in Person from Sibling[40]'s Family

No Observations

db029_40__s3: Who Help in Person from Sibling[40]'s Family

No Observations

db029_1_1_: Number of Children Helped

Mean	SD	Min	Max	Obs
2.19	2.24	1.00	10.00	27

db029_1_2_: Number of Children Helped

Mean	SD	Min	Max	Obs
2.08	1.32	1.00	7.00	24

db029_1_3_: Number of Children Helped

Mean	SD	Min	Max	Obs
1.79	0.71	1.00	3.00	19

db029_1_4_: Number of Children Helped

Mean	SD	Min	Max	Obs
2.15	1.07	1.00	5.00	13

db029_1_5_: Number of Children Helped

Mean	SD	Min	Max	Obs
2.00	0.77	1.00	3.00	11

db029_1_6_: Number of Children Helped

Mean	SD	Min	Max	Obs
2.00	0.71	1.00	3.00	9

db029_1_7_: Number of Children Helped

Mean	SD	Min	Max	Obs
2.75	0.96	2.00	4.00	4

db029_1_8_: Number of Children Helped

No Observations

db029_1_9_: Number of Children Helped

No Observations

db029_1_10_: Number of Children Helped

No Observations

db029_1_11_: Number of Children Helped

No Observations

db029_1_12_: Number of Children Helped

No Observations

db029_1_13_: Number of Children Helped

No Observations

db029_1_14_: Number of Children Helped

No Observations

db029_1_15_: Number of Children Helped

No Observations

db029_1_16_: Number of Children Helped

Mean	SD	Min	Max	Obs
2.77	1.59	1.00	6.00	13

db029_1_17_: Number of Children Helped

Mean	SD	Min	Max	Obs
1.88	1.63	-1.00	6.00	16

db029_1_18_: Number of Children Helped

Mean	SD	Min	Max	Obs
2.13	0.96	1.00	4.00	16

db029_1_19_: Number of Children Helped

Mean	SD	Min	Max	Obs
2.11	0.78	1.00	3.00	9

db029_1_20_: Number of Children Helped

Mean	SD	Min	Max	Obs
3.00	1.73	2.00	5.00	3

db029_1_21_: Number of Children Helped

Mean	SD	Min	Max	Obs
2.50	2.12	1.00	4.00	2

db029_1_22_: Number of Children Helped

Mean	SD	Min	Max	Obs
3.00	1.41	2.00	4.00	2

db029_1_23_: Number of Children Helped

No Observations

db029_1_24_: Number of Children Helped

No Observations

db029_1_25_: Number of Children Helped

No Observations

db029_1_26_: Number of Children Helped

No Observations

db029_1_27_: Number of Children Helped

No Observations

db029_1_28_: Number of Children Helped

No Observations

db029_1_29_: Number of Children Helped

No Observations

db029_1_30_: Number of Children Helped

No Observations

db029_1_31_: Number of Children Helped

Mean	SD	Min	Max	Obs
2.29	1.89	1.00	5.00	7

db029_1_32_: Number of Children Helped

Mean	SD	Min	Max	Obs
1.00	.	1.00	1.00	1

db029_1_33_: Number of Children Helped

Mean	SD	Min	Max	Obs
2.00	.	2.00	2.00	1

db029_1_34_: Number of Children Helped

Mean	SD	Min	Max	Obs
2.00	.	2.00	2.00	1

db029_1_35_: Number of Children Helped

No Observations

db029_1_36_: Number of Children Helped

No Observations

db029_1_37_: Number of Children Helped

No Observations

db029_1_38_: Number of Children Helped

No Observations

db029_1_39_: Number of Children Helped

No Observations

db029_1_40_: Number of Children Helped

No Observations

db030: Number of Relatives Help in Person

Mean	SD	Min	Max	Obs
2.67	3.51	-1.00	20.00	54

db031: Number of Others Who Help in Person

Mean	SD	Min	Max	Obs
2.32	5.34	-1.00	70.00	224

db032_s1: Choose Most Important 7 Helpers

	Freq.	%
0 No	24	9.72
1 Helper[1]	223	90.28
Total	247	100.00

db032_s2: Choose Most Important 7 Helpers

	Freq.	%
0 No	48	19.43
2 Helper[2]	199	80.57
Total	247	100.00

db032_s3: Choose Most Important 7 Helpers

	Freq.	%
0 No	84	34.01
3 Helper[3]	163	65.99
Total	247	100.00

db032_s4: Choose Most Important 7 Helpers

	Freq.	%
0 No	68	27.53
4 Helper[4]	179	72.47
Total	247	100.00

db032_s5: Choose Most Important 7 Helpers

	Freq.	%
0 No	56	22.67
5 Helper[5]	191	77.33
Total	247	100.00

db032_s6: Choose Most Important 7 Helpers

	Freq.	%
0 No	103	41.70
6 Helper[6]	144	58.30
Total	247	100.00

db032_s7: Choose Most Important 7 Helpers

	Freq.	%
0 No	81	32.79
7 Helper[7]	166	67.21
Total	247	100.00

db032_s8: Choose Most Important 7 Helpers

	Freq.	%
0 No	106	42.91
8 Helper[8]	141	57.09
Total	247	100.00

db032_s9: Choose Most Important 7 Helpers

	Freq.	%
0 No	156	63.16
9 Helper[9]	91	36.84
Total	247	100.00

db032_s10: Choose Most Important 7 Helpers

	Freq.	%
0 No	175	70.85
10 Helper[10]	72	29.15
Total	247	100.00

db032_s11: Choose Most Important 7 Helpers

	Freq.	%
0 No	199	80.57
11 Helper[11]	48	19.43
Total	247	100.00

db032_s12: Choose Most Important 7 Helpers

	Freq.	%
0 No	221	89.47
12 Helper[12]	26	10.53
Total	247	100.00

db032_s13: Choose Most Important 7 Helpers

	Freq.	%
0 No	226	91.50
13 Helper[13]	21	8.50
Total	247	100.00

db032_s14: Choose Most Important 7 Helpers

	Freq.	%
0 No	225	91.09
14 Helper[14]	22	8.91
Total	247	100.00

db032_s15: Choose Most Important 7 Helpers

	Freq.	%
0 No	234	94.74
15 Helper[15]	13	5.26
Total	247	100.00

db032_s16: Choose Most Important 7 Helpers

	Freq.	%
0 No	237	95.95
16 Helper[16]	10	4.05
Total	247	100.00

db032_s17: Choose Most Important 7 Helpers

	Freq.	%
0 No	240	97.17
17 Helper[17]	7	2.83
Total	247	100.00

db032_s18: Choose Most Important 7 Helpers

	Freq.	%
0 No	244	98.79
18 Helper[18]	3	1.21
Total	247	100.00

db032_s19: Choose Most Important 7 Helpers

	Freq.	%
0 No	242	97.98
19 Helper[19]	5	2.02
Total	247	100.00

db032_s20: Choose Most Important 7 Helpers

	Freq.	%
0 No	244	98.79
20 Helper[20]	3	1.21
Total	247	100.00

db032_s21: Choose Most Important 7 Helpers

	Freq.	%
0 No	247	100.00
Total	247	100.00

db032_s22: Choose Most Important 7 Helpers

	Freq.	%
0 No	246	99.60
22 Helper[22]	1	0.40
Total	247	100.00

db032_s23: Choose Most Important 7 Helpers

	Freq.	%
0 No	247	100.00
Total	247	100.00

db032_s24: Choose Most Important 7 Helpers

	Freq.	%
0 No	247	100.00
Total	247	100.00

db032_s25: Choose Most Important 7 Helpers

	Freq.	%

0 No	246	99.60
25 Helper[25]	1	0.40
Total	247	100.00

db033_1_: Days Helper[1] Helped

Mean	SD	Min	Max	Obs
18.78	13.10	-1.00	31.00	3,443

db033_2_: Days Helper[2] Helped

Mean	SD	Min	Max	Obs
11.67	12.48	-1.00	31.00	1,647

db033_3_: Days Helper[3] Helped

Mean	SD	Min	Max	Obs
8.71	11.36	-1.00	31.00	1,080

db033_4_: Days Helper[4] Helped

Mean	SD	Min	Max	Obs
7.55	10.27	-1.00	31.00	731

db033_5_: Days Helper[5] Helped

Mean	SD	Min	Max	Obs
6.51	9.43	-1.00	31.00	498

db033_6_: Days Helper[6] Helped

Mean	SD	Min	Max	Obs
5.66	8.81	-1.00	31.00	324

db033_7_: Days Helper[7] Helped

Mean	SD	Min	Max	Obs
5.55	8.73	-1.00	31.00	233

db033_8_: Days Helper[8] Helped

Mean	SD	Min	Max	Obs
5.66	8.96	-1.00	31.00	141

db033_9_: Days Helper[9] Helped

Mean	SD	Min	Max	Obs
5.87	9.03	-1.00	30.00	91

db033_10_: Days Helper[10] Helped

Mean	SD	Min	Max	Obs
4.86	8.01	-1.00	30.00	72

db033_11_: Days Helper[11] Helped

Mean	SD	Min	Max	Obs
5.42	8.63	-1.00	31.00	48

db033_12_: Days Helper[12] Helped

Mean	SD	Min	Max	Obs
7.73	10.75	0.00	30.00	26

db033_13_: Days Helper[13] Helped

Mean	SD	Min	Max	Obs
9.29	11.85	-1.00	30.00	21

db033_14_: Days Helper[14] Helped

Mean	SD	Min	Max	Obs
6.95	9.05	-1.00	30.00	22

db033_15_: Days Helper[15] Helped

Mean	SD	Min	Max	Obs
10.08	10.41	0.00	30.00	13

db033_16_: Days Helper[16] Helped

Mean	SD	Min	Max	Obs
11.80	13.34	0.00	30.00	10

db033_17_: Days Helper[17] Helped

Mean	SD	Min	Max	Obs
5.00	11.18	0.00	30.00	7

db033_18_: Days Helper[18] Helped

Mean	SD	Min	Max	Obs
12.00	15.72	1.00	30.00	3

db033_19_: Days Helper[19] Helped

Mean	SD	Min	Max	Obs
1.40	1.34	0.00	3.00	5

db033_20_: Days Helper[20] Helped

Mean	SD	Min	Max	Obs
2.67	1.53	1.00	4.00	3

db033_21_: Days Helper[21] Helped

No Observations

db033_22_: Days Helper[22] Helped

Mean	SD	Min	Max	Obs
3.00	.	3.00	3.00	1

db033_23_: Days Helper[23] Helped

No Observations

db033_24_: Days Helper[24] Helped

No Observations

db033_25_: Days Helper[25] Helped

Mean	SD	Min	Max	Obs
3.00	.	3.00	3.00	1

db034_1_: Hours Helper[1] Helped

Mean	SD	Min	Max	Obs
3.32	5.61	-1.00	24.00	3,443

db034_2_: Hours Helper[2] Helped

Mean	SD	Min	Max	Obs
3.02	5.12	-1.00	24.00	1,647

db034_3_: Hours Helper[3] Helped

Mean	SD	Min	Max	Obs
2.69	4.65	-1.00	24.00	1,080

db034_4_: Hours Helper[4] Helped

Mean	SD	Min	Max	Obs
3.01	5.08	-1.00	24.00	731

db034_5_: Hours Helper[5] Helped

Mean	SD	Min	Max	Obs
3.01	5.05	-1.00	24.00	498

db034_6_: Hours Helper[6] Helped

Mean	SD	Min	Max	Obs
3.24	5.52	-1.00	24.00	324

db034_7_: Hours Helper[7] Helped

Mean	SD	Min	Max	Obs
3.47	5.56	-1.00	24.00	233

db034_8_: Hours Helper[8] Helped

Mean	SD	Min	Max	Obs
2.89	5.20	-1.00	24.00	141

db034_9_: Hours Helper[9] Helped

Mean	SD	Min	Max	Obs
2.57	4.38	-1.00	24.00	91

db034_10_: Hours Helper[10] Helped

Mean	SD	Min	Max	Obs
3.17	5.64	-1.00	24.00	72

db034_11_: Hours Helper[11] Helped

Mean	SD	Min	Max	Obs
3.02	4.88	-1.00	24.00	48

db034_12_: Hours Helper[12] Helped

Mean	SD	Min	Max	Obs
3.19	3.26	0.00	15.00	26

db034_13_: Hours Helper[13] Helped

Mean	SD	Min	Max	Obs
3.38	5.29	-1.00	24.00	21

db034_14_: Hours Helper[14] Helped

Mean	SD	Min	Max	Obs
2.77	5.06	-1.00	24.00	22

db034_15_: Hours Helper[15] Helped

Mean	SD	Min	Max	Obs
2.00	2.31	-1.00	6.00	13

db034_16_: Hours Helper[16] Helped

Mean	SD	Min	Max	Obs
2.10	3.00	-1.00	8.00	10

db034_17_: Hours Helper[17] Helped

Mean	SD	Min	Max	Obs
4.71	8.75	-1.00	24.00	7

db034_18_: Hours Helper[18] Helped

Mean	SD	Min	Max	Obs
4.67	2.08	3.00	7.00	3

db034_19_: Hours Helper[19] Helped

Mean	SD	Min	Max	Obs
3.40	3.13	0.00	8.00	5

db034_20_: Hours Helper[20] Helped

Mean	SD	Min	Max	Obs
2.67	1.15	2.00	4.00	3

db034_21_: Hours Helper[21] Helped

No Observations

db034_22_: Hours Helper[22] Helped

Mean	SD	Min	Max	Obs
2.00	.	2.00	2.00	1

db034_23_: Hours Helper[23] Helped

No Observations

db034_24_: Hours Helper[24] Helped

No Observations

db034_25_: Hours Helper[25] Helped

Mean	SD	Min	Max	Obs
2.00	.	2.00	2.00	1

db035_1_: Helper[1] Living in Your Home

	Freq.	%
1 Yes	2,915	84.66
2 No	528	15.34
Total	3,443	100.00

db035_2_: Helper[2] Living in Your Home

	Freq.	%
1 Yes	1,054	64.00
2 No	593	36.00
Total	1,647	100.00

db035_3_: Helper[3] Living in Your Home

	Freq.	%
1 Yes	644	59.63
2 No	436	40.37
Total	1,080	100.00

db035_4_: Helper[4] Living in Your Home

	Freq.	%
1 Yes	379	51.85
2 No	352	48.15
Total	731	100.00

db035_5_: Helper[5] Living in Your Home

	Freq.	%
1 Yes	225	45.18
2 No	273	54.82
Total	498	100.00

db035_6_: Helper[6] Living in Your Home

	Freq.	%
1 Yes	134	41.36
2 No	190	58.64
Total	324	100.00

db035_7_: Helper[7] Living in Your Home

	Freq.	%
1 Yes	107	45.92
2 No	126	54.08
Total	233	100.00

db035_8_: Helper[8] Living in Your Home

	Freq.	%
1 Yes	48	34.04
2 No	93	65.96
Total	141	100.00

db035_9_: Helper[9] Living in Your Home

	Freq.	%
1 Yes	36	39.56
2 No	55	60.44
Total	91	100.00

db035_10_: Helper[10] Living in Your Home

	Freq.	%
1 Yes	26	36.11
2 No	46	63.89
Total	72	100.00

db035_11_: Helper[11] Living in Your Home

	Freq.	%
1 Yes	19	39.58
2 No	29	60.42
Total	48	100.00

db035_12_: Helper[12] Living in Your Home

	Freq.	%
1 Yes	7	26.92
2 No	19	73.08
Total	26	100.00

db035_13_: Helper[13] Living in Your Home

	Freq.	%
1 Yes	7	33.33
2 No	14	66.67
Total	21	100.00

db035_14_: Helper[14] Living in Your Home

	Freq.	%
1 Yes	10	45.45
2 No	12	54.55
Total	22	100.00

db035_15_: Helper[15] Living in Your Home

	Freq.	%
1 Yes	7	53.85
2 No	6	46.15
Total	13	100.00

db035_16_: Helper[16] Living in Your Home

	Freq.	%
1 Yes	5	50.00
2 No	5	50.00
Total	10	100.00

db035_17_: Helper[17] Living in Your Home

	Freq.	%
1 Yes	2	28.57
2 No	5	71.43
Total	7	100.00

db035_18_: Helper[18] Living in Your Home

	Freq.	%
1 Yes	1	33.33
2 No	2	66.67
Total	3	100.00

db035_19_: Helper[19] Living in Your Home

	Freq.	%
1 Yes	1	20.00
2 No	4	80.00
Total	5	100.00

db035_20_: Helper[20] Living in Your Home

	Freq.	%
1 Yes	1	33.33
2 No	2	66.67
Total	3	100.00

db035_21_: Helper[21] Living in Your Home

No Observations

db035_22_: Helper[22] Living in Your Home

	Freq.	%
2 No	1	100.00
Total	1	100.00

db035_23_: Helper[23] Living in Your Home

No Observations

db035_24_: Helper[24] Living in Your Home

No Observations

db035_25_: Helper[25] Living in Your Home

	Freq.	%
2 No	1	100.00
Total	1	100.00

db048: Change of Received Care Because of Pademic

	Freq.	%
1 Become More	265	7.64
2 Become Less	216	6.23
3 Don't Change	2,986	86.13
Total	3,467	100.00

db036: Any Possible Helper in the Future

	Freq.	%
1 Yes	10,809	68.07
2 No	5,070	31.93
Total	15,879	100.00

db037_s1: Helper[1]'s Relationship to You

	Freq.	%
0 No	4,820	44.59
1 Spouse	5,989	55.41
Total	10,809	100.00

db037_s2: Helper[2]'s Relationship to You

	Freq.	%
0 No	10,753	99.48
2 Father, Mother, Father-in-Law, Mother-in-Law	56	0.52
Total	10,809	100.00

db037_s3: Helper[3]'s Relationship to You

	Freq.	%
0 No	1,320	12.21
3 Children, Children's Spouse, Grandson, Granddaughter	9,489	87.79
Total	10,809	100.00

db037_s4: Helper[4]'s Relationship to You

	Freq.	%
0 No	10,171	94.10
4 Sibling, Brother-in-Law, Sister-in-Law, Sibling of Spouse, Etc	638	5.90
Total	10,809	100.00

db037_s5: Helper[5]'s Relationship to You

	Freq.	%
0 No	10,693	98.93
5 Other Relative	116	1.07
Total	10,809	100.00

db037_s6: Helper[6]'s Relationship to You

	Freq.	%
0 No	10,598	98.05
6 Paid Helper (Such as Nanny)	211	1.95
Total	10,809	100.00

db037_s7: Helper[7]'s Relationship to You

	Freq.	%
0 No	10,732	99.29
7 Volunteer or Employee of Facility	77	0.71
Total	10,809	100.00

db037_s8: Helper[8]'s Relationship to You

	Freq.	%
0 No	10,486	97.01
8 Nursing Home	323	2.99
Total	10,809	100.00

db037_s9: Helper[9]'s Relationship to You

	Freq.	%
0 No	10,692	98.92
9 Staff from Home-based Nursing Service	117	1.08
Total	10,809	100.00

db037_s10: Helper[10]'s Relationship to You

	Freq.	%
0 No	10,674	98.75
10 Help from the Community	135	1.25
Total	10,809	100.00

db037_s11: Helper[11]'s Relationship to You

	Freq.	%
0 No	10,745	99.41
11 Other, Please Specify	64	0.59
Total	10,809	100.00

db037_1: Number of Paid Helpers

Mean	SD	Min	Max	Obs
0.90	0.55	-1.00	3.00	211

db038_s1: Who Will Help You in Future

	Freq.	%
0 No	23	41.07
1 Father	33	58.93
Total	56	100.00

db038_s2: Who Will Help You in Future

	Freq.	%
0 No	9	16.07
2 Mother	47	83.93
Total	56	100.00

db038_s3: Who Will Help You in Future

	Freq.	%
0 No	38	67.86
3 Father-in-Law	18	32.14
Total	56	100.00

db038_s4: Who Will Help You in Future

	Freq.	%
0 No	30	53.57
4 Mother-in-Law	26	46.43
Total	56	100.00

db039_s1: Which Child's Family Will Help You in Future

	Freq.	%
0 No	998	10.52
1 Preload XChildName[1]	8,491	89.48
Total	9,489	100.00

db039_s2: Which Child's Family Will Help You in Future

	Freq.	%
0 No	2,951	31.10
2 Preload XChildName[2]	6,538	68.90
Total	9,489	100.00

db039_s3: Which Child's Family Will Help You in Future

	Freq.	%
0 No	6,459	68.07
3 Preload XChildName[3]	3,030	31.93
Total	9,489	100.00

db039_s4: Which Child's Family Will Help You in Future

	Freq.	%
0 No	8,183	86.24
4 Preload XChildName[4]	1,306	13.76
Total	9,489	100.00

db039_s5: Which Child's Family Will Help You in Future

	Freq.	%
0 No	9,005	94.90
5 Preload XChildName[5]	484	5.10
Total	9,489	100.00

db039_s6: Which Child's Family Will Help You in Future

	Freq.	%
0 No	9,327	98.29
6 Preload XChildName[6]	162	1.71
Total	9,489	100.00

db039_s7: Which Child's Family Will Help You in Future

	Freq.	%
0 No	9,430	99.38
7 Preload XChildName[7]	59	0.62
Total	9,489	100.00

db039_s8: Which Child's Family Will Help You in Future

	Freq.	%
0 No	9,465	99.75
8 Preload XChildName[8]	24	0.25
Total	9,489	100.00

db039_s9: Which Child's Family Will Help You in Future

	Freq.	%
0 No	9,480	99.91
9 Preload XChildName[9]	9	0.09
Total	9,489	100.00

db039_s10: Which Child's Family Will Help You in Future

	Freq.	%
0 No	9,485	99.96
10 Preload XChildName[10]	4	0.04
Total	9,489	100.00

db039_s11: Which Child's Family Will Help You in Future

	Freq.	%
0 No	9,487	99.98
11 Preload XChildName[11]	2	0.02
Total	9,489	100.00

db039_s12: Which Child's Family Will Help You in Future

	Freq.	%
0 No	9,488	99.99
12 Preload XChildName[12]	1	0.01
Total	9,489	100.00

db039_s13: Which Child's Family Will Help You in Future

	Freq.	%
0 No	9,489	100.00
Total	9,489	100.00

db039_s14: Which Child's Family Will Help You in Future

	Freq.	%
0 No	9,489	100.00
Total	9,489	100.00

db039_s15: Which Child's Family Will Help You in Future

	Freq.	%
0 No	9,489	100.00
Total	9,489	100.00

db039_s16: Which Child's Family Will Help You in Future

	Freq.	%
0 No	9,489	100.00
Total	9,489	100.00

db039_s17: Which Child's Family Will Help You in Future

	Freq.	%
0 No	9,489	100.00
Total	9,489	100.00

db039_s18: Which Child's Family Will Help You in Future

	Freq.	%
0 No	9,489	100.00
Total	9,489	100.00

db039_s19: Which Child's Family Will Help You in Future

	Freq.	%
0 No	9,489	100.00
Total	9,489	100.00

db039_s20: Which Child's Family Will Help You in Future

	Freq.	%
0 No	9,489	100.00
Total	9,489	100.00

db039_s21: Which Child's Family Will Help You in Future

	Freq.	%
0 No	9,489	100.00
Total	9,489	100.00

db039_s22: Which Child's Family Will Help You in Future

	Freq.	%
0 No	9,489	100.00
Total	9,489	100.00

db039_s23: Which Child's Family Will Help You in Future

	Freq.	%
0 No	9,489	100.00
Total	9,489	100.00

db039_s24: Which Child's Family Will Help You in Future

	Freq.	%
0 No	9,489	100.00
Total	9,489	100.00

db039_s25: Which Child's Family Will Help You in Future

	Freq.	%
0 No	9,489	100.00
Total	9,489	100.00

db039_s26: Which Child's Family Will Help You in Future

	Freq.	%
0 No	9,177	96.71
26 Other Child[26]	312	3.29
Total	9,489	100.00

db039_s27: Which Child's Family Will Help You in Future

	Freq.	%
0 No	9,350	98.54
27 Other Child[27]	139	1.46

Total	9,489	100.00

db039_s28: Which Child's Family Will Help You in Future

	Freq.	%
0 No	9,432	99.40
28 Other Child[28]	57	0.60
Total	9,489	100.00

db039_s29: Which Child's Family Will Help You in Future

	Freq.	%
0 No	9,462	99.72
29 Other Child[29]	27	0.28
Total	9,489	100.00

db039_s30: Which Child's Family Will Help You in Future

	Freq.	%
0 No	9,479	99.89
30 Other Child[30]	10	0.11
Total	9,489	100.00

db039_s31: Which Child's Family Will Help You in Future

	Freq.	%
0 No	9,484	99.95
31 Other Child[31]	5	0.05
Total	9,489	100.00

db039_s32: Which Child's Family Will Help You in Future

	Freq.	%
0 No	9,486	99.97
32 Other Child[32]	3	0.03
Total	9,489	100.00

db039_s33: Which Child's Family Will Help You in Future

	Freq.	%
0 No	9,486	99.97
33 Other Child[33]	3	0.03
Total	9,489	100.00

db039_s34: Which Child's Family Will Help You in Future

	Freq.	%
0 No	9,489	100.00
Total	9,489	100.00

db039_s35: Which Child's Family Will Help You in Future

	Freq.	%
0 No	9,487	99.98
35 Other Child[35]	2	0.02
Total	9,489	100.00

db040_s1: Which Sibling's Family Will Help You

	Freq.	%
0 No	240	37.68
1 Preload XSibName[1]	397	62.32
Total	637	100.00

db040_s2: Which Sibling's Family Will Help You

	Freq.	%
0 No	255	40.03
2 Preload XSibName[2]	382	59.97
Total	637	100.00

db040_s3: Which Sibling's Family Will Help You

	Freq.	%
0 No	282	44.27
3 Preload XSibName[3]	355	55.73
Total	637	100.00

db040_s4: Which Sibling's Family Will Help You

	Freq.	%
0 No	381	59.81
4 Preload XSibName[4]	256	40.19
Total	637	100.00

db040_s5: Which Sibling's Family Will Help You

	Freq.	%
0 No	469	73.63
5 Preload XSibName[5]	168	26.37
Total	637	100.00

db040_s6: Which Sibling's Family Will Help You

	Freq.	%
0 No	558	87.60
6 Preload XSibName[6]	79	12.40
Total	637	100.00

db040_s7: Which Sibling's Family Will Help You

	Freq.	%
0 No	608	95.45
7 Preload XSibName[7]	29	4.55
Total	637	100.00

db040_s8: Which Sibling's Family Will Help You

	Freq.	%
0 No	621	97.49
8 Preload XSibName[8]	16	2.51
Total	637	100.00

db040_s9: Which Sibling's Family Will Help You

	Freq.	%
0 No	631	99.06
9 Preload XSibName[9]	6	0.94
Total	637	100.00

db040_s10: Which Sibling's Family Will Help You

	Freq.	%
0 No	635	99.69
10 Preload XSibName[10]	2	0.31
Total	637	100.00

db040_s11: Which Sibling's Family Will Help You

	Freq.	%
0 No	637	100.00
Total	637	100.00

db040_s12: Which Sibling's Family Will Help You

	Freq.	%
0 No	637	100.00
Total	637	100.00

db040_s13: Which Sibling's Family Will Help You

	Freq.	%
0 No	637	100.00
Total	637	100.00

db040_s14: Which Sibling's Family Will Help You

	Freq.	%
0 No	637	100.00
Total	637	100.00

db040_s15: Which Sibling's Family Will Help You

	Freq.	%
0 No	637	100.00
Total	637	100.00

db040_s16: Which Sibling's Family Will Help You

	Freq.	%
0 No	418	65.62
16 Preload XSibName[16]	219	34.38
Total	637	100.00

db040_s17: Which Sibling's Family Will Help You

	Freq.	%
0 No	412	64.68
17 Preload XSibName[17]	225	35.32
Total	637	100.00

db040_s18: Which Sibling's Family Will Help You

	Freq.	%
0 No	437	68.60
18 Preload XSibName[18]	200	31.40
Total	637	100.00

db040_s19: Which Sibling's Family Will Help You

	Freq.	%
0 No	496	77.86
19 Preload XSibName[19]	141	22.14
Total	637	100.00

db040_s20: Which Sibling's Family Will Help You

	Freq.	%
0 No	548	86.03
20 Preload XSibName[20]	89	13.97
Total	637	100.00

db040_s21: Which Sibling's Family Will Help You

	Freq.	%
0 No	603	94.66
21 Preload XSibName[21]	34	5.34
Total	637	100.00

db040_s22: Which Sibling's Family Will Help You

	Freq.	%
0 No	621	97.49
22 Preload XSibName[22]	16	2.51
Total	637	100.00

db040_s23: Which Sibling's Family Will Help You

	Freq.	%
0 No	631	99.06
23 Preload XSibName[23]	6	0.94
Total	637	100.00

db040_s24: Which Sibling's Family Will Help You

	Freq.	%
0 No	636	99.84
24 Preload XSibName[24]	1	0.16
Total	637	100.00

db040_s25: Which Sibling's Family Will Help You

	Freq.	%
0 No	635	99.69
25 Preload XSibName[25]	2	0.31
Total	637	100.00

db040_s26: Which Sibling's Family Will Help You

	Freq.	%
0 No	637	100.00
Total	637	100.00

db040_s27: Which Sibling's Family Will Help You

	Freq.	%
0 No	637	100.00
Total	637	100.00

db040_s28: Which Sibling's Family Will Help You

	Freq.	%
0 No	637	100.00
Total	637	100.00

db040_s29: Which Sibling's Family Will Help You

	Freq.	%
0 No	637	100.00
Total	637	100.00

db040_s30: Which Sibling's Family Will Help You

	Freq.	%
0 No	637	100.00
Total	637	100.00

db040_s31: Which Sibling's Family Will Help You

	Freq.	%
0 No	590	92.62
31 Other Sibling[31]	47	7.38
Total	637	100.00

db040_s32: Which Sibling's Family Will Help You

	Freq.	%
0 No	622	97.65
32 Other Sibling[32]	15	2.35
Total	637	100.00

db040_s33: Which Sibling's Family Will Help You

	Freq.	%
0 No	635	99.69
33 Other Sibling[33]	2	0.31
Total	637	100.00

db040_s34: Which Sibling's Family Will Help You

	Freq.	%
0 No	637	100.00
Total	637	100.00

db040_s35: Which Sibling's Family Will Help You

	Freq.	%
0 No	637	100.00

Total	637	100.00

db040_s36: Which Sibling's Family Will Help You

	Freq.	%
0 No	637	100.00
Total	637	100.00

db040_s37: Which Sibling's Family Will Help You

	Freq.	%
0 No	637	100.00
Total	637	100.00

db040_s38: Which Sibling's Family Will Help You

	Freq.	%
0 No	637	100.00
Total	637	100.00

db040_s39: Which Sibling's Family Will Help You

	Freq.	%
0 No	637	100.00
Total	637	100.00

db040_s40: Which Sibling's Family Will Help You

	Freq.	%
0 No	637	100.00
Total	637	100.00

db041: Number of Relatives Help in Person

Mean	SD	Min	Max	Obs
4.05	6.56	-1.00	50.00	116

db042: Number of Others Who Help in Person

Mean	SD	Min	Max	Obs
3.70	10.39	-1.00	80.00	64

db043: Can't Work Because of Disability or Health

	Freq.	%
1 I Can't Work at All	2,419	12.50
2 I Can't Work for Long Time	4,692	24.25

	Freq.	%
3 I Have No Problem Working	12,234	63.24
Total	19,345	100.00

db044: Can't Do Household Work Because of Disability or Health

	Freq.	%
1 I Can't Do at All	189	1.12
2 I Can't Do for Long Time	3,484	20.58
3 I Have No Problem	13,253	78.30
Total	16,926	100.00

db045: How often the Respondent Receive Assistance

	Freq.	%
1 Never	15,476	80.00
2 A Few Times	2,368	12.24
3 Most or All of the Time	317	1.64
4 The Section Was Completed by a Proxy Respondent	1,184	6.12
Total	19,345	100.00

db046: Relationship to R

	Freq.	%
1 Spouse	626	52.87
2 Mother	4	0.34
3 Father	2	0.17
4 Mother-in-Law	6	0.51
5 Father-in-Law	5	0.42
6 Sibling	25	2.11
7 Brother-in-Law, sister-in-Law	7	0.59
8 Child	282	23.82
9 Spouse of Child	98	8.28
10 Grandchild	48	4.05
11 Other Relative	27	2.28
12 Helper or Other Non-Relative	54	4.56
Total	1,184	100.00

db047: Main Reason for Proxy

	Freq.	%
1 Respondent Has Serious Physical Handicaps	473	39.95
2 Respondent Has Serious Mental Handicaps	122	10.30
3 Respondent Has Rejected this Interview	315	26.60
4 Others, Please Specify	274	23.14
Total	1,184	100.00

dc001: Checking Year

	Freq.	%
1 Correct	11,811	67.21
2 Error	2,845	16.19
997 Don't Know	2,897	16.49

	Freq.	%
999 Refused	20	0.11
Total	17,573	100.00

dc002: Checking Season

	Freq.	%
1 Correct	13,353	75.99
2 Error	2,046	11.64
997 Don't Know	2,155	12.26
999 Refused	19	0.11
Total	17,573	100.00

dc003: Checking Date

	Freq.	%
1 Correct	11,531	65.62
2 Error	2,875	16.36
997 Don't Know	3,150	17.93
999 Refused	17	0.10
Total	17,573	100.00

dc004: Checking Day

	Freq.	%
1 Correct	10,698	60.88
2 Error	2,705	15.39
997 Don't Know	4,155	23.65
999 Refused	14	0.08
Total	17,572	100.00

dc005: Checking Month

	Freq.	%
1 Correct	15,825	90.06
2 Error	973	5.54
997 Don't Know	762	4.34
999 Refused	12	0.07
Total	17,572	100.00

dc006: Self-Rated Memory

	Freq.	%
1 Excellent	92	0.52
2 Very Good	738	4.20
3 Good	1,220	6.94
4 Fair	9,898	56.33
5 Poor	5,568	31.69
997 Don't Know	56	0.32
Total	17,572	100.00

dc007_1: Result for 100-7

	Freq.	%
1 Record Answer	14,149	80.52
997 Don't Know	2,546	14.49
999 Refused	876	4.99
Total	17,571	100.00

dc007_1_1: Specific Result from 100-7

Mean	SD	Min	Max	Obs
663,423.95	78908514.63	0.00	9.39e+09	14,151

dc007_2: Result for dc007_1_1-7

	Freq.	%
1 Record Answer	12,930	91.38
997 Don't Know	1,063	7.51
999 Refused	156	1.10
Total	14,149	100.00

dc007_2_1: Specific Result from dc007_1_1-7

Mean	SD	Min	Max	Obs
726,056.50	82550208.84	-1.00	9.39e+09	12,930

dc007_3: Result for dc007_2_1-7

	Freq.	%
1 Record Answer	12,374	95.70
997 Don't Know	458	3.54
999 Refused	98	0.76
Total	12,930	100.00

dc007_3_1: Specific Result from dc007_2_1-7

Mean	SD	Min	Max	Obs
77.46	11.11	0.00	979.00	12,374

dc007_4: Result for dc007_4_1-7

	Freq.	%
1 Record Answer	12,027	97.20
997 Don't Know	285	2.30
999 Refused	62	0.50
Total	12,374	100.00

dc007_4_1: Specific Result from dc007_3_1-7

Mean	SD	Min	Max	Obs
69.86	11.88	-1.00	972.00	12,027

dc007_5: Result for dc007_4_1-7

	Freq.	%
1 Record Answer	11,706	97.33
997 Don't Know	282	2.34
999 Refused	39	0.32
Total	12,027	100.00

dc007_5_1: Specific Result from dc007_4_1-7

Mean	SD	Min	Max	Obs
62.95	12.63	-1.00	965.00	11,707

dc008: Use Pen Paper or Other Instruments for Mathmatics

	Freq.	%
1 Used	1,615	11.41
2 Not Used	12,534	88.59
Total	14,149	100.00

dc009: Draw

	Freq.	%
1 Correct	5,371	30.57
2 Error	10,105	57.51
3 Not Assessed	610	3.47
997 Don't Know	592	3.37
999 Refused	892	5.08
Total	17,570	100.00

dc010_1: Understand WR Test

	Freq.	%
1 Yes	17,005	96.78
2 No	565	3.22
Total	17,570	100.00

dc010_2: Understand WR Test

	Freq.	%
1 Yes	70	12.39
2 No	495	87.61
Total	565	100.00

dc010_3: Understand WR Test

	Freq.	%
1 Yes	60	12.12
2 No	435	87.88
Total	495	100.00

dc011_s1: Reasons for Rufused Word Recall Test

	Freq.	%
0 No	206	37.66
1 Refused	341	62.34
Total	547	100.00

dc011_s2: Reasons for Rufused Word Recall Test

	Freq.	%
0 No	547	100.00
Total	547	100.00

dc011_s3: Reasons for Rufused Word Recall Test

	Freq.	%
0 No	538	98.35
3 Dumb at Old Age	9	1.65
Total	547	100.00

dc011_s4: Reasons for Rufused Word Recall Test

	Freq.	%
0 No	446	81.54
4 Deaf	101	18.46
Total	547	100.00

dc011_s5: Reasons for Rufused Word Recall Test

	Freq.	%
0 No	349	63.80
5 Others, Please Sepficy	198	36.20
Total	547	100.00

dc012_s1: Wordlist[9]

	Freq.	%
0 No	7,463	42.48
1 Yes	10,107	57.52
Total	17,570	100.00

dc012_s2: Wordlist[3]

	Freq.	%
0 No	12,754	72.59
2 Yes	4,816	27.41
Total	17,570	100.00

dc012_s3: Wordlist[1]

	Freq.	%
0 No	13,906	79.15
3 Yes	3,664	20.85
Total	17,570	100.00

dc012_s4: Wordlist[2]

	Freq.	%
0 No	16,153	91.94
4 Yes	1,417	8.06
Total	17,570	100.00

dc012_s5: Wordlist[10]

	Freq.	%
0 No	13,672	77.81
5 Yes	3,898	22.19
Total	17,570	100.00

dc012_s6: Wordlist[4]

	Freq.	%
0 No	12,744	72.53
6 Yes	4,826	27.47
Total	17,570	100.00

dc012_s7: Wordlist[5]

	Freq.	%
0 No	9,867	56.16
7 Yes	7,703	43.84
Total	17,570	100.00

dc012_s8: Wordlist[6]

	Freq.	%
0 No	14,812	84.30
8 Yes	2,758	15.70
Total	17,570	100.00

dc012_s9: Wordlist[8]

	Freq.	%
0 No	7,724	43.96
9 Yes	9,846	56.04
Total	17,570	100.00

dc012_s10: Wordlist[7]

	Freq.	%
0 No	7,629	43.42
10 Yes	9,941	56.58
Total	17,570	100.00

dc012_s11: None of the Above

	Freq.	%
0 No	16,326	92.92
11 Yes	1,244	7.08
Total	17,570	100.00

dc012_s12: Refused to Recall

	Freq.	%
0 No	17,143	97.57
12 Yes	427	2.43
Total	17,570	100.00

dc012_s13: Can't Understand or Incaple of the Test

	Freq.	%
0 No	17,023	96.89
13 Yes	547	3.11
Total	17,570	100.00

dc013_s1: Wordlist[5]

	Freq.	%
0 No	6,093	36.71
1 Yes	10,503	63.29
Total	16,596	100.00

dc013_s2: Wordlist[2]

	Freq.	%
0 No	12,600	75.92
2 Yes	3,996	24.08
Total	16,596	100.00

dc013_s3: Wordlist[9]

	Freq.	%
0 No	6,196	37.33
3 Yes	10,400	62.67
Total	16,596	100.00

dc013_s4: Wordlist[10]

	Freq.	%
0 No	9,402	56.65
4 Yes	7,194	43.35
Total	16,596	100.00

dc013_s5: Wordlist[3]

	Freq.	%
0 No	9,042	54.48
5 Yes	7,554	45.52
Total	16,596	100.00

dc013_s6: Wordlist[1]

	Freq.	%
0 No	9,680	58.33
6 Yes	6,916	41.67
Total	16,596	100.00

dc013_s7: Wordlist[8]

	Freq.	%
0 No	6,045	36.42
7 Yes	10,551	63.58
Total	16,596	100.00

dc013_s8: Wordlist[4]

	Freq.	%
0 No	6,258	37.71
8 Yes	10,338	62.29
Total	16,596	100.00

dc013_s9: Wordlist[6]

	Freq.	%
0 No	9,163	55.21
9 Yes	7,433	44.79
Total	16,596	100.00

dc013_s10: Wordlist[7]

	Freq.	%
0 No	5,028	30.30
10 Yes	11,568	69.70
Total	16,596	100.00

dc013_s11: None of the Above

	Freq.	%
0 No	15,985	96.32
11 Yes	611	3.68
Total	16,596	100.00

dc013_s12: Refused to Recall

	Freq.	%
0 No	16,330	98.40
12 Yes	266	1.60
Total	16,596	100.00

dc014_s1: Wordlist[1]

	Freq.	%
0 No	7,158	43.83
1 Yes	9,172	56.17
Total	16,330	100.00

dc014_s2: Wordlist[2]

	Freq.	%
0 No	10,072	61.68
2 Yes	6,258	38.32
Total	16,330	100.00

dc014_s3: Wordlist[3]

	Freq.	%
0 No	7,662	46.92
3 Yes	8,668	53.08
Total	16,330	100.00

dc014_s4: Wordlist[4]

	Freq.	%
0 No	7,111	43.55
4 Yes	9,219	56.45
Total	16,330	100.00

dc014_s5: Wordlist[5]

	Freq.	%
0 No	5,178	31.71
5 Yes	11,152	68.29
Total	16,330	100.00

dc014_s6: Wordlist[6]

	Freq.	%
0 No	9,862	60.39
6 Yes	6,468	39.61
Total	16,330	100.00

dc014_s7: Wordlist[7]

	Freq.	%
0 No	6,681	40.91
7 Yes	9,649	59.09
Total	16,330	100.00

dc014_s8: Wordlist[8]

	Freq.	%
0 No	4,381	26.83
8 Yes	11,949	73.17
Total	16,330	100.00

dc014_s9: Wordlist[9]

	Freq.	%
0 No	4,494	27.52
9 Yes	11,836	72.48
Total	16,330	100.00

dc014_s10: Wordlist[10]

	Freq.	%
0 No	4,749	29.08
10 Yes	11,581	70.92
Total	16,330	100.00

dc014_s11: None of the Above

	Freq.	%
0 No	15,741	96.39
11 Yes	589	3.61
Total	16,330	100.00

dc014_s12: Refused to Recall

	Freq.	%
0 No	16,096	98.57
12 Yes	234	1.43
Total	16,330	100.00

dc015_s1: Whether any of the Following Apply

	Freq.	%
0 No	16,297	95.74
1 Interruption Occured during Administration	726	4.26
Total	17,023	100.00

dc015_s2: Whether any of the Following Apply

	Freq.	%
0 No	15,621	91.76
2 Others, Please Specify	1,402	8.24
Total	17,023	100.00

dc015_s3: Whether any of the Following Apply

	Freq.	%
0 No	2,063	12.12
3 None of the above	14,960	87.88
Total	17,023	100.00

dc016: Bothered by Things

	Freq.	%
1 Rarely or None (<1 Day)	8,686	49.44
2 Some or A Little (1-2 Days)	2,746	15.63
3 Occasionally or Moderate Amount of Time (3-4 Days)	3,534	20.12
4 Most of the Time (5-7 Days)	2,321	13.21
997 Don't Know	242	1.38
999 Refused	39	0.22
Total	17,568	100.00

dc017: Had Trouble Keeping Mind

	Freq.	%
1 Rarely or None (<1 Day)	8,574	48.80
2 Some or A Little (1-2 Days)	2,653	15.10
3 Occasionally or Moderate Amount of Time (3-4 Days)	3,531	20.10
4 Most of the Time (5-7 Days)	2,336	13.30
997 Don't Know	426	2.42
999 Refused	48	0.27
Total	17,568	100.00

dc018: Felt Depressed

	Freq.	%
1 Rarely or None (<1 Day)	9,059	51.57
2 Some or A Little (1-2 Days)	2,656	15.12
3 Occasionally or Moderate Amount of Time (3-4 Days)	3,435	19.55
4 Most of the Time (5-7 Days)	1,964	11.18
997 Don't Know	415	2.36
999 Refused	39	0.22
Total	17,568	100.00

dc019: I Felt Everything I Did Was An Effort

	Freq.	%
1 Rarely or None (<1 Day)	9,283	52.84
2 Some or A Little (1-2 Days)	2,242	12.76
3 Occasionally or Moderate Amount of Time (3-4 Days)	3,001	17.08
4 Most of the Time (5-7 Days)	2,738	15.59
997 Don't Know	258	1.47
999 Refused	46	0.26
Total	17,568	100.00

dc020: I Felt Hopeful about the Future

	Freq.	%
1 Rarely or None (<1 Day)	5,188	29.53
2 Some or A Little (1-2 Days)	1,870	10.64
3 Occasionally or Moderate Amount of Time (3-4 Days)	3,375	19.21
4 Most of the Time (5-7 Days)	6,364	36.22
997 Don't Know	708	4.03
999 Refused	63	0.36
Total	17,568	100.00

dc021: I Felt Fearful

	Freq.	%
1 Rarely or None (<1 Day)	13,636	77.62
2 Some or A Little (1-2 Days)	1,273	7.25
3 Occasionally or Moderate Amount of Time (3-4 Days)	1,599	9.10
4 Most of the Time (5-7 Days)	940	5.35
997 Don't Know	91	0.52
999 Refused	28	0.16
Total	17,567	100.00

dc022: My Sleep Was Restless

	Freq.	%
1 Rarely or None (<1 Day)	8,425	47.96
2 Some or A Little (1-2 Days)	2,285	13.01
3 Occasionally or Moderate Amount of Time (3-4 Days)	3,347	19.05
4 Most of the Time (5-7 Days)	3,412	19.42
997 Don't Know	71	0.40
999 Refused	26	0.15

	Freq.	%
Total	17,566	100.00

dc023: I Was Happy

	Freq.	%
1 Rarely or None (<1 Day)	3,643	20.74
2 Some or A Little (1-2 Days)	1,745	9.93
3 Occasionally or Moderate Amount of Time (3-4 Days)	3,993	22.73
4 Most of the Time (5-7 Days)	7,961	45.32
997 Don't Know	183	1.04
999 Refused	41	0.23
Total	17,566	100.00

dc024: I Felt Lonely

	Freq.	%
1 Rarely or None (<1 Day)	12,251	69.74
2 Some or A Little (1-2 Days)	1,500	8.54
3 Occasionally or Moderate Amount of Time (3-4 Days)	2,076	11.82
4 Most of the Time (5-7 Days)	1,482	8.44
997 Don't Know	216	1.23
999 Refused	41	0.23
Total	17,566	100.00

dc025: I Could Not Get on

	Freq.	%
1 Rarely or None (<1 Day)	13,335	75.91
2 Some or A Little (1-2 Days)	1,158	6.59
3 Occasionally or Moderate Amount of Time (3-4 Days)	1,579	8.99
4 Most of the Time (5-7 Days)	1,140	6.49
997 Don't Know	298	1.70
999 Refused	56	0.32
Total	17,566	100.00

dc026: Life Satisfication

	Freq.	%
1 Completely Satisfied	860	4.90
2 Very Satisfied	5,394	30.71
3 Somewhat Satistied	9,357	53.27
4 Not Very Satistied	1,451	8.26
5 Not at All Satisfied	504	2.87
Total	17,566	100.00

dc027: Chidren Satisfication

	Freq.	%
1 Completely Satisfied	1,229	7.12
2 Very Satisfied	8,044	46.58
3 Somewhat Satistied	7,099	41.11
4 Not Very Satistied	664	3.85

	Freq.	%
5 Not at All Satisfied	212	1.23
6 No Child	21	0.12
Total	17,269	100.00

dc028_s1: Wordlist[1]

	Freq.	%
0 No	8,830	53.21
1 Yes	7,764	46.79
Total	16,594	100.00

dc028_s2: Wordlist[2]

	Freq.	%
0 No	10,648	64.17
2 Yes	5,946	35.83
Total	16,594	100.00

dc028_s3: Wordlist[3]

	Freq.	%
0 No	8,056	48.55
3 Yes	8,538	51.45
Total	16,594	100.00

dc028_s4: Wordlist[4]

	Freq.	%
0 No	9,341	56.29
4 Yes	7,253	43.71
Total	16,594	100.00

dc028_s5: Wordlist[5]

	Freq.	%
0 No	6,074	36.60
5 Yes	10,520	63.40
Total	16,594	100.00

dc028_s6: Wordlist[6]

	Freq.	%
0 No	11,639	70.14
6 Yes	4,955	29.86
Total	16,594	100.00

dc028_s7: Wordlist[7]

	Freq.	%

	Freq.	%
0 No	7,861	47.37
7 Yes	8,733	52.63
Total	16,594	100.00

dc028_s8: Wordlist[8]

	Freq.	%
0 No	7,392	44.55
8 Yes	9,202	55.45
Total	16,594	100.00

dc028_s9: Wordlist[9]

	Freq.	%
0 No	6,373	38.41
9 Yes	10,221	61.59
Total	16,594	100.00

dc028_s10: Wordlist[10]

	Freq.	%
0 No	8,336	50.24
10 Yes	8,258	49.76
Total	16,594	100.00

dc028_s11: None of the Above

	Freq.	%
0 No	14,880	89.67
11 Yes	1,714	10.33
Total	16,594	100.00

dc028_s12: Refused to Recall

	Freq.	%
0 No	16,312	98.30
12 Yes	282	1.70
Total	16,594	100.00

dc029_s1: Things Happened during Respondent Interview

	Freq.	%
0 No	17,200	97.92
1 R Had Poor Eysight	366	2.08
Total	17,566	100.00

dc029_s2: Things Happened during Respondent Interview

	Freq.	%

	Freq.	%
0 No	16,819	95.75
2 R Had Poor Hearing and Didn't Wear Hearing Aid	747	4.25
Total	17,566	100.00

dc029_s3: Things Happened during Respondent Interview

	Freq.	%
0 No	17,521	99.74
3 R Wore Hearing Aid	45	0.26
Total	17,566	100.00

dc029_s4: Things Happened during Respondent Interview

	Freq.	%
0 No	17,420	99.17
4 R Had Tremor Hand, Which Interfered Some Tests	146	0.83
Total	17,566	100.00

dc029_s5: Things Happened during Respondent Interview

	Freq.	%
0 No	16,516	94.02
5 Interview Was Interfered by Other Affairs or Noisy	1,050	5.98
Total	17,566	100.00

dc029_s6: Things Happened during Respondent Interview

	Freq.	%
0 No	17,087	97.27
6 Quality Not Good for Emotion Problems of R	479	2.73
Total	17,566	100.00

dc029_s7: Things Happened during Respondent Interview

	Freq.	%
0 No	16,844	95.89
7 Others, Please Specify	722	4.11
Total	17,566	100.00

dc029_s8: Things Happened during Respondent Interview

	Freq.	%
0 No	2,993	17.04
8 None of the Above	14,573	82.96
Total	17,566	100.00

dc030: Laguage Used by Interviweres

	Freq.	%

	Freq.	%
1 Mandarin	9,343	53.19
2 Local Dialect	7,285	41.47
3 Other Dialect, Please Sepcify	938	5.34
Total	17,566	100.00

xrage: Age of Respondent

Mean	SD	Min	Max	Obs
61.56	10.07	18.00	108.00	19,367

xrtype: Respondent Type

	Freq.	%
1 RE Interview	19,237	99.33
2 New Interview	130	0.67
Total	19,367	100.00

xchrodistype_1_: Name of Disease [1]

A String Variable	
Obs:	19,366

xchrodistype_2_: Name of Disease [2]

A String Variable	
Obs:	19,366

xchrodistype_3_: Name of Disease [3]

A String Variable	
Obs:	19,366

xchrodistype_4_: Name of Disease [4]

A String Variable	
Obs:	19,366

xchrodistype_5_: Name of Disease [5]

A String Variable	
Obs:	19,366

xchrodistype_6_: Name of Disease [6]

A String Variable	
Obs:	19,366

xchrodistype_7_: Name of Disease [7]

A String Variable	
Obs:	19,366

xchrodistype_8_: Name of Disease [8]

A String Variable	
Obs:	19,366

xchrodistype_9_: Name of Disease [9]

A String Variable	
Obs:	19,366

xchrodistype_10_: Name of Disease [10]

A String Variable	
Obs:	19,366

xchrodistype_11_: Name of Disease [11]

A String Variable	
Obs:	19,366

xchrodistype_12_: Name of Disease [12]

A String Variable	
Obs:	19,366

xchrodistype_13_: Name of Disease [13]

A String Variable	
Obs:	19,366

xchrodistype_14_: Name of Disease [14]

A String Variable	
Obs:	19,366

xchrodistype_15_: Name of Disease [15]

A String Variable	
Obs:	19,366

xfagepossibility: Specific Life Expectancy

Mean	SD	Min	Max	Obs
78.50	5.67	75.00	115.00	17,662

xpsyacttype_1_: Intensive Physical Activity

A String Variable	
Obs:	19,355

xpsyacttype_2_: Moderate Physical Activity

A String Variable	
Obs:	19,355

xpsyacttype_3_: Light Physical Activity

A String Variable	
Obs:	19,355

xsoctype_1_: Interacted With Friends

A String Variable	
Obs:	6,186

xsoctype_2_: Played Ma-jong, Played Chess, Played Cards, or Went to Community Club

A String Variable	
Obs:	2,834

xsoctype_3_: Provided Help to Family, Friends, or Neighbors Who Do Not Live With You

A String Variable	
Obs:	2,989

xsoctype_4_: Went to a Sport, Social, or Other Kind of Club

A String Variable	
Obs:	1,328

xsoctype_5_: Took Part in a Community-Related Organization

A String Variable	
Obs:	460

xsoctype_6_: Done Voluntary or Charity work, or Cared for a Sick or Disabled Adult Who Does N

A String Variable	
Obs:	608

xsoctype_7_: Attended an Educational or Training Course

A String Variable	
Obs:	307

xsoctype_8_: Others

A String Variable	
Obs:	363

xhelperselect: Have Helper or Not

	Freq.	%
0 No	15,879	82.07
1 Yes	3,468	17.93
Total	19,347	100.00

xhelpernum: Number of Helpers

Mean	SD	Min	Max	Obs
2.81	3.34	1.00	39.00	3,467

xselectnum: Numbers Selected

Mean	SD	Min	Max	Obs
7.00	0.00	7.00	7.00	247

xhelpercurrent: Have Helper in Current

	Freq.	%
0 No	15,879	82.08
1 Yes	3,467	17.92
Total	19,346	100.00

xwordlist_1_: Wordlist[1]

A String Variable	
Obs:	17,570

xwordlist_2_: Wordlist[2]

A String Variable	
Obs:	17,570

xwordlist_3_: Wordlist[3]

A String Variable	
Obs:	17,570

xwordlist_4_: Wordlist[4]

A String Variable	
Obs:	17,570

xwordlist_5_: Wordlist[5]

A String Variable	
Obs:	17,570

xwordlist_6_: Wordlist[6]

A String Variable	
Obs:	17,570

xwordlist_7_: Wordlist[7]

A String Variable	
Obs:	17,570

xwordlist_8_: Wordlist[8]

A String Variable	
Obs:	17,570

xwordlist_9_: Wordlist[9]

A String Variable	
Obs:	17,570

xwordlist_10_: Wordlist[10]

A String Variable	
Obs:	17,570

xwordrecallbr: Had Immediate WR test

	Freq.	%
0 No	972	5.53
1 Yes	16,594	94.47
Total	17,566	100.00

ziwtime: R's LAST IW Time

A String Variable	
Obs:	19,237

zsmoke: Smoking in LAST IW Time

	Freq.	%
1 Yes	7,657	100.00
Total	7,657	100.00

zchildnum: Number of Children in LAST IW Time

Mean	SD	Min	Max	Obs
2.63	1.40	1.00	17.00	18,977

zdisease_1_: Had Doctor DIagnosed Disease[1] Listed in DA007 at ZIWTime

	Freq.	%
1 Yes	6,563	100.00
Total	6,563	100.00

zdisease_2_: Had Doctor DIagnosed Disease[2] Listed in DA007 at ZIWTime

	Freq.	%
1 Yes	3,726	100.00
Total	3,726	100.00

zdisease_3_: Had Doctor DIagnosed Disease[3] Listed in DA007 at ZIWTime

	Freq.	%
1 Yes	2,165	100.00
Total	2,165	100.00

zdisease_4_: Had Doctor DIagnosed Disease[4] Listed in DA007 at ZIWTime

	Freq.	%
1 Yes	337	100.00
Total	337	100.00

zdisease_5_: Had Doctor DIagnosed Disease[5] Listed in DA007 at ZIWTime

	Freq.	%
1 Yes	2,252	100.00
Total	2,252	100.00

zdisease_6_: Had Doctor DIagnosed Disease[6] Listed in DA007 at ZIWTime

	Freq.	%
1 Yes	1,137	100.00
Total	1,137	100.00

zdisease_7_: Had Doctor DIagnosed Disease[7] Listed in DA007 at ZIWTime

	Freq.	%
1 Yes	3,268	100.00
Total	3,268	100.00

zdisease_8_: Had Doctor DIagnosed Disease[8] Listed in DA007 at ZIWTime

	Freq.	%
1 Yes	1,189	100.00
Total	1,189	100.00

zdisease_9_: Had Doctor DIagnosed Disease[9] Listed in DA007 at ZIWTime

	Freq.	%
1 Yes	1,588	100.00
Total	1,588	100.00

zdisease_10_: Had Doctor DIagnosed Disease[10] Listed in DA007 at ZIWTime

	Freq.	%
1 Yes	5,303	100.00
Total	5,303	100.00

zdisease_11_: Had Doctor DIagnosed Disease[11] Listed in DA007 at ZIWTime

	Freq.	%
1 Yes	384	100.00
Total	384	100.00

zdisease_12_: Had Doctor DIagnosed Disease[12] Listed in DA007 at ZIWTime

	Freq.	%
1 Yes	17	100.00
Total	17	100.00

zdisease_13_: Had Doctor DIagnosed Disease[13] Listed in DA007 at ZIWTime

	Freq.	%
1 Yes	344	100.00
Total	344	100.00

zdisease_14_: Had Doctor DIagnosed Disease[14] Listed in DA007 at ZIWTime

	Freq.	%
1 Yes	6,530	100.00
Total	6,530	100.00

zdisease_15_: Had Doctor DIagnosed Disease[15] Listed in DA007 at ZIWTime

	Freq.	%
1 Yes	887	100.00
Total	887	100.00

zselfdisease_1_: Had only Self-known Disease[1] Listed in DA007 at ZIWTime

	Freq.	%
1 Yes	148	100.00
Total	148	100.00

zselfdisease_5_: Had only Self-known Disease[5] Listed in DA007 at ZIWTime

	Freq.	%
1 Yes	225	100.00
Total	225	100.00

zselfdisease_11_: Had only Self-known Disease[11] Listed in DA007 at ZIWTime

	Freq.	%
1 Yes	148	100.00
Total	148	100.00

versionID: Version ID

A String Variable	
Obs:	19,367

MODULE F

Work and Retirement

ID: Individual ID

A String Variable	
Obs:	19,361

householdID: Household ID

A String Variable	
Obs:	19,361

communityID: Community ID

A String Variable	
Obs:	19,361

fa001: Has Farm Work

	Freq.	%
1 Yes	9,062	46.81
2 No	10,297	53.19
Total	19,359	100.00

fa002_s1: Farm Work Self-employed

	Freq.	%
0 No	211	2.33
1 Yes	8,851	97.67
Total	9,062	100.00

fa002_s2: Farm Employed

	Freq.	%
0 No	8,287	91.45
2 Yes	775	8.55
Total	9,062	100.00

fa004: Has Nonfarm Work

	Freq.	%
1 Yes	5,512	28.47
2 No	13,846	71.53
Total	19,358	100.00

fa005: On Vacation

	Freq.	%
1 Yes	304	2.20
2 No	13,542	97.80
Total	13,846	100.00

fa007: Expect to Return to the Job or Not

	Freq.	%
1 Yes	223	73.36
2 No	81	26.64
Total	304	100.00

fa008: Still Get Paid on the Job or Not

	Freq.	%
1 Yes	10	12.35
2 No	71	87.65
Total	81	100.00

fa009: Has More than One Nonfarm Job

	Freq.	%
1 Yes	281	5.12
2 No	5,207	94.88
Total	5,488	100.00

fa010: Main Job Employed or Self Employed

	Freq.	%
1 Employed Nonfarm	375	69.70
2 Self-employed Nonfarm	86	15.99
3 Unpaid Family Business Nonfarm	15	2.79
4 Farm Employed	62	11.52
Total	538	100.00

fa011: Job Employed or Self-Employed

	Freq.	%
1 Employed Nonfarm	3,831	73.57
2 Self-employed Nonfarm	1,000	19.20
3 Unpaid Family Business Nonfarm	376	7.22
Total	5,207	100.00

fa013: Since When Started to Work After the LastIW

	Freq.	%
1 Yes	607	39.99
995 Deny	876	57.71
999 Refuse to Answer	35	2.31
Total	1,518	100.00

fa013_1: Year (When Started to Work since the LastIW)

Mean	SD	Min	Max	Obs
2,019.08	0.96	2,014.00	2,020.00	607

fa013_2: Month (When Started to Work since the LastIW)

Mean	SD	Min	Max	Obs
5.72	2.97	1.00	12.00	551

fa014: Double Check on FA013

	Freq.	%
1 Yes	871	99.43
997 Do not Know	5	0.57
Total	876	100.00

fa015: Reason to Start to Work

	Freq.	%
More Income	365	60.13
Better Health	36	5.93
Family Issues	38	6.26
As Workout	51	8.40
No Specific Reason	40	6.59
Others	77	12.69
Total	607	100.00

fa016: Since When Stopped Working After the LastIW

	Freq.	%
1 Yes	1,277	73.82
995 Deny	415	23.99
999 Refuse to Answer	38	2.20

	Freq.	%
Total	1,730	100.00

fa016_1: Year (Since When Stopped Working)

Mean	SD	Min	Max	Obs
2,018.78	1.12	2,011.00	2,020.00	1,278

fa016_2: Month (Since When Stopped Working)

Mean	SD	Min	Max	Obs
5.98	3.50	1.00	12.00	1,158

fa017: Double Check on FA016

	Freq.	%
1 Yes	412	99.28
997 Do not Know	3	0.72
Total	415	100.00

fa018_1: Year (Since When Stopped Working if not Worked in the LastIW)

Mean	SD	Min	Max	Obs
2,011.47	7.24	1,980.00	2,020.00	415

fa018_2: Month (Since When Stopped Working if not Worked in the LastIW)

Mean	SD	Min	Max	Obs
5.68	3.54	1.00	12.00	226

fa019: Reason to Stop Working

	Freq.	%
External Reason	80	4.73
Inadequate Income	60	3.55
Inconvenience	26	1.54
Health Issues	789	46.63
Family Issues	360	21.28
Retirement	136	8.04
Temporary Suspension	17	1.00
Others	224	13.24
Total	1,692	100.00

fb001: Location of farm work

	Freq.	%
1 Same as the Residence	8,097	91.48
2 Another Neighborhood but Same County as the Residence	379	4.28
3 Other Domestic Location	367	4.15
4 Abroad	3	0.03

999 Refuse to Answer	5	0.06
Total	8,851	100.00

fb002_s1: Industry: Plantation

	Freq.	%
0 No	426	4.81
1 Yes	8,424	95.19
Total	8,850	100.00

fb002_s2: Industry: Forestry

	Freq.	%
0 No	8,180	92.43
2 Yes	670	7.57
Total	8,850	100.00

fb002_s3: Industry: Husbandry

	Freq.	%
0 No	5,170	58.42
3 Yes	3,680	41.58
Total	8,850	100.00

fb002_s4: Industry: Fishing

	Freq.	%
0 No	8,559	96.71
4 Yes	291	3.29
Total	8,850	100.00

fb002_s5: Industry: Farming Technology

	Freq.	%
0 No	8,806	99.50
5 Yes	44	0.50
Total	8,850	100.00

fb002_s997: Do not Know

	Freq.	%
0 No	8,848	99.98
997 Yes	2	0.02
Total	8,850	100.00

fb003: Type (Occupation) of the Household Farm Work

	Freq.	%

	Freq.	%
1 Manual Labor	8,405	94.97
2 Management	163	1.84
3 Machine/Vehicle Operator	166	1.88
4 Sales	51	0.58
5 Other	65	0.73
Total	8,850	100.00

fb005: Months for Household Farm Work

Mean	SD	Min	Max	Obs
7.20	4.29	1.00	12.00	8,850

fb006: Days for Household Farm Work

Mean	SD	Min	Max	Obs
4.63	2.21	1.00	7.00	8,850

fb007: Hours for Household Farm Work

Mean	SD	Min	Max	Obs
5.39	3.00	1.00	24.00	8,850

fc001: Receive Wage from the Workplace or Dispatch Company

	Freq.	%
1 Workplace	3,648	76.24
2 Dispatch Company	155	3.24
3 Individual	982	20.52
Total	4,785	100.00

fc002: Type of the Employer

	Freq.	%
1 Government	424	8.86
2 Institutions	365	7.63
3 NGO	27	0.56
4 Firm	1,338	27.96
5 Individual Firm	1,754	36.66
6 Farmer	545	11.39
7 Individual Household	127	2.65
8 Other	99	2.07
997 Do not Know	106	2.22
Total	4,785	100.00

fc003: Same Employer as in the LastIW

	Freq.	%
1 Yes	1,712	62.69
2 No	972	35.59
995 Deny	47	1.72

	Freq.	%
Total	2,731	100.00

fc004: Location of the Workplace

	Freq.	%
1 Same as the Residence	2,657	55.53
2 Another Neighborhood but Same County as the Residence	1,518	31.72
3 Other Domestic Location	531	11.10
4 Abroad	56	1.17
999 Refuse to Answer	23	0.48
Total	4,785	100.00

fc006: Civil Servant or Not

	Freq.	%
1 Yes	50	11.79
2 No	374	88.21
Total	424	100.00

fc008: Formal Employee or Not

	Freq.	%
1 Yes	198	54.25
2 No	167	45.75
Total	365	100.00

fc012: Supervise Anyone or Not

	Freq.	%
1 Yes	581	12.14
2 No	4,204	87.86
Total	4,785	100.00

fc016: Has Any Written Labor Contract with the Current Employer or Not

	Freq.	%
1 Yes	1,167	24.39
2 No	3,618	75.61
Total	4,785	100.00

fc017: Labor Contract Period

	Freq.	%
1 Defined Preiod	714	61.18
2 Not Defined	329	28.19
3 Same as the Term of the Project	63	5.40
997 Do not Know	61	5.23
Total	1,167	100.00

fc017_1: Year

Mean	SD	Min	Max	Obs
2.09	3.21	0.00	44.00	691

fc017_2: Month

Mean	SD	Min	Max	Obs
0.28	1.23	0.00	9.00	684

fc018: Double Check on the Time When Started Working for This Employer

	Freq.	%
1 Yes	1,540	87.80
2 No	209	11.92
999 Refuse to Answer	5	0.29
Total	1,754	100.00

fc018_1: Year

Mean	SD	Min	Max	Obs
2,014.41	8.27	1,985.00	2,020.00	201

fc018_2: Month

Mean	SD	Min	Max	Obs
5.66	3.04	1.00	12.00	189

fc019_1: When Did You Start Working for This Employer(Year)

Mean	SD	Min	Max	Obs
2,017.52	6.38	1,964.00	2,020.00	2,875

fc019_2: When Did You Start Working for This Employer(Month)

Mean	SD	Min	Max	Obs
5.67	2.63	1.00	12.00	2,675

fc025: Months of Working in the Past Year

Mean	SD	Min	Max	Obs
7.34	4.23	1.00	12.00	4,784

fc026: Days of Working per Week

Mean	SD	Min	Max	Obs

5.37	1.86	1.00	7.00	4,784

fc027: Hours of Working per Day

Mean	SD	Min	Max	Obs
8.64	3.02	1.00	24.00	4,784

fc032: Periodic Pattern of Wage Payment

	Freq.	%
1 Yearly Pay	328	6.86
2 Monthly Pay	2,523	52.74
3 Weekly Pay	12	0.25
4 Daily Pay	782	16.35
5 Hourly Pay	134	2.80
6 Contract-based	302	6.31
7 Performance-based	405	8.47
8 Other	264	5.52
997 Do not Know	32	0.67
999 Refuse to Answer	2	0.04
Total	4,784	100.00

fc033: Annual Salary

Mean	SD	Min	Max	Obs
22,817.66	21,569.12	0.00	150,000.00	319

fc034: Monthly Salary

Mean	SD	Min	Max	Obs
3,372.68	3,049.14	0.00	70,000.00	2,479

fc035: Weekly Salary

Mean	SD	Min	Max	Obs
806.67	713.37	0.00	2,000.00	12

fc036: Daily Salary

Mean	SD	Min	Max	Obs
134.72	72.93	10.00	700.00	774

fc037: Hourly Salary

Mean	SD	Min	Max	Obs
15.29	15.32	5.00	100.00	129

fc038: Estimated Monthly Salary

Mean	SD	Min	Max	Obs
2,252.87	2,405.05	0.00	20,000.00	1,823

fc039: All Other Bonuses Last Year

Mean	SD	Min	Max	Obs
2,186.53	11,203.57	0.00	200,000.00	4,702

fc040: Salary Reported Net Tax

	Freq.	%
Taxable and Deducted	734	15.35
Taxable but not Deducted	398	8.32
Not Taxable	3,556	74.35
997 Do not Know	94	1.97
999 Refuse to Answer	1	0.02
Total	4,783	100.00

fc041: Payroll Tax

	Freq.	%
Percentage	200	17.67
Monthly Payment	400	35.34
Yearly Payment	292	25.80
997 Do not Know	240	21.20
Total	1,132	100.00

fc041_1: Percentage

Mean	SD	Min	Max	Obs
14.41	10.90	1.00	50.00	200

fc041_2: Monthly Tax

Mean	SD	Min	Max	Obs
1,018.20	976.34	100.00	6,100.00	400

fc041_3: Yearly Tax

Mean	SD	Min	Max	Obs
2,922.35	8,845.16	100.00	100,000.00	292

fc042_s1: Meal

	Freq.	%
0 No	3,133	65.50
1 Yes	1,650	34.50

Total	4,783	100.00

fc042_s2: Transportation

	Freq.	%
0 No	4,478	93.62
2 Yes	305	6.38
Total	4,783	100.00

fc042_s3: Housing

	Freq.	%
0 No	3,964	82.88
3 Yes	819	17.12
Total	4,783	100.00

fc042_s4: Others

	Freq.	%
0 No	4,376	91.49
4 Yes	407	8.51
Total	4,783	100.00

fc042_s5: No Benefits

	Freq.	%
0 No	2,245	46.94
5 Yes	2,538	53.06
Total	4,783	100.00

fc042_s999: Do not Know

	Freq.	%
0 No	4,753	99.37
999 Yes	30	0.63
Total	4,783	100.00

fc042_1: Meal Benefits

Mean	SD	Min	Max	Obs
368.40	353.62	10.00	3,000.00	1,514

fc042_2: Transportation Benefits

Mean	SD	Min	Max	Obs
547.32	1,019.29	10.00	10,000.00	254

fc042_3: Housing Benefits

Mean	SD	Min	Max	Obs
720.76	4,139.25	10.00	100,000.00	598

fc042_4: Other Benefits not Mentioned Above

Mean	SD	Min	Max	Obs
271.30	855.06	10.00	10,000.00	370

fd002: Location of the Self Employment Business

	Freq.	%
1 Same as the Residence	1,174	79.49
2 Another Neighborhood but Same County as the Residence	210	14.22
3 Other Domestic Location	72	4.87
4 Abroad	17	1.15
999 Refuse to Answer	4	0.27
Total	1,477	100.00

fd004: Number of Employees

Mean	SD	Min	Max	Obs
1.73	9.01	0.00	200.00	1,477

fd007: Months of Working in the Past Year

Mean	SD	Min	Max	Obs
8.98	3.65	1.00	12.00	1,477

fd008: Days of Working per Week

Mean	SD	Min	Max	Obs
5.73	1.90	1.00	7.00	1,477

fd009: Hours of Working per Day

Mean	SD	Min	Max	Obs
7.62	4.00	1.00	24.00	1,477

fe001: Days of Working on Side Jobs per Week

Mean	SD	Min	Max	Obs
4.45	2.35	0.00	7.00	537

fe002: Hours of Working on Side Jobs per Day

Mean	SD	Min	Max	Obs
6.92	3.54	1.00	24.00	537

ff001: Total Days of Working Last Year

Mean	SD	Min	Max	Obs
140.21	137.50	0.00	366.00	19,028

ff002: Has Recreational Work

	Freq.	%
1 Yes	172	2.49
2 No	6,734	97.51
Total	6,906	100.00

ff003: Did You Search for a Job Last Month

	Freq.	%
1 Yes	137	1.98
2 No	6,769	98.02
Total	6,906	100.00

ff004: Expected Time to Stop Working

	Freq.	%
Until an Age	3,062	24.33
After Some Years	750	5.96
Nonstop Given Good Health	8,484	67.42
997 Do not Know	281	2.23
999 Refuse to Answer	7	0.06
Total	12,584	100.00

ff004_1: Age by Then to Stop Working

Mean	SD	Min	Max	Obs
64.91	7.08	46.00	100.00	3,062

ff004_2: Number of Years to Continue Working

Mean	SD	Min	Max	Obs
2.86	3.07	0.00	20.00	746

ff005: Expected Time to Stop Working Affected by the Pandemic

	Freq.	%
# Years Earlier	245	1.77
# Years Delayed	414	2.99

	Freq.	%
Not Affected	12,948	93.61
997 Do not Know	216	1.56
999 Refuse to Answer	9	0.07
Total	13,832	100.00

ff005_1: Number of Years Earlier

Mean	SD	Min	Max	Obs
3.80	3.16	1.00	30.00	208

ff005_2: Number of Years Delayed

Mean	SD	Min	Max	Obs
2.86	2.22	1.00	20.00	344

fg001: Work at Home During the Pandemic

	Freq.	%
1 Yes	463	3.59
2 No	12,431	96.41
Total	12,894	100.00

fg002: Got Paid Even not Working During the Pandemic

	Freq.	%
1 Yes, Paid Less	421	3.27
2 Yes, Paid More	2	0.02
3 No	12,471	96.72
Total	12,894	100.00

fg002_1: Percentage

Mean	SD	Min	Max	Obs
77.12	28.77	1.00	100.00	405

fg002_2: Duration

Mean	SD	Min	Max	Obs
7.01	5.28	1.00	30.00	407

fg003: Self-Employed Farming Affected by the Pandemic

	Freq.	%
Would not Have This Type of Work Anyway	3,213	24.92
Worked Less	1,965	15.24
Worked More	188	1.46
Not Affected	7,501	58.17
999 Refuse to Answer	27	0.21

	Freq.	%
Total	12,894	100.00

fg003_1: Number of More Weeks

Mean	SD	Min	Max	Obs
5.43	4.08	1.00	32.00	1,850

fg003_2: Number of Fewer Weeks

Mean	SD	Min	Max	Obs
4.03	3.85	1.00	27.00	180

fg004: Employed Work Affected by the Pandemic

	Freq.	%
Would not Have This Type of Work Anyway	6,414	49.74
Worked Less	3,273	25.38
Worked More	139	1.08
Not Affected	3,046	23.62
999 Refuse to Answer	22	0.17
Total	12,894	100.00

fg004_1: Number of More Weeks

Mean	SD	Min	Max	Obs
8.29	5.63	1.00	34.00	3,144

fg004_2: Number of Fewer Weeks

Mean	SD	Min	Max	Obs
5.34	4.50	1.00	24.00	128

fg005: Self-Employed Work Affected by the Pandemic

	Freq.	%
Not Affected	10,092	78.28
Worked Less	1,433	11.11
Worked More	20	0.16
Having not Worked After the Lunar New Year	1,340	10.39
999 Refuse to Answer	8	0.06
Total	12,893	100.00

fg005_1: Number of More Weeks

Mean	SD	Min	Max	Obs
9.04	5.84	1.00	34.00	1,400

fg005_2: Number of Fewer Weeks

Mean	SD	Min	Max	Obs
7.50	6.59	1.00	24.00	20

fg008: Work Hours Affected by the Pandemic

Mean	SD	Min	Max	Obs
4.36	51.91	1.00	999.00	12,893

fg008_1: Number of More Hours per Week

Mean	SD	Min	Max	Obs
12.45	10.24	1.00	40.00	1,645

fg008_2: Number of Fewer Hours per Week

Mean	SD	Min	Max	Obs
11.08	8.73	1.00	40.00	338

fg009: Unemployment Insurance Received During the Pandemic

	Freq.	%
1 Yes	17	0.13
2 No	12,876	99.87
Total	12,893	100.00

fg010_1: Months of Payment

Mean	SD	Min	Max	Obs
4.47	2.42	1.00	8.00	15

fg010_2: Monthly Payment

Mean	SD	Min	Max	Obs
1,173.94	839.14	19.00	2,640.00	16

fh001: Retirement Processed

	Freq.	%
1 Yes	787	4.70
2 No	15,955	95.30
Total	16,742	100.00

fh002: Type of Retirement

	Freq.	%

Formal Retirement	705	89.58
Early Retirement	54	6.86
Internal Retirement First Then Formal Retirement	22	2.80
Internal Retirement but Not Yet Formal Retirement	6	0.76
Total	787	100.00

fh003_1: Year of Retirement

Mean	SD	Min	Max	Obs
2,012.15	8.45	1,972.00	2,020.00	722

fh003_2: Month

Mean	SD	Min	Max	Obs
6.93	3.48	1.00	12.00	658

fh004_1: Expected Year of Retirement

Mean	SD	Min	Max	Obs
2,022.17	2.56	2,020.00	2,026.00	6

fh004_2: Expected Month

Mean	SD	Min	Max	Obs
7.83	4.02	2.00	12.00	6

ff001_min: FF001 Min

Mean	SD	Min	Max	Obs
102.49	86.24	-1.00	250.00	318

ff001_max: FF001 Max

Mean	SD	Min	Max	Obs
104.16	90.34	-1.00	250.00	296

fc036_min: FC036 Min

Mean	SD	Min	Max	Obs
114.88	80.03	-1.00	200.00	8

fc036_max: FC036 Max

Mean	SD	Min	Max	Obs
181.13	151.21	-1.00	500.00	8

fc038_min: FC038 Min

Mean	SD	Min	Max	Obs
1,037.64	1,291.11	-1.00	5,000.00	92

fc038_max: FC038 Max

Mean	SD	Min	Max	Obs
1,427.59	1,954.08	-1.00	10,000.00	111

fc039_min: FC039 Min

Mean	SD	Min	Max	Obs
1,844.61	3,120.02	-1.00	20,000.00	71

fc039_max: FC039 Max

Mean	SD	Min	Max	Obs
2,562.11	3,543.31	-1.00	20,000.00	80

fc034_min: FC034 Min

Mean	SD	Min	Max	Obs
1,768.90	1,831.52	-1.00	5,000.00	39

fc034_max: FC034 Max

Mean	SD	Min	Max	Obs
2,604.33	3,058.37	-1.00	10,000.00	43

fc042_2_min: FC042_2 Min

Mean	SD	Min	Max	Obs
165.45	279.50	-1.00	1,000.00	47

fc042_2_max: FC042_2 Max

Mean	SD	Min	Max	Obs
258.67	362.04	-1.00	1,000.00	49

fc042_1_min: FC042_1 Min

Mean	SD	Min	Max	Obs
207.15	287.73	-1.00	1,000.00	118

fc042_1_max: FC042_1 Max

Mean	SD	Min	Max	Obs
287.55	350.68	-1.00	1,000.00	133

fc042_3_min: FC042_3 Min

Mean	SD	Min	Max	Obs
381.76	800.24	-1.00	5,000.00	170

fc042_3_max: FC042_3 Max

Mean	SD	Min	Max	Obs
675.27	1,063.10	-1.00	5,000.00	222

fc042_4_min: FC042_4 Min

Mean	SD	Min	Max	Obs
62.13	49.95	-1.00	100.00	24

fc042_4_max: FC042_4 Max

Mean	SD	Min	Max	Obs
218.70	213.45	-1.00	500.00	37

fc033_min: FC033 Min

Mean	SD	Min	Max	Obs
14,285.29	19,024.17	-1.00	50,000.00	7

fc033_max: FC033 Max

Mean	SD	Min	Max	Obs
27,777.44	33,829.95	-1.00	100,000.00	9

fc037_min: FC037 Min

Mean	SD	Min	Max	Obs
12.25	12.92	-1.00	30.00	4

fc037_max: FC037 Max

Mean	SD	Min	Max	Obs
15.80	13.72	-1.00	30.00	5

xworking: Working

Mean	SD	Min	Max	Obs
0.64	0.48	0.00	1.00	19,358

xemployed: Self-Employed

Mean	SD	Min	Max	Obs
0.76	0.42	0.00	1.00	6,263

xfgsample: Selected to Answer Work Questions During the Pandemic

Mean	SD	Min	Max	Obs
0.67	0.47	0.00	1.00	19,353

xworkjuststarted: Current Employed Job Started no More Than a Year or a Month

Mean	SD	Min	Max	Obs
0.64	0.86	0.00	2.00	4,784

xmainjobdays: Days of Main Job

Mean	SD	Min	Max	Obs
91.76	143.83	0.00	364.00	19,355

xhhldfarmdays: Days of Household Farming

Mean	SD	Min	Max	Obs
66.79	109.19	0.00	336.00	19,355

xsidejobdays: Days of Side Job

Mean	SD	Min	Max	Obs
6.42	43.07	0.00	364.00	19,355

xgetjob: Started to Work Since LastIW

Mean	SD	Min	Max	Obs
0.08	0.27	0.00	1.00	19,358

xquitjob: Stopped Working Since LastIW

Mean	SD	Min	Max	Obs
0.10	0.30	0.00	1.00	17,840

proxy_7: Answered by a Proxy if Eligible

	Freq.	%
1 Yes	1,775	99.22
2 No	14	0.78
Total	1,789	100.00

xrage: Age

Mean	SD	Min	Max	Obs
61.56	10.07	18.00	108.00	19,361

xiwmonth: Interview Month

Mean	SD	Min	Max	Obs
7.52	0.51	6.00	11.00	19,361

xiwyear: Interview Year

Mean	SD	Min	Max	Obs
2,020.00	0.04	2,017.00	2,020.00	19,361

ziwtime: Preloaded: Time of LastIW

A String Variable	
Obs:	19,232

ziwyear: Preloaded: Year of LastIW

Mean	SD	Min	Max	Obs
2,017.72	0.99	2,011.00	2,018.00	19,232

zworking: Preloaded: Working LastIW

	Freq.	%
0 No	6,446	34.17
1 Working	12,351	65.47
2 Never Worked	68	0.36
Total	18,865	100.00

zrretired: Preloaded: Retirement LastIW

Mean	SD	Min	Max	Obs
0.14	0.35	0.00	1.00	18,888

zfd011_1: Preloaded: Starting Time for the Employer LastIW

Mean	SD	Min	Max	Obs

2,010.64	10.36	1,963.00	2,018.00	4,747

versionID: Version ID

A String Variable	
Obs:	19,361

MODULE G1

Household Income

householdID: Household ID

A String Variable	
Obs:	11,377

communityID: Community ID

A String Variable	
Obs:	11,377

gb001: Family Financial Respondent

	Freq.	%
1 HHMemberName[1]	562	4.94
2 HHMemberName[2]	226	1.99
3 HHMemberName[3]	31	0.27
4 HHMemberName[4]	18	0.16
5 HHMemberName[5]	3	0.03
6 HHMemberName[6]	1	0.01
8 HHMemberName[8]	1	0.01
26 Main Respondent	6,685	58.81
27 Main Respondent's Spouse	3,841	33.79
Total	11,368	100.00

gb002_1_: Wage and Bonus Income HHMemberName[1] Receive

	Freq.	%
1 Yes	2,338	48.12
2 No	2,430	50.01
997 Do not Know	90	1.85
999 Refused to Answer	1	0.02
Total	4,859	100.00

gb002_2_: Wage and Bonus Income HHMemberName[2] Receive

	Freq.	%
1 Yes	970	38.89
2 No	1,473	59.06
997 Do not Know	50	2.00
999 Refused to Answer	1	0.04
Total	2,494	100.00

gb002_3_: Wage and Bonus Income HHMemberName[3] Receive

	Freq.	%
1 Yes	215	26.38
2 No	588	72.15
997 Do not Know	12	1.47
Total	815	100.00

gb002_4_: Wage and Bonus Income HHMemberName[4] Receive

	Freq.	%
1 Yes	114	32.66
2 No	223	63.90
997 Do not Know	12	3.44
Total	349	100.00

gb002_5_: Wage and Bonus Income HHMemberName[5] Receive

	Freq.	%
1 Yes	33	34.74
2 No	61	64.21
997 Do not Know	1	1.05
Total	95	100.00

gb002_6_: Wage and Bonus Income HHMemberName[6] Receive

	Freq.	%
1 Yes	11	28.21
2 No	27	69.23
997 Do not Know	1	2.56
Total	39	100.00

gb002_7_: Wage and Bonus Income HHMemberName[7] Receive

	Freq.	%
1 Yes	5	26.32
2 No	13	68.42
997 Do not Know	1	5.26
Total	19	100.00

gb002_8_: Wage and Bonus Income HHMemberName[8] Receive

	Freq.	%
1 Yes	5	50.00
2 No	5	50.00
Total	10	100.00

gb002_9_: Wage and Bonus Income HHMemberName[9] Receive

	Freq.	%
1 Yes	1	50.00
2 No	1	50.00
Total	2	100.00

gb002_10_: Wage and Bonus Income HHMemberName[10] Receive

	Freq.	%
1 Yes	3	60.00
2 No	2	40.00
Total	5	100.00

gb002_11_: Wage and Bonus Income HHMemberName[11] Receive

	Freq.	%
1 Yes	1	50.00
2 No	1	50.00
Total	2	100.00

gb002_12_: Wage and Bonus Income HHMemberName[12] Receive

	Freq.	%
2 No	1	100.00
Total	1	100.00

gb002_13_: Wage and Bonus Income HHMemberName[13] Receive

	Freq.	%
2 No	1	100.00
Total	1	100.00

gb002_14_: Wage and Bonus Income HHMemberName[14] Receive

	Freq.	%
2 No	1	100.00
Total	1	100.00

gb003_1_: Amount HHMemberName[1] Receive

Mean	SD	Min	Max	Obs

29,183.18	45,651.84	-1.00	1,400,000.00	2,338

gb003_2_: Amount HHMemberName[2] Receive

Mean	SD	Min	Max	Obs
26,761.64	35,135.18	-1.00	400,000.00	970

gb003_3_: Amount HHMemberName[3] Receive

Mean	SD	Min	Max	Obs
20,487.67	23,741.21	-1.00	180,000.00	215

gb003_4_: Amount HHMemberName[4] Receive

Mean	SD	Min	Max	Obs
27,791.90	35,562.11	-1.00	200,000.00	114

gb003_5_: Amount HHMemberName[5] Receive

Mean	SD	Min	Max	Obs
17,845.21	22,823.05	-1.00	100,000.00	33

gb003_6_: Amount HHMemberName[6] Receive

Mean	SD	Min	Max	Obs
46,908.91	67,955.20	-1.00	240,000.00	11

gb003_7_: Amount HHMemberName[7] Receive

Mean	SD	Min	Max	Obs
161,999.60	313,241.38	-1.00	720,000.00	5

gb003_8_: Amount HHMemberName[8] Receive

Mean	SD	Min	Max	Obs
23,560.00	27,748.30	4,000.00	72,000.00	5

gb003_9_: Amount HHMemberName[9] Receive

Mean	SD	Min	Max	Obs
19,000.00	.	19,000.00	19,000.00	1

gb003_10_: Amount HHMemberName[10] Receive

Mean	SD	Min	Max	Obs

15,666.67	6,658.33	8,000.00	20,000.00	3

gb003_11_: Amount HHMemberName[11] Receive

Mean	SD	Min	Max	Obs
-1.00	.	-1.00	-1.00	1

gb003_min_1_: Min Bracket of gb003_1_

Mean	SD	Min	Max	Obs
19,782.79	22,959.49	-1.00	100,000.00	461

gb003_max_1_: Max Bracket of gb003_1_

Mean	SD	Min	Max	Obs
27,451.10	29,720.94	-1.00	100,000.00	463

gb003_min_2_: Min Bracket of gb003_2_

Mean	SD	Min	Max	Obs
16,725.26	22,308.95	-1.00	100,000.00	226

gb003_max_2_: Max Bracket of gb003_2_

Mean	SD	Min	Max	Obs
24,108.31	28,800.36	-1.00	100,000.00	230

gb003_min_3_: Min Bracket of gb003_3_

Mean	SD	Min	Max	Obs
14,183.27	19,668.03	-1.00	100,000.00	49

gb003_max_3_: Max Bracket of gb003_3_

Mean	SD	Min	Max	Obs
24,038.12	30,212.58	-1.00	100,000.00	52

gb003_min_4_: Min Bracket of gb003_4_

Mean	SD	Min	Max	Obs
10,869.09	14,667.29	-1.00	50,000.00	23

gb003_max_4_: Max Bracket of gb003_4_

Mean	SD	Min	Max	Obs

19,564.74	30,782.29	-1.00	100,000.00	23

gb003_min_5_: Min Bracket of gb003_5_

Mean	SD	Min	Max	Obs
12,857.00	12,198.93	-1.00	30,000.00	7

gb003_max_5_: Max Bracket of gb003_5_

Mean	SD	Min	Max	Obs
21,874.75	18,114.57	-1.00	50,000.00	8

gb003_min_6_: Min Bracket of gb003_6_

Mean	SD	Min	Max	Obs
4,999.50	7,071.77	-1.00	10,000.00	2

gb003_max_6_: Max Bracket of gb003_6_

Mean	SD	Min	Max	Obs
14,999.50	21,213.91	-1.00	30,000.00	2

gb003_min_7_: Min Bracket of gb003_7_

Mean	SD	Min	Max	Obs
-1.00	.	-1.00	-1.00	1

gb003_max_7_: Max Bracket of gb003_7_

Mean	SD	Min	Max	Obs
2,499.50	3,536.24	-1.00	5,000.00	2

gb003_min_11_: Min Bracket of gb003_11_

Mean	SD	Min	Max	Obs
10,000.00	.	10,000.00	10,000.00	1

gb003_max_11_: Max Bracket of gb003_11_

Mean	SD	Min	Max	Obs
30,000.00	.	30,000.00	30,000.00	1

gb004_1_: Exclude Insurance and Some Others for HHMemberName[1]

	Freq.	%

	Freq.	%
1 Yes	801	34.26
2 No	1,210	51.75
997 Do not Know	326	13.94
999 Refused to Answer	1	0.04
Total	2,338	100.00

gb004_2_: Exclude Insurance and Some Others for HHMemberName[2]

	Freq.	%
1 Yes	331	34.12
2 No	490	50.52
997 Do not Know	149	15.36
Total	970	100.00

gb004_3_: Exclude Insurance and Some Others for HHMemberName[3]

	Freq.	%
1 Yes	56	26.05
2 No	114	53.02
997 Do not Know	45	20.93
Total	215	100.00

gb004_4_: Exclude Insurance and Some Others for HHMemberName[4]

	Freq.	%
1 Yes	29	25.44
2 No	60	52.63
997 Do not Know	25	21.93
Total	114	100.00

gb004_5_: Exclude Insurance and Some Others for HHMemberName[5]

	Freq.	%
1 Yes	8	24.24
2 No	15	45.45
997 Do not Know	10	30.30
Total	33	100.00

gb004_6_: Exclude Insurance and Some Others for HHMemberName[6]

	Freq.	%
1 Yes	8	72.73
2 No	2	18.18
997 Do not Know	1	9.09
Total	11	100.00

gb004_7_: Exclude Insurance and Some Others for HHMemberName[7]

	Freq.	%
1 Yes	1	20.00

	Freq.	%
2 No	2	40.00
997 Do not Know	2	40.00
Total	5	100.00

gb004_8_: Exclude Insurance and Some Others for HHMemberName[8]

	Freq.	%
1 Yes	1	20.00
2 No	3	60.00
997 Do not Know	1	20.00
Total	5	100.00

gb004_9_: Exclude Insurance and Some Others for HHMemberName[9]

	Freq.	%
997 Do not Know	1	100.00
Total	1	100.00

gb004_10_: Exclude Insurance and Some Others for HHMemberName[10]

	Freq.	%
2 No	2	66.67
997 Do not Know	1	33.33
Total	3	100.00

gb004_11_: Exclude Insurance and Some Others for HHMemberName[11]

	Freq.	%
1 Yes	1	100.00
Total	1	100.00

gb005_1_: Total Excluded Amount for HHMemberName[1]

	Freq.	%
1 Yuan/Month	1,085	46.43
2 Yuan/Year	202	8.64
3 Percentage of Wage	55	2.35
4 No	995	42.58
Total	2,337	100.00

gb005_2_: Total Excluded Amount for HHMemberName[2]

	Freq.	%
1 Yuan/Month	467	48.14
2 Yuan/Year	87	8.97
3 Percentage of Wage	18	1.86
4 No	398	41.03
Total	970	100.00

gb005_3_: Total Excluded Amount for HHMemberName[3]

	Freq.	%
1 Yuan/Month	86	40.00
2 Yuan/Year	19	8.84
3 Percentage of Wage	2	0.93
4 No	108	50.23
Total	215	100.00

gb005_4_: Total Excluded Amount for HHMemberName[4]

	Freq.	%
1 Yuan/Month	52	45.61
2 Yuan/Year	4	3.51
3 Percentage of Wage	1	0.88
4 No	57	50.00
Total	114	100.00

gb005_5_: Total Excluded Amount for HHMemberName[5]

	Freq.	%
1 Yuan/Month	15	45.45
2 Yuan/Year	1	3.03
3 Percentage of Wage	1	3.03
4 No	16	48.48
Total	33	100.00

gb005_6_: Total Excluded Amount for HHMemberName[6]

	Freq.	%
1 Yuan/Month	7	63.64
2 Yuan/Year	2	18.18
4 No	2	18.18
Total	11	100.00

gb005_7_: Total Excluded Amount for HHMemberName[7]

	Freq.	%
1 Yuan/Month	2	40.00
2 Yuan/Year	1	20.00
4 No	2	40.00
Total	5	100.00

gb005_8_: Total Excluded Amount for HHMemberName[8]

	Freq.	%
1 Yuan/Month	2	40.00
4 No	3	60.00
Total	5	100.00

gb005_9_: Total Excluded Amount for HHMemberName[9]

	Freq.	%
1 Yuan/Month	1	100.00
Total	1	100.00

gb005_10_: Total Excluded Amount for HHMemberName[10]

	Freq.	%
1 Yuan/Month	1	33.33
4 No	2	66.67
Total	3	100.00

gb005_11_: Total Excluded Amount for HHMemberName[11]

	Freq.	%
1 Yuan/Month	1	100.00
Total	1	100.00

gb005_1_1_: Yuan/Month for HHMemberName[1]

Mean	SD	Min	Max	Obs
315.86	844.47	-1.00	10,000.00	1,085

gb005_1_2_: Yuan/Month for HHMemberName[2]

Mean	SD	Min	Max	Obs
246.52	591.33	-1.00	6,000.00	467

gb005_1_3_: Yuan/Month for HHMemberName[3]

Mean	SD	Min	Max	Obs
203.58	456.19	-1.00	3,000.00	86

gb005_1_4_: Yuan/Month for HHMemberName[4]

Mean	SD	Min	Max	Obs
226.33	435.19	-1.00	2,000.00	52

gb005_1_5_: Yuan/Month for HHMemberName[5]

Mean	SD	Min	Max	Obs
159.20	346.39	-1.00	1,000.00	15

gb005_1_6_: Yuan/Month for HHMemberName[6]

Mean	SD	Min	Max	Obs
427.86	787.25	-1.00	2,000.00	7

gb005_1_7_: Yuan/Month for HHMemberName[7]

Mean	SD	Min	Max	Obs
199.50	283.55	-1.00	400.00	2

gb005_1_8_: Yuan/Month for HHMemberName[8]

Mean	SD	Min	Max	Obs
279.50	396.69	-1.00	560.00	2

gb005_1_9_: Yuan/Month for HHMemberName[9]

Mean	SD	Min	Max	Obs
-1.00	.	-1.00	-1.00	1

gb005_1_10_: Yuan/Month for HHMemberName[10]

Mean	SD	Min	Max	Obs
-1.00	.	-1.00	-1.00	1

gb005_1_11_: Yuan/Month for HHMemberName[11]

Mean	SD	Min	Max	Obs
-1.00	.	-1.00	-1.00	1

gb005_1_min_1_: Min Bracket of gb005_1_1_

Mean	SD	Min	Max	Obs
412.45	725.80	-1.00	3,000.00	743

gb005_1_max_1_: Max Bracket of gb005_1_1_

Mean	SD	Min	Max	Obs
497.17	813.90	-1.00	3,000.00	759

gb005_1_min_2_: Min Bracket of gb005_1_2_

Mean	SD	Min	Max	Obs
346.98	704.13	-1.00	3,000.00	319

gb005_1_max_2_: Max Bracket of gb005_1_2_

Mean	SD	Min	Max	Obs
379.57	750.75	-1.00	3,000.00	319

gb005_1_min_3_: Min Bracket of gb005_1_3_

Mean	SD	Min	Max	Obs
453.41	908.93	-1.00	3,000.00	61

gb005_1_max_3_: Max Bracket of gb005_1_3_

Mean	SD	Min	Max	Obs
405.87	826.55	-1.00	3,000.00	61

gb005_1_min_4_: Min Bracket of gb005_1_4_

Mean	SD	Min	Max	Obs
225.79	439.58	-1.00	2,000.00	34

gb005_1_max_4_: Max Bracket of gb005_1_4_

Mean	SD	Min	Max	Obs
296.51	527.00	-1.00	2,000.00	35

gb005_1_min_5_: Min Bracket of gb005_1_5_

Mean	SD	Min	Max	Obs
232.58	579.25	-1.00	2,000.00	12

gb005_1_max_5_: Max Bracket of gb005_1_5_

Mean	SD	Min	Max	Obs
316.00	613.61	-1.00	2,000.00	12

gb005_1_min_6_: Min Bracket of gb005_1_6_

Mean	SD	Min	Max	Obs
199.40	274.41	-1.00	500.00	5

gb005_1_max_6_: Max Bracket of gb005_1_6_

Mean	SD	Min	Max	Obs
399.40	548.27	-1.00	1,000.00	5

gb005_1_min_7_: Min Bracket of gb005_1_7_

Mean	SD	Min	Max	Obs
-1.00	.	-1.00	-1.00	1

gb005_1_max_7_: Max Bracket of gb005_1_7_

Mean	SD	Min	Max	Obs
-1.00	.	-1.00	-1.00	1

gb005_1_min_8_: Min Bracket of gb005_1_8_

Mean	SD	Min	Max	Obs
-1.00	.	-1.00	-1.00	1

gb005_1_max_8_: Max Bracket of gb005_1_8_

Mean	SD	Min	Max	Obs
-1.00	.	-1.00	-1.00	1

gb005_1_min_9_: Min Bracket of gb005_1_9_

Mean	SD	Min	Max	Obs
-1.00	.	-1.00	-1.00	1

gb005_1_max_9_: Max Bracket of gb005_1_9_

Mean	SD	Min	Max	Obs
-1.00	.	-1.00	-1.00	1

gb005_1_min_10_: Min Bracket of gb005_1_10_

Mean	SD	Min	Max	Obs
-1.00	.	-1.00	-1.00	1

gb005_1_max_10_: Max Bracket of gb005_1_10_

Mean	SD	Min	Max	Obs
-1.00	.	-1.00	-1.00	1

gb005_1_min_11_: Min Bracket of gb005_1_11_

Mean	SD	Min	Max	Obs
-1.00	.	-1.00	-1.00	1

gb005_1_max_11_: Max Bracket of gb005_1_11_

Mean	SD	Min	Max	Obs
-1.00	.	-1.00	-1.00	1

gb005_2_1_: Yuan/Year for HHMemberName[1]

Mean	SD	Min	Max	Obs
8,460.55	16,827.59	24.00	140,000.00	202

gb005_2_2_: Yuan/Year for HHMemberName[2]

Mean	SD	Min	Max	Obs
7,615.05	9,505.03	70.00	40,000.00	87

gb005_2_3_: Yuan/Year for HHMemberName[3]

Mean	SD	Min	Max	Obs
1,986.84	2,799.37	100.00	10,000.00	19

gb005_2_4_: Yuan/Year for HHMemberName[4]

Mean	SD	Min	Max	Obs
3,077.50	2,798.69	450.00	7,000.00	4

gb005_2_5_: Yuan/Year for HHMemberName[5]

Mean	SD	Min	Max	Obs
4,000.00	.	4,000.00	4,000.00	1

gb005_2_6_: Yuan/Year for HHMemberName[6]

Mean	SD	Min	Max	Obs
7,500.00	3,535.53	5,000.00	10,000.00	2

gb005_2_7_: Yuan/Year for HHMemberName[7]

Mean	SD	Min	Max	Obs
430.00	.	430.00	430.00	1

gb005_3_1_: Percentage of Wage for HHMemberName[1]

Mean	SD	Min	Max	Obs
17.90	17.03	0.80	100.00	55

gb005_3_2_: Percentage of Wage for HHMemberName[2]

Mean	SD	Min	Max	Obs
19.78	11.59	3.00	50.00	18

gb005_3_3_: Percentage of Wage for HHMemberName[3]

Mean	SD	Min	Max	Obs
35.00	21.21	20.00	50.00	2

gb005_3_4_: Percentage of Wage for HHMemberName[4]

Mean	SD	Min	Max	Obs
7.00	.	7.00	7.00	1

gb005_3_5_: Percentage of Wage for HHMemberName[5]

Mean	SD	Min	Max	Obs
10.00	.	10.00	10.00	1

gb006_1__s1: Pension HHMemberName[1] Receive

	Freq.	%
0 No	4,479	92.18
1 Yes	380	7.82
Total	4,859	100.00

gb006_2__s1: Pension HHMemberName[2] Receive

	Freq.	%
0 No	2,314	92.78
1 Yes	180	7.22
Total	2,494	100.00

gb006_3__s1: Pension HHMemberName[3] Receive

	Freq.	%
0 No	759	93.13
1 Yes	56	6.87
Total	815	100.00

gb006_4__s1: Pension HHMemberName[4] Receive

	Freq.	%
0 No	332	95.13
1 Yes	17	4.87
Total	349	100.00

gb006_5__s1: Pension HHMemberName[5] Receive

	Freq.	%
0 No	91	95.79
1 Yes	4	4.21
Total	95	100.00

gb006_6__s1: Pension HHMemberName[6] Receive

	Freq.	%
0 No	37	94.87
1 Yes	2	5.13
Total	39	100.00

gb006_7__s1: Pension HHMemberName[7] Receive

	Freq.	%
0 No	11,376	99.99
1 Yes	1	0.01
Total	11,377	100.00

gb006_8__s1: Pension HHMemberName[8] Receive

	Freq.	%
0 No	11,377	100.00
Total	11,377	100.00

gb006_9__s1: Pension HHMemberName[9] Receive

	Freq.	%
0 No	11,377	100.00
Total	11,377	100.00

gb006_10__s1: Pension HHMemberName[10] Receive

	Freq.	%
0 No	11,377	100.00
Total	11,377	100.00

gb006_11__s1: Pension HHMemberName[11] Receive

	Freq.	%
0 No	11,377	100.00
Total	11,377	100.00

gb006_12__s1: Pension HHMemberName[12] Receive

	Freq.	%
0 No	11,377	100.00
Total	11,377	100.00

gb006_13__s1: Pension HHMemberName[13] Receive

	Freq.	%
0 No	11,377	100.00
Total	11,377	100.00

gb006_14__s1: Pension HHMemberName[14] Receive

	Freq.	%
0 No	11,377	100.00
Total	11,377	100.00

gb006_1__s2: Unemployment Compensation HHMemberName[1] Receive

	Freq.	%
0 No	4,821	99.22
2 Yes	38	0.78
Total	4,859	100.00

gb006_2__s2: Unemployment Compensation HHMemberName[2] Receive

	Freq.	%
0 No	2,478	99.36
2 Yes	16	0.64
Total	2,494	100.00

gb006_3__s2: Unemployment Compensation HHMemberName[3] Receive

	Freq.	%
0 No	811	99.51
2 Yes	4	0.49
Total	815	100.00

gb006_4__s2: Unemployment Compensation HHMemberName[4] Receive

	Freq.	%
0 No	346	99.14
2 Yes	3	0.86
Total	349	100.00

gb006_5__s2: Unemployment Compensation HHMemberName[5] Receive

	Freq.	%

0 No	95	100.00
Total	95	100.00

gb006_6__s2: Unemployment Compensation HHMemberName[6] Receive

	Freq.	%
0 No	39	100.00
Total	39	100.00

gb006_7__s2: Unemployment Compensation HHMemberName[7] Receive

	Freq.	%
0 No	11,377	100.00
Total	11,377	100.00

gb006_8__s2: Unemployment Compensation HHMemberName[8] Receive

	Freq.	%
0 No	11,377	100.00
Total	11,377	100.00

gb006_9__s2: Unemployment Compensation HHMemberName[9] Receive

	Freq.	%
0 No	11,377	100.00
Total	11,377	100.00

gb006_10__s2: Unemployment Compensation HHMemberName[10] Receive

	Freq.	%
0 No	11,377	100.00
Total	11,377	100.00

gb006_11__s2: Unemployment Compensation HHMemberName[11] Receive

	Freq.	%
0 No	11,377	100.00
Total	11,377	100.00

gb006_12__s2: Unemployment Compensation HHMemberName[12] Receive

	Freq.	%
0 No	11,377	100.00
Total	11,377	100.00

gb006_13__s2: Unemployment Compensation HHMemberName[13] Receive

	Freq.	%
0 No	11,377	100.00
Total	11,377	100.00

gb006_14__s2: Unemployment Compensation HHMemberName[14] Receive

	Freq.	%
0 No	11,377	100.00
Total	11,377	100.00

gb006_1__s3: Pension Voucher HHMemberName[1] Receive

	Freq.	%
0 No	4,835	99.51
3 Yes	24	0.49
Total	4,859	100.00

gb006_2__s3: Pension Voucher HHMemberName[2] Receive

	Freq.	%
0 No	2,479	99.40
3 Yes	15	0.60
Total	2,494	100.00

gb006_3__s3: Pension Voucher HHMemberName[3] Receive

	Freq.	%
0 No	813	99.75
3 Yes	2	0.25
Total	815	100.00

gb006_4__s3: Pension Voucher HHMemberName[4] Receive

	Freq.	%
0 No	346	99.14
3 Yes	3	0.86
Total	349	100.00

gb006_5__s3: Pension Voucher HHMemberName[5] Receive

	Freq.	%
0 No	95	100.00
Total	95	100.00

gb006_6__s3: Pension Voucher HHMemberName[6] Receive

	Freq.	%

0 No	39	100.00
Total	39	100.00

gb006_7__s3: Pension Voucher HHMemberName[7] Receive

	Freq.	%
0 No	11,377	100.00
Total	11,377	100.00

gb006_8__s3: Pension Voucher HHMemberName[8] Receive

	Freq.	%
0 No	11,377	100.00
Total	11,377	100.00

gb006_9__s3: Pension Voucher HHMemberName[9] Receive

	Freq.	%
0 No	11,377	100.00
Total	11,377	100.00

gb006_10__s3: Pension Voucher HHMemberName[10] Receive

	Freq.	%
0 No	11,377	100.00
Total	11,377	100.00

gb006_11__s3: Pension Voucher HHMemberName[11] Receive

	Freq.	%
0 No	11,377	100.00
Total	11,377	100.00

gb006_12__s3: Pension Voucher HHMemberName[12] Receive

	Freq.	%
0 No	11,377	100.00
Total	11,377	100.00

gb006_13__s3: Pension Voucher HHMemberName[13] Receive

	Freq.	%
0 No	11,377	100.00
Total	11,377	100.00

gb006_14__s3: Pension Voucher HHMemberName[14] Receive

	Freq.	%
0 No	11,377	100.00
Total	11,377	100.00

gb006_1__s4: Pension Subsidy for the Oldest Old HHMemberName[1] Receive

	Freq.	%
0 No	4,746	97.67
4 Yes	113	2.33
Total	4,859	100.00

gb006_2__s4: Pension Subsidy for the Oldest Old HHMemberName[2] Receive

	Freq.	%
0 No	2,440	97.83
4 Yes	54	2.17
Total	2,494	100.00

gb006_3__s4: Pension Subsidy for the Oldest Old HHMemberName[3] Receive

	Freq.	%
0 No	793	97.30
4 Yes	22	2.70
Total	815	100.00

gb006_4__s4: Pension Subsidy for the Oldest Old HHMemberName[4] Receive

	Freq.	%
0 No	337	96.56
4 Yes	12	3.44
Total	349	100.00

gb006_5__s4: Pension Subsidy for the Oldest Old HHMemberName[5] Receive

	Freq.	%
0 No	93	97.89
4 Yes	2	2.11
Total	95	100.00

gb006_6__s4: Pension Subsidy for the Oldest Old HHMemberName[6] Receive

	Freq.	%
0 No	38	97.44
4 Yes	1	2.56
Total	39	100.00

gb006_7__s4: Pension Subsidy for the Oldest Old HHMemberName[7] Receive

	Freq.	%
0 No	11,377	100.00
Total	11,377	100.00

gb006_8__s4: Pension Subsidy for the Oldest Old HHMemberName[8] Receive

	Freq.	%
0 No	11,377	100.00
Total	11,377	100.00

gb006_9__s4: Pension Subsidy for the Oldest Old HHMemberName[9] Receive

	Freq.	%
0 No	11,377	100.00
Total	11,377	100.00

gb006_10__s4: Pension Subsidy for the Oldest Old HHMemberName[10] Receive

	Freq.	%
0 No	11,377	100.00
Total	11,377	100.00

gb006_11__s4: Pension Subsidy for the Oldest Old HHMemberName[11] Receive

	Freq.	%
0 No	11,377	100.00
Total	11,377	100.00

gb006_12__s4: Pension Subsidy for the Oldest Old HHMemberName[12] Receive

	Freq.	%
0 No	11,377	100.00
Total	11,377	100.00

gb006_13__s4: Pension Subsidy for the Oldest Old HHMemberName[13] Receive

	Freq.	%
0 No	11,377	100.00
Total	11,377	100.00

gb006_14__s4: Pension Subsidy for the Oldest Old HHMemberName[14] Receive

	Freq.	%
0 No	11,377	100.00
Total	11,377	100.00

gb006_1__s5: Workers' Industrial Accident Compensation HHMemberName[1] Receive

	Freq.	%
0 No	4,807	98.93
5 Yes	52	1.07
Total	4,859	100.00

gb006_2__s5: Workers' Industrial Accident Compensation HHMemberName[2] Receive

	Freq.	%
0 No	2,477	99.32
5 Yes	17	0.68
Total	2,494	100.00

gb006_3__s5: Workers' Industrial Accident Compensation HHMemberName[3] Receive

	Freq.	%
0 No	808	99.14
5 Yes	7	0.86
Total	815	100.00

gb006_4__s5: Workers' Industrial Accident Compensation HHMemberName[4] Receive

	Freq.	%
0 No	345	98.85
5 Yes	4	1.15
Total	349	100.00

gb006_5__s5: Workers' Industrial Accident Compensation HHMemberName[5] Receive

	Freq.	%
0 No	95	100.00
Total	95	100.00

gb006_6__s5: Workers' Industrial Accident Compensation HHMemberName[6] Receive

	Freq.	%
0 No	39	100.00
Total	39	100.00

gb006_7__s5: Workers' Industrial Accident Compensation HHMemberName[7] Receive

	Freq.	%

	Freq.	%
0 No	11,377	100.00
Total	11,377	100.00

gb006_8__s5: Workers' Industrial Accident Compensation HHMemberName[8] Receive

	Freq.	%
0 No	11,377	100.00
Total	11,377	100.00

gb006_9__s5: Workers' Industrial Accident Compensation HHMemberName[9] Receive

	Freq.	%
0 No	11,377	100.00
Total	11,377	100.00

gb006_10__s5: Workers' Industrial Accident Compensation HHMemberName[10] Receive

	Freq.	%
0 No	11,377	100.00
Total	11,377	100.00

gb006_11__s5: Workers' Industrial Accident Compensation HHMemberName[11] Receive

	Freq.	%
0 No	11,377	100.00
Total	11,377	100.00

gb006_12__s5: Workers' Industrial Accident Compensation HHMemberName[12] Receive

	Freq.	%
0 No	11,377	100.00
Total	11,377	100.00

gb006_13__s5: Workers' Industrial Accident Compensation HHMemberName[13] Receive

	Freq.	%
0 No	11,377	100.00
Total	11,377	100.00

gb006_14__s5: Workers' Industrial Accident Compensation HHMemberName[14] Receive

	Freq.	%

	Freq.	%
0 No	11,377	100.00
Total	11,377	100.00

gb006_1__s6: Elderly Family Planning Subsidy HHMemberName[1] Receive

	Freq.	%
0 No	4,813	99.05
6 Yes	46	0.95
Total	4,859	100.00

gb006_2__s6: Elderly Family Planning Subsidy HHMemberName[2] Receive

	Freq.	%
0 No	2,472	99.12
6 Yes	22	0.88
Total	2,494	100.00

gb006_3__s6: Elderly Family Planning Subsidy HHMemberName[3] Receive

	Freq.	%
0 No	812	99.63
6 Yes	3	0.37
Total	815	100.00

gb006_4__s6: Elderly Family Planning Subsidy HHMemberName[4] Receive

	Freq.	%
0 No	343	98.28
6 Yes	6	1.72
Total	349	100.00

gb006_5__s6: Elderly Family Planning Subsidy HHMemberName[5] Receive

	Freq.	%
0 No	95	100.00
Total	95	100.00

gb006_6__s6: Elderly Family Planning Subsidy HHMemberName[6] Receive

	Freq.	%
0 No	39	100.00
Total	39	100.00

gb006_7__s6: Elderly Family Planning Subsidy HHMemberName[7] Receive

	Freq.	%
0 No	11,377	100.00
Total	11,377	100.00

gb006_8__s6: Elderly Family Planning Subsidy HHMemberName[8] Receive

	Freq.	%
0 No	11,377	100.00
Total	11,377	100.00

gb006_9__s6: Elderly Family Planning Subsidy HHMemberName[9] Receive

	Freq.	%
0 No	11,377	100.00
Total	11,377	100.00

gb006_10__s6: Elderly Family Planning Subsidy HHMemberName[10] Receive

	Freq.	%
0 No	11,377	100.00
Total	11,377	100.00

gb006_11__s6: Elderly Family Planning Subsidy HHMemberName[11] Receive

	Freq.	%
0 No	11,377	100.00
Total	11,377	100.00

gb006_12__s6: Elderly Family Planning Subsidy HHMemberName[12] Receive

	Freq.	%
0 No	11,377	100.00
Total	11,377	100.00

gb006_13__s6: Elderly Family Planning Subsidy HHMemberName[13] Receive

	Freq.	%
0 No	11,377	100.00
Total	11,377	100.00

gb006_14__s6: Elderly Family Planning Subsidy HHMemberName[14] Receive

	Freq.	%
0 No	11,377	100.00
Total	11,377	100.00

gb006_1__s7: Medical Aid HHMemberName[1] Receive

	Freq.	%
0 No	4,796	98.70
7 Yes	63	1.30

Total	4,859	100.00

gb006_2__s7: Medical Aid HHMemberName[2] Receive

	Freq.	%
0 No	2,463	98.76
7 Yes	31	1.24
Total	2,494	100.00

gb006_3__s7: Medical Aid HHMemberName[3] Receive

	Freq.	%
0 No	806	98.90
7 Yes	9	1.10
Total	815	100.00

gb006_4__s7: Medical Aid HHMemberName[4] Receive

	Freq.	%
0 No	346	99.14
7 Yes	3	0.86
Total	349	100.00

gb006_5__s7: Medical Aid HHMemberName[5] Receive

	Freq.	%
0 No	95	100.00
Total	95	100.00

gb006_6__s7: Medical Aid HHMemberName[6] Receive

	Freq.	%
0 No	38	97.44
7 Yes	1	2.56
Total	39	100.00

gb006_7__s7: Medical Aid HHMemberName[7] Receive

	Freq.	%
0 No	11,377	100.00
Total	11,377	100.00

gb006_8__s7: Medical Aid HHMemberName[8] Receive

	Freq.	%
0 No	11,377	100.00
Total	11,377	100.00

gb006_9__s7: Medical Aid HHMemberName[9] Receive

	Freq.	%
0 No	11,377	100.00
Total	11,377	100.00

gb006_10__s7: Medical Aid HHMemberName[10] Receive

	Freq.	%
0 No	11,377	100.00
Total	11,377	100.00

gb006_11__s7: Medical Aid HHMemberName[11] Receive

	Freq.	%
0 No	11,377	100.00
Total	11,377	100.00

gb006_12__s7: Medical Aid HHMemberName[12] Receive

	Freq.	%
0 No	11,377	100.00
Total	11,377	100.00

gb006_13__s7: Medical Aid HHMemberName[13] Receive

	Freq.	%
0 No	11,377	100.00
Total	11,377	100.00

gb006_14__s7: Medical Aid HHMemberName[14] Receive

	Freq.	%
0 No	11,377	100.00
Total	11,377	100.00

gb006_1__s8: Other Government Subsidy HHMemberName[1] Receive

	Freq.	%
0 No	4,673	96.17
8 Yes	186	3.83
Total	4,859	100.00

gb006_2__s8: Other Government Subsidy HHMemberName[2] Receive

	Freq.	%
0 No	2,422	97.11

	Freq.	%
8 Yes	72	2.89
Total	2,494	100.00

gb006_3__s8: Other Government Subsidy HHMemberName[3] Receive

	Freq.	%
0 No	792	97.18
8 Yes	23	2.82
Total	815	100.00

gb006_4__s8: Other Government Subsidy HHMemberName[4] Receive

	Freq.	%
0 No	339	97.13
8 Yes	10	2.87
Total	349	100.00

gb006_5__s8: Other Government Subsidy HHMemberName[5] Receive

	Freq.	%
0 No	91	95.79
8 Yes	4	4.21
Total	95	100.00

gb006_6__s8: Other Government Subsidy HHMemberName[6] Receive

	Freq.	%
0 No	38	97.44
8 Yes	1	2.56
Total	39	100.00

gb006_7__s8: Other Government Subsidy HHMemberName[7] Receive

	Freq.	%
0 No	11,377	100.00
Total	11,377	100.00

gb006_8__s8: Other Government Subsidy HHMemberName[8] Receive

	Freq.	%
0 No	11,376	99.99
8 Yes	1	0.01
Total	11,377	100.00

gb006_9__s8: Other Government Subsidy HHMemberName[9] Receive

	Freq.	%
0 No	11,377	100.00

	Freq.	%
Total	11,377	100.00

gb006_10__s8: Other Government Subsidy HHMemberName[10] Receive

	Freq.	%
0 No	11,377	100.00
Total	11,377	100.00

gb006_11__s8: Other Government Subsidy HHMemberName[11] Receive

	Freq.	%
0 No	11,377	100.00
Total	11,377	100.00

gb006_12__s8: Other Government Subsidy HHMemberName[12] Receive

	Freq.	%
0 No	11,377	100.00
Total	11,377	100.00

gb006_13__s8: Other Government Subsidy HHMemberName[13] Receive

	Freq.	%
0 No	11,377	100.00
Total	11,377	100.00

gb006_14__s8: Other Government Subsidy HHMemberName[14] Receive

	Freq.	%
0 No	11,377	100.00
Total	11,377	100.00

gb006_1__s9: Other Income Source HHMemberName[1] Receive

	Freq.	%
0 No	4,817	99.14
9 Yes	42	0.86
Total	4,859	100.00

gb006_2__s9: Other Income Source HHMemberName[2] Receive

	Freq.	%
0 No	2,473	99.16
9 Yes	21	0.84
Total	2,494	100.00

gb006_3__s9: Other Income Source HHMemberName[3] Receive

	Freq.	%
0 No	809	99.26
9 Yes	6	0.74
Total	815	100.00

gb006_4__s9: Other Income Source HHMemberName[4] Receive

	Freq.	%
0 No	347	99.43
9 Yes	2	0.57
Total	349	100.00

gb006_5__s9: Other Income Source HHMemberName[5] Receive

	Freq.	%
0 No	95	100.00
Total	95	100.00

gb006_6__s9: Other Income Source HHMemberName[6] Receive

	Freq.	%
0 No	39	100.00
Total	39	100.00

gb006_7__s9: Other Income Source HHMemberName[7] Receive

	Freq.	%
0 No	11,377	100.00
Total	11,377	100.00

gb006_8__s9: Other Income Source HHMemberName[8] Receive

	Freq.	%
0 No	11,377	100.00
Total	11,377	100.00

gb006_9__s9: Other Income Source HHMemberName[9] Receive

	Freq.	%
0 No	11,377	100.00
Total	11,377	100.00

gb006_10__s9: Other Income Source HHMemberName[10] Receive

	Freq.	%
0 No	11,377	100.00
Total	11,377	100.00

gb006_11__s9: Other Income Source HHMemberName[11] Receive

	Freq.	%
0 No	11,377	100.00
Total	11,377	100.00

gb006_12__s9: Other Income Source HHMemberName[12] Receive

	Freq.	%
0 No	11,377	100.00
Total	11,377	100.00

gb006_13__s9: Other Income Source HHMemberName[13] Receive

	Freq.	%
0 No	11,377	100.00
Total	11,377	100.00

gb006_14__s9: Other Income Source HHMemberName[14] Receive

	Freq.	%
0 No	11,377	100.00
Total	11,377	100.00

gb006_1__s10: None of the Above HHMemberName[1] Receive

	Freq.	%
0 No	706	14.53
10 Yes	4,153	85.47
Total	4,859	100.00

gb006_2__s10: None of the Above HHMemberName[2] Receive

	Freq.	%
0 No	311	12.47
10 Yes	2,183	87.53
Total	2,494	100.00

gb006_3__s10: None of the Above HHMemberName[3] Receive

	Freq.	%
0 No	98	12.02
10 Yes	717	87.98
Total	815	100.00

gb006_4__s10: None of the Above HHMemberName[4] Receive

	Freq.	%
0 No	34	9.74
10 Yes	315	90.26
Total	349	100.00

gb006_5__s10: None of the Above HHMemberName[5] Receive

	Freq.	%
0 No	9	9.47
10 Yes	86	90.53
Total	95	100.00

gb006_6__s10: None of the Above HHMemberName[6] Receive

	Freq.	%
0 No	3	7.69
10 Yes	36	92.31
Total	39	100.00

gb006_7__s10: None of the Above HHMemberName[7] Receive

	Freq.	%
0 No	11,359	99.84
10 Yes	18	0.16
Total	11,377	100.00

gb006_8__s10: None of the Above HHMemberName[8] Receive

	Freq.	%
0 No	11,368	99.92
10 Yes	9	0.08
Total	11,377	100.00

gb006_9__s10: None of the Above HHMemberName[9] Receive

	Freq.	%
0 No	11,375	99.98
10 Yes	2	0.02
Total	11,377	100.00

gb006_10__s10: None of the Above HHMemberName[10] Receive

	Freq.	%
0 No	11,372	99.96
10 Yes	5	0.04
Total	11,377	100.00

gb006_11__s10: None of the Above HHMemberName[11] Receive

	Freq.	%
0 No	11,375	99.98
10 Yes	2	0.02
Total	11,377	100.00

gb006_12__s10: None of the Above HHMemberName[12] Receive

	Freq.	%
0 No	11,376	99.99
10 Yes	1	0.01
Total	11,377	100.00

gb006_13__s10: None of the Above HHMemberName[13] Receive

	Freq.	%
0 No	11,376	99.99
10 Yes	1	0.01
Total	11,377	100.00

gb006_14__s10: None of the Above HHMemberName[14] Receive

	Freq.	%
0 No	11,376	99.99
10 Yes	1	0.01
Total	11,377	100.00

gb006_1_1_: Pension Amount HHMemberName[1] Receive

Mean	SD	Min	Max	Obs
10,371.98	17,305.09	-1.00	150,000.00	380

gb006_1_2_: Pension Amount HHMemberName[2] Receive

Mean	SD	Min	Max	Obs
11,314.42	17,217.04	-1.00	108,000.00	180

gb006_1_3_: Pension Amount HHMemberName[3] Receive

Mean	SD	Min	Max	Obs
8,755.68	17,955.51	-1.00	96,000.00	56

gb006_1_4_: Pension Amount HHMemberName[4] Receive

Mean	SD	Min	Max	Obs
4,126.41	6,610.52	-1.00	24,000.00	17

gb006_1_5_: Pension Amount HHMemberName[5] Receive

Mean	SD	Min	Max	Obs
1,422.50	969.76	170.00	2,400.00	4

gb006_1_6_: Pension Amount HHMemberName[6] Receive

Mean	SD	Min	Max	Obs
1,799.50	2,546.29	-1.00	3,600.00	2

gb006_1_7_: Pension Amount HHMemberName[7] Receive

Mean	SD	Min	Max	Obs
26,400.00	.	26,400.00	26,400.00	1

gb006_2_1_: Unemployment Compensation Amount HHMemberName[1] Receive

Mean	SD	Min	Max	Obs
3,089.05	5,210.74	-1.00	20,000.00	38

gb006_2_2_: Unemployment Compensation Amount HHMemberName[2] Receive

Mean	SD	Min	Max	Obs
3,359.31	8,079.45	-1.00	30,000.00	16

gb006_2_3_: Unemployment Compensation Amount HHMemberName[3] Receive

Mean	SD	Min	Max	Obs
-1.00	0.00	-1.00	-1.00	4

gb006_2_4_: Unemployment Compensation Amount HHMemberName[4] Receive

Mean	SD	Min	Max	Obs
-1.00	0.00	-1.00	-1.00	3

gb006_3_1_: Pension Voucher Amount HHMemberName[1] Receive

Mean	SD	Min	Max	Obs
3,693.52	10,622.49	-1.00	46,000.00	25

gb006_3_2_: Pension Voucher Amount HHMemberName[2] Receive

Mean	SD	Min	Max	Obs
882.13	1,299.02	-1.00	3,600.00	15

gb006_3_3_: Pension Voucher Amount HHMemberName[3] Receive

Mean	SD	Min	Max	Obs
599.50	849.24	-1.00	1,200.00	2

gb006_3_4_: Pension Voucher Amount HHMemberName[4] Receive

Mean	SD	Min	Max	Obs
399.33	693.40	-1.00	1,200.00	3

gb006_4_1_: Pension Subsidy for the Oldest Old Amount HHMemberName[1] Receive

Mean	SD	Min	Max	Obs
751.44	809.09	-1.00	4,800.00	113

gb006_4_2_: Pension Subsidy for the Oldest Old Amount HHMemberName[2] Receive

Mean	SD	Min	Max	Obs
748.41	756.36	-1.00	3,600.00	54

gb006_4_3_: Pension Subsidy for the Oldest Old Amount HHMemberName[3] Receive

Mean	SD	Min	Max	Obs
826.86	598.69	-1.00	2,400.00	22

gb006_4_4_: Pension Subsidy for the Oldest Old Amount HHMemberName[4] Receive

Mean	SD	Min	Max	Obs
643.17	452.23	-1.00	1,260.00	12

gb006_4_5_: Pension Subsidy for the Oldest Old Amount HHMemberName[5] Receive

Mean	SD	Min	Max	Obs
599.50	849.24	-1.00	1,200.00	2

gb006_4_6_: Pension Subsidy for the Oldest Old Amount HHMemberName[6] Receive

Mean	SD	Min	Max	Obs
-1.00	.	-1.00	-1.00	1

gb006_5_1_: Workers' Industrial Accident Compensation Amount HHMemberName[1] Receive

Mean	SD	Min	Max	Obs
3,571.52	16,106.77	-1.00	113,422.00	52

gb006_5_2_: Workers' Industrial Accident Compensation Amount HHMemberName[2] Receive

Mean	SD	Min	Max	Obs
2,887.59	6,087.19	-1.00	20,000.00	17

gb006_5_3_: Workers' Industrial Accident Compensation Amount HHMemberName[3] Receive

Mean	SD	Min	Max	Obs
387.86	759.98	-1.00	2,000.00	7

gb006_5_4_: Workers' Industrial Accident Compensation Amount HHMemberName[4] Receive

Mean	SD	Min	Max	Obs
1,899.25	3,800.50	-1.00	7,600.00	4

gb006_6_1_: Elderly Family Planning Subsidy Amount HHMemberName[1] Receive

Mean	SD	Min	Max	Obs
1,018.35	2,759.73	-1.00	15,000.00	46

gb006_6_2_: Elderly Family Planning Subsidy Amount HHMemberName[2] Receive

Mean	SD	Min	Max	Obs
1,162.00	3,140.36	-1.00	15,000.00	22

gb006_6_3_: Elderly Family Planning Subsidy Amount HHMemberName[3] Receive

Mean	SD	Min	Max	Obs
626.33	543.65	-1.00	960.00	3

gb006_6_4_: Elderly Family Planning Subsidy Amount HHMemberName[4] Receive

Mean	SD	Min	Max	Obs
693.00	544.08	-1.00	1,200.00	6

gb006_7_1_: Medical Aid Amount HHMemberName[1] Receive

Mean	SD	Min	Max	Obs
3,991.71	17,775.25	-1.00	140,000.00	63

gb006_7_2_: Medical Aid Amount HHMemberName[2] Receive

Mean	SD	Min	Max	Obs
1,555.61	3,127.61	-1.00	14,000.00	31

gb006_7_3_: Medical Aid Amount HHMemberName[3] Receive

Mean	SD	Min	Max	Obs
1,755.00	3,261.51	-1.00	10,000.00	9

gb006_7_4_: Medical Aid Amount HHMemberName[4] Receive

Mean	SD	Min	Max	Obs
9.33	17.90	-1.00	30.00	3

gb006_7_6_: Medical Aid Amount HHMemberName[6] Receive

Mean	SD	Min	Max	Obs
-1.00	.	-1.00	-1.00	1

gb006_8_1_: Other Government Subsidy Amount HHMemberName[1] Receive

Mean	SD	Min	Max	Obs
2,429.58	4,259.09	-1.00	25,000.00	186

gb006_8_2_: Other Government Subsidy Amount HHMemberName[2] Receive

Mean	SD	Min	Max	Obs
1,962.69	3,851.58	-1.00	25,800.00	72

gb006_8_3_: Other Government Subsidy Amount HHMemberName[3] Receive

Mean	SD	Min	Max	Obs
717.17	1,211.15	-1.00	5,076.00	23

gb006_8_4_: Other Government Subsidy Amount HHMemberName[4] Receive

Mean	SD	Min	Max	Obs
865.60	1,846.38	-1.00	6,000.00	10

gb006_8_5_: Other Government Subsidy Amount HHMemberName[5] Receive

Mean	SD	Min	Max	Obs
770.00	714.33	200.00	1,680.00	4

gb006_8_6_: Other Government Subsidy Amount HHMemberName[6] Receive

Mean	SD	Min	Max	Obs
2,400.00	.	2,400.00	2,400.00	1

gb006_8_8_: Other Government Subsidy Amount HHMemberName[8] Receive

Mean	SD	Min	Max	Obs
2,600.00	.	2,600.00	2,600.00	1

gb006_9_1_: Other Income Source Amount HHMemberName[1] Receive

Mean	SD	Min	Max	Obs
7,586.48	23,176.74	-1.00	139,416.00	42

gb006_9_2_: Other Income Source Amount HHMemberName[2] Receive

Mean	SD	Min	Max	Obs
1,397.29	2,402.83	-1.00	10,000.00	21

gb006_9_3_: Other Income Source Amount HHMemberName[3] Receive

Mean	SD	Min	Max	Obs
499.33	837.14	-1.00	2,000.00	6

gb006_9_4_: Other Income Source Amount HHMemberName[4] Receive

Mean	SD	Min	Max	Obs
-1.00	0.00	-1.00	-1.00	2

gb007_1_: Impact of COVID on Wage for HHMemberName[1]

	Freq.	%
1 Decrease	2,163	44.52
2 Increase	53	1.09
3 Unchange	2,643	54.39
Total	4,859	100.00

gb007_2_: Impact of COVID on Wage for HHMemberName[2]

	Freq.	%
1 Decrease	915	36.69
2 Increase	12	0.48
3 Unchange	1,567	62.83
Total	2,494	100.00

gb007_3_: Impact of COVID on Wage for HHMemberName[3]

	Freq.	%
1 Decrease	215	26.38
2 Increase	1	0.12
3 Unchange	599	73.50
Total	815	100.00

gb007_4_: Impact of COVID on Wage for HHMemberName[4]

	Freq.	%
1 Decrease	114	32.66
3 Unchange	235	67.34
Total	349	100.00

gb007_5_: Impact of COVID on Wage for HHMemberName[5]

	Freq.	%
1 Decrease	36	37.89
3 Unchange	59	62.11
Total	95	100.00

gb007_6_: Impact of COVID on Wage for HHMemberName[6]

	Freq.	%
1 Decrease	9	23.08
2 Increase	1	2.56
3 Unchange	29	74.36
Total	39	100.00

gb007_7_: Impact of COVID on Wage for HHMemberName[7]

	Freq.	%
1 Decrease	4	21.05
3 Unchange	15	78.95
Total	19	100.00

gb007_8_: Impact of COVID on Wage for HHMemberName[8]

	Freq.	%
1 Decrease	2	20.00

	Freq.	%
3 Unchange	8	80.00
Total	10	100.00

gb007_9_: Impact of COVID on Wage for HHMemberName[9]

	Freq.	%
1 Decrease	1	50.00
3 Unchange	1	50.00
Total	2	100.00

gb007_10_: Impact of COVID on Wage for HHMemberName[10]

	Freq.	%
1 Decrease	3	60.00
3 Unchange	2	40.00
Total	5	100.00

gb007_11_: Impact of COVID on Wage for HHMemberName[11]

	Freq.	%
1 Decrease	1	50.00
3 Unchange	1	50.00
Total	2	100.00

gb007_12_: Impact of COVID on Wage for HHMemberName[12]

	Freq.	%
3 Unchange	1	100.00
Total	1	100.00

gb007_13_: Impact of COVID on Wage for HHMemberName[13]

	Freq.	%
3 Unchange	1	100.00
Total	1	100.00

gb007_14_: Impact of COVID on Wage for HHMemberName[14]

	Freq.	%
3 Unchange	1	100.00
Total	1	100.00

gb007_1_1_: Amount Decreased HHMemberName[1] Receive

Mean	SD	Min	Max	Obs
11,780.59	34,885.39	-1.00	1,000,000.00	2,163

gb007_1_2_: Amount Decreased HHMemberName[2] Receive

Mean	SD	Min	Max	Obs
9,358.77	25,827.39	-1.00	700,000.00	915

gb007_1_3_: Amount Decreased HHMemberName[3] Receive

Mean	SD	Min	Max	Obs
8,477.06	12,132.43	-1.00	90,000.00	215

gb007_1_4_: Amount Decreased HHMemberName[4] Receive

Mean	SD	Min	Max	Obs
8,257.66	10,730.37	-1.00	70,000.00	114

gb007_1_5_: Amount Decreased HHMemberName[5] Receive

Mean	SD	Min	Max	Obs
9,833.11	10,546.03	-1.00	50,000.00	36

gb007_1_6_: Amount Decreased HHMemberName[6] Receive

Mean	SD	Min	Max	Obs
8,899.67	9,052.44	-1.00	20,000.00	9

gb007_1_7_: Amount Decreased HHMemberName[7] Receive

Mean	SD	Min	Max	Obs
11,249.75	10,308.13	-1.00	20,000.00	4

gb007_1_8_: Amount Decreased HHMemberName[8] Receive

Mean	SD	Min	Max	Obs
19,000.00	1,414.21	18,000.00	20,000.00	2

gb007_1_9_: Amount Decreased HHMemberName[9] Receive

Mean	SD	Min	Max	Obs
2,600.00	.	2,600.00	2,600.00	1

gb007_1_10_: Amount Decreased HHMemberName[10] Receive

Mean	SD	Min	Max	Obs
5,533.33	3,931.07	2,600.00	10,000.00	3

gb007_1_11_: Amount Decreased HHMemberName[11] Receive

Mean	SD	Min	Max	Obs
3,000.00	.	3,000.00	3,000.00	1

gb007_1_min_1_: Min Bracket of gb007_1_1_

Mean	SD	Min	Max	Obs
3,905.12	5,925.55	-1.00	20,000.00	445

gb007_1_min_2_: Min Bracket of gb007_1_2_

Mean	SD	Min	Max	Obs
2,626.40	5,294.52	-1.00	20,000.00	185

gb007_1_min_3_: Min Bracket of gb007_1_3_

Mean	SD	Min	Max	Obs
3,273.55	6,218.17	-1.00	20,000.00	62

gb007_1_min_4_: Min Bracket of gb007_1_4_

Mean	SD	Min	Max	Obs
1,777.04	4,568.87	-1.00	20,000.00	27

gb007_1_min_5_: Min Bracket of gb007_1_5_

Mean	SD	Min	Max	Obs
1,499.50	1,927.69	-1.00	5,000.00	8

gb007_1_min_6_: Min Bracket of gb007_1_6_

Mean	SD	Min	Max	Obs
999.33	1,732.63	-1.00	3,000.00	3

gb007_1_min_7_: Min Bracket of gb007_1_7_

Mean	SD	Min	Max	Obs
-1.00	.	-1.00	-1.00	1

gb007_1_min_8_: Min Bracket of gb007_1_8_

Mean	SD	Min	Max	Obs
-1.00	.	-1.00	-1.00	1

gb007_1_max_1_: Max Bracket of gb007_1_1_

Mean	SD	Min	Max	Obs
3,802.23	6,034.02	-1.00	20,000.00	431

gb007_1_max_2_: Max Bracket of gb007_1_2_

Mean	SD	Min	Max	Obs
2,638.23	5,104.58	-1.00	20,000.00	180

gb007_1_max_3_: Max Bracket of gb007_1_3_

Mean	SD	Min	Max	Obs
3,102.74	6,086.56	-1.00	20,000.00	58

gb007_1_max_4_: Max Bracket of gb007_1_4_

Mean	SD	Min	Max	Obs
1,999.23	5,440.88	-1.00	20,000.00	26

gb007_1_max_5_: Max Bracket of gb007_1_5_

Mean	SD	Min	Max	Obs
1,374.38	2,264.28	-1.00	5,000.00	8

gb007_1_max_6_: Max Bracket of gb007_1_6_

Mean	SD	Min	Max	Obs
1,666.00	2,887.33	-1.00	5,000.00	3

gb007_1_max_7_: Max Bracket of gb007_1_7_

Mean	SD	Min	Max	Obs
-1.00	.	-1.00	-1.00	1

gb007_1_max_8_: Max Bracket of gb007_1_8_

Mean	SD	Min	Max	Obs
-1.00	.	-1.00	-1.00	1

gb007_2_1_: Amount Increased HHMemberName[1] Receive

Mean	SD	Min	Max	Obs
9,882.38	15,925.61	-1.00	100,000.00	53

gb007_2_2_: Amount Increased HHMemberName[2] Receive

Mean	SD	Min	Max	Obs
6,383.25	7,924.13	-1.00	30,000.00	12

gb007_2_3_: Amount Increased HHMemberName[3] Receive

Mean	SD	Min	Max	Obs
15,000.00	.	15,000.00	15,000.00	1

gb007_2_6_: Amount Increased HHMemberName[6] Receive

Mean	SD	Min	Max	Obs
10,000.00	.	10,000.00	10,000.00	1

gb007_2_min_1_: Min Bracket of gb007_2_1_

Mean	SD	Min	Max	Obs
2,090.18	4,011.76	-1.00	10,000.00	11

gb007_2_min_2_: Min Bracket of gb007_2_2_

Mean	SD	Min	Max	Obs
-1.00	.	-1.00	-1.00	1

gb007_2_max_1_: Max Bracket of gb007_2_1_

Mean	SD	Min	Max	Obs
2,090.18	4,011.76	-1.00	10,000.00	11

gb007_2_max_2_: Max Bracket of gb007_2_2_

Mean	SD	Min	Max	Obs
-1.00	.	-1.00	-1.00	1

gb008_1__s7: Medical Aid HHMemberName[1] Receive

	Freq.	%
0 No	713	99.03
7 Yes	7	0.97
Total	720	100.00

gb008_2__s7: Medical Aid HHMemberName[2] Receive

	Freq.	%
0 No	877	99.89
7 Yes	1	0.11

	Freq.	%
Total	878	100.00

gb008_3__s7: Medical Aid HHMemberName[3] Receive

	Freq.	%
0 No	1,252	99.13
7 Yes	11	0.87
Total	1,263	100.00

gb008_4__s7: Medical Aid HHMemberName[4] Receive

	Freq.	%
0 No	810	99.63
7 Yes	3	0.37
Total	813	100.00

gb008_5__s7: Medical Aid HHMemberName[5] Receive

	Freq.	%
0 No	275	99.64
7 Yes	1	0.36
Total	276	100.00

gb008_6__s7: Medical Aid HHMemberName[6] Receive

	Freq.	%
0 No	11,377	100.00
Total	11,377	100.00

gb008_7__s7: Medical Aid HHMemberName[7] Receive

	Freq.	%
0 No	11,377	100.00
Total	11,377	100.00

gb008_8__s7: Medical Aid HHMemberName[8] Receive

	Freq.	%
0 No	11,377	100.00
Total	11,377	100.00

gb008_9__s7: Medical Aid HHMemberName[9] Receive

	Freq.	%
0 No	11,377	100.00
Total	11,377	100.00

gb008_10__s7: Medical Aid HHMemberName[10] Receive

	Freq.	%
0 No	11,377	100.00
Total	11,377	100.00

gb008_11__s7: Medical Aid HHMemberName[11] Receive

	Freq.	%
0 No	11,377	100.00
Total	11,377	100.00

gb008_12__s7: Medical Aid HHMemberName[12] Receive

	Freq.	%
0 No	11,377	100.00
Total	11,377	100.00

gb008_1__s8: Other Government Subsidy HHMemberName[1] Receive

	Freq.	%
0 No	678	94.17
8 Yes	42	5.83
Total	720	100.00

gb008_2__s8: Other Government Subsidy HHMemberName[2] Receive

	Freq.	%
0 No	829	94.42
8 Yes	49	5.58
Total	878	100.00

gb008_3__s8: Other Government Subsidy HHMemberName[3] Receive

	Freq.	%
0 No	1,208	95.65
8 Yes	55	4.35
Total	1,263	100.00

gb008_4__s8: Other Government Subsidy HHMemberName[4] Receive

	Freq.	%
0 No	792	97.42
8 Yes	21	2.58
Total	813	100.00

gb008_5__s8: Other Government Subsidy HHMemberName[5] Receive

	Freq.	%

	Freq.	%
0 No	272	98.55
8 Yes	4	1.45
Total	276	100.00

gb008_6__s8: Other Government Subsidy HHMemberName[6] Receive

	Freq.	%
0 No	11,374	99.97
8 Yes	3	0.03
Total	11,377	100.00

gb008_7__s8: Other Government Subsidy HHMemberName[7] Receive

	Freq.	%
0 No	11,376	99.99
8 Yes	1	0.01
Total	11,377	100.00

gb008_8__s8: Other Government Subsidy HHMemberName[8] Receive

	Freq.	%
0 No	11,377	100.00
Total	11,377	100.00

gb008_9__s8: Other Government Subsidy HHMemberName[9] Receive

	Freq.	%
0 No	11,377	100.00
Total	11,377	100.00

gb008_10__s8: Other Government Subsidy HHMemberName[10] Receive

	Freq.	%
0 No	11,377	100.00
Total	11,377	100.00

gb008_11__s8: Other Government Subsidy HHMemberName[11] Receive

	Freq.	%
0 No	11,377	100.00
Total	11,377	100.00

gb008_12__s8: Other Government Subsidy HHMemberName[12] Receive

	Freq.	%
0 No	11,377	100.00
Total	11,377	100.00

gb008_1__s9: Other Income Source HHMemberName[1] Receive

	Freq.	%
0 No	708	98.33
9 Yes	12	1.67
Total	720	100.00

gb008_2__s9: Other Income Source HHMemberName[2] Receive

	Freq.	%
0 No	868	98.86
9 Yes	10	1.14
Total	878	100.00

gb008_3__s9: Other Income Source HHMemberName[3] Receive

	Freq.	%
0 No	1,243	98.42
9 Yes	20	1.58
Total	1,263	100.00

gb008_4__s9: Other Income Source HHMemberName[4] Receive

	Freq.	%
0 No	804	98.89
9 Yes	9	1.11
Total	813	100.00

gb008_5__s9: Other Income Source HHMemberName[5] Receive

	Freq.	%
0 No	274	99.28
9 Yes	2	0.72
Total	276	100.00

gb008_6__s9: Other Income Source HHMemberName[6] Receive

	Freq.	%
0 No	11,376	99.99
9 Yes	1	0.01
Total	11,377	100.00

gb008_7__s9: Other Income Source HHMemberName[7] Receive

	Freq.	%
0 No	11,377	100.00
Total	11,377	100.00

gb008_8__s9: Other Income Source HHMemberName[8] Receive

	Freq.	%
0 No	11,377	100.00
Total	11,377	100.00

gb008_9__s9: Other Income Source HHMemberName[9] Receive

	Freq.	%
0 No	11,377	100.00
Total	11,377	100.00

gb008_10__s9: Other Income Source HHMemberName[10] Receive

	Freq.	%
0 No	11,377	100.00
Total	11,377	100.00

gb008_11__s9: Other Income Source HHMemberName[11] Receive

	Freq.	%
0 No	11,377	100.00
Total	11,377	100.00

gb008_12__s9: Other Income Source HHMemberName[12] Receive

	Freq.	%
0 No	11,377	100.00
Total	11,377	100.00

gb008_1__s10: None of the Above HHMemberName[1] Receive

	Freq.	%
0 No	56	7.78
10 Yes	664	92.22
Total	720	100.00

gb008_2__s10: None of the Above HHMemberName[2] Receive

	Freq.	%
0 No	59	6.72
10 Yes	819	93.28
Total	878	100.00

gb008_3__s10: None of the Above HHMemberName[3] Receive

	Freq.	%

	Freq.	%
0 No	75	5.94
10 Yes	1,188	94.06
Total	1,263	100.00

gb008_4__s10: None of the Above HHMemberName[4] Receive

	Freq.	%
0 No	30	3.69
10 Yes	783	96.31
Total	813	100.00

gb008_5__s10: None of the Above HHMemberName[5] Receive

	Freq.	%
0 No	6	2.17
10 Yes	270	97.83
Total	276	100.00

gb008_6__s10: None of the Above HHMemberName[6] Receive

	Freq.	%
0 No	11,262	98.99
10 Yes	115	1.01
Total	11,377	100.00

gb008_7__s10: None of the Above HHMemberName[7] Receive

	Freq.	%
0 No	11,312	99.43
10 Yes	65	0.57
Total	11,377	100.00

gb008_8__s10: None of the Above HHMemberName[8] Receive

	Freq.	%
0 No	11,344	99.71
10 Yes	33	0.29
Total	11,377	100.00

gb008_9__s10: None of the Above HHMemberName[9] Receive

	Freq.	%
0 No	11,357	99.82
10 Yes	20	0.18
Total	11,377	100.00

gb008_10__s10: None of the Above HHMemberName[10] Receive

	Freq.	%
0 No	11,368	99.92
10 Yes	9	0.08
Total	11,377	100.00

gb008_11__s10: None of the Above HHMemberName[11] Receive

	Freq.	%
0 No	11,372	99.96
10 Yes	5	0.04
Total	11,377	100.00

gb008_12__s10: None of the Above HHMemberName[12] Receive

	Freq.	%
0 No	11,375	99.98
10 Yes	2	0.02
Total	11,377	100.00

gb008_7_1_: Medical Aid Amount HHMemberName[1] Receive

Mean	SD	Min	Max	Obs
3,365.57	7,366.51	-1.00	20,000.00	7

gb008_7_2_: Medical Aid Amount HHMemberName[2] Receive

Mean	SD	Min	Max	Obs
10,000.00	.	10,000.00	10,000.00	1

gb008_7_3_: Medical Aid Amount HHMemberName[3] Receive

Mean	SD	Min	Max	Obs
3,093.09	5,820.32	-1.00	18,000.00	11

gb008_7_4_: Medical Aid Amount HHMemberName[4] Receive

Mean	SD	Min	Max	Obs
299.67	360.97	-1.00	700.00	3

gb008_7_5_: Medical Aid Amount HHMemberName[5] Receive

Mean	SD	Min	Max	Obs
-1.00	.	-1.00	-1.00	1

gb008_8_1_: Other Government Subsidy Amount HHMemberName[1] Receive

Mean	SD	Min	Max	Obs
687.52	585.10	-1.00	2,520.00	42

gb008_8_2_: Other Government Subsidy Amount HHMemberName[2] Receive

Mean	SD	Min	Max	Obs
742.69	793.36	-1.00	4,000.00	49

gb008_8_3_: Other Government Subsidy Amount HHMemberName[3] Receive

Mean	SD	Min	Max	Obs
911.09	1,971.90	-1.00	10,000.00	55

gb008_8_4_: Other Government Subsidy Amount HHMemberName[4] Receive

Mean	SD	Min	Max	Obs
504.33	415.61	-1.00	1,400.00	21

gb008_8_5_: Other Government Subsidy Amount HHMemberName[5] Receive

Mean	SD	Min	Max	Obs
512.25	466.51	-1.00	1,000.00	4

gb008_8_6_: Other Government Subsidy Amount HHMemberName[6] Receive

Mean	SD	Min	Max	Obs
316.67	160.73	200.00	500.00	3

gb008_8_7_: Other Government Subsidy Amount HHMemberName[7] Receive

Mean	SD	Min	Max	Obs
500.00	.	500.00	500.00	1

gb008_9_1_: Other Income Source Amount HHMemberName[1] Receive

Mean	SD	Min	Max	Obs
682.50	513.05	150.00	2,000.00	12

gb008_9_2_: Other Income Source Amount HHMemberName[2] Receive

Mean	SD	Min	Max	Obs
572.00	357.08	100.00	1,000.00	10

gb008_9_3_: Other Income Source Amount HHMemberName[3] Receive

Mean	SD	Min	Max	Obs
791.55	1,018.22	-1.00	3,600.00	20

gb008_9_4_: Other Income Source Amount HHMemberName[4] Receive

Mean	SD	Min	Max	Obs
520.44	617.35	-1.00	1,840.00	9

gb008_9_5_: Other Income Source Amount HHMemberName[5] Receive

Mean	SD	Min	Max	Obs
49.50	71.42	-1.00	100.00	2

gb008_9_6_: Other Income Source Amount HHMemberName[6] Receive

Mean	SD	Min	Max	Obs
420.00	.	420.00	420.00	1

gc001: Engage in Agricultural Work

	Freq.	%
1 Yes	5,866	51.62
2 No	5,497	48.38
Total	11,363	100.00

gc002_s1: HHMemberName[1] Engaged in Agricultural Work

	Freq.	%
0 No	4,702	80.16
1 Yes	1,164	19.84
Total	5,866	100.00

gc002_s2: HHMemberName[2] Engaged in Agricultural Work

	Freq.	%
0 No	5,295	90.27
2 Yes	571	9.73
Total	5,866	100.00

gc002_s3: HHMemberName[3] Engaged in Agricultural Work

	Freq.	%
0 No	5,735	97.77
3 Yes	131	2.23
Total	5,866	100.00

gc002_s4: HHMemberName[4] Engaged in Agricultural Work

	Freq.	%
0 No	5,789	98.69
4 Yes	77	1.31
Total	5,866	100.00

gc002_s5: HHMemberName[5] Engaged in Agricultural Work

	Freq.	%
0 No	5,844	99.62
5 Yes	22	0.38
Total	5,866	100.00

gc002_s6: HHMemberName[6] Engaged in Agricultural Work

	Freq.	%
0 No	5,859	99.88
6 Yes	7	0.12
Total	5,866	100.00

gc002_s7: HHMemberName[7] Engaged in Agricultural Work

	Freq.	%
0 No	5,863	99.95
7 Yes	3	0.05
Total	5,866	100.00

gc002_s8: HHMemberName[8] Engaged in Agricultural Work

	Freq.	%
0 No	5,863	99.95
8 Yes	3	0.05
Total	5,866	100.00

gc002_s9: HHMemberName[9] Engaged in Agricultural Work

	Freq.	%
0 No	5,865	99.98
9 Yes	1	0.02
Total	5,866	100.00

gc002_s10: HHMemberName[10] Engaged in Agricultural Work

	Freq.	%
0 No	5,865	99.98
10 Yes	1	0.02
Total	5,866	100.00

gc002_s11: HHMemberName[11] Engaged in Agricultural Work

	Freq.	%
0 No	5,866	100.00
Total	5,866	100.00

gc002_s12: HHMemberName[12] Engaged in Agricultural Work

	Freq.	%
0 No	5,866	100.00
Total	5,866	100.00

gc002_s13: HHMemberName[13] Engaged in Agricultural Work

	Freq.	%
0 No	5,866	100.00
Total	5,866	100.00

gc002_s14: HHMemberName[14] Engaged in Agricultural Work

	Freq.	%
0 No	5,866	100.00
Total	5,866	100.00

gc002_s15: HHMemberName[15] Engaged in Agricultural Work

	Freq.	%
0 No	5,866	100.00
Total	5,866	100.00

gc002_s16: HHMemberName[16] Engaged in Agricultural Work

	Freq.	%
0 No	5,866	100.00
Total	5,866	100.00

gc002_s17: HHMemberName[17] Engaged in Agricultural Work

	Freq.	%
0 No	5,866	100.00
Total	5,866	100.00

gc002_s18: HHMemberName[18] Engaged in Agricultural Work

	Freq.	%
0 No	5,866	100.00
Total	5,866	100.00

gc002_s19: HHMemberName[19] Engaged in Agricultural Work

	Freq.	%
0 No	5,866	100.00
Total	5,866	100.00

gc002_s20: HHMemberName[20] Engaged in Agricultural Work

	Freq.	%
0 No	5,866	100.00
Total	5,866	100.00

gc002_s21: HHMemberName[21] Engaged in Agricultural Work

	Freq.	%
0 No	5,866	100.00
Total	5,866	100.00

gc002_s22: HHMemberName[22] Engaged in Agricultural Work

	Freq.	%
0 No	5,866	100.00
Total	5,866	100.00

gc002_s23: HHMemberName[23] Engaged in Agricultural Work

	Freq.	%
0 No	5,866	100.00
Total	5,866	100.00

gc002_s24: HHMemberName[24] Engaged in Agricultural Work

	Freq.	%
0 No	5,866	100.00
Total	5,866	100.00

gc002_s25: HHMemberName[25] Engaged in Agricultural Work

	Freq.	%
0 No	5,866	100.00
Total	5,866	100.00

gc002_s26: Main Respondent Engage in Agricultural Work

	Freq.	%
0 No	1,011	17.23
26 Yes	4,855	82.77

	Freq.	%
Total	5,866	100.00

gc002_s27: Main Respondent's Spouse Engage in Agricultural Work

	Freq.	%
0 No	2,039	34.76
27 Yes	3,827	65.24
Total	5,866	100.00

gc003: Engage in Cropping or Forestry

	Freq.	%
1 Yes	5,152	87.84
2 No	713	12.16
Total	5,865	100.00

gc004: Earn or Lose Money in Cropping or Forestry

	Freq.	%
1 Earn Money	2,743	53.24
2 Lose Money	802	15.57
3 Break-even	1,607	31.19
Total	5,152	100.00

gc004_1: Money Earned in Cropping or Forestry

Mean	SD	Min	Max	Obs
7,074.21	13,729.23	-1.00	300,000.00	2,743

gc004_1_min: Min Bracket of gc004_1

Mean	SD	Min	Max	Obs
5,340.41	15,756.86	-1.00	100,000.00	88

gc004_1_max: Max Bracket of gc004_1

Mean	SD	Min	Max	Obs
4,625.59	11,418.19	-1.00	100,000.00	147

gc004_2: Money Lost in Cropping or Forestry

Mean	SD	Min	Max	Obs
6,347.69	20,900.76	-1.00	350,000.00	802

gc004_2_min: Min Bracket of gc004_2

Mean	SD	Min	Max	Obs

5,332.91	13,736.22	-1.00	100,000.00	54

gc004_2_max: Max Bracket of gc004_2

Mean	SD	Min	Max	Obs
6,307.40	13,028.89	-1.00	50,000.00	78

gc005: Grow Livestock or Aquatic Life

	Freq.	%
1 Yes	3,046	51.94
2 No	2,819	48.06
Total	5,865	100.00

gc006: Earn or Lose Money in Livestock or Aquatic Life

	Freq.	%
1 Earn Money	1,252	41.10
2 Lose Money	668	21.93
3 Break-even	1,126	36.97
Total	3,046	100.00

gc006_1: Money Earned in Livestock or Aquatic Life

Mean	SD	Min	Max	Obs
8,517.85	20,675.99	-1.00	300,000.00	1,252

gc006_1_min: Min Bracket of gc006_1

Mean	SD	Min	Max	Obs
7,772.27	18,977.37	-1.00	100,000.00	44

gc006_1_max: Max Bracket of gc006_1

Mean	SD	Min	Max	Obs
10,397.05	22,952.79	-1.00	100,000.00	73

gc006_2: Money Lost in Livestock or Aquatic Life

Mean	SD	Min	Max	Obs
11,815.75	52,808.22	-1.00	800,000.00	668

gc006_2_min: Min Bracket of gc006_2

Mean	SD	Min	Max	Obs
8,399.65	22,718.93	-1.00	100,000.00	40

gc006_2_max: Max Bracket of gc006_2

Mean	SD	Min	Max	Obs
5,999.86	14,847.67	-1.00	100,000.00	63

gc007: Impact of COVID on Agricultural Income

	Freq.	%
1 Decrease	1,767	15.55
2 Increase	43	0.38
3 Unchange	9,551	84.07
Total	11,361	100.00

gc007_1: Amount Decreased

Mean	SD	Min	Max	Obs
8,362.13	36,869.73	-1.00	1,000,000.00	1,767

gc007_1_min: Min Bracket of gc007_1

Mean	SD	Min	Max	Obs
2,248.94	3,414.13	-1.00	10,000.00	191

gc007_1_max: Max Bracket of gc007_1

Mean	SD	Min	Max	Obs
2,335.73	3,324.46	-1.00	10,000.00	183

gc007_2: Amount Increased

Mean	SD	Min	Max	Obs
7,106.02	12,129.92	-1.00	60,000.00	43

gc007_2_min: Min Bracket of gc007_2

Mean	SD	Min	Max	Obs
1,733.00	2,831.09	-1.00	5,000.00	3

gc007_2_max: Max Bracket of gc007_2

Mean	SD	Min	Max	Obs
1,833.00	2,754.12	-1.00	5,000.00	3

gd001: Engage in Self-employed Activities

	Freq.	%

	Freq.	%
1 Yes	1,285	11.31
2 No	10,075	88.69
Total	11,360	100.00

gd002: Number of Self-employed Activities

Mean	SD	Min	Max	Obs
1.09	0.30	1.00	3.00	1,285

gd003_s1: HHMemberName[1] Engage in Self-employed Activities

	Freq.	%
0 No	802	62.41
1 Yes	483	37.59
Total	1,285	100.00

gd003_s2: HHMemberName[2] Engage in Self-employed Activities

	Freq.	%
0 No	1,056	82.18
2 Yes	229	17.82
Total	1,285	100.00

gd003_s3: HHMemberName[3] Engage in Self-employed Activities

	Freq.	%
0 No	1,226	95.41
3 Yes	59	4.59
Total	1,285	100.00

gd003_s4: HHMemberName[4] Engage in Self-employed Activities

	Freq.	%
0 No	1,263	98.29
4 Yes	22	1.71
Total	1,285	100.00

gd003_s5: HHMemberName[5] Engage in Self-employed Activities

	Freq.	%
0 No	1,279	99.53
5 Yes	6	0.47
Total	1,285	100.00

gd003_s6: HHMemberName[6] Engage in Self-employed Activities

	Freq.	%
0 No	1,285	100.00

Total	1,285	100.00

gd003_s7: HHMemberName[7] Engage in Self-employed Activities

	Freq.	%
0 No	1,284	99.92
7 Yes	1	0.08
Total	1,285	100.00

gd003_s8: HHMemberName[8] Engage in Self-employed Activities

	Freq.	%
0 No	1,284	99.92
8 Yes	1	0.08
Total	1,285	100.00

gd003_s9: HHMemberName[9] Engage in Self-employed Activities

	Freq.	%
0 No	1,285	100.00
Total	1,285	100.00

gd003_s10: HHMemberName[10] Engage in Self-employed Activities

	Freq.	%
0 No	1,285	100.00
Total	1,285	100.00

gd003_s11: HHMemberName[11] Engage in Self-employed Activities

	Freq.	%
0 No	1,285	100.00
Total	1,285	100.00

gd003_s12: HHMemberName[12] Engage in Self-employed Activities

	Freq.	%
0 No	1,285	100.00
Total	1,285	100.00

gd003_s13: HHMemberName[13] Engage in Self-employed Activities

	Freq.	%
0 No	1,285	100.00
Total	1,285	100.00

gd003_s14: HHMemberName[14] Engage in Self-employed Activities

	Freq.	%
0 No	1,285	100.00
Total	1,285	100.00

gd003_s15: HHMemberName[15] Engage in Self-employed Activities

	Freq.	%
0 No	1,285	100.00
Total	1,285	100.00

gd003_s16: HHMemberName[16] Engage in Self-employed Activities

	Freq.	%
0 No	1,285	100.00
Total	1,285	100.00

gd003_s17: HHMemberName[17] Engage in Self-employed Activities

	Freq.	%
0 No	1,285	100.00
Total	1,285	100.00

gd003_s18: HHMemberName[18] Engage in Self-employed Activities

	Freq.	%
0 No	1,285	100.00
Total	1,285	100.00

gd003_s19: HHMemberName[19] Engage in Self-employed Activities

	Freq.	%
0 No	1,285	100.00
Total	1,285	100.00

gd003_s20: HHMemberName[20] Engage in Self-employed Activities

	Freq.	%
0 No	1,285	100.00
Total	1,285	100.00

gd003_s21: HHMemberName[21] Engage in Self-employed Activities

	Freq.	%
0 No	1,285	100.00
Total	1,285	100.00

gd003_s22: HHMemberName[22] Engage in Self-employed Activities

	Freq.	%
0 No	1,285	100.00
Total	1,285	100.00

gd003_s23: HHMemberName[23] Engage in Self-employed Activities

	Freq.	%
0 No	1,285	100.00
Total	1,285	100.00

gd003_s24: HHMemberName[24] Engage in Self-employed Activities

	Freq.	%
0 No	1,285	100.00
Total	1,285	100.00

gd003_s25: HHMemberName[25] Engage in Self-employed Activities

	Freq.	%
0 No	1,285	100.00
Total	1,285	100.00

gd003_s26: Main Respondent Engage in Self-employed Activities

	Freq.	%
0 No	571	44.44
26 Yes	714	55.56
Total	1,285	100.00

gd003_s27: Main Respondent's Spouse Engage in Self-employed Activities

	Freq.	%
0 No	681	53.00
27 Yes	604	47.00
Total	1,285	100.00

gd004: Earn or Lose Money in Self-employed Activities

	Freq.	%
1 Earn Money	881	68.56
2 Lose Money	204	15.88
3 Break-even	169	13.15
999 Refused to Answer	31	2.41
Total	1,285	100.00

gd004_1: Money Earned in Self-employed Activities

Mean	SD	Min	Max	Obs
40,650.61	77,759.78	-1.00	1,000,000.00	881

gd004_1_min: Min Bracket of gd004_1

Mean	SD	Min	Max	Obs
18,937.14	37,185.10	-1.00	200,000.00	80

gd004_1_max: Max Bracket of gd004_1

Mean	SD	Min	Max	Obs
28,218.03	42,447.04	-1.00	200,000.00	87

gd004_2: Money Lost in Self-employed Activities

Mean	SD	Min	Max	Obs
74,122.74	208,906.71	-1.00	2,000,000.00	204

gd004_2_min: Min Bracket of gd004_2

Mean	SD	Min	Max	Obs
47,580.19	78,643.69	-1.00	200,000.00	31

gd004_2_max: Max Bracket of gd004_2

Mean	SD	Min	Max	Obs
38,928.14	57,869.20	-1.00	200,000.00	28

gd005: Impact of COVID on Incomes from Self-employed Activities

	Freq.	%
1 Decrease	1,310	11.53
2 Increase	17	0.15
3 Unchange	10,033	88.32
Total	11,360	100.00

gd005_1: Amount Decreased

Mean	SD	Min	Max	Obs
21,242.72	47,732.84	-1.00	700,000.00	1,310

gd005_1_min: Min Bracket of gd005_1

Mean	SD	Min	Max	Obs
6,585.66	8,022.44	-1.00	20,000.00	186

gd005_1_max: Max Bracket of gd005_1

Mean	SD	Min	Max	Obs
4,593.27	6,700.13	-1.00	20,000.00	160

gd005_2: Amount Increased

Mean	SD	Min	Max	Obs
15,294.00	17,563.44	-1.00	60,000.00	17

gd005_2_min: Min Bracket of gd005_2

Mean	SD	Min	Max	Obs
5,250.00	6,717.51	500.00	10,000.00	2

gd005_2_max: Max Bracket of gd005_2

Mean	SD	Min	Max	Obs
5,250.00	6,717.51	500.00	10,000.00	2

gd006: Impact of COVID on Self-employed Activities

	Freq.	%
1 Do not Have before and after COVID	10,004	88.07
2 Have before and after COVID	1,139	10.03
3 Do not Have before COVID and Have after	63	0.55
4 Have before COVID and Do not Have after	153	1.35
Total	11,359	100.00

gd007_s1: Government Load

	Freq.	%
0 No	1,167	97.09
1 Yes	35	2.91
Total	1,202	100.00

gd007_s2: Rent Relief

	Freq.	%
0 No	1,149	95.59
2 Yes	53	4.41
Total	1,202	100.00

gd007_s3: Tax Deduction

	Freq.	%
0 No	1,152	95.84

	Freq.	%
3 Yes	50	4.16
Total	1,202	100.00

gd007_s4: Other Subsidies

	Freq.	%
0 No	1,187	98.75
4 Yes	15	1.25
Total	1,202	100.00

gd007_s5: None of Above

	Freq.	%
0 No	135	11.23
5 Yes	1,067	88.77
Total	1,202	100.00

gd007_1: Amount of Government Load

Mean	SD	Min	Max	Obs
146,742.57	340,533.22	-1.00	2,000,000.00	35

gd007_2: Interest Rate of Government Load(%)

Mean	SD	Min	Max	Obs
0.55	2.20	-1.00	6.80	35

gd007_3: Amount of Rent Relief

Mean	SD	Min	Max	Obs
10,046.47	18,051.26	-1.00	105,000.00	53

gd007_4: Amount of Tax Deduction

Mean	SD	Min	Max	Obs
2,925.16	8,642.83	-1.00	50,000.00	50

gd007_6: Amount of Other Subsidies

Mean	SD	Min	Max	Obs
4,223.60	12,782.45	-1.00	50,000.00	15

ge008: Government COVID Subsidy for Household Member and Household

	Freq.	%
1 Yes	311	2.74
2 No	11,048	97.26

	Freq.	%
Total	11,359	100.00

ge008_1: Amount of Government COVID Subsidy

Mean	SD	Min	Max	Obs
464.49	1,312.20	-1.00	13,000.00	311

ge008_1_min: Min Bracket of ge008_1

Mean	SD	Min	Max	Obs
249.14	957.66	-1.00	5,000.00	28

ge008_1_max: Max Bracket of ge008_1

Mean	SD	Min	Max	Obs
471.56	1,664.72	-1.00	10,000.00	36

ge001_s1: Wubao Household/Older

	Freq.	%
0 No	11,231	98.87
1 Yes	128	1.13
Total	11,359	100.00

ge001_s2: Dibao Household/Older

	Freq.	%
0 No	10,332	90.96
2 Yes	1,027	9.04
Total	11,359	100.00

ge001_s3: Tekun Household/Older

	Freq.	%
0 No	11,293	99.42
3 Yes	66	0.58
Total	11,359	100.00

ge001_s4: Jiandanglika Poor Household

	Freq.	%
0 No	10,577	93.12
4 Yes	782	6.88
Total	11,359	100.00

ge001_s5: Other Poor Household

	Freq.	%
0 No	11,253	99.07
5 Yes	106	0.93
Total	11,359	100.00

ge001_s6: None of Above

	Freq.	%
0 No	1,830	16.11
6 Yes	9,529	83.89
Total	11,359	100.00

ge002_1_: Now/Used to be Wubao

	Freq.	%
1 Now	119	92.97
2 Used to be	9	7.03
Total	128	100.00

ge002_2_: Now/Used to be Dibao

	Freq.	%
1 Now	818	79.65
2 Used to be	209	20.35
Total	1,027	100.00

ge002_3_: Now/Used to be Tekun

	Freq.	%
1 Now	54	81.82
2 Used to be	12	18.18
Total	66	100.00

ge002_4_: Now/Used to be Jiandanglika Poor Household

	Freq.	%
1 Now	591	75.58
2 Used to be	191	24.42
Total	782	100.00

ge002_5_: Now/Used to be Other Poor Household

	Freq.	%
1 Now	85	80.19
2 Used to be	21	19.81
Total	106	100.00

ge002_1_1_: Begin Time

Mean	SD	Min	Max	Obs
2,011.91	7.14	1,981.00	2,020.00	119

ge002_1_2_: Begin Time

Mean	SD	Min	Max	Obs
2,014.36	5.38	1,985.00	2,020.00	818

ge002_1_3_: Begin Time

Mean	SD	Min	Max	Obs
2,013.59	6.35	1,990.00	2,020.00	54

ge002_1_4_: Begin Time

Mean	SD	Min	Max	Obs
2,015.50	3.73	1,981.00	2,020.00	591

ge002_1_5_: Begin Time

Mean	SD	Min	Max	Obs
2,015.54	3.90	2,000.00	2,020.00	85

ge002_2_1_: Begin Time

Mean	SD	Min	Max	Obs
2,007.78	10.64	1,985.00	2,017.00	9

ge002_2_2_: Begin Time

Mean	SD	Min	Max	Obs
2,010.32	6.17	1,988.00	2,019.00	209

ge002_2_3_: Begin Time

Mean	SD	Min	Max	Obs
2,013.33	5.26	2,000.00	2,018.00	12

ge002_2_4_: Begin Time

Mean	SD	Min	Max	Obs
2,014.68	3.17	1,993.00	2,020.00	191

ge002_2_5_: Begin Time

Mean	SD	Min	Max	Obs
2,011.67	7.15	1,999.00	2,019.00	21

ge015_1_: End Time

Mean	SD	Min	Max	Obs
2,015.11	6.51	1,998.00	2,018.00	9

ge015_2_: End Time

Mean	SD	Min	Max	Obs
2,015.47	4.59	1,995.00	2,020.00	209

ge015_3_: End Time

Mean	SD	Min	Max	Obs
2,017.83	1.75	2,014.00	2,019.00	12

ge015_4_: End Time

Mean	SD	Min	Max	Obs
2,017.87	2.63	1,995.00	2,020.00	191

ge015_5_: End Time

Mean	SD	Min	Max	Obs
2,014.62	7.38	2,000.00	2,020.00	21

ge003_1__s1: HHMemberName[1] Receive Wubao Subsidies

	Freq.	%
0 No	98	82.35
1 Yes	21	17.65
Total	119	100.00

ge003_1__s2: HHMemberName[2] Receive Wubao Subsidies

	Freq.	%
0 No	109	91.60
2 Yes	10	8.40
Total	119	100.00

ge003_1__s3: HHMemberName[3] Receive Wubao Subsidies

	Freq.	%

	Freq.	%
0 No	118	99.16
3 Yes	1	0.84
Total	119	100.00

ge003_1__s4: HHMemberName[4] Receive Wubao Subsidies

	Freq.	%
0 No	118	99.16
4 Yes	1	0.84
Total	119	100.00

ge003_1__s5: HHMemberName[5] Receive Wubao Subsidies

	Freq.	%
0 No	119	100.00
Total	119	100.00

ge003_1__s6: HHMemberName[6] Receive Wubao Subsidies

	Freq.	%
0 No	119	100.00
Total	119	100.00

ge003_1__s7: HHMemberName[7] Receive Wubao Subsidies

	Freq.	%
0 No	119	100.00
Total	119	100.00

ge003_1__s8: HHMemberName[8] Receive Wubao Subsidies

	Freq.	%
0 No	119	100.00
Total	119	100.00

ge003_1__s9: HHMemberName[9] Receive Wubao Subsidies

	Freq.	%
0 No	119	100.00
Total	119	100.00

ge003_1__s10: HHMemberName[10] Receive Wubao Subsidies

	Freq.	%
0 No	119	100.00
Total	119	100.00

ge003_1__s11: HHMemberName[11] Receive Wubao Subsidies

	Freq.	%
0 No	119	100.00
Total	119	100.00

ge003_1__s12: HHMemberName[12] Receive Wubao Subsidies

	Freq.	%
0 No	119	100.00
Total	119	100.00

ge003_1__s13: HHMemberName[13] Receive Wubao Subsidies

	Freq.	%
0 No	119	100.00
Total	119	100.00

ge003_1__s14: HHMemberName[14] Receive Wubao Subsidies

	Freq.	%
0 No	119	100.00
Total	119	100.00

ge003_1__s15: HHMemberName[15] Receive Wubao Subsidies

	Freq.	%
0 No	119	100.00
Total	119	100.00

ge003_1__s16: HHMemberName[16] Receive Wubao Subsidies

	Freq.	%
0 No	119	100.00
Total	119	100.00

ge003_1__s17: HHMemberName[17] Receive Wubao Subsidies

	Freq.	%
0 No	119	100.00
Total	119	100.00

ge003_1__s18: HHMemberName[18] Receive Wubao Subsidies

	Freq.	%
0 No	119	100.00
Total	119	100.00

ge003_1__s19: HHMemberName[19] Receive Wubao Subsidies

	Freq.	%
0 No	119	100.00
Total	119	100.00

ge003_1__s20: HHMemberName[20] Receive Wubao Subsidies

	Freq.	%
0 No	119	100.00
Total	119	100.00

ge003_1__s21: HHMemberName[21] Receive Wubao Subsidies

	Freq.	%
0 No	119	100.00
Total	119	100.00

ge003_1__s22: HHMemberName[22] Receive Wubao Subsidies

	Freq.	%
0 No	119	100.00
Total	119	100.00

ge003_1__s23: HHMemberName[23] Receive Wubao Subsidies

	Freq.	%
0 No	119	100.00
Total	119	100.00

ge003_1__s24: HHMemberName[24] Receive Wubao Subsidies

	Freq.	%
0 No	119	100.00
Total	119	100.00

ge003_1__s25: HHMemberName[25] Receive Wubao Subsidies

	Freq.	%
0 No	119	100.00
Total	119	100.00

ge003_1__s26: Main Respondent Receive Wubao Subsidies

	Freq.	%
0 No	34	28.57
26 Yes	85	71.43

	Freq.	%
Total	119	100.00

ge003_1__s27: Main Respondent's Spouse Receive Wubao Subsidies

	Freq.	%
0 No	115	96.64
27 Yes	4	3.36
Total	119	100.00

ge003_2__s1: HHMemberName[1] Receive Dibao Subsidies

	Freq.	%
0 No	642	76.61
1 Yes	196	23.39
Total	838	100.00

ge003_2__s2: HHMemberName[2] Receive Dibao Subsidies

	Freq.	%
0 No	770	91.89
2 Yes	68	8.11
Total	838	100.00

ge003_2__s3: HHMemberName[3] Receive Dibao Subsidies

	Freq.	%
0 No	806	96.18
3 Yes	32	3.82
Total	838	100.00

ge003_2__s4: HHMemberName[4] Receive Dibao Subsidies

	Freq.	%
0 No	826	98.57
4 Yes	12	1.43
Total	838	100.00

ge003_2__s5: HHMemberName[5] Receive Dibao Subsidies

	Freq.	%
0 No	837	99.88
5 Yes	1	0.12
Total	838	100.00

ge003_2__s6: HHMemberName[6] Receive Dibao Subsidies

	Freq.	%
0 No	837	99.88

6 Yes	1	0.12
Total	838	100.00

ge003_2__s7: HHMemberName[7] Receive Dibao Subsidies

	Freq.	%
0 No	838	100.00
Total	838	100.00

ge003_2__s8: HHMemberName[8] Receive Dibao Subsidies

	Freq.	%
0 No	838	100.00
Total	838	100.00

ge003_2__s9: HHMemberName[9] Receive Dibao Subsidies

	Freq.	%
0 No	838	100.00
Total	838	100.00

ge003_2__s10: HHMemberName[10] Receive Dibao Subsidies

	Freq.	%
0 No	838	100.00
Total	838	100.00

ge003_2__s11: HHMemberName[11] Receive Dibao Subsidies

	Freq.	%
0 No	838	100.00
Total	838	100.00

ge003_2__s12: HHMemberName[12] Receive Dibao Subsidies

	Freq.	%
0 No	838	100.00
Total	838	100.00

ge003_2__s13: HHMemberName[13] Receive Dibao Subsidies

	Freq.	%
0 No	838	100.00
Total	838	100.00

ge003_2__s14: HHMemberName[14] Receive Dibao Subsidies

	Freq.	%
0 No	838	100.00
Total	838	100.00

ge003_2__s15: HHMemberName[15] Receive Dibao Subsidies

	Freq.	%
0 No	838	100.00
Total	838	100.00

ge003_2__s16: HHMemberName[16] Receive Dibao Subsidies

	Freq.	%
0 No	838	100.00
Total	838	100.00

ge003_2__s17: HHMemberName[17] Receive Dibao Subsidies

	Freq.	%
0 No	838	100.00
Total	838	100.00

ge003_2__s18: HHMemberName[18] Receive Dibao Subsidies

	Freq.	%
0 No	838	100.00
Total	838	100.00

ge003_2__s19: HHMemberName[19] Receive Dibao Subsidies

	Freq.	%
0 No	838	100.00
Total	838	100.00

ge003_2__s20: HHMemberName[20] Receive Dibao Subsidies

	Freq.	%
0 No	838	100.00
Total	838	100.00

ge003_2__s21: HHMemberName[21] Receive Dibao Subsidies

	Freq.	%
0 No	838	100.00
Total	838	100.00

ge003_2__s22: HHMemberName[22] Receive Dibao Subsidies

	Freq.	%
0 No	838	100.00
Total	838	100.00

ge003_2__s23: HHMemberName[23] Receive Dibao Subsidies

	Freq.	%
0 No	838	100.00
Total	838	100.00

ge003_2__s24: HHMemberName[24] Receive Dibao Subsidies

	Freq.	%
0 No	838	100.00
Total	838	100.00

ge003_2__s25: HHMemberName[25] Receive Dibao Subsidies

	Freq.	%
0 No	838	100.00
Total	838	100.00

ge003_2__s26: Main Respondent Receive Dibao Subsidies

	Freq.	%
0 No	264	31.50
26 Yes	574	68.50
Total	838	100.00

ge003_2__s27: Main Respondent's Spouse Receive Dibao Subsidies

	Freq.	%
0 No	548	65.39
27 Yes	290	34.61
Total	838	100.00

ge003_3__s1: HHMemberName[1] Receive Tekun Subsidies

	Freq.	%
0 No	40	74.07
1 Yes	14	25.93
Total	54	100.00

ge003_3__s2: HHMemberName[2] Receive Tekun Subsidies

	Freq.	%
0 No	48	88.89
2 Yes	6	11.11
Total	54	100.00

ge003_3__s3: HHMemberName[3] Receive Tekun Subsidies

	Freq.	%
0 No	52	96.30
3 Yes	2	3.70
Total	54	100.00

ge003_3__s4: HHMemberName[4] Receive Tekun Subsidies

	Freq.	%
0 No	53	98.15
4 Yes	1	1.85
Total	54	100.00

ge003_3__s5: HHMemberName[5] Receive Tekun Subsidies

	Freq.	%
0 No	54	100.00
Total	54	100.00

ge003_3__s6: HHMemberName[6] Receive Tekun Subsidies

	Freq.	%
0 No	54	100.00
Total	54	100.00

ge003_3__s7: HHMemberName[7] Receive Tekun Subsidies

	Freq.	%
0 No	54	100.00
Total	54	100.00

ge003_3__s8: HHMemberName[8] Receive Tekun Subsidies

	Freq.	%
0 No	54	100.00
Total	54	100.00

ge003_3__s9: HHMemberName[9] Receive Tekun Subsidies

	Freq.	%
0 No	54	100.00

Total	54	100.00

ge003_3__s10: HHMemberName[10] Receive Tekun Subsidies

	Freq.	%
0 No	54	100.00
Total	54	100.00

ge003_3__s11: HHMemberName[11] Receive Tekun Subsidies

	Freq.	%
0 No	54	100.00
Total	54	100.00

ge003_3__s12: HHMemberName[12] Receive Tekun Subsidies

	Freq.	%
0 No	54	100.00
Total	54	100.00

ge003_3__s13: HHMemberName[13] Receive Tekun Subsidies

	Freq.	%
0 No	54	100.00
Total	54	100.00

ge003_3__s14: HHMemberName[14] Receive Tekun Subsidies

	Freq.	%
0 No	54	100.00
Total	54	100.00

ge003_3__s15: HHMemberName[15] Receive Tekun Subsidies

	Freq.	%
0 No	54	100.00
Total	54	100.00

ge003_3__s16: HHMemberName[16] Receive Tekun Subsidies

	Freq.	%
0 No	54	100.00
Total	54	100.00

ge003_3__s17: HHMemberName[17] Receive Tekun Subsidies

	Freq.	%

	Freq.	%
0 No	54	100.00
Total	54	100.00

ge003_3__s18: HHMemberName[18] Receive Tekun Subsidies

	Freq.	%
0 No	54	100.00
Total	54	100.00

ge003_3__s19: HHMemberName[19] Receive Tekun Subsidies

	Freq.	%
0 No	54	100.00
Total	54	100.00

ge003_3__s20: HHMemberName[20] Receive Tekun Subsidies

	Freq.	%
0 No	54	100.00
Total	54	100.00

ge003_3__s21: HHMemberName[21] Receive Tekun Subsidies

	Freq.	%
0 No	54	100.00
Total	54	100.00

ge003_3__s22: HHMemberName[22] Receive Tekun Subsidies

	Freq.	%
0 No	54	100.00
Total	54	100.00

ge003_3__s23: HHMemberName[23] Receive Tekun Subsidies

	Freq.	%
0 No	54	100.00
Total	54	100.00

ge003_3__s24: HHMemberName[24] Receive Tekun Subsidies

	Freq.	%
0 No	54	100.00
Total	54	100.00

ge003_3__s25: HHMemberName[25] Receive Tekun Subsidies

	Freq.	%
0 No	54	100.00
Total	54	100.00

ge003_3__s26: Main Respondent Receive Tekun Subsidies

	Freq.	%
0 No	12	22.22
26 Yes	42	77.78
Total	54	100.00

ge003_3__s27: Main Respondent's Spouse Receive Tekun Subsidies

	Freq.	%
0 No	33	61.11
27 Yes	21	38.89
Total	54	100.00

ge003_4__s1: HHMemberName[1] Receive Jiandanglika Subsidies

	Freq.	%
0 No	434	68.03
1 Yes	204	31.97
Total	638	100.00

ge003_4__s2: HHMemberName[2] Receive Jiandanglika Subsidies

	Freq.	%
0 No	530	83.07
2 Yes	108	16.93
Total	638	100.00

ge003_4__s3: HHMemberName[3] Receive Jiandanglika Subsidies

	Freq.	%
0 No	574	89.97
3 Yes	64	10.03
Total	638	100.00

ge003_4__s4: HHMemberName[4] Receive Jiandanglika Subsidies

	Freq.	%
0 No	604	94.67
4 Yes	34	5.33
Total	638	100.00

ge003_4__s5: HHMemberName[5] Receive Jiandanglika Subsidies

	Freq.	%
0 No	630	98.75
5 Yes	8	1.25
Total	638	100.00

ge003_4__s6: HHMemberName[6] Receive Jiandanglika Subsidies

	Freq.	%
0 No	635	99.53
6 Yes	3	0.47
Total	638	100.00

ge003_4__s7: HHMemberName[7] Receive Jiandanglika Subsidies

	Freq.	%
0 No	636	99.69
7 Yes	2	0.31
Total	638	100.00

ge003_4__s8: HHMemberName[8] Receive Jiandanglika Subsidies

	Freq.	%
0 No	637	99.84
8 Yes	1	0.16
Total	638	100.00

ge003_4__s9: HHMemberName[9] Receive Jiandanglika Subsidies

	Freq.	%
0 No	638	100.00
Total	638	100.00

ge003_4__s10: HHMemberName[10] Receive Jiandanglika Subsidies

	Freq.	%
0 No	638	100.00
Total	638	100.00

ge003_4__s11: HHMemberName[11] Receive Jiandanglika Subsidies

	Freq.	%
0 No	638	100.00
Total	638	100.00

ge003_4__s12: HHMemberName[12] Receive Jiandanglika Subsidies

	Freq.	%

	Freq.	%
0 No	638	100.00
Total	638	100.00

ge003_4__s13: HHMemberName[13] Receive Jiandanglika Subsidies

	Freq.	%
0 No	638	100.00
Total	638	100.00

ge003_4__s14: HHMemberName[14] Receive Jiandanglika Subsidies

	Freq.	%
0 No	638	100.00
Total	638	100.00

ge003_4__s15: HHMemberName[15] Receive Jiandanglika Subsidies

	Freq.	%
0 No	638	100.00
Total	638	100.00

ge003_4__s16: HHMemberName[16] Receive Jiandanglika Subsidies

	Freq.	%
0 No	638	100.00
Total	638	100.00

ge003_4__s17: HHMemberName[17] Receive Jiandanglika Subsidies

	Freq.	%
0 No	638	100.00
Total	638	100.00

ge003_4__s18: HHMemberName[18] Receive Jiandanglika Subsidies

	Freq.	%
0 No	638	100.00
Total	638	100.00

ge003_4__s19: HHMemberName[19] Receive Jiandanglika Subsidies

	Freq.	%
0 No	638	100.00
Total	638	100.00

ge003_4__s20: HHMemberName[20] Receive Jiandanglika Subsidies

	Freq.	%
0 No	638	100.00
Total	638	100.00

ge003_4__s21: HHMemberName[21] Receive Jiandanglika Subsidies

	Freq.	%
0 No	638	100.00
Total	638	100.00

ge003_4__s22: HHMemberName[22] Receive Jiandanglika Subsidies

	Freq.	%
0 No	638	100.00
Total	638	100.00

ge003_4__s23: HHMemberName[23] Receive Jiandanglika Subsidies

	Freq.	%
0 No	638	100.00
Total	638	100.00

ge003_4__s24: HHMemberName[24] Receive Jiandanglika Subsidies

	Freq.	%
0 No	638	100.00
Total	638	100.00

ge003_4__s25: HHMemberName[25] Receive Jiandanglika Subsidies

	Freq.	%
0 No	638	100.00
Total	638	100.00

ge003_4__s26: Main Respondent Receive Jiandanglika Subsidies

	Freq.	%
0 No	102	15.99
26 Yes	536	84.01
Total	638	100.00

ge003_4__s27: Main Respondent's Spouse Receive Jiandanglika Subsidies

	Freq.	%
0 No	324	50.78
27 Yes	314	49.22
Total	638	100.00

ge003_5__s1: HHMemberName[1] Receive other Poor Household Subsidies

	Freq.	%
0 No	70	78.65
1 Yes	19	21.35
Total	89	100.00

ge003_5__s2: HHMemberName[2] Receive other Poor Household Subsidies

	Freq.	%
0 No	82	92.13
2 Yes	7	7.87
Total	89	100.00

ge003_5__s3: HHMemberName[3] Receive other Poor Household Subsidies

	Freq.	%
0 No	88	98.88
3 Yes	1	1.12
Total	89	100.00

ge003_5__s4: HHMemberName[4] Receive other Poor Household Subsidies

	Freq.	%
0 No	88	98.88
4 Yes	1	1.12
Total	89	100.00

ge003_5__s5: HHMemberName[5] Receive other Poor Household Subsidies

	Freq.	%
0 No	88	98.88
5 Yes	1	1.12
Total	89	100.00

ge003_5__s6: HHMemberName[6] Receive other Poor Household Subsidies

	Freq.	%
0 No	89	100.00
Total	89	100.00

ge003_5__s7: HHMemberName[7] Receive other Poor Household Subsidies

	Freq.	%
0 No	89	100.00
Total	89	100.00

ge003_5__s8: HHMemberName[8] Receive other Poor Household Subsidies

	Freq.	%
0 No	89	100.00
Total	89	100.00

ge003_5__s9: HHMemberName[9] Receive other Poor Household Subsidies

	Freq.	%
0 No	89	100.00
Total	89	100.00

ge003_5__s10: HHMemberName[10] Receive other Poor Household Subsidies

	Freq.	%
0 No	89	100.00
Total	89	100.00

ge003_5__s11: HHMemberName[11] Receive other Poor Household Subsidies

	Freq.	%
0 No	89	100.00
Total	89	100.00

ge003_5__s12: HHMemberName[12] Receive other Poor Household Subsidies

	Freq.	%
0 No	89	100.00
Total	89	100.00

ge003_5__s13: HHMemberName[13] Receive other Poor Household Subsidies

	Freq.	%
0 No	89	100.00
Total	89	100.00

ge003_5__s14: HHMemberName[14] Receive other Poor Household Subsidies

	Freq.	%
0 No	89	100.00
Total	89	100.00

ge003_5__s15: HHMemberName[15] Receive other Poor Household Subsidies

	Freq.	%
0 No	89	100.00
Total	89	100.00

ge003_5__s16: HHMemberName[16] Receive other Poor Household Subsidies

	Freq.	%
0 No	89	100.00
Total	89	100.00

ge003_5__s17: HHMemberName[17] Receive other Poor Household Subsidies

	Freq.	%
0 No	89	100.00
Total	89	100.00

ge003_5__s18: HHMemberName[18] Receive other Poor Household Subsidies

	Freq.	%
0 No	89	100.00
Total	89	100.00

ge003_5__s19: HHMemberName[19] Receive other Poor Household Subsidies

	Freq.	%
0 No	89	100.00
Total	89	100.00

ge003_5__s20: HHMemberName[20] Receive other Poor Household Subsidies

	Freq.	%
0 No	89	100.00
Total	89	100.00

ge003_5__s21: HHMemberName[21] Receive other Poor Household Subsidies

	Freq.	%
0 No	89	100.00
Total	89	100.00

ge003_5__s22: HHMemberName[22] Receive other Poor Household Subsidies

	Freq.	%
0 No	89	100.00
Total	89	100.00

ge003_5__s23: HHMemberName[23] Receive other Poor Household Subsidies

	Freq.	%
0 No	89	100.00
Total	89	100.00

ge003_5__s24: HHMemberName[24] Receive other Poor Household Subsidies

	Freq.	%
0 No	89	100.00
Total	89	100.00

ge003_5__s25: HHMemberName[25] Receive other Poor Household Subsidies

	Freq.	%
0 No	89	100.00
Total	89	100.00

ge003_5__s26: Main Respondent Receive other Poor Household Subsidies

	Freq.	%
0 No	18	20.22
26 Yes	71	79.78
Total	89	100.00

ge003_5__s27: Main Respondent's Spouse Receive other Poor Household Subsidies

	Freq.	%
0 No	47	52.81
27 Yes	42	47.19
Total	89	100.00

ge004_1_: Amount of Wubao Subsidies

Mean	SD	Min	Max	Obs
5,374.67	3,762.12	-1.00	22,000.00	119

ge004_2_: Amount of Dibao Subsidies

Mean	SD	Min	Max	Obs
3,907.70	5,479.79	-1.00	90,000.00	838

ge004_3_: Amount of Tekun Subsidies

Mean	SD	Min	Max	Obs
2,837.85	4,617.13	-1.00	22,000.00	54

ge004_4_: Amount of Jiandanglika Subsidies

Mean	SD	Min	Max	Obs
3,173.79	9,307.10	-1.00	125,000.00	638

ge004_5_: Amount of other Poor Household Subsidies

Mean	SD	Min	Max	Obs
4,364.85	13,052.07	-1.00	80,400.00	89

ge004_min_1_: Min Bracket of ge004_1_

Mean	SD	Min	Max	Obs
5,000.00	0.00	5,000.00	5,000.00	2

ge004_max_1_: Max Bracket of ge004_1_

Mean	SD	Min	Max	Obs
12,500.00	10,606.60	5,000.00	20,000.00	2

ge004_min_2_: Min Bracket of ge004_2_

Mean	SD	Min	Max	Obs
1,902.16	3,116.68	-1.00	20,000.00	82

ge004_max_2_: Max Bracket of ge004_2_

Mean	SD	Min	Max	Obs
5,040.94	5,228.48	-1.00	20,000.00	85

ge004_min_3_: Min Bracket of ge004_3_

Mean	SD	Min	Max	Obs
1,333.00	1,862.19	-1.00	5,000.00	6

ge004_max_3_: Max Bracket of ge004_3_

Mean	SD	Min	Max	Obs
5,166.67	2,857.74	1,000.00	10,000.00	6

ge004_min_4_: Min Bracket of ge004_4_

Mean	SD	Min	Max	Obs
1,381.49	2,679.70	-1.00	10,000.00	72

ge004_max_4_: Max Bracket of ge004_4_

Mean	SD	Min	Max	Obs
3,713.96	4,618.18	-1.00	20,000.00	77

ge004_min_5_: Min Bracket of ge004_5_

Mean	SD	Min	Max	Obs
5,249.83	7,515.26	-1.00	20,000.00	12

ge004_max_5_: Max Bracket of ge004_5_

Mean	SD	Min	Max	Obs
2,999.87	3,950.70	-1.00	10,000.00	15

ge006_s1: Receive Reforestation Subsidies

	Freq.	%
0 No	10,561	92.98
1 Yes	797	7.02
Total	11,358	100.00

ge006_s2: Receive Agricultural Subsidies

	Freq.	%
0 No	7,655	67.40
2 Yes	3,703	32.60
Total	11,358	100.00

ge006_s3: Receive Work Injury Subsidies

	Freq.	%
0 No	11,333	99.78
3 Yes	25	0.22
Total	11,358	100.00

ge006_s4: Receive Emergency or Disaster Relief

	Freq.	%
0 No	11,282	99.33
4 Yes	76	0.67
Total	11,358	100.00

ge006_s5: Receive Social Subsidies

	Freq.	%
0 No	11,263	99.16
5 Yes	95	0.84
Total	11,358	100.00

ge006_s6: Receive Compensation for Land Seizure

	Freq.	%
0 No	10,959	96.49
6 Yes	399	3.51
Total	11,358	100.00

ge006_s7: Receive Compensation to Pull Down House or Apartment

	Freq.	%
0 No	11,171	98.35
7 Yes	187	1.65
Total	11,358	100.00

ge006_s8: Receive Other Subsidies

	Freq.	%
0 No	10,910	96.06
8 Yes	448	3.94
Total	11,358	100.00

ge006_s9: None of the Above

	Freq.	%
0 No	4,754	41.86
9 Yes	6,604	58.14
Total	11,358	100.00

ge006_1: Amount of Reforestation Subsidies

Mean	SD	Min	Max	Obs
687.16	3,284.78	-1.00	80,000.00	797

ge006_2: Amount of Agricultural Subsidies

Mean	SD	Min	Max	Obs
765.39	3,849.86	-1.00	150,000.00	3,703

ge006_3: Amount of Work Injury Subsidies

Mean	SD	Min	Max	Obs
2,972.08	2,870.16	-1.00	9,600.00	25

ge006_4: Amount of Emergency or Disaster Relief

Mean	SD	Min	Max	Obs
1,035.86	3,238.70	-1.00	20,000.00	76

ge006_5: Amount of Social Subsidies

Mean	SD	Min	Max	Obs
1,206.40	4,284.43	-1.00	30,000.00	95

ge006_6: Amount of Compensation for Land Seizure

Mean	SD	Min	Max	Obs
16,495.18	50,991.96	-1.00	700,000.00	400

ge006_7: Amount of Compensation to Pull Down House or Apartment

Mean	SD	Min	Max	Obs
130,388.84	287,426.62	-1.00	2,000,000.00	187

ge006_8: Amount of Other Subsidies

Mean	SD	Min	Max	Obs
3,458.61	7,457.48	-1.00	60,000.00	448

ge007: Productive Insurance Payment

Mean	SD	Min	Max	Obs
30.24	862.61	-1.00	78,000.00	11,358

ge009: Photovoltaic Power Generation

	Freq.	%
1 Yes	94	0.83
2 No	11,264	99.17
Total	11,358	100.00

ge010: Begin Time for Photovoltaic Power Generation

Mean	SD	Min	Max	Obs
1,802.29	625.53	-1.00	2,020.00	94

ge011: Income from Photovoltaic Power Generation

Mean	SD	Min	Max	Obs
1,349.45	4,120.06	-1.00	30,000.00	94

ge012: Land Rent

Mean	SD	Min	Max	Obs
426.66	4,055.78	-1.00	350,000.00	11,358

ge012_min: Min Bracket of ge012

Mean	SD	Min	Max	Obs
693.33	1,357.43	-1.00	5,000.00	49

ge012_max: Max Bracket of ge012

Mean	SD	Min	Max	Obs
1,414.83	2,425.25	-1.00	10,000.00	59

ge013: House Rent

Mean	SD	Min	Max	Obs
889.93	7,753.70	-1.00	350,000.00	11,358

ge013_min: Min Bracket of ge013

Mean	SD	Min	Max	Obs
1,405.69	2,170.02	-1.00	5,000.00	16

ge013_max: Max Bracket of ge013

Mean	SD	Min	Max	Obs
1,655.63	2,970.89	-1.00	10,000.00	16

ge014: Rental Income from Other Household Assets

	Freq.	%
1 Yes	41	0.36
2 No	11,299	99.48
997 Do not Know	17	0.15
999 Refused to Answer	1	0.01
Total	11,358	100.00

ge014_1: Amount of Rental Income from Other Household Assets

Mean	SD	Min	Max	Obs
7,841.46	14,430.89	0.00	75,000.00	41

gf001: Monthly Household Expenditure

Mean	SD	Min	Max	Obs
2,315.37	3,075.04	0.00	142,000.00	11,356

gf002: Primary Person Who Purchases Food

	Freq.	%
1 Yes	9,273	81.66
2 No	2,083	18.34
Total	11,356	100.00

gf003: Who is Primary Person Purchasing Food

	Freq.	%
1 XChildAliveName[1]	257	12.34
2 XChildAliveName[2]	120	5.76
3 XChildAliveName[3]	70	3.36
4 XChildAliveName[4]	42	2.02
5 XChildAliveName[5]	15	0.72
6 XChildAliveName[6]	7	0.34
7 XChildAliveName[7]	2	0.10
8 XChildAliveName[8]	1	0.05
9 XChildAliveName[9]	1	0.05
11 XChildAliveName[11]	1	0.05
26 XHHOtherMemberName2[1]	179	8.60
27 XHHOtherMemberName2[2]	12	0.58
28 XHHOtherMemberName2[3]	17	0.82
29 XHHOtherMemberName2[4]	6	0.29
36 Main Respondent	429	20.61
37 Main Respondent's Spouse	692	33.24
38 Domestic Servant	12	0.58
39 Neighbor	5	0.24
40 Other	214	10.28
Total	2,082	100.00

gf004: Number of People Ate Meals in the Past Week

Mean	SD	Min	Max	Obs
3.10	1.82	0.00	25.00	11,355

gf005: Number of Meals Provided to Guests

Mean	SD	Min	Max	Obs
1.53	7.58	0.00	251.00	11,355

gf006: Food Expenditure, Excluding Some Items

Mean	SD	Min	Max	Obs
303.70	558.08	0.00	38,000.00	11,355

gf007: Produce Agricultural Products Yourself

	Freq.	%
1 Yes	5,836	51.40
2 No	5,519	48.60
Total	11,355	100.00

gf008: Market Value of Agricultural Products

Mean	SD	Min	Max	Obs
111.74	212.57	0.00	5,500.00	5,836

gf009: Eating Out

Mean	SD	Min	Max	Obs
50.38	216.01	0.00	6,000.00	11,355

gf010: Alcohol, Cigarettes, Cigars and Tobacco

Mean	SD	Min	Max	Obs
70.21	222.79	0.00	15,000.00	11,354

gf011_1: Communication Fees

Mean	SD	Min	Max	Obs
163.79	245.96	-1.00	10,000.00	11,354

gf011_2: Utilities

Mean	SD	Min	Max	Obs
162.12	388.01	-1.00	30,000.00	11,354

gf011_3: Fuels

Mean	SD	Min	Max	Obs
75.93	199.37	-1.00	10,000.00	11,354

gf011_4: Fees for Matron, Housekeepers and Servants

Mean	SD	Min	Max	Obs
27.73	387.03	-1.00	15,000.00	11,354

gf011_5: Local Transportation

Mean	SD	Min	Max	Obs
74.82	268.82	-1.00	10,000.00	11,354

gf011_6: Household Items and Personal Toiletries

Mean	SD	Min	Max	Obs
93.30	224.35	-1.00	10,000.00	11,354

gf011_7: Entertainment

Mean	SD	Min	Max	Obs
9.99	67.23	-1.00	4,000.00	11,354

gf012_s1: Elderly in the Household

	Freq.	%
0 No	55	47.83
1 Yes	60	52.17
Total	115	100.00

gf012_s2: Child in the Household

	Freq.	%
0 No	104	90.43
2 Yes	11	9.57
Total	115	100.00

gf012_s3: Others

	Freq.	%
0 No	88	76.52
3 Yes	27	23.48
Total	115	100.00

gf012_s4: None of the Above

	Freq.	%
0 No	89	77.39
4 Yes	26	22.61
Total	115	100.00

gf013_1: Clothing and Bedding

Mean	SD	Min	Max	Obs
1,755.70	3,462.12	-1.00	100,000.00	11,354

gf013_2: Traveling Expenses

Mean	SD	Min	Max	Obs
777.02	3,372.70	-1.00	100,000.00	11,354

gf013_3: Heating

Mean	SD	Min	Max	Obs
268.94	977.53	-1.00	42,000.00	11,354

gf013_4: Furniture, Durable Goods and Electronics

Mean	SD	Min	Max	Obs
1,055.86	6,511.01	-1.00	350,000.00	11,354

gf013_5: Education and Training

Mean	SD	Min	Max	Obs
1,856.32	7,289.42	-1.00	300,000.00	11,354

gf013_6: Medical Expenditure

Mean	SD	Min	Max	Obs
5,911.29	18,487.35	-1.00	650,000.00	11,354

gf013_7: Fitness Expenditures

Mean	SD	Min	Max	Obs
257.38	1,862.09	-1.00	50,000.00	11,354

gf013_8: Beauty

Mean	SD	Min	Max	Obs
272.93	1,378.69	-1.00	75,000.00	11,354

gf013_9: Automobiles

Mean	SD	Min	Max	Obs
2,237.07	16,954.52	-1.00	500,000.00	11,354

gf013_10: Purchase, Maintenance and Repair

Mean	SD	Min	Max	Obs
880.78	2,543.24	-1.00	100,000.00	11,354

gf013_11: Property Management Fees

Mean	SD	Min	Max	Obs
256.98	1,307.62	-1.00	69,200.00	11,354

gf013_12: Taxes and Fees

Mean	SD	Min	Max	Obs
221.33	6,596.19	-1.00	500,000.00	11,354

gf013_13: Donations

Mean	SD	Min	Max	Obs
116.38	764.19	-1.00	50,000.00	11,354

gf013_14: Rent

Mean	SD	Min	Max	Obs
772.37	4,878.55	-1.00	240,000.00	11,354

gf013_15: Banquet and Wedding Expenditure

Mean	SD	Min	Max	Obs
899.51	7,003.56	-1.00	300,000.00	11,354

gf013_16: COVID Prevention Expenditure

Mean	SD	Min	Max	Obs
184.59	432.88	-1.00	15,000.00	11,354

gf014: Is Expenditure Covered by Income?

	Freq.	%
1 Very Difficult	1,640	14.44
2 Difficult	4,952	43.61
3 Easy	4,298	37.85
4 Very Easy	464	4.09
Total	11,354	100.00

gf015_s1: Stop Paying Rent

	Freq.	%
0 No	6,557	99.47
1 Yes	35	0.53
Total	6,592	100.00

gf015_s2: Stop Paying Mortgage

	Freq.	%
0 No	6,571	99.68
2 Yes	21	0.32
Total	6,592	100.00

gf015_s3: Stop Paying Utility Bill

	Freq.	%
0 No	6,465	98.07

	Freq.	%
3 Yes	127	1.93
Total	6,592	100.00

gf015_s4: None of the Above

	Freq.	%
0 No	167	2.53
4 Yes	6,425	97.47
Total	6,592	100.00

gf016_s1: Reduce Spendings

	Freq.	%
0 No	2,468	37.45
1 Yes	4,123	62.55
Total	6,591	100.00

gf016_s2: Use Savings

	Freq.	%
0 No	5,146	78.08
2 Yes	1,445	21.92
Total	6,591	100.00

gf016_s3: Sale of Assets

	Freq.	%
0 No	6,451	97.88
3 Yes	140	2.12
Total	6,591	100.00

gf016_s4: Help from Relatives and Friends

	Freq.	%
0 No	6,090	92.40
4 Yes	501	7.60
Total	6,591	100.00

gf016_s5: Borrow Money from Relatives and Friends

	Freq.	%
0 No	5,533	83.95
5 Yes	1,058	16.05
Total	6,591	100.00

gf016_s6: Borrow Money from Banks

	Freq.	%

	Freq.	%
0 No	6,271	95.14
6 Yes	320	4.86
Total	6,591	100.00

gf016_s7: Others

	Freq.	%
0 No	6,404	97.16
7 Yes	187	2.84
Total	6,591	100.00

gf016_s8: Others

	Freq.	%
0 No	5,168	78.41
8 Yes	1,423	21.59
Total	6,591	100.00

gf016_1: Amount of Spendings Reduced

Mean	SD	Min	Max	Obs
2,447.66	4,299.23	-1.00	50,000.00	4,123

gf016_2: Amount of Savings

Mean	SD	Min	Max	Obs
13,488.79	31,542.48	-1.00	600,000.00	1,445

gf016_3: Amount of Assets

Mean	SD	Min	Max	Obs
47,281.72	422,592.44	-1.00	5,000,000.00	140

gf016_4: Value of Helps

Mean	SD	Min	Max	Obs
5,720.66	13,599.80	-1.00	200,000.00	501

gf016_5: Amount of Money Borrow from Relatives and Friends

Mean	SD	Min	Max	Obs
26,662.93	58,496.22	-1.00	1,000,000.00	1,058

gf016_6: Amount of Money Borrow from Banks

Mean	SD	Min	Max	Obs

97,629.62	216,523.58	-1.00	3,000,000.00	320

gf016_7: Amount from Other Methods

Mean	SD	Min	Max	Obs
7,673.42	16,583.34	-1.00	150,000.00	187

gf017: Receive Assistance

	Freq.	%
1 Never	8,630	76.02
2 A Few Times	2,378	20.95
3 Most or All of the Time	345	3.04
Total	11,353	100.00

i028: House Size

Mean	SD	Min	Max	Obs
138.17	111.44	1.00	2,129.60	11,352

i001: Building Structure

	Freq.	%
1 Concrete and Steel/Bricks and Wood	10,255	90.33
2 Adobe	655	5.77
3 Wood/Thatched	26	0.23
4 Cave Dwelling	52	0.46
5 Mongolian Yurt/Woolen Felt/Tent	5	0.04
6 Stone	144	1.27
7 Other	216	1.90
Total	11,353	100.00

i002: Time House Built

Mean	SD	Min	Max	Obs
1,872.60	493.88	-1.00	2,020.00	11,353

i003: Time Interval House Built

	Freq.	%
1 0-5 Years	30	4.07
2 5-10 Years	76	10.31
3 10-20 Years	187	25.37
4 20-30 Years	195	26.46
5 30-40 Years	111	15.06
6 More Than 40 Years	138	18.72
Total	737	100.00

i004: One Storey or Multi-level Building

	Freq.	%
1 One-storey Building	4,426	38.99
2 Common Multi-storey Building	2,904	25.58
3 Self-contained Multi-storey Building	4,023	35.44
Total	11,353	100.00

i005: The Storey is Independent or Compound

	Freq.	%
1 Independent Storey	4,054	91.60
2 Compound	372	8.40
Total	4,426	100.00

i006: Which Storey

	Freq.	%
1 On the Ground	2,896	99.72
2 Underground	8	0.28
Total	2,904	100.00

i006_1: Which Storey on the Ground

Mean	SD	Min	Max	Obs
4.74	5.05	1.00	38.00	2,896

i006_2: Which Storey Underground

Mean	SD	Min	Max	Obs
1.38	1.06	1.00	4.00	8

i007: Elevator

	Freq.	%
1 Yes	698	10.93
2 No	5,688	89.07
Total	6,386	100.00

i008: Building Time for Elevator

	Freq.	%
1 The Same Time as Building	679	97.28
2 Later Added	5	0.72
3 Do not Know	14	2.01
Total	698	100.00

i008_1: Time for Later Added Elevator

Mean	SD	Min	Max	Obs
1,210.20	1,105.67	-1.00	2,018.00	5

i009: Handicapped Facilities

	Freq.	%
1 Yes	2,050	18.06
2 No	4,731	41.67
3 Do Not Need	4,572	40.27
Total	11,353	100.00

i010: Steps to Main Entrance of the House

Mean	SD	Min	Max	Obs
19.33	33.46	0.00	540.00	4,731

i011_1: Bedrooms

Mean	SD	Min	Max	Obs
3.18	2.17	0.00	43.00	11,353

i011_2: Livingrooms

Mean	SD	Min	Max	Obs
1.23	0.77	0.00	11.00	11,353

i011_3: Toilets

Mean	SD	Min	Max	Obs
1.27	1.23	0.00	46.00	11,353

i011_4: Kitchens

Mean	SD	Min	Max	Obs
1.03	0.61	0.00	33.00	11,353

i011_5: Balcony

Mean	SD	Min	Max	Obs
0.62	0.80	0.00	11.00	11,353

i012: Distance from the Nearest Toilet to House

Mean	SD	Min	Max	Obs
43.35	103.55	0.00	2,000.00	1,413

i013: Type of Toilet

	Freq.	%
1 Without a Seat	7,120	62.71
2 With a Seat	4,233	37.29
Total	11,353	100.00

i014: Flushable Toilet

	Freq.	%
1 Yes	3,931	55.21
2 No	3,189	44.79
Total	7,120	100.00

i015: Electricity

	Freq.	%
1 Yes	11,277	99.33
2 No	76	0.67
Total	11,353	100.00

i016: Tap Water

	Freq.	%
1 Yes	10,304	90.76
2 No	1,049	9.24
Total	11,353	100.00

i029: Water is Treated by Waterworks

	Freq.	%
1 Yes	6,686	64.89
2 No	3,214	31.19
997 Do not Know	404	3.92
Total	10,304	100.00

i017: In-house Shower

	Freq.	%
1 Unified Hot Water Supply	161	1.42
2 Water Heater	7,968	70.19
3 No	3,223	28.39
Total	11,352	100.00

i018: Coal Gas or Natural Gas Supply

	Freq.	%
1 Yes	3,025	26.65
2 No	8,327	73.35

Total	11,352	100.00

i019: Centralized Heating

	Freq.	%
1 Yes	1,281	11.28
2 No	10,071	88.72
Total	11,352	100.00

i020: Heating Energy Source

	Freq.	%
1 Solar	42	0.42
2 Coal	2,320	23.04
3 Natural Gas	339	3.37
4 Liquefied Petroleum Gas	36	0.36
5 Electric	3,088	30.66
6 Crop Residue/Wood Burning	1,353	13.43
7 Others	109	1.08
8 Do not Heating	2,784	27.64
Total	10,071	100.00

i021: Main Source of Cooking Fuel

	Freq.	%
1 Coal	468	4.12
2 Natural Gas	3,431	30.22
3 Marsh Gas	24	0.21
4 Liquefied Petroleum Gas	1,733	15.27
5 Electric	3,051	26.88
6 Crop Residue/Wood Burning	2,422	21.34
7 Solar Energy	4	0.04
8 Others	151	1.33
9 Do not Cook	68	0.60
Total	11,352	100.00

i022: Telephone

	Freq.	%
1 Yes	1,541	13.57
2 No	9,811	86.43
Total	11,352	100.00

i023: Broad-band Internet Connection

	Freq.	%
1 Yes	6,749	59.45
2 No	4,603	40.55
Total	11,352	100.00

i024: Air Cleaner

	Freq.	%
1 Yes	442	3.89
2 No	10,910	96.11
Total	11,352	100.00

i025: Clear and Tidy in this Household

	Freq.	%
1 Excellent	1,427	12.57
2 Very Clear	2,250	19.82
3 Clear	3,798	33.46
4 Fair	2,614	23.03
5 Poor	509	4.48
6 Not Applicable	754	6.64
Total	11,352	100.00

i026: Temperature in this Household

	Freq.	%
1 Very Hot	30	0.26
2 Hot	706	6.22
3 Bearable	9,715	85.58
4 Cold	141	1.24
5 Very Cold	5	0.04
6 Not Applicable	755	6.65
Total	11,352	100.00

i027: Ground Condition in this Household

	Freq.	%
1 Flat Coverd	7,359	64.83
2 Flat Concrete	3,518	30.99
3 Uneven	475	4.18
Total	11,352	100.00

xgb005text_1_: Text for gb005_1_

A String Variable	
Obs:	1,536

xgb005text_2_: Text for gb005_2_

A String Variable	
Obs:	639

xgb005text_3_: Text for gb005_3_

A String Variable

Obs:	159

xgb005text_4_: Text for gb005_4_

A String Variable	
Obs:	85

xgb005text_5_: Text for gb005_5_

A String Variable	
Obs:	25

xgb005text_6_: Text for gb005_6_

A String Variable	
Obs:	3

xgb005text_7_: Text for gb005_7_

A String Variable	
Obs:	4

xgb005text_8_: Text for gb005_8_

A String Variable	
Obs:	4

xgb005text_9_: Text for gb005_9_

A String Variable	
Obs:	1

xgb005text_10_: Text for gb005_10_

A String Variable	
Obs:	3

xpoorhhname_1_: Wubao Text

A String Variable	
Obs:	1,830

xpoorhhname_2_: Dibao Text

A String Variable

Obs:	1,830

xpoorhhname_3_: Tekun Text

A String Variable	
Obs:	1,830

xpoorhhname_4_: Jiandanglika Poor Household Text

A String Variable	
Obs:	1,830

xpoorlstyr_1_: Wubao Last Year

Mean	SD	Min	Max	Obs
0.93	0.26	0.00	1.00	128

xpoorlstyr_2_: Dibao Last Year

Mean	SD	Min	Max	Obs
0.82	0.39	0.00	1.00	1,027

xpoorlstyr_3_: Tekun Last Year

Mean	SD	Min	Max	Obs
0.82	0.39	0.00	1.00	66

xpoorlstyr_4_: Jiandanglika Poor Household Last Year

Mean	SD	Min	Max	Obs
0.82	0.39	0.00	1.00	782

xpoorlstyr_5_: Other Poor Household Last Year

Mean	SD	Min	Max	Obs
0.84	0.37	0.00	1.00	106

xchildnum: Num of Children

Mean	SD	Min	Max	Obs
2.56	1.38	0.00	10.00	11,376

xhhmembernum: Num of Household Member, Exclude Main Respondent and Spouse

Mean	SD	Min	Max	Obs

1.14	1.55	0.00	14.00	11,376

xhhmemberage_1_: Household Member[1]'s Age

Mean	SD	Min	Max	Obs
34.07	16.99	1.00	117.00	5,578

xhhmemberage_2_: Household Member[2]'s Age

Mean	SD	Min	Max	Obs
30.00	18.62	0.00	99.00	3,313

xhhmemberage_3_: Household Member[3]'s Age

Mean	SD	Min	Max	Obs
16.98	16.15	0.00	95.00	2,054

xhhmemberage_4_: Household Member[4]'s Age

Mean	SD	Min	Max	Obs
14.17	14.93	0.00	98.00	1,147

xhhmemberage_5_: Household Member[5]'s Age

Mean	SD	Min	Max	Obs
13.02	14.77	0.00	95.00	364

xhhmemberage_6_: Household Member[6]'s Age

Mean	SD	Min	Max	Obs
12.87	14.88	0.00	94.00	157

xhhmemberage_7_: Household Member[7]'s Age

Mean	SD	Min	Max	Obs
12.86	15.62	0.00	91.00	84

xhhmemberage_8_: Household Member[8]'s Age

Mean	SD	Min	Max	Obs
12.40	14.79	0.00	59.00	42

xhhmemberage_9_: Household Member[9]'s Age

Mean	SD	Min	Max	Obs

8.14	8.06	0.00	35.00	22

xhhmemberage_10_: Household Member[10]'s Age

Mean	SD	Min	Max	Obs
10.29	10.37	1.00	31.00	14

xhhmemberage_11_: Household Member[11]'s Age

Mean	SD	Min	Max	Obs
11.43	9.93	1.00	28.00	7

xhhmemberage_12_: Household Member[12]'s Age

Mean	SD	Min	Max	Obs
9.67	13.32	1.00	25.00	3

xhhmemberage_13_: Household Member[13]'s Age

Mean	SD	Min	Max	Obs
23.00	.	23.00	23.00	1

xhhmemberage_14_: Household Member[14]'s Age

Mean	SD	Min	Max	Obs
20.00	.	20.00	20.00	1

versionID: Version ID

A String Variable	
Obs:	11,377

This page intentionally left blank

MODULE G2

Individual Income

ID: Individual ID

A String Variable	
Obs:	19,349

householdID: Household ID

A String Variable	
Obs:	19,349

communityID: Community ID

A String Variable	
Obs:	19,349

ga001: Wage and Bonus Income

	Freq.	%
1 Yes	4,920	25.43
2 No	14,425	74.57
Total	19,345	100.00

ga002: Amount

Mean	SD	Min	Max	Obs
26,964.62	40,528.94	-1.00	1,500,000.00	4,919

ga002_min: Min Bracket of ga002

Mean	SD	Min	Max	Obs
19,403.43	25,080.84	-1.00	100,000.00	109

ga002_max: Max Bracket of ga002

Mean	SD	Min	Max	Obs
23,833.13	27,968.75	-1.00	100,000.00	120

ga003: Exclude Insurance and Some Others

	Freq.	%
1 Yes	897	18.24
2 No	3,952	80.34
997 Do not Know	68	1.38
999 Refused to Answer	2	0.04
Total	4,919	100.00

ga004: Total Excluded Amount

	Freq.	%
1 Yuan/Month	777	15.80
2 Yuan/Year	413	8.40
3 Percentage of Wage	162	3.29
4 No	3,565	72.50
Total	4,917	100.00

ga004_1: Yuan/Month

Mean	SD	Min	Max	Obs
641.71	966.74	-1.00	6,100.00	777

ga004_1_min: Min Bracket of ga004_1

Mean	SD	Min	Max	Obs
499.86	784.86	-1.00	3,000.00	274

ga004_1_max: Max Bracket of ga004_1

Mean	SD	Min	Max	Obs
556.53	805.92	-1.00	3,000.00	286

ga004_2: Yuan/Year

Mean	SD	Min	Max	Obs
5,748.23	11,268.78	30.00	100,000.00	413

ga004_3: Percentage of Wage

Mean	SD	Min	Max	Obs
13.78	9.90	0.01	50.00	162

ga005_s1: Pension Receive

	Freq.	%
0 No	9,603	49.65
1 Yes	9,740	50.35
Total	19,343	100.00

ga005_s2: Unemployment Compensation Receive

	Freq.	%
0 No	19,299	99.77
2 Yes	44	0.23
Total	19,343	100.00

ga005_s3: Pension Voucher Receive

	Freq.	%
0 No	19,082	98.65
3 Yes	261	1.35
Total	19,343	100.00

ga005_s4: Pension Subsidy for the Oldest Old Receive

	Freq.	%
0 No	18,171	93.94
4 Yes	1,172	6.06
Total	19,343	100.00

ga005_s5: Workers' Industrial Accident Compensation Receive

	Freq.	%
0 No	19,264	99.59
5 Yes	79	0.41
Total	19,343	100.00

ga005_s6: Elderly Family Planning Subsidy Receive

	Freq.	%
0 No	18,614	96.23
6 Yes	729	3.77
Total	19,343	100.00

ga005_s7: Medical Aid Receive

	Freq.	%
0 No	19,116	98.83
7 Yes	227	1.17
Total	19,343	100.00

ga005_s8: Other Government Subsidy Receive

	Freq.	%
0 No	18,452	95.39
8 Yes	891	4.61
Total	19,343	100.00

ga005_s9: Other Income Source Receive

	Freq.	%
0 No	19,234	99.44
9 Yes	109	0.56
Total	19,343	100.00

ga005_s10: None of the Above Receive

	Freq.	%
0 No	10,911	56.41
10 Yes	8,432	43.59
Total	19,343	100.00

ga005_1: Pension Amount Receive

Mean	SD	Min	Max	Obs
12,374.47	19,223.88	-1.00	567,200.00	9,741

ga005_1_min: Min Bracket of ga005_1

Mean	SD	Min	Max	Obs
1,634.54	4,010.10	-1.00	25,000.00	315

ga005_1_max: Max Bracket of ga005_1

Mean	SD	Min	Max	Obs
2,741.09	4,403.06	-1.00	25,000.00	348

ga005_2: Unemployment Compensation Amount Receive

Mean	SD	Min	Max	Obs
4,180.11	6,314.90	-1.00	27,600.00	44

ga005_3: Pension Voucher Amount Receive

Mean	SD	Min	Max	Obs
2,242.27	5,867.91	-1.00	45,000.00	261

ga005_4: Pension Subsidy for the Oldest Old Amount Receive

Mean	SD	Min	Max	Obs
924.99	838.35	-1.00	7,600.00	1,172

ga005_5: Workers' Industrial Accident Compensation Amount Receive

Mean	SD	Min	Max	Obs
3,351.54	6,842.90	-1.00	37,000.00	79

ga005_6: Elderly Family Planning Subsidy Amount Receive

Mean	SD	Min	Max	Obs
1,106.99	2,021.87	-1.00	50,000.00	729

ga005_7: Medical Aid Amount Receive

Mean	SD	Min	Max	Obs
3,780.04	18,184.31	-1.00	260,000.00	227

ga005_8: Other Government Subsidy Amount Receive

Mean	SD	Min	Max	Obs
2,669.59	4,488.35	-1.00	45,600.00	891

ga005_9: Other Income Source Amount Receive

Mean	SD	Min	Max	Obs
2,952.93	6,888.50	-1.00	40,000.00	109

ga006: Impact of COVID on Wage

	Freq.	%
1 Decrease	3,711	19.19
2 Increase	81	0.42
3 Unchange	15,550	80.39
Total	19,342	100.00

ga006_1: Amount Decreased

Mean	SD	Min	Max	Obs
8,564.39	21,305.38	-1.00	600,000.00	3,711

ga006_1_max: Max Bracket of ga006_1

Mean	SD	Min	Max	Obs
4,774.13	5,561.06	-1.00	20,000.00	266

ga006_1_min: Min Bracket of ga006_1

Mean	SD	Min	Max	Obs
4,565.24	5,665.60	-1.00	20,000.00	267

ga006_2: Amount Increased

Mean	SD	Min	Max	Obs
4,524.10	8,057.95	-1.00	60,000.00	81

ga006_2_max: Max Bracket of ga006_2

Mean	SD	Min	Max	Obs
2,999.83	3,633.35	-1.00	10,000.00	6

ga006_2_min: Min Bracket of ga006_2

Mean	SD	Min	Max	Obs
4,499.67	7,713.86	-1.00	20,000.00	6

ga007: Make a Will

	Freq.	%
1 Yes	88	0.45
2 No	19,253	99.55
Total	19,341	100.00

ga008: Estate Planning

	Freq.	%
1 Have Considered	1,339	6.95
2 Have not Considered	15,078	78.32
3 No Asset	2,836	14.73
Total	19,253	100.00

ga017: Estate Planning Follows Law of Succession

	Freq.	%
1 Yes	298	22.26
2 No, I Have My Plan	1,041	77.74
Total	1,339	100.00

ga009_s1: Estate Planning: Spouse

	Freq.	%
0 No	1,001	88.66
1 Yes	128	11.34

	Freq.	%
Total	1,129	100.00

ga009_s2: Estate Planning: Children/Sons-in-law/Daughters-in-law

	Freq.	%
0 No	134	11.87
2 Yes	995	88.13
Total	1,129	100.00

ga009_s3: Estate Planning: Sibling

	Freq.	%
0 No	1,124	99.56
3 Yes	5	0.44
Total	1,129	100.00

ga009_s4: Estate Planning: Other Relatives

	Freq.	%
0 No	1,122	99.38
4 Yes	7	0.62
Total	1,129	100.00

ga009_s5: Estate Planning: Parents/Parents-in-law

	Freq.	%
0 No	1,124	99.56
5 Yes	5	0.44
Total	1,129	100.00

ga009_s6: Estate Planning: Grandchildren

	Freq.	%
0 No	1,048	92.83
6 Yes	81	7.17
Total	1,129	100.00

ga009_s7: Estate Planning: Friends

	Freq.	%
0 No	1,129	100.00
Total	1,129	100.00

ga009_s8: Estate Planning: Charity

	Freq.	%
0 No	1,128	99.91
8 Yes	1	0.09

Total	1,129	100.00

ga009_s9: Estate Planning: Others

	Freq.	%
0 No	1,106	97.96
9 Yes	23	2.04
Total	1,129	100.00

ga009_s10: Estate Planning: No Arrangement

	Freq.	%
0 No	1,105	97.87
10 Yes	24	2.13
Total	1,129	100.00

ga009_s997: Estate Planning: Do not Know

	Freq.	%
0 No	1,129	100.00
Total	1,129	100.00

ga009_1: Estate Planning: Spouse(%)

Mean	SD	Min	Max	Obs
56.77	37.34	-1.00	100.00	130

ga009_3: Estate Planning: Sibling(%)

Mean	SD	Min	Max	Obs
66.00	46.69	10.00	100.00	5

ga009_4: Estate Planning: Other Relatives(%)

Mean	SD	Min	Max	Obs
87.14	34.02	10.00	100.00	7

ga009_5: Estate Planning: Parents/Parents-in-law(%)

Mean	SD	Min	Max	Obs
26.40	41.91	-1.00	100.00	5

ga009_7: Estate Planning: Friends(%)

No Observations

ga009_8: Estate Planning: Charity(%)

Mean	SD	Min	Max	Obs
50.00	.	50.00	50.00	1

ga009_9: Estate Planning: Others(%)

Mean	SD	Min	Max	Obs
59.96	45.06	-1.00	100.00	23

ga010_s1: Estate Planning: Child[1]/'s Spouse

	Freq.	%
0 No	211	21.21
1 Yes	784	78.79
Total	995	100.00

ga010_s2: Estate Planning: Child[2]/'s Spouse

	Freq.	%
0 No	576	57.89
2 Yes	419	42.11
Total	995	100.00

ga010_s3: Estate Planning: Child[3]/'s Spouse

	Freq.	%
0 No	820	82.41
3 Yes	175	17.59
Total	995	100.00

ga010_s4: Estate Planning: Child[4]/'s Spouse

	Freq.	%
0 No	917	92.16
4 Yes	78	7.84
Total	995	100.00

ga010_s5: Estate Planning: Child[5]/'s Spouse

	Freq.	%
0 No	974	97.89
5 Yes	21	2.11
Total	995	100.00

ga010_s6: Estate Planning: Child[6]/'s Spouse

	Freq.	%
0 No	984	98.89
6 Yes	11	1.11
Total	995	100.00

ga010_s7: Estate Planning: Child[7]/'s Spouse

	Freq.	%
0 No	993	99.80
7 Yes	2	0.20
Total	995	100.00

ga010_s8: Estate Planning: Child[8]/'s Spouse

	Freq.	%
0 No	995	100.00
Total	995	100.00

ga010_s9: Estate Planning: Child[9]/'s Spouse

	Freq.	%
0 No	995	100.00
Total	995	100.00

ga010_s10: Estate Planning: Child[10]/'s Spouse

	Freq.	%
0 No	995	100.00
Total	995	100.00

ga010_s11: Estate Planning: Child[11]/'s Spouse

	Freq.	%
0 No	995	100.00
Total	995	100.00

ga010_s12: Estate Planning: Child[12]/'s Spouse

	Freq.	%
0 No	995	100.00
Total	995	100.00

ga010_s13: Estate Planning: Child[13]/'s Spouse

	Freq.	%
0 No	995	100.00
Total	995	100.00

ga010_s14: Estate Planning: Child[14]/'s Spouse

	Freq.	%
0 No	995	100.00
Total	995	100.00

ga010_s15: Estate Planning: Child[15]/'s Spouse

	Freq.	%
0 No	995	100.00
Total	995	100.00

ga010_s16: Estate Planning: Child[16]/'s Spouse

	Freq.	%
0 No	995	100.00
Total	995	100.00

ga010_s17: Estate Planning: Child[17]/'s Spouse

	Freq.	%
0 No	995	100.00
Total	995	100.00

ga010_s18: Estate Planning: Child[18]/'s Spouse

	Freq.	%
0 No	995	100.00
Total	995	100.00

ga010_s19: Estate Planning: Child[19]/'s Spouse

	Freq.	%
0 No	995	100.00
Total	995	100.00

ga010_s20: Estate Planning: Child[20]/'s Spouse

	Freq.	%
0 No	995	100.00
Total	995	100.00

ga010_s21: Estate Planning: Child[21]/'s Spouse

	Freq.	%
0 No	995	100.00
Total	995	100.00

ga010_s22: Estate Planning: Child[22]/'s Spouse

	Freq.	%
0 No	995	100.00
Total	995	100.00

ga010_s23: Estate Planning: Child[23]/'s Spouse

	Freq.	%
0 No	995	100.00
Total	995	100.00

ga010_s24: Estate Planning: Child[24]/'s Spouse

	Freq.	%
0 No	995	100.00
Total	995	100.00

ga010_s25: Estate Planning: Child[25]/'s Spouse

	Freq.	%
0 No	995	100.00
Total	995	100.00

ga010_s26: Estate Planning: Other Child[26]/'s Spouse

	Freq.	%
0 No	977	98.19
26 Yes	18	1.81
Total	995	100.00

ga010_s27: Estate Planning: Other Child[27]/'s Spouse

	Freq.	%
0 No	989	99.40
27 Yes	6	0.60
Total	995	100.00

ga010_s28: Estate Planning: Other Child[28]/'s Spouse

	Freq.	%
0 No	994	99.90
28 Yes	1	0.10
Total	995	100.00

ga010_s29: Estate Planning: Other Child[29]/'s Spouse

	Freq.	%
0 No	994	99.90
29 Yes	1	0.10
Total	995	100.00

ga010_s30: Estate Planning: Other Child[30]/'s Spouse

	Freq.	%
0 No	994	99.90
30 Yes	1	0.10
Total	995	100.00

ga010_s31: Estate Planning: Other Child[31]/'s Spouse

	Freq.	%
0 No	995	100.00
Total	995	100.00

ga010_s32: Estate Planning: Other Child[32]/'s Spouse

	Freq.	%
0 No	995	100.00
Total	995	100.00

ga010_s33: Estate Planning: Other Child[33]/'s Spouse

	Freq.	%
0 No	995	100.00
Total	995	100.00

ga010_s34: Estate Planning: Other Child[34]/'s Spouse

	Freq.	%
0 No	995	100.00
Total	995	100.00

ga010_s35: Estate Planning: Other Child[35]/'s Spouse

	Freq.	%
0 No	995	100.00
Total	995	100.00

ga011_1_: Estate Planning: Child[1]/'s Spouse(%)

Mean	SD	Min	Max	Obs
71.69	33.56	-1.00	100.00	784

ga011_2_: Estate Planning: Child[2]/'s Spouse(%)

Mean	SD	Min	Max	Obs
50.86	30.86	-1.00	100.00	419

ga011_3_: Estate Planning: Child[3]/'s Spouse(%)

Mean	SD	Min	Max	Obs
42.61	32.25	-1.00	100.00	175

ga011_4_: Estate Planning: Child[4]/'s Spouse(%)

Mean	SD	Min	Max	Obs
41.87	35.08	-1.00	100.00	78

ga011_5_: Estate Planning: Child[5]/'s Spouse(%)

Mean	SD	Min	Max	Obs
29.20	23.26	-1.00	100.00	21

ga011_6_: Estate Planning: Child[6]/'s Spouse(%)

Mean	SD	Min	Max	Obs
37.76	27.52	0.10	100.00	11

ga011_7_: Estate Planning: Child[7]/'s Spouse(%)

Mean	SD	Min	Max	Obs
100.00	0.00	100.00	100.00	2

ga011_8_: Estate Planning: Child[8]/'s Spouse(%)

No Observations

ga011_9_: Estate Planning: Child[9]/'s Spouse(%)

No Observations

ga011_10_: Estate Planning: Child[10]/'s Spouse(%)

No Observations

ga011_11_: Estate Planning: Child[11]/'s Spouse(%)

No Observations

ga011_12_: Estate Planning: Child[12]/'s Spouse(%)

No Observations

ga011_13_: Estate Planning: Child[13]/'s Spouse(%)

No Observations

ga011_14_: Estate Planning: Child[14]/'s Spouse(%)

No Observations

ga011_15_: Estate Planning: Child[15]/'s Spouse(%)

No Observations

ga011_16_: Estate Planning: Child[16]/'s Spouse(%)

No Observations

ga011_17_: Estate Planning: Child[17]/'s Spouse(%)

No Observations

ga011_18_: Estate Planning: Child[18]/'s Spouse(%)

No Observations

ga011_19_: Estate Planning: Child[19]/'s Spouse(%)

No Observations

ga011_20_: Estate Planning: Child[20]/'s Spouse(%)

No Observations

ga011_21_: Estate Planning: Child[21]/'s Spouse(%)

No Observations

ga011_22_: Estate Planning: Child[22]/'s Spouse(%)

No Observations

ga011_23_: Estate Planning: Child[23]/'s Spouse(%)

No Observations

ga011_24_: Estate Planning: Child[24]/'s Spouse(%)

No Observations

ga011_25_: Estate Planning: Child[25]/'s Spouse(%)

No Observations

ga011_26_: Estate Planning: Other Child[26]/'s Spouse(%)

Mean	SD	Min	Max	Obs
64.00	28.06	20.00	100.00	18

ga011_27_: Estate Planning: Other Child[27]/'s Spouse(%)

Mean	SD	Min	Max	Obs
40.83	14.29	20.00	50.00	6

ga011_28_: Estate Planning: Other Child[28]/'s Spouse(%)

Mean	SD	Min	Max	Obs
20.00	.	20.00	20.00	1

ga011_29_: Estate Planning: Other Child[29]/'s Spouse(%)

Mean	SD	Min	Max	Obs
20.00	.	20.00	20.00	1

ga011_30_: Estate Planning: Other Child[30]/'s Spouse(%)

Mean	SD	Min	Max	Obs
20.00	.	20.00	20.00	1

ga011_31_: Estate Planning: Other Child[31]/'s Spouse(%)

No Observations

ga011_32_: Estate Planning: Other Child[32]/'s Spouse(%)

No Observations

ga011_33_: Estate Planning: Other Child[33]/'s Spouse(%)

No Observations

ga011_34_: Estate Planning: Other Child[34]/'s Spouse(%)

No Observations

ga011_35_: Estate Planning: Other Child[35]/'s Spouse(%)

No Observations

ga014_s1: Estate Planning: Child[1]'s Children

	Freq.	%
0 No	28	34.57
1 Yes	53	65.43
Total	81	100.00

ga014_s2: Estate Planning: Child[2]'s Children

	Freq.	%
0 No	50	61.73
2 Yes	31	38.27
Total	81	100.00

ga014_s3: Estate Planning: Child[3]'s Children

	Freq.	%
0 No	68	83.95
3 Yes	13	16.05
Total	81	100.00

ga014_s4: Estate Planning: Child[4]'s Children

	Freq.	%
0 No	78	96.30
4 Yes	3	3.70
Total	81	100.00

ga014_s5: Estate Planning: Child[5]'s Children

	Freq.	%
0 No	80	98.77
5 Yes	1	1.23
Total	81	100.00

ga014_s6: Estate Planning: Child[6]'s Children

	Freq.	%
0 No	81	100.00
Total	81	100.00

ga014_s7: Estate Planning: Child[7]'s Children

	Freq.	%
0 No	81	100.00
Total	81	100.00

ga014_s8: Estate Planning: Child[8]'s Children

	Freq.	%
0 No	81	100.00
Total	81	100.00

ga014_s9: Estate Planning: Child[9]'s Children

	Freq.	%
0 No	81	100.00
Total	81	100.00

ga014_s10: Estate Planning: Child[10]'s Children

	Freq.	%
0 No	81	100.00
Total	81	100.00

ga014_s11: Estate Planning: Child[11]'s Children

	Freq.	%
0 No	81	100.00
Total	81	100.00

ga014_s12: Estate Planning: Child[12]'s Children

	Freq.	%
0 No	81	100.00
Total	81	100.00

ga014_s13: Estate Planning: Child[13]'s Children

	Freq.	%
0 No	81	100.00
Total	81	100.00

ga014_s14: Estate Planning: Child[14]'s Children

	Freq.	%
0 No	81	100.00
Total	81	100.00

ga014_s15: Estate Planning: Child[15]'s Children

	Freq.	%
0 No	81	100.00
Total	81	100.00

ga014_s16: Estate Planning: Child[16]'s Children

	Freq.	%
0 No	81	100.00
Total	81	100.00

ga014_s17: Estate Planning: Child[17]'s Children

	Freq.	%
0 No	81	100.00
Total	81	100.00

ga014_s18: Estate Planning: Child[18]'s Children

	Freq.	%
0 No	81	100.00
Total	81	100.00

ga014_s19: Estate Planning: Child[19]'s Children

	Freq.	%
0 No	81	100.00
Total	81	100.00

ga014_s20: Estate Planning: Child[20]'s Children

	Freq.	%
0 No	81	100.00
Total	81	100.00

ga014_s21: Estate Planning: Child[21]'s Children

	Freq.	%
0 No	81	100.00
Total	81	100.00

ga014_s22: Estate Planning: Child[22]'s Children

	Freq.	%
0 No	81	100.00
Total	81	100.00

ga014_s23: Estate Planning: Child[23]'s Children

	Freq.	%
0 No	81	100.00
Total	81	100.00

ga014_s24: Estate Planning: Child[24]'s Children

	Freq.	%
0 No	81	100.00
Total	81	100.00

ga014_s25: Estate Planning: Child[25]'s Children

	Freq.	%
0 No	81	100.00
Total	81	100.00

ga014_s26: Estate Planning: Other Child[26]'s Children

	Freq.	%
0 No	68	83.95
26 Yes	13	16.05
Total	81	100.00

ga014_s27: Estate Planning: Other Child[27]'s Children

	Freq.	%
0 No	81	100.00
Total	81	100.00

ga014_s28: Estate Planning: Other Child[28]'s Children

	Freq.	%
0 No	81	100.00
Total	81	100.00

ga014_s29: Estate Planning: Other Child[29]'s Children

	Freq.	%
0 No	81	100.00
Total	81	100.00

ga014_s30: Estate Planning: Other Child[30]'s Children

	Freq.	%
0 No	81	100.00

Total	81	100.00

ga014_s31: Estate Planning: Other Child[31]'s Children

	Freq.	%
0 No	81	100.00
Total	81	100.00

ga014_s32: Estate Planning: Other Child[32]'s Children

	Freq.	%
0 No	81	100.00
Total	81	100.00

ga014_s33: Estate Planning: Other Child[33]'s Children

	Freq.	%
0 No	81	100.00
Total	81	100.00

ga014_s34: Estate Planning: Other Child[34]'s Children

	Freq.	%
0 No	81	100.00
Total	81	100.00

ga014_s35: Estate Planning: Other Child[35]'s Children

	Freq.	%
0 No	81	100.00
Total	81	100.00

ga015_1_: Estate Planning: Child[1]'s Children(%)

Mean	SD	Min	Max	Obs
49.86	41.85	-1.00	100.00	53

ga015_2_: Estate Planning: Child[2]'s Children(%)

Mean	SD	Min	Max	Obs
38.36	39.54	-1.00	100.00	31

ga015_3_: Estate Planning: Child[3]'s Children(%)

Mean	SD	Min	Max	Obs
7.25	12.47	-1.00	33.30	13

ga015_4_: Estate Planning: Child[4]'s Children(%)

Mean	SD	Min	Max	Obs
39.67	53.29	-1.00	100.00	3

ga015_5_: Estate Planning: Child[5]'s Children(%)

Mean	SD	Min	Max	Obs
100.00	.	100.00	100.00	1

ga015_6_: Estate Planning: Child[6]'s Children(%)

No Observations

ga015_7_: Estate Planning: Child[7]'s Children(%)

No Observations

ga015_8_: Estate Planning: Child[8]'s Children(%)

No Observations

ga015_9_: Estate Planning: Child[9]'s Children(%)

No Observations

ga015_10_: Estate Planning: Child[10]'s Children(%)

No Observations

ga015_11_: Estate Planning: Child[11]'s Children(%)

No Observations

ga015_12_: Estate Planning: Child[12]'s Children(%)

No Observations

ga015_13_: Estate Planning: Child[13]'s Children(%)

No Observations

ga015_14_: Estate Planning: Child[14]'s Children(%)

No Observations

ga015_15_: Estate Planning: Child[15]'s Children(%)

No Observations

ga015_16_: Estate Planning: Child[16]'s Children(%)

No Observations

ga015_17_: Estate Planning: Child[17]'s Children(%)

No Observations

ga015_18_: Estate Planning: Child[18]'s Children(%)

No Observations

ga015_19_: Estate Planning: Child[19]'s Children(%)

No Observations

ga015_20_: Estate Planning: Child[20]'s Children(%)

No Observations

ga015_21_: Estate Planning: Child[21]'s Children(%)

No Observations

ga015_22_: Estate Planning: Child[22]'s Children(%)

No Observations

ga015_23_: Estate Planning: Child[23]'s Children(%)

No Observations

ga015_24_: Estate Planning: Child[24]'s Children(%)

No Observations

ga015_25_: Estate Planning: Child[25]'s Children(%)

No Observations

ga015_26_: Estate Planning: Other Child[26]'s Children(%)

Mean	SD	Min	Max	Obs

83.00	31.42	-1.00	100.00	13

ga015_27_: Estate Planning: Other Child[27]'s Children(%)

No Observations

ga015_28_: Estate Planning: Other Child[28]'s Children(%)

No Observations

ga015_29_: Estate Planning: Other Child[29]'s Children(%)

No Observations

ga015_30_: Estate Planning: Other Child[30]'s Children(%)

No Observations

ga015_31_: Estate Planning: Other Child[31]'s Children(%)

No Observations

ga015_32_: Estate Planning: Other Child[32]'s Children(%)

No Observations

ga015_33_: Estate Planning: Other Child[33]'s Children(%)

No Observations

ga015_34_: Estate Planning: Other Child[34]'s Children(%)

No Observations

ga015_35_: Estate Planning: Other Child[35]'s Children(%)

No Observations

ga016: Actual Allocation Time

	Freq.	%
1 The Respondent Died	139	46.80
2 The Respondent Alive	158	53.20
Total	297	100.00

ga018: Actual Allocation Time

	Freq.	%
1 Both (Respondent&Spouse) Died	517	45.75
2 Both (Respondent&Spouse) Alive	442	39.12
3 One (Respondent/Spouse) Died	171	15.13
Total	1,130	100.00

xproxytext: Text for Proxy

No Observations

xga004text: Text for ga004

A String Variable	
Obs:	4,020

xga009text: Text for ga009

A String Variable	
Obs:	88

xchildnum: Num of Children

Mean	SD	Min	Max	Obs
2.49	1.31	0.00	10.00	19,349

versionID: Version ID

A String Variable	
Obs:	19,349

MODULE V

COVID Module

ID: Individual ID

A String Variable	
Obs:	19,383

householdID: Household ID

A String Variable	
Obs:	19,383

communityID: Community ID

A String Variable	
Obs:	19,383

va001_s1: Washing Hands

	Freq.	%
0 No	3,927	20.27
1 Yes	15,449	79.73
Total	19,376	100.00

va001_s2: Using Disinfectant

	Freq.	%
0 No	5,310	27.41
2 Yes	14,066	72.59
Total	19,376	100.00

va001_s3: Avoid Handshaking

	Freq.	%
0 No	6,188	31.94
3 Yes	13,188	68.06
Total	19,376	100.00

va001_s4: Masking

	Freq.	%
0 No	1,994	10.29
4 Yes	17,382	89.71
Total	19,376	100.00

va001_s5: Avoid Travels

	Freq.	%
0 No	4,771	24.62
5 Yes	14,605	75.38
Total	19,376	100.00

va001_s6: Avoid Gatherings

	Freq.	%
0 No	3,252	16.78
6 Yes	16,124	83.22
Total	19,376	100.00

va001_s7: Social Distancing

	Freq.	%
0 No	4,452	22.98
7 Yes	14,924	77.02
Total	19,376	100.00

va001_s8: Others

	Freq.	%
0 No	16,335	84.31
8 Yes	3,041	15.69
Total	19,376	100.00

va001_s9: Do not Know About the Pandemic or Preventive Measures

	Freq.	%
0 No	18,728	96.66
9 Yes	648	3.34
Total	19,376	100.00

va002_s1: Televisions

	Freq.	%
0 No	6,318	34.97
1 Yes	11,748	65.03
Total	18,066	100.00

va002_s2: Newspaper

	Freq.	%
0 No	17,446	96.57
2 Yes	620	3.43
Total	18,066	100.00

va002_s3: Internet

	Freq.	%
0 No	12,069	66.81
3 Yes	5,997	33.19
Total	18,066	100.00

va002_s4: Radio

	Freq.	%
0 No	17,315	95.84
4 Yes	751	4.16
Total	18,066	100.00

va002_s5: Friends, Colleagues, Community

	Freq.	%
0 No	8,742	48.39
5 Yes	9,324	51.61
Total	18,066	100.00

va002_s6: Medical practitioners

	Freq.	%
0 No	17,140	94.87
6 Yes	926	5.13
Total	18,066	100.00

va002_s7: Village broadcasts

	Freq.	%
0 No	13,750	76.11
7 Yes	4,316	23.89
Total	18,066	100.00

va002_s8: Posters

	Freq.	%
0 No	16,237	89.88
8 Yes	1,829	10.12
Total	18,066	100.00

va002_s9: Others

	Freq.	%
0 No	17,443	96.55
9 Yes	623	3.45
Total	18,066	100.00

va002_s10: No External Sources

	Freq.	%
0 No	17,556	97.18
10 Yes	510	2.82
Total	18,066	100.00

va003: Masking During the Pandemic

	Freq.	%
Always	14,459	74.63
Sometimes	2,513	12.97
Never	931	4.81
Did not Go Out During the Pandemic	1,472	7.60
Total	19,375	100.00

va004: Masking Nowadays

	Freq.	%
Yes	210	14.27
Still Not	586	39.81
Do not Go Out	676	45.92
Total	1,472	100.00

va005: Masking Nowadays

	Freq.	%
Always	7,985	44.60
Not After the Pandemic	8,848	49.42
Never	932	5.21
Do not Go Out After the Pandemic	137	0.77
Total	17,902	100.00

va005_1: For How Long

Mean	SD	Min	Max	Obs
2.23	1.15	0.00	8.00	8,645

va006_s1: Food

	Freq.	%
0 No	15,668	80.87
1 Yes	3,706	19.13
Total	19,374	100.00

va006_s2: Masks and disinfectants

	Freq.	%
0 No	11,605	59.90
2 Yes	7,769	40.10
Total	19,374	100.00

va006_s3: None

	Freq.	%
0 No	8,417	43.44
3 Yes	10,957	56.56
Total	19,374	100.00

va007: Attitude Towards Government Interventions

	Freq.	%
1 Stricter Than Necessary	2,924	15.09
2 Satisfied	14,252	73.56
3 Less Strict Than Necessary	1,158	5.98
997 Do not Know	1,022	5.28
999 Refuse to Answer	18	0.09
Total	19,374	100.00

vb001_s1: Self

	Freq.	%
0 No	19,372	99.99
1 Yes	2	0.01
Total	19,374	100.00

vb001_s2: Cohabitant

	Freq.	%
0 Yes	19,374	100.00
Total	19,374	100.00

vb001_s3: Other Relatives

	Freq.	%
0 No	19,363	99.94
3 Yes	11	0.06

	Freq.	%
Total	19,374	100.00

vb001_s4: Acquaintance

	Freq.	%
0 No	19,327	99.76
4 Yes	47	0.24
Total	19,374	100.00

vb001_s5: None

	Freq.	%
0 No	127	0.66
5 Yes	19,247	99.34
Total	19,374	100.00

vb001_s999: Refuse to Answer

	Freq.	%
0 No	19,304	99.64
999 Yes	70	0.36
Total	19,374	100.00

vb002_s1: Cohabitant

	Freq.	%
0 Yes	57	100.00
Total	57	100.00

vb002_s2: Other Relatives

	Freq.	%
0 No	56	98.25
2 Yes	1	1.75
Total	57	100.00

vb002_s3: Acquaintance

	Freq.	%
0 No	47	82.46
3 Yes	10	17.54
Total	57	100.00

vb002_s4: None

	Freq.	%
0 No	12	21.05
4 Yes	45	78.95
Total	57	100.00

vb002_s999: Refuse to Answer

	Freq.	%
0 No	55	96.49
999 Yes	2	3.51
Total	57	100.00

vb004: Days of Hospital Stay for COVID treatment

Mean	SD	Min	Max	Obs
0.00	0.00	0.00	0.00	2

vb005_s1: Travels

	Freq.	%
0 No	19,205	99.13
1 Yes	169	0.87
Total	19,374	100.00

vb005_s2: Close contact of COVID cases

	Freq.	%
0 No	19,355	99.90
2 Yes	19	0.10
Total	19,374	100.00

vb005_s3: Building Lockdown

	Freq.	%
0 No	19,234	99.28
3 Yes	140	0.72
Total	19,374	100.00

vb005_s4: After Going to Hospital

	Freq.	%
0 No	19,335	99.80
4 Yes	39	0.20
Total	19,374	100.00

vb005_s5: Tested Positive

	Freq.	%
0 Yes	19,374	100.00
Total	19,374	100.00

vb005_s6: No Quarantine Experience

	Freq.	%
0 No	425	2.19
6 Yes	18,949	97.81
Total	19,374	100.00

vb005_s997: Do not Know

	Freq.	%
0 No	19,308	99.66
997 Yes	66	0.34
Total	19,374	100.00

vb005_s999: Refuse to Answer

	Freq.	%
0 Yes	19,374	100.00
Total	19,374	100.00

vb008: Days of Quarantine

Mean	SD	Min	Max	Obs
24.28	23.85	1.00	210.00	353

vb009_s1: Hospital

	Freq.	%
0 No	344	95.82
1 Yes	15	4.18
Total	359	100.00

vb009_s2: Hotel

	Freq.	%
0 No	336	93.59
2 Yes	23	6.41
Total	359	100.00

vb009_s3: Own Residence

	Freq.	%
0 No	64	17.83
3 Yes	295	82.17
Total	359	100.00

vb009_s4: Others

	Freq.	%

	Freq.	%
0 No	327	91.09
4 Yes	32	8.91
Total	359	100.00

vb009_s997: Do not Know

	Freq.	%
0 No	357	99.44
997 Yes	2	0.56
Total	359	100.00

vb009_s999: Refuse to Answer

	Freq.	%
0 Yes	359	100.00
Total	359	100.00

vb010: Payment of Quarantine

Mean	SD	Min	Max	Obs
31.74	94.32	0.00	350.00	23

vb011: Quarantined with Someone Else in Same Place

	Freq.	%
1 Yes	260	72.42
2 No	97	27.02
997 Do not Know	2	0.56
Total	359	100.00

vb012: COVID Tested

	Freq.	%
1 Yes	1,042	5.38
2 No	18,244	94.17
997 Do not Know	87	0.45
999 Refuse to Answer	1	0.01
Total	19,374	100.00

vb012_1: Year of Last Test

Mean	SD	Min	Max	Obs
2,020.00	0.00	2,020.00	2,020.00	1,042

vb012_2: Month

Mean	SD	Min	Max	Obs
5.44	1.83	1.00	8.00	1,014

vc000: Days of Longest Self-Isolation During the Pandemic

Mean	SD	Min	Max	Obs
19.40	28.73	0.00	240.00	19,020

vc001: Residence During the Lunar New Year Outbreak

	Freq.	%
1 Same as the Residence	17,405	89.85
2 Another Neighborhood but Same County as the Residence	710	3.67
3 Other Domestic Location	1,234	6.37
4 Abroad	16	0.08
999 Refuse to Answer	7	0.04
Total	19,372	100.00

vc002: Times of Going Outdoors During the Lunar New Year Outbreak

	Freq.	%
Increased Greatly	111	0.57
Increased Slightly	66	0.34
Not Changed	6,558	33.85
Decreased Slightly	1,747	9.02
Decreased Greatly	10,890	56.22
Total	19,372	100.00

vc003: Time Spent Outdoors During the Lunar New Year Outbreak

	Freq.	%
Increased Greatly	158	0.82
Increased Slightly	94	0.49
Not Changed	6,735	34.77
Decreased Slightly	1,848	9.54
Decreased Greatly	10,536	54.39
Total	19,371	100.00

vc004: Intense Activities During the Lunar New Year Outbreak

	Freq.	%
Increased Greatly	132	0.68
Increased Slightly	128	0.66
Not Changed	13,371	69.03
Decreased Slightly	1,238	6.39
Decreased Greatly	4,502	23.24
Total	19,371	100.00

vc005: Moderate Activities During the Lunar New Year Outbreak

	Freq.	%
Increased Greatly	438	2.26
Increased Slightly	390	2.01
Not Changed	13,593	70.17

Decreased Slightly	1,417	7.32
Decreased Greatly	3,533	18.24
Total	19,371	100.00

vc006: Light Activities During the Lunar New Year Outbreak

	Freq.	%
Increased Greatly	275	1.42
Increased Slightly	317	1.64
Not Changed	9,717	50.16
Decreased Slightly	2,373	12.25
Decreased Greatly	6,689	34.53
Total	19,371	100.00

vc007: Visiting Others During the Lunar New Year Outbreak

	Freq.	%
Increased Greatly	52	0.27
Increased Slightly	39	0.20
Not Changed	3,570	18.43
Decreased Slightly	1,464	7.56
Decreased Greatly	9,104	47.00
Would not Visit Others Anyway	5,141	26.54
Total	19,370	100.00

vc008: Playing Mahjong and Cards During the Lunar New Year Outbreak

	Freq.	%
Increased Greatly	64	0.33
Increased Slightly	66	0.34
Not Changed	1,232	6.36
Decreased Slightly	430	2.22
Decreased Greatly	3,625	18.71
Would not Play Anyway	13,953	72.03
Total	19,370	100.00

vc009: Dancing Outdoors During the Lunar New Year Outbreak

	Freq.	%
Increased Greatly	15	0.08
Increased Slightly	16	0.08
Not Changed	631	3.26
Decreased Slightly	166	0.86
Decreased Greatly	1,486	7.67
Would not Dance Outdoors Anyway	17,056	88.05
Total	19,370	100.00

vc010: Calling and Messaging During the Lunar New Year Outbreak

	Freq.	%
Increased Greatly	1,992	10.28
Increased Slightly	1,751	9.04

	Freq.	%
Not Changed	10,050	51.88
Decreased Slightly	1,061	5.48
Decreased Greatly	1,863	9.62
No Device at Home	143	0.74
Would not Do This Anyway	2,510	12.96
Total	19,370	100.00

vc011: Making Internet Contact During the Lunar New Year Outbreak

	Freq.	%
Increased Greatly	1,709	8.82
Increased Slightly	1,588	8.20
Not Changed	5,474	28.26
Decreased Slightly	545	2.81
Decreased Greatly	769	3.97
No Device at Home	818	4.22
Would not Do This Anyway	8,467	43.71
Total	19,370	100.00

vc012: Feeling Fears During the Lunar New Year Outbreak

	Freq.	%
Rarely or Never	11,414	58.93
Not often	1,862	9.61
Sometimes	2,784	14.37
Often Times	3,097	15.99
997 Do not Know	209	1.08
999 Refuse to Answer	4	0.02
Total	19,370	100.00

vc013: Feeling Anxiety During the Lunar New Year Outbreak

	Freq.	%
Rarely or Never	12,073	62.33
Not often	1,947	10.05
Sometimes	2,839	14.66
Often Times	2,279	11.77
997 Do not Know	228	1.18
999 Refuse to Answer	4	0.02
Total	19,370	100.00

vc014: Smoking During the Lunar New Year Outbreak

	Freq.	%
Increased Greatly	182	0.94
Increased Slightly	344	1.78
Not Changed	4,197	21.67
Decreased Slightly	414	2.14
Decreased Greatly	601	3.10
Would not Smoke Anyway	13,632	70.38
Total	19,370	100.00

vc015: Drinking During the Lunar New Year Outbreak

	Freq.	%
Increased Greatly	73	0.38
Increased Slightly	240	1.24
Not Changed	4,741	24.48
Decreased Slightly	554	2.86
Decreased Greatly	1,216	6.28
Would not Drink Anyway	12,546	64.77
Total	19,370	100.00

vc016: Sleeping During the Lunar New Year Outbreak

	Freq.	%
Increased Greatly	919	4.74
Increased Slightly	967	4.99
Not Changed	15,262	78.80
Decreased Slightly	1,295	6.69
Decreased Greatly	926	4.78
Total	19,369	100.00

vc017: Eating During the Lunar New Year Outbreak

	Freq.	%
Increased Greatly	162	0.84
Increased Slightly	372	1.92
Not Changed	16,564	85.52
Decreased Slightly	1,542	7.96
Decreased Greatly	728	3.76
Total	19,368	100.00

vd001_s1: Residential Area Completely Shut Down

	Freq.	%
0 No	9,124	47.11
1 Yes	10,244	52.89
Total	19,368	100.00

vd001_s2: Restricted Entry or Exit for Residents

	Freq.	%
0 No	9,491	49.00
2 Yes	9,877	51.00
Total	19,368	100.00

vd001_s3: No Entry Into Residential Area for Non-Residents

	Freq.	%
0 No	8,180	42.23
3 Yes	11,188	57.77
Total	19,368	100.00

vd001_s4: Restricted Entry for Non-Residents

	Freq.	%
0 No	10,870	56.12
4 Yes	8,498	43.88
Total	19,368	100.00

vd001_s5: No Restrictions on Entry and Exit

	Freq.	%
0 No	17,887	92.35
5 Yes	1,481	7.65
Total	19,368	100.00

vd001_s997: Do not Know

	Freq.	%
0 No	18,439	95.20
997 Yes	929	4.80
Total	19,368	100.00

vd001_s999: Refuse to Answer

	Freq.	%
0 No	19,303	99.66
999 Yes	65	0.34
Total	19,368	100.00

vd001_1: Days of Isolation at This Level

Mean	SD	Min	Max	Obs
41.81	23.64	1.00	120.00	9,657

vd001_2: Days of Isolation at This Level

Mean	SD	Min	Max	Obs
40.39	24.69	1.00	200.00	9,177

vd001_3: Days of Isolation at This Level

Mean	SD	Min	Max	Obs
42.26	26.82	1.00	300.00	10,150

vd001_4: Days of Isolation at This Level

Mean	SD	Min	Max	Obs
45.27	32.85	1.00	300.00	7,185

vd002: Days of Outdoor Dancing Cancellation

Mean	SD	Min	Max	Obs
46.13	60.03	0.00	250.00	15,304

vd003: Days of Cancellation of Public Recreation Playing Mahjong and Cards

Mean	SD	Min	Max	Obs
47.22	57.52	0.00	250.00	14,637

xquarantined: Ever Quarantined

Mean	SD	Min	Max	Obs
0.02	0.13	0.00	1.00	19,374

xvcnotinquarantine: String constant: During the Pandemic but Excluding Quarantine Time

A String Variable	
Obs:	359

xvcoutbreak: String constant: During the Lunar New Year Outbreak

A String Variable	
Obs:	19,374

xiwmonth: Interview Month

Mean	SD	Min	Max	Obs
7.52	0.51	6.00	11.00	19,383

xiwyear: Interview Year

Mean	SD	Min	Max	Obs
2,020.00	0.04	2,017.00	2,020.00	19,383

proxy_14: Answered by a Proxy

	Freq.	%
1 Yes	1,705	99.07
2 No	16	0.93
Total	1,721	100.00

versionID: Version ID

A String Variable

Obs:	19,383

MODULE EX

Exit Module

ID: Individual ID

A String Variable	
Obs:	770

householdID: Household ID

A String Variable	
Obs:	770

communityID: Community ID

A String Variable	
Obs:	770

exb001_1: Year of Death

Mean	SD	Min	Max	Obs
2,018.16	1.69	2,011.00	2,020.00	107

exb001_2: Month of Death

Mean	SD	Min	Max	Obs
6.36	3.88	1.00	12.00	107

exb001_3: Day of Death

Mean	SD	Min	Max	Obs
10.65	9.31	1.00	31.00	107

exb002: Solar Calendar or Lunar Calendar

	Freq.	%
1 Solar calendar	588	76.46
2 Lunar calendar	181	23.54
Total	769	100.00

exb003: Residence, Before Death

	Freq.	%
1 Chinese Mainland	769	100.00
Total	769	100.00

exb004: Type of Residence, Before Death

	Freq.	%
1 Family Housing	702	91.41
2 Workplace	3	0.39
3 Nursing home	12	1.56
4 Hospital	48	6.25
5 Other, Please Specify	3	0.39
Total	768	100.00

exb005: Address of Death

	Freq.	%
1 Residence, Before Death	666	86.72
2 Chinese Mainland	102	13.28
Total	768	100.00

exb006: Type of Address of Death

	Freq.	%
1 Home	601	78.26
2 Workplace	5	0.65
3 Hospital	131	17.06
4 On the Way to the Hospital	4	0.52
5 Nursing home	9	1.17
6 Hospice	1	0.13
7 Other, Please Specify	17	2.21
Total	768	100.00

exb007: Was the Death Expected at About the Time It Occurred?

	Freq.	%
1 Expected	365	47.53
2 Unexpected	396	51.56
3 Other, Please Specify	7	0.91
Total	768	100.00

exb008: How Long Is the Time Between the Start of the Final Illness and Death?

	Freq.	%
1 One or two hours (or no warning)	86	11.20
2 Less Than A Day	43	5.60
3 Less Than A Week	53	6.90
4 Less Than A Month	118	15.36
5 Less Than A Year	231	30.08
6 More Than A Year	237	30.86
Total	768	100.00

exb009: Marital Status at the Time of R Died

	Freq.	%
1 Married	461	60.10
2 Separated	4	0.52
3 Divorced	13	1.69
4 Widowed	271	35.33
5 Never Married	16	2.09
6 Cohabitation	2	0.26
Total	767	100.00

exb010: Live With His/Her Spouse or Cohabitant When R Died

	Freq.	%
Yes	428	92.44
2 No	35	7.56
Total	463	100.00

exb011: Death Certificate

	Freq.	%
1 Yes	486	63.36
2 No	281	36.64
Total	767	100.00

exb012: Where Did R's Death Certificate Filed

	Freq.	%
1 Residence, Before Death	375	77.16
2 Other Community of Residence, Before Death	41	8.44
3 Other, Please Specify	70	14.40
Total	486	100.00

exb013: Household Registration Cancelled

	Freq.	%
1 Yes	641	83.79
2 No	124	16.21
Total	765	100.00

exb014: Reason for Not Canceling Household Registration

	Freq.	%
1 Time Is too Short to Deal with the Cancellation	51	41.13
2 The Cancellation Is Not Important	26	20.97
3 Other, Please Specify	47	37.90
Total	124	100.00

exc001_1_: Is ZChildName[1] Still Alive

	Freq.	%
1 Yes	681	98.27
2 No	12	1.73
Total	693	100.00

exc001_2_: Is ZChildName[2] Still Alive

	Freq.	%
1 Yes	621	98.26
2 No	11	1.74
Total	632	100.00

exc001_3_: Is ZChildName[3] Still Alive

	Freq.	%
1 Yes	471	98.74
2 No	6	1.26
Total	477	100.00

exc001_4_: Is ZChildName[4] Still Alive

	Freq.	%
1 Yes	321	98.77
2 No	4	1.23
Total	325	100.00

exc003_1_: XEChildAliveName[1]'s Birth Year

Mean	SD	Min	Max	Obs
1,979.00	24.04	1,962.00	1,996.00	2

exc003_2_: XEChildAliveName[2]'s Birth Year

Mean	SD	Min	Max	Obs
1,974.75	12.63	1,960.00	1,988.00	4

exc003_3_: XEChildAliveName[3]'s Birth Year

Mean	SD	Min	Max	Obs

1,969.00	14.93	1,958.00	1,986.00	3

exc003_4_: XEChildAliveName[4]'s Birth Year

Mean	SD	Min	Max	Obs
1,968.50	3.32	1,966.00	1,973.00	4

exc003_5_: XEChildAliveName[5]'s Birth Year

Mean	SD	Min	Max	Obs
1,966.67	7.64	1,960.00	1,975.00	3

exc003_6_: XEChildAliveName[6]'s Birth Year

Mean	SD	Min	Max	Obs
1,967.00	.	1,967.00	1,967.00	1

exc003_7_: XEChildAliveName[7]'s Birth Year

Mean	SD	Min	Max	Obs
1,950.00	.	1,950.00	1,950.00	1

exc003_8_: XEChildAliveName[8]'s Birth Year

Mean	SD	Min	Max	Obs
1,953.00	.	1,953.00	1,953.00	1

exc003_9_: XEChildAliveName[9]'s Birth Year

Mean	SD	Min	Max	Obs
1,960.00	.	1,960.00	1,960.00	1

exc004_1_: XEChildAliveName[1]'s Gender

	Freq.	%
1 Male	499	73.27
2 Female	182	26.73
Total	681	100.00

exc004_2_: XEChildAliveName[2]'s Gender

	Freq.	%
1 Male	341	54.91
2 Female	280	45.09
Total	621	100.00

exc004_3_: XEChildAliveName[3]'s Gender

	Freq.	%
1 Male	209	44.37
2 Female	262	55.63
Total	471	100.00

exc004_4_: XEChildAliveName[4]'s Gender

	Freq.	%
1 Male	123	38.32
2 Female	198	61.68
Total	321	100.00

exc004_5_: XEChildAliveName[5]'s Gender

	Freq.	%
1 Male	56	30.27
2 Female	129	69.73
Total	185	100.00

exc004_6_: XEChildAliveName[6]'s Gender

	Freq.	%
1 Male	19	21.84
2 Female	68	78.16
Total	87	100.00

exc004_7_: XEChildAliveName[7]'s Gender

	Freq.	%
1 Male	13	29.55
2 Female	31	70.45
Total	44	100.00

exc004_8_: XEChildAliveName[8]'s Gender

	Freq.	%
1 Male	8	53.33
2 Female	7	46.67
Total	15	100.00

exc004_9_: XEChildAliveName[9]'s Gender

	Freq.	%
1 Male	6	50.00
2 Female	6	50.00
Total	12	100.00

exc004_10_: XEChildAliveName[10]'s Gender

	Freq.	%
1 Male	2	33.33
2 Female	4	66.67
Total	6	100.00

exc004_11_: XEChildAliveName[11]'s Gender

	Freq.	%
1 Male	1	33.33
2 Female	2	66.67
Total	3	100.00

exc004_12_: XEChildAliveName[12]'s Gender

	Freq.	%
2 Female	1	100.00
Total	1	100.00

exc005_1_: XEChildAliveName[1]'s Highest Degree

	Freq.	%
2 Did not Finish Primary School	8	12.70
4 Elementary School	10	15.87
5 Middle School	12	19.05
6 High School	10	15.87
7 Vocational School	6	9.52
8 Two-/Three-Year College/Associate Degree	6	9.52
9 Four-Year College/Bachelor's Degree	10	15.87
997 Don't Know	1	1.59
Total	63	100.00

exc005_2_: XEChildAliveName[2]'s Highest Degree

	Freq.	%
1 No Formal Education(Illiterate)	3	4.29
2 Did not Finish Primary School	12	17.14
4 Elementary School	10	14.29
5 Middle School	19	27.14
6 High School	12	17.14
7 Vocational School	4	5.71
8 Two-/Three-Year College/Associate Degree	2	2.86
9 Four-Year College/Bachelor's Degree	5	7.14
10 Master's Degree	1	1.43
997 Don't Know	2	2.86
Total	70	100.00

exc005_3_: XEChildAliveName[3]'s Highest Degree

	Freq.	%
1 No Formal Education(Illiterate)	8	16.67

2 Did not Finish Primary School	3	6.25
4 Elementary School	16	33.33
5 Middle School	12	25.00
6 High School	3	6.25
7 Vocational School	2	4.17
9 Four-Year College/Bachelor's Degree	2	4.17
997 Don't Know	2	4.17
Total	48	100.00

exc005_4_: XEChildAliveName[4]'s Highest Degree

	Freq.	%
1 No Formal Education(Illiterate)	5	16.67
2 Did not Finish Primary School	1	3.33
4 Elementary School	9	30.00
5 Middle School	10	33.33
6 High School	3	10.00
8 Two-/Three-Year College/Associate Degree	1	3.33
9 Four-Year College/Bachelor's Degree	1	3.33
Total	30	100.00

exc005_5_: XEChildAliveName[5]'s Highest Degree

	Freq.	%
1 No Formal Education(Illiterate)	3	13.64
2 Did not Finish Primary School	2	9.09
4 Elementary School	6	27.27
5 Middle School	9	40.91
6 High School	1	4.55
8 Two-/Three-Year College/Associate Degree	1	4.55
Total	22	100.00

exc005_6_: XEChildAliveName[6]'s Highest Degree

	Freq.	%
1 No Formal Education(Illiterate)	2	20.00
2 Did not Finish Primary School	2	20.00
4 Elementary School	3	30.00
5 Middle School	2	20.00
9 Four-Year College/Bachelor's Degree	1	10.00
Total	10	100.00

exc005_7_: XEChildAliveName[7]'s Highest Degree

	Freq.	%
4 Elementary School	3	50.00
5 Middle School	2	33.33
997 Don't Know	1	16.67
Total	6	100.00

exc005_8_: XEChildAliveName[8]'s Highest Degree

	Freq.	%
1 No Formal Education(Illiterate)	1	20.00
2 Did not Finish Primary School	1	20.00
5 Middle School	1	20.00
8 Two-/Three-Year College/Associate Degree	1	20.00
997 Don't Know	1	20.00
Total	5	100.00

exc005_9_: XEChildAliveName[9]'s Highest Degree

	Freq.	%
1 No Formal Education(Illiterate)	1	20.00
2 Did not Finish Primary School	1	20.00
5 Middle School	2	40.00
997 Don't Know	1	20.00
Total	5	100.00

exc005_10_: XEChildAliveName[10]'s Highest Degree

	Freq.	%
5 Middle School	2	100.00
Total	2	100.00

exc005_11_: XEChildAliveName[11]'s Highest Degree

	Freq.	%
5 Middle School	2	100.00
Total	2	100.00

exc006_1_: Number of Months That XEChildAliveName[1] Lived With XRName

Mean	SD	Min	Max	Obs
4.41	5.29	0.00	12.00	681

exc006_2_: Number of Months That XEChildAliveName[2] Lived With XRName

Mean	SD	Min	Max	Obs
2.44	4.34	0.00	12.00	621

exc006_3_: Number of Months That XEChildAliveName[3] Lived With XRName

Mean	SD	Min	Max	Obs
1.78	3.79	0.00	12.00	469

exc006_4_: Number of Months That XEChildAliveName[4] Lived With XRName

Mean	SD	Min	Max	Obs

1.46	3.46	0.00	12.00	321

exc006_5_: Number of Months That XEChildAliveName[5] Lived With XRName

Mean	SD	Min	Max	Obs
1.56	3.57	0.00	12.00	185

exc006_6_: Number of Months That XEChildAliveName[6] Lived With XRName

Mean	SD	Min	Max	Obs
0.71	2.33	0.00	12.00	87

exc006_7_: Number of Months That XEChildAliveName[7] Lived With XRName

Mean	SD	Min	Max	Obs
1.45	3.71	0.00	12.00	44

exc006_8_: Number of Months That XEChildAliveName[8] Lived With XRName

Mean	SD	Min	Max	Obs
1.63	4.21	0.00	12.00	15

exc006_9_: Number of Months That XEChildAliveName[9] Lived With XRName

Mean	SD	Min	Max	Obs
0.25	0.87	0.00	3.00	12

exc006_10_: Number of Months That XEChildAliveName[10] Lived With XRName

Mean	SD	Min	Max	Obs
0.00	0.00	0.00	0.00	6

exc006_11_: Number of Months That XEChildAliveName[11] Lived With XRName

Mean	SD	Min	Max	Obs
4.00	6.93	0.00	12.00	3

exc006_12_: Number of Months That XEChildAliveName[12] Lived With XRName

Mean	SD	Min	Max	Obs
0.00	.	0.00	0.00	1

exc007_1_: How Often XRName See XEChildAliveName[1]

	Freq.	%
1 Almost Every Day	82	17.19

2 2-3 Times a Week	43	9.01
3 Once a week	51	10.69
4 Every Two Weeks	35	7.34
5 Once a Month	63	13.21
6 Once Every Three Months	41	8.60
7 Once Every Six Months	43	9.01
8 Once a Year	62	13.00
9 Almost Never	28	5.87
10 Other	29	6.08
Total	477	100.00

exc007_2_: How Often XRName See XEChildAliveName[2]

	Freq.	%
1 Almost Every Day	95	18.06
2 2-3 Times a Week	51	9.70
3 Once a week	60	11.41
4 Every Two Weeks	38	7.22
5 Once a Month	67	12.74
6 Once Every Three Months	38	7.22
7 Once Every Six Months	52	9.89
8 Once a Year	61	11.60
9 Almost Never	32	6.08
10 Other	32	6.08
Total	526	100.00

exc007_3_: How Often XRName See XEChildAliveName[3]

	Freq.	%
1 Almost Every Day	71	16.99
2 2-3 Times a Week	33	7.89
3 Once a week	45	10.77
4 Every Two Weeks	38	9.09
5 Once a Month	60	14.35
6 Once Every Three Months	40	9.57
7 Once Every Six Months	38	9.09
8 Once a Year	56	13.40
9 Almost Never	19	4.55
10 Other	18	4.31
Total	418	100.00

exc007_4_: How Often XRName See XEChildAliveName[4]

	Freq.	%
1 Almost Every Day	33	11.22
2 2-3 Times a Week	42	14.29
3 Once a week	31	10.54
4 Every Two Weeks	24	8.16
5 Once a Month	33	11.22
6 Once Every Three Months	32	10.88
7 Once Every Six Months	33	11.22
8 Once a Year	35	11.90
9 Almost Never	13	4.42
10 Other	18	6.12
Total	294	100.00

exc007_5_: How Often XRName See XEChildAliveName[5]

	Freq.	%
1 Almost Every Day	15	8.93
2 2-3 Times a Week	21	12.50
3 Once a week	14	8.33
4 Every Two Weeks	21	12.50
5 Once a Month	23	13.69
6 Once Every Three Months	21	12.50
7 Once Every Six Months	13	7.74
8 Once a Year	21	12.50
9 Almost Never	7	4.17
10 Other	12	7.14
Total	168	100.00

exc007_6_: How Often XRName See XEChildAliveName[6]

	Freq.	%
1 Almost Every Day	14	16.67
2 2-3 Times a Week	12	14.29
3 Once a week	5	5.95
4 Every Two Weeks	10	11.90
5 Once a Month	8	9.52
6 Once Every Three Months	9	10.71
7 Once Every Six Months	8	9.52
8 Once a Year	10	11.90
9 Almost Never	5	5.95
10 Other	3	3.57
Total	84	100.00

exc007_7_: How Often XRName See XEChildAliveName[7]

	Freq.	%
1 Almost Every Day	8	20.00
2 2-3 Times a Week	4	10.00
3 Once a week	2	5.00
4 Every Two Weeks	2	5.00
5 Once a Month	5	12.50
6 Once Every Three Months	7	17.50
7 Once Every Six Months	4	10.00
8 Once a Year	5	12.50
9 Almost Never	2	5.00
10 Other	1	2.50
Total	40	100.00

exc007_8_: How Often XRName See XEChildAliveName[8]

	Freq.	%
1 Almost Every Day	3	23.08
3 Once a week	1	7.69
4 Every Two Weeks	2	15.38
5 Once a Month	1	7.69
6 Once Every Three Months	5	38.46
9 Almost Never	1	7.69

Total	13	100.00

exc007_9_: How Often XRName See XEChildAliveName[9]

	Freq.	%
1 Almost Every Day	1	8.33
2 2-3 Times a Week	1	8.33
3 Once a week	1	8.33
4 Every Two Weeks	1	8.33
5 Once a Month	1	8.33
6 Once Every Three Months	4	33.33
7 Once Every Six Months	2	16.67
9 Almost Never	1	8.33
Total	12	100.00

exc007_10_: How Often XRName See XEChildAliveName[10]

	Freq.	%
1 Almost Every Day	1	16.67
4 Every Two Weeks	1	16.67
6 Once Every Three Months	3	50.00
7 Once Every Six Months	1	16.67
Total	6	100.00

exc007_11_: How Often XRName See XEChildAliveName[11]

	Freq.	%
4 Every Two Weeks	2	100.00
Total	2	100.00

exc007_12_: How Often XRName See XEChildAliveName[12]

	Freq.	%
9 Almost Never	1	100.00
Total	1	100.00

exc008: Number of Grandchildren

Mean	SD	Min	Max	Obs
5.47	4.49	0.00	25.00	762

exc009: Number of Great-Grandchildren

Mean	SD	Min	Max	Obs
2.24	4.05	0.00	25.00	762

exda001_1_: Disease[1]

	Freq.	%

	Freq.	%
1 Yes	138	19.41
2 No	573	80.59
Total	711	100.00

exda001_2_: Disease[2]

	Freq.	%
1 Yes	58	9.80
2 No	534	90.20
Total	592	100.00

exda001_3_: Disease[3]

	Freq.	%
1 Yes	86	15.17
2 No	481	84.83
Total	567	100.00

exda001_4_: Disease[4]

	Freq.	%
1 Yes	36	5.63
2 No	604	94.38
Total	640	100.00

exda001_5_: Disease[5]

	Freq.	%
1 Yes	11	1.52
2 No	713	98.48
Total	724	100.00

exda002_1_: When Disease[1] First Diagnosed

	Freq.	%
1 Year	127	92.03
2 Age	11	7.97
Total	138	100.00

exda002_1_1_: Year

Mean	SD	Min	Max	Obs
1,906.73	462.59	-1.00	2,020.00	127

exda002_2_1_: Age

Mean	SD	Min	Max	Obs
59.64	24.56	-1.00	83.00	11

exda002_2_: When Disease[2] First Diagnosed

	Freq.	%
1 Year	50	86.21
2 Age	8	13.79
Total	58	100.00

exda002_1_2_: Year

Mean	SD	Min	Max	Obs
1,652.50	782.58	-1.00	2,020.00	50

exda002_2_2_: Age

Mean	SD	Min	Max	Obs
38.63	28.88	-1.00	68.00	8

exda002_3_: When Disease[3] First Diagnosed

	Freq.	%
1 Year	75	87.21
2 Age	11	12.79
Total	86	100.00

exda002_1_3_: Year

Mean	SD	Min	Max	Obs
1,612.04	811.97	-1.00	2,020.00	75

exda002_2_3_: Age

Mean	SD	Min	Max	Obs
59.09	21.95	-1.00	82.00	11

exda002_4_: When Disease[4] First Diagnosed

	Freq.	%
1 Year	27	75.00
2 Age	9	25.00
Total	36	100.00

exda002_1_4_: Year

Mean	SD	Min	Max	Obs
1,417.48	937.98	-1.00	2,020.00	27

exda002_2_4_: Age

Mean	SD	Min	Max	Obs
66.67	16.76	39.00	86.00	9

exda002_5_: When Disease[5] First Diagnosed

	Freq.	%
1 Year	9	81.82
2 Age	2	18.18
Total	11	100.00

exda002_1_5_: Year

Mean	SD	Min	Max	Obs
1,790.22	671.72	-1.00	2,019.00	9

exda002_2_5_: Age

Mean	SD	Min	Max	Obs
26.50	38.89	-1.00	54.00	2

exda003: Had Heart Attack since Last ZIWTime

	Freq.	%
1 Yes	107	38.08
2 No	174	61.92
Total	281	100.00

exda004: When Was the Most Recent Heart Attack

	Freq.	%
1 Year	100	93.46
2 Age	7	6.54
Total	107	100.00

exda004_1: Year

Mean	SD	Min	Max	Obs
1,836.47	580.78	-1.00	2,020.00	100

exda004_2: Age

Mean	SD	Min	Max	Obs
63.00	30.53	-1.00	85.00	7

exda005_s1: Organ or Body Part have Cancer

	Freq.	%
0 No	174	92.06
1 Brain	15	7.94
Total	189	100.00

exda005_s2: Organ or Body Part have Cancer

	Freq.	%
0 No	187	98.94
2 Oral cavity	2	1.06
Total	189	100.00

exda005_s3: Organ or Body Part have Cancer

	Freq.	%
0 No	184	97.35
3 Larynx	5	2.65
Total	189	100.00

exda005_s4: Organ or Body Part have Cancer

	Freq.	%
0 No	183	96.83
4 Pharynx	6	3.17
Total	189	100.00

exda005_s5: Organ or Body Part have Cancer

	Freq.	%
0 No	186	98.41
5 Thyroid	3	1.59
Total	189	100.00

exda005_s6: Organ or Body Part have Cancer

	Freq.	%
0 No	115	60.85
6 Lung	74	39.15
Total	189	100.00

exda005_s7: Organ or Body Part have Cancer

	Freq.	%
0 No	182	96.30
7 Breast	7	3.70
Total	189	100.00

exda005_s8: Organ or Body Part have Cancer

	Freq.	%
0 No	174	92.06
8 Oesophagus	15	7.94
Total	189	100.00

exda005_s9: Organ or Body Part have Cancer

	Freq.	%
0 No	158	83.60
9 Stomach	31	16.40
Total	189	100.00

exda005_s10: Organ or Body Part have Cancer

	Freq.	%
0 No	148	78.31
10 Liver	41	21.69
Total	189	100.00

exda005_s11: Organ or Body Part have Cancer

	Freq.	%
0 No	183	96.83
11 Pancreas	6	3.17
Total	189	100.00

exda005_s12: Organ or Body Part have Cancer

	Freq.	%
0 No	184	97.35
12 Kidney	5	2.65
Total	189	100.00

exda005_s13: Organ or Body Part have Cancer

	Freq.	%
0 No	187	98.94
13 Prostate	2	1.06
Total	189	100.00

exda005_s14: Organ or Body Part have Cancer

	Freq.	%
0 No	188	99.47
14 Testicle	1	0.53
Total	189	100.00

exda005_s15: Organ or Body Part have Cancer

	Freq.	%
0 No	187	98.94
15 Ovary	2	1.06
Total	189	100.00

exda005_s16: Organ or Body Part have Cancer

	Freq.	%
0 No	187	98.94
16 Cervix	2	1.06
Total	189	100.00

exda005_s17: Organ or Body Part have Cancer

	Freq.	%
0 No	186	98.41
17 Endometrium	3	1.59
Total	189	100.00

exda005_s18: Organ or Body Part have Cancer

	Freq.	%
0 No	174	92.06
18 Colon or Rectum	15	7.94
Total	189	100.00

exda005_s19: Organ or Body Part have Cancer

	Freq.	%
0 No	186	98.41
19 Bladder	3	1.59
Total	189	100.00

exda005_s20: Organ or Body Part have Cancer

	Freq.	%
0 No	188	99.47
20 Skin	1	0.53
Total	189	100.00

exda005_s21: Organ or Body Part have Cancer

	Freq.	%
0 No	186	98.41
21 Non-Hodgkin lymphoma	3	1.59
Total	189	100.00

exda005_s22: Organ or Body Part have Cancer

	Freq.	%
0 No	185	97.88
22 Leukemia	4	2.12
Total	189	100.00

exda005_s23: Organ or Body Part have Cancer

	Freq.	%
0 No	146	77.25
23 Other organ	43	22.75
Total	189	100.00

exda006_s1: Treatments for Cancer Two Years Before Death

	Freq.	%
0 No	101	53.44
1 Taking Chinese Traditional Medicine	88	46.56
Total	189	100.00

exda006_s2: Treatments for Cancer Two Years Before Death

	Freq.	%
0 No	50	26.46
2 Taking Western Modern Medicine	139	73.54
Total	189	100.00

exda006_s3: Treatments for Cancer Two Years Before Death

	Freq.	%
0 No	125	66.14
3 Chemotherapy	64	33.86
Total	189	100.00

exda006_s4: Treatments for Cancer Two Years Before Death

	Freq.	%
0 No	133	70.37
4 Surgery	56	29.63
Total	189	100.00

exda006_s5: Treatments for Cancer Two Years Before Death

	Freq.	%
0 No	161	85.19
5 Radiation Therapy	28	14.81
Total	189	100.00

exda006_s6: Treatments for Cancer Two Years Before Death

	Freq.	%
0 No	165	87.30
6 Other Treatments, Please Specify	24	12.70
Total	189	100.00

exda006_s7: Treatments for Cancer Two Years Before Death

	Freq.	%
0 No	170	89.95
7 None of the Above	19	10.05
Total	189	100.00

exda007: Had Stroke since Last ZIWTime

	Freq.	%
1 Yes	34	21.52
2 No	124	78.48
Total	158	100.00

exda008: When Was the Most Recent Stroke

	Freq.	%
1 Year	32	94.12
2 Age	2	5.88
Total	34	100.00

exda008_1: Year

Mean	SD	Min	Max	Obs
1,702.63	744.86	-1.00	2,020.00	32

exda008_2: Age

Mean	SD	Min	Max	Obs
82.50	0.71	82.00	83.00	2

exda009: Memory Problems

	Freq.	%
1 Yes	179	23.49
2 No	583	76.51
Total	762	100.00

exda010: When Having Apparent Memory Problems

	Freq.	%
1 Year	120	67.04

	Freq.	%
2 Age	59	32.96
Total	179	100.00

exda010_1: Year

Mean	SD	Min	Max	Obs
1,831.88	584.71	-1.00	2,020.00	120

exda010_2: Age

Mean	SD	Min	Max	Obs
75.17	15.31	-1.00	97.00	59

exda011: Memory Problems Happened

	Freq.	%
1 Suddenly	36	20.11
2 Slowly	143	79.89
Total	179	100.00

exda012: Memory Problems Getting Worse

	Freq.	%
1 Yes	148	82.68
2 No	31	17.32
Total	179	100.00

exda013: Fallen Down since ZIWTime

	Freq.	%
1 Yes	245	32.15
2 No	517	67.85
Total	762	100.00

exda014: Times Falling Needing Medical Treatment

Mean	SD	Min	Max	Obs
0.70	0.90	0.00	5.00	245

exda015: Fractured Hip since ZIWTime

	Freq.	%
1 Yes	21	2.76
2 No	741	97.24
Total	762	100.00

exda016: Troubled with Body Pain

	Freq.	%
1 None	252	33.07
2 A Little	178	23.36
3 Somewhat	83	10.89
4 Quite a Bit	115	15.09
5 A Lot	134	17.59
Total	762	100.00

exda017: Other Major Illness

	Freq.	%
1 Yes	149	19.55
2 No	613	80.45
Total	762	100.00

exda019: Severe Fatigue

	Freq.	%
1 Yes	407	53.41
2 No	355	46.59
Total	762	100.00

exda020: Fetal and Urinary Incontinence

	Freq.	%
1 Yes	236	30.97
2 No	526	69.03
Total	762	100.00

exdb001: Difficulty with Dressing

	Freq.	%
1 Don't Have Any Difficulty	407	53.41
2 Have Difficulty But Can Still Do It	47	6.17
3 Have Difficulty and Need Help	126	16.54
4 Can Not Do It	182	23.88
Total	762	100.00

exdb002: Help Dress

	Freq.	%
1 Yes	332	93.52
2 No	23	6.48
Total	355	100.00

exdb003: Time Need Help with Dressing

	Freq.	%
1 Months	190	57.23

	Freq.	%
2 Years	91	27.41
3 Age	7	2.11
4 Since Specific Year	44	13.25
Total	332	100.00

exdb003_1: Months

Mean	SD	Min	Max	Obs
3.07	2.35	0.30	14.00	190

exdb003_2: Years

Mean	SD	Min	Max	Obs
3.63	4.14	0.50	27.00	91

exdb003_3: Age

Mean	SD	Min	Max	Obs
71.43	10.36	57.00	90.00	7

exdb003_4: Since Specific Year

Mean	SD	Min	Max	Obs
2,016.82	3.42	2,000.00	2,019.00	44

exdb004: Difficulty with Bathing or Showering

	Freq.	%
1 Don't Have Any Difficulty	320	41.99
2 Have Difficulty But Can Still Do It	29	3.81
3 Have Difficulty and Need Help	177	23.23
4 Can Not Do It	236	30.97
Total	762	100.00

exdb005: Help Bathing or Showering

	Freq.	%
1 Yes	406	91.86
2 No	36	8.14
Total	442	100.00

exdb006: Time Need Help with Bathing or Showering

	Freq.	%
1 Months	213	52.46
2 Years	124	30.54
3 Age	9	2.22
4 Since Specific Year	60	14.78

Total	406	100.00

exdb006_1: Months

Mean	SD	Min	Max	Obs
3.47	2.76	0.30	18.00	213

exdb006_2: Years

Mean	SD	Min	Max	Obs
3.60	4.16	0.50	27.00	124

exdb006_3: Age

Mean	SD	Min	Max	Obs
64.56	16.13	30.00	87.00	9

exdb006_4: Since Specific Year

Mean	SD	Min	Max	Obs
2,015.32	9.83	1,943.00	2,019.00	60

exdb007: Difficulty with Eating

	Freq.	%
1 Don't Have Any Difficulty	455	59.71
2 Have Difficulty But Can Still Do It	45	5.91
3 Have Difficulty and Need Help	116	15.22
4 Can Not Do It	146	19.16
Total	762	100.00

exdb008: Help Eating

	Freq.	%
1 Yes	277	90.23
2 No	30	9.77
Total	307	100.00

exdb009: Time Need Help with Eating

	Freq.	%
1 Months	181	65.34
2 Years	59	21.30
3 Age	2	0.72
4 Since Specific Year	35	12.64
Total	277	100.00

exdb009_1: Months

Mean	SD	Min	Max	Obs
2.92	2.39	0.50	14.00	181

exdb009_2: Years

Mean	SD	Min	Max	Obs
2.94	2.56	0.50	11.00	59

exdb009_3: Age

Mean	SD	Min	Max	Obs
70.00	0.00	70.00	70.00	2

exdb009_4: Since Specific Year

Mean	SD	Min	Max	Obs
2,017.09	2.24	2,010.00	2,020.00	35

exdb010: Difficulty with Getting up

	Freq.	%
1 Don't Have Any Difficulty	372	48.82
2 Have Difficulty But Can Still Do It	58	7.61
3 Have Difficulty and Need Help	156	20.47
4 Can Not Do It	176	23.10
Total	762	100.00

exdb011: Help Getting up

	Freq.	%
1 Yes	339	86.92
2 No	51	13.08
Total	390	100.00

exdb012: Time Need Help with Getting up

	Freq.	%
1 Months	214	63.13
2 Years	73	21.53
3 Age	6	1.77
4 Since Specific Year	46	13.57
Total	339	100.00

exdb012_1: Months

Mean	SD	Min	Max	Obs
3.17	2.34	0.20	14.00	214

exdb012_2: Years

Mean	SD	Min	Max	Obs
3.64	4.15	0.50	26.00	73

exdb012_3: Age

Mean	SD	Min	Max	Obs
75.17	8.40	65.00	88.00	6

exdb012_4: Since Specific Year

Mean	SD	Min	Max	Obs
2,017.43	1.96	2,010.00	2,020.00	46

exdb013: Difficulty with Using the Toilet

	Freq.	%
1 Don't Have Any Difficulty	381	50.00
2 Have Difficulty But Can Still Do It	45	5.91
3 Have Difficulty and Need Help	159	20.87
4 Can Not Do It	177	23.23
Total	762	100.00

exdb014: Help Using the Toilet

	Freq.	%
1 Yes	348	91.34
2 No	33	8.66
Total	381	100.00

exdb015: Time Need Help with Using the Toilet

	Freq.	%
1 Months	219	62.93
2 Years	73	20.98
3 Age	6	1.72
4 Since Specific Year	50	14.37
Total	348	100.00

exdb015_1: Months

Mean	SD	Min	Max	Obs
3.31	2.56	0.30	18.00	219

exdb015_2: Years

Mean	SD	Min	Max	Obs

3.45	3.74	1.00	27.00	73

exdb015_3: Age

Mean	SD	Min	Max	Obs
71.50	7.15	64.00	82.00	6

exdb015_4: Since Specific Year

Mean	SD	Min	Max	Obs
2,017.38	1.98	2,010.00	2,020.00	50

exdb016: Difficulty with Cooking

	Freq.	%
1 Don't Have Any Difficulty	306	40.16
2 Have Difficulty But Can Still Do It	19	2.49
3 Have Difficulty and Need Help	56	7.35
4 Can Not Do It	381	50.00
Total	762	100.00

exdb017: Help Cooking

	Freq.	%
1 Yes	389	85.31
2 No	67	14.69
Total	456	100.00

exdb018: Time Need Help with Cooking

	Freq.	%
1 Months	166	42.67
2 Years	151	38.82
3 Age	8	2.06
4 Since Specific Year	64	16.45
Total	389	100.00

exdb018_1: Months

Mean	SD	Min	Max	Obs
3.66	2.69	0.30	14.00	166

exdb018_2: Years

Mean	SD	Min	Max	Obs
5.99	9.19	1.00	67.00	151

exdb018_3: Age

Mean	SD	Min	Max	Obs
63.75	27.68	1.00	90.00	8

exdb018_4: Since Specific Year

Mean	SD	Min	Max	Obs
2,015.75	6.07	1,974.00	2,020.00	64

exdb019: Difficulty with Shopping for Groceries

	Freq.	%
1 Don't Have Any Difficulty	334	43.83
2 Have Difficulty But Can Still Do It	12	1.57
3 Have Difficulty and Need Help	64	8.40
4 Can Not Do It	352	46.19
Total	762	100.00

exdb020: Help Shopping for Groceries

	Freq.	%
1 Yes	375	87.62
2 No	53	12.38
Total	428	100.00

exdb021: Time Need Help with Shopping for Groceries

	Freq.	%
1 Months	154	41.07
2 Years	157	41.87
3 Age	14	3.73
4 Since Specific Year	50	13.33
Total	375	100.00

exdb021_1: Months

Mean	SD	Min	Max	Obs
3.96	2.75	0.30	18.00	154

exdb021_2: Years

Mean	SD	Min	Max	Obs
4.75	5.51	0.50	37.00	157

exdb021_3: Age

Mean	SD	Min	Max	Obs
73.57	11.61	57.00	92.00	14

exdb021_4: Since Specific Year

Mean	SD	Min	Max	Obs
2,016.04	4.00	2,000.00	2,020.00	50

exdb022: Difficulty with Making Phone Calls

	Freq.	%
1 Don't Have Any Difficulty	446	58.53
2 Have Difficulty But Can Still Do It	11	1.44
3 Have Difficulty and Need Help	59	7.74
4 Can Not Do It	246	32.28
Total	762	100.00

exdb023: Help Making Phone Calls

	Freq.	%
1 Yes	203	64.24
2 No	113	35.76
Total	316	100.00

exdb024: Time Need Help with Making Phone Calls

	Freq.	%
1 Months	78	38.42
2 Years	75	36.95
3 Age	12	5.91
4 Since Specific Year	38	18.72
Total	203	100.00

exdb024_1: Months

Mean	SD	Min	Max	Obs
3.53	2.31	0.30	14.00	78

exdb024_2: Years

Mean	SD	Min	Max	Obs
7.20	11.61	1.00	80.00	75

exdb024_3: Age

Mean	SD	Min	Max	Obs
61.50	31.17	1.00	92.00	12

exdb024_4: Since Specific Year

Mean	SD	Min	Max	Obs

2,007.61	20.86	1,925.00	2,019.00	38

exdb025: Difficulty with Taking Medications

	Freq.	%
1 Don't Have Any Difficulty	441	57.87
2 Have Difficulty But Can Still Do It	17	2.23
3 Have Difficulty and Need Help	128	16.80
4 Can Not Do It	176	23.10
Total	762	100.00

exdb026: Help Taking Medications

	Freq.	%
1 Yes	303	94.39
2 No	18	5.61
Total	321	100.00

exdb027: Time Need Help with Taking Medications

	Freq.	%
1 Months	152	50.17
2 Years	95	31.35
3 Age	12	3.96
4 Since Specific Year	44	14.52
Total	303	100.00

exdb027_1: Months

Mean	SD	Min	Max	Obs
3.97	2.44	0.50	14.00	152

exdb027_2: Years

Mean	SD	Min	Max	Obs
3.19	3.06	0.50	20.00	95

exdb027_3: Age

Mean	SD	Min	Max	Obs
77.42	10.48	57.00	92.00	12

exdb027_4: Since Specific Year

Mean	SD	Min	Max	Obs
2,016.93	2.34	2,010.00	2,020.00	44

exdb028: Difficulty with Managing Money

	Freq.	%
1 Don't Have Any Difficulty	451	59.19
2 Have Difficulty But Can Still Do It	15	1.97
3 Have Difficulty and Need Help	53	6.96
4 Can Not Do It	243	31.89
Total	762	100.00

exdb029: Help Managing Money

	Freq.	%
1 Yes	253	81.35
2 No	58	18.65
Total	311	100.00

exdb030: Time Need Help with Managing Money

	Freq.	%
1 Months	88	34.78
2 Years	106	41.90
3 Age	10	3.95
4 Since Specific Year	49	19.37
Total	253	100.00

exdb030_1: Months

Mean	SD	Min	Max	Obs
4.42	3.47	0.30	18.00	88

exdb030_2: Years

Mean	SD	Min	Max	Obs
5.18	9.21	0.50	80.00	106

exdb030_3: Age

Mean	SD	Min	Max	Obs
69.10	25.72	1.00	92.00	10

exdb030_4: Since Specific Year

Mean	SD	Min	Max	Obs
2,014.22	7.55	1,975.00	2,020.00	49

exdb031_s1: Helper[1]

	Freq.	%
0 No	294	54.75

1 Spouse	243	45.25
Total	537	100.00

exdb031_s2: Helper[2]

	Freq.	%
0 No	535	99.63
2 Father, Mother, Father-in-Law, Mother-in-Law	2	0.37
Total	537	100.00

exdb031_s3: Helper[3]

	Freq.	%
0 No	187	34.82
3 Children, Children's Spouse, Grandson, Granddaughter	350	65.18
Total	537	100.00

exdb031_s4: Helper[4]

	Freq.	%
0 No	519	96.65
4 Sibling, Brother-in-Law, Sister-in-Law, Sibling of Spouse, Etc	18	3.35
Total	537	100.00

exdb031_s5: Helper[5]

	Freq.	%
0 No	533	99.26
5 Other Relative	4	0.74
Total	537	100.00

exdb031_s6: Helper[6]

	Freq.	%
0 No	523	97.39
6 Paid Helper (Such as Nanny)	14	2.61
Total	537	100.00

exdb031_s7: Helper[7]

	Freq.	%
0 No	536	99.81
7 Volunteer or Employee of Facility	1	0.19
Total	537	100.00

exdb031_s8: Helper[8]

	Freq.	%

	Freq.	%
0 No	525	97.77
8 Nursing Home	12	2.23
Total	537	100.00

exdb031_s9: Helper[9]

	Freq.	%
0 No	536	99.81
9 Staff from Home-based Nursing Service	1	0.19
Total	537	100.00

exdb031_s10: Helper[10]

	Freq.	%
0 No	536	99.81
10 Help from the Community	1	0.19
Total	537	100.00

exdb031_s11: Helper[11]

	Freq.	%
0 No	531	98.88
11 Other, Please Specify	6	1.12
Total	537	100.00

exdb031_1: Number of Paid Helpers

Mean	SD	Min	Max	Obs
1.50	1.34	1.00	6.00	14

exdb032_s1: Who Help the Most

	Freq.	%
0 No	1	50.00
1 Father	1	50.00
Total	2	100.00

exdb032_s2: Who Help the Most

	Freq.	%
0 No	2	100.00
Total	2	100.00

exdb032_s3: Who Help the Most

	Freq.	%
0 No	2	100.00
Total	2	100.00

exdb032_s4: Who Help the Most

	Freq.	%
0 No	1	50.00
4 Mother-in-Law	1	50.00
Total	2	100.00

exdb033_s1: Which Child's Family Helped

	Freq.	%
0 No	118	33.71
1 Preload XEChildAliveName[1]	232	66.29
Total	350	100.00

exdb033_s2: Which Child's Family Helped

	Freq.	%
0 No	161	46.00
2 Preload XEChildAliveName[2]	189	54.00
Total	350	100.00

exdb033_s3: Which Child's Family Helped

	Freq.	%
0 No	199	56.86
3 Preload XEChildAliveName[3]	151	43.14
Total	350	100.00

exdb033_s4: Which Child's Family Helped

	Freq.	%
0 No	250	71.43
4 Preload XEChildAliveName[4]	100	28.57
Total	350	100.00

exdb033_s5: Which Child's Family Helped

	Freq.	%
0 No	296	84.57
5 Preload XEChildAliveName[5]	54	15.43
Total	350	100.00

exdb033_s6: Which Child's Family Helped

	Freq.	%
0 No	323	92.29
6 Preload XEChildAliveName[6]	27	7.71
Total	350	100.00

exdb033_s7: Which Child's Family Helped

	Freq.	%
0 No	336	96.00
7 Preload XEChildAliveName[7]	14	4.00
Total	350	100.00

exdb033_s8: Which Child's Family Helped

	Freq.	%
0 No	348	99.43
8 Preload XEChildAliveName[8]	2	0.57
Total	350	100.00

exdb033_s9: Which Child's Family Helped

	Freq.	%
0 No	348	99.43
9 Preload XEChildAliveName[9]	2	0.57
Total	350	100.00

exdb033_s10: Which Child's Family Helped

	Freq.	%
0 No	349	99.71
10 Preload XEChildAliveName[10]	1	0.29
Total	350	100.00

exdb033_s11: Which Child's Family Helped

	Freq.	%
0 No	349	99.71
11 Preload XEChildAliveName[11]	1	0.29
Total	350	100.00

exdb033_s12: Which Child's Family Helped

	Freq.	%
0 No	350	100.00
Total	350	100.00

exdb033_s13: Which Child's Family Helped

	Freq.	%
0 No	350	100.00
Total	350	100.00

exdb033_s14: Which Child's Family Helped

	Freq.	%
0 No	350	100.00
Total	350	100.00

exdb033_s15: Which Child's Family Helped

	Freq.	%
0 No	350	100.00
Total	350	100.00

exdb033_s16: Which Child's Family Helped

	Freq.	%
0 No	350	100.00
Total	350	100.00

exdb033_s17: Which Child's Family Helped

	Freq.	%
0 No	350	100.00
Total	350	100.00

exdb033_s18: Which Child's Family Helped

	Freq.	%
0 No	350	100.00
Total	350	100.00

exdb033_s19: Which Child's Family Helped

	Freq.	%
0 No	350	100.00
Total	350	100.00

exdb033_s20: Which Child's Family Helped

	Freq.	%
0 No	350	100.00
Total	350	100.00

exdb033_s21: Which Child's Family Helped

	Freq.	%
0 No	350	100.00
Total	350	100.00

exdb033_s22: Which Child's Family Helped

	Freq.	%
0 No	350	100.00
Total	350	100.00

exdb033_s23: Which Child's Family Helped

	Freq.	%
0 No	350	100.00
Total	350	100.00

exdb033_s24: Which Child's Family Helped

	Freq.	%
0 No	350	100.00
Total	350	100.00

exdb033_s25: Which Child's Family Helped

	Freq.	%
0 No	350	100.00
Total	350	100.00

exdb034_1__s1: Who in Child[1]'s Family Helped

	Freq.	%
0 No	15	6.47
1 Himself/herself	217	93.53
Total	232	100.00

exdb034_1__s2: Who in Child[1]'s Family Helped

	Freq.	%
0 No	74	31.90
2 His/her Spouse	158	68.10
Total	232	100.00

exdb034_1__s3: Who in Child[1]'s Family Helped

	Freq.	%
0 No	136	58.62
3 His/her Children	96	41.38
Total	232	100.00

exdb034_2__s1: Who in Child[2]'s Family Helped

	Freq.	%
0 No	10	5.29

1 Himself/herself	179	94.71
Total	189	100.00

exdb034_2__s2: Who in Child[2]'s Family Helped

	Freq.	%
0 No	74	39.15
2 His/her Spouse	115	60.85
Total	189	100.00

exdb034_2__s3: Who in Child[2]'s Family Helped

	Freq.	%
0 No	124	65.61
3 His/her Children	65	34.39
Total	189	100.00

exdb034_3__s1: Who in Child[3]'s Family Helped

	Freq.	%
0 No	5	3.31
1 Himself/herself	146	96.69
Total	151	100.00

exdb034_3__s2: Who in Child[3]'s Family Helped

	Freq.	%
0 No	58	38.41
2 His/her Spouse	93	61.59
Total	151	100.00

exdb034_3__s3: Who in Child[3]'s Family Helped

	Freq.	%
0 No	97	64.24
3 His/her Children	54	35.76
Total	151	100.00

exdb034_4__s1: Who in Child[4]'s Family Helped

	Freq.	%
0 No	3	3.00
1 Himself/herself	97	97.00
Total	100	100.00

exdb034_4__s2: Who in Child[4]'s Family Helped

	Freq.	%
0 No	40	40.00

	Freq.	%
2 His/her Spouse	60	60.00
Total	100	100.00

exdb034_4__s3: Who in Child[4]'s Family Helped

	Freq.	%
0 No	65	65.00
3 His/her Children	35	35.00
Total	100	100.00

exdb034_5__s1: Who in Child[5]'s Family Helped

	Freq.	%
0 No	1	1.85
1 Himself/herself	53	98.15
Total	54	100.00

exdb034_5__s2: Who in Child[5]'s Family Helped

	Freq.	%
0 No	29	53.70
2 His/her Spouse	25	46.30
Total	54	100.00

exdb034_5__s3: Who in Child[5]'s Family Helped

	Freq.	%
0 No	39	72.22
3 His/her Children	15	27.78
Total	54	100.00

exdb034_6__s1: Who in Child[6]'s Family Helped

	Freq.	%
0 No	3	11.11
1 Himself/herself	24	88.89
Total	27	100.00

exdb034_6__s2: Who in Child[6]'s Family Helped

	Freq.	%
0 No	9	33.33
2 His/her Spouse	18	66.67
Total	27	100.00

exdb034_6__s3: Who in Child[6]'s Family Helped

	Freq.	%
0 No	19	70.37

	Freq.	%
3 His/her Children	8	29.63
Total	27	100.00

exdb034_7__s1: Who in Child[7]'s Family Helped

	Freq.	%
1 Himself/herself	14	100.00
Total	14	100.00

exdb034_7__s2: Who in Child[7]'s Family Helped

	Freq.	%
0 No	5	35.71
2 His/her Spouse	9	64.29
Total	14	100.00

exdb034_7__s3: Who in Child[7]'s Family Helped

	Freq.	%
0 No	8	57.14
3 His/her Children	6	42.86
Total	14	100.00

exdb034_8__s1: Who in Child[8]'s Family Helped

	Freq.	%
1 Himself/herself	2	100.00
Total	2	100.00

exdb034_8__s2: Who in Child[8]'s Family Helped

	Freq.	%
0 No	1	50.00
2 His/her Spouse	1	50.00
Total	2	100.00

exdb034_8__s3: Who in Child[8]'s Family Helped

	Freq.	%
3 His/her Children	2	100.00
Total	2	100.00

exdb034_9__s1: Who in Child[9]'s Family Helped

	Freq.	%
0 No	1	50.00
1 Himself/herself	1	50.00
Total	2	100.00

exdb034_9__s2: Who in Child[9]'s Family Helped

	Freq.	%
0 No	1	50.00
2 His/her Spouse	1	50.00
Total	2	100.00

exdb034_9__s3: Who in Child[9]'s Family Helped

	Freq.	%
0 No	1	50.00
3 His/her Children	1	50.00
Total	2	100.00

exdb034_10__s1: Who in Child[10]'s Family Helped

	Freq.	%
1 Himself/herself	1	100.00
Total	1	100.00

exdb034_10__s2: Who in Child[10]'s Family Helped

	Freq.	%
0 No	1	100.00
Total	1	100.00

exdb034_10__s3: Who in Child[10]'s Family Helped

	Freq.	%
0 No	1	100.00
Total	1	100.00

exdb034_11__s1: Who in Child[11]'s Family Helped

	Freq.	%
1 Himself/herself	1	100.00
Total	1	100.00

exdb034_11__s2: Who in Child[11]'s Family Helped

	Freq.	%
2 His/her Spouse	1	100.00
Total	1	100.00

exdb034_11__s3: Who in Child[11]'s Family Helped

	Freq.	%
0 No	1	100.00

Total	1	100.00

exdb034_26__s1: Who in Child[26]'s Family Helped

	Freq.	%
0 No	4	20.00
1 Himself/herself	16	80.00
Total	20	100.00

exdb034_26__s2: Who in Child[26]'s Family Helped

	Freq.	%
0 No	4	20.00
2 His/her Spouse	16	80.00
Total	20	100.00

exdb034_26__s3: Who in Child[26]'s Family Helped

	Freq.	%
0 No	15	75.00
3 His/her Children	5	25.00
Total	20	100.00

exdb034_27__s1: Who in Child[27]'s Family Helped

	Freq.	%
1 Himself/herself	6	100.00
Total	6	100.00

exdb034_27__s2: Who in Child[27]'s Family Helped

	Freq.	%
0 No	2	33.33
2 His/her Spouse	4	66.67
Total	6	100.00

exdb034_27__s3: Who in Child[27]'s Family Helped

	Freq.	%
0 No	4	66.67
3 His/her Children	2	33.33
Total	6	100.00

exdb034_28__s1: Who in Child[28]'s Family Helped

	Freq.	%
1 Himself/herself	3	100.00
Total	3	100.00

exdb034_28__s2: Who in Child[28]'s Family Helped

	Freq.	%
0 No	2	66.67
2 His/her Spouse	1	33.33
Total	3	100.00

exdb034_28__s3: Who in Child[28]'s Family Helped

	Freq.	%
0 No	2	66.67
3 His/her Children	1	33.33
Total	3	100.00

exdb034_29__s1: Who in Child[29]'s Family Helped

	Freq.	%
1 Himself/herself	3	100.00
Total	3	100.00

exdb034_29__s2: Who in Child[29]'s Family Helped

	Freq.	%
0 No	2	66.67
2 His/her Spouse	1	33.33
Total	3	100.00

exdb034_29__s3: Who in Child[29]'s Family Helped

	Freq.	%
0 No	2	66.67
3 His/her Children	1	33.33
Total	3	100.00

exdb034_1_1_: Number of Children Helped

Mean	SD	Min	Max	Obs
1.79	1.09	-1.00	7.00	96

exdb034_1_2_: Number of Children Helped

Mean	SD	Min	Max	Obs
1.88	1.10	1.00	8.00	65

exdb034_1_3_: Number of Children Helped

Mean	SD	Min	Max	Obs
1.70	0.98	1.00	5.00	54

exdb034_1_4_: Number of Children Helped

Mean	SD	Min	Max	Obs
1.77	1.09	-1.00	5.00	35

exdb034_1_5_: Number of Children Helped

Mean	SD	Min	Max	Obs
1.93	0.88	1.00	3.00	15

exdb034_1_6_: Number of Children Helped

Mean	SD	Min	Max	Obs
2.38	1.19	1.00	4.00	8

exdb034_1_7_: Number of Children Helped

Mean	SD	Min	Max	Obs
1.67	0.82	1.00	3.00	6

exdb034_1_8_: Number of Children Helped

Mean	SD	Min	Max	Obs
2.00	1.41	1.00	3.00	2

exdb034_1_9_: Number of Children Helped

Mean	SD	Min	Max	Obs
3.00	.	3.00	3.00	1

exdb034_1_26_: Number of Children Helped

Mean	SD	Min	Max	Obs
2.20	0.45	2.00	3.00	5

exdb034_1_27_: Number of Children Helped

Mean	SD	Min	Max	Obs
1.50	0.71	1.00	2.00	2

exdb034_1_28_: Number of Children Helped

Mean	SD	Min	Max	Obs
1.00	.	1.00	1.00	1

exdb034_1_29_: Number of Children Helped

Mean	SD	Min	Max	Obs
2.00	.	2.00	2.00	1

exdb035_s31: Which Sibling's Family Helps

	Freq.	%
31 Other Sibling[31]	18	100.00
Total	18	100.00

exdb035_s32: Which Sibling's Family Helps

	Freq.	%
0 No	14	77.78
32 Other Sibling[32]	4	22.22
Total	18	100.00

exdb035_s33: Which Sibling's Family Helps

	Freq.	%
0 No	15	83.33
33 Other Sibling[33]	3	16.67
Total	18	100.00

exdb035_s34: Which Sibling's Family Helps

	Freq.	%
0 No	16	88.89
34 Other Sibling[34]	2	11.11
Total	18	100.00

exdb035_s35: Which Sibling's Family Helps

	Freq.	%
0 No	16	88.89
35 Other Sibling[35]	2	11.11
Total	18	100.00

exdb035_s36: Which Sibling's Family Helps

	Freq.	%
0 No	17	94.44
36 Other Sibling[36]	1	5.56
Total	18	100.00

exdb035_s37: Which Sibling's Family Helps

	Freq.	%
0 No	17	94.44
37 Other Sibling[37]	1	5.56
Total	18	100.00

exdb035_s38: Which Sibling's Family Helps

	Freq.	%
0 No	17	94.44
38 Other Sibling[38]	1	5.56
Total	18	100.00

exdb036_31__s1: Who Help in Person from Sibling[31]'s Family

	Freq.	%
0 No	3	16.67
1 Himself/herself	15	83.33
Total	18	100.00

exdb036_31__s2: Who Help in Person from Sibling[31]'s Family

	Freq.	%
0 No	12	66.67
2 His/her Spouse	6	33.33
Total	18	100.00

exdb036_31__s3: Who Help in Person from Sibling[31]'s Family

	Freq.	%
0 No	12	66.67
3 His/her Children	6	33.33
Total	18	100.00

exdb036_32__s1: Who Help in Person from Sibling[32]'s Family

	Freq.	%
0 No	1	25.00
1 Himself/herself	3	75.00
Total	4	100.00

exdb036_32__s2: Who Help in Person from Sibling[32]'s Family

	Freq.	%
0 No	1	25.00
2 His/her Spouse	3	75.00
Total	4	100.00

exdb036_32__s3: Who Help in Person from Sibling[32]'s Family

	Freq.	%
0 No	1	25.00
3 His/her Children	3	75.00
Total	4	100.00

exdb036_33__s1: Who Help in Person from Sibling[33]'s Family

	Freq.	%
0 No	1	33.33
1 Himself/herself	2	66.67
Total	3	100.00

exdb036_33__s2: Who Help in Person from Sibling[33]'s Family

	Freq.	%
0 No	2	66.67
2 His/her Spouse	1	33.33
Total	3	100.00

exdb036_33__s3: Who Help in Person from Sibling[33]'s Family

	Freq.	%
3 His/her Children	3	100.00
Total	3	100.00

exdb036_34__s1: Who Help in Person from Sibling[34]'s Family

	Freq.	%
1 Himself/herself	2	100.00
Total	2	100.00

exdb036_34__s2: Who Help in Person from Sibling[34]'s Family

	Freq.	%
0 No	1	50.00
2 His/her Spouse	1	50.00
Total	2	100.00

exdb036_34__s3: Who Help in Person from Sibling[34]'s Family

	Freq.	%
0 No	1	50.00
3 His/her Children	1	50.00
Total	2	100.00

exdb036_35__s1: Who Help in Person from Sibling[35]'s Family

	Freq.	%
1 Himself/herself	2	100.00
Total	2	100.00

exdb036_35__s2: Who Help in Person from Sibling[35]'s Family

	Freq.	%
0 No	1	50.00
2 His/her Spouse	1	50.00
Total	2	100.00

exdb036_35__s3: Who Help in Person from Sibling[35]'s Family

	Freq.	%
3 His/her Children	2	100.00
Total	2	100.00

exdb036_36__s1: Who Help in Person from Sibling[36]'s Family

	Freq.	%
1 Himself/herself	1	100.00
Total	1	100.00

exdb036_36__s2: Who Help in Person from Sibling[36]'s Family

	Freq.	%
2 His/her Spouse	1	100.00
Total	1	100.00

exdb036_36__s3: Who Help in Person from Sibling[36]'s Family

	Freq.	%
3 His/her Children	1	100.00
Total	1	100.00

exdb036_37__s1: Who Help in Person from Sibling[37]'s Family

	Freq.	%
1 Himself/herself	1	100.00
Total	1	100.00

exdb036_37__s2: Who Help in Person from Sibling[37]'s Family

	Freq.	%
2 His/her Spouse	1	100.00
Total	1	100.00

exdb036_37__s3: Who Help in Person from Sibling[37]'s Family

	Freq.	%
3 His/her Children	1	100.00
Total	1	100.00

exdb036_38__s1: Who Help in Person from Sibling[38]'s Family

	Freq.	%
0 No	1	100.00
Total	1	100.00

exdb036_38__s2: Who Help in Person from Sibling[38]'s Family

	Freq.	%
2 His/her Spouse	1	100.00
Total	1	100.00

exdb036_38__s3: Who Help in Person from Sibling[38]'s Family

	Freq.	%
3 His/her Children	1	100.00
Total	1	100.00

exdb036_1_31_: Number of Children Helped

Mean	SD	Min	Max	Obs
3.00	2.00	1.00	6.00	6

exdb036_1_32_: Number of Children Helped

Mean	SD	Min	Max	Obs
1.67	0.58	1.00	2.00	3

exdb036_1_33_: Number of Children Helped

Mean	SD	Min	Max	Obs
2.00	1.00	1.00	3.00	3

exdb036_1_34_: Number of Children Helped

Mean	SD	Min	Max	Obs
2.00	.	2.00	2.00	1

exdb036_1_35_: Number of Children Helped

Mean	SD	Min	Max	Obs
1.50	0.71	1.00	2.00	2

exdb036_1_36_: Number of Children Helped

Mean	SD	Min	Max	Obs
2.00	.	2.00	2.00	1

exdb036_1_37_: Number of Children Helped

Mean	SD	Min	Max	Obs
2.00	.	2.00	2.00	1

exdb036_1_38_: Number of Children Helped

Mean	SD	Min	Max	Obs
1.00	.	1.00	1.00	1

exdb037: Number of Relatives Help in Person

Mean	SD	Min	Max	Obs
1.25	0.50	1.00	2.00	4

exdb038: Number of Others Who Help in Person

Mean	SD	Min	Max	Obs
2.83	1.17	2.00	5.00	6

exdb039_s1: Choose Most Important 7 Helpers

	Freq.	%
0 No	4	5.88
1 Helper[1]	64	94.12
Total	68	100.00

exdb039_s2: Choose Most Important 7 Helpers

	Freq.	%
0 No	13	19.12
2 Helper[2]	55	80.88
Total	68	100.00

exdb039_s3: Choose Most Important 7 Helpers

	Freq.	%

	Freq.	%
0 No	30	44.12
3 Helper[3]	38	55.88
Total	68	100.00

exdb039_s4: Choose Most Important 7 Helpers

	Freq.	%
0 No	16	23.53
4 Helper[4]	52	76.47
Total	68	100.00

exdb039_s5: Choose Most Important 7 Helpers

	Freq.	%
0 No	18	26.47
5 Helper[5]	50	73.53
Total	68	100.00

exdb039_s6: Choose Most Important 7 Helpers

	Freq.	%
0 No	27	39.71
6 Helper[6]	41	60.29
Total	68	100.00

exdb039_s7: Choose Most Important 7 Helpers

	Freq.	%
0 No	23	33.82
7 Helper[7]	45	66.18
Total	68	100.00

exdb039_s8: Choose Most Important 7 Helpers

	Freq.	%
0 No	22	32.35
8 Helper[8]	46	67.65
Total	68	100.00

exdb039_s9: Choose Most Important 7 Helpers

	Freq.	%
0 No	45	66.18
9 Helper[9]	23	33.82
Total	68	100.00

exdb039_s10: Choose Most Important 7 Helpers

	Freq.	%

	Freq.	%
0 No	47	69.12
10 Helper[10]	21	30.88
Total	68	100.00

exdb039_s11: Choose Most Important 7 Helpers

	Freq.	%
0 No	50	73.53
11 Helper[11]	18	26.47
Total	68	100.00

exdb039_s12: Choose Most Important 7 Helpers

	Freq.	%
0 No	64	94.12
12 Helper[12]	4	5.88
Total	68	100.00

exdb039_s13: Choose Most Important 7 Helpers

	Freq.	%
0 No	63	92.65
13 Helper[13]	5	7.35
Total	68	100.00

exdb039_s14: Choose Most Important 7 Helpers

	Freq.	%
0 No	63	92.65
14 Helper[14]	5	7.35
Total	68	100.00

exdb039_s15: Choose Most Important 7 Helpers

	Freq.	%
0 No	65	95.59
15 Helper[15]	3	4.41
Total	68	100.00

exdb039_s16: Choose Most Important 7 Helpers

	Freq.	%
0 No	64	94.12
16 Helper[16]	4	5.88
Total	68	100.00

exdb039_s17: Choose Most Important 7 Helpers

	Freq.	%

	Freq.	%
0 No	68	100.00
Total	68	100.00

exdb039_s18: Choose Most Important 7 Helpers

	Freq.	%
0 No	67	98.53
18 Helper[18]	1	1.47
Total	68	100.00

exdb039_s19: Choose Most Important 7 Helpers

	Freq.	%
0 No	67	98.53
19 Helper[19]	1	1.47
Total	68	100.00

exdb040_1_: Days Helper[1] Helped

Mean	SD	Min	Max	Obs
25.48	9.55	-1.00	31.00	533

exdb040_2_: Days Helper[2] Helped

Mean	SD	Min	Max	Obs
21.13	11.73	-1.00	31.00	305

exdb040_3_: Days Helper[3] Helped

Mean	SD	Min	Max	Obs
16.90	12.05	-1.00	31.00	216

exdb040_4_: Days Helper[4] Helped

Mean	SD	Min	Max	Obs
14.88	12.19	-1.00	30.00	163

exdb040_5_: Days Helper[5] Helped

Mean	SD	Min	Max	Obs
14.54	11.89	-1.00	31.00	118

exdb040_6_: Days Helper[6] Helped

Mean	SD	Min	Max	Obs
15.95	12.18	-1.00	31.00	82

exdb040_7_: Days Helper[7] Helped

Mean	SD	Min	Max	Obs
16.45	12.23	-1.00	30.00	65

exdb040_8_: Days Helper[8] Helped

Mean	SD	Min	Max	Obs
17.85	12.33	-1.00	30.00	46

exdb040_9_: Days Helper[9] Helped

Mean	SD	Min	Max	Obs
17.48	11.90	-1.00	30.00	23

exdb040_10_: Days Helper[10] Helped

Mean	SD	Min	Max	Obs
16.33	12.68	-1.00	30.00	21

exdb040_11_: Days Helper[11] Helped

Mean	SD	Min	Max	Obs
18.17	13.70	0.00	30.00	18

exdb040_12_: Days Helper[12] Helped

Mean	SD	Min	Max	Obs
14.50	11.45	3.00	30.00	4

exdb040_13_: Days Helper[13] Helped

Mean	SD	Min	Max	Obs
11.40	10.95	2.00	30.00	5

exdb040_14_: Days Helper[14] Helped

Mean	SD	Min	Max	Obs
10.60	11.22	2.00	30.00	5

exdb040_15_: Days Helper[15] Helped

Mean	SD	Min	Max	Obs
12.00	15.62	2.00	30.00	3

exdb040_16_: Days Helper[16] Helped

Mean	SD	Min	Max	Obs
5.75	3.30	2.00	10.00	4

exdb040_18_: Days Helper[18] Helped

Mean	SD	Min	Max	Obs
15.00	.	15.00	15.00	1

exdb040_19_: Days Helper[19] Helped

Mean	SD	Min	Max	Obs
5.00	.	5.00	5.00	1

exdb041_1_: Hours Helper[1] Helped

Mean	SD	Min	Max	Obs
10.55	9.33	-1.00	24.00	533

exdb041_2_: Hours Helper[2] Helped

Mean	SD	Min	Max	Obs
8.29	8.70	-1.00	24.00	305

exdb041_3_: Hours Helper[3] Helped

Mean	SD	Min	Max	Obs
6.56	7.63	-1.00	24.00	216

exdb041_4_: Hours Helper[4] Helped

Mean	SD	Min	Max	Obs
7.18	8.32	-1.00	24.00	163

exdb041_5_: Hours Helper[5] Helped

Mean	SD	Min	Max	Obs
7.33	8.17	-1.00	24.00	118

exdb041_6_: Hours Helper[6] Helped

Mean	SD	Min	Max	Obs
7.68	8.29	-1.00	24.00	82

exdb041_7_: Hours Helper[7] Helped

Mean	SD	Min	Max	Obs
9.31	8.49	-1.00	24.00	65

exdb041_8_: Hours Helper[8] Helped

Mean	SD	Min	Max	Obs
6.89	6.90	-1.00	24.00	46

exdb041_9_: Hours Helper[9] Helped

Mean	SD	Min	Max	Obs
9.48	8.38	-1.00	24.00	23

exdb041_10_: Hours Helper[10] Helped

Mean	SD	Min	Max	Obs
7.67	7.88	-1.00	24.00	21

exdb041_11_: Hours Helper[11] Helped

Mean	SD	Min	Max	Obs
8.89	8.48	-1.00	24.00	18

exdb041_12_: Hours Helper[12] Helped

Mean	SD	Min	Max	Obs
7.75	10.90	1.00	24.00	4

exdb041_13_: Hours Helper[13] Helped

Mean	SD	Min	Max	Obs
7.60	9.32	2.00	24.00	5

exdb041_14_: Hours Helper[14] Helped

Mean	SD	Min	Max	Obs
3.20	2.77	1.00	8.00	5

exdb041_15_: Hours Helper[15] Helped

Mean	SD	Min	Max	Obs
1.00	1.73	-1.00	2.00	3

exdb041_16_: Hours Helper[16] Helped

Mean	SD	Min	Max	Obs
5.00	4.76	2.00	12.00	4

exdb041_18_: Hours Helper[18] Helped

Mean	SD	Min	Max	Obs
12.00	.	12.00	12.00	1

exdb041_19_: Hours Helper[19] Helped

Mean	SD	Min	Max	Obs
4.00	.	4.00	4.00	1

exdb042_1_: Helper[1] Living in Your Home

	Freq.	%
1 Yes	466	87.43
2 No	67	12.57
Total	533	100.00

exdb042_2_: Helper[2] Living in Your Home

	Freq.	%
1 Yes	194	63.61
2 No	111	36.39
Total	305	100.00

exdb042_3_: Helper[3] Living in Your Home

	Freq.	%
1 Yes	121	56.02
2 No	95	43.98
Total	216	100.00

exdb042_4_: Helper[4] Living in Your Home

	Freq.	%
1 Yes	78	47.85
2 No	85	52.15
Total	163	100.00

exdb042_5_: Helper[5] Living in Your Home

	Freq.	%
1 Yes	53	44.92
2 No	65	55.08
Total	118	100.00

exdb042_6_: Helper[6] Living in Your Home

	Freq.	%
1 Yes	42	51.22
2 No	40	48.78
Total	82	100.00

exdb042_7_: Helper[7] Living in Your Home

	Freq.	%
1 Yes	40	61.54
2 No	25	38.46
Total	65	100.00

exdb042_8_: Helper[8] Living in Your Home

	Freq.	%
1 Yes	26	56.52
2 No	20	43.48
Total	46	100.00

exdb042_9_: Helper[9] Living in Your Home

	Freq.	%
1 Yes	13	56.52
2 No	10	43.48
Total	23	100.00

exdb042_10_: Helper[10] Living in Your Home

	Freq.	%
1 Yes	12	57.14
2 No	9	42.86
Total	21	100.00

exdb042_11_: Helper[11] Living in Your Home

	Freq.	%
1 Yes	10	55.56
2 No	8	44.44
Total	18	100.00

exdb042_12_: Helper[12] Living in Your Home

	Freq.	%
1 Yes	2	50.00
2 No	2	50.00
Total	4	100.00

exdb042_13_: Helper[13] Living in Your Home

	Freq.	%
1 Yes	3	60.00
2 No	2	40.00
Total	5	100.00

exdb042_14_: Helper[14] Living in Your Home

	Freq.	%
1 Yes	2	40.00
2 No	3	60.00
Total	5	100.00

exdb042_15_: Helper[15] Living in Your Home

	Freq.	%
1 Yes	2	66.67
2 No	1	33.33
Total	3	100.00

exdb042_16_: Helper[16] Living in Your Home

	Freq.	%
1 Yes	2	50.00
2 No	2	50.00
Total	4	100.00

exdb042_18_: Helper[18] Living in Your Home

	Freq.	%
2 No	1	100.00
Total	1	100.00

exdb042_19_: Helper[19] Living in Your Home

	Freq.	%
2 No	1	100.00
Total	1	100.00

exdb043_s1: Aids Used

	Freq.	%
0 No	301	56.05
1 Walking Stick	236	43.95
Total	537	100.00

exdb043_s2: Aids Used

	Freq.	%
0 No	503	93.67
2 Walker	34	6.33
Total	537	100.00

exdb043_s3: Aids Used

	Freq.	%
0 No	396	73.74
3 Manual Wheelchair	141	26.26
Total	537	100.00

exdb043_s4: Aids Used

	Freq.	%
0 No	526	97.95
4 Electric Wheelchair	11	2.05
Total	537	100.00

exdb043_s5: Aids Used

	Freq.	%
0 No	424	78.96
5 Catheter	113	21.04
Total	537	100.00

exdb043_s6: Aids Used

	Freq.	%
0 No	331	61.64
6 Toilet Series	206	38.36
Total	537	100.00

exdb043_s7: Aids Used

	Freq.	%
0 No	388	72.25
7 None of the Above	149	27.75
Total	537	100.00

exea001_s1: Urban Employee Medical Insurance (yi-bao)

Mean	SD	Min	Max	Obs
0.14	0.35	0.00	1.00	762

exea001_s2: Urban and Rural Resident Medical Insurance

Mean	SD	Min	Max	Obs
0.18	0.57	0.00	2.00	762

exea001_s3: Urban Resident Medical Insurance

Mean	SD	Min	Max	Obs
0.11	0.55	0.00	3.00	762

exea001_s4: New Cooperative Medical Insurance (he-zuo-yi-liao)

Mean	SD	Min	Max	Obs
2.65	1.89	0.00	4.00	762

exea001_s5: Government Medical Insurance (gong-fei-yi-liao)

Mean	SD	Min	Max	Obs
0.10	0.72	0.00	5.00	762

exea001_s6: Medical Aid

Mean	SD	Min	Max	Obs
0.09	0.75	0.00	6.00	762

exea001_s7: Private Medical Insurance: Purchased by Work Unit

Mean	SD	Min	Max	Obs
0.02	0.36	0.00	7.00	762

exea001_s8: Private Medical Insurance: Purchased by Individual

Mean	SD	Min	Max	Obs
0.17	1.15	0.00	8.00	762

exea001_s9: Urban Non-employed Persons's Health Insurance

Mean	SD	Min	Max	Obs
0.04	0.56	0.00	9.00	762

exea001_s10: Long-term Care Insurance

Mean	SD	Min	Max	Obs
0.00	0.00	0.00	0.00	762

exea001_s11: Other Medical Insurance (Specify)

Mean	SD	Min	Max	Obs
0.23	1.58	0.00	11.00	762

exea001_s12: No Insurance

Mean	SD	Min	Max	Obs
0.63	2.68	0.00	12.00	762

exea001_verify: Verify Health Insurance

Mean	SD	Min	Max	Obs
1.00	0.00	1.00	1.00	39

exea002: Supplemental Insurance

	Freq.	%
1 Yes	63	8.76
2 No	656	91.24
Total	719	100.00

exea003_4_: Where Did R Set Up XEXMINS_4_

	Freq.	%
1 Residence Before Death	483	95.64
2 (If It Is Not In This County)The Place of R's HuKou	18	3.56
3 Other	4	0.79
Total	505	100.00

exea003_2_: Where Did R Set Up XEXMINS_2_

	Freq.	%
1 Residence Before Death	66	95.65
2 (If It Is Not In This County)The Place of R's HuKou	3	4.35
Total	69	100.00

exea003_3_: Where Did R Set Up XEXMINS_3_

	Freq.	%
1 Residence Before Death	24	88.89
2 (If It Is Not In This County)The Place of R's HuKou	3	11.11
Total	27	100.00

exea003_1_: Where Did R Set Up XEXMINS_1_

	Freq.	%
1 Residence Before Death	83	76.85
2 (If It Is Not In This County)The Place of R's HuKou	10	9.26

	Freq.	%
3 Other	15	13.89
Total	108	100.00

exea003_6_: Where Did R Set Up XEXMINS_6_

	Freq.	%
1 Residence Before Death	12	100.00
Total	12	100.00

exea003_5_: Where Did R Set Up XEXMINS_5_

	Freq.	%
1 Residence Before Death	12	75.00
2 (If It Is Not In This County)The Place of R's HuKou	1	6.25
3 Other	3	18.75
Total	16	100.00

exea003_8_: Where Did R Set Up XEXMINS_8_

	Freq.	%
1 Residence Before Death	13	81.25
2 (If It Is Not In This County)The Place of R's HuKou	2	12.50
3 Other	1	6.25
Total	16	100.00

exea003_11_: Where Did R Set Up XEXMINS_11_

	Freq.	%
1 Residence Before Death	15	93.75
2 (If It Is Not In This County)The Place of R's HuKou	1	6.25
Total	16	100.00

exea003_9_: Where Did R Set Up XEXMINS_9_

	Freq.	%
1 Residence Before Death	3	100.00
Total	3	100.00

exea003_7_: Where Did R Set Up XEXMINS_7_

	Freq.	%
2 (If It Is Not In This County)The Place of R's HuKou	1	50.00
3 Other	1	50.00
Total	2	100.00

exea008_1_4_: Year of XEXMINS_4_ Begin

Mean	SD	Min	Max	Obs
2,006.45	8.24	1,937.00	2,020.00	505

exea008_1_2_: Year of XEXMINS_2_ Begin

Mean	SD	Min	Max	Obs
2,007.91	7.63	1,975.00	2,019.00	69

exea008_1_3_: Year of XEXMINS_3_ Begin

Mean	SD	Min	Max	Obs
2,006.33	9.70	1,977.00	2,018.00	27

exea008_1_1_: Year of XEXMINS_1_ Begin

Mean	SD	Min	Max	Obs
1,990.28	19.04	1,930.00	2,020.00	108

exea008_1_6_: Year of XEXMINS_6_ Begin

Mean	SD	Min	Max	Obs
2,014.25	6.65	1,994.00	2,019.00	12

exea008_1_5_: Year of XEXMINS_5_ Begin

Mean	SD	Min	Max	Obs
1,973.56	21.65	1,947.00	2,014.00	16

exea008_1_8_: Year of XEXMINS_8_ Begin

Mean	SD	Min	Max	Obs
2,013.63	5.97	2,000.00	2,020.00	16

exea008_1_11_: Year of XEXMINS_11_ Begin

Mean	SD	Min	Max	Obs
2,014.38	4.32	2,001.00	2,019.00	16

exea008_1_9_: Year of XEXMINS_9_ Begin

Mean	SD	Min	Max	Obs
2,013.33	3.06	2,010.00	2,016.00	3

exea008_1_7_: Year of XEXMINS_7_ Begin

Mean	SD	Min	Max	Obs
1,995.50	28.99	1,975.00	2,016.00	2

exea008_2_4_: Month of XEXMINS_4_ Begin

Mean	SD	Min	Max	Obs
6.10	3.94	1.00	12.00	137

exea008_2_2_: Month of XEXMINS_2_ Begin

Mean	SD	Min	Max	Obs
7.64	3.67	1.00	12.00	22

exea008_2_3_: Month of XEXMINS_3_ Begin

Mean	SD	Min	Max	Obs
7.30	4.03	1.00	12.00	10

exea008_2_1_: Month of XEXMINS_1_ Begin

Mean	SD	Min	Max	Obs
6.20	4.03	1.00	12.00	25

exea008_2_6_: Month of XEXMINS_6_ Begin

Mean	SD	Min	Max	Obs
8.25	3.10	4.00	11.00	4

exea008_2_5_: Month of XEXMINS_5_ Begin

Mean	SD	Min	Max	Obs
6.00	1.83	4.00	8.00	4

exea008_2_8_: Month of XEXMINS_8_ Begin

Mean	SD	Min	Max	Obs
5.00	4.06	1.00	10.00	5

exea008_2_11_: Month of XEXMINS_11_ Begin

Mean	SD	Min	Max	Obs
5.50	3.46	1.00	10.00	8

exea008_2_9_: Month of XEXMINS_9_ Begin

Mean	SD	Min	Max	Obs
9.50	0.71	9.00	10.00	2

exea008_2_7_: Month of XEXMINS_7_ Begin

No Observations

exea009_s1: I Do Not Need It

Mean	SD	Min	Max	Obs
0.05	0.22	0.00	1.00	39

exea009_s2: Cannot Afford It

Mean	SD	Min	Max	Obs
0.36	0.78	0.00	2.00	39

exea009_s3: Do Not Know Where or from Whom to Get It

Mean	SD	Min	Max	Obs
0.31	0.92	0.00	3.00	39

exea009_s5: Do Not Have Suitable Programs for Me to Buy

Mean	SD	Min	Max	Obs
0.13	0.80	0.00	5.00	39

exea009_s6: Never Thought of It

Mean	SD	Min	Max	Obs
0.92	2.19	0.00	6.00	39

exea009_s7: Others

Mean	SD	Min	Max	Obs
4.31	3.45	0.00	7.00	39

exeb001_s1: Urban Employee Medical Insurance (yi-bao)

Mean	SD	Min	Max	Obs
0.00	0.00	0.00	0.00	39

exeb001_s2: Urban and Rural Resident Medical Insurance

Mean	SD	Min	Max	Obs
0.00	0.00	0.00	0.00	39

exeb001_s3: Urban Resident Medical Insurance

Mean	SD	Min	Max	Obs
0.00	0.00	0.00	0.00	39

exeb001_s4: New Cooperative Medical Insurance (he-zuo-yi-liao)

Mean	SD	Min	Max	Obs
0.31	1.08	0.00	4.00	39

exeb001_s5: Government Medical Insurance (gong-fei)

Mean	SD	Min	Max	Obs
0.00	0.00	0.00	0.00	39

exeb001_s6: Medical Aid

Mean	SD	Min	Max	Obs
0.00	0.00	0.00	0.00	39

exeb001_s7: Private Medical Insurance: Purchased by Work Unit

Mean	SD	Min	Max	Obs
0.00	0.00	0.00	0.00	39

exeb001_s8: Private Medical Insurance: Purchased by Individual

Mean	SD	Min	Max	Obs
0.00	0.00	0.00	0.00	39

exeb001_s9: Urban Non-employed Persons's Health Insurance

Mean	SD	Min	Max	Obs
0.00	0.00	0.00	0.00	39

exeb001_s10: Long-term Care Insurance

Mean	SD	Min	Max	Obs
0.00	0.00	0.00	0.00	39

exeb001_s11: Other Medical Insurance (Specify)

Mean	SD	Min	Max	Obs
0.00	0.00	0.00	0.00	39

exeb001_s12: No Insurance

Mean	SD	Min	Max	Obs
11.08	3.24	0.00	12.00	39

exeb003_1_4_: Year of Stoping Participate in New Cooperative Medical Insurance

Mean	SD	Min	Max	Obs
2,018.67	0.58	2,018.00	2,019.00	3

exeb003_2_4_: Month of Stopping Participate in New Cooperative Medical Insurance

Mean	SD	Min	Max	Obs
9.33	2.31	8.00	12.00	3

exeb004_4_: Reason of Stopping Participate in New Cooperative Medical Insurance

Mean	SD	Min	Max	Obs
7.00	0.00	7.00	7.00	3

exeb005: Receive Money from Any Health Insurance

Mean	SD	Min	Max	Obs
1.88	0.33	1.00	2.00	722

exeb006: How Much Is this Money

Mean	SD	Min	Max	Obs
14,516.44	52,142.33	0.00	400,000.00	72

exec001: Date of Last Physical Examination

	Freq.	%
1 Time of Physical Examination	270	35.53
2 Didn't Take Physical Examination Since Last Interview	490	64.47
Total	760	100.00

exec001_1: Year

Mean	SD	Min	Max	Obs
2,018.23	1.90	2,000.00	2,020.00	270

exec001_2: Month

Mean	SD	Min	Max	Obs

5.87	2.89	1.00	12.00	181

exed030: Times of Respondent Visited A Public Hospital, Private Hospital, or Been Visited

Mean	SD	Min	Max	Obs
1.62	4.48	0.00	60.00	760

exed031: Total Cost

Mean	SD	Min	Max	Obs
9,777.10	34,266.11	0.00	400,000.00	221

exed031_min: EXED031_min

A String Variable	
Obs:	43

exed031_max: EXED031_max

A String Variable	
Obs:	21

exed032: Cost of Self-paid

	Freq.	%
1 Cost of Self-paid	205	74.01
2 Didn't Pay Anything	72	25.99
Total	277	100.00

exed032_1: EXED032_1

Mean	SD	Min	Max	Obs
7,546.54	32,764.23	1.00	400,000.00	163

exed032_1_min: EXED032_1_min

A String Variable	
Obs:	26

exed032_1_max: EXED032_1_max

A String Variable	
Obs:	10

exed033_s1: XEXMIns[1]

Mean	SD	Min	Max	Obs
0.09	0.29	0.00	1.00	277

exed033_s2: XEXMIns[2]

Mean	SD	Min	Max	Obs
0.15	0.53	0.00	2.00	277

exed033_s3: XEXMIns[3]

Mean	SD	Min	Max	Obs
0.10	0.53	0.00	3.00	277

exed033_s4: XEXMIns[4]

Mean	SD	Min	Max	Obs
1.92	2.00	0.00	4.00	277

exed033_s5: XEXMIns[5]

Mean	SD	Min	Max	Obs
0.13	0.79	0.00	5.00	277

exed033_s6: XEXMIns[6]

Mean	SD	Min	Max	Obs
0.04	0.51	0.00	6.00	277

exed033_s7: XEXMIns[7]

Mean	SD	Min	Max	Obs
0.05	0.59	0.00	7.00	277

exed033_s8: XEXMIns[8]

Mean	SD	Min	Max	Obs
0.03	0.48	0.00	8.00	277

exed033_s9: XEXMIns[9]

Mean	SD	Min	Max	Obs
0.03	0.54	0.00	9.00	277

exed033_s10: XEXMIns[10]

Mean	SD	Min	Max	Obs
0.00	0.00	0.00	0.00	277

exed033_s11: XEXMIns[11]

Mean	SD	Min	Max	Obs
0.12	1.14	0.00	11.00	277

exed033_s12: Reimbursed by R's union

Mean	SD	Min	Max	Obs
0.00	0.00	0.00	0.00	277

exed033_s13: No Insurance

Mean	SD	Min	Max	Obs
0.66	2.85	0.00	13.00	277

exed033_s14: Not Revelent to R

Mean	SD	Min	Max	Obs
3.44	6.04	0.00	14.00	277

exef001: Take Any Purchased Medicine in the Month before Death

	Freq.	%
1 Yes	406	53.42
2 No	354	46.58
Total	760	100.00

exef002: Total Cost for Purchased Medicine

Mean	SD	Min	Max	Obs
3,210.92	10,639.09	0.00	120,000.00	297

exef002_min: EXEF002_min

A String Variable	
Obs:	75

exef002_max: EXEF002_max

A String Variable	
Obs:	39

exef003: Self-Paid

Mean	SD	Min	Max	Obs
1.15	0.36	1.00	2.00	406

exef003_1: EXEF003_1

Mean	SD	Min	Max	Obs
2,760.22	8,817.78	1.00	75,000.00	276

exef003_1_min: EXEF003_1_min

A String Variable	
Obs:	37

exef003_1_max: EXEF003_1_max

A String Variable	
Obs:	16

exef005_s1: XEXMIns[1]

Mean	SD	Min	Max	Obs
0.08	0.27	0.00	1.00	406

exef005_s2: XEXMIns[2]

Mean	SD	Min	Max	Obs
0.09	0.41	0.00	2.00	406

exef005_s3: XEXMIns[3]

Mean	SD	Min	Max	Obs
0.05	0.39	0.00	3.00	406

exef005_s4: XEXMIns[4]

Mean	SD	Min	Max	Obs
1.27	1.86	0.00	4.00	406

exef005_s5: XEXMIns[5]

Mean	SD	Min	Max	Obs
0.04	0.43	0.00	5.00	406

exef005_s6: XEXMIns[6]

Mean	SD	Min	Max	Obs
0.01	0.30	0.00	6.00	406

exef005_s7: XEXMIns[7]

Mean	SD	Min	Max	Obs
0.03	0.49	0.00	7.00	406

exef005_s8: XEXMIns[8]

Mean	SD	Min	Max	Obs
0.02	0.40	0.00	8.00	406

exef005_s9: XEXMIns[9]

Mean	SD	Min	Max	Obs
0.04	0.63	0.00	9.00	406

exef005_s10: XEXMIns[10]

Mean	SD	Min	Max	Obs
0.00	0.00	0.00	0.00	406

exef005_s11: XEXMIns[11]

Mean	SD	Min	Max	Obs
0.03	0.55	0.00	11.00	406

exef005_s12: Reimbursed by R's union

Mean	SD	Min	Max	Obs
0.03	0.60	0.00	12.00	406

exef005_s13: No Insurance

Mean	SD	Min	Max	Obs
1.57	4.24	0.00	13.00	406

exef005_s14: Not Revelent to R

Mean	SD	Min	Max	Obs
5.83	6.91	0.00	14.00	406

exeg000: Dead in Hospital

Mean	SD	Min	Max	Obs
1.82	0.39	1.00	2.00	760

exeg001: How Long Been A Patient in that Hospital before Death

Mean	SD	Min	Max	Obs
2.61	1.12	1.00	4.00	138

exeg001_1: EXEG001_1

Mean	SD	Min	Max	Obs
4.48	5.51	0.00	20.00	28

exeg001_2: EXEG001_2

Mean	SD	Min	Max	Obs
2.72	1.78	1.00	6.00	32

exeg001_3: EXEG001_3

Mean	SD	Min	Max	Obs
1.94	0.98	1.00	4.00	34

exeg001_4: EXEG001_4

Mean	SD	Min	Max	Obs
2.65	2.20	1.00	9.50	40

exeg001_5: EXEG001_5

No Observations

exeg002: Why Had He/She Been Admitted to the Hospital?

	Freq.	%
1 Surgery	19	13.77
2 Receive Other Treatments	20	14.49
3 Relieve Symptoms	51	36.96
4 Others	48	34.78
Total	138	100.00

exeg003: In Addition to that Hospital Stay, in the One Month before His/Her Death Had He/

Mean	SD	Min	Max	Obs
1.72	0.45	1.00	2.00	138

exeg004: In the One Year before His/Her Death Had He/She Been A Patient in A Hospital Ove

Mean	SD	Min	Max	Obs
1.43	0.50	1.00	2.00	760

exeg005: How Many Different Times Was He/She A Patient in A Hospital Overnight in the One

Mean	SD	Min	Max	Obs
3.63	5.21	1.00	50.00	430

exeg006: During Any of Those Hospital Stays did R Spend any Time in An Intensive Care Uni

	Freq.	%
1 Yes	73	16.98
2 No	357	83.02
Total	430	100.00

exeg006_1: EXEG006_1

Mean	SD	Min	Max	Obs
16.39	28.12	1.00	180.00	69

exeg007_s1: Respirator

Mean	SD	Min	Max	Obs
0.41	0.49	0.00	1.00	430

exeg007_s2: Artificial liver

Mean	SD	Min	Max	Obs
0.04	0.29	0.00	2.00	430

exeg007_s3: Artificial lung

Mean	SD	Min	Max	Obs
0.10	0.53	0.00	3.00	430

exeg007_s4: None of above

Mean	SD	Min	Max	Obs
2.34	1.97	0.00	4.00	430

exeg008: During Any of Those Hospital Stays Did R Use Kidney Dialysis Services?

Mean	SD	Min	Max	Obs
1.92	0.27	1.00	2.00	430

exeg009: During Any of Those Hospital Stays did R Receive Antibiotics to Treat Pneumonia

Mean	SD	Min	Max	Obs
1.87	0.33	1.00	2.00	430

exeg010: Total Cost for Hospital Bills

Mean	SD	Min	Max	Obs
46,798.83	78,083.82	0.00	750,000.00	356

exeg011: Self-paid Part

Mean	SD	Min	Max	Obs
1.28	0.45	1.00	2.00	430

exeg011_1: Self-paid Part

Mean	SD	Min	Max	Obs
29,286.06	50,887.97	48.00	570,000.00	248

exeg012_s1: XEXMIns[1]

Mean	SD	Min	Max	Obs
0.16	0.36	0.00	1.00	430

exeg012_s2: XEXMIns[2]

Mean	SD	Min	Max	Obs
0.20	0.59	0.00	2.00	430

exeg012_s3: XEXMIns[3]

Mean	SD	Min	Max	Obs
0.07	0.45	0.00	3.00	430

exeg012_s4: XEXMIns[4]

Mean	SD	Min	Max	Obs
2.51	1.94	0.00	4.00	430

exeg012_s5: XEXMIns[5]

Mean	SD	Min	Max	Obs
0.10	0.72	0.00	5.00	430

exeg012_s6: XEXMIns[6]

Mean	SD	Min	Max	Obs
0.10	0.76	0.00	6.00	430

exeg012_s7: XEXMIns[7]

Mean	SD	Min	Max	Obs
0.05	0.58	0.00	7.00	430

exeg012_s8: XEXMIns[8]

Mean	SD	Min	Max	Obs
0.06	0.67	0.00	8.00	430

exeg012_s9: XEXMIns[9]

Mean	SD	Min	Max	Obs
0.10	0.97	0.00	9.00	430

exeg012_s10: XEXMIns[10]

Mean	SD	Min	Max	Obs
0.00	0.00	0.00	0.00	430

exeg012_s11: XEXMIns[11]

Mean	SD	Min	Max	Obs
0.15	1.29	0.00	11.00	430

exeg012_s12: Reimbursed by R's Union

Mean	SD	Min	Max	Obs
0.11	1.15	0.00	12.00	430

exeg012_s13: No Insurance

Mean	SD	Min	Max	Obs
0.36	2.14	0.00	13.00	430

exeg012_s14: Not Revelent to R

Mean	SD	Min	Max	Obs
0.46	2.49	0.00	14.00	430

exf001: Time of Quitting Job

	Freq.	%
1 Year and Month	86	47.25
2 Worked Till Death	51	28.02
995 Denial of Working LastIW	34	18.68
997 Do not Know	10	5.49
999 Refuse to Answer	1	0.55
Total	182	100.00

exf001_1: Year of Quitting Job

Mean	SD	Min	Max	Obs
1,948.63	370.58	-1.00	2,020.00	87

exf001_2: Month of Quitting Job

Mean	SD	Min	Max	Obs
6.36	4.10	-1.00	12.00	87

exf002: Days Worked During the Last Working Week

Mean	SD	Min	Max	Obs
4.59	3.16	-1.00	7.00	137

exf003_1: Hours Worked Per Day During the Last Working Week

Mean	SD	Min	Max	Obs
5.99	3.81	1.00	24.00	137

exf003_2: Hours Worked in Farming

Mean	SD	Min	Max	Obs
3.22	3.27	-1.00	12.00	137

exf003_3: Hours Worked in Non-farming Activities

Mean	SD	Min	Max	Obs
2.51	4.50	-1.00	24.00	137

exf004: Retirement Processed

	Freq.	%
1 Yes	40	6.30
2 No	576	90.71
997 Do not Know	19	2.99
Total	635	100.00

exf005: Inherited From Pensions

Mean	SD	Min	Max	Obs
2,875.76	9,976.69	0.00	84,000.00	552

exf006: Receive A Lump-Sum Compensation From Pensions

	Freq.	%
1 Yes	193	25.39
2 No	567	74.61
Total	760	100.00

exf007: How Mucn Was It

Mean	SD	Min	Max	Obs
23,257.92	45,305.97	80.00	400,000.00	157

exf008: Receive A Lump-sum Compensation From Other Places?

	Freq.	%
1 Yes	87	11.45
2 No	673	88.55
Total	760	100.00

exf009: How Mucn Was It

Mean	SD	Min	Max	Obs
9,611.04	25,147.96	300.00	185,000.00	77

exf010: Was R Insured By Life Insurance?

	Freq.	%
1 Yes	28	3.68
2 No	732	96.32
Total	760	100.00

exf011: How Much Did the Life Insurance Pay Upon the Death of R

Mean	SD	Min	Max	Obs
24,650.00	35,171.42	1,000.00	150,000.00	18

exf012: Did R Have Commercial Pension?

	Freq.	%
1 Yes	6	0.79
2 No	754	99.21
Total	760	100.00

exf013: How Much Have Been Inherited From Commercial Pension?

Mean	SD	Min	Max	Obs
23,875.00	17,890.29	10,000.00	50,000.00	4

exg001: Own House

	Freq.	%
1 Yes	331	43.55
2 No	429	56.45
Total	760	100.00

exg001_1: Total Value of House Properties

Mean	SD	Min	Max	Obs
109,691.54	503,275.20	-1.00	8,000,000.00	331

exg002_s1: House Disposed: Spouse

	Freq.	%
0 No	263	79.46
1 Yes	68	20.54
Total	331	100.00

exg002_s2: House Disposed: Children/Sons-in-law/Daughters-in-law

	Freq.	%
0 No	189	57.10
2 Yes	142	42.90
Total	331	100.00

exg002_s3: House Disposed: Sibling

	Freq.	%
0 No	326	98.49
3 Yes	5	1.51
Total	331	100.00

exg002_s4: House Disposed: Other Relatives

	Freq.	%

	Freq.	%
0 No	328	99.09
4 Yes	3	0.91
Total	331	100.00

exg002_s5: House Disposed: Parents/Parents-in-laws

	Freq.	%
0 No	331	100.00
Total	331	100.00

exg002_s6: House Disposed: Grandchildren

	Freq.	%
0 No	325	98.19
6 Yes	6	1.81
Total	331	100.00

exg002_s7: House Disposed: Friends

	Freq.	%
0 No	331	100.00
Total	331	100.00

exg002_s8: House Disposed: Charity

	Freq.	%
0 No	331	100.00
Total	331	100.00

exg002_s9: House Disposed: Others

	Freq.	%
0 No	325	98.19
9 Yes	6	1.81
Total	331	100.00

exg002_s10: House Disposed: No Allocation

	Freq.	%
0 No	221	66.77
10 Yes	110	33.23
Total	331	100.00

exg002_1: House Disposed: Spouse(%)

Mean	SD	Min	Max	Obs
88.91	25.41	-1.00	100.00	68

exg002_3: House Disposed: Sibling(%)

Mean	SD	Min	Max	Obs
90.00	22.36	50.00	100.00	5

exg002_4: House Disposed: Other Relatives(%)

Mean	SD	Min	Max	Obs
100.00	0.00	100.00	100.00	3

exg002_5: House Disposed: Parents/Parents-in-laws(%)

No Observations

exg002_7: House Disposed: Friends(%)

No Observations

exg002_8: House Disposed: Charity(%)

No Observations

exg002_9: House Disposed: Others(%)

Mean	SD	Min	Max	Obs
32.67	52.16	-1.00	100.00	6

exg003_s1: House Disposed: Child[1]/'s Spouse

	Freq.	%
0 No	57	40.14
1 Yes	85	59.86
Total	142	100.00

exg003_s2: House Disposed: Child[2]/'s Spouse

	Freq.	%
0 No	101	71.13
2 Yes	41	28.87
Total	142	100.00

exg003_s3: House Disposed: Child[3]/'s Spouse

	Freq.	%
0 No	119	83.80
3 Yes	23	16.20
Total	142	100.00

exg003_s4: House Disposed: Child[4]/'s Spouse

	Freq.	%
0 No	129	90.85
4 Yes	13	9.15
Total	142	100.00

exg003_s5: House Disposed: Child[5]/'s Spouse

	Freq.	%
0 No	133	93.66
5 Yes	9	6.34
Total	142	100.00

exg003_s6: House Disposed: Child[6]/'s Spouse

	Freq.	%
0 No	141	99.30
6 Yes	1	0.70
Total	142	100.00

exg003_s7: House Disposed: Child[7]/'s Spouse

	Freq.	%
0 No	142	100.00
Total	142	100.00

exg003_s8: House Disposed: Child[8]/'s Spouse

	Freq.	%
0 No	142	100.00
Total	142	100.00

exg003_s9: House Disposed: Child[9]/'s Spouse

	Freq.	%
0 No	142	100.00
Total	142	100.00

exg003_s10: House Disposed: Child[10]/'s Spouse

	Freq.	%
0 No	142	100.00
Total	142	100.00

exg003_s11: House Disposed: Child[11]/'s Spouse

	Freq.	%
0 No	142	100.00
Total	142	100.00

exg003_s12: House Disposed: Child[12]/'s Spouse

	Freq.	%
0 No	142	100.00
Total	142	100.00

exg003_s13: House Disposed: Child[13]/'s Spouse

	Freq.	%
0 No	142	100.00
Total	142	100.00

exg003_s14: House Disposed: Child[14]/'s Spouse

	Freq.	%
0 No	142	100.00
Total	142	100.00

exg003_s15: House Disposed: Child[15]/'s Spouse

	Freq.	%
0 No	142	100.00
Total	142	100.00

exg003_s16: House Disposed: Child[16]/'s Spouse

	Freq.	%
0 No	142	100.00
Total	142	100.00

exg003_s17: House Disposed: Child[17]/'s Spouse

	Freq.	%
0 No	142	100.00
Total	142	100.00

exg003_s18: House Disposed: Child[18]/'s Spouse

	Freq.	%
0 No	142	100.00
Total	142	100.00

exg003_s19: House Disposed: Child[19]/'s Spouse

	Freq.	%
0 No	142	100.00
Total	142	100.00

exg003_s20: House Disposed: Child[20]/'s Spouse

	Freq.	%
0 No	142	100.00
Total	142	100.00

exg003_s21: House Disposed: Child[21]/'s Spouse

	Freq.	%
0 No	142	100.00
Total	142	100.00

exg003_s22: House Disposed: Child[22]/'s Spouse

	Freq.	%
0 No	142	100.00
Total	142	100.00

exg003_s23: House Disposed: Child[23]/'s Spouse

	Freq.	%
0 No	142	100.00
Total	142	100.00

exg003_s24: House Disposed: Child[24]/'s Spouse

	Freq.	%
0 No	142	100.00
Total	142	100.00

exg003_s25: House Disposed: Child[25]/'s Spouse

	Freq.	%
0 No	142	100.00
Total	142	100.00

exg003_s26: House Disposed: Other Child[26]/'s Spouse

	Freq.	%
0 No	131	92.25
26 Yes	11	7.75

Total	142	100.00

exg003_s27: House Disposed: Other Child[27]/'s Spouse

	Freq.	%
0 No	142	100.00
Total	142	100.00

exg003_s28: House Disposed: Other Child[28]/'s Spouse

	Freq.	%
0 No	142	100.00
Total	142	100.00

exg003_s29: House Disposed: Other Child[29]/'s Spouse

	Freq.	%
0 No	142	100.00
Total	142	100.00

exg003_s30: House Disposed: Other Child[30]/'s Spouse

	Freq.	%
0 No	142	100.00
Total	142	100.00

exg003_s31: House Disposed: Other Child[31]/'s Spouse

	Freq.	%
0 No	142	100.00
Total	142	100.00

exg003_s32: House Disposed: Other Child[32]/'s Spouse

	Freq.	%
0 No	142	100.00
Total	142	100.00

exg003_s33: House Disposed: Other Child[33]/'s Spouse

	Freq.	%
0 No	142	100.00
Total	142	100.00

exg003_s34: House Disposed: Other Child[34]/'s Spouse

	Freq.	%

	Freq.	%
0 No	142	100.00
Total	142	100.00

exg003_s35: House Disposed: Other Child[35]/'s Spouse

	Freq.	%
0 No	142	100.00
Total	142	100.00

exg004_1_: House Disposed: Child[1]/'s Spouse(%)

Mean	SD	Min	Max	Obs
81.85	31.00	-1.00	100.00	85

exg004_2_: House Disposed: Child[2]/'s Spouse(%)

Mean	SD	Min	Max	Obs
54.43	36.55	-1.00	100.00	41

exg004_3_: House Disposed: Child[3]/'s Spouse(%)

Mean	SD	Min	Max	Obs
58.62	41.27	-1.00	100.00	23

exg004_4_: House Disposed: Child[4]/'s Spouse(%)

Mean	SD	Min	Max	Obs
74.46	41.66	-1.00	100.00	13

exg004_5_: House Disposed: Child[5]/'s Spouse(%)

Mean	SD	Min	Max	Obs
57.67	50.59	-1.00	100.00	9

exg004_6_: House Disposed: Child[6]/'s Spouse(%)

Mean	SD	Min	Max	Obs
-1.00	.	-1.00	-1.00	1

exg004_26_: House Disposed: Other Child[26]/'s Spouse(%)

Mean	SD	Min	Max	Obs
86.36	23.35	50.00	100.00	11

exg004_27_: House Disposed: Other Child[27]/'s Spouse(%)

No Observations

exg007_s1: House Disposed: Child[1]'s Children

	Freq.	%
0 No	5	83.33
1 Yes	1	16.67
Total	6	100.00

exg007_s2: House Disposed: Child[2]'s Children

	Freq.	%
0 No	4	66.67
2 Yes	2	33.33
Total	6	100.00

exg007_s3: House Disposed: Child[3]'s Children

	Freq.	%
0 No	6	100.00
Total	6	100.00

exg007_s4: House Disposed: Child[4]'s Children

	Freq.	%
0 No	5	83.33
4 Yes	1	16.67
Total	6	100.00

exg007_s5: House Disposed: Child[5]'s Children

	Freq.	%
0 No	6	100.00
Total	6	100.00

exg007_s6: House Disposed: Child[6]'s Children

	Freq.	%
0 No	6	100.00
Total	6	100.00

exg007_s7: House Disposed: Child[7]'s Children

	Freq.	%
0 No	6	100.00
Total	6	100.00

exg007_s8: House Disposed: Child[8]'s Children

	Freq.	%
0 No	6	100.00
Total	6	100.00

exg007_s9: House Disposed: Child[9]'s Children

	Freq.	%
0 No	6	100.00
Total	6	100.00

exg007_s10: House Disposed: Child[10]'s Children

	Freq.	%
0 No	6	100.00
Total	6	100.00

exg007_s11: House Disposed: Child[11]'s Children

	Freq.	%
0 No	6	100.00
Total	6	100.00

exg007_s12: House Disposed: Child[12]'s Children

	Freq.	%
0 No	6	100.00
Total	6	100.00

exg007_s13: House Disposed: Child[13]'s Children

	Freq.	%
0 No	6	100.00
Total	6	100.00

exg007_s14: House Disposed: Child[14]'s Children

	Freq.	%
0 No	6	100.00
Total	6	100.00

exg007_s15: House Disposed: Child[15]'s Children

	Freq.	%
0 No	6	100.00
Total	6	100.00

exg007_s16: House Disposed: Child[16]'s Children

	Freq.	%
0 No	6	100.00
Total	6	100.00

exg007_s17: House Disposed: Child[17]'s Children

	Freq.	%
0 No	6	100.00
Total	6	100.00

exg007_s18: House Disposed: Child[18]'s Children

	Freq.	%
0 No	6	100.00
Total	6	100.00

exg007_s19: House Disposed: Child[19]'s Children

	Freq.	%
0 No	6	100.00
Total	6	100.00

exg007_s20: House Disposed: Child[20]'s Children

	Freq.	%
0 No	6	100.00
Total	6	100.00

exg007_s21: House Disposed: Child[21]'s Children

	Freq.	%
0 No	6	100.00
Total	6	100.00

exg007_s22: House Disposed: Child[22]'s Children

	Freq.	%
0 No	6	100.00
Total	6	100.00

exg007_s23: House Disposed: Child[23]'s Children

	Freq.	%
0 No	6	100.00
Total	6	100.00

exg007_s24: House Disposed: Child[24]'s Children

	Freq.	%
0 No	6	100.00
Total	6	100.00

exg007_s25: House Disposed: Child[25]'s Children

	Freq.	%
0 No	6	100.00
Total	6	100.00

exg007_s26: House Disposed: Other Child[26]'s Children

	Freq.	%
0 No	4	66.67
26 Yes	2	33.33
Total	6	100.00

exg007_s27: House Disposed: Other Child[27]'s Children

	Freq.	%
0 No	6	100.00
Total	6	100.00

exg007_s28: House Disposed: Other Child[28]'s Children

	Freq.	%
0 No	6	100.00
Total	6	100.00

exg007_s29: House Disposed: Other Child[29]'s Children

	Freq.	%
0 No	6	100.00
Total	6	100.00

exg007_s30: House Disposed: Other Child[30]'s Children

	Freq.	%
0 No	6	100.00
Total	6	100.00

exg007_s31: House Disposed: Other Child[31]'s Children

	Freq.	%
0 No	6	100.00

	Freq.	%
Total	6	100.00

exg007_s32: House Disposed: Other Child[32]'s Children

	Freq.	%
0 No	6	100.00
Total	6	100.00

exg007_s33: House Disposed: Other Child[33]'s Children

	Freq.	%
0 No	6	100.00
Total	6	100.00

exg007_s34: House Disposed: Other Child[34]'s Children

	Freq.	%
0 No	6	100.00
Total	6	100.00

exg007_s35: House Disposed: Other Child[35]'s Children

	Freq.	%
0 No	6	100.00
Total	6	100.00

exg008_1_: House Disposed: Child[1]'s Children(%)

Mean	SD	Min	Max	Obs
100.00	.	100.00	100.00	1

exg008_2_: House Disposed: Child[2]'s Children(%)

Mean	SD	Min	Max	Obs
100.00	0.00	100.00	100.00	2

exg008_3_: House Disposed: Child[3]'s Children(%)

No Observations

exg008_4_: House Disposed: Child[4]'s Children(%)

Mean	SD	Min	Max	Obs
100.00	.	100.00	100.00	1

exg008_26_: House Disposed: Other Child[26]'s Children(%)

Mean	SD	Min	Max	Obs
75.00	35.36	50.00	100.00	2

exg009: Cash Left

	Freq.	%
1 Yes	167	21.97
2 No	593	78.03
Total	760	100.00

exg010: Cash Amount

Mean	SD	Min	Max	Obs
10,930.83	49,402.54	-1.00	600,000.00	167

exg011: Deposit

	Freq.	%
1 Yes	113	14.87
2 No	647	85.13
Total	760	100.00

exg012: Deposit Amount

Mean	SD	Min	Max	Obs
54,379.81	99,445.32	-1.00	600,000.00	113

exg013: Financial Asset

	Freq.	%
1 Yes	3	0.39
2 No	757	99.61
Total	760	100.00

exg014: Financial Asset Amount

Mean	SD	Min	Max	Obs
13,833.33	14,631.59	1,500.00	30,000.00	3

exg015: In-kind

	Freq.	%
1 Yes	125	16.45
2 No	635	83.55
Total	760	100.00

exg016: Value of In-kind

Mean	SD	Min	Max	Obs
10,588.25	23,912.15	-1.00	150,000.00	125

exg017: Value of Other Legacy

Mean	SD	Min	Max	Obs
46.68	666.86	-1.00	14,000.00	760

exg018: Total Value Correct

	Freq.	%
1 Yes	695	91.45
2 No	65	8.55
Total	760	100.00

exg018_1: The Correct Total Value

Mean	SD	Min	Max	Obs
30,669.55	87,425.53	-1.00	600,000.00	65

exg019: Make a Will

	Freq.	%
1 Yes	19	2.50
2 No	741	97.50
Total	760	100.00

exg020: Was Will Notarized

	Freq.	%
1 Yes	2	10.53
2 No	17	89.47
Total	19	100.00

exg021_s1: Estate Planning: Spouse

	Freq.	%
0 No	18	94.74
1 Yes	1	5.26
Total	19	100.00

exg021_s2: Estate Planning: Children/Sons-in-law/Daughters-in-law

	Freq.	%
0 No	5	26.32

	Freq.	%
2 Yes	14	73.68
Total	19	100.00

exg021_s3: Estate Planning: Sibling

	Freq.	%
0 No	19	100.00
Total	19	100.00

exg021_s4: Estate Planning: Other Relatives

	Freq.	%
0 No	19	100.00
Total	19	100.00

exg021_s5: Estate Planning: Parents/Parents-in-law

	Freq.	%
0 No	19	100.00
Total	19	100.00

exg021_s6: Estate Planning: Grandchildren

	Freq.	%
0 No	17	89.47
6 Yes	2	10.53
Total	19	100.00

exg021_s7: Estate Planning: Friends

	Freq.	%
0 No	19	100.00
Total	19	100.00

exg021_s8: Estate Planning: Charity

	Freq.	%
0 No	19	100.00
Total	19	100.00

exg021_s9: Estate Planning: Others

	Freq.	%
0 No	18	94.74
9 Yes	1	5.26
Total	19	100.00

exg021_s10: Estate Planning: No Arrangement

	Freq.	%
0 No	15	78.95
10 Yes	4	21.05
Total	19	100.00

exg021_1: Estate Planning: Spouse(%)

Mean	SD	Min	Max	Obs
20.00	.	20.00	20.00	1

exg021_3: Estate Planning: Sibling(%)

No Observations

exg021_4: Estate Planning: Other Relatives(%)

No Observations

exg021_5: Estate Planning: Parents/Parents-in-law(%)

No Observations

exg021_7: Estate Planning: Friends(%)

No Observations

exg021_8: Estate Planning: Charity(%)

No Observations

exg021_9: Estate Planning: Others(%)

Mean	SD	Min	Max	Obs
100.00	.	100.00	100.00	1

exg022_s1: Estate Planning: Child[1]/'s Spouse

	Freq.	%
0 No	4	28.57
1 Yes	10	71.43
Total	14	100.00

exg022_s2: Estate Planning: Child[2]/'s Spouse

	Freq.	%
0 No	8	57.14

	Freq.	%
2 Yes	6	42.86
Total	14	100.00

exg022_s3: Estate Planning: Child[3]/'s Spouse

	Freq.	%
0 No	10	71.43
3 Yes	4	28.57
Total	14	100.00

exg022_s4: Estate Planning: Child[4]/'s Spouse

	Freq.	%
0 No	9	64.29
4 Yes	5	35.71
Total	14	100.00

exg022_s5: Estate Planning: Child[5]/'s Spouse

	Freq.	%
0 No	13	92.86
5 Yes	1	7.14
Total	14	100.00

exg022_s6: Estate Planning: Child[6]/'s Spouse

	Freq.	%
0 No	14	100.00
Total	14	100.00

exg022_s7: Estate Planning: Child[7]/'s Spouse

	Freq.	%
0 No	14	100.00
Total	14	100.00

exg022_s8: Estate Planning: Child[8]/'s Spouse

	Freq.	%
0 No	14	100.00
Total	14	100.00

exg022_s9: Estate Planning: Child[9]/'s Spouse

	Freq.	%
0 No	14	100.00
Total	14	100.00

exg022_s10: Estate Planning: Child[10]/'s Spouse

	Freq.	%
0 No	14	100.00
Total	14	100.00

exg022_s11: Estate Planning: Child[11]/'s Spouse

	Freq.	%
0 No	14	100.00
Total	14	100.00

exg022_s12: Estate Planning: Child[12]/'s Spouse

	Freq.	%
0 No	14	100.00
Total	14	100.00

exg022_s13: Estate Planning: Child[13]/'s Spouse

	Freq.	%
0 No	14	100.00
Total	14	100.00

exg022_s14: Estate Planning: Child[14]/'s Spouse

	Freq.	%
0 No	14	100.00
Total	14	100.00

exg022_s15: Estate Planning: Child[15]/'s Spouse

	Freq.	%
0 No	14	100.00
Total	14	100.00

exg022_s16: Estate Planning: Child[16]/'s Spouse

	Freq.	%
0 No	14	100.00
Total	14	100.00

exg022_s17: Estate Planning: Child[17]/'s Spouse

	Freq.	%
0 No	14	100.00
Total	14	100.00

exg022_s18: Estate Planning: Child[18]/'s Spouse

	Freq.	%
0 No	14	100.00
Total	14	100.00

exg022_s19: Estate Planning: Child[19]/'s Spouse

	Freq.	%
0 No	14	100.00
Total	14	100.00

exg022_s20: Estate Planning: Child[20]/'s Spouse

	Freq.	%
0 No	14	100.00
Total	14	100.00

exg022_s21: Estate Planning: Child[21]/'s Spouse

	Freq.	%
0 No	14	100.00
Total	14	100.00

exg022_s22: Estate Planning: Child[22]/'s Spouse

	Freq.	%
0 No	14	100.00
Total	14	100.00

exg022_s23: Estate Planning: Child[23]/'s Spouse

	Freq.	%
0 No	14	100.00
Total	14	100.00

exg022_s24: Estate Planning: Child[24]/'s Spouse

	Freq.	%
0 No	14	100.00
Total	14	100.00

exg022_s25: Estate Planning: Child[25]/'s Spouse

	Freq.	%
0 No	14	100.00
Total	14	100.00

exg022_s26: Estate Planning: Other Child[26]/'s Spouse

	Freq.	%
0 No	14	100.00
Total	14	100.00

exg022_s27: Estate Planning: Other Child[27]/'s Spouse

	Freq.	%
0 No	14	100.00
Total	14	100.00

exg022_s28: Estate Planning: Other Child[28]/'s Spouse

	Freq.	%
0 No	14	100.00
Total	14	100.00

exg022_s29: Estate Planning: Other Child[29]/'s Spouse

	Freq.	%
0 No	14	100.00
Total	14	100.00

exg022_s30: Estate Planning: Other Child[30]/'s Spouse

	Freq.	%
0 No	14	100.00
Total	14	100.00

exg022_s31: Estate Planning: Other Child[31]/'s Spouse

	Freq.	%
0 No	14	100.00
Total	14	100.00

exg022_s32: Estate Planning: Other Child[32]/'s Spouse

	Freq.	%
0 No	14	100.00
Total	14	100.00

exg022_s33: Estate Planning: Other Child[33]/'s Spouse

	Freq.	%
0 No	14	100.00
Total	14	100.00

exg022_s34: Estate Planning: Other Child[34]/'s Spouse

	Freq.	%
0 No	14	100.00
Total	14	100.00

exg022_s35: Estate Planning: Other Child[35]/'s Spouse

	Freq.	%
0 No	14	100.00
Total	14	100.00

exg023_1_: Estate Planning: Child[1]/'s Spouse(%)

Mean	SD	Min	Max	Obs
56.13	42.30	-1.00	100.00	10

exg023_2_: Estate Planning: Child[2]/'s Spouse(%)

Mean	SD	Min	Max	Obs
31.33	38.50	-1.00	100.00	6

exg023_3_: Estate Planning: Child[3]/'s Spouse(%)

Mean	SD	Min	Max	Obs
25.57	21.55	-1.00	50.00	4

exg023_4_: Estate Planning: Child[4]/'s Spouse(%)

Mean	SD	Min	Max	Obs
50.46	46.85	-1.00	100.00	5

exg023_5_: Estate Planning: Child[5]/'s Spouse(%)

Mean	SD	Min	Max	Obs
-1.00	.	-1.00	-1.00	1

exg026_s1: Estate Planning: Child[1]'s Children

	Freq.	%
0 No	1	50.00
1 Yes	1	50.00
Total	2	100.00

exg026_s2: Estate Planning: Child[2]'s Children

	Freq.	%
0 No	2	100.00
Total	2	100.00

exg026_s3: Estate Planning: Child[3]'s Children

	Freq.	%
0 No	2	100.00
Total	2	100.00

exg026_s4: Estate Planning: Child[4]'s Children

	Freq.	%
0 No	2	100.00
Total	2	100.00

exg026_s5: Estate Planning: Child[5]'s Children

	Freq.	%
0 No	2	100.00
Total	2	100.00

exg026_s6: Estate Planning: Child[6]'s Children

	Freq.	%
0 No	2	100.00
Total	2	100.00

exg026_s7: Estate Planning: Child[7]'s Children

	Freq.	%
0 No	2	100.00
Total	2	100.00

exg026_s8: Estate Planning: Child[8]'s Children

	Freq.	%
0 No	2	100.00
Total	2	100.00

exg026_s9: Estate Planning: Child[9]'s Children

	Freq.	%
0 No	2	100.00
Total	2	100.00

exg026_s10: Estate Planning: Child[10]'s Children

	Freq.	%
0 No	2	100.00
Total	2	100.00

exg026_s11: Estate Planning: Child[11]'s Children

	Freq.	%
0 No	2	100.00
Total	2	100.00

exg026_s12: Estate Planning: Child[12]'s Children

	Freq.	%
0 No	2	100.00
Total	2	100.00

exg026_s13: Estate Planning: Child[13]'s Children

	Freq.	%
0 No	2	100.00
Total	2	100.00

exg026_s14: Estate Planning: Child[14]'s Children

	Freq.	%
0 No	2	100.00
Total	2	100.00

exg026_s15: Estate Planning: Child[15]'s Children

	Freq.	%
0 No	2	100.00
Total	2	100.00

exg026_s16: Estate Planning: Child[16]'s Children

	Freq.	%
0 No	2	100.00
Total	2	100.00

exg026_s17: Estate Planning: Child[17]'s Children

	Freq.	%
0 No	2	100.00
Total	2	100.00

exg026_s18: Estate Planning: Child[18]'s Children

	Freq.	%
0 No	2	100.00
Total	2	100.00

exg026_s19: Estate Planning: Child[19]'s Children

	Freq.	%
0 No	2	100.00
Total	2	100.00

exg026_s20: Estate Planning: Child[20]'s Children

	Freq.	%
0 No	2	100.00
Total	2	100.00

exg026_s21: Estate Planning: Child[21]'s Children

	Freq.	%
0 No	2	100.00
Total	2	100.00

exg026_s22: Estate Planning: Child[22]'s Children

	Freq.	%
0 No	2	100.00
Total	2	100.00

exg026_s23: Estate Planning: Child[23]'s Children

	Freq.	%
0 No	2	100.00
Total	2	100.00

exg026_s24: Estate Planning: Child[24]'s Children

	Freq.	%
0 No	2	100.00
Total	2	100.00

exg026_s25: Estate Planning: Child[25]'s Children

	Freq.	%
0 No	2	100.00
Total	2	100.00

exg026_s26: Estate Planning: Other Child[26]'s Children

	Freq.	%
0 No	1	50.00
26 Yes	1	50.00
Total	2	100.00

exg026_s27: Estate Planning: Other Child[27]'s Children

	Freq.	%
0 No	2	100.00
Total	2	100.00

exg026_s28: Estate Planning: Other Child[28]'s Children

	Freq.	%
0 No	2	100.00
Total	2	100.00

exg026_s29: Estate Planning: Other Child[29]'s Children

	Freq.	%
0 No	2	100.00
Total	2	100.00

exg026_s30: Estate Planning: Other Child[30]'s Children

	Freq.	%
0 No	2	100.00
Total	2	100.00

exg026_s31: Estate Planning: Other Child[31]'s Children

	Freq.	%
0 No	2	100.00
Total	2	100.00

exg026_s32: Estate Planning: Other Child[32]'s Children

	Freq.	%
0 No	2	100.00
Total	2	100.00

exg026_s33: Estate Planning: Other Child[33]'s Children

	Freq.	%
0 No	2	100.00

	Freq.	%
Total	2	100.00

exg026_s34: Estate Planning: Other Child[34]'s Children

	Freq.	%
0 No	2	100.00
Total	2	100.00

exg026_s35: Estate Planning: Other Child[35]'s Children

	Freq.	%
0 No	2	100.00
Total	2	100.00

exg027_1_: Estate Planning: Child[1]'s Children(%)

Mean	SD	Min	Max	Obs
20.00	.	20.00	20.00	1

exg027_26_: Estate Planning: Other Child[26]'s Children(%)

Mean	SD	Min	Max	Obs
50.00	.	50.00	50.00	1

exg028: Equal Share among Sibling for Sibling Share

No Observations

exg029: Any Estate is Inherited

	Freq.	%
1 Yes	95	12.50
2 No	665	87.50
Total	760	100.00

exg029_1: Total Value of Estate Inherited

Mean	SD	Min	Max	Obs
42,776.55	80,259.27	-1.00	560,000.00	95

exg030: Follow the Will

	Freq.	%
1 Yes	2	66.67
2 No	1	33.33
Total	3	100.00

exg031_s1: Legacy Disposed without Housing: Spouse

	Freq.	%
0 No	68	73.12
1 Yes	25	26.88
Total	93	100.00

exg031_s2: Legacy Disposed without Housing: Children/Sons-in-law/Daughters-in-law

	Freq.	%
0 No	38	40.86
2 Yes	55	59.14
Total	93	100.00

exg031_s3: Legacy Disposed without Housing: Sibling

	Freq.	%
0 No	92	98.92
3 Yes	1	1.08
Total	93	100.00

exg031_s4: Legacy Disposed without Housing: Other Relatives

	Freq.	%
0 No	92	98.92
4 Yes	1	1.08
Total	93	100.00

exg031_s5: Legacy Disposed without Housing: Parents/Parents-in-law

	Freq.	%
0 No	93	100.00
Total	93	100.00

exg031_s6: Legacy Disposed without Housing: Grandchildren

	Freq.	%
0 No	91	97.85
6 Yes	2	2.15
Total	93	100.00

exg031_s7: Legacy Disposed without Housing: Friends

	Freq.	%
0 No	93	100.00
Total	93	100.00

exg031_s8: Legacy Disposed without Housing: Charity

	Freq.	%
0 No	93	100.00
Total	93	100.00

exg031_s9: Legacy Disposed without Housing: Others

	Freq.	%
0 No	91	97.85
9 Yes	2	2.15
Total	93	100.00

exg031_s10: Legacy Disposed without Housing: No Distribution

	Freq.	%
0 No	80	86.02
10 Yes	13	13.98
Total	93	100.00

exg031_1: Legacy Disposed without Housing: Spouse(%)

Mean	SD	Min	Max	Obs
69.80	45.45	-1.00	100.00	25

exg031_3: Legacy Disposed without Housing: Sibling(%)

Mean	SD	Min	Max	Obs
25.00	.	25.00	25.00	1

exg031_4: Legacy Disposed without Housing: Other Relatives(%)

Mean	SD	Min	Max	Obs
100.00	.	100.00	100.00	1

exg031_5: Legacy Disposed without Housing: Parents/Parents-in-law(%)

No Observations

exg031_7: Legacy Disposed without Housing: Friends(%)

No Observations

exg031_8: Legacy Disposed without Housing: Charity(%)

No Observations

exg031_9: Legacy Disposed without Housing: Others(%)

Mean	SD	Min	Max	Obs
-1.00	0.00	-1.00	-1.00	2

exg032_s1: Legacy Disposed without Housing: Child[1]/'s Spouse

	Freq.	%
0 No	22	40.00
1 Yes	33	60.00
Total	55	100.00

exg032_s2: Legacy Disposed without Housing: Child[2]/'s Spouse

	Freq.	%
0 No	32	58.18
2 Yes	23	41.82
Total	55	100.00

exg032_s3: Legacy Disposed without Housing: Child[3]/'s Spouse

	Freq.	%
0 No	36	65.45
3 Yes	19	34.55
Total	55	100.00

exg032_s4: Legacy Disposed without Housing: Child[4]/'s Spouse

	Freq.	%
0 No	40	72.73
4 Yes	15	27.27
Total	55	100.00

exg032_s5: Legacy Disposed without Housing: Child[5]/'s Spouse

	Freq.	%
0 No	49	89.09
5 Yes	6	10.91
Total	55	100.00

exg032_s6: Legacy Disposed without Housing: Child[6]/'s Spouse

	Freq.	%
0 No	53	96.36
6 Yes	2	3.64
Total	55	100.00

exg032_s7: Legacy Disposed without Housing: Child[7]/'s Spouse

	Freq.	%
0 No	54	98.18
7 Yes	1	1.82
Total	55	100.00

exg032_s8: Legacy Disposed without Housing: Child[8]/'s Spouse

	Freq.	%
0 No	54	98.18
8 Yes	1	1.82
Total	55	100.00

exg032_s9: Legacy Disposed without Housing: Child[9]/'s Spouse

	Freq.	%
0 No	55	100.00
Total	55	100.00

exg032_s10: Legacy Disposed without Housing: Child[10]/'s Spouse

	Freq.	%
0 No	55	100.00
Total	55	100.00

exg032_s11: Legacy Disposed without Housing: Child[11]/'s Spouse

	Freq.	%
0 No	54	98.18
11 Yes	1	1.82
Total	55	100.00

exg032_s12: Legacy Disposed without Housing: Child[12]/'s Spouse

	Freq.	%
0 No	55	100.00
Total	55	100.00

exg032_s13: Legacy Disposed without Housing: Child[13]/'s Spouse

	Freq.	%
0 No	55	100.00
Total	55	100.00

exg032_s14: Legacy Disposed without Housing: Child[14]/'s Spouse

	Freq.	%
0 No	55	100.00

	Freq.	%
Total	55	100.00

exg032_s15: Legacy Disposed without Housing: Child[15]/'s Spouse

	Freq.	%
0 No	55	100.00
Total	55	100.00

exg032_s16: Legacy Disposed without Housing: Child[16]/'s Spouse

	Freq.	%
0 No	55	100.00
Total	55	100.00

exg032_s17: Legacy Disposed without Housing: Child[17]/'s Spouse

	Freq.	%
0 No	55	100.00
Total	55	100.00

exg032_s18: Legacy Disposed without Housing: Child[18]/'s Spouse

	Freq.	%
0 No	55	100.00
Total	55	100.00

exg032_s19: Legacy Disposed without Housing: Child[19]/'s Spouse

	Freq.	%
0 No	55	100.00
Total	55	100.00

exg032_s20: Legacy Disposed without Housing: Child[20]/'s Spouse

	Freq.	%
0 No	55	100.00
Total	55	100.00

exg032_s21: Legacy Disposed without Housing: Child[21]/'s Spouse

	Freq.	%
0 No	55	100.00
Total	55	100.00

exg032_s22: Legacy Disposed without Housing: Child[22]/'s Spouse

	Freq.	%

	Freq.	%
0 No	55	100.00
Total	55	100.00

exg032_s23: Legacy Disposed without Housing: Child[23]/'s Spouse

	Freq.	%
0 No	55	100.00
Total	55	100.00

exg032_s24: Legacy Disposed without Housing: Child[24]/'s Spouse

	Freq.	%
0 No	55	100.00
Total	55	100.00

exg032_s25: Legacy Disposed without Housing: Child[25]/'s Spouse

	Freq.	%
0 No	55	100.00
Total	55	100.00

exg032_s26: Legacy Disposed without Housing: Other Child[26]/'s Spouse

	Freq.	%
0 No	53	96.36
26 Yes	2	3.64
Total	55	100.00

exg032_s27: Legacy Disposed without Housing: Other Child[27]/'s Spouse

	Freq.	%
0 No	54	98.18
27 Yes	1	1.82
Total	55	100.00

exg032_s28: Legacy Disposed without Housing: Other Child[28]/'s Spouse

	Freq.	%
0 No	54	98.18
28 Yes	1	1.82
Total	55	100.00

exg032_s29: Legacy Disposed without Housing: Other Child[29]/'s Spouse

	Freq.	%
0 No	54	98.18
29 Yes	1	1.82
Total	55	100.00

exg032_s30: Legacy Disposed without Housing: Other Child[30]/'s Spouse

	Freq.	%
0 No	54	98.18
30 Yes	1	1.82
Total	55	100.00

exg032_s31: Legacy Disposed without Housing: Other Child[31]/'s Spouse

	Freq.	%
0 No	55	100.00
Total	55	100.00

exg032_s32: Legacy Disposed without Housing: Other Child[32]/'s Spouse

	Freq.	%
0 No	55	100.00
Total	55	100.00

exg032_s33: Legacy Disposed without Housing: Other Child[33]/'s Spouse

	Freq.	%
0 No	55	100.00
Total	55	100.00

exg032_s34: Legacy Disposed without Housing: Other Child[34]/'s Spouse

	Freq.	%
0 No	55	100.00
Total	55	100.00

exg032_s35: Legacy Disposed without Housing: Other Child[35]/'s Spouse

	Freq.	%
0 No	55	100.00
Total	55	100.00

exg033_1_: Legacy Disposed without Housing: Child[1]/'s Spouse(%)

Mean	SD	Min	Max	Obs
63.60	41.38	-1.00	100.00	33

exg033_2_: Legacy Disposed without Housing: Child[2]/'s Spouse(%)

Mean	SD	Min	Max	Obs
35.17	31.56	-1.00	100.00	23

exg033_3_: Legacy Disposed without Housing: Child[3]/'s Spouse(%)

Mean	SD	Min	Max	Obs
42.15	37.93	-1.00	100.00	19

exg033_4_: Legacy Disposed without Housing: Child[4]/'s Spouse(%)

Mean	SD	Min	Max	Obs
40.19	30.38	3.60	100.00	15

exg033_5_: Legacy Disposed without Housing: Child[5]/'s Spouse(%)

Mean	SD	Min	Max	Obs
20.47	15.71	3.60	50.00	6

exg033_6_: Legacy Disposed without Housing: Child[6]/'s Spouse(%)

Mean	SD	Min	Max	Obs
14.60	2.97	12.50	16.70	2

exg033_7_: Legacy Disposed without Housing: Child[7]/'s Spouse(%)

Mean	SD	Min	Max	Obs
12.50	.	12.50	12.50	1

exg033_8_: Legacy Disposed without Housing: Child[8]/'s Spouse(%)

Mean	SD	Min	Max	Obs
12.50	.	12.50	12.50	1

exg033_9_: Legacy Disposed without Housing: Child[9]/'s Spouse(%)

No Observations

exg033_10_: Legacy Disposed without Housing: Child[10]/'s Spouse(%)

No Observations

exg033_11_: Legacy Disposed without Housing: Child[11]/'s Spouse(%)

Mean	SD	Min	Max	Obs
100.00	.	100.00	100.00	1

exg033_12_: Legacy Disposed without Housing: Child[12]/'s Spouse(%)

No Observations

exg033_26_: Legacy Disposed without Housing: Other Child[26]/'s Spouse(%)

Mean	SD	Min	Max	Obs
62.50	53.03	25.00	100.00	2

exg033_27_: Legacy Disposed without Housing: Other Child[27]/'s Spouse(%)

Mean	SD	Min	Max	Obs
25.00	.	25.00	25.00	1

exg033_28_: Legacy Disposed without Housing: Other Child[28]/'s Spouse(%)

Mean	SD	Min	Max	Obs
25.00	.	25.00	25.00	1

exg033_29_: Legacy Disposed without Housing: Other Child[29]/'s Spouse(%)

Mean	SD	Min	Max	Obs
25.00	.	25.00	25.00	1

exg033_30_: Legacy Disposed without Housing: Other Child[30]/'s Spouse(%)

Mean	SD	Min	Max	Obs
25.00	.	25.00	25.00	1

exg036_s1: Legacy Disposed without Housing: Child[1]'s Children

	Freq.	%
0 No	1	50.00
1 Yes	1	50.00
Total	2	100.00

exg036_s2: Legacy Disposed without Housing: Child[2]'s Children

	Freq.	%
0 No	1	50.00
2 Yes	1	50.00
Total	2	100.00

exg036_s3: Legacy Disposed without Housing: Child[3]'s Children

	Freq.	%
0 No	1	50.00
3 Yes	1	50.00

	Freq.	%
Total	2	100.00

exg036_s4: Legacy Disposed without Housing: Child[4]'s Children

	Freq.	%
0 No	1	50.00
4 Yes	1	50.00
Total	2	100.00

exg036_s5: Legacy Disposed without Housing: Child[5]'s Children

	Freq.	%
0 No	1	50.00
5 Yes	1	50.00
Total	2	100.00

exg036_s6: Legacy Disposed without Housing: Child[6]'s Children

	Freq.	%
0 No	2	100.00
Total	2	100.00

exg036_s7: Legacy Disposed without Housing: Child[7]'s Children

	Freq.	%
0 No	2	100.00
Total	2	100.00

exg036_s8: Legacy Disposed without Housing: Child[8]'s Children

	Freq.	%
0 No	2	100.00
Total	2	100.00

exg036_s9: Legacy Disposed without Housing: Child[9]'s Children

	Freq.	%
0 No	2	100.00
Total	2	100.00

exg036_s10: Legacy Disposed without Housing: Child[10]'s Children

	Freq.	%
0 No	2	100.00
Total	2	100.00

exg036_s11: Legacy Disposed without Housing: Child[11]'s Children

	Freq.	%
0 No	2	100.00
Total	2	100.00

exg036_s12: Legacy Disposed without Housing: Child[12]'s Children

	Freq.	%
0 No	2	100.00
Total	2	100.00

exg036_s13: Legacy Disposed without Housing: Child[13]'s Children

	Freq.	%
0 No	2	100.00
Total	2	100.00

exg036_s14: Legacy Disposed without Housing: Child[14]'s Children

	Freq.	%
0 No	2	100.00
Total	2	100.00

exg036_s15: Legacy Disposed without Housing: Child[15]'s Children

	Freq.	%
0 No	2	100.00
Total	2	100.00

exg036_s16: Legacy Disposed without Housing: Child[16]'s Children

	Freq.	%
0 No	2	100.00
Total	2	100.00

exg036_s17: Legacy Disposed without Housing: Child[17]'s Children

	Freq.	%
0 No	2	100.00
Total	2	100.00

exg036_s18: Legacy Disposed without Housing: Child[18]'s Children

	Freq.	%
0 No	2	100.00
Total	2	100.00

exg036_s19: Legacy Disposed without Housing: Child[19]'s Children

	Freq.	%
0 No	2	100.00
Total	2	100.00

exg036_s20: Legacy Disposed without Housing: Child[20]'s Children

	Freq.	%
0 No	2	100.00
Total	2	100.00

exg036_s21: Legacy Disposed without Housing: Child[21]'s Children

	Freq.	%
0 No	2	100.00
Total	2	100.00

exg036_s22: Legacy Disposed without Housing: Child[22]'s Children

	Freq.	%
0 No	2	100.00
Total	2	100.00

exg036_s23: Legacy Disposed without Housing: Child[23]'s Children

	Freq.	%
0 No	2	100.00
Total	2	100.00

exg036_s24: Legacy Disposed without Housing: Child[24]'s Children

	Freq.	%
0 No	2	100.00
Total	2	100.00

exg036_s25: Legacy Disposed without Housing: Child[25]'s Children

	Freq.	%
0 No	2	100.00
Total	2	100.00

exg036_s26: Legacy Disposed without Housing: Other Child[26]'s Children

	Freq.	%
0 No	1	50.00
26 Yes	1	50.00

Total	2	100.00

exg036_s27: Legacy Disposed without Housing: Other Child[27]'s Children

	Freq.	%
0 No	2	100.00
Total	2	100.00

exg036_s28: Legacy Disposed without Housing: Other Child[28]'s Children

	Freq.	%
0 No	2	100.00
Total	2	100.00

exg036_s29: Legacy Disposed without Housing: Other Child[29]'s Children

	Freq.	%
0 No	2	100.00
Total	2	100.00

exg036_s30: Legacy Disposed without Housing: Other Child[30]'s Children

	Freq.	%
0 No	2	100.00
Total	2	100.00

exg036_s31: Legacy Disposed without Housing: Other Child[31]'s Children

	Freq.	%
0 No	2	100.00
Total	2	100.00

exg036_s32: Legacy Disposed without Housing: Other Child[32]'s Children

	Freq.	%
0 No	2	100.00
Total	2	100.00

exg036_s33: Legacy Disposed without Housing: Other Child[33]'s Children

	Freq.	%
0 No	2	100.00
Total	2	100.00

exg036_s34: Legacy Disposed without Housing: Other Child[34]'s Children

	Freq.	%

	Freq.	%
0 No	2	100.00
Total	2	100.00

exg036_s35: Legacy Disposed without Housing: Other Child[35]'s Children

	Freq.	%
0 No	2	100.00
Total	2	100.00

exg037_1_: Legacy Disposed without Housing: Child[1]'s Children(%)

Mean	SD	Min	Max	Obs
3.60	.	3.60	3.60	1

exg037_2_: Legacy Disposed without Housing: Child[2]'s Children(%)

Mean	SD	Min	Max	Obs
1.80	.	1.80	1.80	1

exg037_3_: Legacy Disposed without Housing: Child[3]'s Children(%)

Mean	SD	Min	Max	Obs
3.60	.	3.60	3.60	1

exg037_4_: Legacy Disposed without Housing: Child[4]'s Children(%)

Mean	SD	Min	Max	Obs
3.60	.	3.60	3.60	1

exg037_5_: Legacy Disposed without Housing: Child[5]'s Children(%)

Mean	SD	Min	Max	Obs
3.60	.	3.60	3.60	1

exg037_26_: Legacy Disposed without Housing: Other Child[26]'s Children(%)

Mean	SD	Min	Max	Obs
25.00	.	25.00	25.00	1

k01: Kind of Burial

	Freq.	%
1 Cremation	398	52.37
2 Ground Burial	355	46.71
3 Sky Burial	4	0.53
5 Other, Please Specify	3	0.39
Total	760	100.00

k02: Where Was He/She Buried

	Freq.	%
1 Contracted Land	321	42.63
2 Collective Burial Ground	153	20.32
3 Commercial Burial Ground	74	9.83
4 Waster Land	69	9.16
5 Ashes Kept In Funeral Home	43	5.71
6 Other, Please Specify	93	12.35
Total	753	100.00

k03: How Much Does the Burial Ground Cost?

	Freq.	%
1 Yuan Total	221	97.36
2 Yuan Per Year	6	2.64
Total	227	100.00

k03_1: K03_1

Mean	SD	Min	Max	Obs
10,947.17	18,783.77	0.00	150,000.00	205

k03_2: K03_2

Mean	SD	Min	Max	Obs
16.67	40.82	0.00	100.00	6

k04: In Which Year Was the Burial Ground Purchased?

Mean	SD	Min	Max	Obs
2,016.52	6.99	1,956.00	2,020.00	227

k06_s1: Spouse

Mean	SD	Min	Max	Obs
0.18	0.38	0.00	1.00	227

k06_s2: Child

Mean	SD	Min	Max	Obs
0.81	0.98	0.00	2.00	227

k06_s3: Child

Mean	SD	Min	Max	Obs
0.81	1.33	0.00	3.00	227

k06_s4: Child

Mean	SD	Min	Max	Obs
0.76	1.57	0.00	4.00	227

k06_s5: Child

Mean	SD	Min	Max	Obs
0.64	1.67	0.00	5.00	227

k06_s6: Child

Mean	SD	Min	Max	Obs
0.32	1.35	0.00	6.00	227

k06_s7: Child

Mean	SD	Min	Max	Obs
0.31	1.44	0.00	7.00	227

k06_s8: Child

Mean	SD	Min	Max	Obs
0.11	0.92	0.00	8.00	227

k06_s9: Child

Mean	SD	Min	Max	Obs
0.08	0.84	0.00	9.00	227

k06_s10: Child

Mean	SD	Min	Max	Obs
0.09	0.94	0.00	10.00	227

k06_s11: Child

Mean	SD	Min	Max	Obs
0.05	0.73	0.00	11.00	227

k06_s12: Child

Mean	SD	Min	Max	Obs
0.00	0.00	0.00	0.00	227

k06_s13: Child

Mean	SD	Min	Max	Obs
0.00	0.00	0.00	0.00	227

k06_s14: Child

Mean	SD	Min	Max	Obs
0.00	0.00	0.00	0.00	227

k06_s15: Child

Mean	SD	Min	Max	Obs
0.00	0.00	0.00	0.00	227

k06_s16: Child

Mean	SD	Min	Max	Obs
0.00	0.00	0.00	0.00	227

k06_s17: Child

Mean	SD	Min	Max	Obs
0.00	0.00	0.00	0.00	227

k06_s18: Child

Mean	SD	Min	Max	Obs
0.00	0.00	0.00	0.00	227

k06_s19: Child

Mean	SD	Min	Max	Obs
0.00	0.00	0.00	0.00	227

k06_s20: Child

Mean	SD	Min	Max	Obs
0.00	0.00	0.00	0.00	227

k06_s21: Child

Mean	SD	Min	Max	Obs
0.00	0.00	0.00	0.00	227

k06_s22: Child

Mean	SD	Min	Max	Obs
0.00	0.00	0.00	0.00	227

k06_s23: Child

Mean	SD	Min	Max	Obs
0.00	0.00	0.00	0.00	227

k06_s24: Child

Mean	SD	Min	Max	Obs
0.00	0.00	0.00	0.00	227

k06_s25: Child

Mean	SD	Min	Max	Obs
0.00	0.00	0.00	0.00	227

k06_s26: Child

Mean	SD	Min	Max	Obs
0.00	0.00	0.00	0.00	227

k06_s27: Child's Spouse

Mean	SD	Min	Max	Obs
2.85	8.32	0.00	27.00	227

k06_s28: Child's Spouse

Mean	SD	Min	Max	Obs
2.34	7.77	0.00	28.00	227

k06_s29: Child's Spouse

Mean	SD	Min	Max	Obs
2.04	7.44	0.00	29.00	227

k06_s30: Child's Spouse

Mean	SD	Min	Max	Obs
1.32	6.17	0.00	30.00	227

k06_s31: Child's Spouse

Mean	SD	Min	Max	Obs
0.41	3.55	0.00	31.00	227

k06_s32: Child's Spouse

Mean	SD	Min	Max	Obs
0.56	4.22	0.00	32.00	227

k06_s33: Child's Spouse

Mean	SD	Min	Max	Obs
0.29	3.09	0.00	33.00	227

k06_s34: Child's Spouse

Mean	SD	Min	Max	Obs
0.30	3.18	0.00	34.00	227

k06_s35: Child's Spouse

Mean	SD	Min	Max	Obs
0.31	3.28	0.00	35.00	227

k06_s36: Child's Spouse

Mean	SD	Min	Max	Obs
0.16	2.39	0.00	36.00	227

k06_s37: Child's Spouse

Mean	SD	Min	Max	Obs
0.00	0.00	0.00	0.00	227

k06_s38: Child's Spouse

Mean	SD	Min	Max	Obs
0.00	0.00	0.00	0.00	227

k06_s39: Child's Spouse

Mean	SD	Min	Max	Obs
0.00	0.00	0.00	0.00	227

k06_s40: Child's Spouse

Mean	SD	Min	Max	Obs
0.00	0.00	0.00	0.00	227

k06_s41: Child's Spouse

Mean	SD	Min	Max	Obs
0.00	0.00	0.00	0.00	227

k06_s42: Child's Spouse

Mean	SD	Min	Max	Obs
0.00	0.00	0.00	0.00	227

k06_s43: Child's Spouse

Mean	SD	Min	Max	Obs
0.00	0.00	0.00	0.00	227

k06_s44: Child's Spouse

Mean	SD	Min	Max	Obs
0.00	0.00	0.00	0.00	227

k06_s45: Child's Spouse

Mean	SD	Min	Max	Obs
0.00	0.00	0.00	0.00	227

k06_s46: Child's Spouse

Mean	SD	Min	Max	Obs
0.00	0.00	0.00	0.00	227

k06_s47: Child's Spouse

Mean	SD	Min	Max	Obs
0.00	0.00	0.00	0.00	227

k06_s48: Child's Spouse

Mean	SD	Min	Max	Obs
0.00	0.00	0.00	0.00	227

k06_s49: Child's Spouse

Mean	SD	Min	Max	Obs
0.00	0.00	0.00	0.00	227

k06_s50: Child's Spouse

Mean	SD	Min	Max	Obs
0.00	0.00	0.00	0.00	227

k06_s51: Child's Spouse

Mean	SD	Min	Max	Obs
0.00	0.00	0.00	0.00	227

k06_s52: Grandson

Mean	SD	Min	Max	Obs
1.37	8.36	0.00	52.00	227

k07: Which Child Was the Parent of the Grandchild?

Mean	SD	Min	Max	Obs
2.00	2.00	1.00	6.00	6

k09_min: K09_min

A String Variable	
Obs:	89

k09_max: K09_max

A String Variable	
Obs:	89

k09: How Much Did the Whole Funeral Cost

Mean	SD	Min	Max	Obs
27,897.43	29,072.82	0.00	350,000.00	671

k10_s1: Spouse

Mean	SD	Min	Max	Obs
0.18	0.38	0.00	1.00	760

k10_s2: Child

Mean	SD	Min	Max	Obs
1.19	0.98	0.00	2.00	760

k10_s3: Child

Mean	SD	Min	Max	Obs
1.41	1.50	0.00	3.00	760

k10_s4: Child

Mean	SD	Min	Max	Obs
1.29	1.87	0.00	4.00	760

k10_s5: Child

Mean	SD	Min	Max	Obs
0.99	2.00	0.00	5.00	760

k10_s6: Child

Mean	SD	Min	Max	Obs
0.67	1.89	0.00	6.00	760

k10_s7: Child

Mean	SD	Min	Max	Obs
0.37	1.56	0.00	7.00	760

k10_s8: Child

Mean	SD	Min	Max	Obs
0.24	1.37	0.00	8.00	760

k10_s9: Child

Mean	SD	Min	Max	Obs
0.09	0.92	0.00	9.00	760

k10_s10: Child

Mean	SD	Min	Max	Obs
0.08	0.89	0.00	10.00	760

k10_s11: Child

Mean	SD	Min	Max	Obs
0.06	0.80	0.00	11.00	760

k10_s12: Child

Mean	SD	Min	Max	Obs
0.03	0.62	0.00	12.00	760

k10_s13: Child

Mean	SD	Min	Max	Obs
0.00	0.00	0.00	0.00	760

k10_s14: Child

Mean	SD	Min	Max	Obs
0.00	0.00	0.00	0.00	760

k10_s15: Child

Mean	SD	Min	Max	Obs
0.00	0.00	0.00	0.00	760

k10_s16: Child

Mean	SD	Min	Max	Obs
0.00	0.00	0.00	0.00	760

k10_s17: Child

Mean	SD	Min	Max	Obs
0.00	0.00	0.00	0.00	760

k10_s18: Child

Mean	SD	Min	Max	Obs
0.00	0.00	0.00	0.00	760

k10_s19: Child

Mean	SD	Min	Max	Obs
0.00	0.00	0.00	0.00	760

k10_s20: Child

Mean	SD	Min	Max	Obs
0.00	0.00	0.00	0.00	760

k10_s21: Child

Mean	SD	Min	Max	Obs
0.00	0.00	0.00	0.00	760

k10_s22: Child

Mean	SD	Min	Max	Obs
0.00	0.00	0.00	0.00	760

k10_s23: Child

Mean	SD	Min	Max	Obs
0.00	0.00	0.00	0.00	760

k10_s24: Child

Mean	SD	Min	Max	Obs
0.00	0.00	0.00	0.00	760

k10_s25: Child

Mean	SD	Min	Max	Obs
0.00	0.00	0.00	0.00	760

k10_s26: Child

Mean	SD	Min	Max	Obs
0.00	0.00	0.00	0.00	760

k10_s27: Child’s Spouse

Mean	SD	Min	Max	Obs
5.40	10.81	0.00	27.00	760

k10_s28: Child’s Spouse

Mean	SD	Min	Max	Obs
4.46	10.25	0.00	28.00	760

k10_s29: Child’s Spouse

Mean	SD	Min	Max	Obs
3.59	9.55	0.00	29.00	760

k10_s30: Child's Spouse

Mean	SD	Min	Max	Obs
2.25	7.91	0.00	30.00	760

k10_s31: Child's Spouse

Mean	SD	Min	Max	Obs
1.55	6.76	0.00	31.00	760

k10_s32: Child's Spouse

Mean	SD	Min	Max	Obs
0.84	5.13	0.00	32.00	760

k10_s33: Child's Spouse

Mean	SD	Min	Max	Obs
0.52	4.12	0.00	33.00	760

k10_s34: Child's Spouse

Mean	SD	Min	Max	Obs
0.13	2.13	0.00	34.00	760

k10_s35: Child's Spouse

Mean	SD	Min	Max	Obs
0.14	2.20	0.00	35.00	760

k10_s36: Child's Spouse

Mean	SD	Min	Max	Obs
0.09	1.85	0.00	36.00	760

k10_s37: Child's Spouse

Mean	SD	Min	Max	Obs
0.10	1.90	0.00	37.00	760

k10_s38: Child's Spouse

Mean	SD	Min	Max	Obs
0.00	0.00	0.00	0.00	760

k10_s39: Child's Spouse

Mean	SD	Min	Max	Obs
0.00	0.00	0.00	0.00	760

k10_s40: Child's Spouse

Mean	SD	Min	Max	Obs
0.00	0.00	0.00	0.00	760

k10_s41: Child's Spouse

Mean	SD	Min	Max	Obs
0.00	0.00	0.00	0.00	760

k10_s42: Child's Spouse

Mean	SD	Min	Max	Obs
0.00	0.00	0.00	0.00	760

k10_s43: Child's Spouse

Mean	SD	Min	Max	Obs
0.00	0.00	0.00	0.00	760

k10_s44: Child's Spouse

Mean	SD	Min	Max	Obs
0.00	0.00	0.00	0.00	760

k10_s45: Child's Spouse

Mean	SD	Min	Max	Obs
0.00	0.00	0.00	0.00	760

k10_s46: Child's Spouse

Mean	SD	Min	Max	Obs
0.00	0.00	0.00	0.00	760

k10_s47: Child's Spouse

Mean	SD	Min	Max	Obs
0.00	0.00	0.00	0.00	760

k10_s48: Child's Spouse

Mean	SD	Min	Max	Obs
0.00	0.00	0.00	0.00	760

k10_s49: Child's Spouse

Mean	SD	Min	Max	Obs
0.00	0.00	0.00	0.00	760

k10_s50: Child's Spouse

Mean	SD	Min	Max	Obs
0.00	0.00	0.00	0.00	760

k10_s51: Child's Spouse

Mean	SD	Min	Max	Obs
0.00	0.00	0.00	0.00	760

k10_s52: Grandson

Mean	SD	Min	Max	Obs
1.98	9.97	0.00	52.00	760

k10_s53: Granddaughter

Mean	SD	Min	Max	Obs
1.05	7.38	0.00	53.00	760

k10_s54: Relatives

Mean	SD	Min	Max	Obs
3.34	13.02	0.00	54.00	760

k10_s55: R's Union

Mean	SD	Min	Max	Obs
0.87	6.86	0.00	55.00	760

k10_s56: R's Insurance

Mean	SD	Min	Max	Obs
0.59	5.72	0.00	56.00	760

k10_s57: Other People

Mean	SD	Min	Max	Obs
3.15	13.03	0.00	57.00	760

k11: Which Child Was the Parent of the Grandchild?

Mean	SD	Min	Max	Obs
1.97	1.30	1.00	6.00	30

k13: How Much Money Did R's Family Receive From the Whole Funeral?

Mean	SD	Min	Max	Obs
19,983.78	34,567.49	0.00	400,000.00	637

k13_min: K13_min

A String Variable	
Obs:	123

k13_max: K13_max

A String Variable	
Obs:	123

exv001: COVID-19 Confirmed Infection

	Freq.	%
2 No	196	100.00
Total	196	100.00

exv002: Suspected Case

	Freq.	%
1 Yes	1	0.51
2 No	194	98.98
999 Refuse to Answer	1	0.51
Total	196	100.00

exv003: Days of Hospital Stay

	Freq.	%
2 No	1	100.00

Total	1	100.00

exv004_s1: Travels

	Freq.	%
0 Yes	196	100.00
Total	196	100.00

exv004_s2: Close contact of COVID cases

	Freq.	%
0 Yes	196	100.00
Total	196	100.00

exv004_s3: Building Lockdown

	Freq.	%
0 No	193	98.47
3 Yes	3	1.53
Total	196	100.00

exv004_s4: After Going to Hospital

	Freq.	%
0 No	195	99.49
4 Yes	1	0.51
Total	196	100.00

exv004_s5: Tested Positive

	Freq.	%
0 Yes	196	100.00
Total	196	100.00

exv004_s6: No Quarantine Experience

	Freq.	%
0 No	4	2.04
6 Yes	192	97.96
Total	196	100.00

exv004_s997: Do not Know

	Freq.	%
0 Yes	196	100.00
Total	196	100.00

exv004_s999: Refuse to Answer

	Freq.	%
0 Yes	196	100.00
Total	196	100.00

exv005: Quarantine Days

Mean	SD	Min	Max	Obs
31.75	21.73	7.00	60.00	4

exv006_s1: Hospital

	Freq.	%
0 No	3	75.00
1 Yes	1	25.00
Total	4	100.00

exv006_s2: Hotel

	Freq.	%
0 Yes	4	100.00
Total	4	100.00

exv006_s3: Own Residence

	Freq.	%
0 No	1	25.00
3 Yes	3	75.00
Total	4	100.00

exv006_s4: Others

	Freq.	%
0 Yes	4	100.00
Total	4	100.00

exv006_s999: Refuse to Answer

	Freq.	%
0 Yes	4	100.00
Total	4	100.00

exv007: Quarantine Co-resident

	Freq.	%
1 Yes	4	100.00
Total	4	100.00

exv008: COVID Tested

	Freq.	%
1 Yes	13	6.63
2 No	182	92.86
999 Refuse to Answer	1	0.51
Total	196	100.00

exv008_1: COVID Tested Month

Mean	SD	Min	Max	Obs
3.69	2.63	-1.00	7.00	13

exv009: Days of Self-isolation

Mean	SD	Min	Max	Obs
18.44	40.25	-1.00	240.00	196

exv010: Treatment Delayed

	Freq.	%
1 Yes	28	14.29
2 No	168	85.71
Total	196	100.00

exv011_s1: 1. No Appointment Could Be Made or Regular Hospital Appointments Were Cancelled

	Freq.	%
0 No	22	78.57
1 Yes	6	21.43
Total	28	100.00

exv011_s2: 2. Hospitals Rescheduled All Regular Appointments

	Freq.	%
0 No	27	96.43
2 Yes	1	3.57
Total	28	100.00

exv011_s3: 3. Decided to Wait

	Freq.	%
0 No	24	85.71
3 Yes	4	14.29
Total	28	100.00

exv011_s4: 4. Was Afraid of Going to the Hospital

	Freq.	%
0 No	24	85.71
4 Yes	4	14.29
Total	28	100.00

exv011_s5: 5. Other, Please Specify

	Freq.	%
0 No	12	42.86
5 Yes	16	57.14
Total	28	100.00

exv012_s1: 1. Major Surgery Requiring Hospitalization

	Freq.	%
0 No	25	89.29
1 Yes	3	10.71
Total	28	100.00

exv012_s2: 2. Minor Surgery that Can Be Done on An Outpatient Basis or in A Day

	Freq.	%
0 No	26	92.86
2 Yes	2	7.14
Total	28	100.00

exv012_s3: 3. To See A Doctor for A Regular Outpatient Visit

	Freq.	%
0 No	16	57.14
3 Yes	12	42.86
Total	28	100.00

exv012_s4: 4. To Get Prescription Drugs

	Freq.	%
0 No	23	82.14
4 Yes	5	17.86
Total	28	100.00

exv012_s5: 5. To See A Dentist for Oral Treatment

	Freq.	%
0 No	27	96.43
5 Yes	1	3.57
Total	28	100.00

exv012_s6: 6. Other, Please Specify

	Freq.	%
0 No	17	60.71
6 Yes	11	39.29
Total	28	100.00

exv013_s1: 1. A New Symptom or Disease

	Freq.	%
0 No	11	91.67
1 Yes	1	8.33
Total	12	100.00

exv013_s2: 2. Treatment of An Existing Condition

	Freq.	%
0 No	1	8.33
2 Yes	11	91.67
Total	12	100.00

exv013_s3: 3. Routine Medical Screening

	Freq.	%
0 No	10	83.33
3 Yes	2	16.67
Total	12	100.00

exv014_s1: 1. Due to Confirmed Infection with COVID-19

	Freq.	%
0 Yes	196	100.00
Total	196	100.00

exv014_s2: 2. Unable to Have Timely Access to Routine Medical Care

	Freq.	%
0 No	180	91.84
2 Yes	16	8.16
Total	196	100.00

exv014_s3: 3. Unable to Receive Emergency Treatment in Time

	Freq.	%
0 No	190	96.94
3 Yes	6	3.06
Total	196	100.00

exv014_s4: 4. Unable to Receive Care and Attention

	Freq.	%
0 No	191	97.45
4 Yes	5	2.55
Total	196	100.00

exv014_s5: 5. Accident Caused by the Pandemic

	Freq.	%
0 Yes	196	100.00
Total	196	100.00

exv014_s6: 6. Other Reasons, Please Specify

	Freq.	%
0 No	194	98.98
6 Yes	2	1.02
Total	196	100.00

exv014_s7: 7. Not Related to the Pandemic

	Freq.	%
0 No	18	9.18
7 Yes	178	90.82
Total	196	100.00

ex005: Relationship to the Deceased?

	Freq.	%
1 Father	101	13.22
2 Mother	104	13.61
3 Spouse	279	36.52
4 Sibling	15	1.96
5 Other Relative	222	29.06
6 No Relation	43	5.63
Total	764	100.00

xezdisease_1_: Cancer

	Freq.	%
1 Yes	51	100.00
Total	51	100.00

xezdisease_2_: Chronic Lung Diseases

	Freq.	%
1 Yes	170	100.00
Total	170	100.00

xezdisease_3_: Heart Diseases

	Freq.	%
1 Yes	195	100.00
Total	195	100.00

xezdisease_4_: Stroke

	Freq.	%
1 Yes	122	100.00
Total	122	100.00

xezdisease_5_: Emotional Problems

	Freq.	%
1 Yes	38	100.00
Total	38	100.00

xechrodistype_1_: Disease[1]

A String Variable	
Obs:	762

xechrodistype_2_: Disease[2]

A String Variable	
Obs:	762

xechrodistype_3_: Disease[3]

A String Variable	
Obs:	762

xechrodistype_4_: Disease[4]

A String Variable	
Obs:	762

xechrodistype_5_: Disease[5]

A String Variable	
Obs:	762

xedisease_3_: Heart Diseases

	Freq.	%

	Freq.	%
1 Yes	281	36.88
2 No	481	63.12
Total	762	100.00

xedisease_1_: Cancer

	Freq.	%
1 Yes	189	24.80
2 No	573	75.20
Total	762	100.00

xedisease_4_: Stroke

	Freq.	%
1 Yes	158	20.73
2 No	604	79.27
Total	762	100.00

xehelperselect: Have Helper or Not

	Freq.	%
0 No	225	29.53
1 Yes	537	70.47
Total	762	100.00

xehelpernum: Number of Helpers

Mean	SD	Min	Max	Obs
3.54	3.62	1.00	26.00	537

xeselectnum: Numbers Selected

Mean	SD	Min	Max	Obs
7.00	0.00	7.00	7.00	68

xck_exf005: Value of exf005

Mean	SD	Min	Max	Obs
2,088.71	8,596.72	0.00	84,000.00	760

xck_exf007: Value of exf007

Mean	SD	Min	Max	Obs
4,804.60	22,597.84	0.00	400,000.00	760

xck_exf009: Value of exf009

Mean	SD	Min	Max	Obs
973.75	8,470.36	0.00	185,000.00	760

xck_exf011: Value of exf011

Mean	SD	Min	Max	Obs
583.82	6,463.40	0.00	150,000.00	760

xck_exf013: Value of exf013

Mean	SD	Min	Max	Obs
125.66	2,062.35	0.00	50,000.00	760

xck_exeb006: Value of exeb006

Mean	SD	Min	Max	Obs
1,375.24	16,505.33	0.00	400,000.00	760

xck_exg010: Value of exg010

Mean	SD	Min	Max	Obs
2,401.93	23,543.48	0.00	600,000.00	760

xck_exg012: Value of exg012

Mean	SD	Min	Max	Obs
8,085.43	42,826.44	0.00	600,000.00	760

xck_exg014: Value of exg014

Mean	SD	Min	Max	Obs
54.61	1,147.82	0.00	30,000.00	760

xck_exg016: Value of exg016

Mean	SD	Min	Max	Obs
1,741.51	10,432.73	0.00	150,000.00	760

xck_exg017: Value of exg017

Mean	SD	Min	Max	Obs
46.73	666.85	0.00	14,000.00	760

xexgtotalvalue: Total Value of Estate without Housing

Mean	SD	Min	Max	Obs
22,281.98	67,375.75	0.00	800,000.00	760

versionID: Version ID

A String Variable	
Obs:	770

This page intentionally left blank

MODULE AUX1

Sample Information

ID: Individual ID

A String Variable	
Obs:	20,180

householdID: Household ID

A String Variable	
Obs:	20,180

communityID: Community ID

A String Variable	
Obs:	20,180

died: Whether Individual Died

	Freq.	%
0 Alive	19,395	96.11
1 Died	785	3.89
Total	20,180	100.00

crosssection: Whether Cross-Section Sample

	Freq.	%
0 No	2,816	13.95
1 Yes	17,364	86.05
Total	20,180	100.00

iyear: Interview Year

A String Variable	
Obs:	20,180

imonth: Interview Month

A String Variable	
Obs:	20,180

versionID: Version ID

A String Variable	
Obs:	20,180

MODULE AUX2

Sampling Weights

ID: Individual ID

A String Variable	
Obs:	17,364

householdID: Household ID

A String Variable	
Obs:	17,364

communityID: Community ID

A String Variable	
Obs:	17,364

HH_weight: Household Base Weight

Mean	SD	Min	Max	Obs
28,854.24	39,583.01	4,532.62	1,206,623.84	17,364

HH_weight_ad1: Household Weight with Household Response Adjustment

Mean	SD	Min	Max	Obs
33,097.53	61,473.05	4,753.04	2,414,264.56	17,364

INDV_weight: Individual Base Weight

Mean	SD	Min	Max	Obs
32,371.69	35,234.57	7,370.20	1,178,908.25	17,364

INDV_weight_ad2: Individual Weight with Household and Individual Response Ad-

justment

Mean	SD	Min	Max	Obs
37,468.96	45,991.97	7,660.46	1,446,489.24	17,364

versionID: Version ID

A String Variable	
Obs:	17,364
