# Supplementary material for: Association of internet use and health service utilization with self-rated health in middle-aged and older adults: findings from a nationally representative longitudinal survey
Source: Front Public Health. 2024 Oct 3;12:1429983. doi: 10.3389/fpubh.2024.1429983 (PMC11483889; doi:10.3389/fpubh.2024.1429983)
Supplement: Supplementary file 6 [file Data_Sheet_6.PDF]

中国健康与养老追踪调查（CHARLS）

第五轮（2020）追访问卷

逻辑流程图

版本号: 20231106

2023 年 11 月

北京大学国家发展研究院
北京大学中国社会科学调查中心

目录

CV 过滤问卷 **1**
MAIN. 主逻辑 2
CV1. 双人或单人已访家户 3
CV2. 双人户婚姻及分户 4
CV3. 主要受访者婚姻 5

B 基本信息 **7**
MAIN. 主逻辑 8
B. 代理模式确认 9
BA. 基本信息 10

C 家庭信息 **11**
MAIN. 主逻辑 12
C. 代理模式确认 13
CA. 子女信息 14
CB. 家户成员信息 15
CC. 春节期间家庭联系 16

D 健康状况与功能 **17**
MAIN. 主逻辑 19
D. 代理模式确认 21
DA. 健康状况（一） 22
DA. 健康状况（二） 23
DA. 健康状况（三） 24
DB. 身体功能障碍以及辅助者（一） 25
DB. 身体功能障碍以及辅助者（二） 26
DB. 身体功能障碍以及辅助者（三） 27
DC. 认知和抑郁（一） 28
DC. 认知和抑郁（二） 29

F 工作与退休 **31**
MAIN. 主逻辑 32

F. 代理模式确认 33
FA. 工作概况（一） 34
FA. 工作概况（二） 35
FB. 农业自雇工作 36
FC. 受雇工作（一） 37
FC. 受雇工作（二） 38
FD. 非农自雇工作 39
FE. 非主要工作 40
FF. 求职与就业 41
FG. 疫情期间工作 42
FH. 退休手续 43

G 收入与支出 45

G1 家户收入与支出 46
MAIN. 主逻辑 46
G1. 代理模式确认 47
GB. 其他家户成员收入 48
GC. 家庭农业收入 49
GD. 个体经营和私营企业收入 50
GE. 家户公共转移支出收入 51
GF. 家户生活支出 52
I. 住房情况 53
G2 个人收入 54
MAIN. 主逻辑 54
G2. 代理模式确认 55
GA. 个人收入 56

V 疫情 57

MAIN. 主逻辑 58
V. 代理模式确认 59
VA. 疾病防范意识 60
VB. 个人患病和隔离 61
VC. 疫情期间个人活动 62
VD. 疫期居住地管控 63

EX 退出问卷 65

MAIN. 主逻辑 66
EXB. 基本信息 67
EXC. 家庭 68

EXD. 健康状况与功能（一） 69
EXD. 健康状况与功能（二） 70
EXD. 健康状况与功能（三） 71
EXE. 医疗保健与保险（一） 72
EXE. 医疗保健与保险（二） 73
EXF. 工作与退休 74
EXFN. 养老金 75
EXG. 收入、支出与资产 76
EXK. 殡葬 77
EXV. 疫情相关 78
VA. 死因分析 79

This page intentionally left blank

CV　过滤问卷

MAIN. 主逻辑

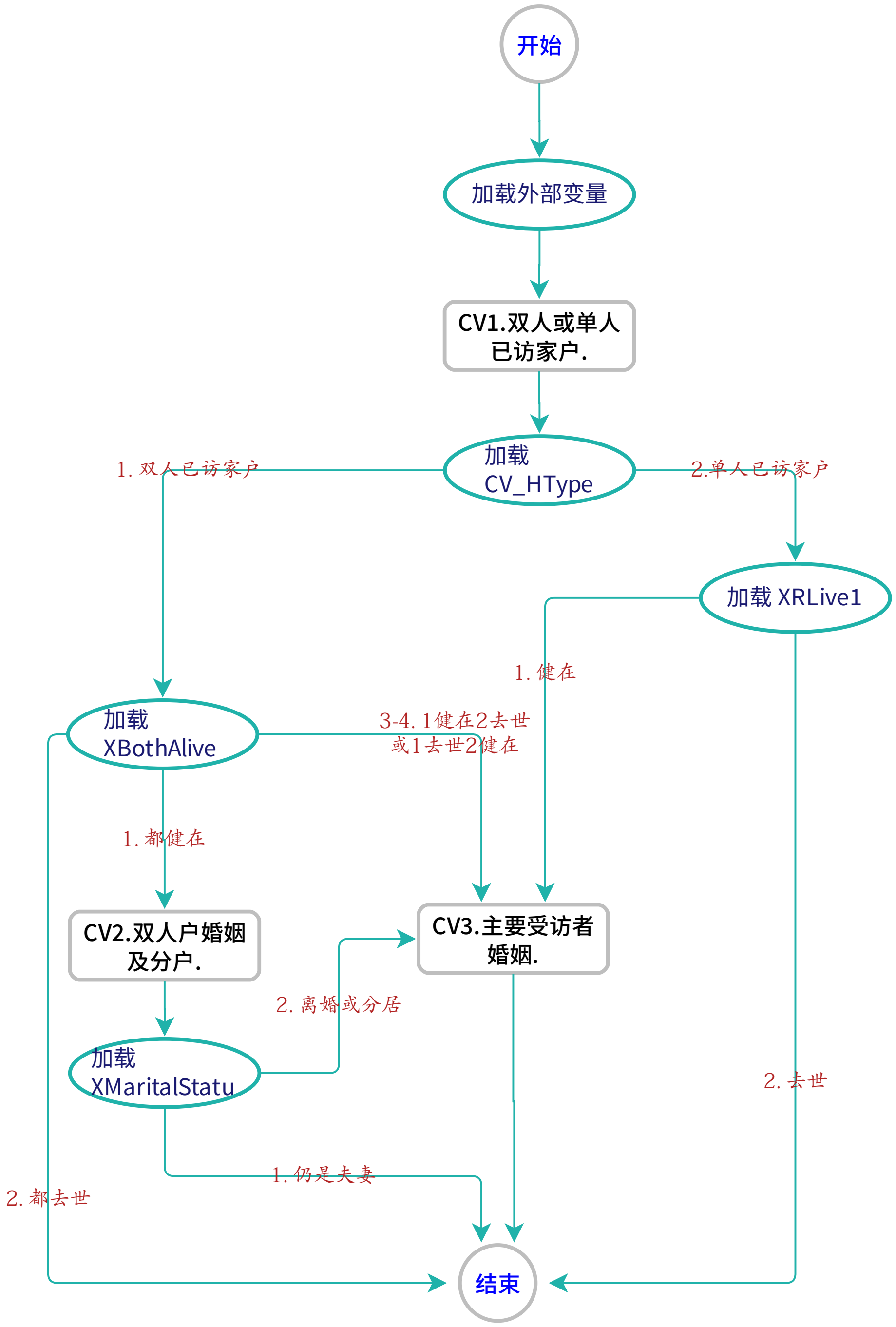
开始
加载外部变量
CV1.双人或单人已访家户.
加载 CV_HType
1. 双人已访家户
2.单人已访家户
加载 XRLive1
1. 健在
2. 去世
加载 XBothAlive
3-4. 1健在2去世或1去世2健在
1. 都健在
2. 都去世
CV2.双人户婚姻及分户.
CV3.主要受访者婚姻.
加载 XMaritalStatu
2. 离婚或分居
1. 仍是夫妻
结束

CV1. 双人或单人已访家户

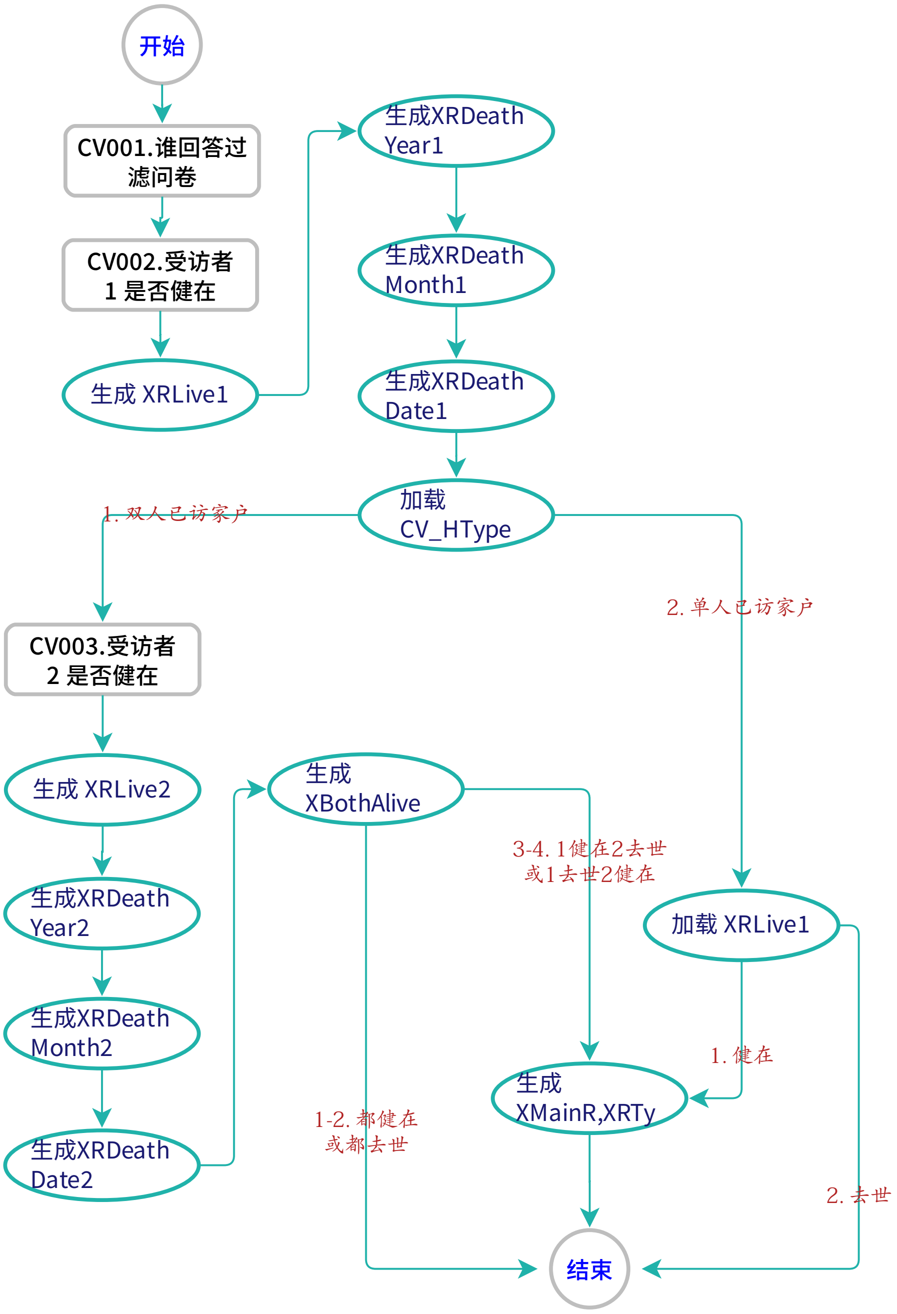

CV2. 双人户婚姻及分户

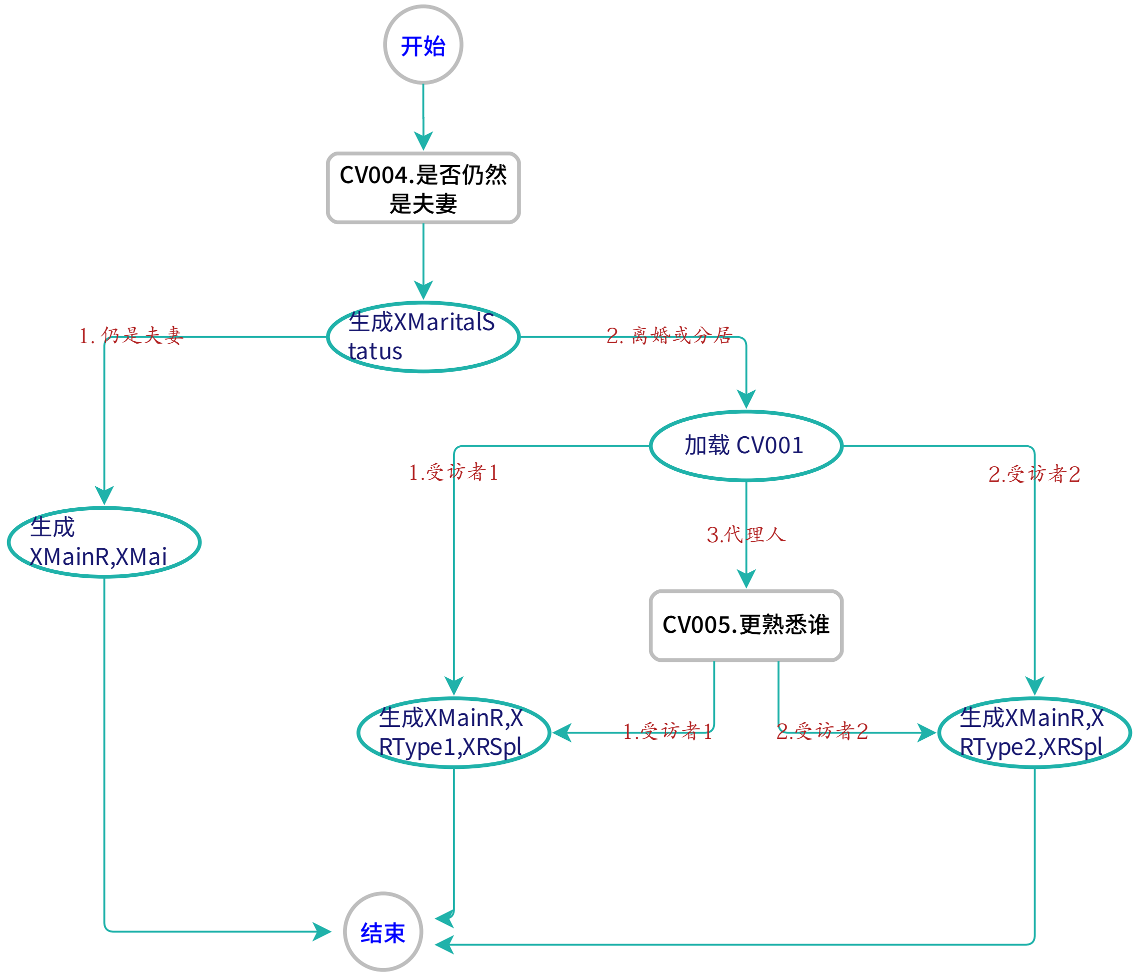

CV3. 主要受访者婚姻

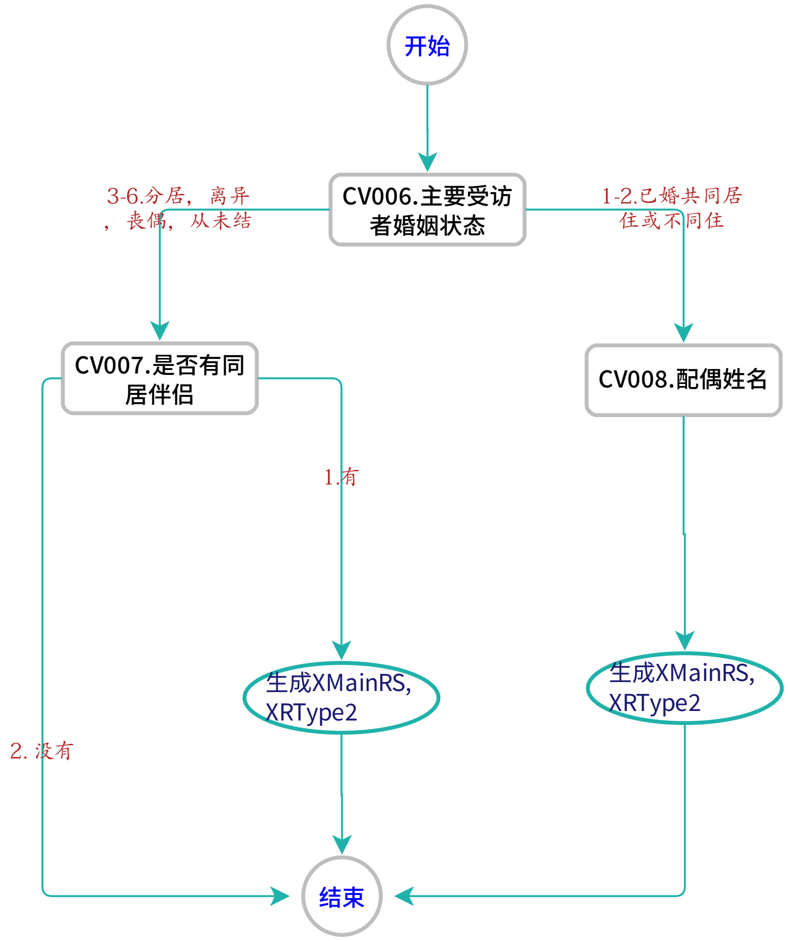
开始
CV006.主要受访者婚姻状态
3-6.分居，离异，丧偶，从未结
1-2.已婚共同居住或不同住
CV007.是否有同居伴侣
CV008.配偶姓名
1.有
2. 没有
生成XMainRS, XRType2
生成XMainRS, XRType2
结束

This page intentionally left blank

B　基本信息

MAIN. 主逻辑

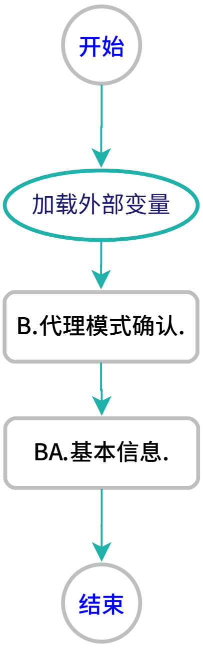

B. 代理模式确认

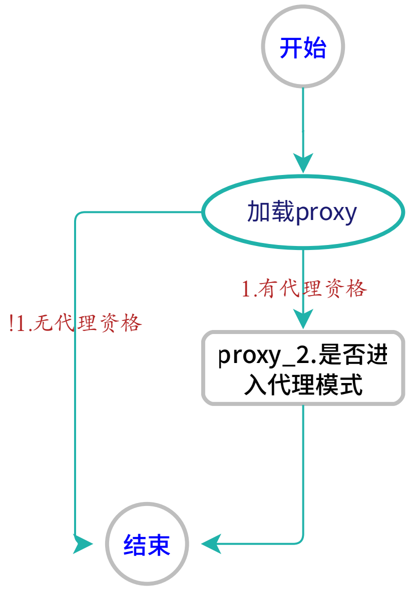

BA. 基本信息

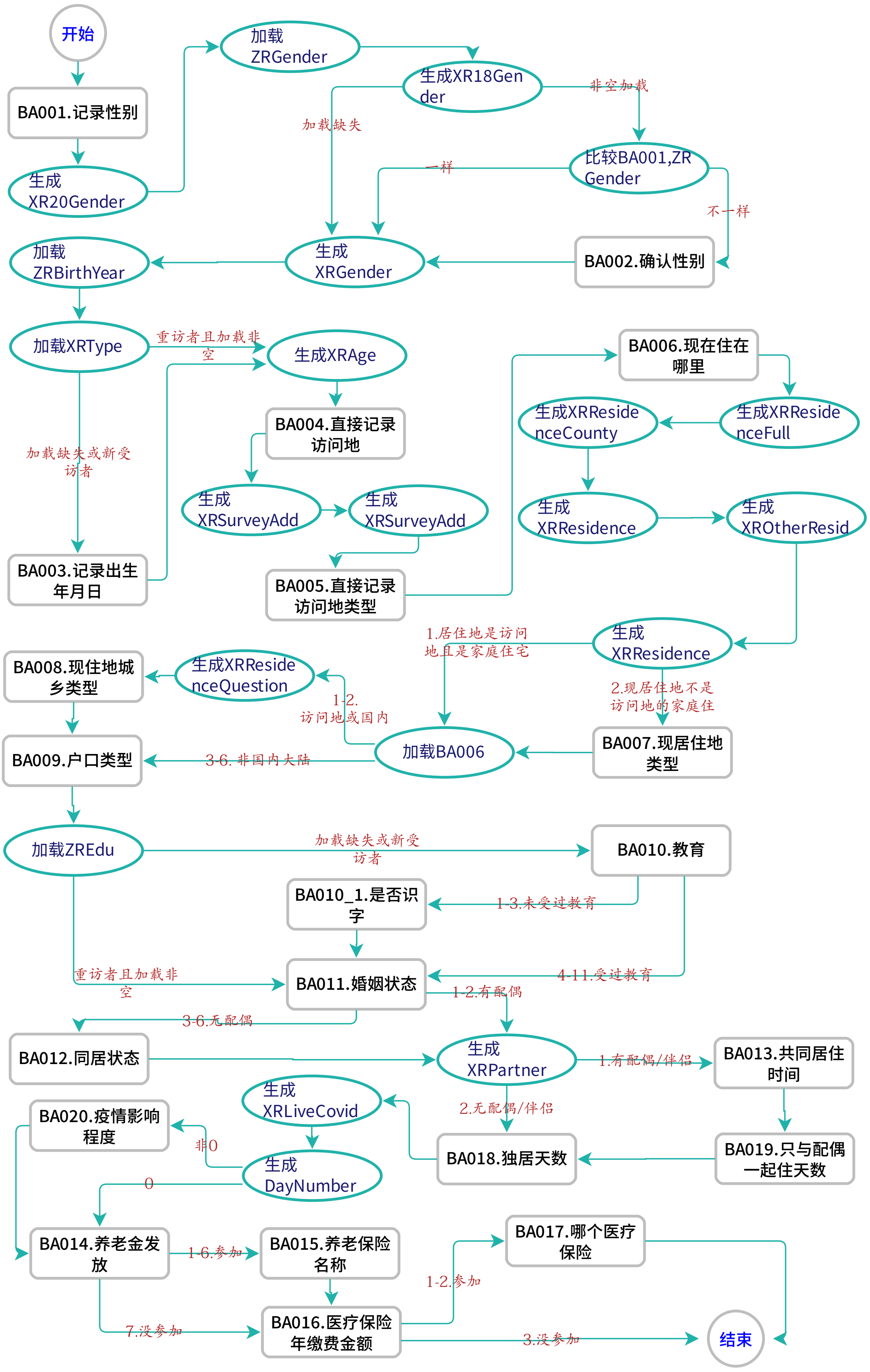
开始
BA001.记录性别
生成XR20Gender
加载ZRGender
生成XR18Gender
非空加载
加载缺失
比较BA001,ZRGender
一样
不一样
BA002.确认性别
生成XRGender
加载ZRBirthYear
加载XRType
重访者且加载非空
生成XRAge
BA004.直接记录访问地
加载缺失或新受访者
生成XRSurveyAdd
生成XRSurveyAdd
BA003.记录出生年月日
BA005.直接记录访问地类型
BA006.现在住在哪里
生成XRResidenceFull
生成XRResidenceCounty
生成XRResidence
生成XROtherResid
生成XRResidence
1.居住地是访问地且是家庭住宅
2.现居住地不是访问地的家庭住
BA008.现住地城乡类型
生成XRResidenceQuestion
1-2.访问地或国内
加载BA006
BA007.现居住地类型
BA009.户口类型
3-6.非国内大陆
加载ZREdu
加载缺失或新受访者
BA010.教育
BA010_1.是否识字
1-3.未受过教育
重访者且加载非空
BA011.婚姻状态
4-11.受过教育
1-2.有配偶
3-6.无配偶
BA012.同居状态
生成XRPartner
1.有配偶/伴侣
BA013.共同居住时间
2.无配偶/伴侣
生成XRLiveCovid
BA020.疫情影响程度
非0
生成DayNumber
0
BA018.独居天数
BA019.只与配偶一起住天数
BA014.养老金发放
1-6.参加
BA015.养老保险名称
BA017.哪个医疗保险
1-2.参加
BA016.医疗保险年缴费金额
7.没参加
3.没参加
结束

C　家庭信息

MAIN. 主逻辑

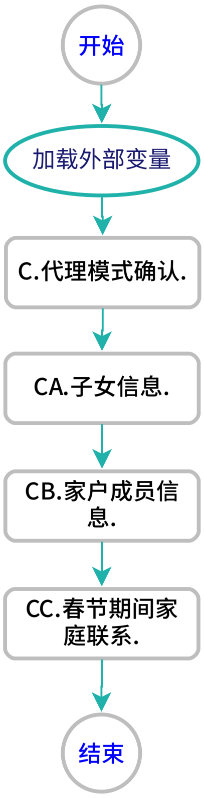

C. 代理模式确认

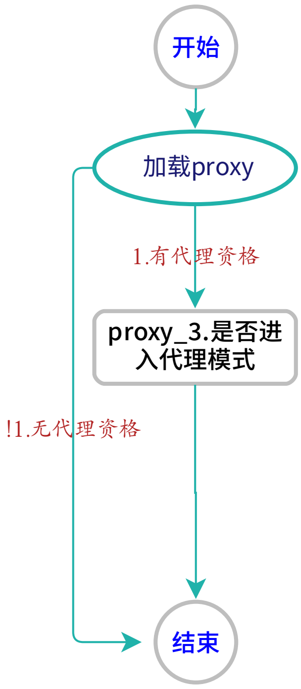

CA. 子女信息

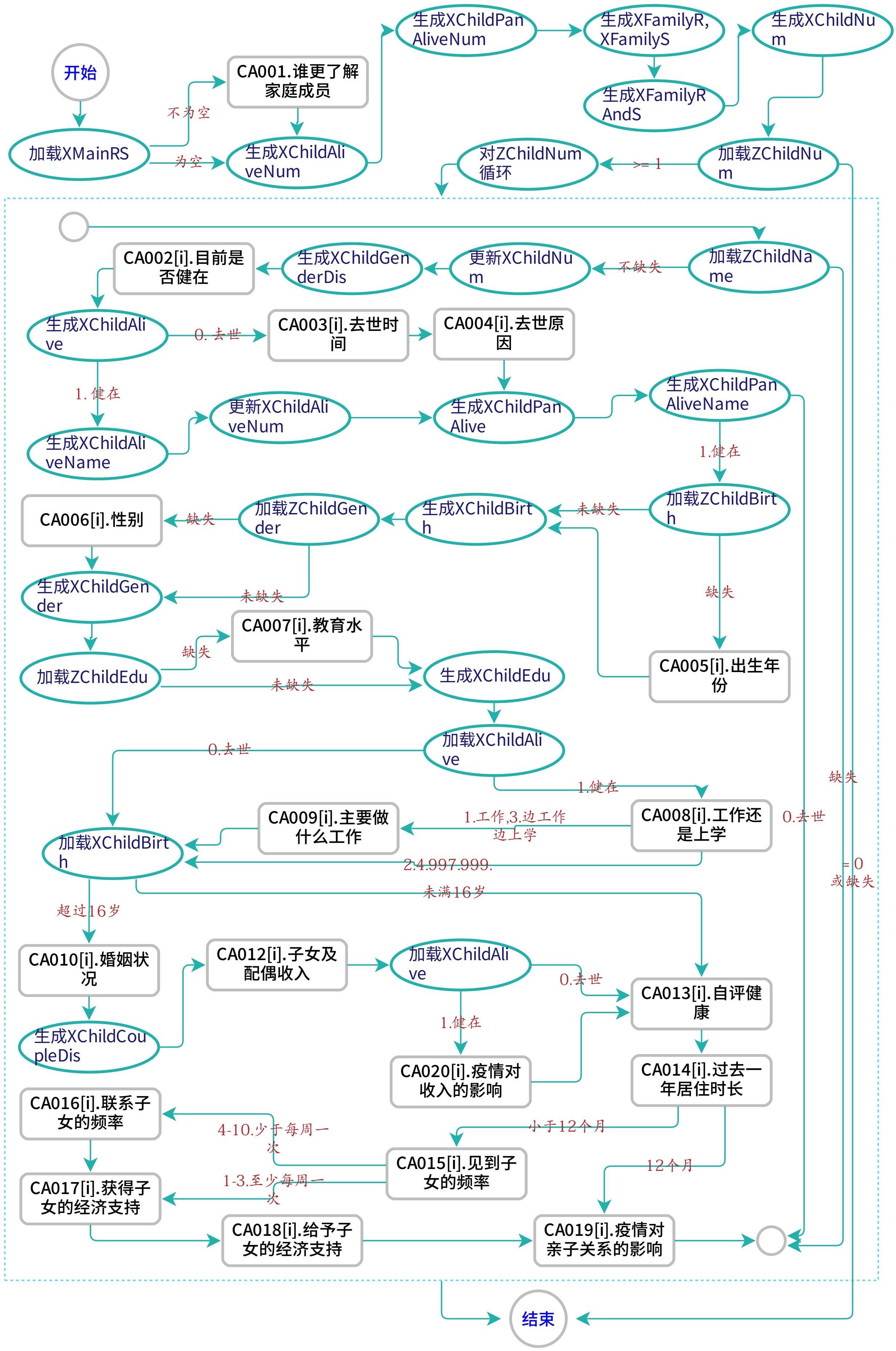
开始
加载XMainRS
不为空
为空
CA001.谁更了解家庭成员
生成XChildAliveNum
生成XChildPanAliveNum
生成XFamilyR, XFamilyS
生成XFamilyRAndS
生成XChildNum
加载ZChildNum
>= 1
对ZChildNum循环
加载ZChildName
不缺失
更新XChildNum
生成XChildGenderDis
CA002[i].目前是否健在
生成XChildAlive
0. 去世
CA003[i].去世时间
CA004[i].去世原因
1. 健在
生成XChildAliveName
更新XChildAliveNum
生成XChildPanAlive
生成XChildPanAliveName
1.健在
加载ZChildBirth
未缺失
生成XChildBirth
缺失
CA005[i].出生年份
加载ZChildGender
CA006[i].性别
缺失
生成XChildGender
未缺失
加载ZChildEdu
缺失
CA007[i].教育水平
未缺失
生成XChildEdu
加载XChildAlive
0.去世
1.健在
CA008[i].工作还是上学
1.工作,3.边工作边上学
CA009[i].主要做什么工作
2,4,997,999.
加载XChildBirth
未满16岁
超过16岁
CA010[i].婚姻状况
生成XChildCoupleDis
CA012[i].子女及配偶收入
加载XChildAlive
0.去世
1.健在
CA020[i].疫情对收入的影响
CA013[i].自评健康
CA014[i].过去一年居住时长
小于12个月
12个月
CA015[i].见到子女的频率
4-10.少于每周一次
1-3.至少每周一次
CA016[i].联系子女的频率
CA017[i].获得子女的经济支持
CA018[i].给予子女的经济支持
CA019[i].疫情对亲子关系的影响
缺失
0.去世
=0
或缺失
结束

CB. 家户成员信息

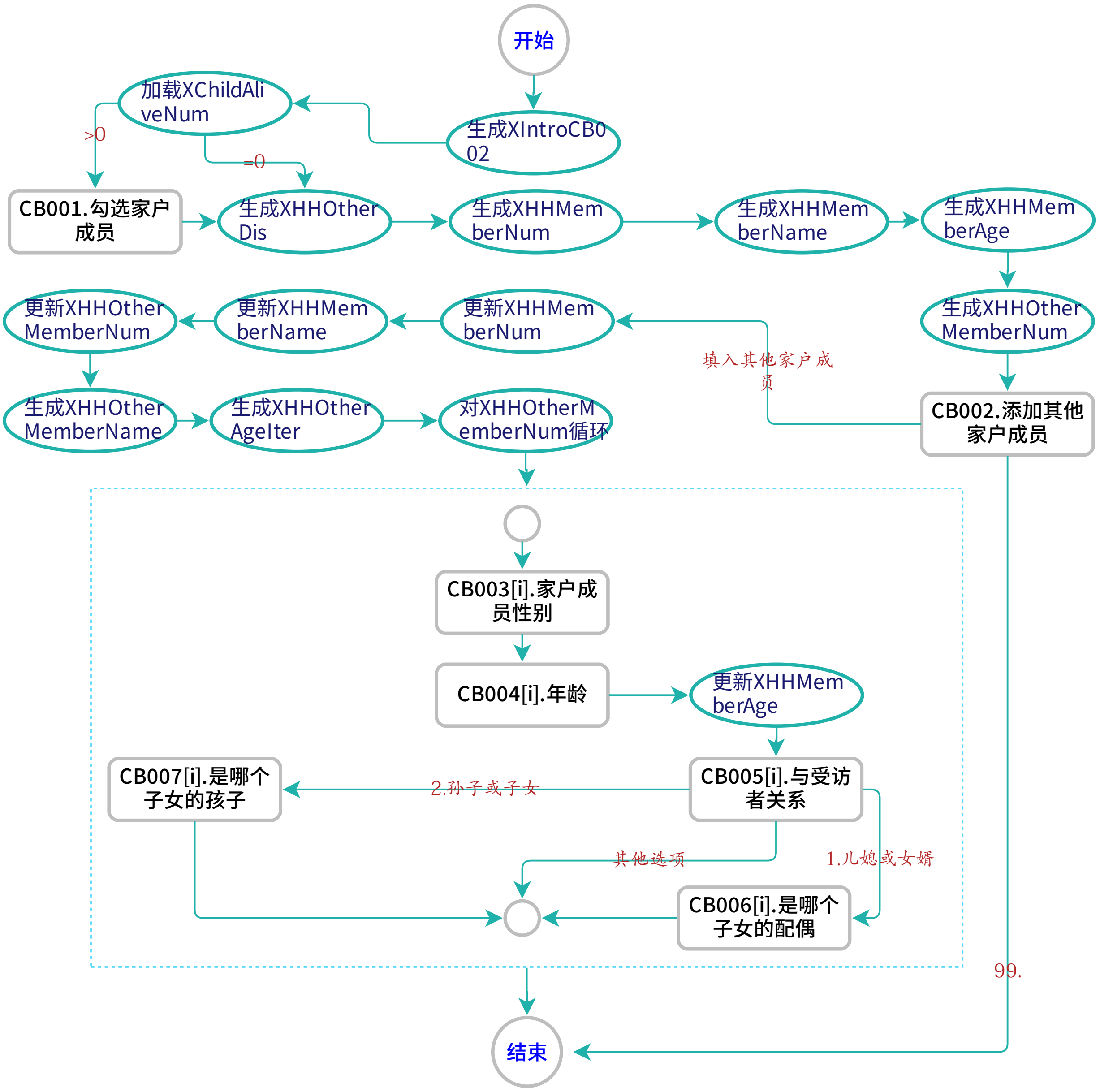
开始
生成XIntroCB002
加载XChildAliveNum
>0
=0
CB001.勾选家户成员
生成XHHOtherDis
生成XHHMemberNum
生成XHHMemberName
生成XHHMemberAge
生成XHHOtherMemberNum
CB002.添加其他家户成员
填入其他家户成员
更新XHHMemberNum
更新XHHMemberName
更新XHHOtherMemberNum
生成XHHOtherMemberName
生成XHHOtherAgeIter
对XHHOtherMemberNum循环
CB003[i].家户成员性别
CB004[i].年龄
更新XHHMemberAge
CB005[i].与受访者关系
2.孙子或子女
CB007[i].是哪个子女的孩子
其他选项
1.儿媳或女婿
CB006[i].是哪个子女的配偶
99.
结束

CC. 春节期间家庭联系

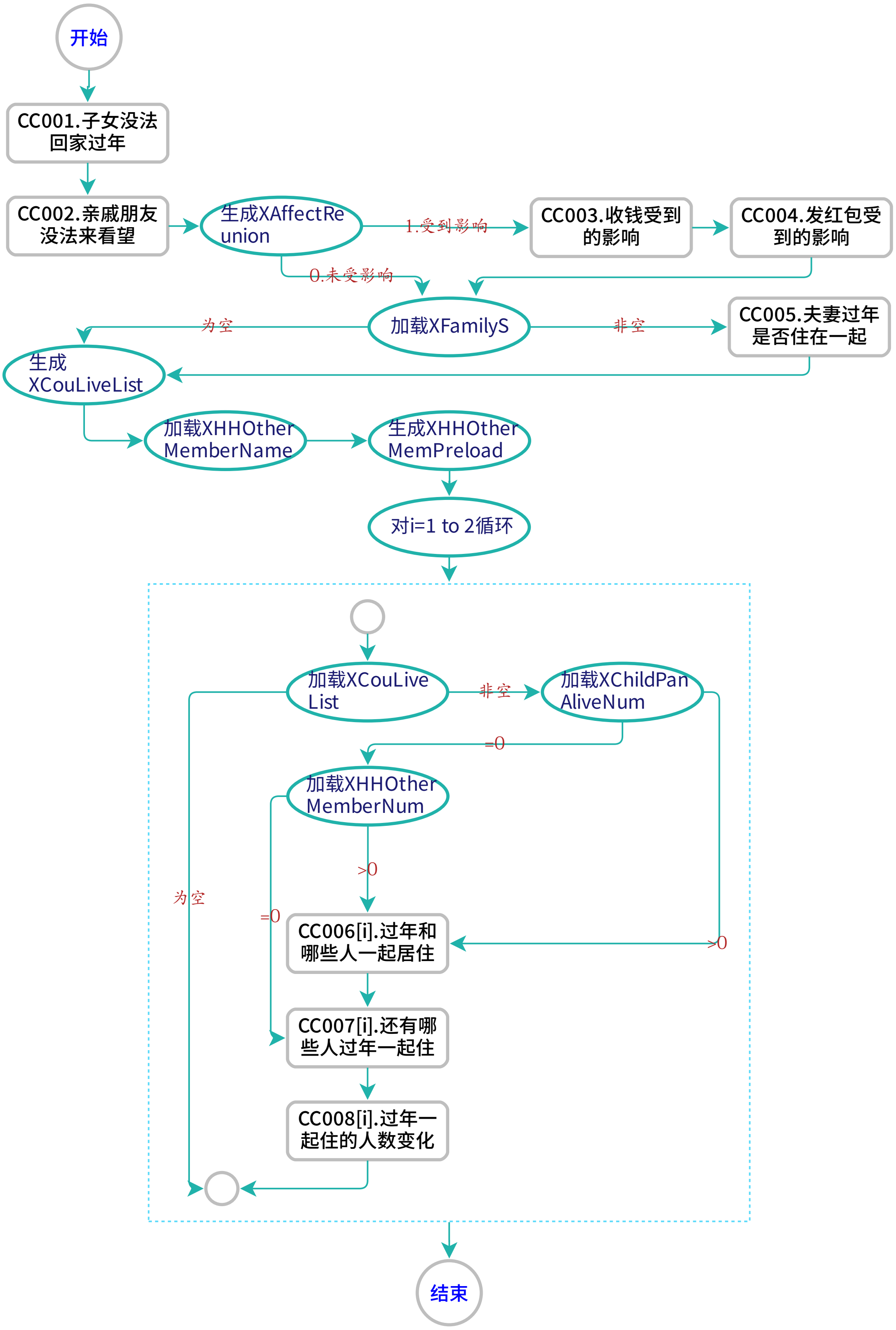
开始
CC001.子女没法回家过年
CC002.亲戚朋友没法来看望
生成XAffectReunion
1.受到影响
0.未受影响
CC003.收钱受到的影响
CC004.发红包受到的影响
加载XFamilyS
为空
非空
CC005.夫妻过年是否住在一起
生成XCouLiveList
加载XHHOtherMemberName
生成XHHOtherMemPreload
对i=1 to 2循环
加载XCouLiveList
非空
加载XChildPanAliveNum
=0
加载XHHOtherMemberNum
>0
为空
=0
CC006[i].过年和哪些人一起居住
>0
CC007[i].还有哪些人过年一起住
CC008[i].过年一起住的人数变化
结束

D　健康状况与功能

MAIN. 主逻辑

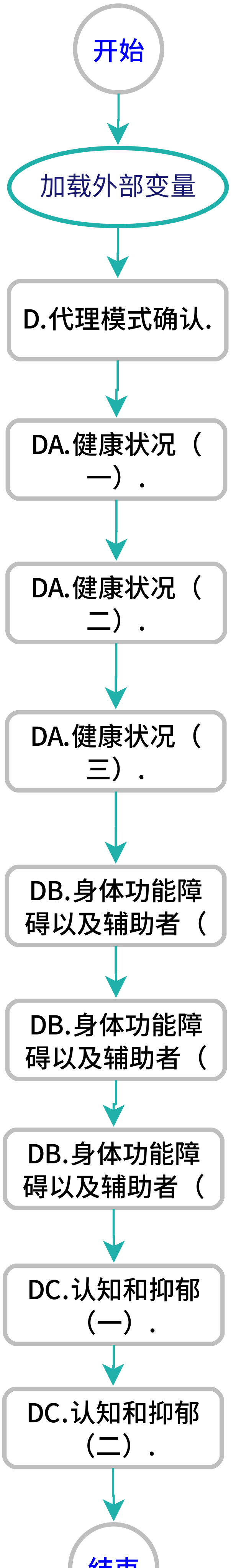
开始
加载外部变量
D.代理模式确认.
DA.健康状况（一）.
DA.健康状况（二）.
DA.健康状况（三）.
DB.身体功能障碍以及辅助者（
DB.身体功能障碍以及辅助者（
DB.身体功能障碍以及辅助者（
DC.认知和抑郁（一）.
DC.认知和抑郁（二）.

D. 代理模式确认

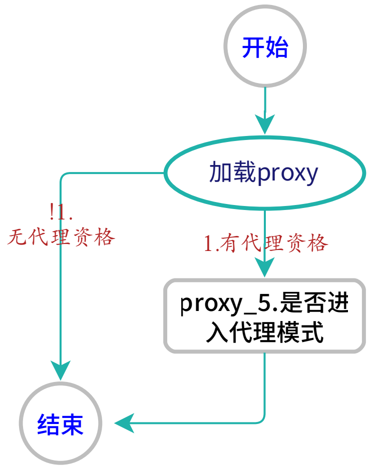

DA. 健康状况（一）

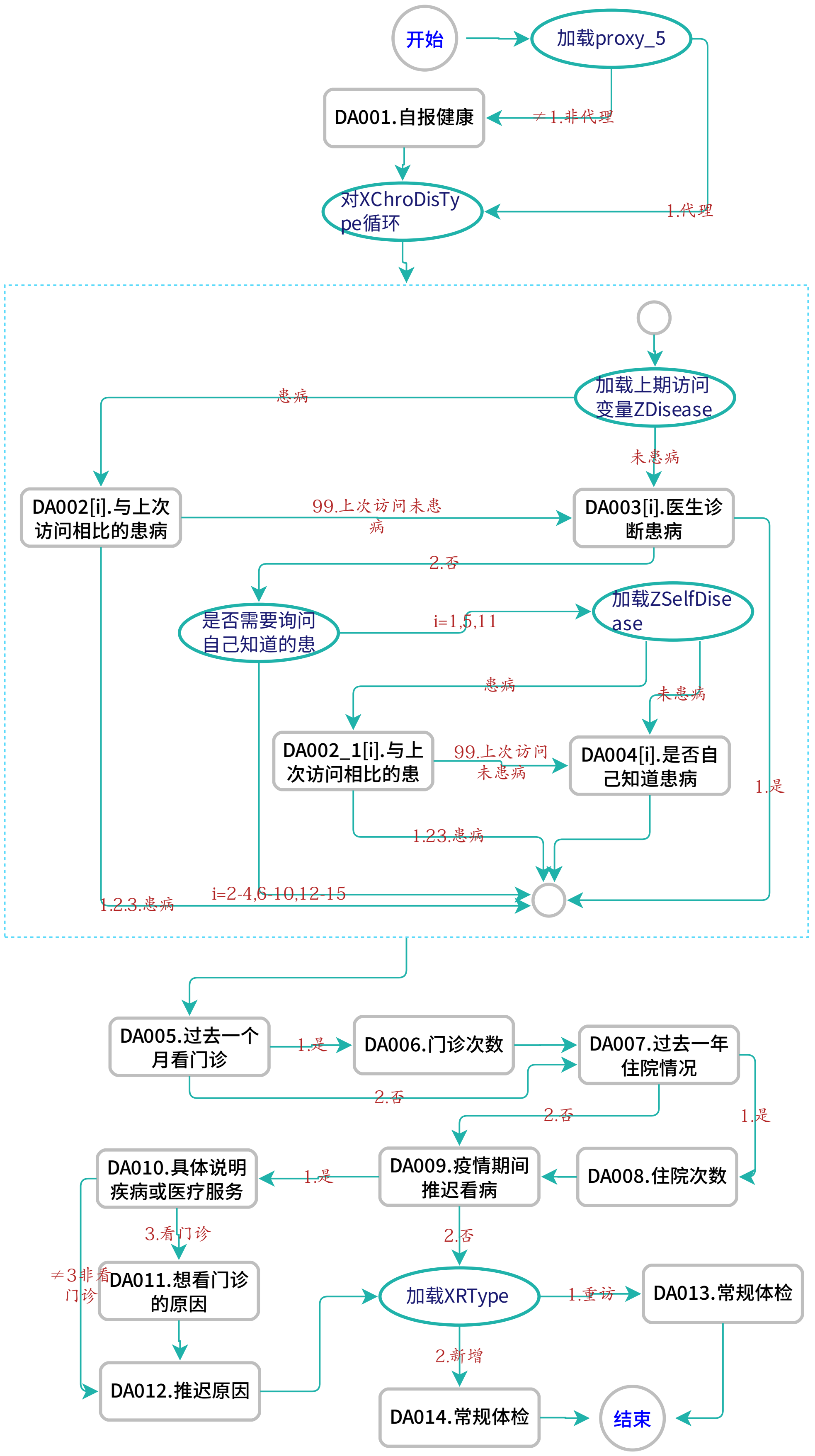
开始
加载proxy_5
≠1.非代理
1.代理
DA001.自报健康
对XChroDisType循环
加载上期访问变量ZDisease
患病
未患病
DA002[i].与上次访问相比的患病
99.上次访问未患病
DA003[i].医生诊断患病
2.否
是否需要询问自己知道的患
i=1,5,11
加载ZSelfDisease
患病
未患病
DA002_1[i].与上次访问相比的患
99.上次访问未患病
DA004[i].是否自己知道患病
1.是
1.23.患病
1.2.3.患病
i=2-4,6-10,12-15
DA005.过去一个月看门诊
1.是
DA006.门诊次数
DA007.过去一年住院情况
2.否
2.否
1.是
DA010.具体说明疾病或医疗服务
1.是
DA009.疫情期间推迟看病
DA008.住院次数
3.看门诊
2.否
≠3非看门诊
DA011.想看门诊的原因
加载XRType
1.重访
DA013.常规体检
2.新增
DA012.推迟原因
DA014.常规体检
结束

DA. 健康状况（二）

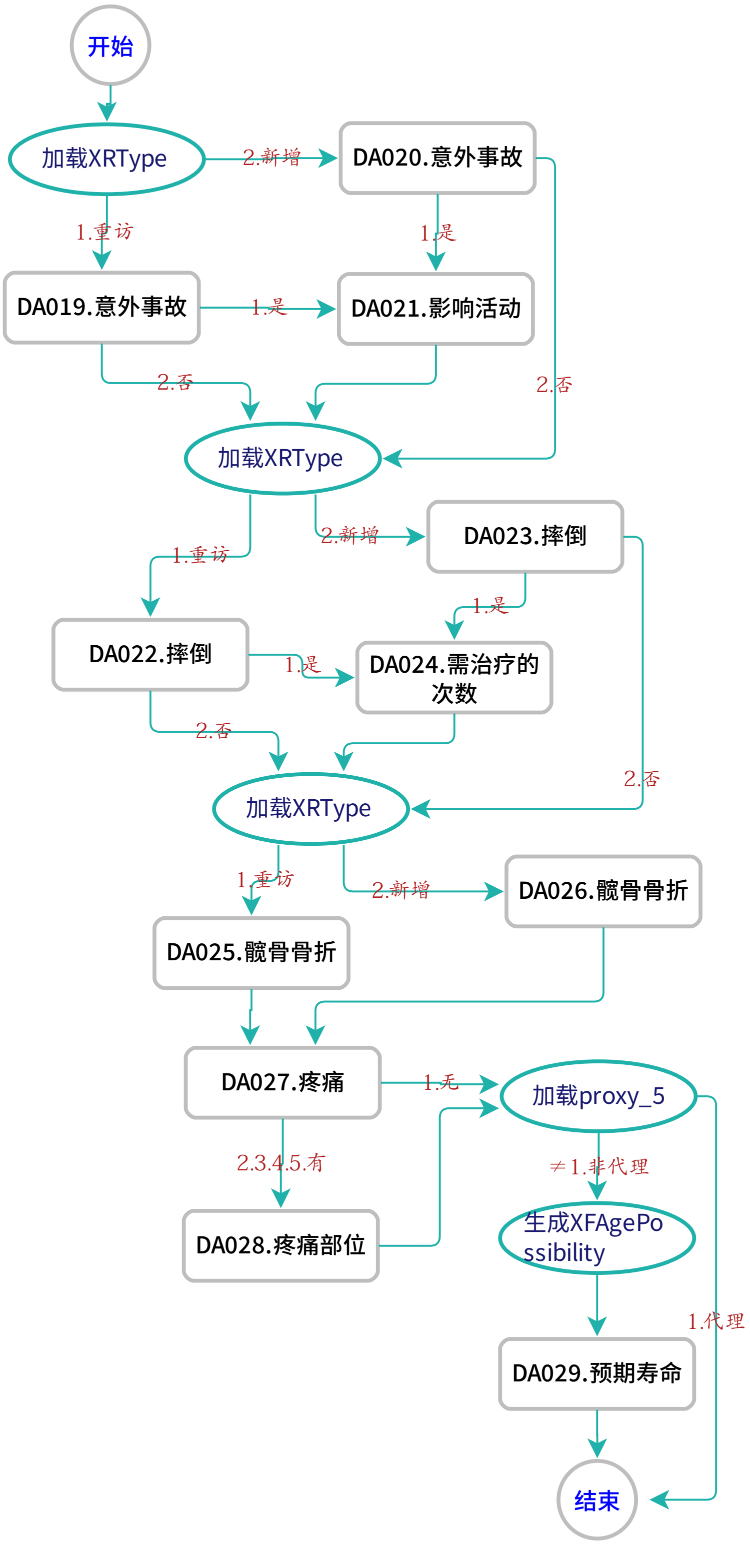
开始
加载XRType
2.新增
DA020.意外事故
1.重访
1.是
DA019.意外事故
1.是
DA021.影响活动
2.否
2.否
加载XRType
2.新增
DA023.摔倒
1.重访
1.是
DA022.摔倒
1.是
DA024.需治疗的次数
2.否
2.否
加载XRType
1.重访
2.新增
DA026.髋骨骨折
DA025.髋骨骨折
DA027.疼痛
1.无
加载proxy_5
2.3.4.5.有
≠1.非代理
DA028.疼痛部位
生成XFAgePossibility
1.代理
DA029.预期寿命
结束

DA. 健康状况（三）

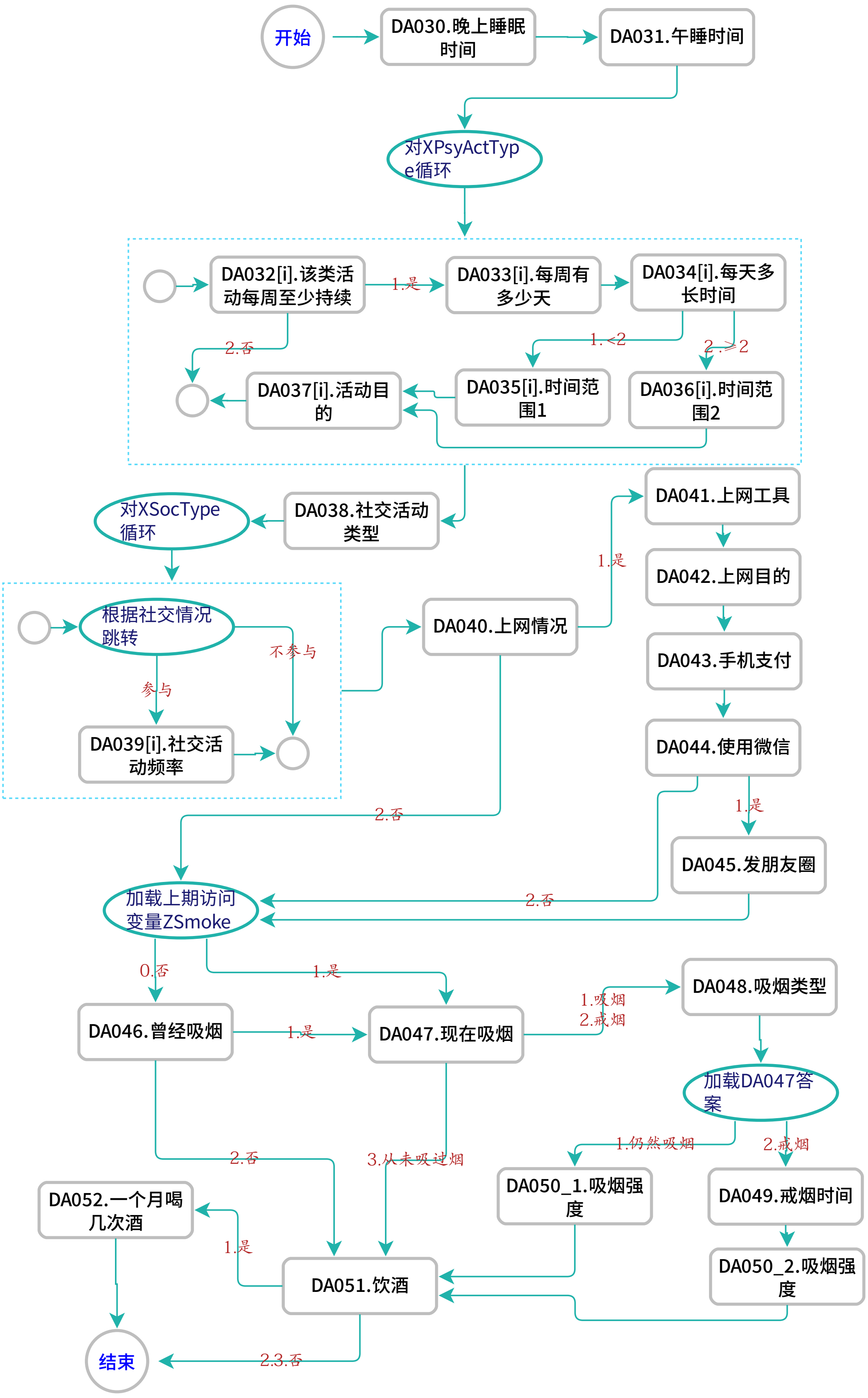
开始
DA030.晚上睡眠时间
DA031.午睡时间
对XPsyActType循环
DA032[i].该类活动每周至少持续
1.是
DA033[i].每周有多少天
DA034[i].每天多长时间
2.否
1.<2
2.>2
DA035[i].时间范围1
DA036[i].时间范围2
DA037[i].活动目的
DA038.社交活动类型
对XSocType循环
根据社交情况跳转
不参与
参与
DA039[i].社交活动频率
DA040.上网情况
1.是
DA041.上网工具
DA042.上网目的
DA043.手机支付
DA044.使用微信
1.是
DA045.发朋友圈
2.否
2.否
加载上期访问变量ZSmoke
0.否
1.是
DA046.曾经吸烟
1.是
DA047.现在吸烟
1.吸烟
2.戒烟
DA048.吸烟类型
加载DA047答案
1.仍然吸烟
2.戒烟
DA050_1.吸烟强度
DA049.戒烟时间
DA050_2.吸烟强度
2.否
3.从未吸过烟
DA052.一个月喝几次酒
1.是
DA051.饮酒
结束
2.3.否

DB. 身体功能障碍以及辅助者（一）

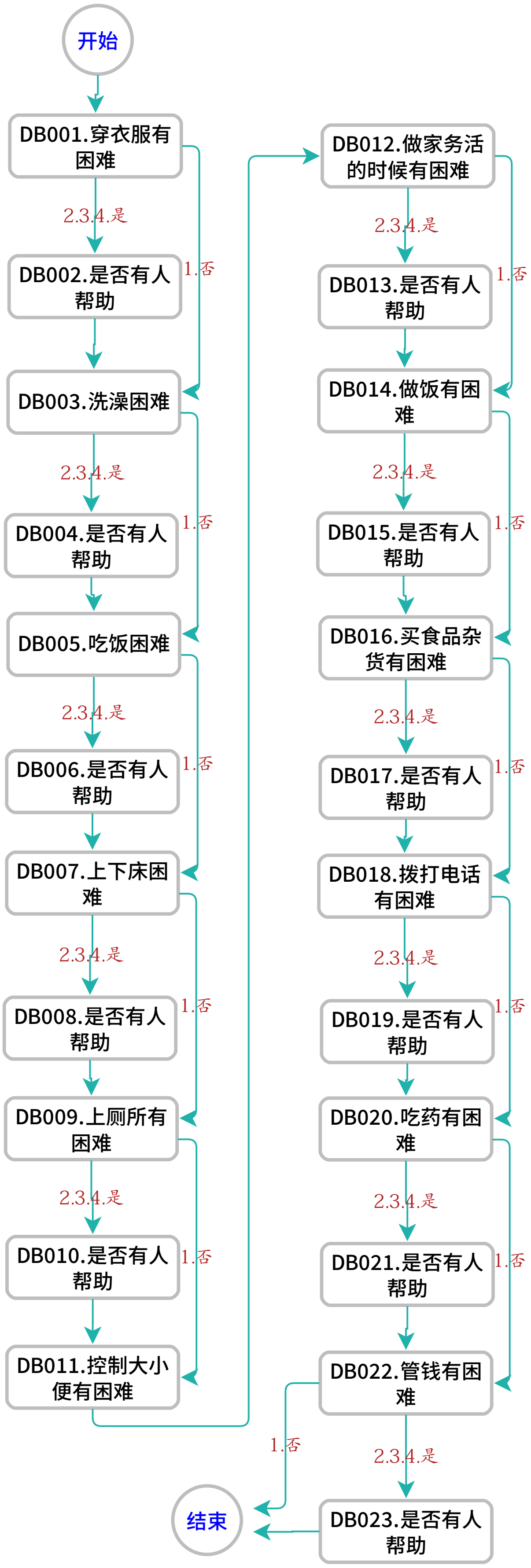

DB. 身体功能障碍以及辅助者（二）

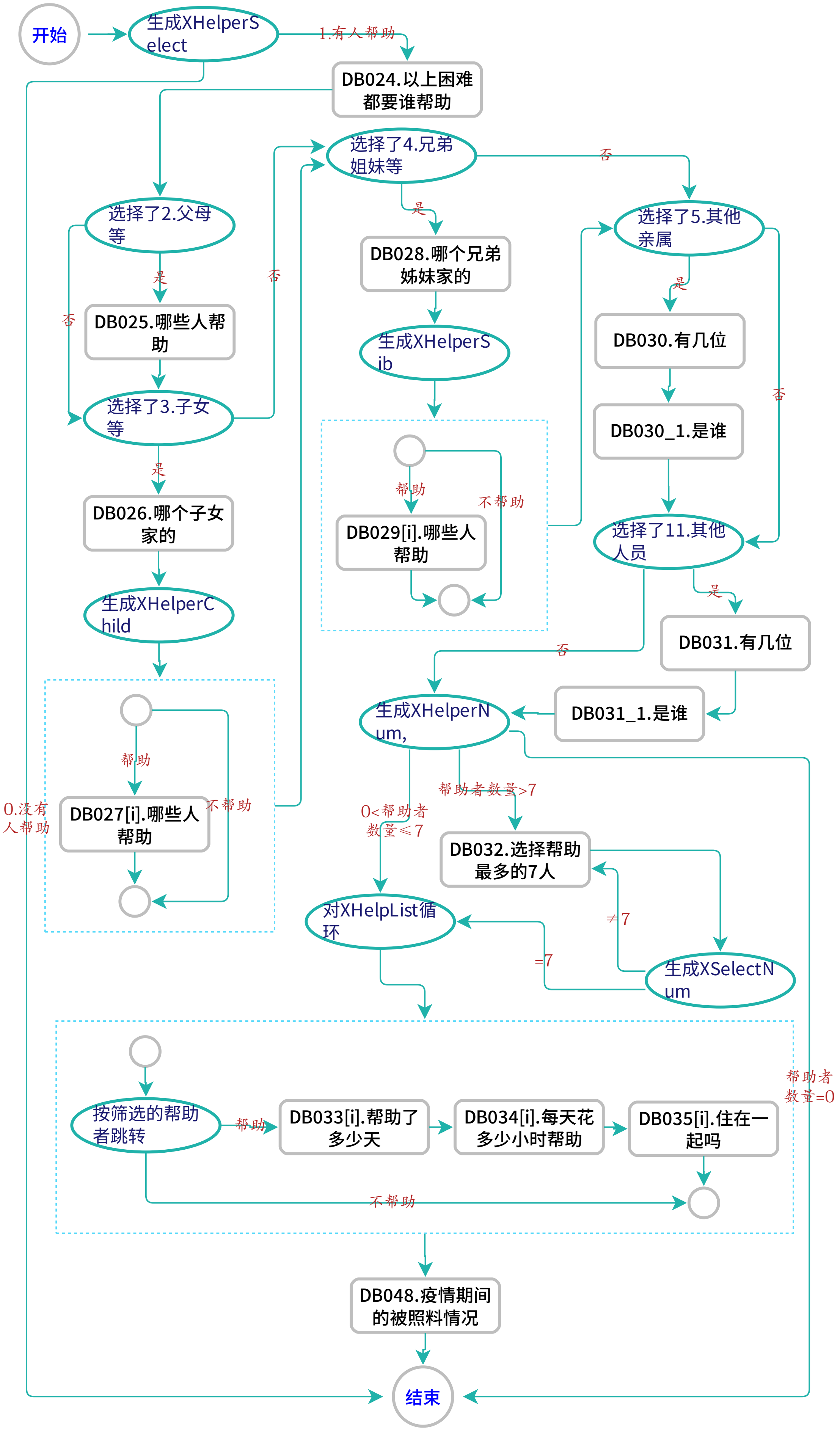

DB. 身体功能障碍以及辅助者（三）

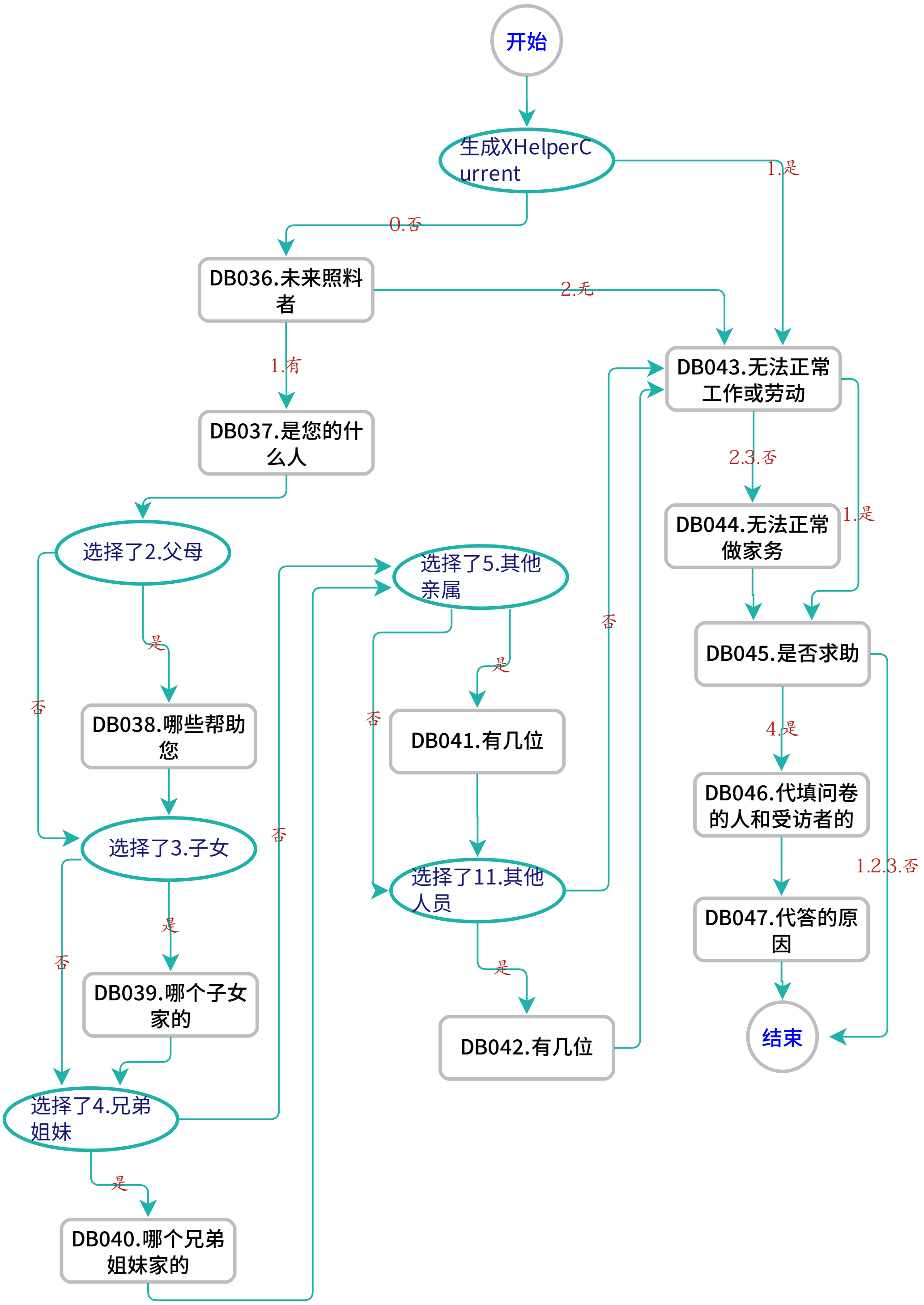

DC. 认知和抑郁（一）

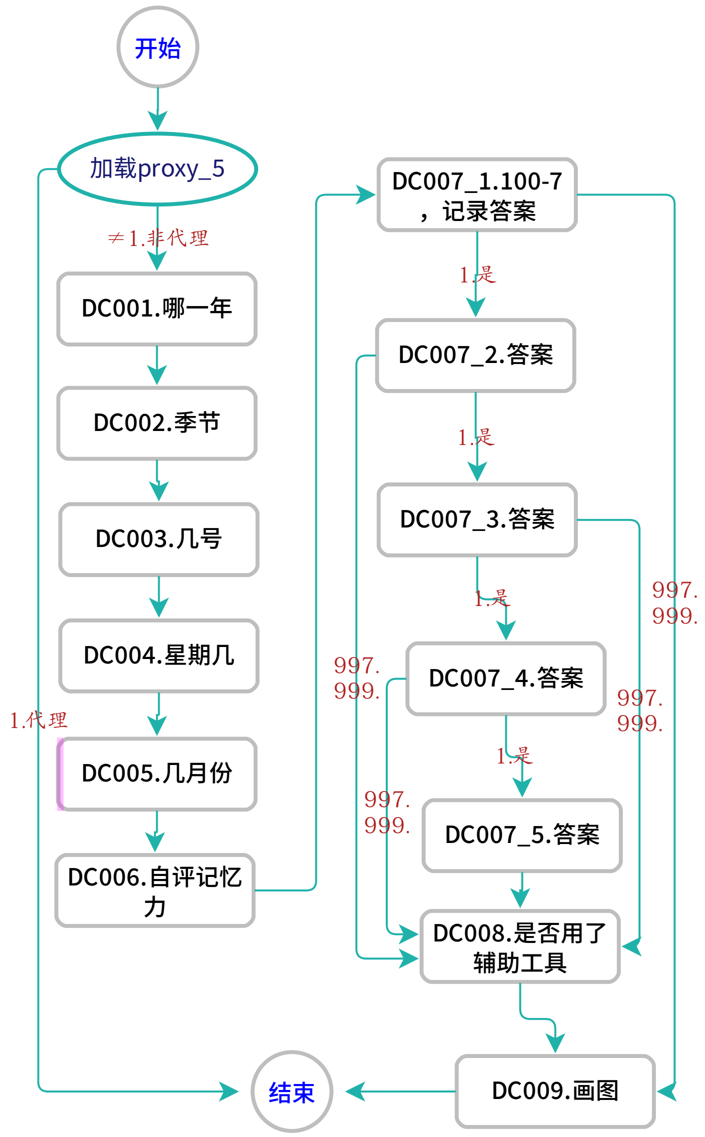
开始
加载proxy_5
≠1.非代理
DC001.哪一年
DC002.季节
DC003.几号
DC004.星期几
1.代理
DC005.几月份
DC006.自评记忆力
DC007_1.100-7，记录答案
1.是
DC007_2.答案
1.是
DC007_3.答案
1.是
DC007_4.答案
1.是
DC007_5.答案
997.
999.
997.
999.
997.
999.
997.
999.
DC008.是否用了辅助工具
DC009.画图
结束

DC. 认知和抑郁（二）

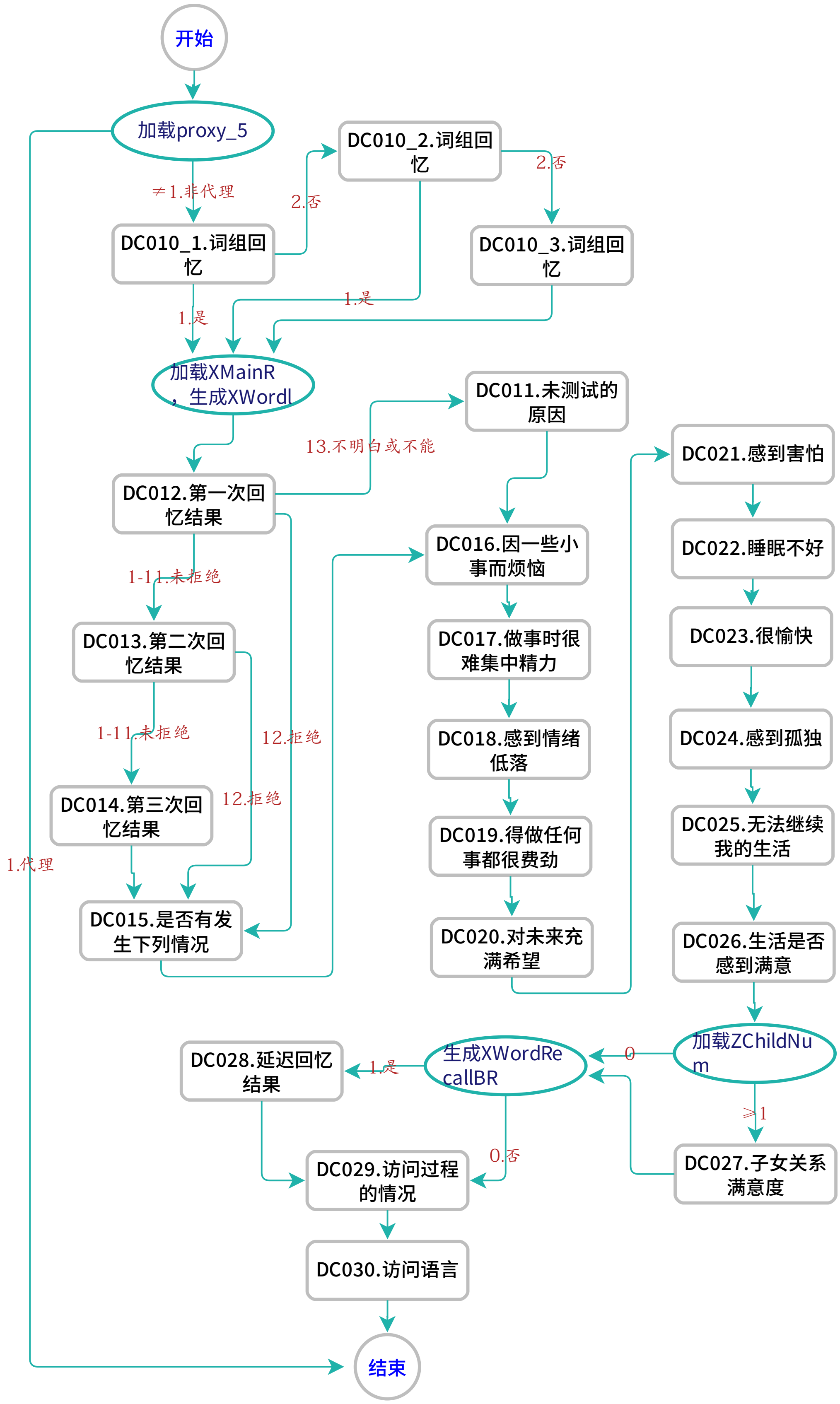

开始
加载proxy_5
≠1.非代理
1.代理
DC010_1.词组回忆
2.否
DC010_2.词组回忆
2.否
DC010_3.词组回忆
1.是
1.是
加载XMainR，生成XWordl
DC011.未测试的原因
13.不明白或不能
DC012.第一次回忆结果
1-11.未拒绝
12.拒绝
DC013.第二次回忆结果
1-11.未拒绝
12.拒绝
DC014.第三次回忆结果
DC015.是否有发生下列情况
DC016.因一些小事而烦恼
DC017.做事时很难集中精力
DC018.感到情绪低落
DC019.得做任何事都很费劲
DC020.对未来充满希望
DC021.感到害怕
DC022.睡眠不好
DC023.很愉快
DC024.感到孤独
DC025.无法继续我的生活
DC026.生活是否感到满意
加载ZChildNum
0
≥1
DC027.子女关系满意度
生成XWordRecallBR
1.是
0.否
DC028.延迟回忆结果
DC029.访问过程的情况
DC030.访问语言
结束

F　工作与退休

MAIN. 主逻辑

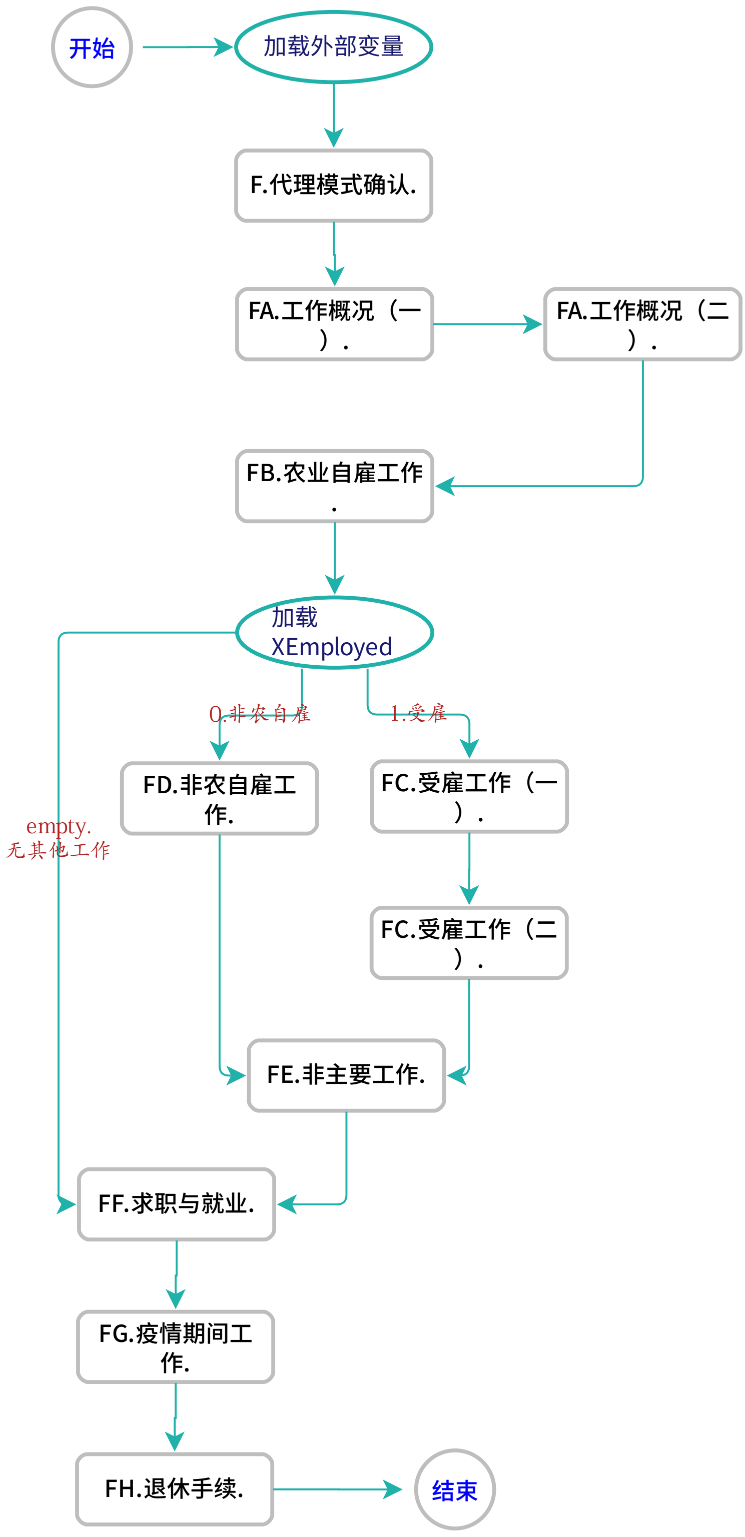
开始
加载外部变量
F.代理模式确认.
FA.工作概况（一）.
FA.工作概况（二）.
FB.农业自雇工作.
加载 XEmployed
0.非农自雇
1.受雇
empty.
无其他工作
FD.非农自雇工作.
FC.受雇工作（一）.
FC.受雇工作（二）.
FE.非主要工作.
FF.求职与就业.
FG.疫情期间工作.
FH.退休手续.
结束

F. 代理模式确认

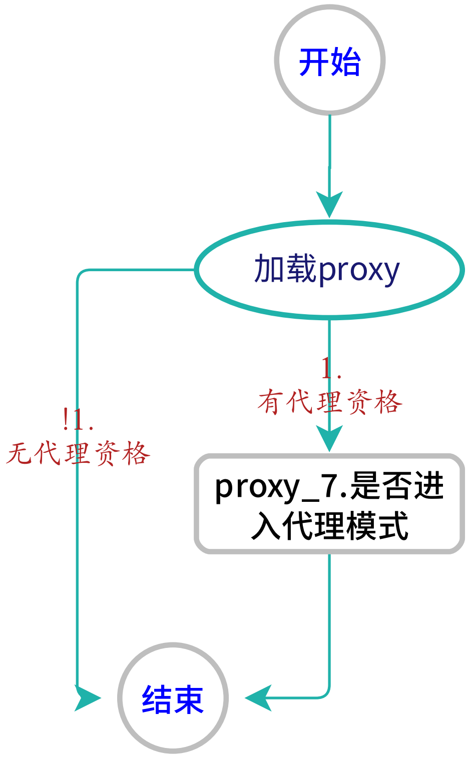

FA. 工作概况（一）

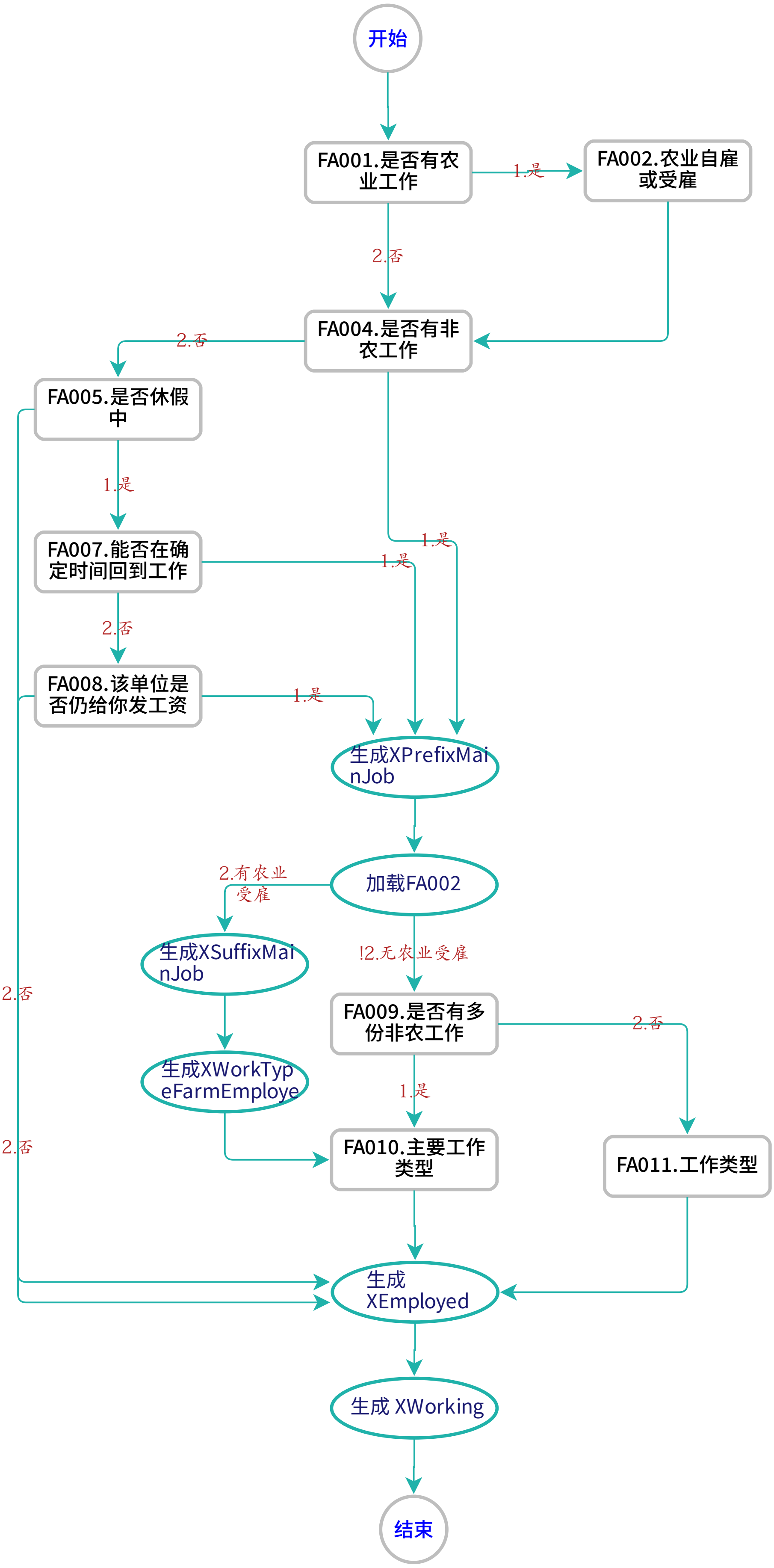
开始
FA001.是否有农业工作
1.是
FA002.农业自雇或受雇
2.否
FA004.是否有非农工作
2.否
FA005.是否休假中
1.是
FA007.能否在确定时间回到工作
1.是
1.是
2.否
FA008.该单位是否仍给你发工资
1.是
生成XPrefixMainJob
加载FA002
2.有农业受雇
生成XSuffixMainJob
!2.无农业受雇
2.否
生成XWorkTypeFarmEmploye
FA009.是否有多份非农工作
2.否
1.是
FA010.主要工作类型
FA011.工作类型
2.否
生成XEmployed
生成 XWorking
结束

FA. 工作概况（二）

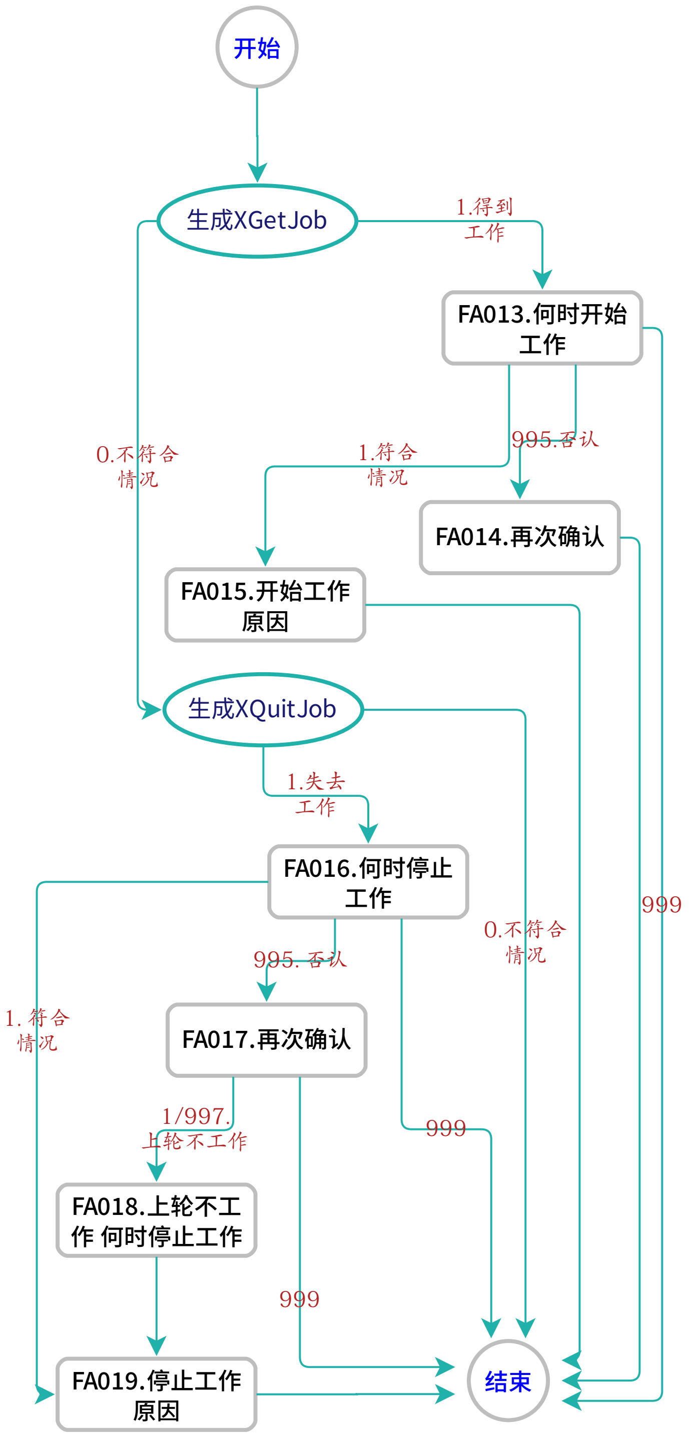
开始
生成XGetJob
1.得到工作
FA013.何时开始工作
995.否认
1.符合情况
0.不符合情况
FA014.再次确认
FA015.开始工作原因
生成XQuitJob
1.失去工作
FA016.何时停止工作
0.不符合情况
999
995. 否认
1. 符合情况
FA017.再次确认
1/997.上轮不工作
999
FA018.上轮不工作 何时停止工作
999
FA019.停止工作原因
结束

FB. 农业自雇工作

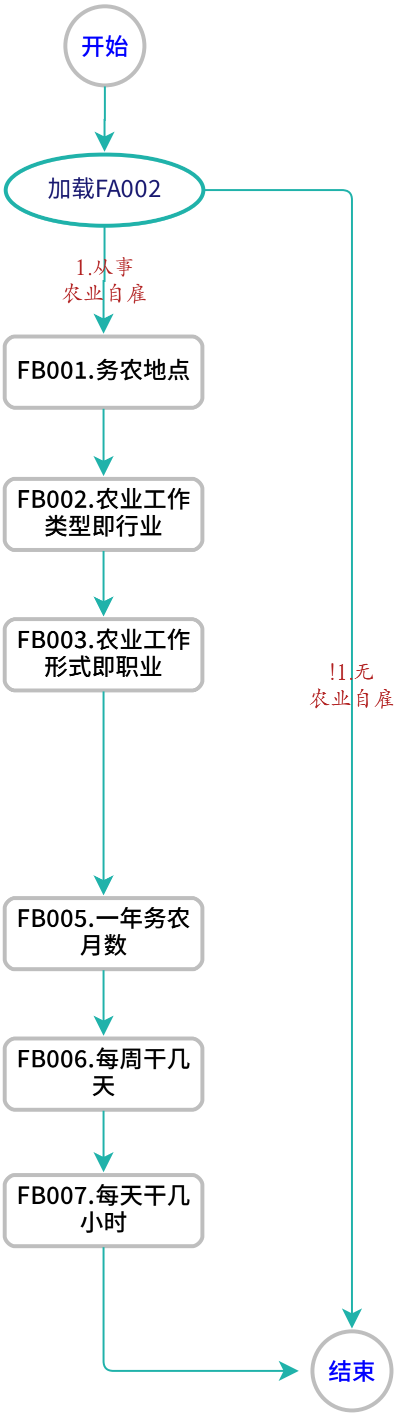
开始
加载FA002
1.从事
农业自雇
FB001.务农地点
FB002.农业工作
类型即行业
FB003.农业工作
形式即职业
FB005.一年务农
月数
FB006.每周干几
天
FB007.每天干几
小时
!1.无
农业自雇
结束

FC. 受雇工作（一）

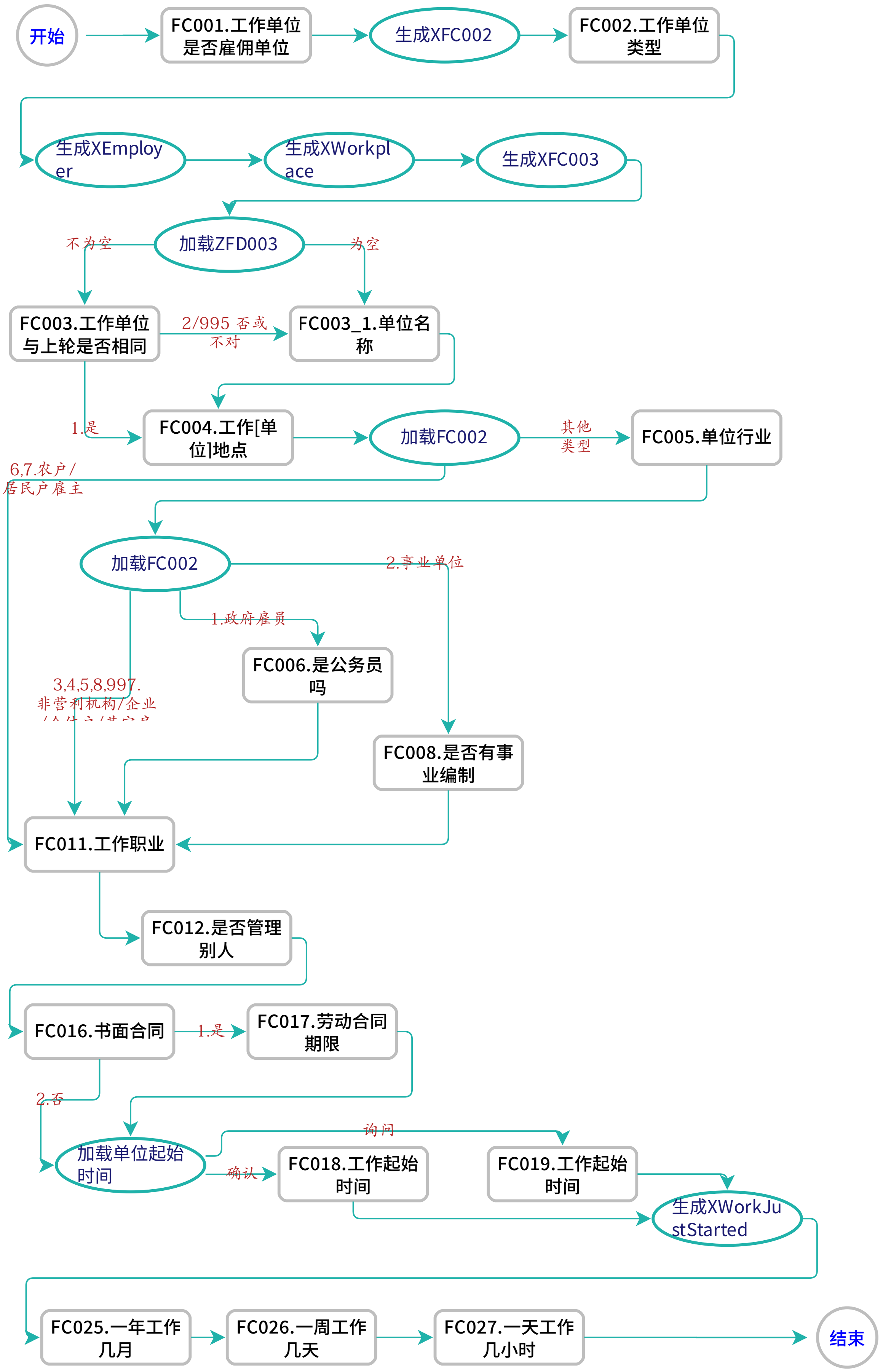
开始
FC001.工作单位是否雇佣单位
生成XFC002
FC002.工作单位类型
生成XEmployer
生成XWorkplace
生成XFC003
加载ZFD003
不为空
为空
FC003.工作单位与上轮是否相同
2/995 否或不对
FC003_1.单位名称
1.是
FC004.工作[单位]地点
加载FC002
其他类型
FC005.单位行业
6,7.农户/居民户雇主
加载FC002
2.事业单位
1.政府雇员
FC006.是公务员吗
3,4,5,8,997.
非营利机构/企业
FC008.是否有事业编制
FC011.工作职业
FC012.是否管理别人
FC016.书面合同
1.是
FC017.劳动合同期限
2.否
加载单位起始时间
询问
确认
FC018.工作起始时间
FC019.工作起始时间
生成XWorkJustStarted
FC025.一年工作几月
FC026.一周工作几天
FC027.一天工作几小时
结束

FC. 受雇工作（二）

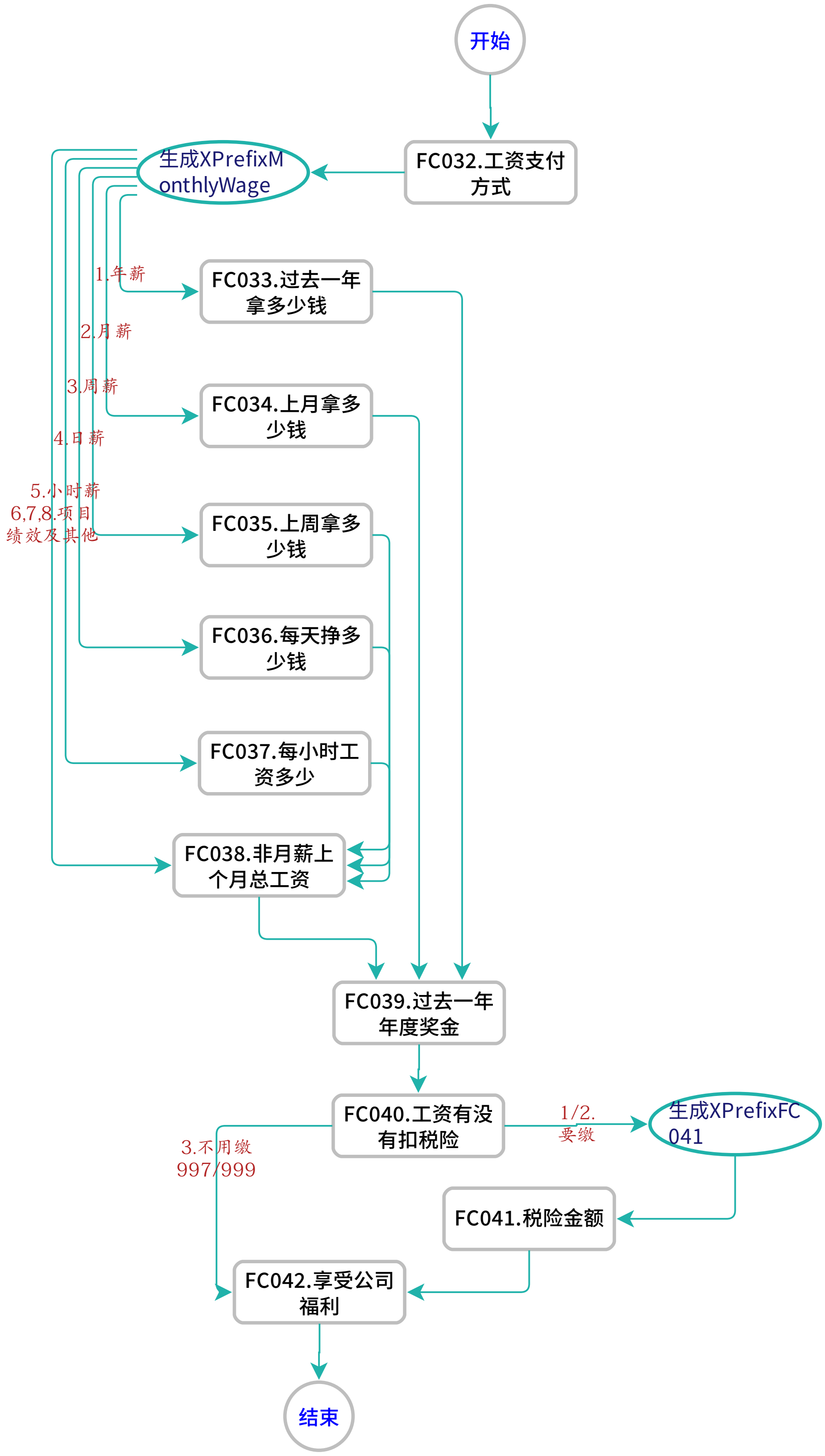
开始
FC032.工资支付方式
生成XPrefixMonthlyWage
1.年薪
2.月薪
3.周薪
4.日薪
5.小时薪
6,7,8.项目绩效及其他
FC033.过去一年拿多少钱
FC034.上月拿多少钱
FC035.上周拿多少钱
FC036.每天挣多少钱
FC037.每小时工资多少
FC038.非月薪上个月总工资
FC039.过去一年年度奖金
FC040.工资有没有扣税险
1/2.要缴
3.不用缴
997/999
生成XPrefixFC041
FC041.税险金额
FC042.享受公司福利
结束

FD. 非农自雇工作

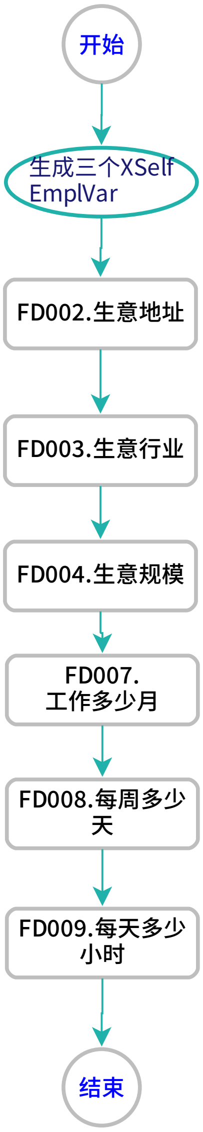
开始
生成三个XSelf EmplVar
FD002.生意地址
FD003.生意行业
FD004.生意规模
FD007.工作多少月
FD008.每周多少天
FD009.每天多少小时
结束

FE. 非主要工作

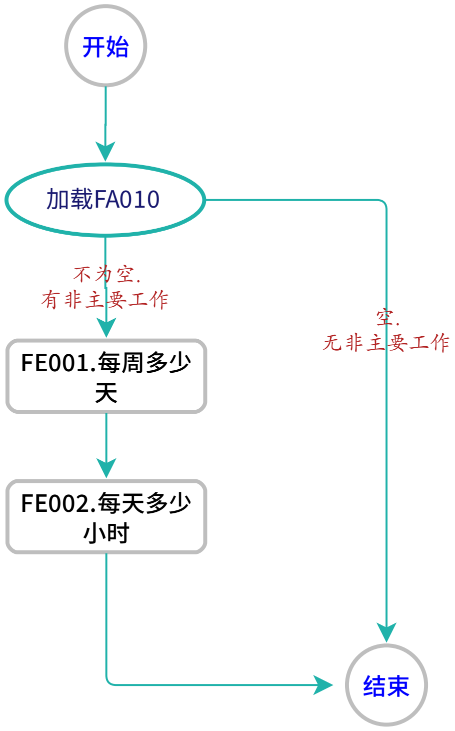

FF. 求职与就业

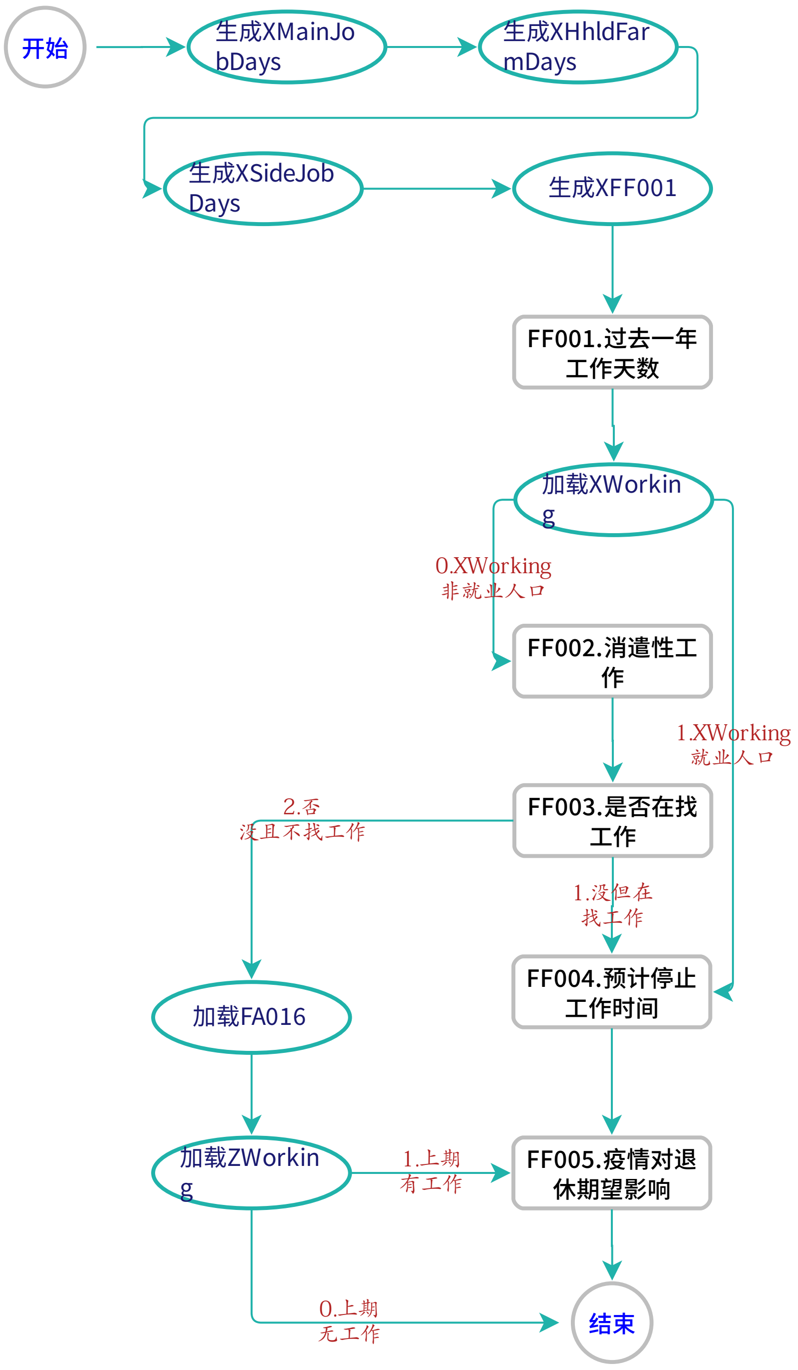
开始
生成XMainJobDays
生成XHhldFarmDays
生成XSideJobDays
生成XFF001
FF001.过去一年工作天数
加载XWorking
0.XWorking
非就业人口
FF002.消遣性工作
1.XWorking
就业人口
2.否
没且不找工作
FF003.是否在找工作
1.没但在
找工作
FF004.预计停止工作时间
加载FA016
加载ZWorking
1.上期
有工作
FF005.疫情对退休期望影响
0.上期
无工作
结束

FG. 疫情期间工作

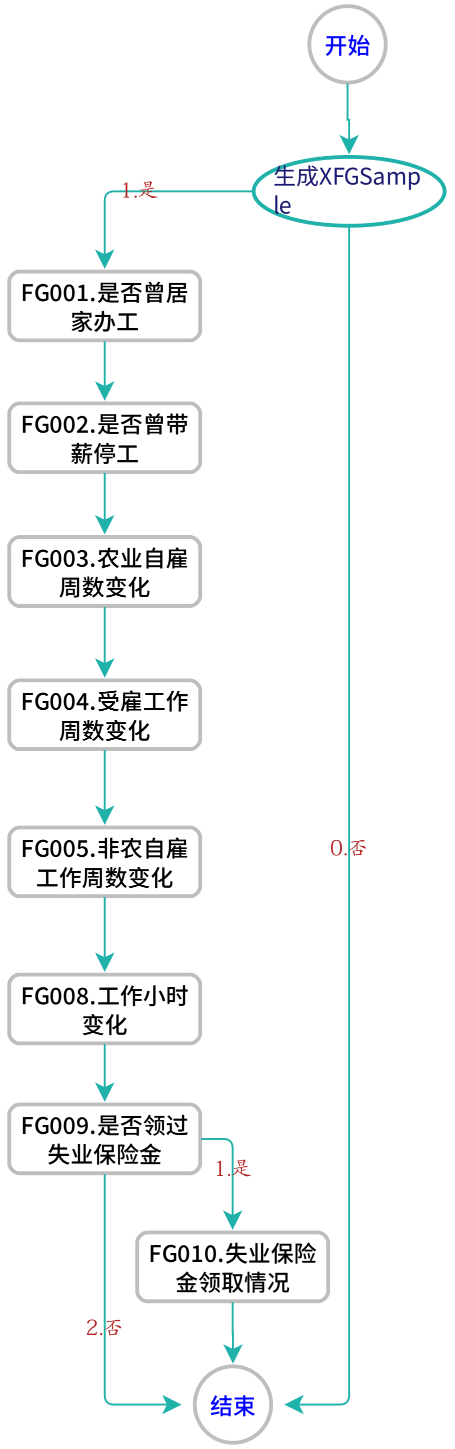
开始
生成XFGSample
1.是
0.否
FG001.是否曾居家办工
FG002.是否曾带薪停工
FG003.农业自雇周数变化
FG004.受雇工作周数变化
FG005.非农自雇工作周数变化
FG008.工作小时变化
FG009.是否领过失业保险金
1.是
2.否
FG010.失业保险金领取情况
结束

FH. 退休手续

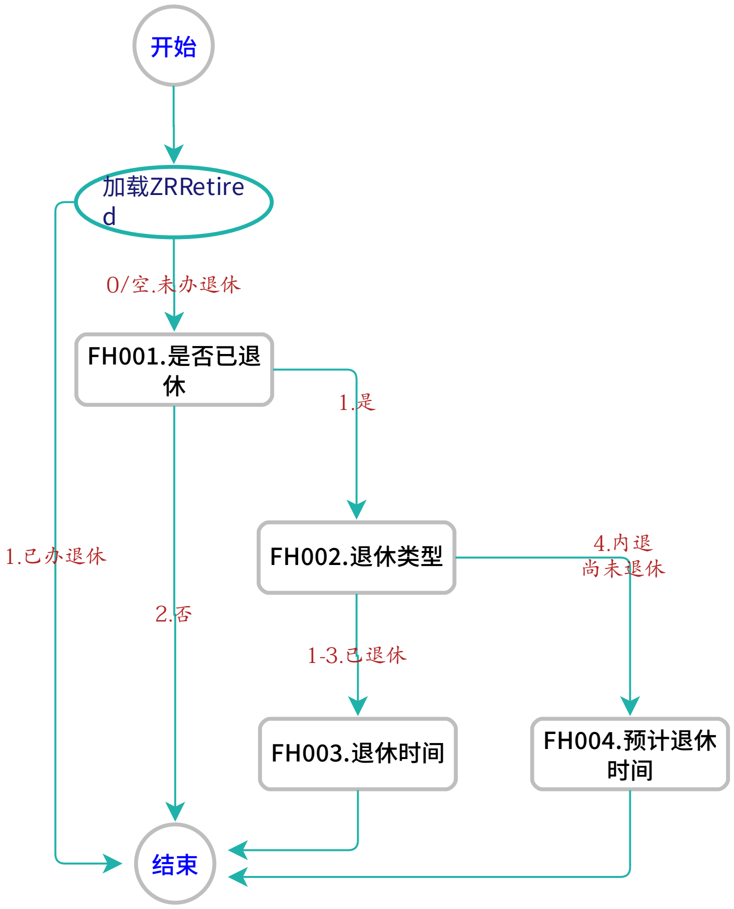
开始
加载ZRRetired
0/空.未办退休
FH001.是否已退休
1.是
FH002.退休类型
4.内退
尚未退休
1.已办退休
2.否
1-3.已退休
FH003.退休时间
FH004.预计退休时间
结束

This page intentionally left blank

G　收入与支出

G1 家户收入与支出

MAIN. 主逻辑

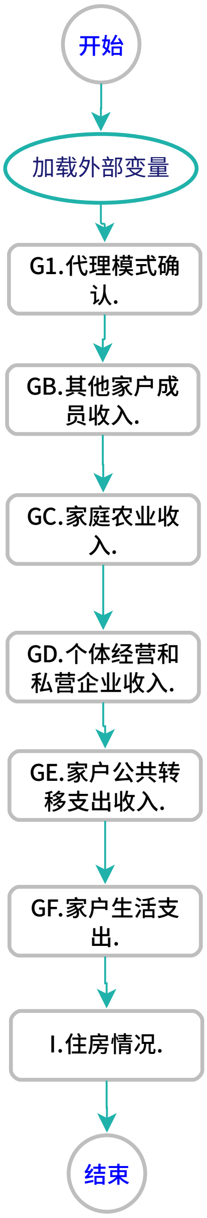

G1. 代理模式确认

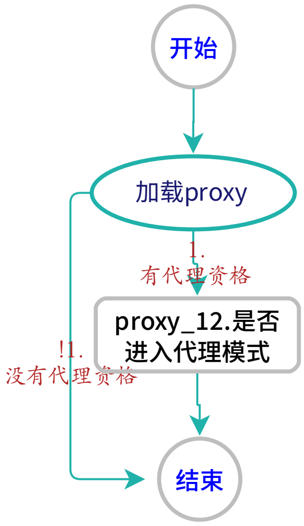

GB. 其他家户成员收入

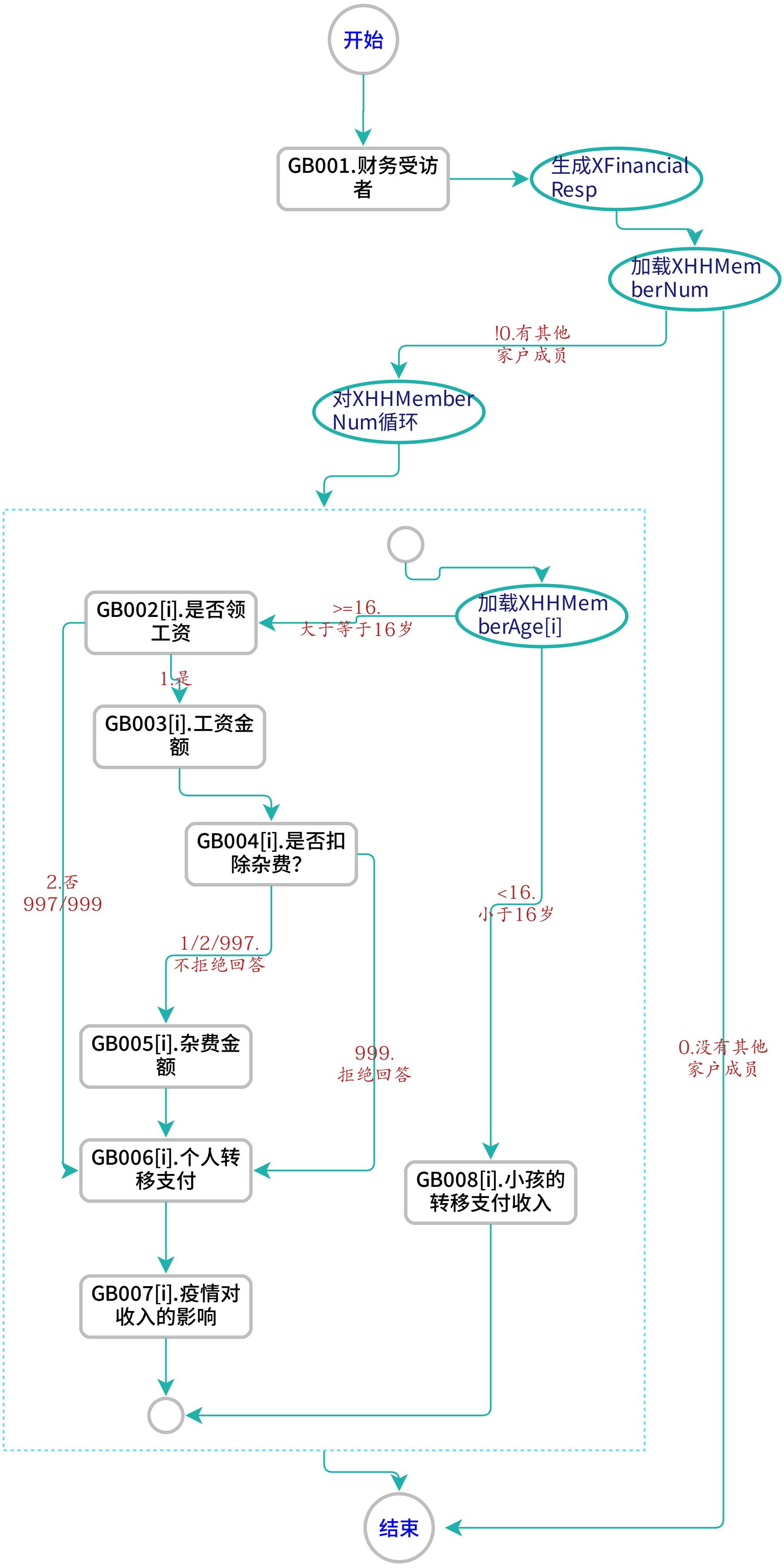
开始
GB001.财务受访者
生成XFinancialResp
加载XHHMemberNum
!0.有其他家户成员
0.没有其他家户成员
对XHHMemberNum循环
加载XHHMemberAge[i]
>=16.
大于等于16岁
<16.
小于16岁
GB002[i].是否领工资
1.是
2.否
997/999
GB003[i].工资金额
GB004[i].是否扣除杂费?
1/2/997.
不拒绝回答
999.
拒绝回答
GB005[i].杂费金额
GB006[i].个人转移支付
GB007[i].疫情对收入的影响
GB008[i].小孩的转移支付收入
结束

GC. 家庭农业收入

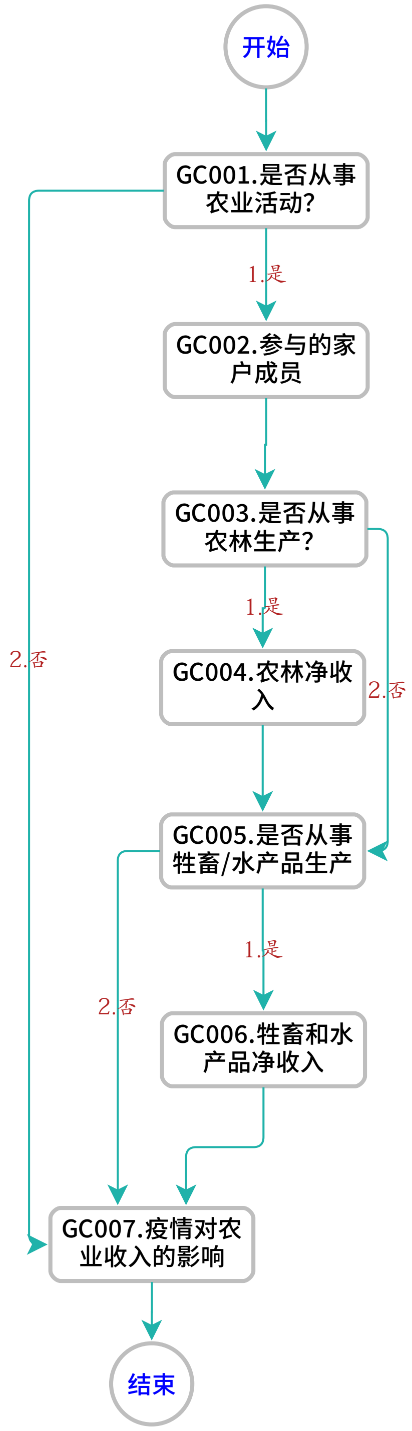
开始
GC001.是否从事农业活动?
1.是
2.否
GC002.参与的家户成员
GC003.是否从事农林生产?
1.是
2.否
GC004.农林净收入
GC005.是否从事牲畜/水产品生产
1.是
2.否
GC006.牲畜和水产品净收入
GC007.疫情对农业收入的影响
结束

GD. 个体经营和私营企业收入

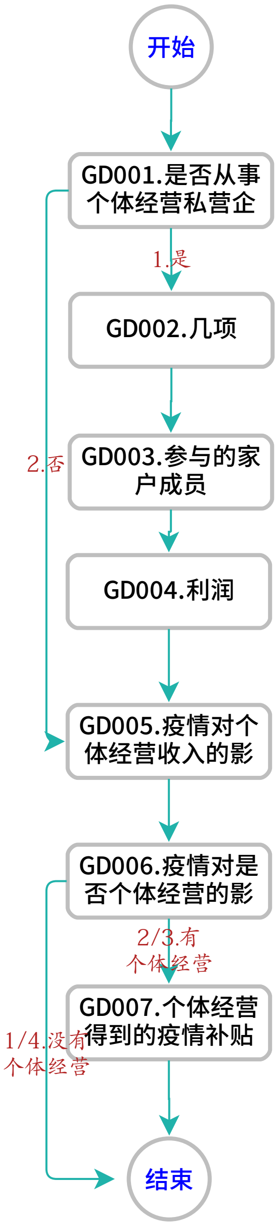

GE. 家户公共转移支出收入

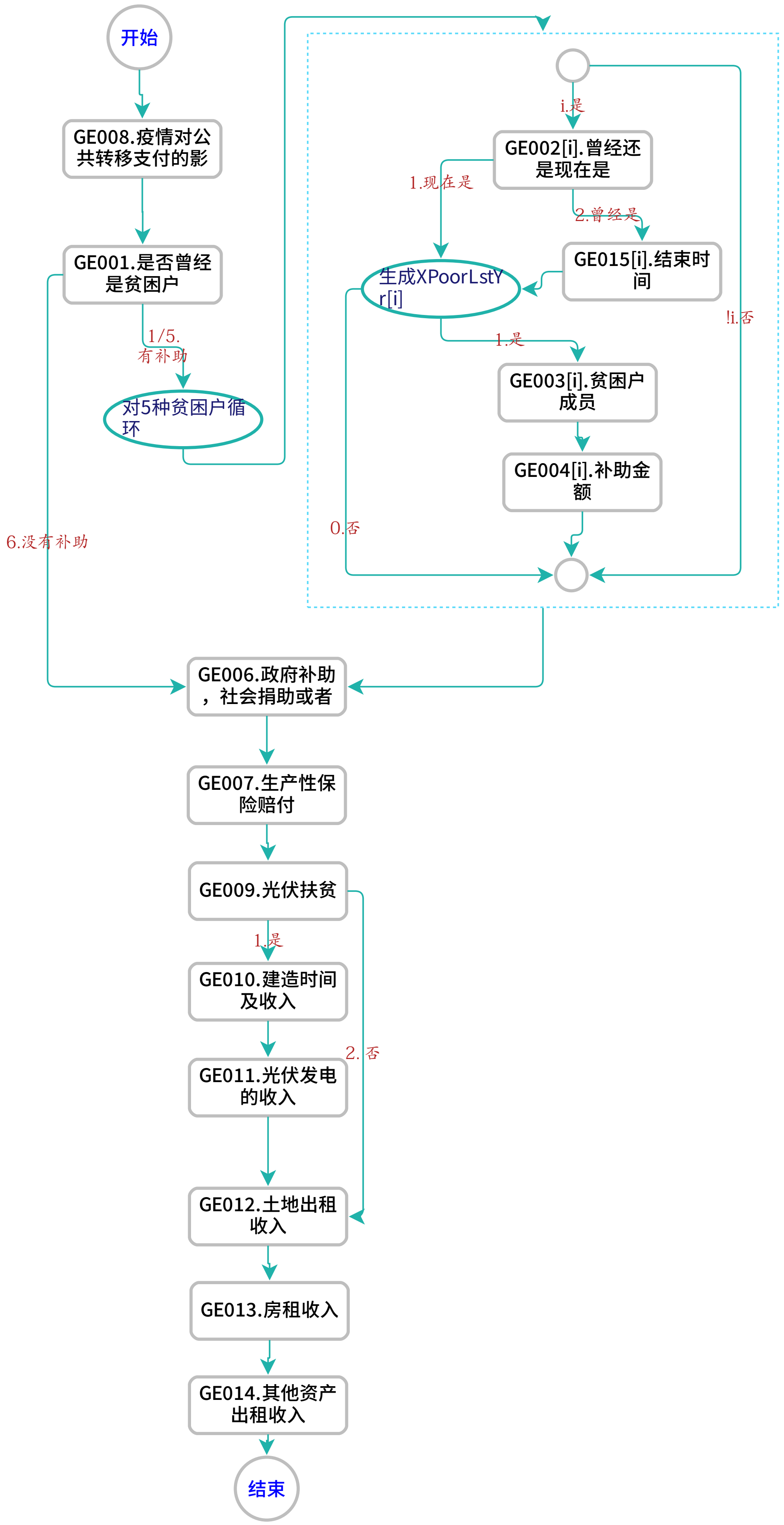

GF. 家户生活支出

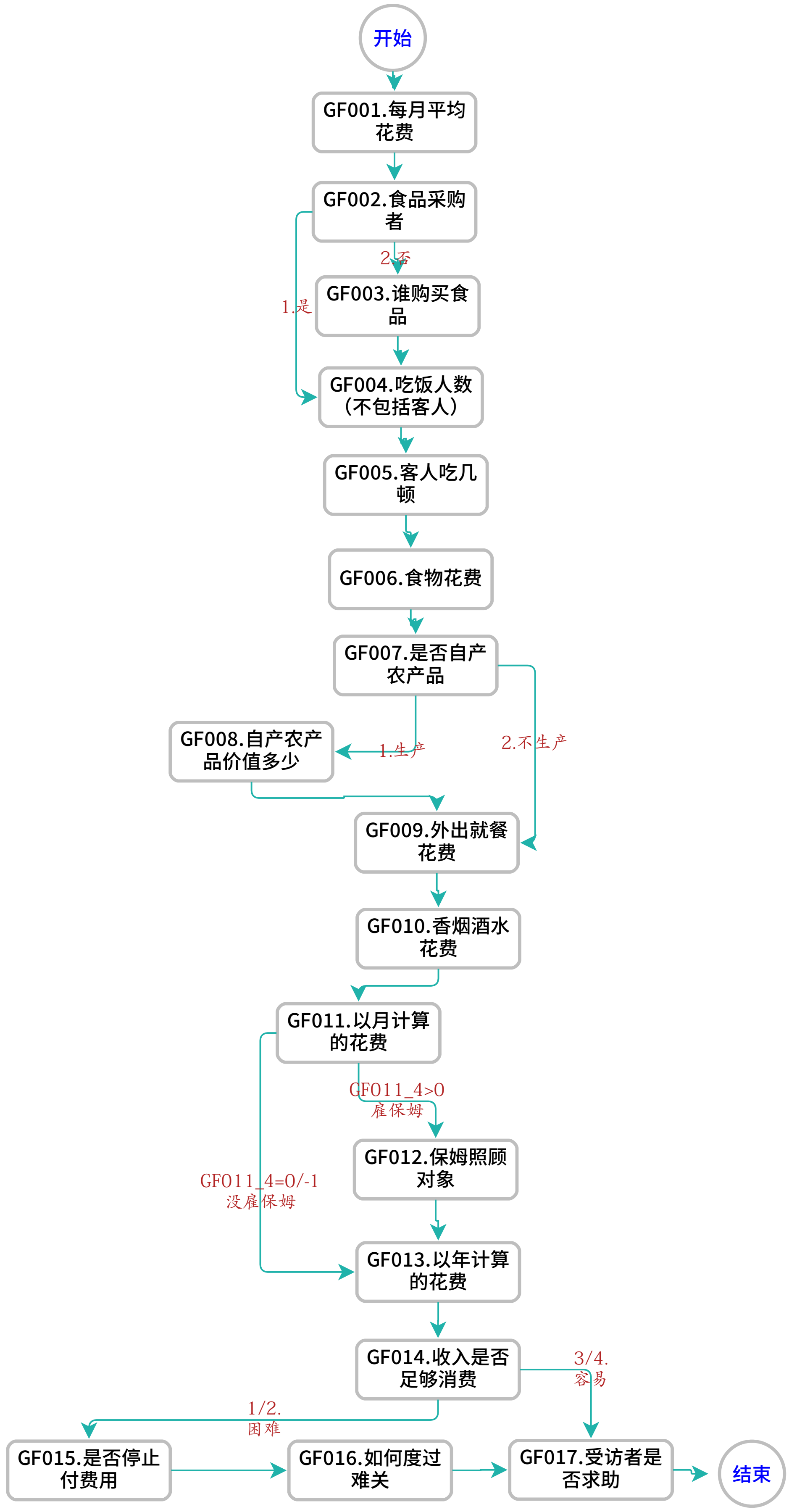
开始
GF001.每月平均花费
GF002.食品采购者
2.否
1.是
GF003.谁购买食品
GF004.吃饭人数（不包括客人）
GF005.客人吃几顿
GF006.食物花费
GF007.是否自产农产品
GF008.自产农产品价值多少
1.生产
2.不生产
GF009.外出就餐花费
GF010.香烟酒水花费
GF011.以月计算的花费
GF011_4>0
雇保姆
GF012.保姆照顾对象
GF011_4=0/-1
没雇保姆
GF013.以年计算的花费
GF014.收入是否足够消费
3/4.
容易
1/2.
困难
GF015.是否停止付费用
GF016.如何度过难关
GF017.受访者是否求助
结束

I. 住房情况

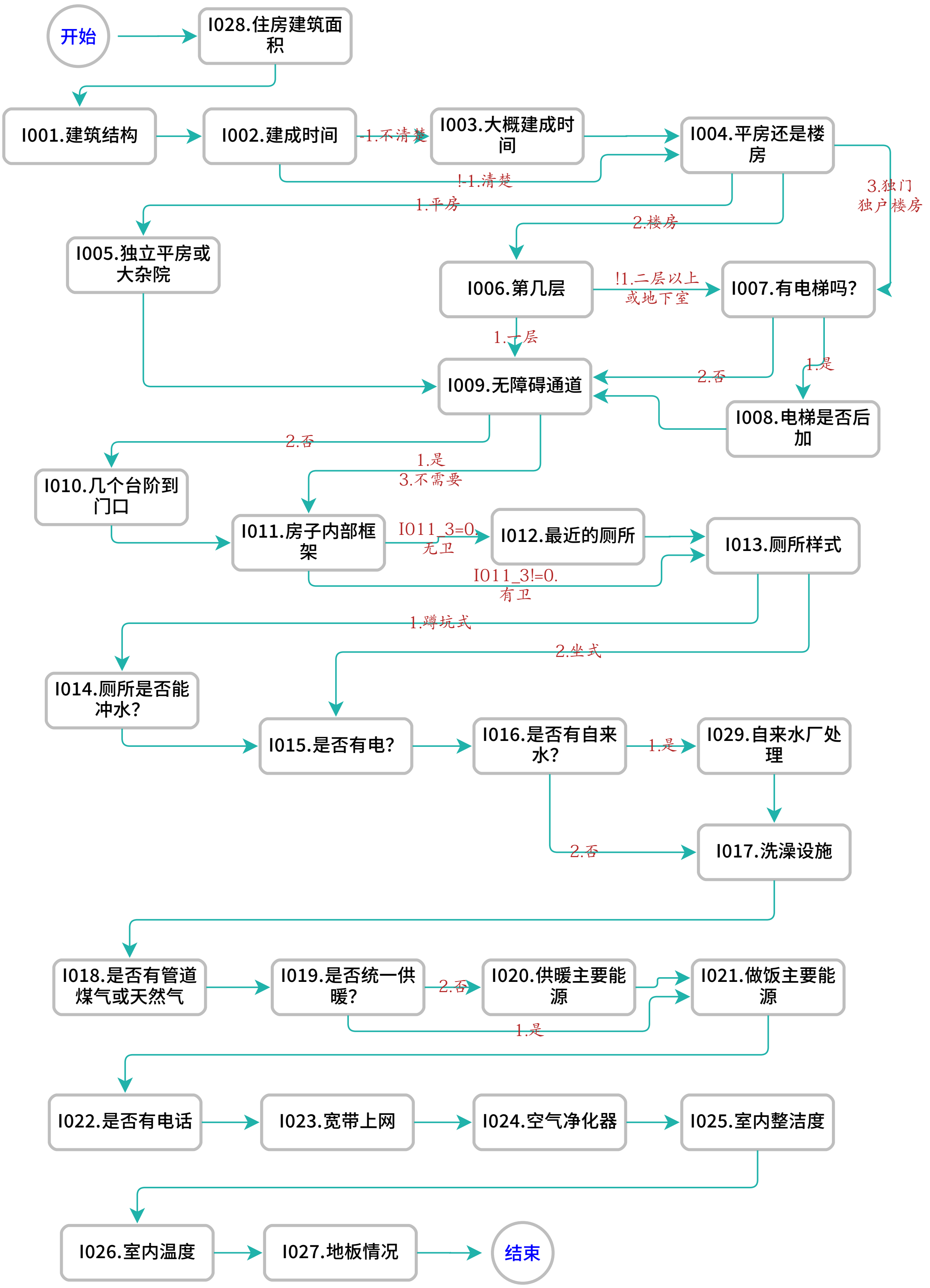
开始
I028.住房建筑面积
I001.建筑结构
I002.建成时间
1.不清楚
I003.大概建成时间
!1.清楚
I004.平房还是楼房
1.平房
2.楼房
3.独门独户楼房
I005.独立平房或大杂院
I006.第几层
!1.二层以上或地下室
I007.有电梯吗?
1.一层
I009.无障碍通道
2.否
1.是
I008.电梯是否后加
2.否
I010.几个台阶到门口
1.是
3.不需要
I011.房子内部框架
I011_3=0.无卫
I012.最近的厕所
I011_3!=0.有卫
I013.厕所样式
1.蹲坑式
2.坐式
I014.厕所是否能冲水?
I015.是否有电?
I016.是否有自来水?
1.是
I029.自来水厂处理
2.否
I017.洗澡设施
I018.是否有管道煤气或天然气
I019.是否统一供暖?
2.否
I020.供暖主要能源
1.是
I021.做饭主要能源
I022.是否有电话
I023.宽带上网
I024.空气净化器
I025.室内整洁度
I026.室内温度
I027.地板情况
结束

G2 个人收入

MAIN. 主逻辑

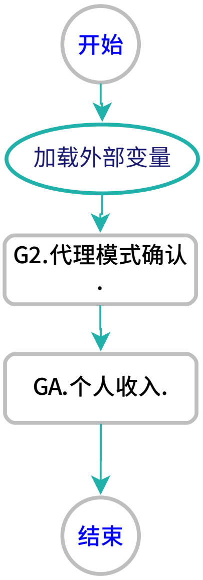

G2. 代理模式确认

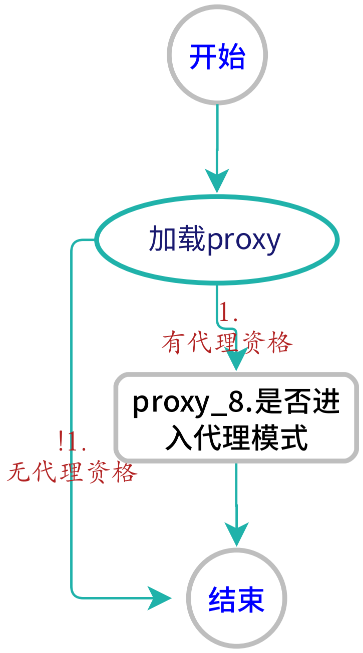

GA. 个人收入

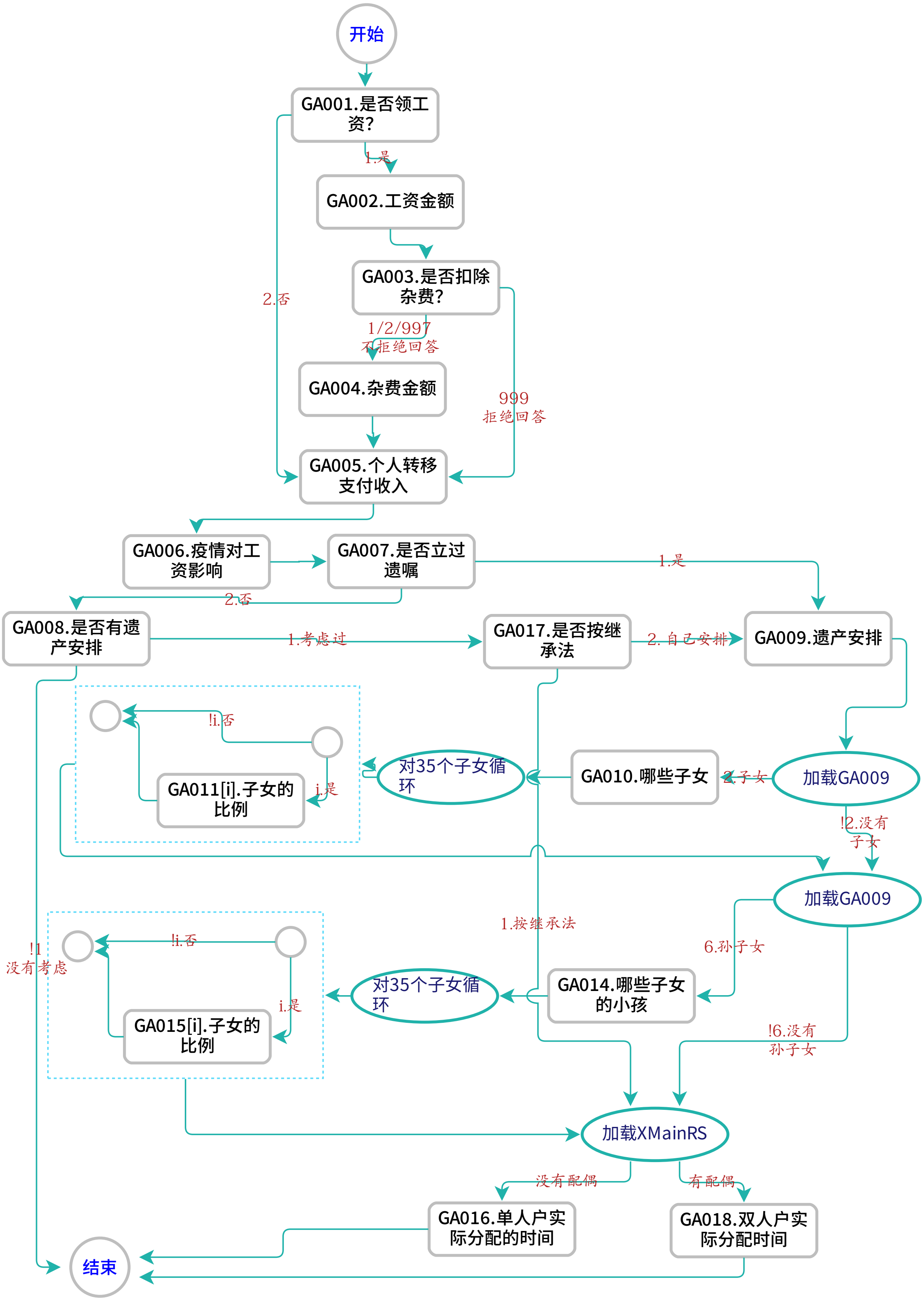
开始
GA001.是否领工资?
1.是
2.否
GA002.工资金额
GA003.是否扣除杂费?
1/2/997
不拒绝回答
999
拒绝回答
GA004.杂费金额
GA005.个人转移支付收入
GA006.疫情对工资影响
GA007.是否立过遗嘱
1.是
2.否
GA008.是否有遗产安排
1.考虑过
GA017.是否按继承法
2.自己安排
GA009.遗产安排
!i.否
i.是
GA011[i].子女的比例
对35个子女循环
GA010.哪些子女
2.子女
加载GA009
!2.没有子女
加载GA009
1.按继承法
6.孙子女
!1
没有考虑
!i.否
i.是
GA015[i].子女的比例
对35个子女循环
GA014.哪些子女的小孩
!6.没有孙子女
加载XMainRS
没有配偶
有配偶
GA016.单人户实际分配的时间
GA018.双人户实际分配时间
结束

V　疫情

MAIN. 主逻辑

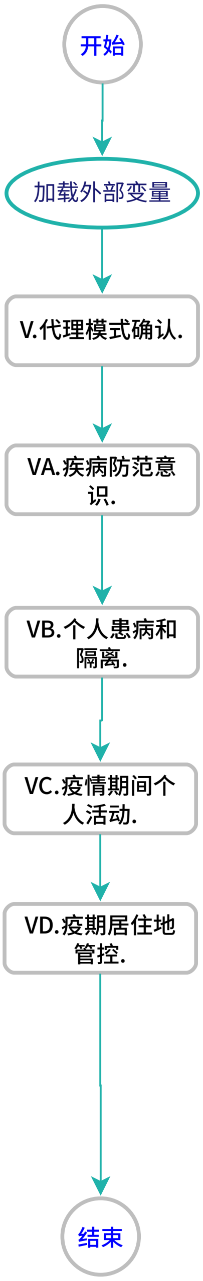
开始
加载外部变量
V.代理模式确认.
VA.疾病防范意识.
VB.个人患病和隔离.
VC.疫情期间个人活动.
VD.疫期居住地管控.
结束

V. 代理模式确认

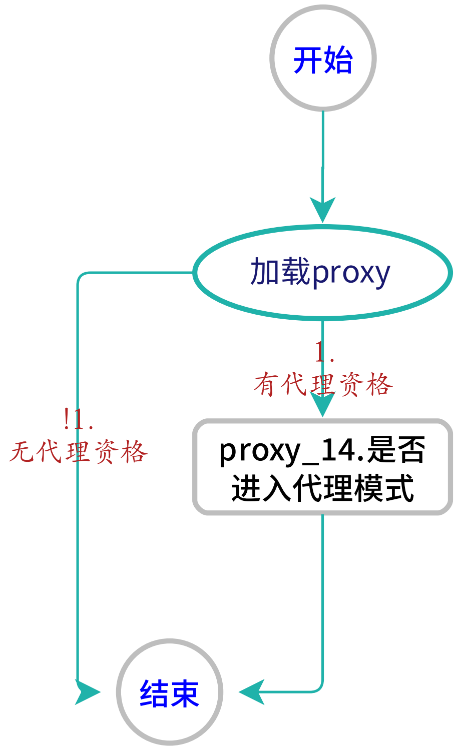

VA. 疾病防范意识

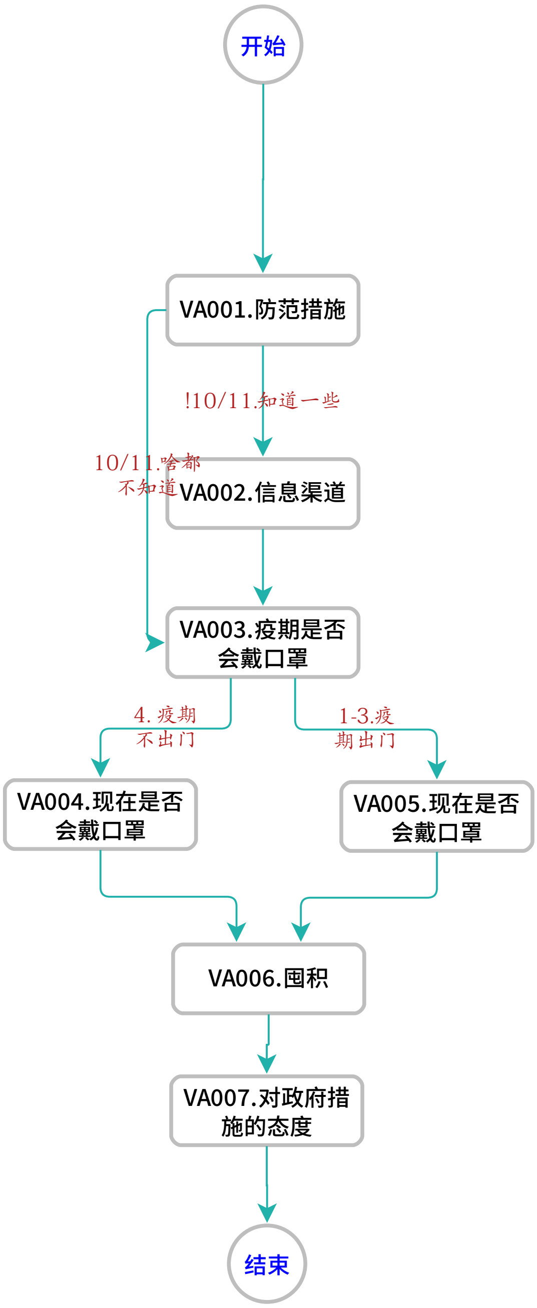
开始
VA001.防范措施
!10/11.知道一些
10/11.啥都不知道
VA002.信息渠道
VA003.疫期是否会戴口罩
4. 疫期不出门
1-3.疫期出门
VA004.现在是否会戴口罩
VA005.现在是否会戴口罩
VA006.囤积
VA007.对政府措施的态度
结束

VB. 个人患病和隔离

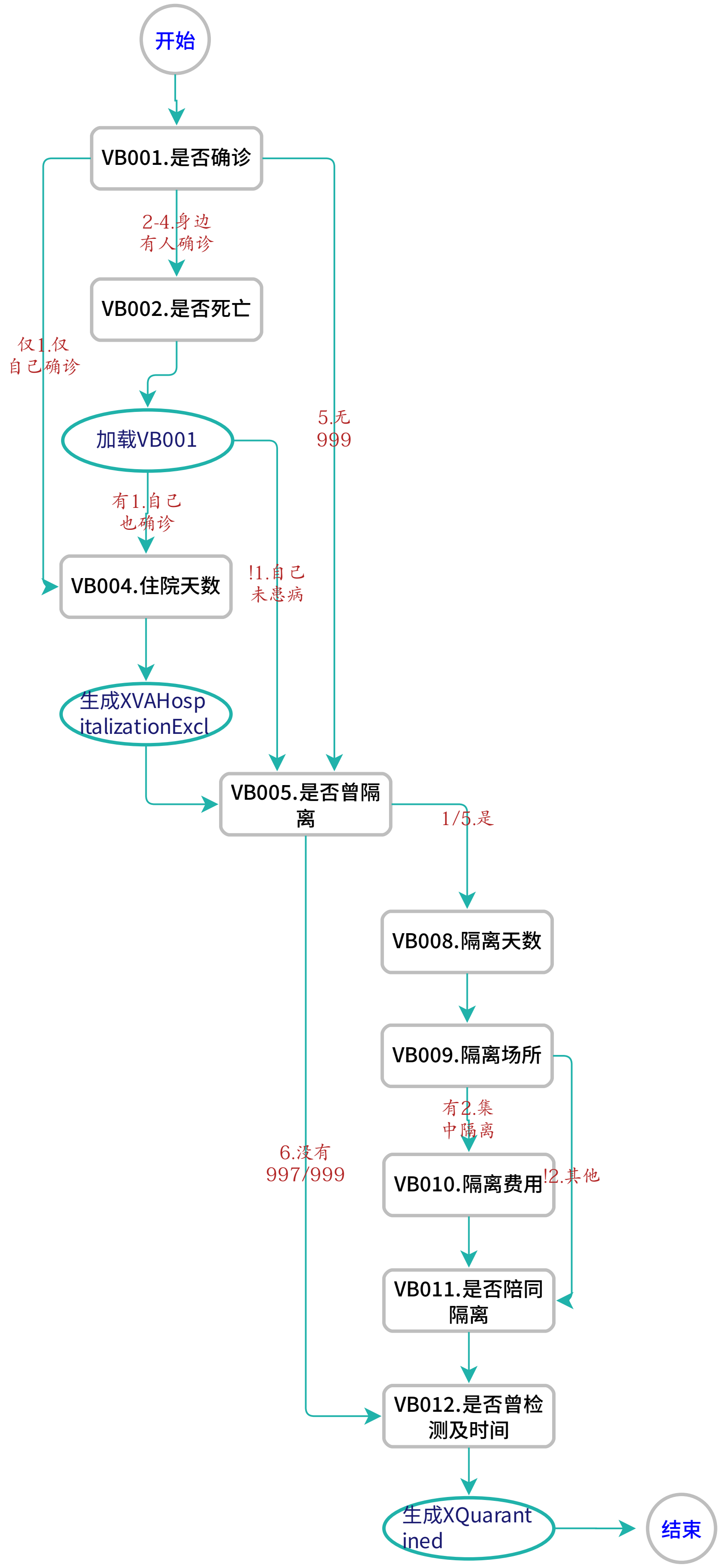
开始
VB001.是否确诊
2-4.身边有人确诊
VB002.是否死亡
仅1.仅自己确诊
5.无999
加载VB001
有1.自己也确诊
VB004.住院天数
!1.自己未患病
生成XVAHospitalizationExcl
VB005.是否曾隔离
1/5.是
VB008.隔离天数
VB009.隔离场所
有2.集中隔离
6.没有997/999
VB010.隔离费用
!2.其他
VB011.是否陪同隔离
VB012.是否曾检测及时间
生成XQuarantined
结束

VC. 疫情期间个人活动

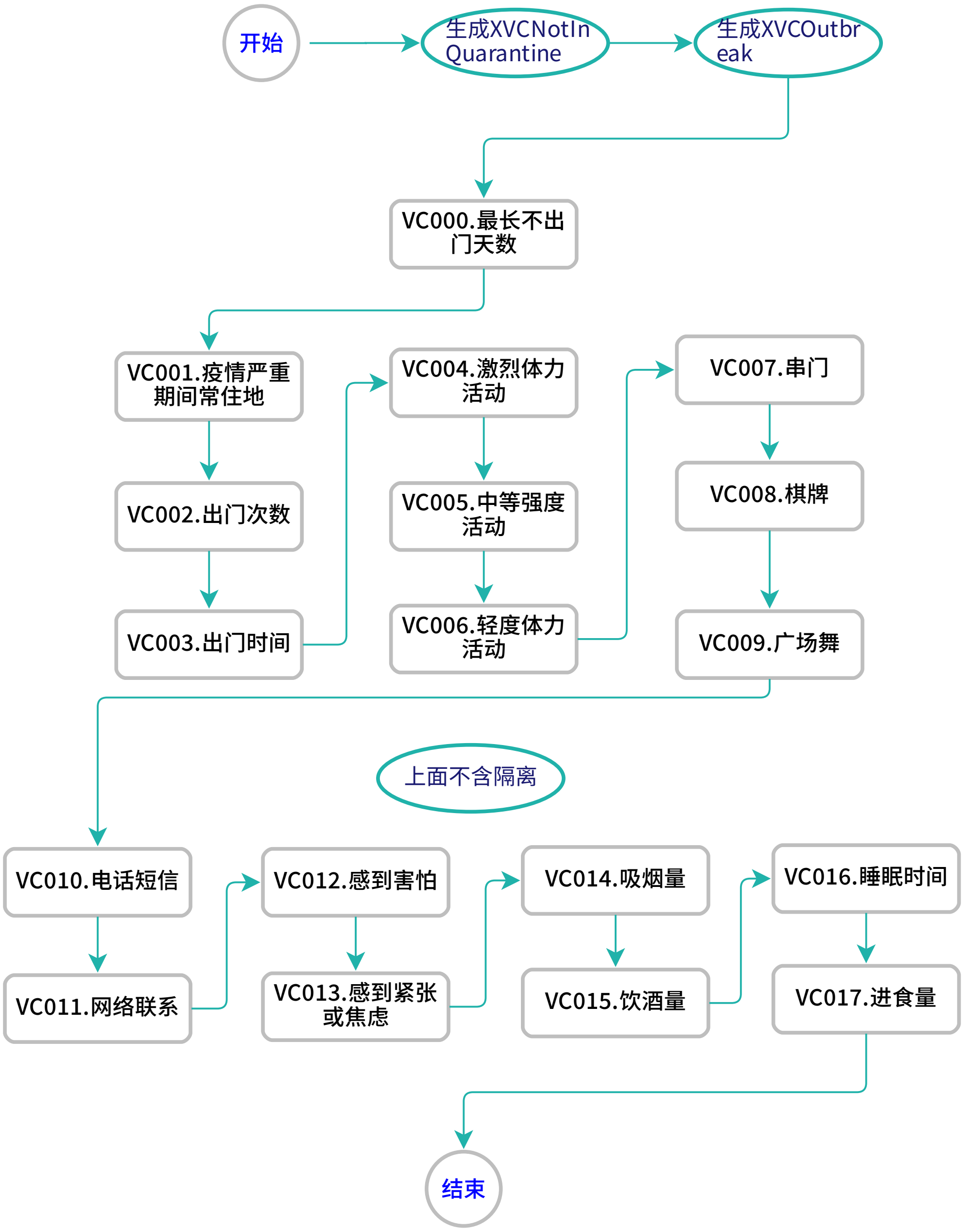
开始
生成XVCNotIn Quarantine
生成XVCOutbreak
VC000.最长不出门天数
VC001.疫情严重期间常住地
VC002.出门次数
VC003.出门时间
VC004.激烈体力活动
VC005.中等强度活动
VC006.轻度体力活动
VC007.串门
VC008.棋牌
VC009.广场舞
上面不含隔离
VC010.电话短信
VC011.网络联系
VC012.感到害怕
VC013.感到紧张或焦虑
VC014.吸烟量
VC015.饮酒量
VC016.睡眠时间
VC017.进食量
结束

VD. 疫期居住地管控

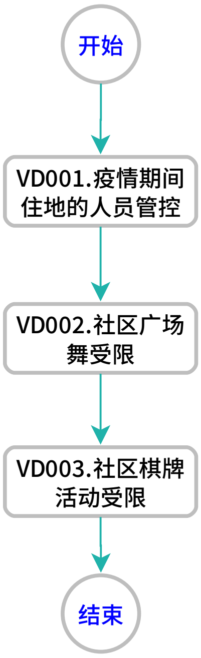

EX　退出问卷

MAIN. 主逻辑

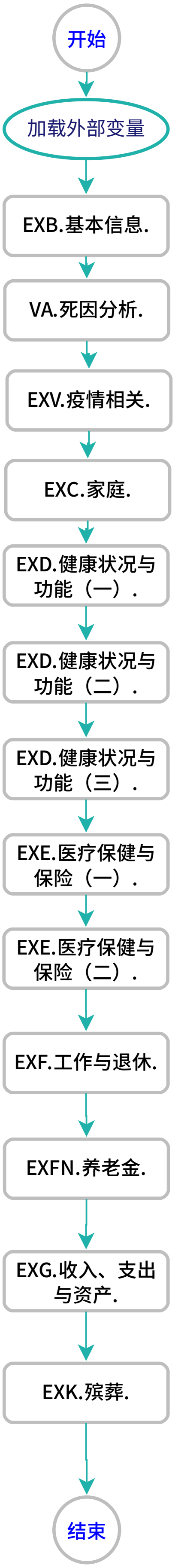
开始
加载外部变量
EXB.基本信息.
VA.死因分析.
EXV.疫情相关.
EXC.家庭.
EXD.健康状况与功能（一）.
EXD.健康状况与功能（二）.
EXD.健康状况与功能（三）.
EXE.医疗保健与保险（一）.
EXE.医疗保健与保险（二）.
EXF.工作与退休.
EXFN.养老金.
EXG.收入、支出与资产.
EXK.殡葬.
结束

EXB. 基本信息

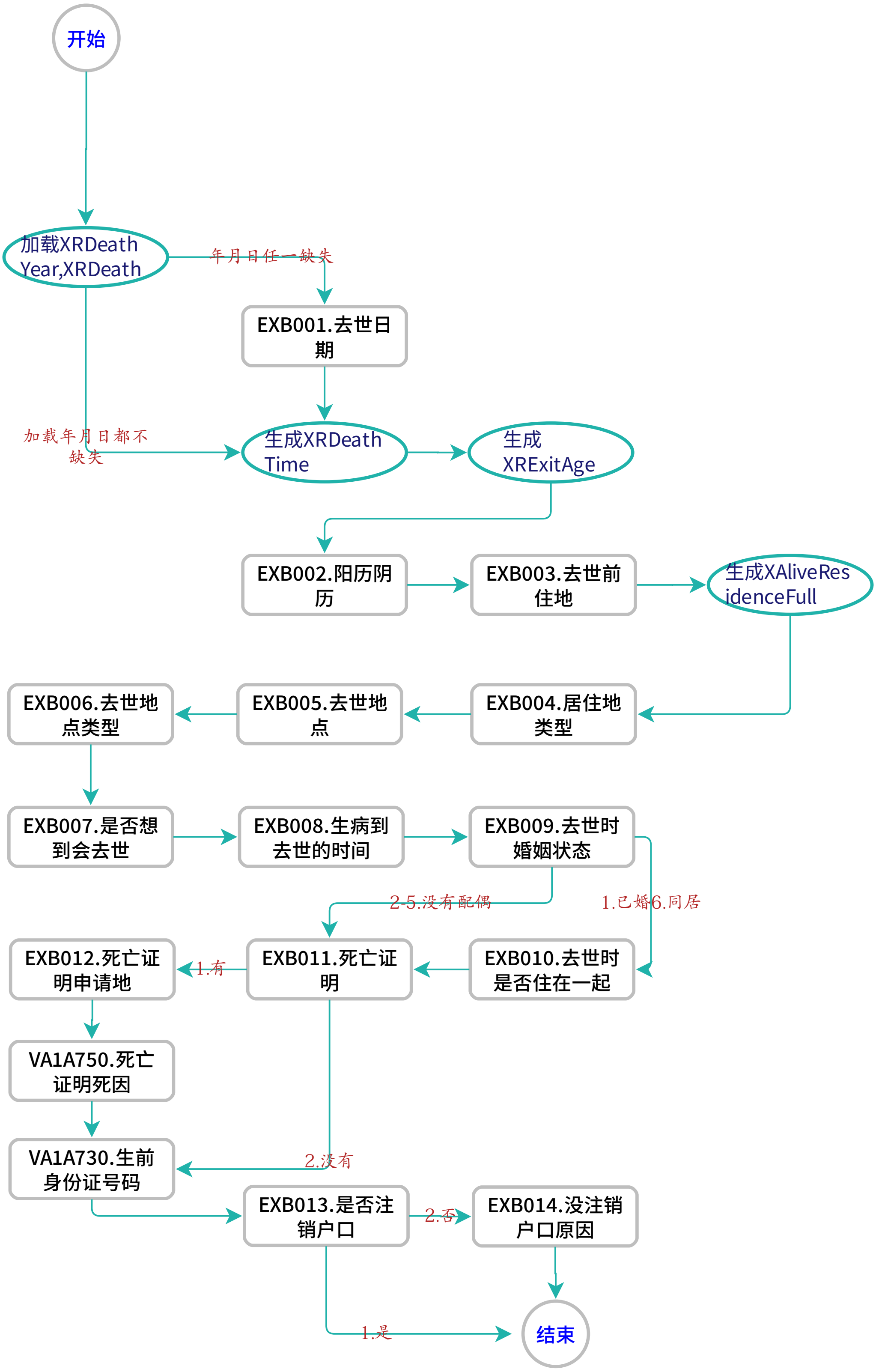
开始
加载XRDeath Year,XRDeath
年月日任一缺失
EXB001.去世日期
加载年月日都不缺失
生成XRDeath Time
生成 XRExitAge
EXB002.阳历阴历
EXB003.去世前住地
生成XAliveResidenceFull
EXB004.居住地类型
EXB005.去世地点
EXB006.去世地点类型
EXB007.是否想到会去世
EXB008.生病到去世的时间
EXB009.去世时婚姻状态
2-5.没有配偶
1.已婚6.同居
EXB010.去世时是否住在一起
EXB011.死亡证明
1.有
EXB012.死亡证明申请地
VA1A750.死亡证明死因
VA1A730.生前身份证号码
2.没有
EXB013.是否注销户口
2.否
EXB014.没注销户口原因
1.是
结束

EXC. 家庭

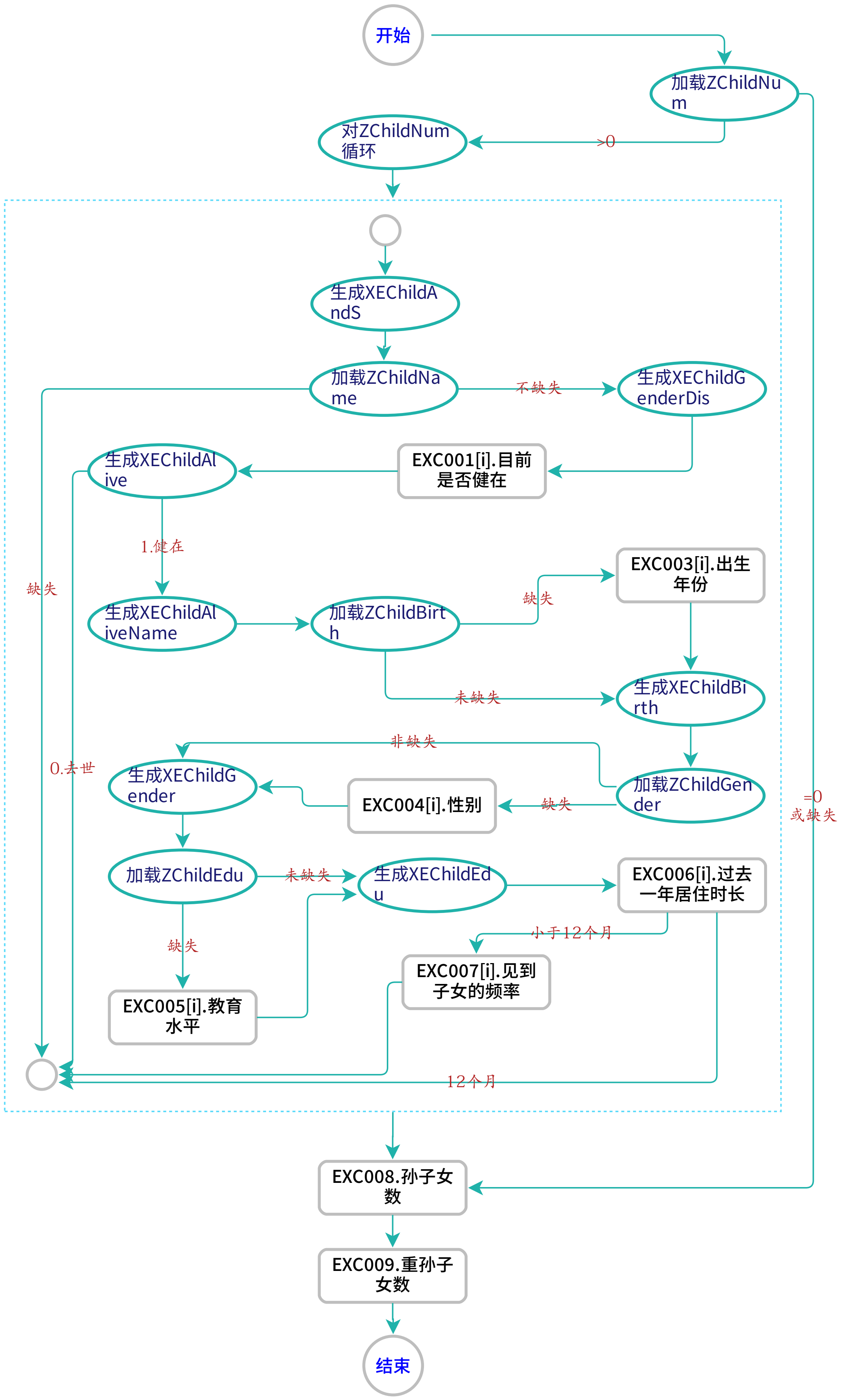
开始
加载ZChildNum
>0
对ZChildNum循环
生成XEChildAndS
加载ZChildName
不缺失
生成XEChildGenderDis
EXC001[i].目前是否健在
生成XEChildAlive
1.健在
缺失
生成XEChildAliveName
加载ZChildBirth
缺失
EXC003[i].出生年份
未缺失
生成XEChildBirth
非缺失
0.去世
生成XEChildGender
EXC004[i].性别
缺失
加载ZChildGender
=0
或缺失
加载ZChildEdu
未缺失
生成XEChildEdu
EXC006[i].过去一年居住时长
缺失
小于12个月
EXC005[i].教育水平
EXC007[i].见到子女的频率
12个月
EXC008.孙子女数
EXC009.重孙子女数
结束

EXD. 健康状况与功能（一）

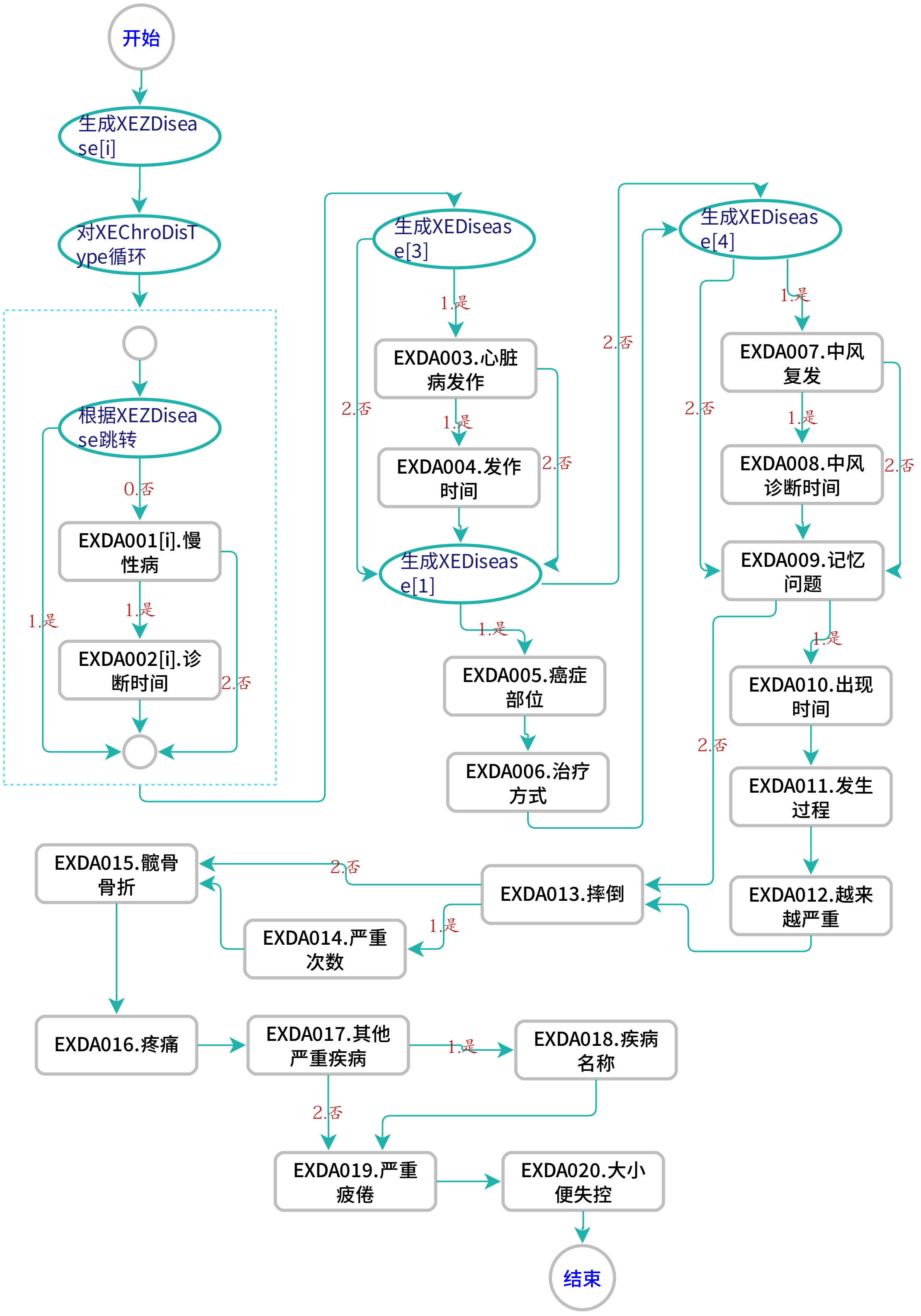

EXD. 健康状况与功能（二）

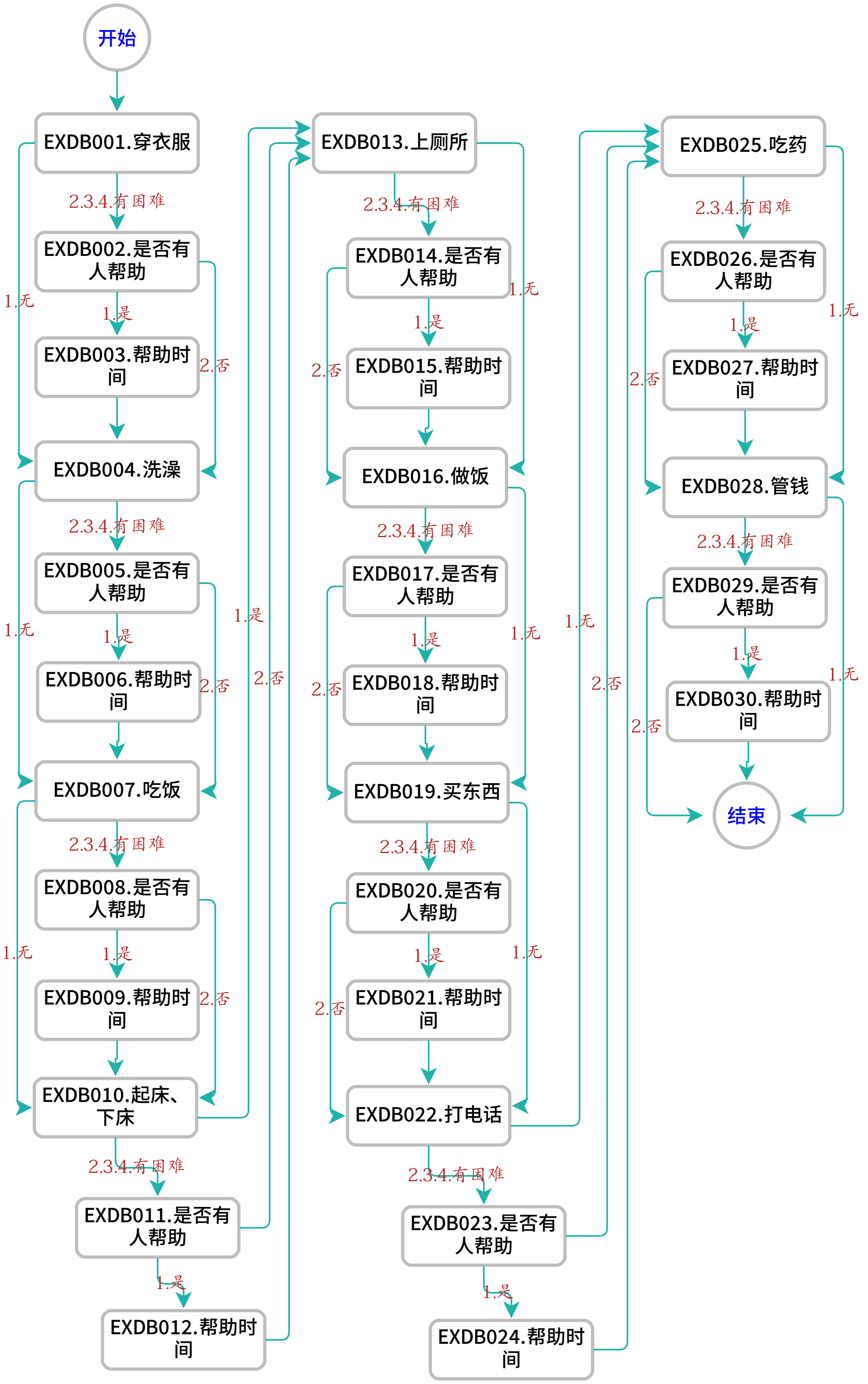

EXD. 健康状况与功能（三）

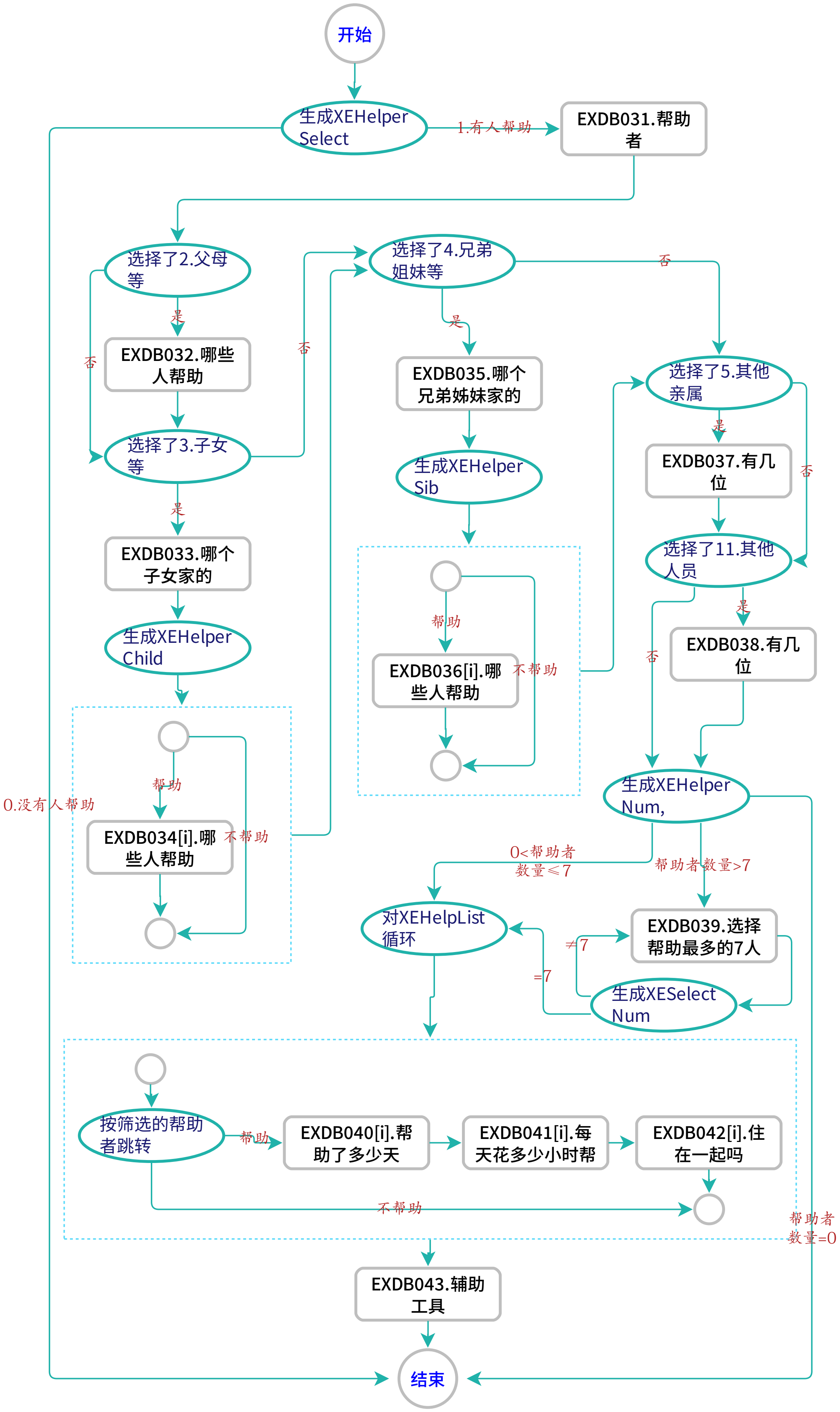

EXE. 医疗保健与保险（一）

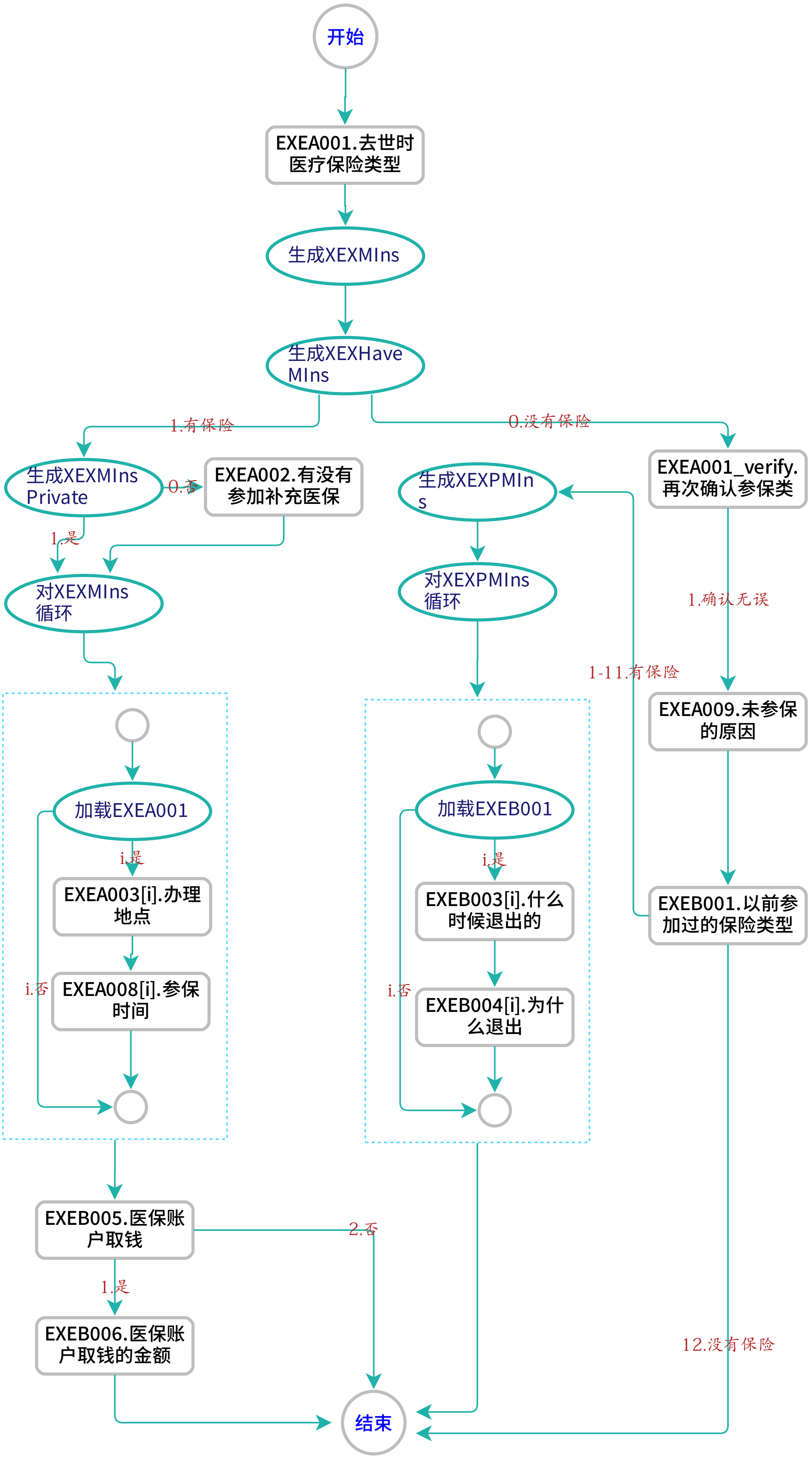

EXE. 医疗保健与保险（二）

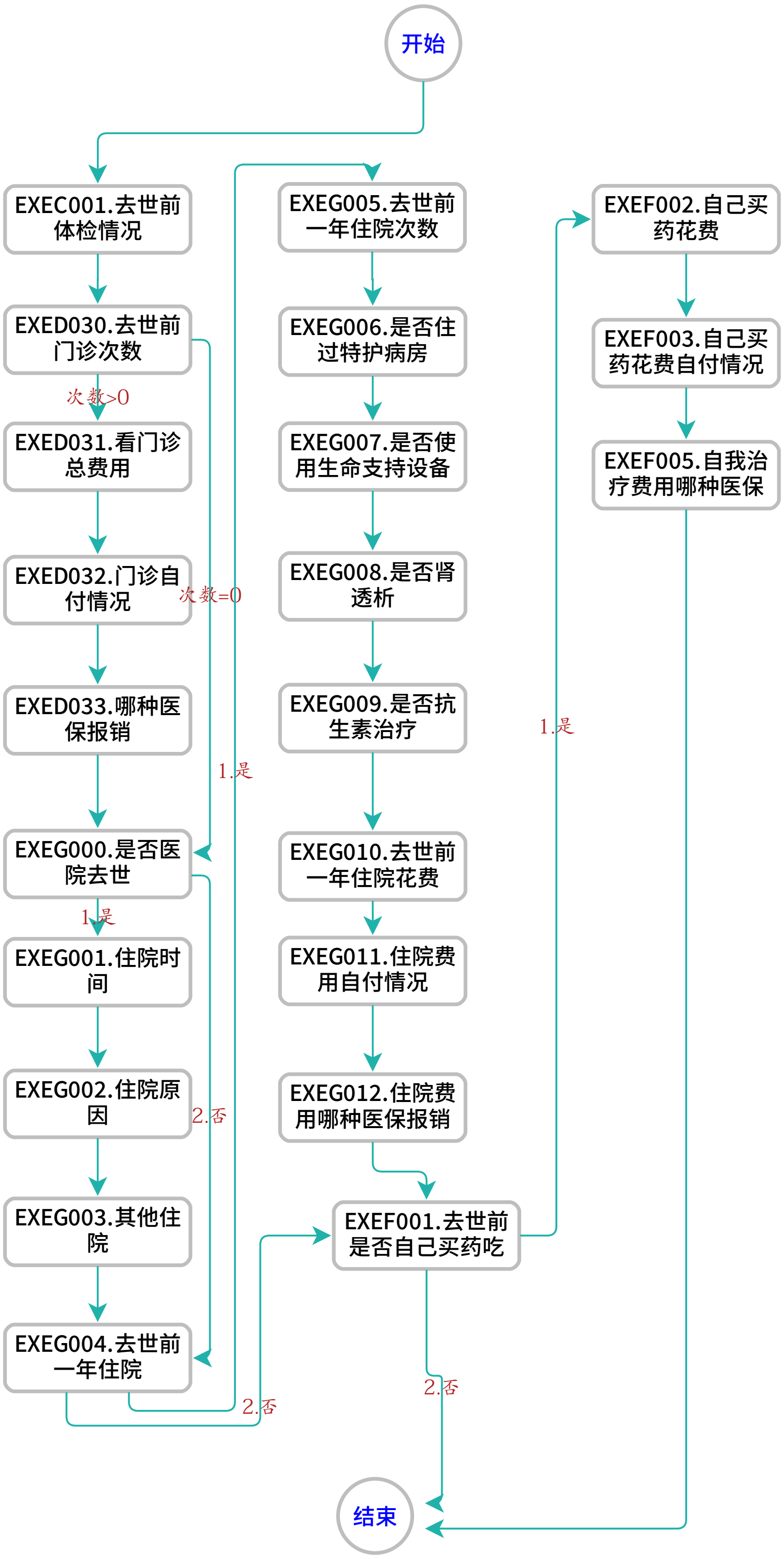

EXF. 工作与退休

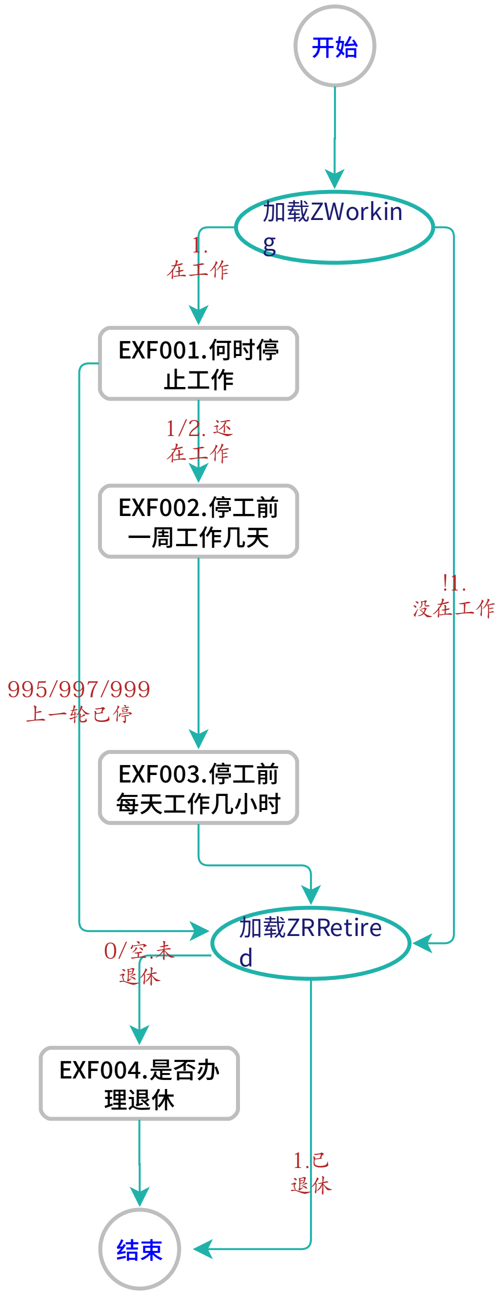
开始
加载ZWorking
1.
在工作
EXF001.何时停止工作
1/2. 还
在工作
EXF002.停工前一周工作几天
!1.
没在工作
995/997/999
上一轮已停
EXF003.停工前每天工作几小时
加载ZRRetired
0/空.未
退休
EXF004.是否办理退休
1.已
退休
结束

EXFN. 养老金

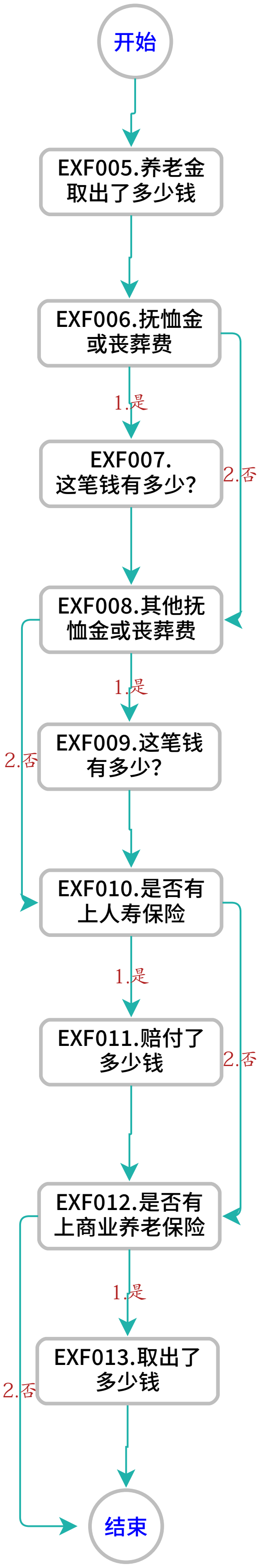
开始
EXF005.养老金取出了多少钱
EXF006.抚恤金或丧葬费
1.是
2.否
EXF007.这笔钱有多少?
EXF008.其他抚恤金或丧葬费
1.是
2.否
EXF009.这笔钱有多少?
EXF010.是否有上人寿保险
1.是
2.否
EXF011.赔付了多少钱
EXF012.是否有上商业养老保险
1.是
2.否
EXF013.取出了多少钱
结束

EXG. 收入、支出与资产

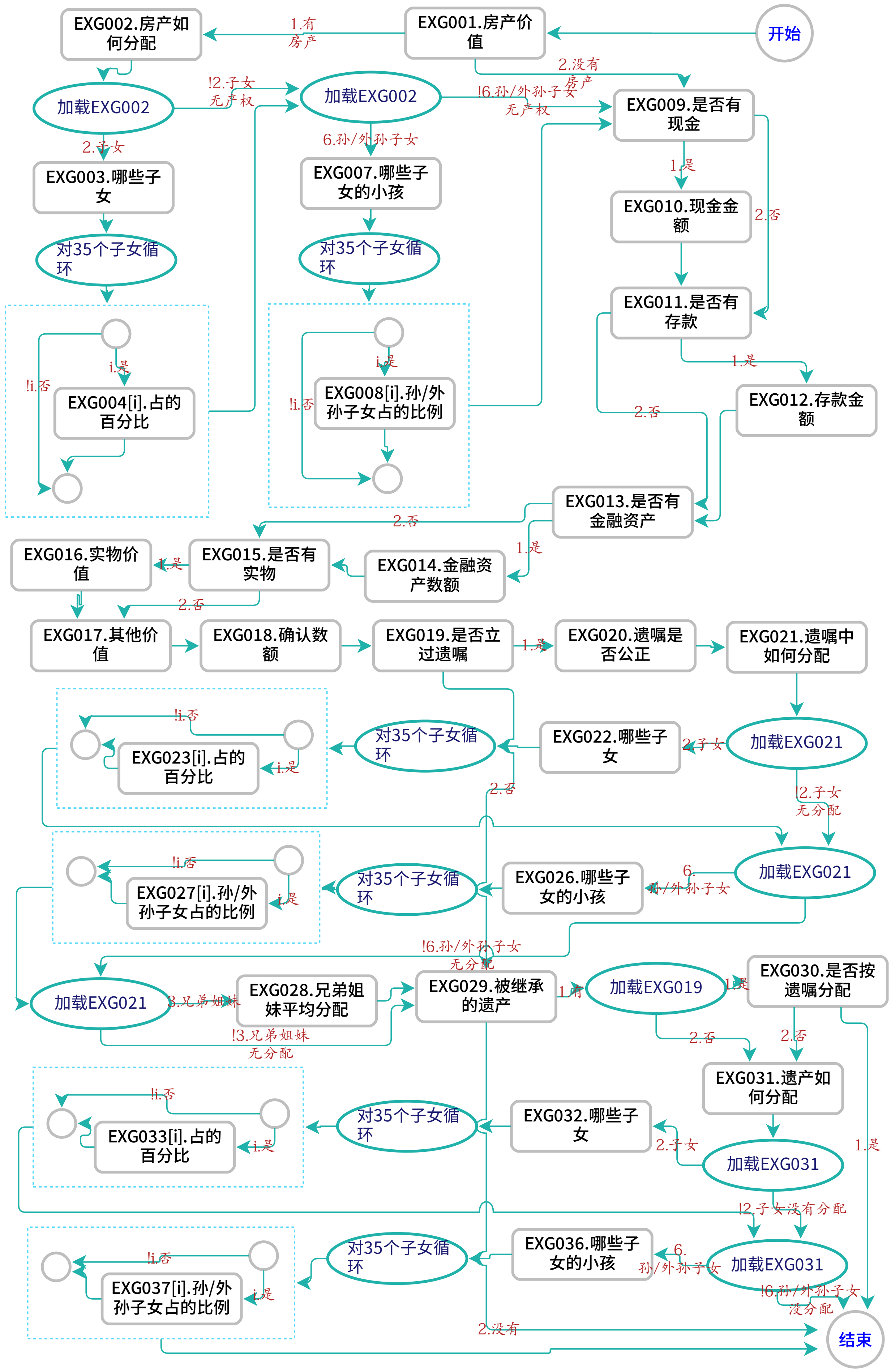
开始
EXG001.房产价值
1.有房产
2.没有房产
EXG002.房产如何分配
加载EXG002
!2.子女无产权
2.子女
EXG003.哪些子女
对35个子女循环
EXG004[i].占的百分比
i.是
!i.否
加载EXG002
!6.孙/外孙子女无产权
6.孙/外孙子女
EXG007.哪些子女的小孩
对35个子女循环
EXG008[i].孙/外孙子女占的比例
i.是
!i.否
EXG009.是否有现金
1.是
2.否
EXG010.现金金额
EXG011.是否有存款
1.是
2.否
EXG012.存款金额
EXG013.是否有金融资产
1.是
2.否
EXG014.金融资产数额
EXG015.是否有实物
1.是
2.否
EXG016.实物价值
EXG017.其他价值
EXG018.确认数额
EXG019.是否立过遗嘱
1.是
2.否
EXG020.遗嘱是否公正
EXG021.遗嘱中如何分配
加载EXG021
2.子女
!2.子女无分配
EXG022.哪些子女
对35个子女循环
EXG023[i].占的百分比
i.是
!i.否
加载EXG021
6.孙/外孙子女
!6.孙/外孙子女无分配
EXG026.哪些子女的小孩
对35个子女循环
EXG027[i].孙/外孙子女占的比例
i.是
!i.否
加载EXG021
3.兄弟姐妹
!3.兄弟姐妹无分配
EXG028.兄弟姐妹平均分配
EXG029.被继承的遗产
1.有
2.没有
加载EXG019
1.是
2.否
EXG030.是否按遗嘱分配
1.是
2.否
EXG031.遗产如何分配
加载EXG031
2.子女
!2.子女没有分配
EXG032.哪些子女
对35个子女循环
EXG033[i].占的百分比
i.是
!i.否
加载EXG031
6.孙/外孙子女
!6.孙/外孙子女没分配
EXG036.哪些子女的小孩
对35个子女循环
EXG037[i].孙/外孙子女占的比例
i.是
!i.否
结束

EXK. 殡葬

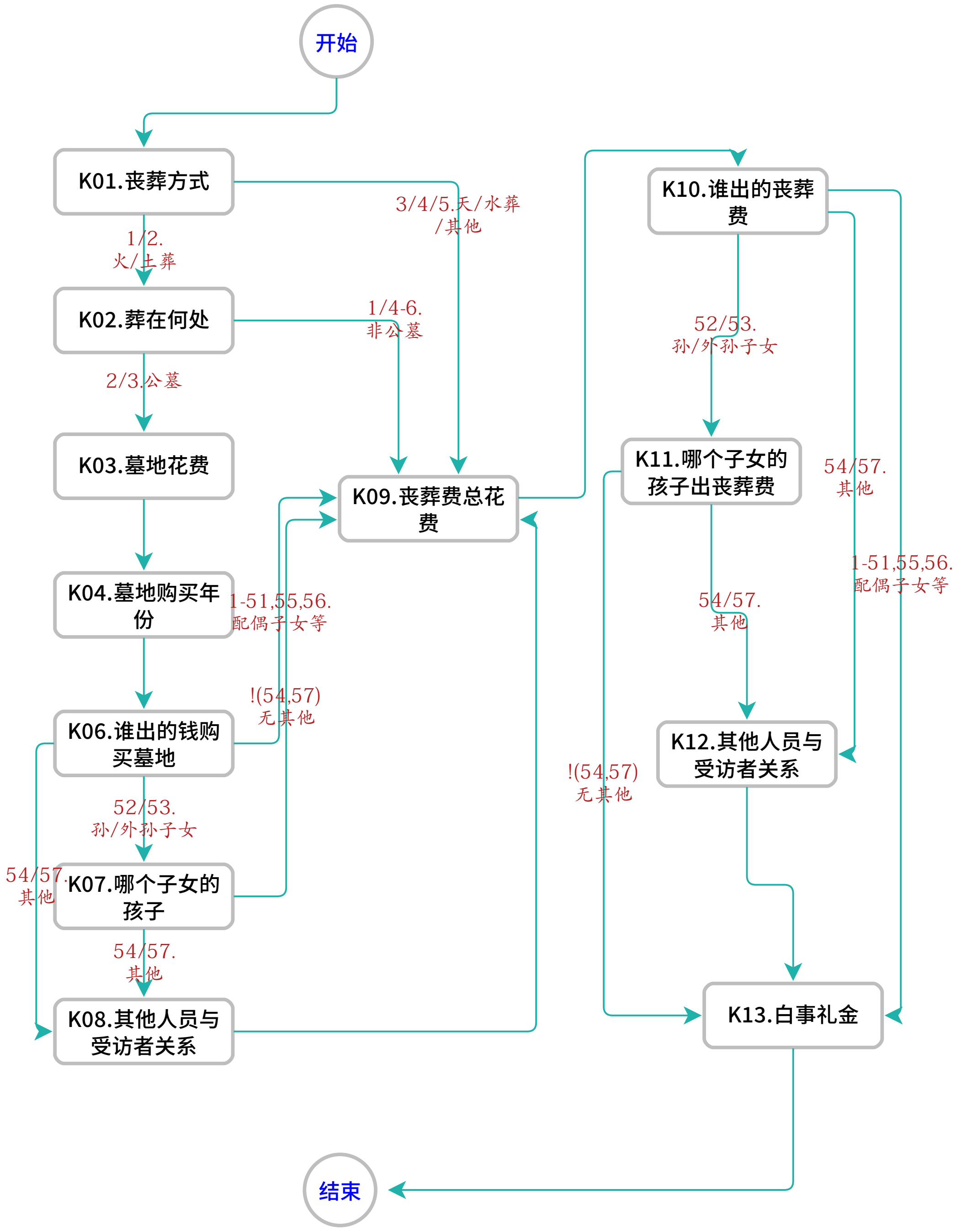
开始
K01.丧葬方式
1/2.
火/土葬
3/4/5.天/水葬
/其他
K02.葬在何处
1/4-6.
非公墓
2/3.公墓
K03.墓地花费
K04.墓地购买年份
1-51,55,56.
配偶子女等
!(54,57)
无其他
K06.谁出的钱购买墓地
52/53.
孙/外孙子女
54/57.
其他
K07.哪个子女的孩子
54/57.
其他
K08.其他人员与受访者关系
K09.丧葬费总花费
K10.谁出的丧葬费
52/53.
孙/外孙子女
54/57.
其他
K11.哪个子女的孩子出丧葬费
1-51,55,56.
配偶子女等
54/57.
其他
!(54,57)
无其他
K12.其他人员与受访者关系
K13.白事礼金
结束

EXV. 疫情相关

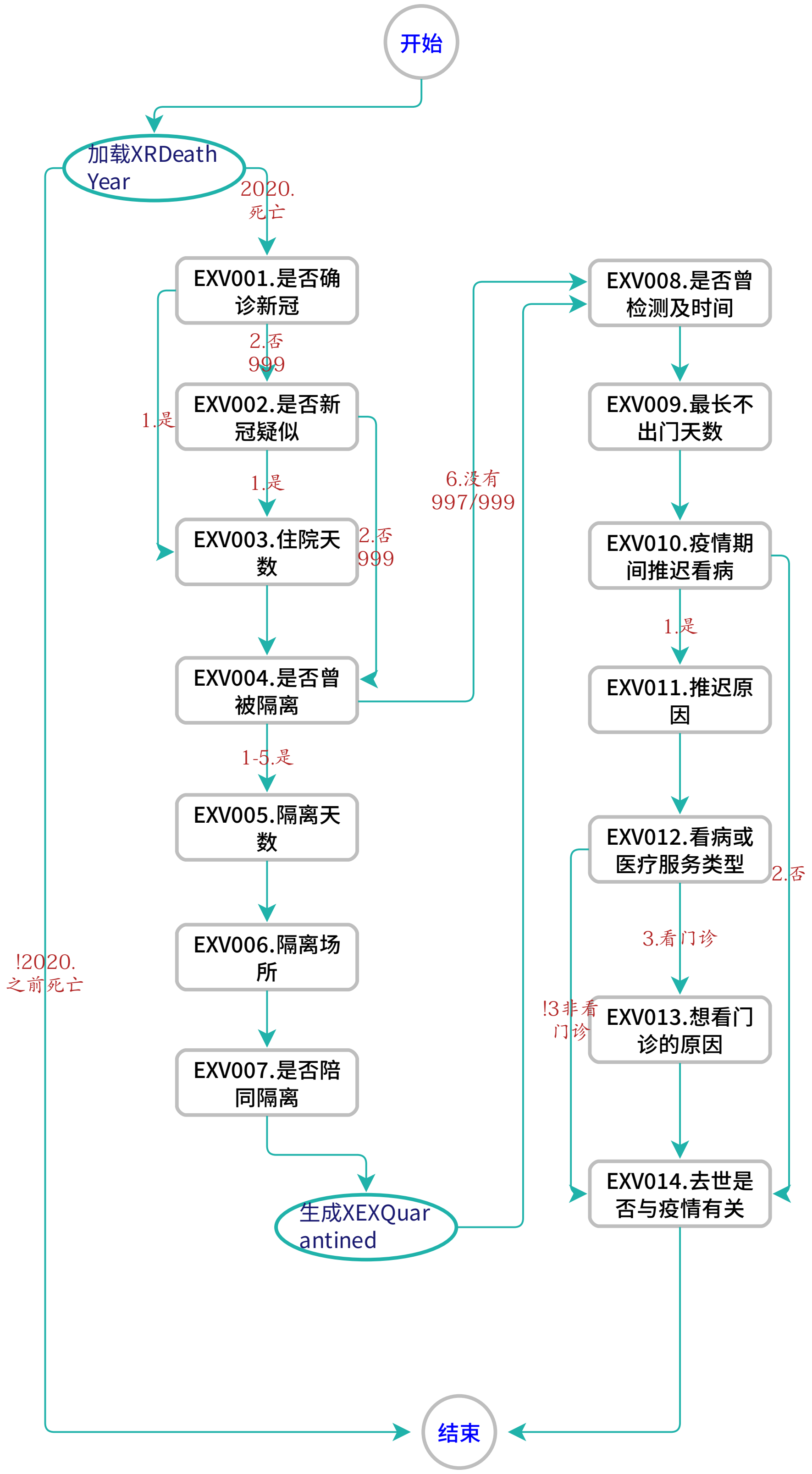
开始
加载XRDeath Year
2020.死亡
EXV001.是否确诊新冠
2.否 999
1.是
EXV002.是否新冠疑似
1.是
2.否 999
EXV003.住院天数
EXV004.是否曾被隔离
1-5.是
6.没有 997/999
EXV005.隔离天数
EXV006.隔离场所
EXV007.是否陪同隔离
生成XEXQuar antined
EXV008.是否曾检测及时间
EXV009.最长不出门天数
EXV010.疫情期间推迟看病
1.是
EXV011.推迟原因
EXV012.看病或医疗服务类型
2.否
3.看门诊
!3非看门诊
EXV013.想看门诊的原因
EXV014.去世是否与疫情有关
!2020.之前死亡
结束

VA. 死因分析

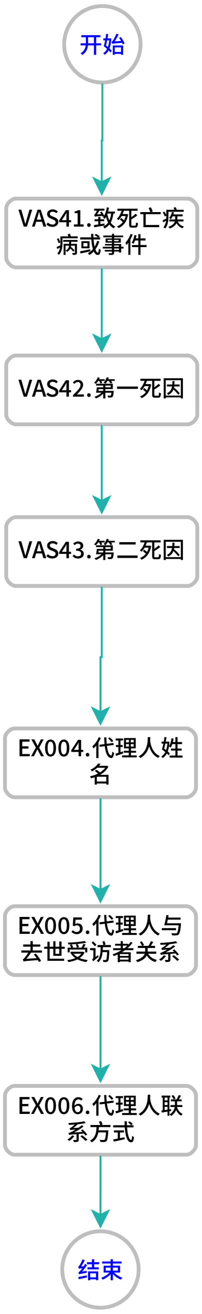
开始
VAS41.致死亡疾病或事件
VAS42.第一死因
VAS43.第二死因
EX004.代理人姓名
EX005.代理人与去世受访者关系
EX006.代理人联系方式
结束

This page intentionally left blank
