# Supplementary material for: Association of internet use and health service utilization with self-rated health in middle-aged and older adults: findings from a nationally representative longitudinal survey
Source: Front Public Health. 2024 Oct 3;12:1429983. doi: 10.3389/fpubh.2024.1429983 (PMC11483889; doi:10.3389/fpubh.2024.1429983)
Supplement: Supplementary file 7 [file Data_Sheet_7.PDF]

中国健康与养老追踪调查（CHARLS）

第五轮（2020）追访问卷

版本号: 20231106

2023 年 11 月

北京大学国家发展研究院

北京大学中国社会科学调查中心

目录

CV 过滤问卷 **1**

CV1. 双人或单人已访家户 2

CV2. 双人户婚姻及分户 2

CV3. 主要受访者婚姻 2

辅助变量定义 3

B 基本信息 **7**

B. 代理模式确认 8

BA. 基本信息 8

辅助变量定义 10

C 家庭信息 **13**

C. 代理模式确认 14

CA. 子女信息 14

CB. 家户成员信息 17

CC. 春节期间家庭联系 18

辅助变量定义 20

D 健康状况与功能 **25**

D. 代理模式确认 26

DA. 健康状况（一） 26

DA. 健康状况（二） 28

DA. 健康状况（三） 29

DB. 身体功能障碍以及辅助者（一） 32

DB. 身体功能障碍以及辅助者（二） 35

DB. 身体功能障碍以及辅助者（三） 36

DC. 认知和抑郁（一） 38

DC. 认知和抑郁（二） 40

辅助变量定义 45

F 工作与退休 **51**

F. 代理模式确认 52

FA. 工作概况（一） 52
FA. 工作概况（二） 53
FB. 农业自雇工作 54
FC. 受雇工作（一） 55
FC. 受雇工作（二） 57
FD. 非农自雇工作 59
FE. 非主要工作 60
FF. 求职与就业 60
FG. 疫情期间工作 61
FH. 退休手续 62
辅助变量定义 63

G 收入与支出 **67**
G1 家户收入与支出 68
G1. 代理模式确认 68
GB. 其他家户成员收入 68
GC. 家庭农业收入 69
GD. 个体经营和私营企业收入 71
GE. 家户公共转移支出收入 72
GF. 家户生活支出 74
I. 住房情况 77
G2 个人收入 81
G2. 代理模式确认 81
GA. 个人收入 81
辅助变量定义 83

V 疫情 **87**
V. 代理模式确认 88
VA. 疾病防范意识 88
VB. 个人患病和隔离 89
VC. 疫情期间个人活动 91
VD. 疫期居住地管控 94
辅助变量定义 95

EX 退出问卷 **97**
EXB. 基本信息 98
EXC. 家庭 100
EXD. 健康状况与功能（一） 101
EXD. 健康状况与功能（二） 104

EXD. 健康状况与功能（三） 107
EXE. 医疗保健与保险（一） 109
EXE. 医疗保健与保险（二） 111
EXF. 工作与退休 113
EXFN. 养老金 114
EXG. 收入、支出与资产 114
EXK. 殡葬 118
EXV. 疫情相关 119
VA. 死因分析 121
辅助变量定义 122

附录　函数说明 131

A. 题干中的函数 132
B. 辅助变量中的函数 132

This page intentionally left blank

CV　过滤问卷

CV1. 双人或单人已访家户

CV001 以下列表中，回答过滤问卷的是谁？

1. [ZRName1]
2. [ZRName2]
3. 代理人，姓名 ______ (**CV001_1**)，是 [ZRName1] 的什么人？ ______ (**CV001_2**)

CV002 [ZRName1] 是否健在？

【**访员注意**：如果回答过滤问卷的就是 [ZRName1]，无需提问该题，直接选择健在。用 4 位数表示年。】

1. 健在
2. 去世，去世时间是 ______ [$hc([2011,2020],int,\varnothing)$，$sc([ZIWYear,2020],int,\varnothing)$] (**CV002_1**) 年 ______ [$hc([1,12],int,-1)$] (**CV002_2**) 月 ______ [$hc([1,31],int,-1)$] (**CV002_3**) 日

CV003 [ZRName2] 是否健在？

【**访员注意**：如果回答过滤问卷的就是 [ZRName2]，无需提问该题，直接选择健在。用 4 位数表示年。】

1. 健在
2. 去世，去世时间是 ______ [$hc([2011,2020],int,\varnothing)$，$sc([ZIWYear,2020],int,\varnothing)$] (**CV003_1**) 年 ______ [$hc([1,12],int,-1)$] (**CV003_2**) 月 ______ [$hc([1,31],int,-1)$] (**CV003_3**) 日

CV2. 双人户婚姻及分户

CV004 [ZRName1] 与 [ZRName2] 目前是否仍然是夫妻或同居关系？

1. [ZRName1] 与 [ZRName2] 当前仍是夫妻或同居关系
2. [ZRName1] 与 [ZRName2] 已经离婚了
3. [ZRName1] 与 [ZRName2] 已经长期分居，且预期未来不会再作为配偶共同生活

CV005 对于 [ZRName1] 与 [ZRName2]，您更熟悉谁的情况？

1. [ZRName1]
2. [ZRName2]

CV3. 主要受访者婚姻

CV006 [XMainR] 当前的婚姻状态是？

1. 已婚并与配偶共同居住
2. 已婚，但因为工作等原因暂时没有跟配偶在一起居住
3. 分居，不再作为配偶共同生活
4. 离异
5. 丧偶
6. 从未结婚

CV007 [XMainR] 现在没有配偶，或者与已婚配偶分居且不再作为配偶共同生活，那么 [XMainR] 当前是否有伴侣以配偶身份共同生活？

1. 有，姓名 ______ (**CV007_1**)
2. 没有

CV008 [XMainR] 的配偶叫什么名字? ______

辅助变量定义

XRLive1 受访者 1 健在或去世情况

```
if (equal("CV002", "1")) {
    add("XRLive1", "1")
}
if (equal("CV002", "2")) {
    add("XRLive1", "2")
}
```

XRLive2 受访者 2 健在或去世情况

```
if (equal("CV003", "1")) {
    add("XRLive2", "1")
}
if (equal("CV003", "2")) {
    add("XRLive2", "2")
}
```

XBothAlive 受访者 1 和 2 健在或去世情况

```
if (equal("CV002","1") && (equal("CV003","1")) {
    add("XBothAlive", "1")
}
if (equal("CV002","2") && (equal("CV003","2")) {
    add("XBothAlive", "2")
}
if (equal("CV002","1") && (equal("CV003","2")) {
    add("XBothAlive", "3")
}
if (equal("CV002","2") && (equal("CV003","1")) {
    add("XBothAlive", "4")
}
```

XMaritalStatus 双人户婚姻

```
if (equal("CV004", "1")) {
    add("XMaritalStatus", "1")
}
if (equal("CV004", "2") || equal("CV004", "3")) {
    add("XMaritalStatus", "2")
}
```

XMainR 主要受访者姓名

```
if (equal("CV_HType", "1") && equal("XBothAlive", "3")) {
    add("XMainR", value("ZRName1"))
    add("XRType1", "1")
}
if (equal("CV_HType", "1") && equal("XBothAlive", "4")) {
    add("XMainR", value("ZRName2"))
    add("XRType2", "1")
}
if (equal("CV_HType", "2") && equal(" XRLive1", "1")) {
    add("XMainR", "ZRName1")
    add("XRType1", "1")
}

if (equal("XMaritalStatus", "1")) {
    add("XMainR", "ZRName1")
    add("XRType1", "1")
    add("XMainRS", "ZRName2")
    add("XRType2", "1")
}

if (((equal("CV004", "2") || equal("CV004", "3")) && equal("CV001", "1")) ||
    ((equal("CV004", "2") || equal("CV004", "3")) && equal("CV001", "3") && equal("CV005", "1"))) {
```

```
        add("XMainR", "ZRName1")
        add("XRType1", "1")
        add("XRSplited2", "1")
    } else {
        add("XRSplited2", "0")
    }

    if (((equal("CV004", "2") || equal("CV004", "3")) && equal("CV001", "2")) ||
        ((equal("CV004", "2") || equal("CV004", "3")) && equal("CV001", "3") && equal("CV005", "2"))) {
        add("XMainR", "ZRName2")
        add("XRType2", "1")
        add("XRSplited1", "1")
    } else {
        add("XRSplited1", "0")
    }
```

XMainRS 主要受访者的配偶姓名

```
see above
```

XRType1 受访者 1 类型

```
see above
```

XRType2 受访者 2 类型

```
see above
```

XRSplited1 受访者 1 的新家户

```
if (((equal("CV004", "2") || equal("CV004", "3")) && equal("CV001", "2")) || ((equal("CV004", "2") ||
↪   equal("CV004", "3")) && equal("CV001", "3") && equal("CV005", "2"))) {
    add("XMainR", "ZRName2")
    add("XRType2", "1")
    add("XRSplited1", "1")
} else {
    add("XRSplited1", "0")
}
```

XRSplited2 受访者 2 的新家户

```
if (((equal("CV004", "2") || equal("CV004", "3")) && equal("CV001", "1")) || ((equal("CV004", "2") ||
↪   equal("CV004", "3")) && equal("CV001", "3") && equal("CV005", "1"))) {
    add("XMainR", "ZRName1")
    add("XRType1", "1")
    add("XRSplited2", "1")
} else {
    add("XRSplited2", "0")
}
```

XRDeathYear1 受访者 1 去世年

```
add("XRDeathYear1", value("CV002_1"))
```

XRDeathMonth1 受访者 1 去世月

```
add("XRDeathMonth1", value("CV002_2"))
```

XRDeathDate1 受访者 1 去世日

```
add("XRDeathDate1", value("CV002_3"))
```

XRDeathYear2 受访者 2 去世年

```
add("XRDeathYear2", value("CV003_1"))
```

XRDeathMonth2 受访者 2 去世月

```
add("XRDeathMonth2", value("CV003_2"))
```

XRDeathDate2 受访者 2 去世日

```
add("XRDeathDate2", value("CV003_3"))
```

ZRName1 受访者 1 姓名

ZRName2 受访者 2 姓名

ZIWTime 上轮访问时间

This page intentionally left blank

B　基本信息

B. 代理模式确认

proxy_2 访员记录，对于基本信息模块是否使用代理问卷模式？

1. 是
2. 否

BA. 基本信息

BA001 访员记录 [XRName] 的性别

1. 男
2. 女

BA002 上次访问时我们记录的 [XRName] 的性别是 [XR18Gender]，这次你记录的性别是 [XR20Gender]，请再次确认 [XRName] 的性别

1. 男
2. 女

BA003 [XRName] 的真实、阳历出生日期是 ______ [hc([1900, 2000], *int*, ∅)] (**BA003_1**) 年 ______ [hc([1, 12], *int*, −1)] (**BA003_2**) 月 ______ [hc([1, 31], *int*, −1)] (**BA003_3**) 日

【**访员注意**：年份必填，用 4 位数表示年；月份和日期不知道或忘记可填“-1”。】

BA004 无需提问，请访员直接记录访问地址：

______ (**BA004_1**) 省/市/区县

______ (**BA004_2**) 乡/镇/街道/村/社区

______ (**BA004_3**) 小区/楼号/单元/门牌号

BA005 无需提问，请访员直接记录访问地类型。

1. 家庭住宅
2. 工作场所
3. 其它，请注明：______ (**BA005_1**)

BA006 [XRName] 现在住在哪里？不包括临时出差、旅游、临时一两天的探亲访友。

1. 访问地：[XRSurveyAdd]
2. 非访问地：中国大陆 ______ (**BA006_1**) 省/市/区县 ______ (**BA006_2**) 乡/镇/街道/村/社区 ______ (**BA006_3**) 小区/楼号/单元/门牌号
3. 中国香港
4. 中国澳门
5. 中国台湾
6. 国外：______ (**BA006_4**)

BA007 [XRName] 在居住地址 [XRResidenceFull] 居住的类型是？

1. 家庭住宅
2. 养老院或其他养老机构
3. 医院
4. 其它，请注明 ______ (**BA007_1**)

BA008 [XRResidenceQuestion] ?

1. 城或镇中心区
2. 城乡或镇乡结合区
3. 农村
4. 特殊区域

BA009 [XRName] 现在的户口类型是？
1. 农业
2. 非农业
3. 统一居民户口
4. 没有户口

BA010 [XRName] 现在获得的最高教育水平是什么（不包括成人教育）？
1. 未受过教育（文盲）
2. 未读完小学
3. 私塾毕业
4. 小学毕业
5. 初中毕业
6. 高中毕业
7. 中专（包括中等师范、职高）毕业
8. 大专毕业
9. 本科毕业
10. 硕士毕业
11. 博士毕业

BA010_1 [XRName] 是否识字？
1. 是
2. 否

BA011 [XRName] 目前的婚姻状态是？
1. 已婚与配偶一同居住
2. 已婚，但因为工作等原因暂时没有跟配偶在一起居住
3. 分居（不再作为配偶共同生活）
4. 离异
5. 丧偶
6. 从未结婚

BA012 [XRName] 现在是否有伴侣以配偶身份共同生活（同居）？
1. 有
2. 没有

BA013 过去一年，[XRName] 和他/她的配偶或伴侣共同居住了多长时间？ ______ [hc$([0,12], real, \varnothing)$] 个月

BA014 [XRName] 的社会养老保险多长时间发放一次？

【**访员注意**：若不知道发放频率，只知道领取频率，则记录最短多长时间领取一次，并备注是领取频率。】
1. 一个月
2. 一个季度
3. 半年

4. 一年
5. 超过一年，是 ______ [hc$((1,50), real, \varnothing)$] (**BA014_1**) 年
6. 参加了养老保险，但是还未到领取年龄
7. 没有参加任何养老保险

BA015 [XRName] 参保的是哪个社会养老保险?

【**访员注意**：若受访者不知道自己的参保类型，可以询问可能了解的子女、村干部等。】

1. 政府机关/事业单位养老保险
2. 企业职工养老保险
3. 城镇居民养老保险
4. 新型农村居民社会养老保险（新农保）
5. 城乡居民养老保险
6. 其它，请注明：______ (**BA015_1**)

BA016 [XRName] 的社会医疗保险一年缴费多少钱?

【**访员注意**：追问后，若受访者不知道社会医疗保险缴费金额，可填写"-1"。若每年缴费金额不同，记录最近一次缴费金额。】

1. 年缴费 ______ [hc$((0,100000], real, -1)$] (**BA016_1**) 元
2. 有医疗保险，但是不需要缴费
3. 没有参加任何医疗保险

BA017 [XRName] 参保的是哪个社会医疗保险?

【**访员注意**：若受访者不知道自己的参保类型，可以询问可能了解的子女、村干部等。】

1. 城镇职工医疗保险（医保）
2. 城乡居民医疗保险（合并城镇居民和新型农村合作医疗保险）
3. 城镇居民医疗保险
4. 新型农村合作医疗保险（合作医疗）
5. 公费医疗
6. 其它，请注明：______ (**BA017_1**)

BA018 今年上半年，[XRName] 有多少天是自己一个人住的? ______ [hc$([0,182], int, \varnothing)$]

BA019 今年上半年，[XRName] 有多少天只是和配偶/伴侣一起住（即除了配偶之外没有其他人一起住)? ______ [hc$([0,182], int, \varnothing)$]

BA020 今年上半年，[XRName] 有 [DayNumber] 天是没有和 [XRLiveCovid] 一起住的，这在多大程度上是因为新冠疫情造成的?（用百分数表示） ______ [hc$([0,100], int, \varnothing)$] %

【**访员注意**：本题不是提问疫情多大程度上影响了受访者独自居住或只与配偶居住的天数，而是问受访者有若干天是独自居住或只与配偶居住这个情况，在多大程度上是疫情造成的。】

辅助变量定义

XR20Gender 访员本次记录性别

```
if (equal("BA001", "1")) {
    add("XR20Gender", "男")
}
if (equal("BA001", "2")) {
    add("XR20Gender", "女")
}
```

XR18Gender 18 年调查记录性别

```
if (equal("ZRGender", "1")) {
    add("XR18Gender", "男")
}
if (equal("ZRGender", "2")) {
    add("XR18Gender", "女")
}
```

XRGender 性别（确认后）

```
if (!empty("ZRGender") && equal("BA001", value("ZRGender"))) {
    add("XRGender", value("BA001"))
}
if (!empty("ZRGender") && !equal("BA001", value("ZRGender"))) {
    add("XRGender", value("BA002"))
}
if (empty("ZRGender")) {
    add("XRGender", value("BA001"))
}
```

XRAge 年龄

```
if (empty("ZRBirthYear") || equal("XRType", "2")) {
    add("XRAge", 2020 - value("BA003_1"))
}
if (equal("XRType", "1") && !empty("ZRBirthYear")) {
    add("XRAge", 2020 - value("ZRBirthYear"))
}
```

XRResidenceFull 五级地址：省市县乡村、香港、澳门、台湾、国外

```
if (equal("BA006", "1")) {
    add("XRResidenceFull",value("BA004_1")+value("BA004_2"))
}
if (equal("BA006", "2")) {
    add("XRResidenceFull",value("BA006_1")+value("BA006_2"))
}
if (equal("BA006", "3")) {
    add("XRResidenceFull", "中国香港")
}
if (equal("BA006", "4")) {
    add("XRResidenceFull", "中国澳门")
}
if (equal("BA006", "5")) {
    add("XRResidenceFull", "中国台湾")
}
if (equal("BA006", "6")) {
    add("XRResidenceFull", "国外: "+value("BA006_4"))
}
```

XRResidenceCounty 前三级地址：省市县

```
if (equal("BA006", "1")) {
    add("XRResidenceCounty",value("BA004_1"))
}
if (equal("BA006", "2")) {
    add("XRResidenceCounty",value("BA006_1"))
}
```

XRResidenceCommunity 后三级地址：县乡村

```
if (equal("BA006", "1")) {
    add("XRResidenceCommunity",value("BA004_2"))
}
if (equal("BA006", "2")) {
    add("XRResidenceCommunity",value("BA006_2"))
}
```

XRSurveyAdd 访问地址（包括门牌号）

```
add("XRSurveyAdd", value("BA004_1")+value("BA004_2")+value("BA004_3"))
```

XRResidenceSurveyHome 是否现住地就是访问地且是家庭住宅

```
if (equal("BA006", "1") && equal("BA005", "1")) {
    add("XRResidenceSurveyHome", "1")
}
if ((equal("BA006", "1") && !equal("BA005", "1")) || equal("BA006", "2") || equal("BA006", "3") ||
↪   equal("BA006", "4") || equal("BA006", "5") || equal("BA006", "6") ) {
    add("XRResidenceSurveyHome", "2")
}
```

XRResidenceQuestion da008（一般居住地城乡类别）题干不同问法

```
if (equal("BA006", "1")) {
    add("XRResidenceQuestion", "请访问员自行填写"+value("XRSurveyAddFull")+"是农村还是城市")
}
if (equal("BA006", "2")) {
    add("XRResidenceQuestion",
    ↪   value("XRName")+"在居住地"+value("XROtherInlandFull")+"居住时主要生活在农村还是城市")
}
```

XRSurveyAddFull 调查地址：五级地址（不包括门牌号）

```
add("XRSurveyAddFull", value("BA004_1")+value("BA004_2"))
```

XROtherInlandFull 现在住址：非访问地，中国大陆其他地址五级地址（不包括门牌号）

```
if (equal("BA006", "2")) {
    add("XROtherInlandFull", value("BA006_1")+value("BA006_2"))
}
```

XRPartner 是否有配偶/伴侣

```
if ((equal("BA011", "1") || equal("BA011", "2")) || ((equal("BA011", "3") || equal("BA011", "4") ||
↪   equal("BA011", "5") ||
    equal("BA011", "6")) && equal("BA012", "1"))) {
    add("XRPartner", "1")
}
if ((equal("BA011", "3") || equal("BA011", "4") || equal("BA011", "5") || equal("BA011", "6")) &&
↪   equal("BA012", "2")) {
    add("XRPartner", "2")
}
```

XRLiveCovid 不与其他人共同居住，多大程度是因疫情导致的，题干问法

```
if (equal("XRPartner", "1")) {
    add("XRLiveCovid", "除配偶/伴侣之外的其他任何人")
}
if (equal("XRPartner", "2")) {
    add("XRLiveCovid", "其他任何人")
}
```

DayNumber 没有和（除配偶/伴侣之外的）其他任何人一起住的天数

```
if (equal("XRPartner", "1")) {
    add("DayNumber", value("BA018")+value("BA019"))
}
if (equal("XRPartner", "2")) {
    add("DayNumber", value("BA018"))
}
```

XRName 受访者姓名

C　家庭信息

C. 代理模式确认

proxy_3 访员记录，对于工作模块是否使用代理问卷模式?

1. 是
2. 否

CA. 子女信息

CA001 接下来的我们会问到一些关于 [XMainR] 和 [XMainRS] 家庭成员的问题，包括子女、家户成员等等，请问 [XMainR] 和 [XMainRS] 谁更了解这方面的情况?

1. [XMainR]
2. [XMainRS]

【引语：下面我想问一些关于 [XFamilyR] 子女的问题】

CA002[i] 现在 [ZChildName[i]] （性别：[XChildGenderDis[i]]）还健在吗?

1. 是
2. 否

CA003[i] [ZChildName[i]] 是什么时候去世的? ______ [hc([2011, 2020], int, ∅)，sc([ZIWYear, 2020], int, ∅)] (**CA003_1[i]**) 年 ______ [hc([1, 12], int, ∅)] (**CA003_2[i]**) 月 ______ [hc([1, 31], int, −1)] (**CA003_3[i]**) 日

【**访员注意**：去世的年份和月份为必填项，用于生成问卷的逻辑。对于去世的具体日子，如果受访者拒绝回答或者忘记了，请填入"-1"】

CA004[i] [ZChildName[i]] 去世的主要原因是什么? ______

【**访员注意**：如果是因疾病死亡，请详细写明病因，例如因癌症去世，要注明是哪种类型的癌症（胃癌、肺癌等）；如果因传染病去世，要写明传染病的类型（结核病、痢疾）；如果是因为事故死亡，要写明事故类型，如车祸、火灾、意外中毒等】

CA005[i] [XChildPanAliveName[i]] 是什么时候出生的? ______ [hc([1910,ZIWYear], int, ∅)，sc([1940, ZIWYear], int, ∅)] 年

【**访员注意**：用 4 位数表示年，如果受访者记不得出生年份，可以通过子女今年多大，或者哪年去世的，去世时多大以及子女出生时受访者多大等信息推算出生年份。】

CA006[i] [XChildPanAliveName[i]] 的性别是?

1. 男
2. 女

CA007[i] 不包括成人教育，[XChildPanAliveName[i]] 的最高学历是?

1. 未受过正规教育
2. 未读完小学
3. 私塾
4. 小学毕业
5. 初中毕业
6. 高中毕业
7. 中专（包括中等师范、职高）毕业

8. 大专毕业
9. 本科毕业
10. 硕士毕业
11. 博士毕业
997. 不知道
999. 拒绝回答

CA008[*i*] [XChildAliveName[*i*]] 现在是在工作还是在上学？工作包括务农、挣工资的工作、从事个体、私营活动或不拿工资为家庭经营活动帮工
1. 工作
2. 上学
3. 边工作边上学
4. 既不工作也不上学，请注明现在主要做什么 ______ (**CA008_1[i]**)
997. 不知道
999. 拒绝回答

CA009[*i*] [XChildAliveName[*i*]] 这份工作属于什么职业？也就是说，[XChildAliveName[*i*]] 的工作现在具体做些什么？ ______ （例 1：餐厅面点厨师；例 2：生产线绕线工人）

CA010[*i*] [XChildPanAliveName[*i*]] 目前的婚姻状况是？
1. 已婚并与配偶一同居住
2. 已婚，但因为工作等原因暂时没有跟配偶在一起居住
3. 分居（不再作为配偶共同生活）
4. 离异
5. 丧偶
6. 从未结婚
997. 不知道
999. 拒绝回答

CA012[*i*] 过去一年，[XChildCoupleDis[*i*]] 去年的总收入属于下面哪一类？
1. 0 没有收入
2. 少于 2 千元
3. 2 千与 5 千之间
4. 5 千与 1 万之间
5. 1 万与 2 万之间
6. 2 万与 3 万之间
7. 3 万与 5 万之间
8. 5 万与 10 万之间
9. 10 万与 15 万之间
10. 15 万与 20 万之间
11. 20 万与 30 万之间
12. 多于 30 万
997. 不知道
999. 拒绝回答

CA013[*i*] 请问 [XChildPanAliveName[*i*]] 现在的身体情况怎么样？是很好，好，一般，不好还是很不好？

1. 很好
2. 好
3. 一般
4. 不好
5. 很不好
997. 不知道
999. 拒绝回答

【引语：接下来我们将询问 [XFamilyR] 与子女间的交往以及相互之间的经济帮助。】

CA014[*i*] 过去一年，[XChildPanAliveName[*i*]] 与 [XFamilyRAndS] 一起居住了多长时间？______ [hc ($[0, 12], real, \varnothing$)] 月

【访员注意：短暂的走亲戚不算一起居住；没有一起居住请填写 0；一直住在一起请填写 12】

CA015[*i*] [XFamilyRAndS] 和 [XChildPanAliveName[*i*]] 不在一起住的时候，[XFamilyRAndS] 多长时间能见到 [XChildPanAliveName[*i*]] 一次？

1. 差不多每天
2. 每周 2-3 次
3. 每周一次
4. 每半个月一次
5. 每月一次
6. 每三个月一次
7. 半年一次
8. 每年一次
9. 几乎从来没有
10. 其他

CA016[*i*] [XFamilyRAndS] 和 [XChildPanAliveName[*i*]] 不在一起住的时候，[XFamilyRAndS] 多长时间跟 [XChildPanAliveName[*i*]] 通过电话、短信、微信、信件或者电子邮件联系一次？

1. 差不多每天
2. 每周 2-3 次
3. 每周一次
4. 每半个月一次
5. 每月一次
6. 每三个月一次
7. 半年一次
8. 每年一次
9. 几乎从来没有
10. 其他

【引语：有时候，家庭之间会有多种形式的互相帮助，而每一种形式的帮助都很重要。所以，接下来我们想了解一下，[XFamilyRAndS] 有没有从子女那里得到或者给子女什么经济帮助】

CA017[*i*] 过去一年，在 [XChildPanAliveName[*i*]] 不和 [XFamilyR] 住在一起时，[XFamilyRAndS] 从 [XChildPanAliveName[*i*]] 得到多少经济支持？

总共给钱 ______ [hc($[0, \infty), int, -1$)，sc($[0, 500000], int, -1$)，ub($[-1], [100, 500, 1500, 5000, 20000]$)] (**CA017_1[i]**) 元，其中，定期给钱共计 ______ [hc($[0, \infty), int, -1$)，sc($[0, 500000], int, -1$)，ub($[-1], [100, 200, 1000, 3000, 10000]$)] (**CA017_2[i]**) 元（如定期提供生活费、支付每月水电费及

电话费、支付房贷、房租费用或其他定期的费用)；
总共给物 ______ [hc$([0,\infty), int, -1)$，sc$([0,500000], int, -1)$，ub$([-1],[100,500,1500,5000,20000])$] (**CA017_3[i]**) 元，其中，定期给物共计 ______ [hc$([0,\infty), int, -1)$，sc$([0,500000], int, -1)$，ub$([-1],[100,200,1000,3000,10000])$] (**CA017_4[i]**) 元（如定期提供粮食、买菜、买衣服或其他物品等)。
【**访员注意**：包括从 [XChildPanAliveName[i]] 的孩子那里收到的经济支持。
定期是指按月、按季度、按半年给钱或东西，时间上大致固定；
定期给的金额不应超过总共给的金额。
没有给钱或物，请填入“0”；如果受访者拒绝回答或者忘记了，请填入“-1”】

CA018[i] 过去一年，在 [XChildPanAliveName[i]] 不和 [XFamilyR] 住在一起时，[XFamilyRAndS] 给 [XChildPanAliveName[i]] 多少经济支持？
总共给钱 ______ [hc$([0,\infty), int, -1)$，sc$([0,500000], int, -1)$，ub$([-1],[100,500,1500,5000,20000])$] (**CA018_1[i]**) 元，其中，定期给钱共计 ______ [hc$([0,\infty), int, -1)$，sc$([0,500000], int, -1)$，ub$([-1],[100,200,1000,3000,10000])$] (**CA018_2[i]**) 元（如定期提供生活费、支付每月水电费及电话费、支付房贷、房租费用或其他定期的费用)；
总共给物 ______ [hc$([0,\infty), int, -1)$，sc$([0,500000], int, -1)$，ub$([-1],[100,500,1500,5000,20000])$] (**CA018_3[i]**) 元，其中，定期给物共计 ______ [hc$([0,\infty), int, -1)$，sc$([0,500000], int, -1)$，ub$([-1],[100,200,1000,3000,10000])$] (**CA018_4[i]**) 元（如定期提供零食、买菜、买衣服或其他物品等)。
【**访员注意**：包括给 [XChildPanAliveName[i]] 的孩子的经济支持。
定期是指按月、按季度、按半年给钱或东西，时间上大致固定；
定期给的金额不应超过总共给的金额。
没有给钱或物，请填入“0”；如果受访者拒绝回答或者忘记了，请填入“-1”】

CA019[i] 今年爆发的新冠疫情是否影响到了 [XFamilyRAndS] 与 [XChildPanAliveName[i]] 之间的关系？
1. 变好了
2. 变差了
3. 没影响

CA020[i] 春节后的疫情是否对 [XChildCoupleDis[i]] 的收入造成了影响？是增加、减少，还是没影响？
【**访员注意**：如果受访者拒绝回答或者忘记了，请填入“-1”
在条件允许的情况下，可以向子女了解疫情对收入造成的影响】
1. 增加，估计增加了百分之 ______ [hc$((0,\infty), int, -1)$，sc$((0,100], int, -1)$] (**CA020_1[i]**)
2. 减少，估计减少了百分之 ______ [hc$((0,100], int, -1)$] (**CA020_2[i]**)
3. 没影响
997. 不知道
999. 拒绝回答

CB. 家户成员信息

【**引语**：我们想了解一下，除了 [XFamilyRAndS] 外的其他家户成员的情况，家户成员是指和 [XFamilyRAndS] 一同居住，且共享生活收支的人】

CB001 以下这些人哪些是 [XFamilyRAndS] 的家户成员？（可多选）

【**访员注意**：家户成员是指和受访者共同居住且共享生活收支的人，在实地比较难以判断的是子女是否是受访者的家户成员，一般可以通过询问受访者是否与某个子女分家等方式来确定】

1-25. [XChildAliveName[i]]

99. 以上都不是

[conflict(99, $[99]^c$)]

【引语：[XIntroCB002]】

CB002 [XHHOtherDis] 哪些人是 [XFamilyRAndS] 的家户成员？请勾选并填入姓名

1-10. ______ (**CB002_1[i]**)

99. 没有其他家户成员

[conflict(99, $[99]^c$)]

CB003[i] 家户成员 [XHHOtherMemberName[i]] 的性别是？

1. 男性
2. 女性

CB004[i] 家户成员 [XHHOtherMemberName[i]] 今年多大？ ______ [hc($[0, 120], int, -1$), sc($[0, 100], int, -1$)] 岁

【**访员注意**：如果受访者拒绝回答或者忘记了，请填入“-1”】

CB005[i] [XHHOtherMemberName[i]] 是 [XFamilyR] 的什么人？

1. 儿媳或女婿
2. 孙子或孙女
3. 姐夫妹夫或嫂子弟媳
4. 父亲
5. 母亲
6. 岳母/婆婆
7. 岳父/公公
8. 子女
9. 兄弟姐妹
10. 其他亲戚，请注明：______ (**CB005_1[i]**)

CB006[i] [XHHOtherMemberName[i]] 是 [XFamilyR] 哪个孩子的配偶？

【**访员注意**：此处加载健在子女的名单。如果该家户成员是已经去世子女的配偶，请在文字框中添加该去世子女的姓名】

1-25. [XChildAliveName[i]]

99. 其他，姓名：______ (**CB006_1[i]**)

CB007[i] [XHHOtherMemberName[i]] 是 [XFamilyR] 哪个子女的孩子？

【**访员注意**：此处加载健在子女的名单。如果该家户成员是已经去世子女的孩子，请在文字框中添加该去世子女的姓名】

1-25. [XChildAliveName[i]]

99. 其他，姓名：______ (**CB007_1[i]**)

CC. 春节期间家庭联系

【引语：下面几个问题是关于春节期间 [XFamilyR] 一家团聚受疫情影响的情况。】

CC001 今年春节期间，[XFamilyR] 的孩子或者孙子女是否因疫情原因没法回家过年？

1. 是，有 ______ [hc($[1,15], int, \emptyset$)，sc($[1,8], int, \emptyset$)] **(CC001_1)** 个子女及孙子女没法回家过年
2. 否

CC002 今年春节期间，是否有亲戚朋友因为疫情原因没法来看望 [XFamilyR]？

1. 是
2. 否

CC003 和往年春节相比，晚辈和亲戚朋友给 [XFamilyRAndS] 的钱是否减少了？

1. 是，大约 ______ [hc($(0,100000], int, \emptyset$)，sc($(0,50000], int, \emptyset$)] **(CC003_1)** 元
2. 否

CC004 春节期间，通常长辈会给晚辈发红包。那么今年春节 [XFamilyRAndS] 发红包是否省下了一些钱？

1. 是，大约 ______ [hc($(0,100000], int, \emptyset$)，sc($(0,50000], int, \emptyset$)] **(CC004_1)** 元
2. 否

CC005 今年正月里，[XFamilyRAndS] 是否大部分时间住在一起？

1. 是
2. 否

CC006[i] [XCouLiveList[i]] 在今年正月里都和哪些人一起居住？（可多选）

【访员注意：包括短时间的居住】

1-25. [XChildPanAliveName[i]]
26-35. [XHHOtherMemPreload[i]]
99. 以上都没有

[conflict($99, [99]^c$)]

CC007[i] 除了刚才提到的人，还有哪些人今年正月里和 [XCouLiveList[i]] 一起居住？（可多选）

【访员注意：包括短时间的居住】

1. ______ **(CC007_1[i])**
2. ______ **(CC007_2[i])**
3. ______ **(CC007_3[i])**
4. ______ **(CC007_4[i])**
5. ______ **(CC007_5[i])**
6. ______ **(CC007_6[i])**
7. ______ **(CC007_7[i])**
8. ______ **(CC007_8[i])**
9. ______ **(CC007_9[i])**
10. ______ **(CC007_10[i])**
99. 没有其他人

[conflict($99, [99]^c$)]

CC008[i] 与往年相比，今年正月里与 [XCouLiveList[i]] 共同居住的人是增加了还是减少了？

1. 增加了

2. 减少了
3. 没变化

辅助变量定义

XFamilyR 家庭受访者姓名

```
if (!empty("XMainRS")) {
    if (equal("CA001", "1")) {
        add("XFamilyR", value("XMainR"))
        add("XFamilyS", value("XMainRS"))
    } else if (equal("CA001", "2")) {
        add("XFamilyR", value("XMainRS"))
          add("XFamilyS", value("XMainR"))
    }
} else {
    add("XFamilyR", value("XMainR"))
    add("XFamilyS", "")
}
```

XFamilyS 家庭受访者配偶姓名

see above

XFamilyRAndS 题干中一起加载家庭受访者与配偶姓名。如果配偶存在显示为“[家庭受访者姓名] 和 [配偶姓名]”，否则只显示家庭受访者姓名。

```
if (!empty("XFamilyS")) {
    add("XFamilyRAndS",
    value("XFamilyR")+"和"+value("XFamilyS"))
} else {
    add("XFamilyRAndS", value("XFamilyR"))
}
```

XChildNum 姓名不为空的子女数

```
add("XChildNum", "0")
for (var i = 1; i <= value("ZChildNum"); i++) {
    if (!empty("ZChildName[i]")) {
        add("XChildNum", value("XChildNum")+1)
    }
}
```

XChildGenderDis 题干中显示加载的性别

```
if (equal("ZChildGender[i]", "1")) {
    add("XChildGenderDis[i]", "男性")
} else if (equal("ZChildGender[i]", "2")) {
    add("XChildGenderDis[i]", "女性")
} else {
    add("XChildGenderDis[i]", "缺失")
}
```

XChildAlive 访问时子女是否健在

```
if (equal("CA002[i]", "1")) {
    add("XChildAlive[i]", "1")
} else if (equal("CA002[i]", "2")) {
    add("XChildAlive[i]", "0")
}
```

XChildAliveName 访问时健在子女的姓名，去世的为空

```
if (equal("XChildAlive[i]", "1")) {
    add("XChildAliveName[i]", value("ZChildName[i]"))
}
```

XChildAliveNum 健在子女的数目

```
add("XChildAliveNum", "0")
for (var i = 1; i <= value("ZChildNum"); i++) {
    if (!empty("ZChildName[i]") && equal("XChildAlive[i]", "1")) {
        add("XChildAliveNum", value("XChildAliveNum")+1)
    }
}
```

XChildPanAliveName 疫情开始时健在子女姓名，去世的为空

```
if (equal("XChildPanAlive[i]", "1")) {
    add("XChildPanAliveName[i]", value("ZChildName[i]"))
    add("XChildPanAliveNum", value("XChildPanAliveNum")+1)
}
```

XChildPanAliveNum 疫情开始时健在子女数量

```
see above
```

XChildPanAlive 疫情开始时子女是否健在

```
if (equal("XChildAlive[i]", "1")) {
    add("XChildPanAlive[i]", "1")
} else if (greater("2019", "CA003_1[i]", true)) || (equal("CA003_1[i]", "2020") && equal("CA003_2[i]",
↪   "1")) {
    add("XChildPanAlive[i]", "0")
} else if (equal("CA003_1[i]", "2020") && greater("CA003_2[i]", "1", false)) {
    add("XChildPanAlive[i]", "1")
}
```

XChildGender 子女性别

```
if (!empty("ZChildGender[i]")) {
    add("XChildGender[i]", value("ZChildGender[i]"))
} else {
    add("XChildGender[i]", value("CA006[i]"))
}
```

XChildBirth 子女出生年份

```
if (!empty("ZChildBirth[i]")) {
    add("XChildBirth[i]", value("ZChildBirth[i]"))
} else {
    add("XChildBirth[i]", value("CA005[i]"))
}
```

XChildEdu 子女最高教育程度

```
if (!empty("ZChildEdu[i]")) {
    add("XChildEdu[i]", value("ZChildEdu[i]"))
} else {
    add("XChildEdu[i]", value("CA007[i]"))
}
```

XChildCoupleDis 如果存在配偶，则显示“[子女姓名] 和他/她的配偶”

```
if (equal("CA010[i]", "1") || equal("CA010[i]", "2")) {
    add("XChildCoupleDis[i]", value("XChildPanAliveName[i]")+"和他/她的配偶")
} else if (equal("CA010[i]", "3") || equal("CA010[i]", "4") || equal("CA010[i]", "5") || equal("CA010[i]",
↪   "6") || equal("CA010[i]", "997") || equal("CA010[i]", "999")) {
    add("XChildCoupleDis[i]", value("XChildPanAliveName[i]"))
}
```

XIntroCB002 如果访问时健在子女为 0，则在 CB002 显示引语

```
if (greater("XChildAliveNum", "0")) {
    add("XIntroCB002", "")
} else {
    add("XIntroCB002", "我们想了解一下，除了"+pre("XFamilyRAndS")+"外的其他家户成员的情况，
    ↪   家户成员是指和"+pre("XFamilyRAndS")+"一同居住，且共享生活收支的人")
}
```

XHHOtherDis 如果需要询问子女是否为家户成员，则显示 “除了刚刚选择的家户成员以外，还有”

```
if (equal("XChildAliveNum", "0")) {
    add("XHHOtherDis", "")
} else if (greater("XChildAliveNum", "0")) {
    add("XHHOtherDis", "除了刚刚选择的家户成员以外，还有")
}
```

XHHMemberNum 家户成员数，最小为 0，最大为 25

```
for (var i1 = 1; i1 <=25 ; i1++) {
    if (selected("CB001", i1)) {
        add("XHHMemberNum", value("XHHMemberNum")+1)
        add("XHHMemberName["+value("XHHMemberNum")+"]", value("XChildAliveName["+i1+"]"))
        add("XHHMemberAge["+value("XHHMemberNum")+"]", 2020-value("XChildBirth["+i1+"]"))
    }
}
for (var i1 = 1; i1 <=10 ; i1++) {
    if (selected("CB002", i1)) {
            add("XHHMemberNum", value("XHHMemberNum")+1)
            add("XHHMemberName["+value("XHHMemberNum")+"]", value("CB002_1[i1]"))
    }
}
```

XHHMemberName 家户成员姓名

see above

XHHMemberAge 家户成员年龄

```
for (var i1 = 1; i1 <=25 ; i1++) {
    if (selected("CB001", i1)) {
        add("XHHMemberAge["+value("XHHMemberNum")+"]", 2020-value("XChildBirth["+i1+"]"))
    }
}
if (!equal("CB004[i]", "-1")) {
    add("XHHOtherAgeIter", value("XHHOtherAgeIter")+1)
    add("XHHMemberAge["+value("XHHOtherAgeIter")+"]", value("CB004[i]"))
} else {
    add("XHHOtherAgeIter", value("XHHOtherAgeIter")+1)
    add("XHHMemberAge["+value("XHHOtherAgeIter")+"]", "")
}
```

XHHOtherMemberNum 其他家户成员数，最大为 10

```
add("XHHOtherMemberNum", "0")
for (var i1 = 1; i1 <=10 ; i1++) {
    if (selected("CB002", i1)) {
           add("XHHOtherMemberNum", value("XHHOtherMemberNum")+1)
           add("XHHOtherMemberName["+value("XHHOtherMemberNum")+"]", value("CB002_1[i1]"))
    }
}
```

XHHOtherMemberName 其他家户成员姓名

see above

XHHOtherAgeIter 计算家户成员年龄的中间变量

```
if (equal("XChildAliveNum", "0")) {
    add("XHHOtherAgeIter", "0")
} else if (selected("CB001", 99)) {
    add("XHHOtherAgeIter", "0")
} else {
    add("XHHOtherAgeIter", count("CB001"))
}
```

XAffectReunion 家庭春节团聚是否受到疫情影响

```
if (equal("CC001", "1") || equal("CC002", "1")) {
    add("XAffectReunion", "1")
} else {
```

```
    add("XAffectReunion", "0")
}
```

XCouLiveList 根据家庭受访者与配偶是否一起居住，生成一起或单独询问的姓名列表

```
if (empty("XFamilyS")) {
    add("XCouLiveList[1]", value("XFamilyR"))
} else if (!empty("XFamilyS") && equal("CC005", "2")) {
    add("XCouLiveList[1]", value("XFamilyR"))
    add("XCouLiveList[2]", value("XFamilyS"))
} else {
    add("XCouLiveList[1]", value("XFamilyRAndS"))
}
```

XHHOtherMemPreload 生成自 i=26 开始的其他家户成员列表

```
for (var k = 1; k <= 10; k++) {
    add("XHHOtherMemPreload["+(k+25)+"]", value("XHHOtherMemberName[k]"))
}
```

ZChildName 子女姓名

D　健康状况与功能

D. 代理模式确认

proxy_5 访员记录，对于健康模块是否使用代理问卷模式?

1. 是
2. 否

DA. 健康状况（一）

【引语：下面我将问到一些关于 [XRName] 的健康状况的问题。】

DA001 您认为您的健康状况怎样？是很好，好，一般，不好，还是很不好?

【访员注意：必须读出所有的选项】

1. 很好
2. 好
3. 一般
4. 不好
5. 很不好

997. 不知道

DA002[*i*] 与 [ZIWTime] 相比，[XRName] 的 [XChroDisType[*i*]] 好一些了，跟原来差不多还是更差?

1. 更好
2. 更差
3. 与原来差不多

99. 上次访问未患有此项疾病

DA002_1[*i*] [ZIWTime] 时，[XRName] 知道自己患有 [XChroDisType[*i*]]。与 [ZIWTime] 相比，[XRName] 的 [XChroDisType[*i*]] 好一些了，跟原来差不多还是更差?

1. 更好
2. 更差
3. 与原来差不多

99. 上次访问未患有此项疾病

DA003[*i*] 是否有医生曾经告诉过 [XRName] 有 [XChroDisType[*i*]]？

1. 是
2. 否

DA004[*i*] [XRName] 是否知道自己患有 [XChroDisType[*i*]]？

1. 知道自己患有
2. 知道自己没有
3. 不知道有没有

DA005 过去一个月里，[XRName] 是否去医疗机构看过门诊或者接受过上门医疗服务？(不包括去医院做体检)

1. 是
2. 否

DA006 过去一个月中，[XRName] 去医疗机构看过几次门诊（包括上门医疗服务）？ ______ [hc((0, 99), *int*, ∅)，sc([1, 20), *int*, ∅)] 次

DA007 过去一年内，[XRName] 住过院吗？

【**访员注意**：过去一年指从今天往前数的一年之内】

1. 是
2. 否

DA008 过去一年，[XRName] 接受过几次住院治疗？ ______ [hc((0, 99), *int*, ∅)，sc([1, 20), *int*, ∅)] 次

【**访员注意**：不在过去一年内发生的住院不计，过去一年指从今天往前数的一年之内】

DA009 疫情期间，[XRName] 是否曾经需要去看病，包括去看牙，但受疫情影响，被迫推迟，或没能够去看？

1. 是
2. 否

DA010 可以具体说明一下疫情期间 [XRName] 是想去看什么病或者想得到什么医疗服务，结果被推迟或取消了吗？（可多选）

【**访员注意**：请读出每一个选项，勾选所有符合的选项】

1. 需要住院的大的手术
2. 门诊或者日间病房就可以做的小的手术
3. 去看医生门诊
4. 去拿处方药
5. 去看牙，口腔治疗
6. 其他，请简单说明 ______ (**DA010_1**)

DA011 [XRName] 疫情期间想去看门诊，是因为新出现的症状或疾病，或者是因为治疗已经得了的疾病，还是常规的体检筛查？（可多选）

1. 新出现的症状或疾病
2. 治疗已经得了的疾病
3. 常规的体检筛查

DA012 为什么 [XRName] 看病被推迟，或者没能够去看呢？（可多选，选项免读）

1. 没法预约，或医院的常规挂号都取消了
2. 医院把常规的诊疗安排都改期了
3. 我自己决定可以等等再去看
4. 我害怕去医院
5. 其他，请说明 ______ (**DA012_1**)

DA013 自 [ZIWTime] 以来，[XRName] 最近一次常规体检时什么时候？（注意: 不包括 CHARLS 体检和去医疗机构看病所做的检查）

【**访员注意**：用 4 位数表示年，按照实际的月份填写月。例：1 月写作“1”，而不是“01”，12 月写作“12”。如果记不住月份，请填入“-1”】

1. ______ [hc([1900, 2020], *int*, ∅)，sc([1920, 2020], *int*, ∅)] (**DA013_1**) 年 ______ [hc([1, 12], *int*, −1)] (**DA013_2**) 月
2. 自上次访问以来没有参加过常规体检

DA014 [XRName] 最近一次常规体检时什么时候（注意: 不包括去医疗机构看病所做的检查）？

【**访员注意**：用 4 位数表示年，按照实际的月份填写月。例：1 月写作“1”，而不是“01”，12 月写作“12”。如果记不住月份，请填入“-1”】

1. ______ [hc($[1900, 2020], int, \varnothing$)，sc($[1920, 2020], int, \varnothing$)] **(DA014_1)** 年 ______ [hc($[1, 12], int, -1$)] **(DA014_2)** 月
2. 一辈子没有参加过常规体检

DA. 健康状况（二）

DA019 自 [ZIWTime] 以来, [XRName] 是否经历过交通事故，或任何的重大意外伤害，并接受了治疗?

1. 是
2. 否

DA020 [XRName] 是否经历过交通事故，或任何的重大意外伤害，并接受了治疗?

1. 是
2. 否

DA021 事故导致的伤害是否影响 [XRName] 现在的日常活动?

1. 是
2. 否

DA022 自 [ZIWTime] 以来，[XRName] 有没有摔倒过?

1. 是
2. 否

DA023 [XRName] 有没有摔倒过?

1. 是
2. 否

DA024 有多少次摔倒受伤严重到需要接受治疗? ______ [hc($[0, 99], int, \varnothing$)，sc($[0, 20), int, \varnothing$)] 次

DA025 自 [ZIWTime] 以来，[XRName] 有没有过髋骨骨折?

【**访员注意**：髋骨”指人体腰部的骨骼，共左右两块。幼年时，髋骨分为髂骨, 坐骨和耻骨以及软骨连接。成年后，它们之间的软骨会骨化，成为一个整体，即髋骨】

1. 有
2. 没有

DA026 [XRName] 有没有过髋骨骨折?

【**访员注意**：髋骨”指人体腰部的骨骼，共左右两块。幼年时，髋骨分为髂骨, 坐骨和耻骨以及软骨连接。成年后，它们之间的软骨会骨化，成为一个整体，即髋骨】

1. 有
2. 没有

DA027 [XRName] 是否经常因为疼痛而难受? 是完全没有、有一点、有一些、比较多、还是非常多?

【**访员注意**：这里询问的是身体所有部位的疼痛情况】

1. 完全没有
2. 有一点
3. 有一些
4. 比较多

5. 非常多

DA028 身体哪些部位感到疼痛？请列出所有部位。

1. 头
2. 肩膀
3. 胳膊
4. 手腕
5. 手指
6. 胸
7. 胃
8. 背
9. 腰
10. 臀部
11. 腿
12. 膝盖
13. 脚踝
14. 脚趾
15. 脖子
16. 其它部位，请注明 _____ (**DA028_1**)

DA029 下面我想问一下您主观预期寿命的一个问题，这个问题反映了您对自身健康状况的预期。假定有五个级别，最低一级代表可能性最小，最高一级代表可能性最大，您设想您活到 [XFAge-Possibility] 这个年龄的可能性有多大？是几乎不可能、不太可能、有可能、很可能、还是简直一定？

1. 几乎不可能
2. 不太可能
3. 有可能
4. 很可能
5. 简直一定

997. 不知道

DA. 健康状况（三）

DA030 过去一个月内，[XRName] 平均每天晚上真正睡着的时间大约是几小时？（可能短于 [XRName] 在床上躺着的时间） _____ [hc$([0, 24], real, -1)$] 小时

【**访员注意**：不知道请填写-1】

DA031 过去一个月内，[XRName] 通常午睡多长时间？ _____ [hc$([0, 300], int, -1)$] 分钟

【**访员注意**：如果受访者不午睡，请记录为 0；不知道请填写-1】

【**引语**：下面有一些问题是有关 [XRName] 通常每周花了多少时间做这些活动?】

DA032[i] 下面请回忆下 [XRName] 通常每周做的 [XPsyActType[i]] ，只需回忆 [XRName] 每次运动了至少十分钟的活动。[XRName] 通常每周有没有至少持续做这种类型的活动十分钟？

1. 是
2. 否

DA033[i] [XRName] 通常每周有多少天做 [XPsyActType[i]] 至少十分钟? ______ [hc([1, 7], int, ∅)] 天

DA034[i] 在做 [XPsyActType[i]] 的这些天里，[XRName] 一天花多少时间做 [XPsyActType[i]]？

1. <2 小时
2. >=2 小时

DA035[i] 在做 [XPsyActType[i]] 的这些天里，[XRName] 一天花多少时间做 [XPsyActType[i]]？

1. <30 分钟
2. >=30 分

DA036[i] 在做 [XPsyActType[i]] 的这些天里，[XRName] 一天花多少时间做 [XPsyActType[i]]？

1. <4 小时
2. >=4 小时

DA037[i] 做 [XPsyActType[i]] 是因为工作需要、娱乐活动、体育锻炼还是其他?

1. 工作需要
2. 娱乐
3. 体育锻炼
4. 其他 ______ (**DA037_1[i]**)

DA038 [XRName] 过去一个月是否进行了下列社交活动?（可多选）

1. 串门、跟朋友交往
2. 打麻将、下棋、打牌、去社区活动室
3. 向与您不住在一起的亲人、朋友或者邻居提供帮助
4. 跳舞、健身、练气功等
5. 参加社团组织活动
6. 志愿者活动，或者慈善活动，或者照顾与您不住在一起的病人或残疾人
7. 上学或者参加培训课程
8. 其他社交活动，请注明 ______ (**DA038_1**)
9. 以上均没有

[conflict(9, $[9]^c$)]

DA039[i] 过去一个月，[XRName] 每隔多长时间会做刚才说的这些活动 [XSocType[i]]？差不多每天，差不多每周或不经常?

1. 差不多每天
2. 差不多每周
3. 不经常

DA040 过去一个月，您是否上网？包括用手机网络聊天、看新闻、看视频、玩游戏、理财等

1. 是
2. 否

DA041 请问 [XRName] 使用以下哪些工具上网?（可多选）

1. 台式电脑
2. 笔记本电脑
3. 平板电脑（如 IPAD）
4. 手机
5. 其他设备，请注明 ______ (**DA041_1**)

DA042 请问 [XRName] 上网一般做什么？（可多选）

1. 聊天
2. 看新闻
3. 看视频
4. 玩游戏
5. 理财
6. 其他，请注明 ______ (**DA042_1**)

DA043 [XRName] 是否会用手机支付，如支付宝、微信钱包等？

1. 会
2. 不会

DA044 [XRName] 是否使用微信？

1. 使用
2. 不使用

DA045 [XRName] 发不发微信朋友圈？

1. 发
2. 不发

DA046 [XRName] 吸过烟吗？（包括香烟、旱烟、用烟管吸烟或咀嚼烟草）

1. 是
2. 否

DA047 [XRName] 现在还在吸烟还是戒烟了？

1. 仍然抽烟
2. 戒烟
3. 从未吸过烟

DA048 [XRName] 吸烟时，一般抽什么烟？

1. 用烟管吸烟（烟袋、旱烟）
2. 自己卷烟抽
3. 带滤咀香烟
4. 不带滤咀香烟
5. 雪茄
6. 水烟

DA049 [XRName] 这次成功戒烟是在多少岁或那一年？

【访员注意：不知道请填写-1】

1. 年龄 ______ [hc([0, 120], int, −1)，sc((0, 100], int, −1)] (**DA049_1**) 岁
2. 年份 ______ [hc([1900, 2020], int, −1)，sc([1920, 2020], int, −1)] (**DA049_2**) 年

DA050_1 [XRName] 现在平均一天抽多少支香烟？ ______ [hc([0, 200], int, ∅)] 支

DA050_2 [XRName] 戒烟前平均一天抽多少支香烟？ ______ [hc([0, 200], int, −1)] 支

【访员注意：不知道请填写-1】

DA051 在过去的一年，[XRName] 喝酒吗，包括啤酒、葡萄酒、米酒、黄酒或白酒、药酒等？喝酒频率如何？

1. 喝酒，每月超过一次
2. 喝酒，但每月少于一次
3. 什么都不喝

DA052 过去一年内 [XRName] 平均一个月喝几次酒？
1. 每月一次
2. 每月 2-3 次
3. 每周一次
4. 每周 2-3 次
5. 每周 4-6 次
6. 每天一次
7. 一天两次
8. 一天超过两次

DB. 身体功能障碍以及辅助者（一）

【引语：下面我们想了解一下 [XRName] 日常生活的情况。请问 [XRName] 目前是否因为身体、精神、情感或者记忆方面的原因导致完成下面我们提到的一些日常行为有困难。我们指的“困难”不包括那些预计三个月内能够解决的困难。】

DB001 请问 [XRName] 是否因为健康和记忆的原因，自己穿衣服有困难？穿衣服包括从衣橱中拿出衣服，穿上衣服，扣上钮扣，系上腰带。
1. 没有困难
2. 有困难但仍可以完成
3. 有困难，需要帮助
4. 无法完成

DB002 穿衣服的时候是否有人帮助 [XRName]？
1. 有
2. 没有

DB003 请问 [XRName] 是否因为健康和记忆的原因，洗澡有困难？
1. 没有困难
2. 有困难但仍可以完成
3. 有困难，需要帮助
4. 无法完成

DB004 洗澡的时候是否有人帮助 [XRName]？
1. 有
2. 没有

DB005 请问 [XRName] 是否因为健康和记忆的原因，自己吃饭有困难，比如自己夹菜？（定义：当饭菜准备好以后，自己吃饭定义为用餐）
1. 没有困难
2. 有困难但仍可以完成
3. 有困难，需要帮助
4. 无法完成

DB006 吃饭的时候是否有人帮助 [XRName]？
1. 有
2. 没有

DB007 [XRName] 起床、下床有没有困难?
1. 没有困难
2. 有困难但仍可以完成
3. 有困难，需要帮助
4. 无法完成

DB008 起床、下床是否有人帮 [XRName]？
1. 有
2. 没有

DB009 请问 [XRName] 是否因为健康和记忆的原因，上厕所有困难，包括蹲下、站起?
1. 没有困难
2. 有困难但仍可以完成
3. 有困难，需要帮助
4. 无法完成

DB010 上厕所是否有人帮 [XRName]？
1. 有
2. 没有

DB011 请问 [XRName] 是否因为健康和记忆的原因，控制大小便有困难?（自己能够使用导尿管或者尿袋算能够控制自理）
1. 没有困难
2. 有困难但仍可以完成
3. 有困难，需要帮助
4. 无法完成

DB012 请问 [XRName] 是否因为健康和记忆的原因，做家务活的时候有困难?（定义：做家务，我们指的是房屋清洁，洗碗盘，整理被褥和房间摆设）

【**访员注意**：如果受访者不能拖地，但是可以擦洗桌子，或者受访者不能整理重的被褥，但是可以整理一些轻便的，请选择（3）】
1. 没有困难
2. 有困难但仍可以完成
3. 有困难，需要帮助
4. 无法完成

DB013 做家务的时候是否有人帮助 [XRName]？
1. 有
2. 没有

DB014 请问 [XRName] 是否因为健康和记忆的原因，做饭有困难?（定义：做饭我们定义为准备原材料，做饭菜，端上餐桌）

【**访员注意**：如果由于健康原因，受访者需要别人帮忙洗菜切菜，或者受访者只能自己煮米饭但不能做菜，也就是说，由于健康原因受访者只能完成做饭的一些简单的动作，那么选择（3）】

1. 没有困难
2. 有困难但仍可以完成
3. 有困难，需要帮助
4. 无法完成

DB015 做饭的时候是否有人帮助 [XRName]？
1. 有
2. 没有

DB016 请问 [XRName] 是否因为健康和记忆的原因，自己去商店买食品杂货有困难？我们这里说的买东西是指决定买什么和付钱。
1. 没有困难
2. 有困难但仍可以完成
3. 有困难，需要帮助
4. 无法完成

DB017 是否有人帮助 [XRName] 去商店买食品杂货等？
1. 有
2. 没有

DB018 请问 [XRName] 是否因为健康和记忆的原因，拨打电话有困难？
1. 没有困难
2. 有困难但仍可以完成
3. 有困难，需要帮助
4. 无法完成

DB019 打电话的时候是否有人帮助 [XRName]？
1. 有
2. 没有

DB020 请问 [XRName] 是否因为健康和记忆的原因，自己吃药有困难？吃药是指能记得什么时间吃和吃多少。
1. 没有困难
2. 有困难但仍可以完成
3. 有困难，需要帮助
4. 无法完成

DB021 吃药的时候是否有人帮助 [XRName]？
1. 有
2. 没有

DB022 请问 [XRName] 是否因为健康和记忆的原因，管钱有困难，比如支付账单、记录支出项目、管理财物？
1. 没有困难
2. 有困难但仍可以完成
3. 有困难，需要帮助
4. 无法完成

DB023 是否有人帮助 [XRName] 管钱？

1. 有
2. 没有

DB. 身体功能障碍以及辅助者（二）

DB024 请问在以上（穿衣、洗澡、吃饭、起床、入厕、家务、做饭、购物、打电话、吃药、管钱等）困难中，都有谁帮助 [XRName]？（可多选）

1. 配偶
2. 父母、岳父母、公公、婆婆
3. 子女、儿媳/女婿、孙子女/外孙子女
4. 兄弟姐妹及其配偶、子女，[XRName] 配偶的兄弟姐妹及其配偶、子女
5. 其他亲属
6. 雇佣人员（如保姆），共 ______ [hc((0, 99), *int*, −1)，sc([1, 10), *int*, −1)] (**DB024_1**) 位
7. 志愿者或者志愿机构人员
8. 养老院人员
9. 居家养老服务机构人员
10. 社区提供的帮助
11. 其他人员，请注明 ______ (**DB024_2**)

DB025 在父母、岳父母、公公、婆婆中，帮助 [XRName] 的是哪几位?（可多选）

1. 父亲
2. 母亲
3. 岳父/公公
4. 岳母/婆婆

DB026 帮助 [XRName] 的子女、儿媳/女婿、孙子女/外孙子女，是以下哪个子女家的?（可多选）

1-25. [XChildPanAliveName[*i*]]
26-35. 其他子女，名字为 ______ (**DB026_1[i]**)

DB027[*i*] [XHelperChild[*i*]] 家的哪些人亲自帮助 [XRName]？（可多选）

1. [XHelperChild[*i*]] 本人
2. [XHelperChild[*i*]] 的配偶
3. [XHelperChild[*i*]] 的孩子，即 [XRName] 的（外）孙子女，亲自帮助 [XRName] 的 [XHelperChild[*i*]] 的孩子有几个 ______ [hc((0, 99), *int*, −1)，sc([1, 10), *int*, −1)] (**DB027_1[i]**)

DB028 帮助 [XRName] 的兄弟姐妹及其配偶、子女，[XRName] 配偶的兄弟姐妹及其配偶、子女，是以下哪个兄弟姐妹家的?（可多选）

1-30. [XSibName[*i*]]
31-40. 其他兄弟姐妹，名字为 ______ (**DB028_1[i]**)

DB029[*i*] [XHelperSib[*i*]] 家的哪些人亲自帮助 [XRName]？（可多选）

1. [XHelperSib[*i*]] 本人
2. [XHelperSib[*i*]] 的配偶
3. [XHelperSib[*i*]] 的孩子，即 [XRName] 的外甥外甥女、侄子侄女，亲自帮助 [XRName] 的 [XHelperSib[*i*]] 的孩子有几个 ______ [hc((0, 99), *int*, −1)，sc([1, 10), *int*, −1)] (**DB029_1[i]**) 个

DB030 亲自为 [XRName] 提供帮助的其他亲属共有位？ ______ [hc((0, 99), *int*, −1)，sc([1, 10), *int*, −1)] 位

【**访员注意**：不知道请填写-1】

DB030_1 都是 [XRName] 的什么人？ ______

DB031 亲自为 [XRName] 提供帮助的其他人共有几位？ ______ [hc((0, 99), *int*, −1)，sc([1, 10), *int*, −1)] 位

【**访员注意**：不知道请填写-1】

DB031_1 都是 [XRName] 的什么人？ ______

DB032 在下面所列的所有帮助者中，请选择帮助 [XRName] 最多的 7 类人。

1-99. [XHelper[*i*]]

DB033[*i*] 在过去一个月内，[XHelpList[*i*]] 帮助了 [XRName] 多少天？ ______ [hc([0, 31], *int*, −1)] 天

【**访员注意**：不知道请填写-1】

DB034[*i*] 在 [XHelpList[*i*]] 帮助 [XRName] 的那些天，他/她大概每天花多少小时帮助 [XRName] ？ ______ [hc([0, 24], *int*, −1)] 小时

【**访员注意**：少于一个小时请记为 1；
不知道请填写-1】

DB035[*i*] [XHelpList[*i*]] 在照顾 [XRName] 的时候，是否和 [XRName] 住在一起？

1. 是
2. 否

DB048 有没有因为疫情的缘故，使 [XRName] 获得的照料发生变化呢？是获得的照料更多了、更少了，还是没变？

1. 更多了
2. 更少了
3. 没有变

DB. 身体功能障碍以及辅助者（三）

DB036 如果以后 [XRName] 在日常生活方面需要照顾，比如吃饭，穿衣，有亲人或朋友能长期照顾 [XRName] 吗？

1. 是
2. 否

DB037 他/她是 [XRName] 的什么人？（可多选）

1. 配偶
2. 父母、岳父母、公公、婆婆
3. 子女、儿媳/女婿、孙子女/外孙子女
4. 兄弟姐妹及其配偶、子女，[XRName] 配偶的兄弟姐妹及其配偶、子女
5. 其他亲属
6. 雇佣人员（如保姆），共 ______ [hc((0, 99), *int*, −1)，sc([1, 10), *int*, −1)] (**DB037_1**) 位
7. 志愿者或者志愿机构人员

8. 养老院人员
9. 居家养老服务机构人员
10. 社区提供的帮助
11. 其他人员，请注明 ______ (**DB037_2**)

DB038 在父母、岳父母、公公、婆婆中，以后会帮助 [XRName] 的是哪几位?（可多选）
1. 父亲
2. 母亲
3. 岳父/公公
4. 岳母/婆婆

DB039 以后会帮助 [XRName] 的子女、儿媳/女婿、孙子女/外孙子女，是以下哪个子女家的?（可多选）
1-25. [XChildPanAliveName[i]]
26-35. 其他子女，名字为 ______ (**DB039_1[i]**)

DB040 以后会帮助 [XRName] 的兄弟姐妹及其配偶、子女，[XRName] 配偶的兄弟姐妹及其配偶、子女，是以下哪个兄弟姐妹家的?（多选题）
1-30. [XSibName[i]]
31-40. 其他兄弟姐妹，名字为 ______ (**DB040_1[i]**)

DB041 以后会亲自为 [XRName] 提供帮助的其他亲属共有几位? ______ [hc$((0,99),int,-1)$，sc$([1,10),int,-1)$] 位
【访员注意：不知道请填写-1】

DB042 以后会亲自为 [XRName] 提供帮助的其他人共有几位? ______ [hc$((0,99),int,-1)$，sc$([1,10),int,-1)$] 位
【访员注意：不知道请填写-1】

【引语：下面我们想知道，[XRName] 有没有因为身体原因影响到 [XRName] 的工作能力。】

DB043 您看我这句话是否符合 [XRName] 的情况：因为残疾或健康原因，我无法正常工作或劳动。
1. 我完全无法正常工作或劳动
2. 我不能长时间工作或劳动
3. 我做起来没有问题

DB044 您看我这句话是否符合 [XRName] 的情况：因为残疾或健康原因，我无法正常做家务。
1. 我完全无法正常做家务
2. 我不能长时间做家务
3. 我做起来没有问题

DB045 访员观察：[XRName] 填写该部分问卷时是否求助?
【访员注意：访员注意：如果是协助回答，请记录受访者的反应】
1. 从不
2. 有一些时候
3. 大多数时候
4. 受访者不在场，完全请人代填

DB046 访员观察：代填者和 [XRName] 是什么关系?

【访员注意：代填问卷的人和受访者是什么关系。如果不清楚，请问代填者】

1. 配偶
2. 母亲
3. 父亲
4. 岳母/婆婆
5. 岳父公公
6. 兄弟姐妹
7. 姐夫妹夫/嫂子弟媳
8. 孩子
9. 孩子的配偶
10. 孙子女
11. 其他亲戚
12. 帮忙的人或者其他非亲属

DB047 访员观察：[XRName] 不在场，完全请人代填的主要原因是什么？

【访员注意：记录代答的原因】

1. 受访者有严重身体障碍
2. 受访者有严重精神障碍
3. 受访者拒访
4. 其他，请注明 ______ (**DB047_1**)

DC. 认知和抑郁（一）

【引语：首先我将会问你一些问题以检查你的记忆力和注意力。其中有些问题简单，有些问题比较难。】

DC001 今年是哪一年？

1. 正确
2. 错误

997. 不知道
999. 拒绝回答

DC002 现在是什么季节？

1. 正确
2. 错误

997. 不知道
999. 拒绝回答

DC003 今天是这个月的几号？

【访员注意：阴历日期也正确，不允许受访者查手机或者日历】

1. 正确
2. 错误

997. 不知道
999. 拒绝回答

DC004 今天是星期几？

1. 正确
2. 错误

997．不知道
999．拒绝回答

DC005 现在是几月份？

【**访员注意**：阴历月份也正确，不允许受访者查手机或者日历】

1．正确
2．错误
997．不知道
999．拒绝回答

DC006 您觉得自己现在的记忆力怎么样？是极好、很好、好、一般还是不好？

1．极好
2．很好
3．好
4．一般
5．不好
997．不知道

【**引语**：接下来我需要您仔细听我说的话，然后照我说的话做。您现在可以仔细听我讲话吗？准备好了吗，我们开始。请您算一算 100 减去 7，然后从所得的数字再减去 7，如此一直计算下去，请您将每减一个 7 后的答案告诉我，直到我说"停"为止。】

DC007_1 记录答案：

【**访员注意**：指导语读完后，受访者在计算过程中，请不要再向受访者发出任何指令，也不能提醒受访者应该怎么做。请记住不能给任何额外提示，只能说"继续"；
当受访者不给正面回应，访员在提醒 3 次"继续"后，如果受访者仍然不知道做什么，可以选择"不知道"选项；
此处的测试允许受访者使用纸和笔辅助完成】

1．记录答案 ______ [] (**DC007_1_1**)
997．不知道
999．拒绝回答

DC007_2 记录答案：

1．记录答案 ______ [] (**DC007_2_1**)
997．不知道
999．拒绝回答

DC007_3 记录答案：

1．记录答案 ______ [] (**DC007_3_1**)
997．不知道
999．拒绝回答

DC007_4 记录答案：

1．记录答案 ______ [] (**DC007_4_1**)
997．不知道
999．拒绝回答

DC007_5 记录答案：

1. 记录答案 ______ [] (**DC007_5_1**)
997. 不知道
999. 拒绝回答

DC008 访员观察：受访者在回答这些算术题时，是否用了纸、笔或其他辅助工具？
1. 用了辅助工具
2. 没用辅助工具

DC009 这里有一幅图。请您按照图的样子画在这里。transferPic("DC009")
请点击下方空白框，将受访者画好的图形拍照到访问系统留存 ______ (**DC009_photo**)
【**访员注意**：如果受访者所复制的图形满足
A) 两个四边相交的五边形，
B) 五边内的所有角都是完整的，
则算作正确】
1. 正确
2. 错误
3. 未评估（受访者因为身体客观原因无法完成）
997. 不知道
999. 拒绝回答

DC. 认知和抑郁（二）

DC010_1 接下来我将向您读十个词语。请您跟着我将这十个词语依次大声朗读出来。接着我会请您回忆这十个词语。您明白了么？transferPic("XWordlist","9 3 1 2 10 4 5 6 8 7")
【**访员注意**：请检查受访者是否明白如何进行这项测试。
以缓慢、稳定的速度向受访者读词，请该受访者在你读完后重复。频率掌握在约每两秒一个词。
只有在受访者听力有障碍的情况下，才能给受访者展示词表，一边展示一边读词。在换到下一组词语前，你应该大声朗读完，并请受访者重复该词。】
1. 是
2. 否

DC010_2 接下来我将向您读十个词语。请您跟着我将这十个词语依次大声朗读出来。接着我会请您回忆这十个词语。您明白了么？transferPic("XWordlist","9 3 1 2 10 4 5 6 8 7")
【**访员注意**：请检查受访者是否明白如何进行这项测试。
以缓慢、稳定的速度向受访者读词，请该受访者在你读完后重复。频率掌握在约每两秒一个词。
只有在受访者听力有障碍的情况下，才能给受访者展示词表，一边展示一边读词。在换到下一组词语前，你应该大声朗读完，并请受访者重复该词。】
1. 是
2. 否

DC010_3 接下来我将向您读十个词语。请您跟着我将这十个词语依次大声朗读出来。接着我会请您回忆这十个词语。您明白了么？transferPic("XWordlist","9 3 1 2 10 4 5 6 8 7")
【**访员注意**：请检查受访者是否明白如何进行这项测试。
以缓慢、稳定的速度向受访者读词，请该受访者在你读完后重复。频率掌握在约每两秒一个词。
只有在受访者听力有障碍的情况下，才能给受访者展示词表，一边展示一边读词。在换到下一组词语前，你应该大声朗读完，并请受访者重复该词。】

1. 是
2. 否

DC011 访员观察：请记录受访者为什么无法完成此项测试？（可多选）
1. 拒绝或不愿意进行此项测试
2. 终生无法说话
3. 进入老年阶段后开始无法说话
4. 耳聋或听力不好
5. 其他，请注明 ______ (**DC011_1**)

DC012 现在请告诉我您可以想起来的词语。

【**访员注意**：以缓慢、稳定的速度向受访者读词，请该受访者在你读完后重复。频率掌握在约每两秒一个词。只有在受访者听力有障碍的情况下，才能给受访者展示词表，一边展示一边读词。在换到下一组词语前，你应该大声朗读完，并请受访者重复该词。

受访者需要多长时间就给多长时间，最多可以有两分钟】

1. [XWordlist[9]]
2. [XWordlist[3]]
3. [XWordlist[1]]
4. [XWordlist[2]]
5. [XWordlist[10]]
6. [XWordlist[4]]
7. [XWordlist[5]]
8. [XWordlist[6]]
9. [XWordlist[8]]
10. [XWordlist[7]]
11. 没有回忆起任何词语
12. 拒绝回忆
13. 受访者不明白或不能进行此项测试

[conflict(11, 12, 13, [11, 12, 13]^c)]

【**引语**：现在我将以一个不同的顺序来向您读出和刚才相同的一系列词语。请您跟着我依次将这十个词语大声朗读出来。接着我会请您回忆这十个词语。准备好了么】

DC013 现在请告诉我您可以想起来的词语。transferPic("XWordlist","5 2 9 10 3 1 8 4 6 7")

【**访员注意**：以缓慢、稳定的速度向受访者读词，请该受访者在你读完后重复。频率掌握在约每两秒一个词。只有在受访者听力有障碍的情况下，才能给受访者展示词表，一边展示一边读词。在换到下一组词语前，你应该大声朗读完，并请受访者重复该词。

受访者需要多长时间就给多长时间，最多可以有两分钟】

1. [XWordlist[5]]
2. [XWordlist[2]]
3. [XWordlist[9]]
4. [XWordlist[10]]
5. [XWordlist[3]]
6. [XWordlist[1]]
7. [XWordlist[8]]
8. [XWordlist[4]]
9. [XWordlist[6]]

10. [XWordlist[7]]
11. 没有回忆起任何词语
12. 拒绝回忆

[conflict(11, 12, [11, 12]^c)]

【引语：现在我将以一个不同的顺序来向您读出和刚才相同的一系列词语。请您跟着我依次将这十个词语大声朗读出来。接着我会请您回忆这十个词语。准备好了么?】

DC014 现在请告诉我您可以想起来的词语。transferPic("XWordlist","1 2 3 4 5 6 7 8 9 10")

【**访员注意**：以缓慢、稳定的速度向受访者读词，请该受访者在你读完后重复。频率掌握在约每两秒一个词。只有在受访者听力有障碍的情况下，才能给受访者展示词表，一边展示一边读词。在换到下一组词语前，你应该大声朗读完，并请受访者重复该词。

受访者需要多长时间就给多长时间，最多可以有两分钟】

1. [XWordlist[1]]
2. [XWordlist[2]]
3. [XWordlist[3]]
4. [XWordlist[4]]
5. [XWordlist[5]]
6. [XWordlist[6]]
7. [XWordlist[7]]
8. [XWordlist[8]]
9. [XWordlist[9]]
10. [XWordlist[10]]
11. 没有回忆起任何词语
12. 拒绝回忆

[conflict(11, 12, [11, 12]^c)]

DC015 访员观察：请注明在整个过程中是否有发生下列情况（多选）

1. 当进行词表时有被迫中断
2. 其他情况 ______ (**DC015_1**)
3. 以上情况均未发

[conflict(3, [3]^c)]

【引语：下面 10 道问题是有关您上周的感觉及行为，每道题目的答案都是一样的，包括很少或者根本没有，不太多，有时或者说有一半的时间还是大多数的时间，请您选择合适的答案】

DC016 我因一些小事而烦恼。

【**访员注意**：如果受访者无法理解题目的意思，请继续向受访者重复题干，不能随便回答不知道】

1. 很少或者根本没有（<1 天）
2. 不太多（1～2 天）
3. 有时或者说有一半的时间（3～4 天）
4. 大多数的时间（5～7 天）

997. 不知道
999. 拒绝回答

DC017 我在做事时很难集中精力。

【**访员注意**：如果受访者无法理解题目的意思，请继续向受访者重复题干，不能随便回答不知道】

1. 很少或者根本没有（<1 天）

2. 不太多（1～2 天）
3. 有时或者说有一半的时间（3～4 天）
4. 大多数的时间（5～7 天）
997. 不知道
999. 拒绝回答

DC018 我感到情绪低落。

【**访员注意**：如果受访者无法理解题目的意思，请继续向受访者重复题干，不能随便回答不知道】

1. 很少或者根本没有（<1 天）
2. 不太多（1～2 天）
3. 有时或者说有一半的时间（3～4 天）
4. 大多数的时间（5～7 天）
997. 不知道
999. 拒绝回答

DC019 我觉得做任何事都很费劲。

【**访员注意**：如果受访者无法理解题目的意思，请继续向受访者重复题干，不能随便回答不知道】

1. 很少或者根本没有（<1 天）
2. 不太多（1～2 天）
3. 有时或者说有一半的时间（3～4 天）
4. 大多数的时间（5～7 天）
997. 不知道
999. 拒绝回答

DC020 我对未来充满希望。

【**访员注意**：如果受访者无法理解题目的意思，请继续向受访者重复题干，不能随便回答不知道】

1. 很少或者根本没有（<1 天）
2. 不太多（1～2 天）
3. 有时或者说有一半的时间（3～4 天）
4. 大多数的时间（5～7 天）
997. 不知道
999. 拒绝回答

DC021 我感到害怕。

【**访员注意**：如果受访者无法理解题目的意思，请继续向受访者重复题干，不能随便回答不知道】

1. 很少或者根本没有（<1 天）
2. 不太多（1～2 天）
3. 有时或者说有一半的时间（3～4 天）
4. 大多数的时间（5～7 天）
997. 不知道
999. 拒绝回答

DC022 我的睡眠不好。

【**访员注意**：如果受访者无法理解题目的意思，请继续向受访者重复题干，不能随便回答不知道】

1. 很少或者根本没有（<1 天）
2. 不太多（1～2 天）
3. 有时或者说有一半的时间（3～4 天）

4. 大多数的时间（5～7 天）
997. 不知道
999. 拒绝回答

DC023 我很愉快。

【**访员注意**：如果受访者无法理解题目的意思，请继续向受访者重复题干，不能随便回答不知道】

1. 很少或者根本没有（<1 天）
2. 不太多（1～2 天）
3. 有时或者说有一半的时间（3～4 天）
4. 大多数的时间（5～7 天）
997. 不知道
999. 拒绝回答

DC024 我感到孤独。

【**访员注意**：如果受访者无法理解题目的意思，请继续向受访者重复题干，不能随便回答不知道】

1. 很少或者根本没有（<1 天）
2. 不太多（1～2 天）
3. 有时或者说有一半的时间（3～4 天）
4. 大多数的时间（5～7 天）
997. 不知道
999. 拒绝回答

DC025 我觉得我无法继续我的生活。

【**访员注意**：如果受访者无法理解题目的意思，请继续向受访者重复题干，不能随便回答不知道】

1. 很少或者根本没有（<1 天）
2. 不太多（1～2 天）
3. 有时或者说有一半的时间（3～4 天）
4. 大多数的时间（5～7 天）
997. 不知道
999. 拒绝回答

DC026 总体来看，您对自己的生活是否感到满意？是极其满意，非常满意，比较满意，不太满意还是一点也不满意？

1. 极其满意
2. 非常满意
3. 比较满意
4. 不太满意
5. 一点也不满意

DC027 您对您和您子女的关系满意吗？是极其满意，非常满意，比较满意，不太满意还是一点也不满意？

【**访员注意**：仅对当前有存活子女的受访者询问此问题】

1. 极其满意
2. 非常满意
3. 比较满意
4. 不太满意
5. 一点也不满意

6. 现在没有子女

【引语：几分钟前我请您读了十张卡片上的词语。现在我想请您尽量尝试去回忆这十个词。好，现在请您告诉我您还记得这十个词里的哪几个词？越多越好。】

DC028 请选择受访者成功回忆起的词语。

【**访员注意**：受访者需要多长时间就给多长时间，最多可以有两分钟。】

1. [XWordlist[1]]
2. [XWordlist[2]]
3. [XWordlist[3]]
4. [XWordlist[4]]
5. [XWordlist[5]]
6. [XWordlist[6]]
7. [XWordlist[7]]
8. [XWordlist[8]]
9. [XWordlist[9]]
10. [XWordlist[10]]
11. 没有回忆起任何词语
12. 拒绝回忆

[conflict(11, 12, $[11, 12]^c$)]

DC029 访员观察：受访者访问过程中，有没有以下情况发生？（可多选）

1. 受访者视力不好
2. 受访者听力不好，未戴助听器
3. 受访者戴助听器
4. 受访者手抖，影响了某些测试
5. 访问过程中受到其他事务或噪音的干扰
6. 受访者本身情绪问题，导致问卷质量不高
7. 其他，请注明 ______ (**DC029_1**)
8. 以上都没有

[conflict(8, $[8]^c$)]

DC030 访员观察：访员在访问过程中使用的语言？

1. 普通话
2. 当地方言
3. 其他方言，请注明 ______ (**DC030_1**)

辅助变量定义

XChroDisType 慢性病类型

```
add("XChroDisType", ["高血压病", "血脂异常（高血脂或低血脂）", "糖尿病或血糖升高
↪ （包括糖耐量异常和空腹血糖升高）", "癌症等恶性肿瘤（不包括轻度皮肤癌）",
↪ "慢性肺部疾患如慢性支气管炎或肺气肿、肺心病（不包括肿瘤或癌）", "肝脏疾病（除脂肪肝、肿瘤或癌外）",
↪ "心脏病（如心肌梗塞、冠心病、心绞痛、充血性心力衰竭和其他心脏疾病）", "中风", "肾脏疾病
↪ （不包括肿瘤或癌）", "胃部疾病或消化系统疾病（不包括肿瘤或癌）", "情感及精神问题", "与记忆有关的疾病
↪ （老年痴呆症、脑萎缩）", "帕金森症", "关节炎或风湿病", "哮喘（非肺部疾病）"])
```

XFAgePossibility 询问活到特定年龄的具体数值

```
if (!greater("XRAge", "65") && !equal("XRAge", "65")) {
    add("XFAgePossibility", "75")
}
if ((greater("XRAge", "65") || equal("XRAge", "65")) && !greater("XRAge", "69")) {
    add("XFAgePossibility", "80")
}
if ((greater("XRAge", "70") || equal("XRAge", "70")) && !greater("XRAge", "74")) {
    add("XFAgePossibility", "85")
}
if ((greater("XRAge", "75") || equal("XRAge", "75")) && !greater("XRAge", "79")) {
    add("XFAgePossibility", "90")
}
if ((greater("XRAge", "80") || equal("XRAge", "80")) && !greater("XRAge", "84")) {
    add("XFAgePossibility", "95")
}
if ((greater("XRAge", "85") || equal("XRAge", "85")) && !greater("XRAge", "89")) {
    add("XFAgePossibility", "100")
}
if ((greater("XRAge", "90") || equal("XRAge", "90")) && !greater("XRAge", "94")) {
    add("XFAgePossibility", "105")
}
if ((greater("XRAge", "95") || equal("XRAge", "95")) && !greater("XRAge", "99")) {
    add("XFAgePossibility", "110")
}
if (greater("XRAge", "100") || equal("XRAge", "100")) {
    add("XFAgePossibility", "115")
}
```

XPsyActType 体力活动类型

```
add("XPsyActType", ["非常消耗体力的激烈活动（激烈的活动会让你呼吸急促，比如搬运重物、挖地、耕作、有氧运动、
↪  快速骑车、骑车载货等）", "中等强度的体力活动（中等体力的活动让您的呼吸比平时快一些，比如搬运轻便的东西、
↪  常规速度骑自行车、拖地、打太极拳、疾走）", "轻度体力活动如走路
↪  （走路包括工作或者在家的时候从一个地方走到另一个地方，以及其他您为了休闲、运动、锻炼或娱乐而散步）"])
```

XSocType 社交活动类型

```
if (selected("DA038","1")) {
     add("XSocType[1]", "串门、跟朋友交往")
}
if (selected("DA038","2")) {
    add("XSocType[2]", "打麻将、下棋、打牌、去社区活动室")
}
if (selected("DA038","3")) {
    add("XSocType[3]", "向与您不住在一起的亲人、朋友或者邻居提供帮助")
}
if (selected("DA038","4")) {
    add("XSocType[4]", "跳舞、健身、练气功等")
}
if (selected("DA038","5")) {
    add("XSocType[5]", "参加社团组织活动")
}
if (selected("DA038","6")) {
    add("XSocType[6]", "志愿者活动，或者慈善活动，或者照顾与您不住在一起的病人或残疾人")
}
if (selected("DA038","7")) {
    add("XSocType[7]", "上学或者参加培训课程")
}
if (selected("DA038","8")) {
    add("XSocType[8]", "其他社交活动")
}
```

XHelperSelect 是否有人帮助

```
if (equal("DB002", "1") || equal("DB004", "1") || equal("DB006", "1") || equal("DB008", "1") ||
↪  equal("DB010", "1") ||equal("DB013", "1") || equal("DB015", "1") || equal("DB017", "1") ||
↪  equal("DB019", "1") || equal("DB021", "1")  || equal("DB023", "1")) {
    add("XHelperSelect", "1")
} else {
    add("XHelperSelect", "0")
}
```

XHelperChild 提供帮助的子女姓名列表

```
for (var i1 = 1; i1 <= 25; i1++) {
    add("XHelperChild[i1]", value("XChildPanAliveName[i1]"))
}
for (var i1 = 26; i1 <= 35; i1++) {
    add("XHelperChild[i1]", value("DB026_1[i1]"))
}
```

XSibName 生成兄弟姐妹姓名列表

```
for (var i1 = 1; i1 <= 15; i1++) {
    if (!empty("ZSibName[i1]")) {
        add("XSibName[i1]", pre("XRName")+"的兄弟姐妹"+pre("ZSibName[i1]"))
    }
}
for (var i1 = 1; i1 <= 15; i1++) {
    if (!empty("ZSibNameS[i1]")) {
        add("XSibName["+(i1+15)+"]", pre("XRName")+"的配偶的兄弟姐妹"+pre("ZSibNameS[i1]"))
    }
}
```

XHelperSib 提供帮助的兄弟姐妹姓名

```
for (var i1 = 1; i1 < 31; i1++) {
    add("XHelperSib[i1]", value("XSibName[i1]"))
}
for (var i1 = 31; i1 <= 40; i1++) {
    add("XHelperSib[i1]", value("DB028_1[i1]"))
}
```

XHelperNum 提供帮助者的数量

```
add("XHelperNum", "0")
if (selected("DB024", "1")) {
    add("XHelperNum", value("XHelperNum")+1)
    add("XHelper["+value("XHelperNum")+"]", "配偶")
}
if (selected("DB024", "5")) {
    add("XHelperNum", value("XHelperNum")+1)
    add("XHelper["+value("XHelperNum")+"]", "其他亲属")
}

if (selected("DB024", "6")) {
    add("XHelperNum", value("XHelperNum")+1)
    add("XHelper["+value("XHelperNum")+"]", "雇佣人员")
}

if (selected("DB024", "7")) {
    add("XHelperNum", value("XHelperNum")+1)
    add("XHelper["+value("XHelperNum")+"]", "志愿者")
}

if (selected("DB024", "8")) {
    add("XHelperNum", value("XHelperNum")+1)
    add("XHelper["+value("XHelperNum")+"]", "养老院人员")
}

if (selected("DB024", "9")) {
    add("XHelperNum", value("XHelperNum")+1)
    add("XHelper["+value("XHelperNum")+"]", "居家养老服务机构人员")
}

if (selected("DB024", "10")) {
    add("XHelperNum", value("XHelperNum")+1)
    add("XHelper["+value("XHelperNum")+"]", "社区")
}

if (selected("DB024", "11")) {
    add("XHelperNum", value("XHelperNum")+1)
    add("XHelper["+value("XHelperNum")+"]", "其他人员")
}
if (selected("DB025", "1")) {
    add("XHelperNum", value("XHelperNum")+1)
    add("XHelper["+value("XHelperNum")+"]", "父亲")
}
```

```
if (selected("DB025", "2")) {
    add("XHelperNum", value("XHelperNum")+1)
    add("XHelper["+value("XHelperNum")+"]", "母亲")
}
if (selected("DB025", "3")) {
    add("XHelperNum", value("XHelperNum")+1)
    add("XHelper["+value("XHelperNum")+"]", "岳父/公公")
}
if (selected("DB025", "4")) {
    add("XHelperNum", value("XHelperNum")+1)
    add("XHelper["+value("XHelperNum")+"]", "岳母/婆婆")
}
for (var i1 = 1; i1 < 26; i1++) {
    if (selected("DB026", i1) && selected("DB027[i1]", "1")) {
        add("XHelperNum", value("XHelperNum")+1)
        add("XHelper["+value("XHelperNum")+"]", value("XChildPanAliveName[i1]")+"本人")
    }
    if (selected("DB026", i1) &&  selected("DB027[i1]", "2")) {
        add("XHelperNum", value("XHelperNum")+1)
        add("XHelper["+value("XHelperNum")+"]", value("XChildPanAliveName[i1]")+"配偶")
    }
    if (selected("DB026", i1) &&  selected("DB027[i1]", "3")) {
        add("XHelperNum", value("XHelperNum")+1)
        add("XHelper["+value("XHelperNum")+"]", value("XChildPanAliveName[i1]")+"的孩子")
    }
}
for (var i1 = 26; i1 < 36; i1++) {
    if (selected("DB026", i1) && selected("DB027[i1]", "1")) {
        add("XHelperNum", value("XHelperNum")+1)
        add("XHelper["+value("XHelperNum")+"]", value("DB026_1[i1]")+"本人")
    }
    if (selected("DB026", i1) &&  selected("DB027[i1]", "2")) {
        add("XHelperNum", value("XHelperNum")+1)
        add("XHelper["+value("XHelperNum")+"]", value("DB026_1[i1]")+"配偶")
    }
    if (selected("DB026", i1) &&  selected("DB027[i1]", "3")) {
        add("XHelperNum", value("XHelperNum")+1)
        add("XHelper["+value("XHelperNum")+"]", value("DB026_1[i1]")+"的孩子")
    }
}
for (var i1 = 1; i1 < 31; i1++) {
    if (selected("DB028", i1) &&  selected("DB029[i1]", "1")) {
        add("XHelperNum", value("XHelperNum")+1)
        add("XHelper["+value("XHelperNum")+"]", value("XSibName[i1]")+"本人")
    }
    if (selected("DB028", i1) &&  selected("DB029[i1]", "2")) {
        add("XHelperNum", value("XHelperNum")+1)
        add("XHelper["+value("XHelperNum")+"]", value("XSibName[i1]")+"配偶")
    }
    if (selected("DB028", i1) &&  selected("DB029[i1]", "3")) {
        add("XHelperNum", value("XHelperNum")+1)
        add("XHelper["+value("XHelperNum")+"]", value("XSibName[i1]")+"的孩子")
    }
}
for (var i1 = 31; i1 < 41; i1++) {
    if (selected("DB028", i1) &&  selected("DB029[i1]", "1")) {
        add("XHelperNum", value("XHelperNum")+1)
        add("XHelper["+value("XHelperNum")+"]", value("DB028_1[i1]")+"本人")
    }
    if (selected("DB028", i1) &&  selected("DB029[i1]", "2")) {
        add("XHelperNum", value("XHelperNum")+1)
        add("XHelper["+value("XHelperNum")+"]", value("DB028_1[i1]")+"配偶")
    }
    if (selected("DB028", i1) &&  selected("DB029[i1]", "3")) {
        add("XHelperNum", value("XHelperNum")+1)
        add("XHelper["+value("XHelperNum")+"]", value("DB028_1[i1]")+"的孩子")
    }
}
```

XHelper 帮助者的身份/名字

see above

XSelectNum 判断选出来的帮助者数量是否多于 7 个

```
add("XSelectNum", "0")
for (var i1 = 1; i1 < 99; i1++) {
    if (selected("DB032", i1)) {
        add("XSelectNum", value("XSelectNum")+1)
    }
}
```

XHelpList 选出来的主要帮助者

```
for (var i1 = 1; i1 < value("XHelperNum")+1; i1++) {
    if ( greater("XHelperNum", "7") && !equal("XHelperNum", "7") && selected("DB032", i1) ) {
        add("XHelpList[i1]", value("XHelper[i1]"))
    }
}
for (var i1 = 1; i1 < value("XHelperNum")+1; i1++) {
    if ( !greater("XHelperNum", "7") ) {
        add("XHelpList[i1]", value("XHelper[i1]"))
    }
}
```

XHelperCurrent 现在是否有照料者

```
if (greater("XHelperNum", "0")) {
    add("XHelperCurrent", "1")
} else {
    add("XHelperCurrent", "0")
}
```

XWordlist 生成词语测试的量表

```
if (!empty("XMainR")) {
    add("XWordlist", ["河流", "书本", "眼睛", "瓦房", "扁担", "邮票", "摩托", "小草", "鸡蛋", "主席"])
}
if (empty("XMainR")) {
    add("XWordlist", ["天空", "报纸", "胳膊", "楼房", "木棍", "车票", "汽车", "花朵", "牛奶", "总理"])
}
```

XWordRecallBR 是否做了即时词组回忆测试

```
if  (!selected("DC012", "13") && !selected("DC012", "12") && !empty("DC012"))  {
    add("XWordRecallBR", "1")
} else {
    add("XWordRecallBR", "0")
}
```

This page intentionally left blank

F　工作与退休

F. 代理模式确认

proxy_7 访员记录，对于工作模块是否使用代理问卷模式？

1. 是
2. 否

FA. 工作概况（一）

【引语：接下来我想了解一下 [XRName] 近来的工作情况。】

FA001 过去一年，[XRName] 有没有干过 10 天以上的农活？种地、管理果树、采集农林产品、养鱼、打鱼、养牲畜或者去市场销售自家生产的农产品都算是农活。

【访员注意：工作模块和收入模块对农业活动的定义是一致的，区别在于工作模块询问受访者自己干不干，收入模块询问家户成员干不干。】

1. 是
2. 否

FA002 [XRName] 干农活是给自家干，还是挣工资给其他农户、农场干，还是都有？（多选题，选项免读）

1. 给自家干
2. 挣工资，给其他农户/农场干

FA004 那我们考虑非农工作，上周 [XRName] 有没有工作了至少一个小时？挣工资打工、做生意或者给家庭生意帮工都算是工作。

【访员注意：工作模块和收入模块对受雇和非农自雇的定义是一致的，区别在于工作模块询问受访者自己干不干，收入模块询问家户成员干不干。】

1. 是
2. 否

FA005 [XRName] 是否有工作，上周没干过，目前正处在临时放假、休病假、或其他假期中，或者正在在职培训？

1. 是
2. 否

FA007 [XRName] 能够在确定的时间或者 6 个月以内，回到原工作么？

1. 是
2. 否

FA008 原单位或雇主是否仍然给 [XRName] 发工资？

1. 是
2. 否

FA009 [XPrefixMainJob] [XRName] 目前的非农工作是否不只一份？

【访员注意：一份接着一份的零工，不算是多份工作。】

1. 是，至少两份
2. 否，只有一份

FA010 [XPrefixMainJob] [XRName] 有多份工作。那么其中 [XRName] 的主要工作是什么？也就是问 [XRName] 平常工作时间最长的那份工作是什么？这份主要工作具体是 [XSuffixMainJob] 挣工资做非农工作，个体做生意，还是不拿工资为家庭经营帮工？我们后面会着重了解这份主要工作。(选项免读)

【**访员注意**：优先考虑未终止的工作。】

1. 非农受雇
2. 从事个体或者私营经济活动
3. 不拿工资为家庭经营活动帮工
4. [XWorkTypeFarmEmployed]

FA011 [XRName] 这份工作是挣工资打工上班，个体做生意，还是不拿工资为家庭经营帮工？(选项免读)

1. 非农受雇
2. 从事个体或者私营经济活动
3. 不拿工资为家庭经营活动帮工

FA. 工作概况（二）

FA013 上一次访问时我们获知，在 [ZIWTime] [XRName] 当时没在工作（包括务农），那么从那时起到现在，[XRName] 是什么时候开始工作（包括务农）的？

【**访员注意**：如果受访者回答上次访问时在工作，请选“否定上轮记录”。

如果受访者确认上次访问时不在工作，只是无法回答开始工作的月份，月份可填“-1”。】

1. ______ [hc([ZIWYear, 2020], int, ∅)，sc([2018, 2020], int, ∅)] (**FA013_1**) 年 ______ [hc([1, 12], int, −1)] (**FA013_2**) 月

995. 否定上轮记录：那时在工作

999. 拒绝回答

FA014 我们说 [XRName] 当时没在工作，是指 [ZIWTime] 的之前一年没干过农活而且之前一周也没干过非农工作。说 [XRName] 目前在工作，是指过去一年干过农活或者上一周干过非农工作或者在休假。请再确认一下，[XRName] 当时确实在工作吗？

【**访员注意**：如果受访者认为关于目前在工作的回答不准确，请返回前面进行修改。

如果受访者认为我们的记录无误，请返回上一题重新回答开始工作的时间。】

1. 受访者确认那时在工作

997. 不知道

999. 拒绝回答

FA015 [XRName] 开始工作的主要原因是什么？

1. 赚更多收入，或是生产更多自用的东西（如在家闲着没事干，又如种菜自己吃）
2. 健康原因（如之前自身健康状况不佳，现在康复）
3. 家庭原因（如之前在照料家庭成员，现在不用了）
4. 锻炼身体
5. 没特殊原因，就是想干活，完全不为了钱或者生产的东西
6. 其他，请说明 ______ (**FA015_1**)

FA016 上一次访问时我们获知，[ZIWTime] [XRName] 当时在工作。和我刚才的说法一样，干农活、挣工资打工、做生意和给家庭生意帮工都算是工作。那么从那时起到现在，[XRName] 是什么时

候停止工作的？

【访员注意：如果受访者回答上次访问时不在工作，请选“否定上轮记录”。如果受访者确认上次访问时在工作，只是无法回答停止工作的月份，月份可填“-1”。】

1. ______ [hc([ZIWYear,XIWYear], *int*, ∅)，sc([2018,XIWYear], *int*, ∅)] (**FA016_1**) 年 ______ [hc([1, 12], *int*, −1)] (**FA016_2**) 月

995. 否定上轮记录：那时没在工作

999. 拒绝回答

FA017 我们说 [XRName] 当时在工作，是指 [ZIWTime] 的之前一年干过农活或者之前一周干过非农工作。说 [XRName] 目前没在工作，是指过去一年没干过农活而且上一周没干过非农工作。请再确认一下，[XRName] 当时确实没在工作吗？

【访员注意：如果受访者认为关于目前没在工作的回答不准确，请返回前面进行修改。
如果受访者认为我们的记录无误，请返回上一题重新回答停止工作的时间。】

1. 受访者确认那时没在工作

997. 不知道

999. 拒绝回答

FA018 抱歉我们之前记录错了，[ZIWTime] [XRName] 不在工作，那么请问 [XRName] 上一次的工作是什么时候停止的？ ______ [hc([1950, 2020], *int*, ∅)] (**FA018_1**) 年 ______ [hc([1, 12], *int*, −1)] (**FA018_2**) 月

【访员注意：如果受访者无法回答月份，请填“-1”。】

FA019 [XRName] 停止工作的主要原因是什么？

1. 被动原因（如被单位辞退、店面被拆、土地被征用）
2. 生意赚钱少或觉得工资太低（包括把耕地租出去了）
3. 除了收入以外的工作原因（如路太远、不喜欢）
4. 健康原因（如自身健康状况不佳）
5. 家庭原因（如需照料家庭成员，又如家人不让干，又如结婚生子）
6. 退休原因（到了退休年龄而离开工作岗位）
7. 临时停工（如自雇经营者有事临时停业）
8. 其他，请说明 ______ (**FA019_1**)

FB. 农业自雇工作

【引语：接下来我想了解一下 [XRName] 给自家干的农活。】

FB001 过去一年中，[XRName] 干农活的地点是？

【访员注意：如受访者不知道街道和社区，可酌情选择】

1. 居住地的同个村/社区：[XRResidenceFull]
2. 居住地同区县 [XRResidenceCounty] 的其他村/社区：______ (**FB001_1**) 乡/镇/街道/村/社区
3. 居住地区县以外：______ (**FB001_2**) 省/市/区县 ______ (**FB001_3**) 乡/镇/街道/村/社区
4. 以上选项均不适用的地区（港澳台及国外）

999. 拒绝回答

FB002 [XRName] 家的农活属于下列哪种类型？（可多选）

1. 种植业（谷物豆薯、棉麻糖烟、蔬菜水果、中药茶草）

2. 林业（育种造林、木竹采运、森林经营）
3. 畜牧业（牲畜、家禽等饲养，狩猎）
4. 渔业（水产养殖、水产捕捞）
5. 专业及辅助性农林牧渔活动
997. 不知道
[conflict(997, [997]^c)]

FB003 [XRName] 给自家干的农活主要是下列哪种形式？
1. 生产劳动
2. 经营管理
3. 机械操作
4. 采购销售
5. 其他

FB005 过去一年中，[XRName] 有几个月在给自家干农活？ ______ [hc([1, 12], *int*, ∅)] 个月

FB006 过去一年 [XRName] 给自家干农活的月份中，一般每周干几天？ ______ [hc([1, 7], *int*, ∅)] 天
【**访员注意**：如果每月不足四天，每周平均不到一天，填“1”。】

FB007 过去一年 [XRName] 给自家干农活的日子里，一般每天要干几个小时？ ______ [hc([1, 24], *int*, ∅), sc([1, 16], *int*, ∅)] 小时

FC. 受雇工作（一）

【**引语**：接下来我想了解一下刚才说到的 [XRName] 那份挣工资 [XWorkTypeFarmEmployed] 工作。如果 [XRName] 从事的是零工工作，雇主单位不固定，下面的工作单位都是指现在这份工作或是最近一份工作的单位。】

FC001 [XRName] 的工资是从工作单位/老板拿，还是要通过某个派遣单位或个体包工头领取？（选项免读）
1. 工作单位（可以是个人，并为之工作）
2. 劳务派遣单位
3. 个体包工头（作为劳务中介，只发工资）

FC002 [XRName] 的工作单位（或雇主）属于哪种类型？ [XFC002]
1. 政府部门
2. 事业单位
3. 非营利机构，比如社团、协会、学会等
4. 企业
5. 个体户
6. 农户
7. 居民户
8. 其他，请注明 ______ (**FC002_1**)
997. 不知道

FC003 我记得上一次访问时，在 [ZIWTime] [XRName] 的 [XWorkplace] 是 [ZFD003]，现在 [XRName] 的 [XWorkplace] 还是这个吗？
【**访员注意**：如单位/雇主没有划分部门，部门名称填“无”】

1. 是，单位中现所在部门的名称：（例 1：农园餐厅；例 2：纺纱生产车间） ______ (**FC003_2**)
2. 否

995. 否定上轮记录：是同个 [XWorkplace]，但上次的名称写得不对

FC003_1 现在 [XRName] 的 [XFC003]？工作单位/雇主名称是：（例 1：北京大学餐饮服务中心；例 2：北京华信服装服饰有限责任公司；例 3：张老板） ______
所在部门名称是：（例 1：农园餐厅；例 2：纺纱生产车间） ______ (**FC003_2**)
【**访员注意**：如单位/雇主没有划分部门，部门名称填“无”】

FC004 [XRName] 现在工作（单位）的地点是在？
【**访员注意**：如受访者不知道街道和社区，可酌情选择】
1. 居住地的同个村/社区：[XRResidenceFull]
2. 居住地同区县 [XRResidenceCounty] 的其他村/社区：______ (**FC004_1**) 乡/镇/街道/村/社区
3. 居住地区县以外：______ (**FC004_2**) 省/市/区县 ______ (**FC004_3**) 乡/镇/街道/村/社区
4. 以上选项均不适用的地区（港澳台及国外）

999. 拒绝回答

FC005 [XRName] 的 [XWorkplace] 主要属于哪个行业，也就是说，[XWorkplace] 现在制造什么产品或者提供什么服务？（例 1：为校内提供餐饮服务；例 2：制造纱质布料） ______
【**访员注意**：请按《行业与职业填写规范》的要求填写】

FC006 [XRName] 现在是公务员吗？
1. 是
2. 否

FC008 [XRName] 现在是正式编制内的员工吗？
1. 是
2. 否

FC011 [XRName] 这份工作属于什么职业？也就是说，[XRName] 的工作现在具体做些什么？（例 1：餐厅面点厨师；例 2：生产线绕线工人） ______
【**访员注意**：请按《行业与职业填写规范》的要求填写】

FC012 [XRName] 是否管理别人？
1. 是
2. 否

FC016 [XRName] 现在与 [XEmployer] 签了书面的劳动合同吗？
1. 是
2. 否

FC017 [XRName] 现在的劳动合同的期限是多长？
【**访员注意**：如果受访者回答是固定期限，只是记不清具体年月，请选择固定期限，对年月填“-1”。】
1. 固定期限 ______ [$hc([0,50], int, -1)$] (**FC017_1**) 年零 ______ [$hc([0,12], int, -1)$] (**FC017_2**) 个月
2. 无固定期限
3. 以完成一定工作任务为期限的劳动合同

997. 不知道
999. 拒绝回答

FC018 我记得 [XRName] 是在 [ZFD011_1] 年开始在这个 [XWorkplace] 工作的，请问正确吗？如果错误或当中中断过，请告知这次工作开始的时间。

【**访员注意**：如果受访者无法回答年月，请填“-1”。】

1. 正确
2. 错误或当中中断过，正确的工作起始时间是 ______ [hc([1950,XIWYear], *int*, −1)] (**FC018_1**) 年 ______ [hc([1, 12], *int*, −1)] (**FC018_2**) 月

999. 拒绝回答

FC019 什么时候 [XRName] 开始在这个 [XWorkplace] 工作？ ______ [hc([1950,XIWYear], *int*, −1)] (**FC019_1**) 年 ______ [hc([1, 12], *int*, −1)] (**FC019_2**) 月

【**访员注意**：如果受访者无法回答年月，请填“-1”。】

FC025 过去一年中，有几个月 [XRName] 在干这份工作？带薪休假和不扣工资的病假不需要扣除。______ [hc([1, 12], *int*, ∅)] 个月

【**访员注意**：特例：对于此工作，受访者过去一年一直处于休假状态，且不拿工资，则工作月数、每周天数、每周小时数均填“1”。】

FC026 过去一年的这些月份中，[XRName] 一般每周有几天在干这份工作？这里不算带薪休假、不扣工资的病假和休息日。______ [hc([1, 7], *int*, ∅)] 天

【**访员注意**：如果每月不足四天，每周平均不到一天，填“1”。】

FC027 过去一年的这些日子里，这份工作 [XRName] 一般每天干几个小时？工作时间不包括午休时间，但包括加班时间。______ [hc([1, 24], *int*, ∅)，sc([1, 16], *int*, ∅)] 小时

FC. 受雇工作（二）

FC032 [XRName] 的工资主要是怎么支付的？是定期支付、按项目支付、按绩效支付还是其他方式？如果是定期支付，请问是按年、月、周、日还是小时领取工资？如果单位拖欠工资奖金或者刚开始上班，请告诉我们 [XRName] 应该得到的工资情况。（选项免读）

【**访员注意**：应得而未拿到的工资指未来可以拿到，如果某项工资奖金已经没有索取的可能性，则不能算作应得工资。】

1. 按年领取
2. 按月领取
3. 按周领取
4. 按天领取
5. 小时工资
6. 按项目
7. 按绩效，包括计件工资
8. 其他，请注明 ______ (**FC032_1**)

997. 不知道
999. 拒绝回答

FC033 [XRName] 过去一年有 [FC025] 个月在干这份工作。由于 [XRName] 的工资主要是按年领的，可能还没领到手，请问对应于这 [FC025] 个月从 [XEmployer] 拿到的工资应该有多少？请把奖金

等各种收入都算在内，包括工作中获得的小费、红包、和礼品。______ [hc([0, 10000000], *int*, −1)，sc([1000, 1000000], *int*, −1)，ub([−1], [10000, 30000, 50000, 100000, 200000])] 元
【访员注意：如受访者无法回答，请填“-1”。】

FC034 上个月 [XRName] 从 [XEmployer] 拿到的工资应该有多少？[XPrefixMonthlyWage] 请把奖金等各种收入都算在内，包括工作中获得的小费、红包、和礼品。______ [hc([0, 10000000], *int*, −1)，sc([100, 100000], *int*, −1)，ub([−1], [500, 1000, 2500, 5000, 10000])] 元
【访员注意：如受访者无法回答，请填“-1”。】

FC035 [XRName] 上周从 [XEmployer] 拿到的工资应该有多少？请把奖金等各种收入都算在内，包括工作中获得的小费、红包、和礼品。______ [hc([0, 10000000], *int*, −1)，sc([100, 100000], *int*, −1)，ub([−1], [100, 300, 500, 1000, 2000])] 元
【访员注意：如受访者无法回答，请填“-1”。】

FC036 [XRName] 一般每天从 [XEmployer] 拿到多少工资？请把奖金等各种收入都算在内，包括工作中获得的小费、红包、和礼品。______ [hc([1, 10000000], *int*, −1)，sc([100, 100000], *int*, −1)，ub([−1], [20, 50, 100, 200, 500])] 元
【访员注意：如受访者无法回答，请填“-1”。】

FC037 [XRName] 一般每小时从 [XEmployer] 拿到多少工资？请把奖金等各种收入都算在内，包括工作中获得的小费、红包、和礼品。______ [hc([1, 10000000], *int*, −1)，sc([10, 100000], *int*, −1)，ub([−1], [10, 30, 50, 100, 200])] 元
【访员注意：如受访者无法回答，请填“-1”。】

FC038 那么算下来，上个月 [XRName] 总共从 [XEmployer] 拿到的工资应该有多少？[XPrefixMonthlyWage] 同样，请把奖金等各种收入都算在内，包括工作中获得的小费、红包、和礼品。______ [hc([0, 10000000], *int*, −1)，sc([100, 100000], *int*, −1)，ub([−1], [500, 1000, 2500, 5000, 10000])] 元
【访员注意：如受访者无法回答，请填“-1”。】

FC039 过去一年中，[XRName] 从 [XEmployer] 应拿到的其他所有奖金一共有多少？这里指的是不和薪酬一起按期支付的奖金，比如年终奖、过节费等。______ [hc([0, 10000000], *int*, −1)，sc([0, 1000000], *int*, −1)，ub([−1], [1000, 3000, 5000, 10000, 20000])] 元
【访员注意：如受访者无法回答，请填“-1”。】

FC040 有些人的工资拿到手前，还要缴纳个人所得税、养老保险、医疗保险、住房公积金或其他杂费。上面提到的工资奖金金额中，是否已经扣除了这些缴费？

1. 需要缴纳，已经扣除
2. 需要缴纳，没有扣除
3. 不需要缴纳税险或其他杂费

997. 不知道
999. 拒绝回答

FC041 [XPrefixFC041] 多少？
【访员注意：如果受访者报告具体的税险金额有困难，可以填报第一个选项占工资比例，精确到十位数亦可。】

1. 相当于工资的 ______ [hc([1, 50], *int*, ∅)] (**FC041_1**) %
2. ______ [hc([100, 100000], *int*, ∅)] (**FC041_2**) 元/月
3. ______ [hc([100, 100000], *int*, ∅)] (**FC041_3**) 元/年

997. 不知道

FC042 [XRName] [XEmployer] 提供了哪些福利？（可多选）每个月的福利各值多少钱？

【**访员注意**：值多少钱指的是净价值，就是福利项目的市场价格与员工实际成本之间的差值。例如员工住单位宿舍月付 500 元，同样条件宿舍的市场租金 2000 元，则其净价值为 1500 元。
如果受访者无法估算净价值，可填“-1”。】

1. 餐食：免费早、午、晚餐及餐费补贴，净价值约 ______ [hc$([10, 10000], int, -1)$，ub$([-1], [100, 500, 1000])$] (**FC042_1**) 元
2. 交通：单位配车、班车及交通费补贴，净价值约 ______ [hc$([10, 50000], int, -1)$，ub$([-1], [100, 500, 1000])$] (**FC042_2**) 元
3. 住宿：免费或租金低于市价的单位宿舍及住房补贴，净价值约 ______ [hc$([10, 100000], int, -1)$，ub$([-1], [500, 2000, 5000])$] (**FC042_3**) 元
4. 定期发放的实物、及其他补贴，总净价值约 ______ [hc$([10, 100000], int, -1)$，ub$([-1], [100, 500, 1000])$] (**FC042_4**) 元
5. 除工资奖金外，没有任何福利（排他选项）

999. 拒绝回答

[conflict$(5, 999, [5, 999]^c)$]

FD. 非农自雇工作

【**引语**：接下来我想了解一下刚才说到的那份 [XRName] [XSelfEmplType] 。】

FD002 [XRName] [XSelfEmplName] 现在的地址是？

【**访员注意**：如受访者不知道街道和社区，可酌情选择】

1. 居住地的同个村/社区：[XRResidenceFull]
2. 居住地同区县 [XRResidenceCounty] 的其他村/社区：______ (**FD002_1**) 乡/镇/街道/村/社区
3. 居住地区县以外：______ (**FD002_2**) 省/市/区县 ______ (**FD002_3**) 乡/镇/街道/村/社区
4. 以上选项均不适用的地区（港澳台及国外）

999. 拒绝回答

FD003 [XRName] [XSelfEmplName] 现在主要做什么？就是说属于哪个行业？生产什么产品或者从事什么经营活动？（例 1：制造纱质布料；例 2：销售服装）______

【**访员注意**：请按《行业与职业填写规范》的要求填写】

FD004 [XRName] [XSelfEmplName] 现在一般雇了多少人？ ______ [hc$([0, 9999], int, \varnothing)$] 人（不雇人填“0”）

FD007 过去一年中，有几个月 [XRName] 在 [XSelfEmplVerb] 这份生意？ ______ [hc$([1, 12], int, \varnothing)$] 个月

FD008 过去一年的这些月份中，[XRName] 一般每周有几天在 [XSelfEmplVerb] 这份生意？ ______ [hc$([1, 7], int, \varnothing)$] 天

【**访员注意**：如果每月不足四天，每周平均不到一天，填“1”。】

FD009 过去一年的这些日子里，这份生意 [XRName] 一般每天 [XSelfEmplVerb] 几个小时？ ______ [hc$([1, 24], int, \varnothing)$]，sc$([1, 16], int, \varnothing)$] 小时

FE. 非主要工作

【引语：刚才您说过 [XPrefixMainJob] [XRName] 有多份工作，也就是说，除了主要工作，[XRName] 还有 [XSuffixMainJob] 其他的工作。】

FE001 现在我们考虑所有其他的工作，算在一起，[XRName] 一般每周干多少天？ ______ [hc($[0,7]$, $real$, $\varnothing$)，sc($(0,7]$, $real$, $\varnothing$)] 天

FE002 对于这些“所有其他的工作”，一般 [XRName] 每天做多少小时？ ______ [hc($[1,24]$, int, $\varnothing$)，sc($[1,16]$, int, $\varnothing$)] 小时

FF. 求职与就业

FF001 过去一年中，有多少天 [XRName] 曾工作过？干农活、挣工资打工、做生意和为家庭生意帮工都算是工作。这里问的是 [XRName] 真正在干活和上班的日子，不包括休息日。 ______ [hc($[0,366]$, int, -1)，ub($[-1]$, $[10,50,100,150,250]$)] 天（如果受访者无法回答，请填“-1”。如果从没工作过，填“0”）

【访员注意：

1. 之前获知，如果受访者一年的工作天数分配保持恒定，受访者约有 [XHhldFarmDays] 天曾从事农业自雇，约有 [XMainJobDays] 天曾从事目前的主要工作，约有 [XSideJobDays] 天曾从事目前的非主要工作。这些数字仅供参考，不能直接加总。注意受访者可能会在同一天从事多种工作。不同工作的工作天数可能会有重合。[XFF001]

2. 答案精确到十位天数即可。

3. 如果发现之前各项工作时长的回答有问题，请访员回到相应的子模块进行更改。】

FF002 [XRName] 目前是否有消遣性的工作，并能从中获得少量收入？

1. 是
2. 否

FF003 过去一个月 [XRName] 是否找过工作？

1. 是
2. 否

FF004 [XRName] 计划在多大年龄时停止工作，即停止一切以挣钱为目的的活动，也不再为家庭经营活动帮工，将来也不打算从事比消遣性工作更劳累的活动？请告知大概年龄。

【访员注意：停止工作的年龄应大于等于受访者当前的年龄 [XRAge] 岁】

1. 计划在 ______ [hc($[XRAge, 120]$, int, -1)，sc($[60,100]$, int, -1)] (**FF004_1**) 岁时停止工作
2. 计划再过 ______ [hc($[0,50]$, int, -1)] (**FF004_2**) 年停止工作
3. 只要健康允许，就一直工作

997. 不知道
999. 拒绝回答

FF005 设想一下，如果没有疫情，[XRName] 可能有一个在多大年纪停止工作的想法，但是因为疫情的原因，情况可能发生了变化。那么，[XRName] 计划停止工作的时间受疫情影响是推迟了，还是提前了，还是没有变化？

【访员注意：如果有几个月的变化，不到一年，也取整为一年。如果受访者无法回答具体年数，可填“-1”。有没有疫情，只要健康允许，都准备一直工作，这种情况下选择“没有变化”。】

1. 提前了 ______ [hc([1, 50], *int*, −1)] (**FF005_1**) 年
2. 推迟了 ______ [hc([1, 50], *int*, −1)] (**FF005_2**) 年
3. 没有变化
997. 不知道
999. 拒绝回答

FG. 疫情期间工作

【引语：下面我想了解一下疫情对 [XRName] 工作的影响。】

FG001 疫情期间，有些单位采用了网络或电话会议、线上办公、线上销售等远程办公方式。[XRName] 在疫情期间是否曾远程办工，待在家里从事老板或单位的工作？
1. 是
2. 否

FG002 有一种情况，[XRName] 有没有经历过？就是疫情期间有那么一段时间 [XVCNotInQuarantine]，[XRName] 的老板或公司没开工，这样不干活还可以拿一部分工资？
【访员注意：如果受访者无法回答，请填“-1”。】
1. 有，这种情况下平均拿到的工资相当于正常工资的 ______ [hc([1, 100], *int*, −1)] (**FG002_1**) %，这样的情况共持续了 ______ [hc([1, 50], *int*, −1)] (**FG002_2**) 周
2. 有，这种情况下平均拿到的工资比正常工资还要高
3. 否，没这种情况

FG003 疫情期间，有些人受限制没法下地去干农活。设想一下，如果没有疫情，[XRName] 可能会给自家干农活，但是因为疫情的原因，情况可能发生了变化。那么，从过完年到现在，[XRName] 给自家干农活的天数受疫情影响是增加了，还是减少了，还是没有影响？干农活，就是刚才介绍的农业活动，包括农林牧渔和卖自家生产的农产品。
【访员注意：如果受访者无法回答周数，请填“-1”。】
1. 有或没有疫情都不会给自家干农活
2. 少开工了 ______ [hc([1, 40], *int*, −1)] (**FG003_1**) 周
3. 多开工了 ______ [hc([1, 40], *int*, −1)] (**FG003_2**) 周
4. 没变化
999. 拒绝回答

FG004 疫情期间，有些上班的人复工推迟或是丢了工作。再设想一下，如果没有疫情，[XRName] 可能会去挣工资打工上班，但是因为疫情的原因，情况可能发生了变化。那么，从过完年到现在，[XRName] 挣工资打工上班的天数受疫情影响是增加了，还是减少了，还是没有影响？
【访员注意：如果受访者无法回答周数，请填“-1”。】
1. 有或没有疫情都不会去挣工资打工上班
2. 少开工上班了 ______ [hc([1, 40], *int*, −1)] (**FG004_1**) 周
3. 多开工上班了 ______ [hc([1, 40], *int*, −1)] (**FG004_2**) 周
4. 没变化
999. 拒绝回答

FG005 疫情期间，有些店铺开不了门，开了门也没有生意。再设想一下，如果没有疫情，[XRName] 可能会做个体生意，但是因为疫情的原因，情况可能发生了变化。那么，从过完年到现在，

[XRName] 做生意的天数是增加了，还是减少了，还是没有影响？做生意包括自由职业和不拿工资给家庭生意帮工，不包括农业生产经营。

【**访员注意**：如果受访者无法回答周数，请填“-1”。】

1. 有或没有疫情都不会从事非农自雇工作
2. 少开工了 ______ [hc([1, 40], *int*, −1)] (**FG005_1**) 周
3. 多开工了 ______ [hc([1, 40], *int*, −1)] (**FG005_2**) 周
4. 没变化

999. 拒绝回答

FG008 疫情期间，有些人只上半天班，如果没有疫情，他们可能就是上全天班。对于 [XRName] 来说，因为疫情的原因，情况可能也发生了变化。那么，从过完年到现在，在开工的月份，疫情使 [XRName] 每周的工作小时数增加了，还是减少了，还是没有影响？

【**访员注意**：这里不应考虑受访者不工作的日子，天数减少和小时数减少不能重复计算。如果疫情的影响导致受访者整天没上班，这种情况应该记为天数的减少，记录到前面三道题中。如果受访者无法回答具体小时数，请填“-1”。】

1. 没有变化
2. 减少了 ______ [hc([1, 40], *int*, −1)] (**FG008_1**) 个小时
3. 增加了 ______ [hc([1, 40], *int*, −1)] (**FG008_2**) 个小时
4. 过完年没工作过

999. 拒绝回答

FG009 疫情期间 [XRName] 是否曾领取过失业保险金？

1. 是
2. 否

FG010 [XRName] 领了 ______ [hc([1, 10], *int*, −1)] (**FG010_1**) 个月？每个月领取的失业保险金平均来说是 ______ [hc([1, 10000], *int*, −1)] (**FG010_2**) 元。

【**访员注意**：如果受访者无法回答，请填“-1”。】

FH. 退休手续

FH001 [XRName] 是否已经办理了退休手续，包括提前退休和内退？退休是指从政府和企事业单位退休，也包括参加了基本养老保险的灵活就业人员所办理的退休。

【**访员注意**：开始领取城居保、新农保、城乡居民养老保险金，不算是办理了退休手续。】

1. 是
2. 否

FH002 [XRName] 办理的是正常退休、提前退休、还是先内退然后办的正式退休或者内退等将来再办正式退休？（选项免读）

1. 正常退休
2. 提前退休
3. 先内退然后又正式退休
4. 内退但目前还没有办理正式退休

FH003 您的正式退休手续是在哪年哪月办理的？______ [hc([1950,XIWYear], *int*, −1)，sc([ZIWYear,XIWYear], *int*, −1)] (**FH003_1**) 年 ______ [hc([1, 12], *int*, −1)] (**FH003_2**) 月

【**访员注意**：如果受访者无法回答，请填“-1”。】

FH004 您将会在哪年哪月办理正式退休手续? ______ [hc([XIWYear, 2050], *int*, −1)] (**FH004_1**) 年 ______ [hc([1, 12], *int*, −1)] (**FH004_2**) 月

【**访员注意**: 如果受访者无法回答, 请填 "-1"。】

辅助变量定义

XWorking 是否就业

```
if (equal("FA001", "1")  || equal("FA004", "1") || equal("FA007", "1") || equal("FA008", "1")) {
    add("XWorking", "1")
} else {
    add("XWorking", "0")
}
```

XEmployed 是否自雇

```
if (equal("FA010", "2") || equal("FA010", "3") || equal("FA011", "2") || equal("FA011", "3")) {
    add("XEmployed", "0")
} else if (equal("FA010", "1") || equal("FA010", "4") || equal("FA011", "1") || selected("FA002", "2")) {
    add("XEmployed", "1")
}
```

XFGSample 是否问疫情期间工作

```
if (equal("XWorking","1")) {    //目前有工作
    add("XFGSample","1")
} else if (equal("FF003","1")) {    //目前在找工作
    add("XFGSample","1")
} else if (equal("FF005","1") || equal("FF005","2")) {  //疫情改变了退休预期
    add("XFGSample","1")
} else if (equal("FA016_1","2020")) {   // 停止工作时间在1月1号后
    add("XFGSample","1")
} else {
    add("XFGSample","0")
}
```

XPrefixMainJob 主要工作提问前缀

```
if (selected("FA002","1")) {
    add("XPrefixMainJob", "除去给自家干农活, ")
}
```

XSuffixMainJob 主要工作提问后缀

```
if (selected("FA002","2")) {
    add("XSuffixMainJob", "挣工资干农活, ")
}
```

XEmployer 工资发放单位

```
if (equal("WC001","2")) {
    add("XEmployer", "派遣单位")
} else if (equal("WC001","3")) {
    add("XEmployer", "包工头")
} else if (equal("WC001","1") && greater("WC002","4")) {
    add("XEmployer", "雇主")
} else {
    add("XEmployer", "工作单位")
}
```

XWorkplace 工作单位

```
if (greater("WC002","4"))) {
    add("XWorkplace", "雇主")
} else {
    add("XWorkplace", "工作单位")
}
```

XSelfEmplName 非农自雇/无偿家庭帮工工作名称

```
if (equal("FA010","2") || equal("FA011","2") ) {
    add("XSelfEmplName","的生意")
    add("XSelfEmplType","个体/私营生意")
    add("XSelfEmplVerb","做")
} else {
    add("XSelfEmplName","帮工的家庭生意")
    add("XSelfEmplType","不拿工资帮工的家庭生意")
    add("XSelfEmplVerb","帮工")
}
```

XSelfEmplType 非农自雇/无偿家庭帮工生意性质

see above

XSelfEmplVerb 非农自雇/无偿家庭帮工生意动词

see above

XFC002 WC002 措辞

```
if (equal("FC001","2")) {
    add("XFC002", "注意，是用人单位，不是派遣单位")
} else if (equal("FC001","3")) {
    add("XFC002", "注意，是用人单位，不是包工头")
}
```

XFC003 WC003 措辞

```
if (greater("FC002","4")) {
    add("XFC003", "工作单位的名称是什么")
} else {
    add("XFC003", "雇主怎么称呼")
}
```

XWorkJustStarted 当前受雇工作是否开始不到一年或一月

```
if ((equal("FC019_1", "2020") && greater("FC019_2", value("XIWMonth")-2)) || (equal("FC018_1", "2020") &&
↪ greater("FC018_2", value("XIWMonth")-2))) {
    add("XWorkJustStarted", "1")    // 不到一个月
} else if ((equal("FC019_1", "2020") || (equal("FC019_1", "2019") && greater("FC019_2",
↪ value("XIWMonth")-1))) || (equal("FC018_1", "2020") || (equal("FC018_1", "2019") && greater("FC018_2",
↪ value("XIWMonth")-1)))) {
    add("XWorkJustStarted", "2")    // 不到一年
} else {
    add("XWorkJustStarted", "0")    // 一年前
}
```

XMainJobDays 主要工作天数

```
if (equal("XEmployed","1")) {
    add("XMainJobDays", value("FC026")*52)
} else if (equal("XEmployed","1")) {
    add("XMainJobDays", value("FD008")*52)
} else {
    add("XMainJobDays", "0")
}
```

XHhldFarmDays 农业自雇天数

```
add("XHhldFarmDays", value("FB006")*value("FB005")*4)
if (empty("XHhldFarmDays")) {
add("XHhldFarmDays", "0")
}
```

XSideJobDays 非主要工作天数

```
add("XSideJobDays", value("FE001")*52)
if (empty("XSideJobDays")) {
    add("XSideJobDays", "0")
}
```

XFF001 FF001 访员注意

```
if (equal("XWorking","0")) {
    add("XFF001", "另外要注意到受访者虽然目前不在工作，但过去一年的早期可能曾从事过非农工作。")
} else if (empty("XEmployed")) {
    add("XFF001", "另外要注意到受访者虽然目前没在从事非农工作，但过去一年的早期可能曾从事过非农工作。")
} else if (greater("12", "FC025")) {
    add("XFF001", "另外要注意到受访者虽然目前在从事非农工作，但过去一年从事该工作的时间不到12个月，
    ↪ 其他时间可能没在工作或者在从事其他不一样的工作。")
}
```

XGetJob 无工作 → 工作

```
if (equal("ZWorking","0") && equal("XWorking","1")) {
    add("XGetJob", "1")
} else {
    add("XGetJob", "0")
}
```

XQuitJob 工作 → 无工作

```
if (equal("ZWorking","1") && equal("XWorking","0")) {
    add("XQuitJob", "1")
}else {
    add("XQuitJob", "0")
}
```

XPrefixMonthlyWage 对刚开始上班的人，问上月工资时的措辞

```
if (equal("XWorkJustStarted", "1")) {
    add("XPrefixMonthlyWage", "刚开始上班，如果还没领到过工资，请告知下个月会拿到多少工资。")
}
```

XPrefixFC041 FC041 措辞

```
if (equal("FC040", "1")) {
    add("XPrefixFC041", "那扣了")
} else {
    add("XPrefixFC041", "那工资拿到手，还会再扣")
}
```

XWorkTypeFarmEmployed 主要工作农业受雇选项

```
if (selected("FA002","2")) {
    add("XSuffixMainJob", "农业受雇")
}
```

This page intentionally left blank

G　收入与支出

G1 家户收入与支出

G1. 代理模式确认

proxy_12 访员记录，对于家户收入以及住房模块是否使用代理问卷模式？

1. 是
2. 否

GB. 其他家户成员收入

GB001 以下哪个家户成员是家庭财务受访者？财务受访者须熟悉家庭的经济状况。

【**访员注意**：如果家户支出和收入模块要使用代理，请申请/使用已有的 [XFLHHFinancialNameList] 的个人代理，然后在本题选择 [XFLHHFinancialNameList] 。】

1-25. [XHHMemberName[i]]
26. [XMainR]
27. [XMainRS]

【**引语**：请确保没有其他人在场。下面我们想问问您家其他家户成员的收入情况。我们将会对您的回答严格保密，并且仅用于学术研究。】

GB002[i] 过去一年，[XHHMemberName[i]] 有没有领工资，包括奖金、各种补贴，不包括退休、退职和内退工资？这里的工资来自所有的受聘工作。

1. 有
2. 没有
997. 不知道
999. 拒绝回答

GB003[i] 过去一年，[XHHMemberName[i]] 一共领了多少钱？ ______ [hc$((0,\infty),int,-1)$，sc$((0,240000],int,-1)$，ub$([-1],[5000,10000,30000,50000,100000])$] 元

【**访员注意**：如果受访者拒绝回答或者忘记了，填入“-1”。】

GB004[i] 上面提到的 [XHHMemberName[i]] 的工资有没有扣除各类保险、所得税、住房公积金或其他杂费？

1. 有
2. 没有
997. 不知道
999. 拒绝回答

GB005[i] [XHHMemberName[i]] [XGB005Text[i]] 被扣除的或上交的个人所得税、各类保险、住房公积金或其他杂费一共是多少元？

【**访员注意**：如果受访者拒绝回答或者忘记了，选第一个选项，填入“-1”。】

1. ______ [hc$((0,\infty),int,-1)$，sc$((0,10000],int,-1)$，ub$([-1],[300,500,1000,2000,3000])$] (**GB005_1[i]**) 元/月
2. ______ [hc$((0,\infty),int,\varnothing)$，sc$((0,100000],int,\varnothing)$] (**GB005_2[i]**) 元/年
3. 或相当于工资的 ______ [hc$((0,100],real,\varnothing)$] (**GB005_3[i]**) %
4. 没有，0 元

GB006[i] 过去一年，[XHHMemberName[i]] 有没有领到下列转移支付收入？注意的是，疫情发生后的，个人得到的疫情补贴也属于转移支付收入，故须在此填写（可多选）。

【访员注意：如果受访者拒绝回答或者忘记了，填入“-1”。】

1. 退休金或养老金，包括政府机关和事业单位退休金，企业职工基本养老保险，企业补充养老保险，退职或内退补偿金，农村或城乡或城镇居民养老保险，商业养老保险，人寿保险，征地养老保险等，领了 ______ [hc$((0,\infty),int,-1)$，sc$((0,100000],int,-1)$] (**GB006_1[i]**) 元
2. 失业补助，领了 ______ [hc$((0,\infty),int,-1)$，sc$((0,50000],int,-1)$] (**GB006_2[i]**) 元
3. 养老卡或养老券，领了 ______ [hc$((0,\infty),int,-1)$，sc$((0,50000],int,-1)$] (**GB006_3[i]**) 元
4. 高龄老人养老补助，领了 ______ [hc$((0,\infty),int,-1)$，sc$((0,50000],int,-1)$] (**GB006_4[i]**) 元
5. 工伤保险金包括误工补贴、伤残补助等，领了 ______ [hc$((0,\infty),int,-1)$，sc$((0,50000],int,-1)$] (**GB006_5[i]**) 元
6. 独生子女老年补助，领了 ______ [hc$((0,\infty),int,-1)$，sc$((0,50000],int,-1)$] (**GB006_6[i]**) 元
7. 医疗救助，领了 ______ [hc$((0,\infty),int,-1)$，sc$((0,100000],int,-1)$] (**GB006_7[i]**) 元
8. 政府给个人的其他补助，不包括低保、五保、特困和贫困补助，请注明 ______ (**GB006_8_1[i]**)，领了 ______ [hc$((0,\infty),int,-1)$，sc$((0,100000],int,-1)$] (**GB006_8[i]**) 元
9. 社会给个人的其他转移支付收入，如社会捐助等，请注明 ______ (**GB006_9_1[i]**)，领了 ______ [hc$((0,\infty),int,-1)$，sc$((0,100000],int,-1)$] (**GB006_9[i]**) 元
10. 以上均没有

[conflict$(10,[10]^c)$]

GB007[i] 考虑一下，如果没有疫情，[XHHMemberName[i]] 可能会有一些工资收入，但是因为疫情的原因，情况可能发生变化。那么，从春节到现在，疫情使 [XHHMemberName[i]] 工资收入增加了，还是减少了，或者没有影响？

【访员注意：如果受访者拒绝回答或者忘记了，填入“-1”。】

1. 减少，减少了 ______ [hc$((0,\infty),int,-1)$，sc$((0,50000],int,-1)$，ub$([-1],[1000,3000,5000,10000,20000])$] (**GB007_1[i]**) 元
2. 增加，增加了 ______ [hc$((0,\infty),int,-1)$，sc$((0,50000],int,-1)$，ub$([-1],[1000,3000,5000,10000,20000])$] (**GB007_2[i]**) 元
3. 没有变化

GB008[i] 过去一年，[XHHMemberName[i]] 有没有领到下列转移支付收入？注意的是，疫情发生后的，个人得到的疫情补贴也属于转移支付收入，故须在此填写（可多选）。

【访员注意：如果受访者拒绝回答或者忘记了，填入“-1”。】

7. 医疗救助，领了 ______ [hc$((0,\infty),int,-1)$，sc$((0,50000],int,-1)$] (**GB008_7[i]**) 元
8. 政府给个人的其他补助，不包括低保、五保、特困和贫困补助，请注明 ______ (**GB008_8_1[i]**)，领了 ______ [hc$((0,\infty),int,-1)$，sc$((0,50000],int,-1)$] (**GB008_8[i]**) 元
9. 社会给个人的其他转移支付收入，如社会捐助等，请注明 ______ (**GB008_9_1[i]**)，领了 ______ [hc$((0,\infty),int,-1)$，sc$((0,50000],int,-1)$] (**GB008_9[i]**) 元
10. 以上均没有

[conflict$(10,[10]^c)$]

GC. 家庭农业收入

【引语：下面，我们将问一些有关您家农业收入与支出的问题。个人问卷里农业工作是关于受访者个人的，而这里是家户层面的，即使受访者不从事农业活动，但是家户有成员从事农业活动也算家户从事农业活动。】

GC001 过去一年，[XMainR] 家的家户成员，包括 [XFLHHNameList]，有没有从事种地、管理果树、采集农林产品、养鱼、打鱼、养牲畜等农业活动，或者去市场销售自家生产的农产品？

1. 有
2. 没有

GC002 过去一年，从事这些农业活动的家户成员有哪些？（可多选）

1-25. [XHHMemberName[i]]
26. [XMainR]
27. [XMainRS]

GC003 [XMainR] 家的家户成员，包括 [XFLHHNameList]，过去一年是否从事了种植业或林业，包括种植花木、蔬菜和各类农作物，种植蘑菇、木耳等林下作物，种植茶叶，或野生农林产品的采集？

1. 是
2. 否

GC004 过去一年，[XMainR] 家从以上种植业/林业生产以及野生农林产品的采集中，一共得到多少净收入？是赚钱了还是亏损了？净收入为产出的价值减去投入。产出包括出售、自己消费和储存的部分，投入包括种子、化肥、农药、灌溉、雇工、租用机器、塑料薄膜、租用别人土地、燃料、运输费、加工费、包装费，管理费等。

【访员注意：如果受访者拒绝回答或者忘记了，填入“-1”。】

1. 赚钱，净收入为 ______ [hc$((0,\infty),int,-1)$，sc$((0,100000],int,-1)$，ub$([-1],[2000,5000,10000,50000,100000])$] (**GC004_1**) 元
2. 亏损，损失了 ______ [hc$((0,\infty),int,-1)$，sc$((0,100000],int,-1)$，ub$([-1],[2000,5000,10000,50000,100000])$] (**GC004_2**) 元
3. 不赚不赔

GC005 [XMainR] 家的家户成员，包括 [XFLHHNameList]，过去一年养过牲畜或者水产品，或捕获过野生动物或水产品吗？牲畜包括家禽、家畜（如鸡、鸭、牛、猪和羊）等，水产品包括鱼等。

1. 是
2. 否

GC006 过去一年，[XMainR] 家，从牲畜和水产品养殖活动以及野生动物和水产品捕捞活动中，一共得到多少净收入？是赚钱了还是亏损了？净收入为产出的价值减去投入。产出包括最终产品以及副产品在市场上出售的收入以及自家消费那部分的价值，投入包括喂养费、医药费、放牧费、畜舍栅栏费、雇工费等。

【访员注意：如果受访者拒绝回答或者忘记了，填入“-1”。】

1. 赚钱，净收入为 ______ [hc$((0,\infty),int,-1)$，sc$((0,100000],int,-1)$，ub$([-1],[2000,5000,10000,50000,100000])$] (**GC006_1**) 元
2. 亏损，损失了 ______ [hc$((0,\infty),int,-1)$，sc$((0,100000],int,-1)$，ub$([-1],[2000,5000,10000,50000,100000])$] (**GC006_2**) 元
3. 不赚不赔

GC007 考虑一下，如果没有疫情，[XMainR] 家的家户成员，包括 [XFLHHNameList]，可能会有一些农业收入，但是因为疫情的原因，情况可能发生变化。那么，从春节到现在，疫情使 [XMainR]

家农业生产的净收入增加了，还是减少了，或者没有影响？

【访员注意：如果受访者拒绝回答或者忘记了，填入“-1”。】

1. 减少，减少了 ______ [hc$((0,\infty), int, -1)$, sc$((0,10000], int, -1)$, ub$([-1],[200,500,1000,5000,10000])$] **(GC007_1)** 元
2. 增加，增加了 ______ [hc$((0,\infty), int, -1)$, sc$((0,10000], int, -1)$, ub$([-1],[200,500,1000,5000,10000])$] **(GC007_2)** 元
3. 没有变化

GD. 个体经营和私营企业收入

GD001 过去一年，[XMainR] 家的家户成员，包括 [XFLHHNameList]，是否从事某些个体经营或开办私营企业？

【访员注意：受访者在个人问卷询问过个体经营问题，但那是个人层面的。家户层面的个体经营和私营企业，只要是有家户成员参与即可，范围比个人问卷的大。】

1. 是
2. 否

GD002 过去一年，[XMainR] 家的家户成员，包括 [XFLHHNameList]，从事几项个体经营活动或开办几家私营企业？ ______ [hc$([1,25], int, \varnothing)$] 项

GD003 过去一年，从事这 [GD002] 项个体经营或私营企业的家户成员有哪些？（可多选）

1-25. [XHHMemberName[i]]

26. [XMainR]

27. [XMainRS]

GD004 您能否精确估计一下 [XMainR] 家过去一年从这 [GD002] 项经营活动净赚或亏损多少钱？如果经营活动有非家户成员参与，只计算家户成员的净收入。别忘了考虑下列各种成本：固定资本的折旧、能源、住房和设备租用费、原材料、交通费、营销、工资、税收和杂费，过去一年发生的固定资本投资不要算到成本里。

【访员注意：如果受访者拒绝回答或者忘记了，填入“-1”。】

1. 赚钱，净赚了 ______ [hc$((0,\infty), int, -1)$, sc$((0,500000], int, -1)$, ub$([-1],[5000,10000,50000,100000,200000])$] **(GD004_1)** 元
2. 赔钱，赔了 ______ [hc$((0,\infty), int, -1)$, sc$((0,500000], int, -1)$, ub$([-1],[5000,10000,50000,100000,200000])$] **(GD004_2)** 元
3. 不赚不赔

999. 拒绝回答

GD005 考虑一下，如果没有疫情，[XMainR] 家的家户成员，包括 [XFLHHNameList]，可能会有一些个体经营和私营企业收入，但是因为疫情的原因，情况可能发生变化。那么，从春节到现在，疫情使 [XMainR] 家个体经营和私营企业的净利润增加了，还是减少了，或者没有影响？

【访员注意：如果受访者拒绝回答或者忘记了，填入“-1”。】

1. 减少，减少了 ______ [hc$((0,\infty), int, -1)$, sc$((0,50000], int, -1)$, ub$([-1],[500,1000,5000,10000,20000])$] **(GD005_1)** 元
2. 增加，增加了 ______ [hc$((0,\infty), int, -1)$, sc$((0,50000], int, -1)$, ub$([-1],[500,1000,5000,10000,20000])$] **(GD005_2)** 元
3. 没有变化

GD006 因为疫情的影响，[XMainR] 家的个体经营和私营企业状况是否发生了变化?

1. 我家疫情前后都没有有个体经营和私营企业
2. 我家疫情前后都有个体经营和私营企业
3. 我家疫情前没有个体经营和私营企业，疫情发生后有了
4. 我家疫情前有个体经营和私营企业，疫情发生后关闭了

GD007 因为疫情的影响，从春节到现在，[XMainR] 家的个体经营和私营企业是否得到下列补贴或者补助?（可多选）

【**访员注意**：如果受访者拒绝回答或者忘记了，填入“-1”。】

1. 政府贷款，贷款了 ______ [hc$((0,\infty), int, -1)$，sc$((0,500000], int, -1)$] (**GD007_1**) 元，利息为 ______ [hc$([0,100], real, -1)$] (**GD007_2**) % 每年
2. 厂房/店面租金减免，从春节到现在一共减少了 ______ [hc$((0,\infty), int, -1)$，sc$((0,50000], int, -1)$] (**GD007_3**) 元
3. 税收减免，从春节到现在一共减免了 ______ [hc$((0,\infty), int, -1)$，sc$((0,50000], int, -1)$] (**GD007_4**) 元
4. 其他，请说明 ______ (**GD007_5**)，从春节到现在一共得到了或减免了 ______ [hc$((0,\infty), int, -1)$，sc$((0,50000], int, -1)$] (**GD007_6**) 元
5. 以上都没有

[conflict$(5,[5]^c)$]

GE. 家户公共转移支出收入

GE001 [XMainR] 家或者 [XMainR] 家的家户成员，包括 [XFLHHNameList]，是否曾经是或现在是五保户/老人、低保户/老人、特困户/老人或贫困户?（可多选）

1. 五保户/老人
2. 低保户/老人
3. 特困户/老人
4. 建档立卡贫困户
5. 其他类型贫困户，请注明 ______ (**GE001_1**)
6. 以上都没有

[conflict$(6,[6]^c)$]

GE002$[i]$ [XMainR] 家或其家户成员，曾经是还是现在是 [XPoorHHName$[i]$]？从哪年开始的? 如果中间中断过，请按最久远那个开始时间做开始时间。

1. 现在是，开始时间是 ______ [hc$((1980,2020], int, \varnothing)$] (**GE002_1[i]**) 年
2. 曾经是现在不是了，开始时间是 ______ [hc$((1980,2020], int, \varnothing)$] (**GE002_2[i]**) 年

GE003$[i]$ 下面的哪些家户成员从政府领取了 [XPoorHHName$[i]$] 补助? 可能有非家户成员和家户成员一起从政府领取了该项补助，这里不考虑非家户成员（可多选）。

1-25. [XHHMemberName$[i]$]
26. [XMainR]
27. [XMainRS]

GE004$[i]$ 过去一年，[XMainR] 家的家户成员，包括 [XFLHHNameListPoor$[i]$] 从刚才谈到的 [XPoorHHName$[i]$] 一共拿到了多少补助? 如果该项补助有非家户成员共同领取，只需计算家户成员那部分。实物需折算成现金。 ______ [hc$([0,\infty), int, -1)$，sc$((0,20000], int, -1)$，ub$([-1],[500,1000,5000,10000,20000])$] 元

【访员注意：如果受访者拒绝回答或者忘记了，填入“-1”。】

GE006 [XMainR] 家过去一年有没有收到下列家户层面的政府补助，社会捐助或者补偿？不包括之前谈到的五保户、低保户、特困户户或贫困户补助。注意的是，疫情发生后的，家户层面得到的疫情补贴也属于转移支付收入，故须在此填写（可多选）。

【访员注意：如果受访者拒绝回答或者忘记了，填入“-1”。】

1. 退耕还林：有多少？______ [hc$((0,\infty),int,-1)$，sc$((0,20000],int,-1)$] (**GE006_1**) 元
2. 农业补助：有多少？______ [hc$((0,\infty),int,-1)$，sc$((0,20000],int,-1)$] (**GE006_2**) 元
3. 工伤人员供养直系亲属抚恤金：有多少？______ [hc$((0,\infty),int,-1)$，sc$((0,20000],int,-1)$] (**GE006_3**) 元
4. 重大灾害后，政府的捐助和补助，包括救济金、赈灾款等：加上实物救助的价值一共有多少？______ [hc$((0,\infty),int,-1)$，sc$((0,20000],int,-1)$] (**GE006_4**) 元
5. 社会捐助和补助，包括食品、衣服，书包和众筹医疗费等：加上实物救助的价值一共有多少？______ [hc$((0,\infty),int,-1)$，sc$((0,20000],int,-1)$] (**GE006_5**) 元
6. 征地补偿金：有多少？______ [hc$((0,\infty),int,-1)$，sc$((0,20000],int,-1)$] (**GE006_6**) 元
7. 住房拆迁补偿：有多少？______ [hc$((0,\infty),int,-1)$，sc$((0,20000],int,-1)$] (**GE006_7**) 元
8. 其他补助，请注明______ (**GE006_8_1**)：有多少？______ [hc$((0,\infty),int,-1)$，sc$((0,20000],int,-1)$] (**GE006_8**) 元
9. 没有收到任何政府和社会的补助和捐助

[conflict$(9,[9]^c)$]

GE007 [XMainR] 家过去一年有没有得到生产性保险赔付？得到多少？（如果没有，填 0）生产性保险赔付包括农业保险赔付等。______ [hc$([0,\infty),int,-1)$，sc$([0,20000],int,-1)$] 元

【访员注意：如果受访者拒绝回答或者忘记了，填入“-1”。】

【引语：这部分询问家户及其成员从公共机构所得的各种转移支付收入。公共转移支付具有福利支出的性质，如政府给五保户和特困户的补助金等。】

GE008 因为疫情的影响，[XMainR] 家以及 [XMainR] 家的家户成员，包括 [XFLHHNameList]，是否得到的政府给予的疫情补助？包括政府给家户的农业疫情补贴，不包括自己家个体经营/私营企业得到的疫情补贴，如店面/厂房租金的减少以及税收减免。实物补贴需折算成现金。

【访员注意：如果受访者拒绝回答或者忘记了，填入“-1”。】

1. 是，各种疫情补贴一共有______ [hc$((0,\infty),int,-1)$，sc$((0,20000],int,-1)$，ub$([-1],[500,1000,5000,10000,20000])$] (**GE008_1**) 元
2. 否

GE009 [XMainR] 家是否在住房屋顶、农业大棚或其他地方铺设太阳能电池板，从而发电给自家用，多余的电量卖给国家电网？

1. 是
2. 否

GE010 [XMainR] 家什么时候铺设的太阳能电池板？______ [hc$((1990,2020],int,-1)$] 年

【访员注意：如果受访者拒绝回答或者忘记了，填入“-1”。】

GE011 过去一年，[XMainR] 家从光伏发电中得到多少收入？包括自家消费的电量和出售给国家电网的电量的价值。______ [hc$([0,\infty),int,-1)$，sc$((0,20000],int,-1)$] 元

【访员注意：如果受访者拒绝回答或者忘记了，填入“-1”。】

GE012 [XMainR] 家就是否有集体分配的土地？[XMainR] 家是否出租土地给他人？如果出租的话，过去一年的租金有多少？如果没有土地或者没有出租，请填“0”。______ [hc$([0,\infty),int,-1)$，sc$([0,20000],int,-1)$，ub$([-1],[500,1000,5000,10000,20000])$] 元

【访员注意：如果受访者拒绝回答或者忘记了，填入“-1”。】

GE013 [XFamilyRAndS] 是否有房产？[XFamilyRAndS] 名下的房产出租了吗？过去一年得到的租金有多少？如果没有房产或者没有出租，请填“0”。如果房产多人所有，请根据产权计算 [XFamilyRAndS] 应该分到的租金。______ [hc$([0,\infty),int,-1)$，sc$([0,20000],int,-1)$，ub$([-1],[500,1000,5000,10000,20000])$] 元

【访员注意：如果受访者拒绝回答或者忘记了，填入“-1”。】

GE014 除了出租房屋或者土地，过去一年 [XMainR] 家从出租其他家庭资产，如树木、固定资本的使用、耐用品或者牲畜，收取了多少租金？

【访员注意：如果受访者拒绝回答或者忘记了，填入“-1”。】

1. 有出租，过去一年的租金为 ______ [hc$([0,\infty),int,-1)$，sc$((0,20000],int,-1)$] **(GE014_1)** 元
2. 没有出租

997. 不知道
999. 拒绝回答

GE015[i] 您说 [XMainR] 家或其家户成员曾经是 [XPoorHHName[i]]，开始时间是 [GE002_2[i]]，那么结束时间是什么时候？______ [hc$([$GE002_2[i]$,2020],int,\varnothing)$] 年

GF. 家户生活支出

【引语：请大声念出“本部分是所有家户成员的生活支出，包括 [XFLHHNameList]。”】

GF001 平均而言，[XMainR] 家的家户成员，包括 [XFLHHNameList]，一个月一共花费多少钱？包括房租、食物、衣服、通讯支出、水电费、燃料费、服务支出、娱乐支出、日用品和医疗支出等。______ [hc$((0,\infty),int,\varnothing)$，sc$((0,20000],int,\varnothing)$] 元

GF002 我们想知道 [XMainR] 家最近一周的食品支出，您负责为家里购买食品吗？

1. 是
2. 否

GF003 谁负责为 [XMainR] 家购买食品？

1-25. [XChildAliveName[i]]
26-35. [XHHOtherMemberName2[i]]
36. [XMainR]
37. [XMainRS]
38. 保姆
39. 邻居
40. 其他，请注明 ______ **(GF003_1)**

【引语: 如果可能的话，由负责为该家庭购买食品的人来回答 GF004-GF010。】

GF004 最近一周，不包括客人，[XMainR] 家里一般有几口人吃饭？______ [hc$([0,\infty),int,\varnothing)$，sc$([1,10],int,\varnothing)$] 人

GF005 最近一周，[XMainR] 家的客人在您家吃了几顿饭，按人次计算？______ [hc$([0,\infty), int, \emptyset)$，sc$([0,100], int, \emptyset)$] 人次

【**引语**：下面的一系列问题是关于 [XMainR] 家的生活支出的。这些支出包括所有家户成员的生活支出。只要是家户成员，其在外的支出也须包括在内，如住宿费和伙食费等。】

GF006 最近一周，[XMainR] 家花了多少钱购买食品，不包括摆酒、办酒席、外出就餐、购买香烟、酒水等？ ______ [hc$([0,\infty), int, \emptyset)$，sc$((0,6000], int, \emptyset)$] 元

GF007 [XMainR] 家自己生产农产品吗，包括粮食作物、肉类、蛋类、水产品、油、蔬菜水果、烟酒、饮料及乳制品、加工食品、调料等？

1. 生产
2. 不生产

GF008 最近一周，[XMainR] 家里人消费的自家生产的农产品在市场上卖的话值多少钱？ ______ [hc$([0,\infty), int, \emptyset)$，sc$((0,6000], int, \emptyset)$] 元

GF009 最近一周，[XMainR] 家的家户成员，包括 [XFLHHNameList]，花了多少钱外出就餐，不包括摆酒和办酒席？ ______ [hc$([0,\infty), int, \emptyset)$，sc$([0,3000], int, \emptyset)$] 元

GF010 最近一周，[XMainR] 家的家户成员，包括 [XFLHHNameList]，花了多少钱购买香烟、酒水等？ ______ [hc$([0,\infty), int, \emptyset)$，sc$([0,3000], int, \emptyset)$] 元

GF011 下面我们想了解 [XMainR] 家，家户成员包括 [XFLHHNameList]，过去一个月在以下各项消费中的支出。

1. 邮电、通讯支出，包括电话、手机、上网、邮寄等 ______ [hc$([0,\infty), int, -1)$，sc$((0,5000], int, -1)$] (**GF011_1**) 元
2. 水费、电费 ______ [hc$([0,\infty), int, -1)$，sc$((0,5000], int, -1)$] (**GF011_2**) 元
3. 燃料费，包括煤炭、煤制品、柴草、木炭、天然气、液化气等 ______ [hc$([0,\infty), int, -1)$，sc$((0,5000], int, -1)$] (**GF011_3**) 元
4. 保姆、小时工、佣人等的支出 ______ [hc$([0,\infty), int, -1)$，sc$((0,5000], int, -1)$] (**GF011_4**) 元
5. 在当地的交通费 ______ [hc$([0,\infty), int, -1)$，sc$((0,5000], int, -1)$] (**GF011_5**) 元
6. 日用品，包括洗漱用品、家居用品、厨卫用品、装饰用品等 ______ [hc$([0,\infty), int, -1)$，sc$((0,5000], int, -1)$] (**GF011_6**) 元
7. 文化娱乐支出，包括书报杂志、光盘、影剧票、歌舞厅和网吧 ______ [hc$([0,\infty), int, -1)$，sc$((0,5000], int, -1)$] (**GF011_7**) 元

【**访员注意**：没有相应项支出用“0”元表示，记不清则请受访者估计一个数字。如果受访者拒绝回答或者忘记了，填入“-1”。】

GF012 [XMainR] 家雇佣的保姆/小时工/佣人会照顾以下人员吗？（可多选）

1. 家户里的老人
2. 家户里的小孩
3. 其他，请注明 ______ (**GF012_1**)
4. 以上都不是

[conflict$(4, [4]^c)$]

GF013 下面我们想了解 [XMainR] 家，家户成员包括 [XFLHHNameList]，过去一年在以下各项消费中的支出。

1. 衣着消费 ______ [hc$([0,\infty), int, -1)$，sc$([0,100000], int, -1)$] (**GF013_1**) 元

2. 家庭的旅行支出，包括在外地工作往返老家的费用，旅游的车费和旅馆费等 ______ [hc$([0,\infty), int, -1)$，sc$([0,100000], int, -1)$] **(GF013_2)** 元
3. 家庭的取暖费支出，指集中供暖 ______ [hc$([0,\infty), int, -1)$，sc$([0,100000], int, -1)$] **(GF013_3)** 元
4. 家具、耐用消费品及电器的购买、维修及配件费用。家具、耐用消费品及电器包括电冰箱、洗衣机、电视、电脑和高档乐器如钢琴等。______ [hc$([0,\infty), int, -1)$，sc$([0,100000], int, -1)$] **(GF013_4)** 元
5. 教育和培训支出，包括学杂费、培训费等 ______ [hc$([0,\infty), int, -1)$，sc$([0,100000], int, -1)$] **(GF013_5)** 元
6. 医疗支出，包括直接或间接。注：间接医疗支出，指因为医疗而产生的交通费、营养费、家人陪护花费等。不包括医保已经赔付部分 ______ [hc$([0,\infty), int, -1)$，sc$([0,100000], int, -1)$] **(GF013_6)** 元
7. 保健费用，包括健身锻炼及产品器械、保健品等 ______ [hc$([0,\infty), int, -1)$，sc$([0,100000], int, -1)$] **(GF013_7)** 元
8. 美容支出，包括化妆品、美容护理、按摩等 ______ [hc$([0,\infty), int, -1)$，sc$([0,100000], int, -1)$] **(GF013_8)** 元
9. 汽车的购买、维修及配件费用 ______ [hc$([0,\infty), int, -1)$，sc$([0,100000], int, -1)$] **(GF013_9)** 元
10. 各种交通工具和通讯工具的购买、维修及配件费用。注：交通工具包括自行车和电动自行车但不包括汽车，通讯工具包括电话和手机 ______ [hc$([0,\infty), int, -1)$，sc$([0,100000], int, -1)$] **(GF013_10)** 元
11. 物业费，包括车位费 ______ [hc$([0,\infty), int, -1)$，sc$([0,100000], int, -1)$] **(GF013_11)** 元
12. 上交给政府相关部门的税费和杂费，不包括所得税 ______ [hc$([0,\infty), int, -1)$，sc$([0,100000], int, -1)$] **(GF013_12)** 元
13. 社会捐助支出，包括现金，食品、衣服等 ______ [hc$([0,\infty), int, -1)$，sc$([0,100000], int, -1)$] **(GF013_13)** 元
14. 房子或者床位的租金，包括家户成员在外的住宿费，如住校宿舍费，不包括旅游的宾馆费 ______ [hc$([0,\infty), int, -1)$，sc$([0,100000], int, -1)$] **(GF013_14)** 元
15. 摆酒和办酒席的支出 ______ [hc$([0,\infty), int, -1)$，sc$([0,100000], int, -1)$] **(GF013_15)** 元
16. 防疫支出，包括购买口罩，防护服和消毒液 ______ [hc$([0,\infty), int, -1)$，sc$([0,100000], int, -1)$] **(GF013_16)** 元

【**访员注意**：没有相应项支出用“0”元表示，记不清则请受访者估计一个数字。如果受访者拒绝回答或者忘记了，填入“-1”。】

GF014 自疫情发生以来，您觉得 [XMainR] 家的收入是否能够负担日常的花费？
1. 非常困难
2. 有点困难
3. 容易
4. 很容易

GF015 自疫情发生以来，[XMainR] 家是否停止付房租，房贷以及水电煤气等费用？（可多选）
1. 停止付房租
2. 停止还房贷
3. 停止付水电煤气费
4. 以上都没有

[conflict$(4,[4]^c)$]

GF016 既然收入很难维持日常生活，[XMainR] 家是如何度过难关的呢？以下的金额都是自疫情发生以来到现在一共的数额（可多选）。

【访员注意：如果受访者拒绝回答或者忘记了，填入“-1”。】

1. 减少支出，一共减少了 ______ [hc((0, ∞), *int*, −1)，sc((0, 50000], *int*, −1)] (**GF016_1**) 元
2. 使用储蓄，取出了 ______ [hc((0, ∞), *int*, −1)，sc((0, 50000], *int*, −1)] (**GF016_2**) 元
3. 出售资产，如 ______ (**GF016_9**)，得到了 ______ [hc((0, ∞), *int*, −1)，sc((0, 50000], *int*, −1)] (**GF016_3**) 元
4. 从亲戚朋友那得到帮助，即不需要还的钱，得到了 ______ [hc((0, ∞), *int*, −1)，sc((0, 50000], *int*, −1)] (**GF016_4**) 元
5. 向亲戚朋友借钱，借到了 ______ [hc((0, ∞), *int*, −1)，sc((0, 50000], *int*, −1)] (**GF016_5**) 元
6. 向银行借钱，借到了 ______ [hc((0, ∞), *int*, −1)，sc((0, 50000], *int*, −1)] (**GF016_6**) 元
7. 其他，如 ______ (**GF016_8**)，得到了 ______ [hc((0, ∞), *int*, −1)，sc((0, 50000], *int*, −1)] (**GF016_7**) 元
8. 什么都不做

[conflict(8, $[8]^c$)]

GF017 受访者填写该部分问卷时是否求助？

1. 从未
2. 偶尔几次
3. 大多数时间

I. 住房情况

I001 [XFinancialResp] 正在居住的房子是什么建筑结构？

【访员注意：如访员在受访者家中，访员自己记录。否则，询问受访者相关情况。】

1. 钢筋混凝土或砖木结构
2. 土坯房/土房
3. 木草屋/茅草屋
4. 窑洞
5. 蒙古包/毡房/帐篷
6. 石头房
7. 其它结构，请注明 ______ (**I001_1**)

I002 [XFinancialResp] 正在居住的房子是什么时候入住的？ ______ [hc([1900, 2020], *int*, −1)] 年

【访员注意：如果受访者拒绝回答或者忘记了，填入“-1”。】

I003 如果不清楚具体年份，从以下选项中选择：

1. 0-5 年
2. 5-10 年
3. 10-20 年
4. 20-30 年
5. 30-40 年
6. 40 年以上

I004 [XFinancialResp] 正在居住的房子所在建筑是平房还是楼房？

【访员注意：如访员在受访者家中，访员自己记录。否则，询问受访者相关情况。】

1. 平房

2. 一般楼房
3. 独门独户楼房

I005 是独立的平房，还是大杂院？

【**访员注意**：如访员在受访者家中，访员自己记录。否则，询问受访者相关情况。】

1. 独立的平房
2. 大杂院

I006 该住房在第几层？

【**访员注意**：如果住在地下二层，请在选项 2，填 2 层。如访员在受访者家中，访员自己记录。否则，询问受访者相关情况。】

1. 地上 ______ [hc($[1,\infty), int, \varnothing$)，sc($[1,20], int, \varnothing$)] (**I006_1**) 层
2. 地下 ______ [hc($[1,\infty), int, \varnothing$)，sc($[1,5], int, \varnothing$)] (**I006_2**) 层

I007 有电梯吗？

【**访员注意**：如访员在受访者家中且清楚答案，访员自己记录。否则，询问受访者相关情况。】

1. 是
2. 否

I008 请问楼房的电梯是楼房建好就有还是后来加上的？

【**访员注意**：如果受访者拒绝回答或者忘记了，填入"-1"。】

1. 刚建好就有
2. 后来加的，加的时间是 ______ [hc($[1900,2020], int, -1$)] (**I008_1**) 年
3. 不知道

I009 住宅是否有无障碍通道？（例如：没有台阶的斜坡等）

【**访员注意**：如访员在受访者家中且清楚答案，访员自己记录。否则，询问受访者相关情况。】

1. 是
2. 否
3. 平地无台阶，不需要无障碍通道

I010 从外面回来要爬多少个阶梯才能到家门口？如果有电梯是指坐电梯情形下要走的阶梯 ______ [hc($[0,\infty), int, \varnothing$)，sc($(0,30], int, \varnothing$)] 个

【**访员注意**：如访员在受访者家中且清楚答案，访员自己记录。否则，询问受访者相关情况。】

I011 正在居住的房子有 ______ [hc($[0,\infty), int, \varnothing$)，sc($(0,20], int, \varnothing$)] (**I011_1**) 室 ______ [hc($[0,\infty), int, \varnothing$)，sc($(0,20], int, \varnothing$)] (**I011_2**) 厅 ______ [hc($[0,\infty), int, \varnothing$)，sc($(0,20], int, \varnothing$)] (**I011_3**) 卫生间（厕所） ______ [hc($[0,\infty), int, \varnothing$)，sc($(0,20], int, \varnothing$)] (**I011_4**) 厨房 ______ [hc($[0,\infty), int, \varnothing$)，sc($(0,20], int, \varnothing$)] (**I011_5**) 阳台。

【**访员注意**：如访员在受访者家中且清楚答案，访员自己记录。否则，询问受访者相关情况。】

I012 离 [XFinancialResp] 正在居住的房子最近的厕所多远？ ______ [hc($[0,\infty), int, \varnothing$)，sc($(0,100], int, \varnothing$)] 米

I013 厕所是什么样的？是蹲坑式还是坐式？如果两者都有，选坐式。

【**访员注意**：如访员在受访者家中且清楚答案，访员自己记录。否则，询问受访者相关情况。】

1. 蹲坑式
2. 坐式

I014 厕所能冲水吗?

【**访员注意**：如访员在受访者家中且清楚答案，访员自己记录。否则，询问受访者相关情况。】

1. 是
2. 否

I015 [XFinancialResp] 正在居住的房子是否有电?

【**访员注意**：如访员在受访者家中且清楚答案，访员自己记录。否则，询问受访者相关情况。】

1. 是
2. 否

I016 是否有自来水？即开水龙头就有水。

【**访员注意**：如访员在受访者家中且清楚答案，访员自己记录。否则，询问受访者相关情况。】

1. 是
2. 否

I017 住房内有无洗澡设施？是什么样的?

1. 统一供热水
2. 家庭自装热水器
3. 无

I018 是否有管道煤气或天然气?

1. 是
2. 否

I019 是否有集中统一供暖？(不包括土暖气和可制暖的空调)

1. 是
2. 否

I020 如果给住的地方供暖，所用的主要能源是什么?

1. 太阳能
2. 煤炭、蜂窝煤
3. 管道天然气或煤气
4. 液化石油气
5. 电
6. 秸秆、柴火
7. 其他，请注明 ______ **(I020_1)**
8. 不供暖

I021 如果做饭的话，做饭用的主要燃料是什么?

1. 煤炭、蜂窝煤
2. 管道天然气或煤气
3. 沼气
4. 液化石油气
5. 电
6. 秸秆、柴火
7. 太阳能
8. 其他，请注明 ______ **(I021_1)**
9. 不做饭

I022 [XFinancialResp] 住的地方装电话了吗?

【**访员注意**：如访员在受访者家中且清楚答案，访员自己记录。否则，询问受访者相关情况。】

1. 是
2. 否

I023 [XFinancialResp] 正在居住的地方可以宽带上网吗?

【**访员注意**：如访员在受访者家中且清楚答案，访员自己记录。否则，询问受访者相关情况。】

1. 是
2. 否

I024 [XFinancialResp] 正在居住的地方是否有空气净化器?

【**访员注意**：如访员在受访者家中且清楚答案，访员自己记录。否则，询问受访者相关情况。】

1. 是
2. 否

I025 这户人家的室内整洁度如何?

【**访员注意**：访员自己记录。】

1. 非常整洁
2. 很整洁
3. 整洁
4. 一般
5. 不整洁
6. 不适用

I026 这户人家的室内温度如何?

【**访员注意**：访员自己记录。】

1. 很热
2. 比较热
3. 还可以
4. 比较冷
5. 很冷
6. 不适用

I027 这户人家的地板情况如何?

【**访员注意**：如访员在受访者家中且清楚答案，访员自己记录。否则，询问受访者相关情况。】

1. 平整的有覆盖物的地面，如地毯、木地板、大理石、瓷砖、或地板革等材料
2. 平整的水泥地面
3. 不平的地面

I028 [XFinancialResp] 现在住房的建筑面积是多大? ______ [hc$((0,\infty), real, \varnothing)$, sc$((10,500], real, \varnothing)$] 平方米

I029 自来水是否经过自来水厂处理?

1. 是
2. 否

997. 不知道

G2 个人收入

G2. 代理模式确认

proxy_8 访员记录，对于个人收入模块是否使用代理问卷模式？

1. 是
2. 否

GA. 个人收入

【引语：向主要受访者及其配偶分别提问此部分问卷。这部分不允许请人完全代填。】

GA001 过去一年，[XRName] 有没有领工资，包括奖金、各种补贴，不包括退休、退职或内退工资？这里的工资来自所有的受聘工作。

1. 有
2. 没有

GA002 过去一年，[XRName] 一共领了多少钱？ ______ [hc $((0,\infty),int,-1)$，sc $((0,240000],int,-1)$，ub $([-1],[5000,10000,30000,50000,100000])$] 元

【访员注意：如果受访者拒绝回答或者忘记了，填入"-1"。】

GA003 上面提到的工资有没有扣除各类保险、所得税、住房公积金或其他杂费？

1. 有
2. 没有

997. 不知道
999. 拒绝回答

GA004 [XRName] [XGA004Text] 被扣除的或上交的个人所得税、各类保险、住房公积金或其他杂费一共是多少元？

【访员注意：如果受访者拒绝回答或者忘记了，选第一个选项，填入"-1"。】

1. ______ [hc $((0,\infty),int,-1)$，sc $((0,10000],int,-1)$，ub $([-1],[300,500,1000,2000,3000])$] (**GA004_1**) 元/月
2. ______ [hc $((0,\infty),int,\varnothing)$，sc $((0,100000],int,\varnothing)$] (**GA004_2**) 元/年
3. 或相当于工资的 ______ [hc $((0,100],real,\varnothing)$] (**GA004_3**) %
4. 没有，0 元

GA005 过去一年，[XRName] 有没有领到下列转移支付收入？注意的是，疫情发生后的，个人得到的疫情补贴也属于转移支付收入，故须在此填写（可多选）。

【访员注意：如果受访者拒绝回答或者忘记了，填入"-1"。】

1. 退休金或养老金，包括政府机关和事业单位退休金，企业职工基本养老保险，企业补充养老保险，退职或内退补偿金，农村或城乡或城镇居民养老保险，商业养老保险，人寿保险，征地养老保险等，领了 ______ [hc $((0,\infty),int,-1)$，sc $((0,100000],int,-1)$，ub $([-1],[1000,3000,6000,10000,25000])$] (**GA005_1**) 元
2. 失业补助，领了 ______ [hc $((0,\infty),int,-1)$，sc $((0,50000],int,-1)$] (**GA005_2**) 元
3. 养老卡或券，领了 ______ [hc $((0,\infty),int,-1)$，sc $((0,50000],int,-1)$] (**GA005_3**) 元
4. 高龄老人养老补助，领了 ______ [hc $((0,\infty),int,-1)$，sc $((0,50000],int,-1)$] (**GA005_4**) 元

5. 工伤保险金包括误工补贴、伤残补助等，领了 ______ [hc((0, ∞), int, −1)， sc((0, 50000], int, −1)] (GA005_5) 元
6. 独生子女老年补助，领了 ______ [hc((0, ∞), int, −1)， sc((0, 50000], int, −1)] (GA005_6) 元
7. 医疗救助，领了 ______ [hc((0, ∞), int, −1)， sc((0, 100000], int, −1)] (GA005_7) 元
8. 政府给个人的其他补助，不包括低保、五保、特困和贫困补助，请注明 ______ (GA005_8_1)，领了 ______ [hc((0, ∞), int, −1)， sc((0, 100000], int, −1)] (GA005_8) 元
9. 社会给个人的其他转移支付收入，如社会捐助等，请注明 ______ (GA005_9_1)，领了 ______ [hc((0, ∞), int, −1)， sc((0, 100000], int, −1)] (GA005_9) 元
10. 以上均没有

[conflict(10, $[10]^c$)]

GA006 考虑一下，如果没有疫情，[XRName] 可能会有一些工资收入，但是因为疫情的原因，情况可能发生变化。那么，从春节到现在，疫情使 [XRName] 工资收入增加了，还是减少了，或者没有影响？

【访员注意：如果受访者拒绝回答或者忘记了，填入“-1”。】

1. 减少，减少了 ______ [hc((0, ∞), int, −1)， sc((0, 50000], int, −1)， ub([−1], [1000, 3000, 5000, 10000, 20000])] (GA006_1) 元
2. 增加，增加了 ______ [hc((0, ∞), int, −1)， sc((0, 50000], int, −1)， ub([−1], [1000, 3000, 5000, 10000, 20000])] (GA006_2) 元
3. 没有变化

GA007 [XRName] 是否立过遗嘱？

1. 是
2. 否

GA008 [XRName] 是否考虑过自己去世之后的财产安排？

1. 考虑过
2. 没有考虑过
3. 没有财产

GA009 [XGA009Text] 如果 [XRName] 去世了，会对财产如何分配？

【访员注意：如果受访者拒绝回答或者忘记了，填入“-1”。】

1. 配偶 ______ [hc((0, 100], real, −1)] (GA009_1) %
2. 子女、女婿、儿媳
3. 兄弟姐妹 ______ [hc((0, 100], real, −1)] (GA009_3) %
4. 其他亲戚 ______ [hc((0, 100], real, −1)] (GA009_4) %
5. 父母、岳父母 ______ [hc((0, 100], real, −1)] (GA009_5) %
6. 孙子女、外孙子女
7. 朋友 ______ [hc((0, 100], real, −1)] (GA009_7) %
8. 慈善机构 ______ [hc((0, 100], real, −1)] (GA009_8) %
9. 其他，请注明 ______ (GA009_9_1)， ______ [hc((0, 100], real, −1)] (GA009_9) %
10. 不分配

997. [XProxyText]

[conflict(10, 997, $[10, 997]^c$)]

GA010 财产分配给了哪些子女/子女的配偶？子女的配偶算作子女部分。如果财产分配到的子女不在选项里，请添加 [XRName] 的子女名字（可多选）。

1-25. [XChildAliveName[i]]
26-35. 其他子女，名字为 ______ (**GA010_1[i]**)

GA011[i] [XGAChildList1[i]] 及其配偶获得财产的百分比是 ______ [hc((0, 100], *real*, −1)] %。
【**访员注意**：如果受访者拒绝回答或者忘记了，填入"-1"。】

GA014 孙子女/外孙子女是哪些子女的孩子？如果财产分配到的孙子女的父亲/外孙子女的母亲不在子女选项里，请添加 [XRName] 的子女名字（可多选）。
1-25. [XChildAliveName[i]]
26-35. 其他子女，名字为 ______ (**GA014_1[i]**)

GA015[i] [XGAChildList2[i]] 的孩子获得财产的百分比是 ______ [hc((0, 100], *real*, −1)] %。
【**访员注意**：如果受访者拒绝回答或者忘记了，填入"-1"。】

GA016 [XRName] 觉得财产（遗产）实际分配的时间是什么时候？自己去世之前、之后？
1. [XRName] 去世之后分配
2. [XRName] 在世的时候分配
997. [XProxyText]
[conflict(997, $[997]^c$)]

GA017 [XRName] 的财产分配是否按照继承法分配？
1. 按继承法规定，有继承资格的人平均分配
2. 自己安排

GA018 [XRName] 觉得财产（遗产）实际分配的时间是什么时候？自己和配偶去世之前、之后或者其中一个去世之后？
1. [XRName] 及其配偶都去世之后分配
2. [XRName] 及其配偶都在世的时候分配
3. [XRName] 或其配偶有一个去世就分配
997. [XProxyText]
[conflict(997, $[997]^c$)]

辅助变量定义

XFLHHFinancialNameList 将主要受访者和其配偶名字，用"或"连在一起

```
if (!empty("XMainRS")) {
        add("XFLHHFinancialNameList", value("XMainR")+"或"+value("XMainRS"))
} else {
        add("XFLHHFinancialNameList", value("XMainR"))
}
```

XFinancialResp 家庭财务受访者

```
for (var i1 = 1; i1 < 26; i1++) {
  if (equal("GB001", i1)) {
^^I^^Iadd("XFinancialResp", value("XHHMemberName[i1]"))
^^I}
}
if (equal("GB001", 26)) {
^^Iadd("XFinancialResp", value("XMainR"))
}
if (equal("GB001", 27)) {
^^Iadd("XFinancialResp", value("XMainRS"))
}
```

XGB005Text GB005 题干用词

```
add("XGB005Text[i]", "")
if (equal("GB004[i]", "2") || equal("GB004[i]", "997")) {
^^Iadd("XGB005Text[i]", "应该")
}
```

XFLHHNameList 家户成员名字用逗号连在一起，题干用词

```
if (!empty("XMainRS")) {
        add("XFLHHNameList", value("XMainR")+", "+value("XMainRS"))
} else {
        add("XFLHHNameList", value("XMainR"))
}

for (var i1 = 1; greater("XHHMemberNum", i1, true) ; i1++){
        add("XFLHHNameList", value("XFLHHNameList")+", "+value("XHHMemberName[i1]"))
}
```

XHHOtherMemberName2 非子女的家户成员名字

```
for (var k = 1; k <= 10; k++) {
    add("XHHOtherMemberName2["+(k+25)+"]", value("XHHOtherMemberName[k]"))
}
```

XPoorHHName 政府给予的贫困补助名字列表

```
add("XPoorHHName[1]", "五保户")
add("XPoorHHName[2]", "低保户")
add("XPoorHHName[3]", "建档立卡贫困户")
add("XPoorHHName[4]", "您说的其他类型贫困户，即"+value("GE001_1"))
```

XPoorLstYr 过去一年，是否有政府给予的贫困补助列表

```
add("XPoorLstYr[i]", "0")
if (equal("GE002[i]", "1")) {
    add("XPoorLstYr[i]", "1")
}
if (equal("GE002[i]", "2") &&  equal("GE015[i]", "2020")) {
    add("XPoorLstYr[i]", "1")
}
```

XFLHHNameListPoor 过去一年，领取各项贫困补助的家户成员列表

```
add("XFLHHNameListPoor[i]", "")
for (var i1 = 1; i1 < 26; i1++) {
  ^^Iif (selected("GE003[i]", i1)) {
^^I^^Iadd("XFLHHNameListPoor[i]", value("XHHMemberName[i1]")+", "+value("XFLHHNameListPoor[i]"))
^^I}
}

if (selected("GE003[i]", 26)) {
^^I^^Iadd("XFLHHNameListPoor[i]", value("XMainR")+", "+value("XFLHHNameListPoor[i]"))^^I
}

if (selected("GE003[i]", 27)) {
^^I^^Iadd("XFLHHNameListPoor[i]", value("XMainRS")+", "+value("XFLHHNameListPoor[i]"))^^I
}
```

XProxyText 在代理模式下，某些题目增加”不知道“选项

```
add("XProxyText", "")
if (equal("proxy_8", "1")) {
^^Iadd("XProxyText", "不知道")
}
```

XGA004Text GA004 题干用词

```
add("XGA004Text", "")
if (equal("GA003", "2") || equal("GA003", "997")) {
^^Iadd("XGA004Text", "应该")
}
```

XGA009Text GA009 题干用词

```
add("XGA009Text", "")
if (equal("GA007", "1")) {
^^Iadd("XGA009Text", "在遗嘱中，")
}
```

XGAChildList1 GA 部分子女列表 1

```
for (var i1 = 1; i1 <=25 ; i1++) {
    add("XGAChildList1[i1]", value("XChildAliveName[i1]"))
}
for (var i1 = 26; i1 <=35 ; i1++) {
    add("XGAChildList1[i1]", value("GA010_1[i1]"))
}
```

XGAChildList2 GA 部分子女列表 2

```
for (var i1 = 1; i1 <=25 ; i1++) {
    add("XGAChildList2[i1]", value("XChildAliveName[i1]"))
}
for (var i1 = 26; i1 <=35 ; i1++) {
    add("XGAChildList2[i1]", value("GA014_1[i1]"))
}
```

This page intentionally left blank

V　疫情

V. 代理模式确认

proxy_14 访员记录，对于疫情模块是否使用代理问卷模式？

1. 是
2. 否

VA. 疾病防范意识

【引语：下面我想了解一下疫情对 [XRName] 的影响。】

VA001 [XRName] 是否知道下列的这些做法可以降低感染新冠病毒的风险？（可多选）

【访员注意：“疫情模块”仅涉及有关于疫情防控的受访者信息。疫情对受访者医疗、就业和收入的影响，其相关问题请参见其他模块。】

1. 洗手
2. 使用酒精和消毒剂
3. 避免与他人握手
4. 戴口罩、戴手套
5. 避免旅行
6. 避免去人多的群体性集会
7. 和别人面对面说话的时候保持距离
8. 其他，请简略说明 ______ (**VA001_1**)
10. [XRName] 知道疫情的存在，但不知道任何的预防措施（排他选项）
11. [XRName] 之前没听说过这个疾病，不知道疫情的存在（排他选项）

[conflict(10, 11, $[10, 11]^c$)]

VA002 [XRName] 是从哪里知道怎么做可以降低感染风险的？（可多选，选项免读）

1. 电视新闻
2. 报纸新闻
3. 网络媒体如手机新闻和微信等
4. 电台广播
5. 亲朋好友、同事领导、社区人员
6. 医务人员
7. 大喇叭广播
8. 宣传海报
9. 其他
10. 没有外部渠道，[XRName] 全凭自己的理解和生活经验（排他选项）

[conflict(10, $[10]^c$)]

VA003 疫情期间，[XRName] 出门戴口罩吗？（读前三个选项）

1. 每次出门都戴
2. 有时候出门不戴
3. 从来不戴
4. 疫情期间没出过门

VA004 [XRName] 现在会出门吗？出门会戴口罩吗？（选项免读）

1. 现在出门会戴

2. 现在出门也不戴
3. 一直不出门

VA005 [XRName] 现在出门会戴口罩吗？如果之前戴，现在不戴，是从什么时候开始不戴口罩的？（选项免读）

【**访员注意**：现在有时戴有时候不戴，选第一个选项。如果受访者无法回答月份，请填“-1”。】

1. 现在出门还戴
2. 疫情期间戴过，现在出门不戴：不戴口罩已经有 ______ [hc([0, 14], *int*, −1)，sc([0, 6], *int*, −1)] (**VA005_1**) 个月
3. 疫情期间和现在都不戴
4. 疫情之后没出过门

VA006 在武汉因为疫情封城之后的三天里，就是大年三十到正月初二（1 月 24 日到 1 月 26 日），对于下面的东西 [XRName] 有没有因为疫情而买得比平时多，先囤着？（可多选）

1. 粮油蔬菜
2. 口罩、洗手液或消毒液
3. 以上都没有

[conflict(3, $[3]^c$)]

VA007 疫情期间，咱们政府为控制新冠肺炎采取了一些措施，[XRName] 觉得是太严格了、合适还是不够严格？（选项免读）

1. 太严格了
2. 比较合适
3. 不够严格

997. 不知道
999. 拒绝回答

VB. 个人患病和隔离

VB001 [XRName] 自己，身边，和熟人中是否有人曾被认定为新冠肺炎确诊病例或疑似病例？如果有，和 [XRName] 的关系是？（可多选，选项免读）

1. 自己
2. 一起住的人：______ (**VB001_1**)
3. 其他亲属（不一起住）：______ (**VB001_2**)
4. 熟人朋友（如好友，邻居，同事等）：______ (**VB001_3**)
5. 没有

999. 拒绝回答

[conflict(5, 999, $[5, 999]^c$)]

VB002 他们康复得都好吧，有人去世了吗？如果有，和 [XRName] 的关系是？（可多选，选项免读）

1. 一起住的人：______ (**VB002_1**)
2. 其他亲属（不一起住）：______ (**VB002_2**)
3. 熟人朋友（如好友，邻居，同事等）：______ (**VB002_3**)
4. 没有

999. 拒绝回答

[conflict(4, 999, $[4, 999]^c$)]

VB004 为治疗新冠肺炎，[XRName] 住院了吗？在医院一共住了 ______ [hc([0, 250], *int*, −1)] 天？（没住院填“0”）

【**访员注意**：如果受访者无法回答，请填“-1”。】

VB005 [XVAHospitalizationExcluded] [XRName] 是否由于以下原因曾被隔离或医学观察？居家隔离和封楼都算被隔离。（可多选）

1. 旅行出差（包括春节后去上班）
2. 是新冠病例密切接触者
3. 住宅或住宅楼被封闭（不含小区封闭）
4. 去医疗机构诊疗时或出院后被要求隔离
5. 核酸检测显示阳性
6. 没有隔离经历（排他选项）

997. 不知道
999. 拒绝回答

[conflict(6, 997, 999, [6, 997, 999]^c)]

VB008 [XRName] 总共被隔离了 ______ [hc([1, 250], *int*, −1)，sc([1, 14], *int*, −1)] 天？

【**访员注意**：如果有多次隔离经历，请告知加总后的时长。如果受访者无法回答，请填“-1”。】

VB009 [XRName] 被隔离的场所是在？（可多选）

1. 医院
2. 酒店等集中的医学观察点
3. 自己住处
4. 其他：______ (**VB009_1**)

997. 不知道
999. 拒绝回答

[conflict(997, 999, [997, 999]^c)]

VB010 [XRName] 为隔离支付了多少费用？包括食宿费，总计 ______ [hc([0, 100000], *int*, −1)] 元。

【**访员注意**：如果受访者无法回答，请填“-1”。】

VB011 在 [XRName] 的隔离经历中，是否曾有他人在隔离住所内与 [XRName] 共同隔离，互相可以见面交谈？

1. 是
2. 否

997. 不知道
999. 拒绝回答

VB012 [XRName] 是否接受过新冠病毒检测？

【**访员注意**：如果受访者无法回答月份，请填“-1”。】

1. 是，最近一次检测时间是在 ______ [hc([2020,XIWYear], *int*, −1)] (**VB012_1**) 年 ______ [hc([1, 12], *int*, −1)，sc([1,XIWMonth], *int*, −1)] (**VB012_2**) 月
2. 否

997. 不知道
999. 拒绝回答

VC. 疫情期间个人活动

VC000 疫情期间 [XVCNotInQuarantine]，[XRName] 是否曾经因为担心会被传染而好几天都没出家门？这种情况最长的一次有几天？ ______ [hc([0, 250], *int*, −1)] 天（无此情况或每天都出家门，填“0”）

【**访员注意**：如果受访者无法回答，请填“-1”。】

【**引语**：下面我想了解一下 [XRName] 在疫情最严重时间的一些活动，也就是今年春节后 [XVCOutbreak] 这段时间，从 1 月 25 日到 2 月 22 日左右。】

VC001 [XVCOutbreak]，[XRName] 一般都住在哪儿？

【**访员注意**：如受访者不知道街道和社区，可酌情选择】

1. 现居住地的同个村/社区：[XRResidenceFull]
2. 现居住地同区县 [XRResidenceCounty] 的其他村/社区：______ (**VC001_1**) 乡/镇/街道/村/社区
3. 现居住地区县以外：______ (**VC001_2**) 省/市/区县 ______ (**VC001_3**) 乡/镇/街道/村/社区
4. 以上选项均不适用的地区（港澳台及国外）

999. 拒绝回答

VC002 [XVCOutbreak] [XVCNotInQuarantine]，[XRName] 每天的出门次数和如果疫情没发生这样的情况相比，是增加了，减少了，还是没变化？

【**访员注意**：疫情没发生这样的情况，不能解释为“去年正月里”或“现在正常的时候”。

特例：当受访者对于疫情一无所知，使得访员无法描述疫情没发生的情况，访员可将“和如果疫情没发生这样的情况相比”解释为“与去年正月里相比，不考虑身体状况变化的因素”。下同。】

1. 大幅增加
2. 小幅增加
3. 没有变化
4. 小幅减少
5. 大幅减少

VC003 [XVCOutbreak] [XVCNotInQuarantine]，[XRName] 每天出门在外的时间和如果疫情没发生这样的情况相比，是增加了，减少了，还是没变化？

【**访员注意**：疫情没发生这样的情况，不能解释为“去年正月里”或“现在正常的时候”。】

1. 大幅增加
2. 小幅增加
3. 没有变化
4. 小幅减少
5. 大幅减少

VC004 [XVCOutbreak] [XVCNotInQuarantine]，[XRName] 每天进行激烈活动的时间和如果疫情没发生这样的情况相比，是增加了，减少了，还是没变化？激烈活动非常消耗体力，会让人呼吸急促，包括搬运重物、挖地、耕作、有氧运动、快速骑车、骑车载货等，也包括室内的激烈活动。

【**访员注意**：疫情没发生这样的情况，不能解释为“去年正月里”或“现在正常的时候”。】

1. 大幅增加
2. 小幅增加

3. 没有变化
4. 小幅减少
5. 大幅减少

VC005 [XVCOutbreak] [XVCNotInQuarantine]，[XRName] 每天进行中等强度体力活动的时间和如果疫情没发生这样的情况相比，是增加了，减少了，还是没变化？中等强度的体力活动包括搬运轻便的东西，拖地，常规速度骑车，快走等。

【**访员注意**：疫情没发生这样的情况，不能解释为“去年正月里”或“现在正常的时候”。】

1. 大幅增加
2. 小幅增加
3. 没有变化
4. 小幅减少
5. 大幅减少

VC006 [XVCOutbreak] [XVCNotInQuarantine]，[XRName] 每天进行轻度体力活动，如走路，散步等活动的时间和如果疫情没发生这样的情况相比，是增加了，减少了，还是没变化？

【**访员注意**：疫情没发生这样的情况，不能解释为“去年正月里”或“现在正常的时候”。】

1. 大幅增加
2. 小幅增加
3. 没有变化
4. 小幅减少
5. 大幅减少

VC007 [XVCOutbreak] [XVCNotInQuarantine]，[XRName] 串门的频率和如果疫情没发生这样的情况相比，是增加了，减少了，还是没变化？

【**访员注意**：疫情没发生这样的情况，不能解释为“去年正月里”或“现在正常的时候”。】

1. 大幅增加
2. 小幅增加
3. 没有变化
4. 小幅减少
5. 大幅减少
6. 从不串门

VC008 [XVCOutbreak] [XVCNotInQuarantine]，[XRName] 打麻将、下棋和打牌的频率和如果疫情没发生这样的情况相比，是增加了，减少了，还是没变化？

【**访员注意**：疫情没发生这样的情况，不能解释为“去年正月里”或“现在正常的时候”。】

1. 大幅增加
2. 小幅增加
3. 没有变化
4. 小幅减少
5. 大幅减少
6. 从不参与棋牌活动

VC009 [XVCOutbreak] [XVCNotInQuarantine]，[XRName] 跳广场舞的频率和如果疫情没发生这样的情况相比，是增加了，减少了，还是没变化？

【**访员注意**：疫情没发生这样的情况，不能解释为“去年正月里”或“现在正常的时候”。】

1. 大幅增加

2. 小幅增加
3. 没有变化
4. 小幅减少
5. 大幅减少
6. 从不跳广场舞

VC010 [XVCOutbreak]，[XRName] 打电话和发短信的频率，和如果疫情没发生这样的情况相比，是增加了，减少了，还是没变化?

【**访员注意**：疫情没发生这样的情况，不能解释为“去年正月里”或“现在正常的时候”。】

1. 大幅增加
2. 小幅增加
3. 没有变化
4. 小幅减少
5. 大幅减少
6. 住处不具备条件
7. 从不如此

VC011 [XVCOutbreak]，[XRName] 使用网络和亲友联系，比如发微信、语音和视频聊天等的频率，和如果疫情没发生这样的情况相比，是增加了，减少了，还是没变化?

【**访员注意**：疫情没发生这样的情况，不能解释为“去年正月里”或“现在正常的时候”。】

1. 大幅增加
2. 小幅增加
3. 没有变化
4. 小幅减少
5. 大幅减少
6. 住处不具备条件
7. 从不如此

VC012 [XVCOutbreak]，[XRName] 是否曾因为疫情或和疫情有关的事情感到害怕?

【**访员注意**：疫情没发生这样的情况，不能解释为“去年正月里”或“现在正常的时候”。】

1. 很少或者根本没有
2. 不太多
3. 有时或者说有一半的时间
4. 大多数的时间

997. 不知道
999. 拒绝回答

VC013 [XVCOutbreak]，[XRName] 是否曾因为疫情或和疫情有关的事情感到紧张或焦虑?

【**访员注意**：疫情没发生这样的情况，不能解释为“去年正月里”或“现在正常的时候”。】

1. 很少或者根本没有
2. 不太多
3. 有时或者说有一半的时间
4. 大多数的时间

997. 不知道
999. 拒绝回答

VC014 [XVCOutbreak]，[XRName] 的吸烟量，和如果疫情没发生这样的情况相比，是增加了，减少

了，还是没变化?

【访员注意：“从未吸烟”包括近年来从未吸烟。疫情没发生这样的情况，不能解释为“去年正月里”或“现在正常的时候”。】

1. 大幅增加
2. 小幅增加
3. 没有变化
4. 小幅减少
5. 大幅减少
6. 从不吸烟

VC015 [XVCOutbreak]，[XRName] 的饮酒量，和如果疫情没发生这样的情况相比，是增加了，减少了，还是没变化?

【访员注意：“从未饮酒”包括近年来从未饮酒。疫情没发生这样的情况，不能解释为“去年正月里”或“现在正常的时候”。】

1. 大幅增加
2. 小幅增加
3. 没有变化
4. 小幅减少
5. 大幅减少
6. 从不饮酒

VC016 [XVCOutbreak]，[XRName] 每天晚上的睡眠时间和如果疫情没发生这样的情况相比，是增加了，减少了，还是没变化? 睡眠时间指的是真正睡着的时间，有可能短于在床上躺着的时间。

【访员注意：疫情没发生这样的情况，不能解释为“去年正月里”或“现在正常的时候”。】

1. 大幅增加
2. 小幅增加
3. 没有变化
4. 小幅减少
5. 大幅减少

VC017 [XVCOutbreak]，[XRName] 每天的进食量和如果疫情没发生这样的情况相比，是增加了，减少了，还是没变化?

【访员注意：疫情没发生这样的情况，不能解释为“去年正月里”或“现在正常的时候”。】

1. 大幅增加
2. 小幅增加
3. 没有变化
4. 小幅减少
5. 大幅减少

VD. 疫期居住地管控

VD001 想通过您了解一下，由于疫情管控的原因，[XRName] 春节以来居住过的小区或村是否实行了以下各类对内外部人员出入的限制? [XRName] 所经历过的限制各有多少天? 这四类限制的询问顺序按由紧到松排列，限制时期不能重合，时间长度加起来不应该超过 [XIWMonth] 个月。(可多选，逐项提示)

【访员注意：只考虑受访者经历过的限制。因搬家或疫情反复，被限制的经历可能会不连续，需要将不连续的同一阶段加总在一起。
出入证和刷脸这些鉴别区内人员的手段，是对访客的限制，并不是对区内人员出入的限制。
如某项限制经历尚未结束，时长截止到访问日。访员可以询问大概的起始和结束日期以协助受访者计算。
如果受访者无法回答经历天数，填“-1”。】

1. 内部人员封闭式管理，外来人员禁入 ：如无特殊情况，区内人员完全不可进出，总计经历 ______ [hc$([1,120], int, -1)$] (**VD001_1**) 天
2. 内部人员半封闭式管理，外来人员禁入 ：区内人员可以进出，但每日出入次数受到限制，如每户限每天次数允许外出采购生活必需品等，总计经历 ______ [hc$([1,200], int, -1)$] (**VD001_2**) 天
3. 内部人员进出不受限制，外来人员禁入： 访客不能进入小区/村居，总计经历 ______ [hc$([1,300], int, -1)$] (**VD001_3**) 天
4. 内部人员进出不受限制，外来人员有条件进入 ：访客持有健康码能进入小区/村居，总计经历 ______ [hc$([1,400], int, -1)$，sc$([1,300], int, -1)$] (**VD001_4**) 天
5. 以上各类管控都未曾经历，内外部人员的出入自由与疫情前相同（排他选项）

997. 完全不知道上述四种管控措施是否曾经实行过
999. 拒绝回答

[conflict$(5, 997, 999, [5, 997, 999]^c)$]

VD002 想通过您了解一下，自春节以来，[XRName] 住过的社区或村的广场舞活动曾经被取消过吗？把所有 [XRName] 经历过的广场舞取消都算上，到今天为止，总共有 ______ [hc$([0,250], int, -1)$] 天？（如未经历过，填“0”）

【访员注意：如果受访者无法回答，请填“-1”。】

VD003 想通过您了解一下，自春节以来，[XRName] 住过的社区或村的麻将馆或社区活动室等棋牌娱乐场馆曾被取消过吗？把所有 [XRName] 经历过的公共棋牌活动取消都算上，到今天为止，总共有 ______ [hc$([0,250], int, -1)$] 天？（如未经历过，填“0”）

【访员注意：如果受访者无法回答，请填“-1”。】

辅助变量定义

XQuarantined 是否曾隔离

```
if (selected("VB005", "1") || selected("VB005", "2") || selected("VB005", "3") || selected("VB005", "4")
↪  || selected("VB005", "5")) {
    add("XQuarantined", "1")
} else {
    add("XQuarantined", "0")
}
```

XVCOutbreak 措辞：疫情活动考察期间

```
add("XVCOutbreak", "正月里")
```

XVCNotInQuarantine 措辞：疫情期间排除隔离

```
if (equal("XQuarantined", "1")) {
    add("XVCNotInQuarantine", "，不包括强制性隔离")
}
```

XVAHospitalizationExcluded 措辞：隔离排除住院时间

```
if (greater("VB004", "0")) {
    add("XVAHospitalizationExcluded", "除去那几天的住院时间，")
}
```

EX　退出问卷

EXB. 基本信息

【引语：抱歉，之前的信息有点不完整，我想再确认一下。】

EXB001 [XRName] 的去世日期是？ ______ [hc([2011, 2020], *int*, ∅)，sc([ZIWYear, 2020], *int*, ∅)] (**EXB001_1**) 年 ______ [hc([1, 12], *int*, ∅)] (**EXB001_2**) 月 ______ [hc([1, 31], *int*, ∅)] (**EXB001_3**) 日

【**访员注意**: 用 4 位数表示年。】

EXB002 [XRName] 去世日期是公历（阳历）还是农历（阴历）?

1. 公历（阳历）
2. 农历（阴历）

EXB003 [XRName] 去世前居住在哪里?

1. 中国大陆 ______ (**EXB003_1**) 省/市/区县 ______ (**EXB003_2**) 乡/镇/街道/村/社区 ______ (**EXB003_3**) 小区/楼号/单元/门牌号
2. 中国香港
3. 中国澳门
4. 中国台湾
5. 国外： ______ (**EXB003_4**)

EXB004 [XRName] 在去世前的居住地类型是什么? 是家庭住宅、工作场所、养老院或其他养老机构、医院病房还是其他?

1. 家庭住宅
2. 工作场所
3. 养老院或其他养老机构
4. 医院病房
5. 其他，请注明 ______ (**EXB004_1**)

EXB005 [XRName] 是在哪里去世的?

1. [XAliveResidenceFull]
2. 中国大陆： ______ (**EXB005_1**) 省/市/区县 ______ (**EXB005_2**) 乡/镇/街道/村/社区 ______ (**EXB005_3**) 小区/楼号/单元/门牌号
3. 中国香港
4. 中国澳门
5. 中国台湾
6. 国外： ______ (**EXB005_4**)

EXB006 在 [XRName] 去世的时候，他/她是在家里、工作场所、医院、养老院、救助机构还是其他地方?

1. 家里
2. 工作场所
3. 医院
4. 去医院的路上
5. 养老院
6. 救助机构
7. 其它地方，请注明 ______ (**EXB006_1**)

EXB007 你们是否想到过 [XRName] 会在那个时候去世?

1. 意料之中的
2. 意料之外的
3. 其它，请注明 _____ (**EXB007_1**)

EXB008 [XRName] 最后生病直到去世是多长时间？一个或两个小时、不到一天、不到一周、不到一个月、不到一年还是几年？
1. 一两个小时或没有任何征兆
2. 不到一天
3. 不到一周
4. 不到一个月
5. 不到一年
6. 一年及以上，是 _____ [hc([1,XRExitAge], *real*, ∅)，sc([1, 20], *real*, ∅)] (**EXB008_1**) 年

EXB009 [XRName] 去世时的婚姻状况？
1. 已婚
2. 分居（不再作为配偶共同生活）
3. 离异
4. 丧偶
5. 从未结婚
6. 同居

EXB010 [XRName] 去世时是否跟配偶/同居伴侣住在一起？
1. 是
2. 否

EXB011 [XRName] 有死亡证明吗？
1. 有
2. 没有

EXB012 [XRName] 死亡证明是在哪里申请的？
【**访员注意**：请家属尽量找到死亡医学证明，证明书上面可能有相关申请地址信息。】
1. 生前居住地 [XAliveResidenceFull] 所在村/社区
2. 生前居住地 [XAliveResidenceFull] 所在县/市/区的其他村/社区 _____ (**EXB012_1**)
3. 其它： _____ (**EXB012_2**) 省/市/县 _____ (**EXB012_3**) 乡/镇/街道/村/社区
4. 国外： _____ (**EXB012_4**)

EXB013 [XRName] 的户口是否已经注销了？
1. 是
2. 否

EXB014 没有注销的原因是？
1. 时间太短，没来得及办理
2. 销户这件事并不重要
3. 其它，请注明 _____ (**EXB014_1**)

VA1A730 能麻烦您提供一下 [XRName] 生前的身份证号码 _____
【**访员注意**：请家属尽量找到死亡医学证明，证明书上面有相关身份证号码信息。如果没有死亡证明，请询问代理人是否能够提供其他文件材料包含 [XRName] 生前的身份证号码信息或者请代理人回忆出身份证号码信

息，这一身份证号信息主要将用于匹配国家死因登记系统个人记录，仅用于科学研究，不会泄露任何个人隐私信息。如果代理人无法回答，填“-1”。】

VA1A750 请将死亡医学证明上面报告的死亡原因记录下来 ______
在得到允许后，请对死亡医学证明拍照记录。______ (**VA1A750_1**)
【**访员注意**：请代理人/家属尽量找到死亡医学证明，并将上面报告的死因记录下来。并向代理人/家属解释数据保密规定，这部分信息仅用于科学研究，不会泄露任何个人隐私信息，在得到允许后，请对死亡医学证明拍照记录。】

EXC. 家庭

【**引语**：下面我想问一些关于 [XRName] 子女的问题】

EXC001[i] 现在 [ZChildName[i]] （性别：[XEChildGenderDis[i]]）还健在吗？
1. 是
2. 否

EXC003[i] [XEChildAliveName[i]] 是什么时候出生的？ ______ [hc($[1910, 2018], int, \varnothing$)，sc($[1940, 2018], int, \varnothing$)] 年
【**访员注意**：用 4 位数表示年，如果受访者记不得出生年份，可以通过子女今年多大，或者哪年去世的，去世时多大以及子女出生时受访者多大等信息推算出生年份。】

EXC004[i] [XEChildAliveName[i]] 的性别是？
1. 男
2. 女

EXC005[i] 不包括成人教育，[XEChildAliveName[i]] 的最高学历是？
1. 未受过正规教育
2. 未读完小学
3. 私塾
4. 小学毕业
5. 初中毕业
6. 高中毕业
7. 中专（包括中等师范、职高）毕业
8. 大专毕业
9. 本科毕业
10. 硕士毕业
11. 博士毕业
997. 不知道
999. 拒绝回答

EXC006[i] 去世前一年，[XEChildAliveName[i]] 与 [XRName] 一起居住了多长时间？ ______ [hc($[0, 12], real, \varnothing$)] 月
【**访员注意**：短暂的走亲戚不算一起居住；没有一起居住请填写 0；一直住在一起请填写 12】

EXC007[i] 去世前一年，[XRName] 和 [XEChildAliveName[i]] 不在一起住的时候，[XRName] 多长时间能见到 [XEChildAliveName[i]] 一次？

1. 差不多每天
2. 每周 2-3 次
3. 每周一次
4. 每半个月一次
5. 每月一次
6. 每三个月一次
7. 半年一次
8. 每年一次
9. 几乎从来没有
10. 其他

EXC008 [XRName] 去世前一共有几个孙子女？（包括亲生子女、继子女和收养子女的孩子，包括外孙子女） ______ [hc$([0, 25], int, \emptyset)$]

EXC009 [XRName] 去世前一共有多少个重孙子女（包括重外孙子女）？ ______ [hc$([0, 25], int, \emptyset)$]

EXD. 健康状况与功能（一）

EXDA001[i] 自 [ZIWTime] 以来，是否有医生诊断 [XRName] 患有 [XEChroDisType[i]]？
1. 是
2. 否

EXDA002[i] 第一次医生诊断出 [XRName] 患有 [XEChroDisType[i]] 是在什么时候？

【**访员注意**：用 4 位数记录年份；

不知道请填写-1】

1. 年份 ______ [hc$([1900, 2020], int, -1)$，sc$([1920, 2020), int, -1)$] (**EXDA002_1[i]**) 年
2. 年龄 ______ [hc$([0, 120], int, -1)$，sc$((0, 100], int, -1)$] (**EXDA002_2[i]**) 岁

EXDA003 自 [ZIWTime] 到 [XRName] 去世，他/她是否发作过心脏病或心肌梗塞？
1. 是
2. 否

EXDA004 [XRName] 最近一次发作心脏病是什么时候？

【**访员注意**：用 4 位数记录年份；

不知道请填写-1】

1. 年份 ______ [hc$([1900, 2020], int, -1)$，sc$([1920, 2020), int, -1)$] (**EXDA004_1**) 年
2. 年龄 ______ [hc$([0, 120], int, -1)$，sc$((0, 100], int, -1)$] (**EXDA004_2**) 岁

EXDA005 [XRName] 身体的哪个器官或部位患有或曾经患有癌症？包括原发和已转移的肿瘤。（可多选）

【**访员注意**：若受访者所患癌症已痊愈，仍需记录】

1. 大脑
2. 口腔
3. 喉
4. 咽
5. 甲状腺
6. 肺

7. 乳房
8. 食管
9. 胃
10. 肝脏
11. 胰腺
12. 肾脏
13. 前列腺
14. 睾丸
15. 卵巢
16. 子宫颈
17. 子宫内膜
18. 结肠或直肠
19. 膀胱
20. 皮肤
21. 非何杰金淋巴瘤（非霍奇金淋巴瘤）
22. 白血病
23. 其他器官 ______ (**EXDA005_1**)

EXDA006 在去世前的两年里，[XRName] 有没有采用以下方式来治疗肿瘤或缓解肿瘤所引起的疼痛、恶心等症状？（可多选）

【**访员注意**：请逐项读出选项，并让受访者逐一回答

(1)“化学疗法”指用化学合成药物治疗疾病的方法。化学药物治疗（简称化疗）是目前治疗肿瘤及某些身免疫性疾病的主要手段之一。

(2)“手术治疗”是最早应用的治疗癌症的方法，也是目前许多早期癌症治疗的首选疗法。

(3)“放射疗法”是用各种不同能量的射线照射肿瘤，以抑制和杀灭癌细胞的一种治疗方法】

1. 服用中药
2. 服用西药
3. 化学疗法
4. 手术治疗
5. 放射疗法
6. 其他治疗方法，请注明 ______ (**EXDA006_1**)
7. 以上都没有

[conflict(7, [7]^c)]

EXDA007 自 [ZIWTime] 以来，是否有医生诊断 [XRName] 中风复发？

1. 是
2. 否

EXDA008 [XRName] 最近一次中风的诊断时间是什么时候？

【**访员注意**：用 4 位数记录年份；

不知道请填写-1】

1. 年份 ______ [hc([1900, 2020], *int*, −1)，sc([1920, 2020), *int*, −1)] (**EXDA008_1**) 年
2. 年龄 ______ [hc([0, 120], *int*, −1)，sc((0, 100], *int*, −1)] (**EXDA008_2**) 岁

EXDA009 [XRName] 去世一个月之前是否存在记忆问题？

1. 是
2. 否

EXDA010 [XRName] 多大年龄的时候记忆出现明显问题？

【**访员注意**：用 4 位数记录年份；
不知道请填写-1】

1. 年份 ______ [hc([1900, 2020], *int*, −1)，sc([1920, 2020), *int*, −1)] (**EXDA010_1**) 年
2. 年龄 ______ [hc([0, 120], *int*, −1)，sc((0, 100], *int*, −1)] (**EXDA010_2**) 岁

EXDA011 记忆问题是突然发生，还是有一个缓慢过程？

1. 突发
2. 慢慢发生

EXDA012 记忆问题是否越来越严重？

1. 是
2. 否

EXDA013 自 [ZIWTime] 以来，[XRName] 有没有摔倒过？

1. 有
2. 没有

EXDA014 [XRName] 有多少次摔倒受伤严重到需要接受治疗？ ______ [hc([0, 99], *int*, ∅)] 次

EXDA015 自 [ZIWTime] 之后，[XRName] 有没有过髋骨骨折？

1. 有
2. 没有

EXDA016 [XRName] 经常为身体疼痛而难受吗？是完全没有、有一点、有一些、比较多、还是非常多？

1. 完全没有
2. 有一点
3. 有一些
4. 比较多
5. 非常多

EXDA017 自 [ZIWTime] 以来直到去世，[XRName] 是否还有其他严重的疾病没有问到的？

1. 是
2. 否

EXDA018 这些疾病是什么？ ______

EXDA019 [XRName] 去世前一年内，是否至少有一个月的时间身体感到严重疲倦？

1. 是
2. 否

EXDA020 [XRName] 去世前一年内，是否至少有一个月的时间大小便失控？

1. 是
2. 否

EXD. 健康状况与功能（二）

EXDB001 请问 [XRName] 是否因为健康和记忆的原因，去世前三个月自己穿衣服有困难，包括从衣橱中拿出衣服，穿上衣服，扣上钮扣，系上腰带?

1. 没有困难
2. 有困难但仍可以完成
3. 有困难，需要帮助
4. 无法完成

EXDB002 穿衣服的时候是否有人帮助?

1. 有
2. 没有

EXDB003 需要人帮助穿衣服的时间有多久?

1. ______ [hc$((0,99], real, \varnothing)$] (**EXDB003_1**) 月
2. ______ [hc$((0,99], real, \varnothing)$] (**EXDB003_2**) 年
3. 自 ______ [hc$([1,120], int, \varnothing)$，sc$((1,100], int, \varnothing)$] (**EXDB003_3**) 岁以来
4. 自 ______ [hc$([1900,2020], int, \varnothing)$，sc$([1920,2020], int, \varnothing)$] (**EXDB003_4**) 年以来

EXDB004 请问 [XRName] 是否因为健康和记忆的原因，去世前三个月自己洗澡有困难?

1. 没有困难
2. 有困难但仍可以完成
3. 有困难，需要帮助
4. 无法完成

EXDB005 洗澡的时候是否有人帮助?

1. 有
2. 没有

EXDB006 需要人帮助洗澡的时间有多久?

1. ______ [hc$((0,99], real, \varnothing)$] (**EXDB006_1**) 月
2. ______ [hc$((0,99], real, \varnothing)$] (**EXDB006_2**) 年
3. 自 ______ [hc$([1,120], int, \varnothing)$，sc$((1,100], int, \varnothing)$] (**EXDB006_3**) 岁以来
4. 自 ______ [hc$([1900,2020], int, \varnothing)$，sc$([1920,2020], int, \varnothing)$] (**EXDB006_4**) 年以来

EXDB007 请问 [XRName] 是否因为健康和记忆的原因，去世前三个月自己吃饭有困难?

1. 没有困难
2. 有困难但仍可以完成
3. 有困难，需要帮助
4. 无法完成

EXDB008 吃饭的时候是否有人帮助?

1. 有
2. 没有

EXDB009 吃饭需要人帮助的时间有多久?

1. ______ [hc$((0,99], real, \varnothing)$] (**EXDB009_1**) 月
2. ______ [hc$((0,99], real, \varnothing)$] (**EXDB009_2**) 年

3. 自 ______ [hc$([1,120], int, \varnothing)$，sc$((1,100], int, \varnothing)$] **(EXDB009_3)** 岁以来
4. 自 ______ [hc$([1900,2020], int, \varnothing)$，sc$([1920,2020], int, \varnothing)$] **(EXDB009_4)** 年以来

EXDB010 请问 [XRName] 是否因为健康和记忆的原因，去世前三个月自己起床、下床有困难?
1. 没有困难
2. 有困难但仍可以完成
3. 有困难，需要帮助
4. 无法完成

EXDB011 起床、下床的时候是否有人帮助?
1. 有
2. 没有

EXDB012 需要人帮助起床、下床的时间有多久?
1. ______ [hc$((0,99], real, \varnothing)$] **(EXDB012_1)** 月
2. ______ [hc$((0,99], real, \varnothing)$] **(EXDB012_2)** 年
3. 自 ______ [hc$([1,120], int, \varnothing)$，sc$((1,100], int, \varnothing)$] **(EXDB012_3)** 岁以来
4. 自 ______ [hc$([1900,2020], int, \varnothing)$，sc$([1920,2020], int, \varnothing)$] **(EXDB012_4)** 年以来

EXDB013 请问 [XRName] 是否因为健康和记忆的原因，去世前三个月自己上厕所有困难?
1. 没有困难
2. 有困难但仍可以完成
3. 有困难，需要帮助
4. 无法完成

EXDB014 上厕所的时候是否有人帮助?
1. 有
2. 没有

EXDB015 需要人帮助上厕所的时间有多久?
1. ______ [hc$((0,99], real, \varnothing)$] **(EXDB015_1)** 月
2. ______ [hc$((0,99], real, \varnothing)$] **(EXDB015_2)** 年
3. 自 ______ [hc$([1,120], int, \varnothing)$，sc$((1,100], int, \varnothing)$] **(EXDB015_3)** 岁以来
4. 自 ______ [hc$([1900,2020], int, \varnothing)$，sc$([1920,2020], int, \varnothing)$] **(EXDB015_4)** 年以来

EXDB016 请问 [XRName] 是否因为健康和记忆的原因，去世前三个月自己做饭有困难?（定义：做饭我们定义为准备原材料，做饭菜，端上餐桌）
1. 没有困难
2. 有困难但仍可以完成
3. 有困难，需要帮助
4. 无法完成

EXDB017 做饭是否有人帮助?
1. 有
2. 没有

EXDB018 需要人帮助做饭的时间有多久?
1. ______ [hc$((0,99], real, \varnothing)$] **(EXDB018_1)** 月
2. ______ [hc$((0,99], real, \varnothing)$] **(EXDB018_2)** 年

3. 自 ______ [hc($[1,120], int, \varnothing$)，sc($(1,100], int, \varnothing$)] (**EXDB018_3**) 岁以来
4. 自 ______ [hc($[1900,2020], int, \varnothing$)，sc($[1920,2020], int, \varnothing$)] (**EXDB018_4**) 年以来

EXDB019 请问 [XRName] 是否因为健康和记忆的原因，去世前三个月自己去商店买食品杂货有困难?
1. 没有困难
2. 有困难但仍可以完成
3. 有困难，需要帮助
4. 无法完成

EXDB020 买食品杂货是否有人帮助?
1. 有
2. 没有

EXDB021 需要人帮他/她去商店买食品杂货的时间有多久?
1. ______ [hc($(0,99], real, \varnothing$)] (**EXDB021_1**) 月
2. ______ [hc($(0,99], real, \varnothing$)] (**EXDB021_2**) 年
3. 自 ______ [hc($[1,120], int, \varnothing$)，sc($(1,100], int, \varnothing$)] (**EXDB021_3**) 岁以来
4. 自 ______ [hc($[1900,2020], int, \varnothing$)，sc($[1920,2020], int, \varnothing$)] (**EXDB021_4**) 年以来

EXDB022 请问 [XRName] 是否因为健康和记忆的原因，去世前三个月自己打电话有困难?

【**访员注意**：如果已过世的曾经受访者从来没有做过，并且家属根据已过世受访者的表现判断因为健康或记忆原因肯定打不了电话，根据实际情况选择有困难需要帮助或者无法完成。】
1. 没有困难
2. 有困难但仍可以完成
3. 有困难，需要帮助
4. 无法完成

EXDB023 打电话是否有人帮助?

【**访员注意**：如果已过世的受访者从来没打过电话，选否。】
1. 有
2. 没有

EXDB024 需要人帮助打电话的时间有多久?
1. ______ [hc($(0,99], real, \varnothing$)] (**EXDB024_1**) 月
2. ______ [hc($(0,99], real, \varnothing$)] (**EXDB024_2**) 年
3. 自 ______ [hc($[1,120], int, \varnothing$)，sc($(1,100], int, \varnothing$)] (**EXDB024_3**) 岁以来
4. 自 ______ [hc($[1900,2020], int, \varnothing$)，sc($[1920,2020], int, \varnothing$)] (**EXDB024_4**) 年以来

EXDB025 请问 [XRName] 是否因为健康和记忆的原因，去世前三个月自己吃药有困难？吃药是指能记得什么时间吃和吃多少
1. 没有困难
2. 有困难但仍可以完成
3. 有困难，需要帮助
4. 无法完成

EXDB026 吃药是否有人帮助?
1. 有

2. 没有

EXDB027 需要人帮助他/她吃药的时间有多久?

1. ______ [hc$((0,99],real,\varnothing)$] (**EXDB027_1**) 月
2. ______ [hc$((0,99],real,\varnothing)$] (**EXDB027_2**) 年
3. 自 ______ [hc$([1,120],int,\varnothing)$, sc$((1,100],int,\varnothing)$] (**EXDB027_3**) 岁以来
4. 自 ______ [hc$([1900,2020],int,\varnothing)$, sc$([1920,2020],int,\varnothing)$] (**EXDB027_4**) 年以来

EXDB028 请问 [XRName] 是否因为健康和记忆的原因，去世前三个月自己管钱有困难?? 比如支付账单、记录支出项目、管理财物?

1. 没有困难
2. 有困难但仍可以完成
3. 有困难，需要帮助
4. 无法完成

EXDB029 管钱是否有人帮助?

1. 有
2. 没有

EXDB030 需要人帮他/她管钱的时间有多久?

1. ______ [hc$((0,99],real,\varnothing)$] (**EXDB030_1**) 月
2. ______ [hc$((0,99],real,\varnothing)$] (**EXDB030_2**) 年
3. 自 ______ [hc$([1,120],int,\varnothing)$, sc$((1,100],int,\varnothing)$] (**EXDB030_3**) 岁以来
4. 自 ______ [hc$([1900,2020],int,\varnothing)$, sc$([1920,2020],int,\varnothing)$] (**EXDB030_4**) 年以来

EXD. 健康状况与功能（三）

EXDB031 请问在以上困难中，谁帮助 [XRName] 最多?（在穿衣、洗澡、吃饭、起床、入厕、做饭、购物、打电话、吃药、管钱等困难中）（可多选）

【**访员注意**：养老院人员选项仅针对住在养老院或在养老院去世的受访者】

1. 配偶
2. 父母、岳父母、公公、婆婆
3. 子女、儿媳/女婿、孙子女/外孙子女
4. 兄弟姐妹及其配偶、子女，[XRName] 配偶的兄弟姐妹及其配偶、子女
5. 其他亲属
6. 雇佣人员（如保姆），共 ______ [hc$((0,99),int,-1)$, sc$([1,10),int,-1)$] (**EXDB031_1**) 位
7. 志愿者或者志愿机构人员
8. 养老院人员
9. 居家养老服务机构人员
10. 社区提供的帮助
11. 其他人员，请注明 ______ (**EXDB031_2**)

EXDB032 在父母、岳父母、公公、婆婆中，帮助 [XRName] 的是哪几位?（可多选）

1. 父亲
2. 母亲
3. 岳父/公公
4. 岳母/婆婆

EXDB033 帮助 [XRName] 的子女、儿媳/女婿、孙子女/外孙子女，是以下哪个子女家的？（可多选）

1-25. [XEChildAliveName[i]]
26-35. 其他子女，名字为 _____ (**EXDB033_1[i]**)

EXDB034[i] [XEHelperChild[i]] 家的哪些人亲自帮助 [XRName]？（可多选）

1. [XEHelperChild[i]] 本人
2. [XEHelperChild[i]] 的配偶
3. [XEHelperChild[i]] 的孩子，即 [XRName] 的（外）孙子女，亲自帮助 [XRName] 的 [XEHelperChild[i]] 的孩子有 _____ [hc$((0, 99), int, -1)$，sc$([1, 10), int, -1)$] (**EXDB034_1[i]**) 个

EXDB035 帮助 [XRName] 的兄弟姐妹及其配偶、子女，[XRName] 配偶的兄弟姐妹及其配偶、子女，是以下哪个兄弟姐妹家的？（可多选）

1-30. [XESibName[i]]
31-40. 其他兄弟姐妹，姓名为 _____ (**EXDB035_1[i]**)

EXDB036[i] [XEHelperSib[i]] 家的哪些人亲自帮助 [XRName]？（可多选）

1. [XEHelperSib[i]] 本人
2. [XEHelperSib[i]] 的配偶
3. [XEHelperSib[i]] 的孩子，即 [XRName] 的外甥外甥女、侄子侄女，亲自帮助 [XRName] 的 [XEHelperSib[i]] 的孩子有几个 _____ [hc$((0, 99), int, -1)$，sc$([1, 10), int, -1)$] (**EXDB036_1[i]**) 个

EXDB037 亲自为 [XRName] 提供帮助的其他亲属共有 _____ [hc$((0, 99), int, -1)$，sc$([1, 10), int, -1)$] 位，都是 [XRName] 的什么人？ _____ (**EXDB037_1**)

【访员注意：不知道请填写-1】

EXDB038 亲自为 [XRName] 提供帮助的其他人共有位 _____ [hc$((0, 99), int, -1)$，sc$([1, 10), int, -1)$] 位，都是 [XRName] 的什么人？ _____ (**EXDB038_1**)

【访员注意：不知道请填写-1】

EXDB039 在下面所列的所有帮助者中，请选择帮助 [XRName] 最多的 7 类人。

1-99. [XEHelper[i]]

EXDB040[i] 在 [XRName] 去世前的一个月内，[XEHelpList[i]] 帮助了 [XRName] 多少天？ _____ [hc$([0, 31], int, -1)$] 天

【访员注意：不知道请填写-1】

EXDB041[i] 在 [XEHelpList[i]] 帮助 [XRName] 的那些天，他/她大概每天花多少小时帮助 [XRName]？ _____ [hc$([0, 24], int, -1)$] 小时

【访员注意：少于一个小时请记为 1；
不知道请填写-1】

EXDB042[i] [XEHelpList[i]] 在照顾 [XRName] 的时候，是否和 [XRName] 住在一起？

1. 是
2. 否

EXDB043 [XRName] 使用以下辅助工具么？（可多选）

1. 拐杖

2. 代步器
3. 手动轮椅
4. 电动轮椅
5. 导尿管，导尿袋
6. 座便器
7. 以上都没有

[conflict(7, $[7]^c$)]

EXE. 医疗保健与保险（一）

【引语：现在我们想了解一下 [XRName] 去世前享受的健康保险或福利。】

EXEA001 [XRName] 去世的时候是否参加了以下医疗保险？（可多选）

1. 城镇职工基本医疗保险（医保）
2. 城乡居民基本医疗保险（合并城镇居民和新型农村合作医疗保险）
3. 城镇居民基本医疗保险
4. 新型农村合作医疗保险（合作医疗）
5. 公费医疗
6. 医疗救助
7. 商业医疗保险: 单位购买
8. 商业医疗保险: 个人购买
9. 城镇无业居民大病医疗保险
10. 长期护理保险
11. 其它医疗保险，请注明 ______ (**EXEA001_1**)
12. 没有保险

[conflict(12, $[12]^c$)]

EXEA001_verify 您确认 [XRName] 生前没有参加以上任何医疗保险吗？

【访员注意：如果代理人回答 [XRName] 其实参加过以上保险，请返回上一题进行修改，并选择 [XRName] 生前参加的对应医疗保险。】

1. 确认 [XRName] 生前没有参加以上任何医疗保险

EXEA002 [XRName] 过世前有没有参加补充医疗保险？（例如大病医疗等）

【访员注意：“补充医疗保险”指由于国家的基本医疗保险只能满足参保人的基本医疗需求，超过基本医疗保险范围之外的医疗需求可以通过补充医疗保险予以补充，是相对于基本医疗保险而言的，包括企业补充医疗保险、商业医疗保险、社会互助和社区医疗保险等多种形式。】

1. 有
2. 没有

EXEA003[i] [XRName] 的 [XEXMIns[i]] 是在哪里办的？

1. 去世之前的一般居住地：[EXB003_1]
2. （如户口不在一般居住地县/市）户籍所在地
3. 其他 ______ (**EXEA003_1[i]**) 省/市/县

EXEA008[i] [XRName] 是什么时候参加 [XEXMIns[i]] 的？ ______ [hc($[1900, 2020], int,$)] (**EXEA008_1[i]**) 年 ______ [hc($[1, 12], int, -1$)] (**EXEA008_2[i]**) 月

【访员注意：若受访者不清楚参保时间，请大概估计出参保年份。用 4 位数表示年，按照实际的月份填写月。例：1 月写作“1”，而不是“01”，12 月写作“12”。如果记不住月份，请填入“-1”】

EXEA009 [XRName] 去世前没有参加任何医疗保险的主要原因是什么 (可多选)?
1. 不需要
2. 认为保险费太贵了
3. 不知道该去哪办
4. 不相信健康保险机构
5. 没有合适的保险项目
6. 从没有想过这个问题
7. 其他原因 ______ (**EXEA009_1**)

EXEB001 [XRName] 以前是否参加过以下医疗保险?（可多选）
1. 城镇职工基本医疗保险（医保）
2. 城乡居民基本医疗保险（合并城镇居民和新型农村合作医疗保险）
3. 城镇居民基本医疗保险
4. 新型农村合作医疗保险（合作医疗）
5. 公费医疗
6. 医疗救助
7. 商业医疗保险: 单位购买
8. 商业医疗保险: 个人购买
9. 城镇无业居民大病医疗保险
10. 长期护理保险
11. 其它医疗保险，请注明 ______ (**EXEB001_1**)
12. 没有保险

[conflict$(12, [12]^c)$]

EXEB003[i] 请问 [XRName] 什么时候退出 [XEXPMIns[i]]？ ______ [hc$([1900, 2020], int,)$] (**EXEB003_1[i]**) 年 ______ [hc$([1, 12], int, -1)$] (**EXEB003_2[i]**) 月

【访员注意：若受访者不清楚退出保险时间，请大概估计出退保年份。用 4 位数表示年，按照实际的月份填写月。例：1 月写作“1”，而不是“01”，12 月写作“12”。如果记不住月份，请填入“-1”。】

EXEB004[i] 请问 [XRName] 为什么会退出 [XEXPMIns[i]]？
1. 单位不存在了
2. 本地不再提供这种保险
3. 我从单位辞职/被辞退了
4. 自己不想参加了
5. 家人不愿意给参加了
6. 因为保费太贵了
7. 其它，请注明 ______ (**EXEB004_1[i]**)

EXEB005 [XRName] 去世后，家人或朋友是否从医保账户中取钱?
1. 是
2. 否

EXEB006 这笔钱有多少? ______ [hc$([0, \infty), real, -1)$], sc$([0, 50000), real, -1)$]

EXE. 医疗保健与保险（二）

EXEC001 自上次访问到去世的那段时间里，[XRName] 最近一次常规体检是什么时候?

【**访员注意**：常规体检是为了让自己掌握自身健康状况而进行的全面健康检查。去医院只测量血压、血糖或只是为了检查需求进行血常规等检查不算常规体检。用 4 位数表示年，若记不清楚常规体检时间，请大概估计出体检年份。按照实际的月份填写月。例：1 月写作“1”，而不是“01”，12 月写作“12”。如果记不住月份，请填入“-1”】

1. ______ [hc($[1900, 2020], int,$)] (**EXEC001_1**) 年 ______ [hc($[1, 12], int, -1$)] (**EXEC001_2**) 月
2. 自上次访问以来没有参加过常规体检

EXED030 除了住院以外，[XRName] 去世前一个月去医疗机构看过门诊或者接受过上门医疗服务有几次? ______ [hc($[0, \infty), int,$)，sc($[0, 10], int,$)] 次

EXED031 除了住院以外，[XRName] 去世前一个月去医疗机构看过门诊或者接受过上门医疗服务的总费用大概是多少? ______ [hc($[0, \infty), real, -1$)，sc($[0, 30000], real, -1$)，ub($[-1], [50, 100, 200, 500, 1000]$)] 元

【**访员注意**：如果受访者无法回答，请填“-1”。】

EXED032 在这些就诊费用里面，[XRName] 自己支付了多少钱?

【**访员注意**：如果受访者无法回答，请填“-1”。】

1. 其中自付部分 ______ [hc($(0, \infty), real, -1$)，sc($(0, 30000], real, -1$)，ub($[-1], [50, 100, 250, 500, 800]$)] (**EXED032_1**) 元
2. 没有付任何钱

EXED033 就诊的费用（包含诊疗费和药费）使用或将使用哪种医疗保险报销?（可多选）

1-11. [XEXMIns[i]]

12. 单位报销
13. 没有保险
14. 不适用

EXEF001 去世前一个月，[XRName] 是否自己买药吃?

【**访员注意**：此处不包括凭处方取药的情况，但服用别人送的药或自己存的药也算自己买药吃。】

1. 是
2. 否

EXEF002 去世前一个月，[XRName] 自己买药的花费大概是多少? 包括自付和报销部分的总花费。 ______ [hc($[0, \infty), real, -1$)，sc($[0, 2000], real, -1$)，ub($[-1], [10, 30, 100, 200, 300]$)] 元

【**访员注意**：如果受访者无法回答，请填“-1”。】

EXEF003 除了报销的部分，[XRName] 自己支付了多少钱?

【**访员注意**：如果受访者无法回答，请填“-1”。】

1. 其中自付部分 ______ [hc($(0, \infty), real, -1$)，sc($(0, 2000], real, -1$)，ub($[-1], [10, 30, 70, 100, 200]$)] (**EXEF003_1**) 元
2. 没有付任何钱

EXEF005 [XRName] 自我治疗的费用使用了哪种医疗保险报销?（可多选）

1-11. [XEXMIns[i]]

12. 单位报销

13. 没有保险
14. 不适用

EXEG000 [XRName] 是在医院去世的吗?
1. 是
2. 否

EXEG001 之前您告诉我们 [XRName] 是在医院去世的，去世之前作为那所医院的病人住院多长时间?

【**访员注意**：不足 1 小时的话，请填写 1 小时】
1. ______ [hc$([0,24), real, -1)$] (**EXEG001_1**) 小时
2. ______ [hc$([1,7), real, -1)$] (**EXEG001_2**) 天
3. ______ [hc$([1,5), real, -1)$] (**EXEG001_3**) 周
4. ______ [hc$([1,12), real, -1)$] (**EXEG001_4**) 月
5. ______ [hc$([1,\infty), real, -1)$] (**EXEG001_5**) 年

EXEG002 [XRName] 因何住院，外科手术、其他治疗方式、一些方式减轻症状还是其他?
1. 外科手术
2. 其他治疗方式
3. 减轻症状
4. 其他 ______ (**EXEG002_1**)

EXEG003 除了去世那次住院，[XRName] 去世前一个月是否还住过院?
1. 是
2. 否

EXEG004 [XRName] 去世前一年，是否住过院?
1. 是
2. 否

EXEG005 [XRName] 去世前一年住过几次院 (包括去世时的住院)? ______ [hc$((0,\infty), int,)$] 次

EXEG006 住院时，[XRName] 是否住过特护病房 ICU?
1. 是，去世前一年住特护病房 ICU 的总天数是 ______ [hc$([1,366], int, -1)$]，sc$([1,180], int, -1)$] (**EXEG006_1**) 天
2. 否

EXEG007 住院时，[XRName] 是否使用过生命支持设备，比如呼吸器、人工肝或人工肺?（可多选）
1. 呼吸器
2. 人工肝
3. 人工肺
4. 以上都没有

[conflict$(4,[4]^c)$]

EXEG008 住院时，[XRName] 是否做过肾透析?
1. 是
2. 否

EXEG009 住院时，[XRName] 是否用过抗生素治疗肺炎或者其他传染疾病?

1. 是
2. 否

EXEG010 [XRName] 去世前一年，所有住院花费的总费用大概是多少，只包括付给医院的费用，不包括陪护的工资、自己或家人的交通费和住宿费，但包括医院病房费？ ______ [hc($[0, \infty), int, -1$)，sc($[0, 300000], int, -1$)，ub($[-1], [1500, 3000, 7000, 15000, 30000]$)] 元

【访员注意：如果受访者无法回答，请填“-1”。】

EXEG011 在这些住院费用里面，[XRName] 自己支付了多少钱？

【访员注意：如果受访者无法回答，请填“-1”。】

1. 其中自付部分 ______ [hc($(0, \infty), real, -1$)，sc($(0, 300000], real, -1$)，ub($[-1], [1000, 2000, 5000, 10000, 20000]$)] (**EXEG011_1**) 元
2. 没有付任何钱

EXEG012 住院费用使用或将使用哪种医疗保险报销？（可多选）

1-11. [XEXMIns[i]]
12. 单位报销
13. 没有保险
14. 不适用

EXF. 工作与退休

EXF001 根据我们的记录，[XRName] 在上一次调查 [ZIWTime] 那时在工作，[XRName] 是在哪年哪月停止工作的？

【访员注意：如果受访者无法回答年月，请填“-1”。】

1. ______ [hc([ZIWYear,XIWYear], $int, -1$)] (**EXF001_1**) 年 ______ [hc($[1, 12], int, -1$)] (**EXF001_2**) 月
2. [XRName] 工作直到去世

995. 上一轮调查时 [XRName] 没在工作
997. 不知道
999. 拒绝回答

EXF002 在 [XRName] 停止工作的前一周，[XRName] 工作了几天？ ______ [hc($[1, 7], int, -1$)] 天

【访员注意：如果受访者无法回答，请填“-1”。】

EXF003 在这周 [XRName] 工作的日子里，[XRName] 平均每天工作几个小时？ ______ [hc($[1, 24], int, \varnothing$)，sc($[1, 16], int, \varnothing$)] (**EXF003_1**) 小时/每天，其中：农业活动 ______ [hc([0,EXF003_1], $int, -1$)] (**EXF003_2**) 小时/每天；非农业活动 ______ [hc([0,EXF003_1], $int, -1$)] (**EXF003_3**) 小时/每天。

【访员注意：农业和非农业工作时长之和应等于总工作时长。
如果受访者无法回答，请填“-1”。】

EXF004 根据我们的记录，在上一次调查时，[XRName] 还没有办理退休手续，请问 [XRName] 在去世前办理了退休手续吗？

1. 是
2. 否

997. 不知道
999. 拒绝回答

EXFN. 养老金

EXF005 [XRName] 去世后，一共从养老金或养老保险账户中取出了多少钱？ ______ [hc $([0, 10000000], int, -1)$] 元
【访员注意：如果代理人无法回答，请填“-1”。】

EXF006 [XRName] 去世后，是否从养老保险或养老金中获得了一次性的抚恤金或丧葬费？
1. 是
2. 否

EXF007 这笔钱有多少？ ______ [hc $([10, 10000000], int, -1)$] 元
【访员注意：如果代理人无法回答，请填“-1”。】

EXF008 [XRName] 去世后，是否从退休金/养老保险以外的其他地方获得了一次性的抚恤金或丧葬费？
1. 是
2. 否

EXF009 这笔钱有多少？ ______ [hc $([10, 10000000], int, -1)$] 元
【访员注意：如果代理人无法回答，请填“-1”。】

EXF010 [XRName] 生前是否有上人寿保险？
1. 是
2. 否

EXF011 [XRName] 去世后，人寿保险赔付了多少钱？ ______ [hc $([10, 10000000], int, -1)$] 元
【访员注意：如果代理人无法回答，请填“-1”。】

EXF012 [XRName] 生前是否有上商业养老保险？
1. 是
2. 否

EXF013 [XRName] 去世后，从商业养老保险取出了多少钱？ ______ [hc $([10, 10000000], int, -1)$] 元
【访员注意：如果代理人无法回答，请填“-1”。】

EXG. 收入、支出与资产

EXG001 [XRName] 去世时，是否有房产？如果有房产，其所拥有房产的总价值是多少？如果仅有部分产权的，计算其所拥有部分的房产价值。有多处房产的，计算几所总价值。
【访员注意：如果受访者拒绝回答或者忘记了，填入“-1”。】
1. 有房产，总价值为 ______ [hc $((0, \infty), int, -1)$，sc $((0, 1000000], int, -1)$] (**EXG001_1**) 元
2. 没有房产

EXG002 [XRName] 的房产是如何分配的，他们各自的继承比例是多少？如果是把房产卖了，也请计算各自分配的比例（可多选）。
【访员注意：如果受访者拒绝回答或者忘记了，填入“-1”。】
1. 配偶 ______ [hc $((0, 100], real, -1)$] (**EXG002_1**) %
2. 子女、女婿、儿媳

3. 兄弟姐妹 ______ [hc((0, 100], *real*, −1)] (**EXG002_3**) %
4. 其他亲戚 ______ [hc((0, 100], *real*, −1)] (**EXG002_4**) %
5. 父母、岳父母 ______ [hc((0, 100], *real*, −1)] (**EXG002_5**) %
6. 孙子女、外孙子女
7. 朋友 ______ [hc((0, 100], *real*, −1)] (**EXG002_7**) %
8. 慈善机构 ______ [hc((0, 100], *real*, −1)] (**EXG002_8**) %
9. 其他，请注明 ______ (**EXG002_9_1**)， ______ [hc((0, 100], *real*, −1)] (**EXG002_9**) %
10. 没有分配

[conflict(10, $[10]^c$)]

EXG003 继承房产的是哪些子女/子女的配偶？子女的配偶算作子女部分。如果继承房产的子女不在选项里，请添加 [XRName] 的子女名字（可多选）。

1-25. [ZChildName[*i*]]

26-35. 其他子女，名字为 ______ (**EXG003_1[i]**)

EXG004[*i*] [XEXGChildList1[*i*]] 及其配偶获得房子价值的百分比是 ______ [hc((0, 100], *real*, −1)] %。

【访员注意：如果受访者拒绝回答或者忘记了，填入“-1”。】

EXG007 孙子女/外孙子女是哪些子女的孩子？如果继承房产的孙子女的父亲/外孙子女的母亲不在子女选项里，请添加 [XRName] 的子女名字（可多选）。

1-25. [ZChildName[*i*]]

26-35. 其他，子女名字为 ______ (**EXG007_1[i]**)

EXG008[*i*] [XEXGChildList4[*i*]] 的孩子获得房子价值的百分比是 ______ [hc((0, 100], *real*, −1)] %。

【访员注意：如果受访者拒绝回答或者忘记了，填入“-1”。】

EXG009 除了前面提到的房产、医保账户、退休金/养老金账户及相关抚恤金、其他抚恤金或丧葬费、人寿保险赔付款项和商业养老保险之外，[XRName] 去世后有没有留下现金？

1. 是
2. 否

EXG010 [XRName] 去世后，留下的现金有多少？ ______ [hc((0, ∞), *int*, −1)，sc((0, 100000), *int*, −1)] 元

【访员注意：如果受访者拒绝回答或者忘记了，填入“-1”。】

EXG011 除了前面提到的房产、医保账户、退休金/养老金账户及相关抚恤金、其他抚恤金或丧葬费、人寿保险赔付款项和商业养老保险之外，[XRName] 去世后有没有留下存款？

1. 是
2. 否

EXG012 [XRName] 去世后，留下的存款有多少？ ______ [hc((0, ∞), *int*, −1)，sc((0, 100000), *int*, −1)] 元

【访员注意：如果受访者拒绝回答或者忘记了，填入“-1”。】

EXG013 除了前面提到的房产、医保账户、退休金/养老金账户及相关抚恤金、其他抚恤金或丧葬费、人寿保险赔付款项和商业养老保险之外，[XRName] 去世后有没有留下股票、基金、国库券等金融资产？

1. 是
2. 否

EXG014 [XRName] 去世后，留下的股票、基金、国库券等金融资产有多少？ ______ [hc((0, ∞), *int*, −1)，sc((0, 100000), *int*, −1)] 元

【访员注意：如果受访者拒绝回答或者忘记了，填入“-1”。】

EXG015 除了前面提到的房产、医保账户、退休金/养老金账户及相关抚恤金、其他抚恤金或丧葬费、人寿保险赔付款项和商业养老保险之外，[XRName] 去世后有没有留下实物？

1. 是
2. 否

EXG016 [XRName] 去世后，留下的实物价值多少？ ______ [hc((0, ∞), *int*, −1)，sc((0, 100000), *int*, −1)] 元

【访员注意：如果受访者拒绝回答或者忘记了，填入“-1”。】

EXG017 除了前面提到的房产、医保账户、退休金/养老金账户及相关抚恤金、其他抚恤金或丧葬费、人寿保险赔付款项、商业养老保险、现金、存款、金融资产、实物之外，[XRName] 去世后有没有留下其他财产？有的话，是多少？ ______ [hc([0, ∞), *int*, −1)，sc([0, 100000), *int*, −1)] 元

【访员注意：如果受访者拒绝回答或者忘记了，填入“-1”。】

EXG018 除去房产以外，[XRName] 留下的以下遗产：医保账户，退休金/养老金及相关抚恤金，丧葬费，人寿保险赔付，现金，存款，股票基金国库券等金融资产，实物价值以及其他。总数 [XEXGTotalValue] 是否正确？

【访员注意：如果受访者拒绝回答或者忘记了，填入“-1”。】

1. 是
2. 否，应该是 ______ [hc([0, ∞), *int*, −1)，sc([0, 200000], *int*, −1)] (**EXG018_1**) 元

EXG019 在 [XRName] 去世之前，他/她有没有立遗嘱？

1. 是
2. 否

EXG020 [XRName] 的遗嘱有没有经过遗嘱公证？

1. 是
2. 否

EXG021 在 [XRName] 的遗嘱中，他/她对遗产是如何分配的？不包括房产（可多选）。

【访员注意：如果受访者拒绝回答或者忘记了，填入“-1”。】

1. 配偶 ______ [hc((0, 100], *real*, −1)] (**EXG021_1**) %
2. 子女、女婿、儿媳
3. 兄弟姐妹 ______ [hc((0, 100], *real*, −1)] (**EXG021_3**) %
4. 其他亲戚 ______ [hc((0, 100], *real*, −1)] (**EXG021_4**) %
5. 父母、岳父母 ______ [hc((0, 100], *real*, −1)] (**EXG021_5**) %
6. 孙子女、外孙子女
7. 朋友 ______ [hc((0, 100], *real*, −1)] (**EXG021_7**) %
8. 慈善机构 ______ [hc((0, 100], *real*, −1)] (**EXG021_8**) %
9. 其他，请注明 ______ (**EXG021_9_1**)， ______ [hc((0, 100], *real*, −1)] (**EXG021_9**) %
10. 没有分配

[conflict(10, $[10]^c$)]

EXG022 是哪些子女/子女的配偶？子女的配偶算作子女部分。如果遗嘱中提到的子女不在选项里，

请添加 [XRName] 的子女名字（可多选）。
1-25. [ZChildName[i]]
26-35. 其他，子女名字为 ______ (**EXG022_1[i]**)

EXG023[i] [XEXGChildList2[i]] 及其配偶在遗嘱中获得遗产的百分比是 ______ [hc((0, 100], *real*, −1)] %。
【访员注意：如果受访者拒绝回答或者忘记了，填入“-1”。】

EXG026 孙子女/外孙子女是哪些子女的孩子？如果遗嘱中提到的孙子女的父亲/外孙子女的母亲不在选项里，请添加 [XRName] 的子女名字（可多选）。
1-25. [ZChildName[i]]
26-35. 其他，子女名字为 ______ (**EXG026_1[i]**)

EXG027[i] [XEXGChildList5[i]] 的孩子在遗嘱中获得遗产的百分比是 ______ [hc((0, 100], *real*, −1)] %。
【访员注意：如果受访者拒绝回答或者忘记了，填入“-1”。】

EXG028 在遗嘱中，分给兄弟姐妹的遗产是在兄弟姐妹间平均分配的吗？
1. 是
2. 否

EXG029 [XRName] 留下的所有遗产，去掉丧葬等的花费，是否还有遗产被继承？最后被继承的部分价值是多少？不包括房产
【访员注意：如果受访者拒绝回答或者忘记了，填入“-1”。】
1. 还有遗产被继承，价值 ______ [hc((0, ∞), *int*, −1)，sc((0, 200000], *int*, −1)] (**EXG029_1**) 元
2. 没有遗产被继承

EXG030 分 [XRName] 的遗产时，是不是完全按照他/她的遗嘱执行的？
1. 是
2. 否

EXG031 [XRName] 的遗产是如何分配的？不包括房产（可多选）。
【访员注意：如果受访者拒绝回答或者忘记了，填入“-1”。】
1. 配偶 ______ [hc((0, 100], *real*, −1)] (**EXG031_1**) %
2. 子女、女婿、儿媳
3. 兄弟姐妹 ______ [hc((0, 100], *real*, −1)] (**EXG031_3**) %
4. 其他亲戚 ______ [hc((0, 100], *real*, −1)] (**EXG031_4**) %
5. 父母、岳父母 ______ [hc((0, 100], *real*, −1)] (**EXG031_5**) %
6. 孙子女、外孙子女
7. 朋友 ______ [hc((0, 100], *real*, −1)] (**EXG031_7**) %
8. 慈善机构 ______ [hc((0, 100], *real*, −1)] (**EXG031_8**) %
9. 其他，请注明 ______ (**EXG031_9_1**)，______ [hc((0, 100], *real*, −1)] (**EXG031_9**) %
10. 没有分配

[conflict(10, [10]^c)]

EXG032 是哪些子女/子女的配偶？子女的配偶算作子女部分。如果继承遗产的子女不在选项里，请添加 [XRName] 的子女名字（可多选）。
1-25. [ZChildName[i]]

26-35. 其他，子女名字为 ______ (**EXG032_1[i]**)

EXG033[i] [XEXGChildList3[i]] 及其配偶分得遗产的百分比是 ______ [hc((0, 100], *real*, −1)] %。

【**访员注意**：如果受访者拒绝回答或者忘记了，填入“-1”。】

EXG036 孙子女/外孙子女是哪些子女的孩子？如果继承遗产的孙子女的父亲/外孙子女的母亲不在子女选项里，请添加 [XRName] 的子女名字（可多选）。

1-25. [ZChildName[i]]
26-35. 其他，子女名字为 ______ (**EXG036_1[i]**)

EXG037[i] [XEXGChildList6[i]] 的孩子分得遗产的百分比是 ______ [hc((0, 100], *real*, −1)] %。

【**访员注意**：如果受访者拒绝回答或者忘记了，填入“-1”。】

EXK. 殡葬

K01 [XRName] 的丧葬方式是什么?

1. 火葬
2. 土葬
3. 天葬
4. 水葬
5. 其他，请注明 ______ (**K01_1**)

K02 [XRName] 葬在何处?

1. 自家的承包地
2. 村集体的公墓
3. 商业经营的公墓
4. 荒地
5. 骨灰存放在殡仪馆
6. 其他，请注明 ______ (**K02_1**)

K03 坟墓/墓地花了多少钱？没有花钱请填 0。

1. 共 ______ [hc([0, ∞), *real*, −1)，sc([0, 50000), *real*, −1)] (**K03_1**) 元
2. ______ [hc([0, ∞), *real*, −1)，sc([0, 30000), *real*, −1)] (**K03_2**) 元/年

K04 坟墓/墓地是哪一年购买的？ ______ [hc([1900, 2020], *int*,)] 年

K06 谁出的钱购置墓地？可多选。

1. [XRName] 的配偶
2-26. [XKChildName[i]]
27-51. [XKChildAndS[i]]
52. 孙子/外孙子
53. 孙女/外孙女
54. 亲属
55. 单位（包括村集体）
56. 保险
57. 其他人员

K07 K06 之中选择了孙子女或外孙子女，请具体说明，[XRName] 哪个孩子是这个孙子女或外孙子女的父母？

1-25. [ZChildName[i]]

K08 K06 之中选择了亲属或其他人员，请具体说明，他/她是受访者的什么人？______

K09 [XRName] 的丧葬费共花费了多少钱？包括棺材/骨灰盒费用、遗照、出殡仪式、做法事等费用，不包括墓地/殡仪馆费用。共 ______ [hc($[0, \infty), real, -1$)，sc($[0, 100000), real, -1$)，ub($[-1], [1000, 3000, 5000, 8000, 10000]$)] 元

K10 丧葬费谁出的钱？可多选。

1. [XRName] 的配偶
2-26. [XKChildName[i]]
27-51. [XKChildAndS[i]]
52. 孙子/外孙子
53. 孙女/外孙女
54. 亲属
55. 单位（包括村集体）
56. 保险
57. 其他人员

K11 K10 之中选择了孙子女或外孙子女，请具体说明，[XRName] 哪个孩子是这个孙子女或外孙子女的父母？

1-25. [ZChildName[i]]

K12 K10 之中选择了亲属或其他人员，请具体说明，他/她是受访者的什么人？______

K13 给 [XRName] 办丧事时，家里共收到多少礼金？共 ______ [hc($[0, \infty), int, -1$)，sc($[0, 50000], int, -1$)，ub($[-1], [1000, 3000, 5000, 8000, 10000]$)] 元

EXV. 疫情相关

EXV001 [XRName] 去世前是否曾被认定为新冠肺炎确诊病例？

1. 是
2. 否
999. 拒绝回答

EXV002 [XRName] 去世前是否曾被认定为新冠肺炎疑似病例？

1. 是
2. 否
999. 拒绝回答

EXV003 为治疗新冠肺炎，[XRName] 住院了吗？在医院一共住了 ______ [hc($[0, 250], int, -1$)] 天？

【**访员注意**：没住院填“0”。如果受访者无法回答，请填“-1”。】

EXV004 [XEXVAHospitalizationExcluded] [XRName] 是否由于以下原因曾被隔离或医学观察？居家隔离和封楼都算被隔离。（多选题）

1. 旅行出差（包括春节后去某地上班）

2. 是新冠病例密切接触者
3. 住宅或住宅楼被封闭（不含小区封闭）
4. 去医疗机构诊疗时或出院后被要求隔离
5. 核酸检测显示阳性
6. 没有隔离经历（排他选项）
997. 不知道
999. 拒绝回答

[conflict(6, 997, 999, $[6, 997, 999]^c$)]

EXV005 [XRName] 总共被隔离了 ______ [hc([1, 250], *int*, −1)，sc([1, 14], *int*, −1)] 天?

【**访员注意**：如果有多次隔离经历，请记录加总后的时长。如果受访者无法回答，请填“-1”。】

EXV006 [XRName] 被隔离的场所是在?（多选题）

1. 医院
2. 酒店等集中的医学观察点
3. 自己住处
4. 其他：______ (**EXV006_1**)
999. 拒绝回答

[conflict(999, $[999]^c$)]

EXV007 在 [XRName] 的隔离经历中，[XRName] 在隔离住所是否曾有他人陪同，共同隔离?（访员注意：如果有多次隔离经历，只要有一次隔离有他人陪同，就可以认为“是”）

1. 是
2. 否
999. 拒绝回答

EXV008 [XRName] 是否接受过新冠病毒检测?

【**访员注意**：如果受访者无法回答月份，请填“-1”。】

1. 是，最近一次检测时间是在 ______ [hc([1, 12], *int*, −1)] (**EXV008_1**) 月份
2. 否
999. 拒绝回答

EXV009 疫情期间，[XEXVCNotInQuarantine] ，[XRName] 是否曾经因为担心染病好几天都没出门?[XRName] 最长曾经连续几天没出过门? ______ [hc([0, 250], *int*, −1)] 天（每天都出门，填“0”）

【**访员注意**：如果受访者无法回答，请填“-1”。】

EXV010 疫情期间，[XRName] 是否曾经需要去看病，包括去看牙，但受疫情影响，被迫推迟，或没能够去看?

1. 是
2. 否

EXV011 为什么 [XRName] 看病被推迟，或者没能够去看呢?（多选题，选项免读）

1. 没法预约，或医院的常规挂号都取消了
2. 医院把常规的诊疗安排都改期了
3. [XRName] 自己决定可以等等再去看
4. [XRName] 害怕去医院
5. 其他，请说明 ______ (**EXV011_1**)

EXV012 可以具体说明一下疫情期间 [XRName] 是想去看什么病或者想得到什么医疗服务，结果被推迟或取消了吗？（多选题）

【**访员注意**：请读出每一个选项，勾选所有符合的选项】

1. 需要住院的大的手术
2. 门诊手术或者日间病房就可以做的小的手术
3. 去看医生普通门诊
4. 去拿处方药
5. 去看牙，口腔治疗
6. 其他，请简单说明 ______ (**EXV012_1**)

EXV013 [XRName] 疫情期间想去看门诊，是因为新出现的症状或疾病，或者是因为要治疗已经得了的疾病，还是常规的体检筛查？（多选题）

1. 新出现的症状或疾病
2. 治疗已经得了的疾病
3. 常规的体检筛查

EXV014 在您看来，[XRName] 去世是否和新冠传染病疫情有关？（多选题）

1. 确诊感染新冠病毒去世
2. 因为疫情导致无法及时接受常规诊疗
3. 因为疫情导致无法及时接受急诊治疗
4. 因为疫情导致无法接受护理和照料
5. 因为疫情导致的意外
6. 其他原因，请简要说明 ______ (**EXV014_1**)
7. 去世和传染病疫情无关

[conflict(7,[7]^c)]

VA. 死因分析

EX004 回答退出问卷的代理人姓名 ______

EX005 [XRName] 和代理人是什么关系？

1. 父亲
2. 母亲
3. 配偶
4. 兄弟姐妹
5. 其他亲戚（请注明）______ (**EX005_1**)
6. 没有亲属关系

EX006 回答问卷的代理人联系方式 ______

VAS41 您能告诉我导致 [XRName] 死亡的疾病或事件是什么吗？ ______

如果有相关医学文件（不包含死亡证明，已询问过），比如病历、医生诊断书等，在得到允许后，请对关键医学文件拍照记录。 ______ (**VAS41_1**)

VAS42 您能告诉我 [XRName] 的第一死因是 ______

如果有相关医学文件（不包含死亡证明和上题已采集的文件，已询问过），比如病历、医生诊断书等，在得到允许后，请对关键医学文件拍照记录。 ______ (**VAS42_1**)

VAS43 您能告诉我 [XRName] 的第二死因是 ______

【**访员注意**：如果受访者无法回答，填“-1”。】

辅助变量定义

XRDeathTime 去世时间：某年某月某日

```
if (!empty("XRDeathYear") && !empty("XRDeathMonth") && !empty("XRDeathDate") &&
    !equal("XRDeathMonth", "-1") && !equal("XRDeathDate", "-1")) {
    add("XRDeathTime", value("XRDeathYear")+"年"+value("XRDeathMonth")+"月"+value("XRDeathDate")+"日")
}

if (empty("XRDeathYear") || empty("XRDeathMonth") || empty("XRDeathDate") || equal("XRDeathMonth", "-1")
↪   ||
    equal("XRDeathDate", "-1")) {
    add("XRDeathTime", value("EXB001_1")+"年"+value("EXB001_2")+"月"+value("EXB001_3")+"日")
}
```

XAliveResidenceFull 生前一般居住地选项 1. 中国大陆五级地址 + 门牌号

```
if (equal("EXB003", "1")) {
    add("XAliveResidenceFull",value("EXB003_1")+value("EXB003_2")+value("EXB003_3"))
}
```

XEZDisease 根据加载变量生成上期患病情况

```
if (equal("ZDisease[4]", "1")) {
    add("XEZDisease[1]", "1")
}
if (equal("ZDisease[5]", "1")) {
    add("XEZDisease[2]", "1")
}
if (equal("ZDisease[7]", "1")) {
    add("XEZDisease[3]", "1")
}
if (equal("ZDisease[8]", "1")) {
    add("XEZDisease[4]", "1")
}
if (equal("ZDisease[11]", "1")) {
    add("XEZDisease[5]", "1")
}
```

XEChroDisType 慢性病类型

```
add("XEChroDisType", ["癌症等恶性肿瘤（不包括轻度皮肤癌）", "慢性肺部疾患如慢性支气管炎或肺气肿、肺心病
↪ （不包括肿瘤或癌）", "心脏病（如心肌梗塞、冠心病、心绞痛、充血性心力衰竭和其他心脏疾病）", "中风",
↪  "情感及精神问题"])
```

XEDisease[3]是否患心脏病

```
if ( equal("EXDA001[3]", "1") || equal("XEZDisease[3]", "1") ) {
    add("XEDisease[3]", "1")
} else {
    add("XEDisease[3]", "2")
}
```

XEDisease[1]是否患癌症

```
if (equal("EXDA001[1]", "1") || equal("XEZDisease[1]", "1")) {
    add("XEDisease[1]", "1")
} else {
    add("XEDisease[1]", "2")
}
```

XEDisease[4]是否患中风

```
if (equal("EXDA001[4]", "1") || equal("XEZDisease[4]", "1")) {
    add("XEDisease[4]", "1")
} else {
```

```
    add("XEDisease[4]", "2")
}
```

XEHelperSelect 是否有人帮助

```
if (equal("EXDB002", "1") || equal("EXDB005", "1") || equal("EXDB008", "1") || equal("EXDB011", "1") ||
↪  equal("EXDB014", "1") || equal("EXDB017", "1") || equal("EXDB020", "1") || equal("EXDB023", "1") ||
↪  equal("EXDB026", "1") || equal("EXDB029", "1") ) {
    add("XEHelperSelect", "1")
} else {
    add("XEHelperSelect", "0")
}
```

XEHelperChild 提供帮助的子女姓名列表

```
for (var i1 = 1; i1 <= 25; i1++) {
    add("XEHelperChild[i1]", value("XEChildAliveName[i1]"))
}
for (var i1 = 26; i1 <= 35; i1++) {
    add("XEHelperChild[i1]", value("EXDB033_1[i1]"))
}
```

XESibName 生成兄弟姐妹姓名列表 生成兄弟姐妹姓名列表

```
for (var i1 = 1; i1 <= 15; i1++) {
    if (!empty("ZSibName[i1]")) {
        add("XESibName[i1]", pre("XRName")+"的兄弟姐妹"+pre("ZSibName[i1]"))
    }
}
for (var i1 = 1; i1 <= 15; i1++) {
    if (!empty("ZSibNameS[i1]")) {
        add("XESibName["+(i1+15)+"]", pre("XRName")+"的配偶的兄弟姐妹"+pre("ZSibNameS[i1]"))
    }
}
```

XEHelperSib 提供帮助的兄弟姐妹姓名

```
for (var i1 = 1; i1 < 31; i1++) {
    add("XEHelperSib[i1]", value("XESibName[i1]"))
}
for (var i1 = 31; i1 <= 40; i1++) {
    add("XEHelperSib[i1]", value("EXDB035_1[i1]"))
}
```

XEHelperNum 提供帮助者的数量

```
add("XEHelperNum", "0")
if (selected("EXDB031", "1")) {
    add("XEHelperNum", value("XEHelperNum")+1)
    add("XEHelper["+value("XEHelperNum")+"]", "配偶")
}
if (selected("EXDB031", "5")) {
    add("XEHelperNum", value("XEHelperNum")+1)
    add("XEHelper["+value("XEHelperNum")+"]", "其他亲属")
}

if (selected("EXDB031", "6")) {
    add("XEHelperNum", value("XEHelperNum")+1)
    add("XEHelper["+value("XEHelperNum")+"]", "雇佣人员")
}

if (selected("EXDB031", "7")) {
    add("XEHelperNum", value("XEHelperNum")+1)
    add("XEHelper["+value("XEHelperNum")+"]", "志愿者")
}

if (selected("EXDB031", "8")) {
    add("XEHelperNum", value("XEHelperNum")+1)
    add("XEHelper["+value("XEHelperNum")+"]", "养老院人员")
}

if (selected("EXDB031", "9")) {
    add("XEHelperNum", value("XEHelperNum")+1)
```

```
    add("XEHelper["+value("XEHelperNum")+"]", "居家养老服务机构人员")
}

if (selected("EXDB031", "10")) {
    add("XEHelperNum", value("XEHelperNum")+1)
    add("XEHelper["+value("XEHelperNum")+"]", "社区")
}

if (selected("EXDB031", "11")) {
    add("XEHelperNum", value("XEHelperNum")+1)
    add("XEHelper["+value("XEHelperNum")+"]", "其他人员")
}
if (selected("EXDB032", "1")) {
    add("XEHelperNum", value("XEHelperNum")+1)
    add("XEHelper["+value("XEHelperNum")+"]", "父亲")
}
if (selected("EXDB032", "2")) {
    add("XEHelperNum", value("XEHelperNum")+1)
    add("XEHelper["+value("XEHelperNum")+"]", "母亲")
}
if (selected("EXDB032", "3")) {
    add("XEHelperNum", value("XEHelperNum")+1)
    add("XEHelper["+value("XEHelperNum")+"]", "岳父/公公")
}
if (selected("EXDB032", "4")) {
    add("XEHelperNum", value("XEHelperNum")+1)
    add("XEHelper["+value("XEHelperNum")+"]", "岳母/婆婆")
}
for (var i1 = 1; i1 < 26; i1++) {
    if (selected("EXDB033", i1) && selected("EXDB034[i1]", "1")) {
        add("XEHelperNum", value("XEHelperNum")+1)
        add("XEHelper["+value("XEHelperNum")+"]", value("XEChildAliveName[i1]")+"本人")
    }
    if (selected("EXDB033", i1) &&  selected("EXDB034[i1]", "2")) {
        add("XEHelperNum", value("XEHelperNum")+1)
        add("XEHelper["+value("XEHelperNum")+"]", value("XEChildAliveName[i1]")+"配偶")
    }
    if (selected("EXDB033", i1) &&  selected("EXDB034[i1]", "3")) {
        add("XEHelperNum", value("XEHelperNum")+1)
        add("XEHelper["+value("XEHelperNum")+"]", value("XEChildAliveName[i1]")+"的孩子")
    }
}
for (var i1 = 26; i1 <= 35; i1++) {
    if (selected("EXDB033", i1) && selected("EXDB034[i1]", "1")) {
        add("XEHelperNum", value("XEHelperNum")+1)
        add("XEHelper["+value("XEHelperNum")+"]", value("EXDB033_1[i1]")+"本人")
    }
    if (selected("EXDB033", i1) &&  selected("EXDB034[i1]", "2")) {
        add("XEHelperNum", value("XEHelperNum")+1)
        add("XEHelper["+value("XEHelperNum")+"]", value("EXDB033_1[i1]")+"配偶")
    }
    if (selected("EXDB033", i1) &&  selected("EXDB034[i1]", "3")) {
        add("XEHelperNum", value("XEHelperNum")+1)
        add("XEHelper["+value("XEHelperNum")+"]", value("EXDB033_1[i1]")+"的孩子")
    }
}
for (var i1 = 1; i1 < 31; i1++) {
    if (selected("EXDB035", i1) &&  selected("EXDB036[i1]", "1")) {
        add("XEHelperNum", value("XEHelperNum")+1)
        add("XEHelper["+value("XEHelperNum")+"]", value("XESibName[i1]")+"本人")
    }
    if (selected("EXDB035", i1) &&  selected("EXDB036[i1]", "2")) {
        add("XEHelperNum", value("XEHelperNum")+1)
        add("XEHelper["+value("XEHelperNum")+"]", value("XESibName[i1]")+"配偶")
    }
    if (selected("EXDB035", i1) &&  selected("EXDB036[i1]", "3")) {
        add("XEHelperNum", value("XEHelperNum")+1)
        add("XEHelper["+value("XEHelperNum")+"]", value("XESibName[i1]")+"的孩子")
    }
}
for (var i1 = 31; i1 <= 40; i1++) {
    if (selected("EXDB035", i1) &&  selected("EXDB036[i1]", "1")) {
        add("XEHelperNum", value("XEHelperNum")+1)
        add("XEHelper["+value("XEHelperNum")+"]", value("EXDB035_1[i1]")+"本人")
```

```
    }
    if (selected("EXDB035", i1) && selected("EXDB036[i1]", "2")) {
        add("XEHelperNum", value("XEHelperNum")+1)
        add("XEHelper["+value("XEHelperNum")+"]", value("EXDB035_1[i1]")+"配偶")
    }
    if (selected("EXDB035", i1) && selected("EXDB036[i1]", "3")) {
        add("XEHelperNum", value("XEHelperNum")+1)
        add("XEHelper["+value("XEHelperNum")+"]", value("EXDB035_1[i1]")+"的孩子")
    }
}
```

XEHelper 帮助者的身份/名字

```
see above
```

XEHelpList 选出来的主要帮助者

```
for (var i1 = 1; i1 < value("XEHelperNum")+1; i1++) {
    if ( greater("XEHelperNum", "7") && !equal("XEHelperNum", "7") && selected("EXDB039", i1) ) {
        add("XEHelpList[i1]", value("XEHelper[i1]"))
    }
}
for (var i1 = 1; i1 < value("XEHelperNum")+1; i1++) {
    if ( !greater("XEHelperNum", "7") ) {
        add("XEHelpList[i1]", value("XEHelper[i1]"))
    }
}
```

XESelectNum XSelectNum 判断选出来的帮助者数量是否多于 7 个

```
add("XESelectNum", "0")
for (var i1 = 1; i1 < 99; i1++) {
    if (selected("EXDB039", i1)) {
        add("XESelectNum", value("XESelectNum")+1)
    }
}
```

XEChildAndS 如果子女姓名不为空，则显示”XXX 的配偶”

```
if (empty("ZChildName[i]")) {
    add("XEChildAndS[i]", "")
} else {
    add("XEChildAndS[i]", value("ZChildName[i]")+"的配偶")
}
```

XEChildGenderDis 题干中显示加载的性别

```
if (equal("ZChildGender[i]", "1")) {
    add("XEChildGenderDis[i]", "男性")
} else if (equal("ZChildGender[i]", "2")) {
    add("XEChildGenderDis[i]", "女性")
} else {
    add("XEChildGenderDis[i]", "缺失")
}
```

XEChildAlive 访问时子女是否健在

```
if (equal("EXC001[i]", "1")) {
    add("XEChildAlive[i]", "1")
} else if (equal("EXC001[i]", "2")) {
     add("XEChildAlive[i]", "0")
}
```

XEChildAliveName 访问时健在的子女姓名

```
if (equal("XEChildAlive[i]", "1")) {
    add("XEChildAliveName[i]", value("ZChildName[i]"))
}
```

XEChildBirth 子女出生年份

```
if (!empty("ZChildBirth[i]")) {
    add("XEChildBirth[i]", value("ZChildBirth[i]"))
} else {
    add("XEChildBirth[i]", value("EXC003[i]"))
}
```

XEChildGender 子女性别

```
if (!empty("ZChildGender[i]")) {
    add("XEChildGender[i]", value("ZChildGender[i]"))
} else {
    add("XEChildGender[i]", value("EXC004[i]"))
}
```

XEChildEdu 子女最高教育程度

```
if (!empty("ZChildEdu[i]")) {
    add("XEChildEdu[i]", value("ZChildEdu[i]"))
} else {
    add("XEChildEdu[i]", value("EXC005[i]"))
}
```

XEXMIns 去世时候参加的医疗保险

```
add("XEXMIns[1]", "城镇职工基本医疗保险（医保）")
add("XEXMIns[2]", "城乡居民基本医疗保险（合并城镇居民和新型农村合作医疗保险）")
add("XEXMIns[3]", "城镇居民基本医疗保险")
add("XEXMIns[4]", "新型农村合作医疗保险（合作医疗）")
add("XEXMIns[5]", "公费医疗")
add("XEXMIns[6]", "医疗救助")
add("XEXMIns[7]", "商业医疗保险：单位购买")
add("XEXMIns[8]", "商业医疗保险：个人购买")
add("XEXMIns[9]", "城镇无业居民大病医疗保险")
add("XEXMIns[10]", "长期护理保险")
add("XEXMIns[12]", "没有保险")
if (selected("EXEA001", "11")){
    add("XEXMIns[11]", value("EXEA001_1"))
}
```

XEXHaveMIns 去世时候是否有参加的医疗保险

```
if (selected("EXEA001", "1") || selected("EXEA001", "2") || selected("EXEA001", "3") ||
↪   selected("EXEA001", "4") || selected("EXEA001", "5") || selected("EXEA001", "6") ||
↪   selected("EXEA001", "7") || selected("EXEA001", "8") || selected("EXEA001", "9") ||
↪   selected("EXEA001", "10")|| selected("EXEA001", "11")) {
    add("XEXHaveMIns", "1")
}
if (selected("EXEA001", "12")) {
    add("XEXHaveMIns", "0")
}
```

XEXMInsPrivate 去世时是否仅仅有商业医疗保险

```
if (!empty("EXEA001")){
add("XEXMInsPrivate", "0")
}
if ((!selected("EXEA001", "1")) && (!selected("EXEA001", "2")) && (!selected("EXEA001", "3")) &&
↪   (!selected("EXEA001", "4")) && (!selected("EXEA001", "5")) && (!selected("EXEA001", "6")) &&
↪   (!selected("EXEA001", "9")) && (!selected("EXEA001", "10")) && (!selected("EXEA001", "11")) &&
↪   (selected("EXEA001", "7") || selected("EXEA001", "8")))  {
    add("XEXMInsPrivate", "1")
}
```

XEXPMIns 以前参加的医疗保险

```
add("XEXPMIns[1]", "城镇职工基本医疗保险（医保）")
add("XEXPMIns[2]", "城乡居民基本医疗保险（合并城镇居民和新型农村合作医疗保险）")
add("XEXPMIns[3]", "城镇居民基本医疗保险")
add("XEXPMIns[4]", "新型农村合作医疗保险（合作医疗）")
add("XEXPMIns[5]", "公费医疗")
add("XEXPMIns[6]", "医疗救助")
add("XEXPMIns[7]", "商业医疗保险：单位购买")
add("XEXPMIns[8]", "商业医疗保险：个人购买")
```

```
add("XEXPMIns[9]", "城镇无业居民大病医疗保险")
add("XEXPMIns[10]", "长期护理保险")
add("XEXPMIns[12]", "没有保险")
if (selected("EXEB001", "11")){
    add("XEXPMIns[11]", value("EXEB001_1"))
}
```

XEXQuarantined 是否曾被隔离

```
if (selected("EXV004", "1") || selected("EXV004", "2") || selected("EXV004", "3") || selected("EXV004",
↪  "4")|| selected("EXV004", "5")) {
    add("XEXQuarantined", "1")
} else {
    add("XEXQuarantined", "0")
}
```

XEXVAHospitalizationExcluded 措辞：隔离排除住院时间

```
if (greater("EXV003", "0")) {
  add("XEXVAHospitalizationExcluded", "除去那几天因为新冠的住院时间,")
}
```

XVCNotInQuarantine 措辞：疫情期间排除隔离

```
if (equal("XEXQuarantined", "1")) {
    add("XEXVCNotInQuarantine", ", 不包括强制性隔离")
}
```

XKChildName 生成选项为 2-26 序号的子女列表

```
for (var k = 1; k <= 25; k++) {
        add("XKChildName["+(k+1)+"]", value("ZChildName[k]"))
}
```

XKChildAndS 生成选项为 27-51 序号的子女”XXX 的配偶” 列表

```
for (var j = 1; j <= 25; j++) {
        add("XKChildAndS["+(j+26)+"]", value("XEChildAndS[j]"))
}
```

XEXGChildList1 根据 EXG003 的回答，生成 1 到 35 的子女名字列表

```
for (var i1 = 1; i1 <=25 ; i1++) {
    add("XEXGChildList1[i1]", value("ZChildName[i1]"))
}
for (var i1 = 26; i1 <=35 ; i1++) {
    add("XEXGChildList1[i1]", value("EXG003_1[i1]"))
}
```

XEXGChildList4 根据 EXG007 的回答，生成 1 到 35 的子女名字列表

```
for (var i1 = 1; i1 <=25 ; i1++) {
    add("XEXGChildList4[i1]", value("ZChildName[i1]"))
}
for (var i1 = 26; i1 <=35 ; i1++) {
    add("XEXGChildList4[i1]", value("EXG007_1[i1]"))
}
```

XEXGChildList2 根据 EXG022 的回答，生成 1 到 35 的子女名字列表

```
for (var i1 = 1; i1 <=25 ; i1++) {
    add("XEXGChildList2[i1]", value("ZChildName[i1]"))
}
for (var i1 = 26; i1 <=35 ; i1++) {
    add("XEXGChildList2[i1]", value("EXG022_1[i1]"))
}
```

XEXGChildList5 根据 EXG026 的回答，生成 1 到 35 的子女名字列表

```
for (var i1 = 1; i1 <=25 ; i1++) {
    add("XEXGChildList5[i1]", value("ZChildName[i1]"))
}
```

```
for (var i1 = 26; i1 <=35 ; i1++) {
    add("XEXGChildList5[i1]", value("EXG026_1[i1]"))
}
```

XEXGChildList3 根据 EXG032 的回答，生成 1 到 35 的子女名字列表

```
for (var i1 = 1; i1 <=25 ; i1++) {
    add("XEXGChildList3[i1]", value("ZChildName[i1]"))
}
for (var i1 = 26; i1 <=35 ; i1++) {
    add("XEXGChildList3[i1]", value("EXG032_1[i1]"))
}
```

XEXGChildList6 根据 EXG036 的回答，生成 1 到 35 的子女名字列表

```
for (var i1 = 1; i1 <=25 ; i1++) {
    add("XEXGChildList6[i1]", value("ZChildName[i1]"))
}
for (var i1 = 26; i1 <=35 ; i1++) {
    add("XEXGChildList6[i1]", value("EXG036_1[i1]"))
}
```

XEXGTotalValue 确认除房产外遗产总数额。如果某项遗产为空或者-1，那么替代为 0。

```
add("XCK_EXF005", value("EXF005"))
if (empty("XCK_EXF005") || equal("XCK_EXF005", "-1")) {
  add("XCK_EXF005", "0")
}

add("XCK_EXF007", value("EXF007"))
if (empty("XCK_EXF007") || equal("XCK_EXF007", "-1")) {
  add("XCK_EXF007", "0")
}

add("XCK_EXF009", value("EXF009"))
if (empty("XCK_EXF009") || equal("XCK_EXF009", "-1")) {
  add("XCK_EXF009", "0")
}

add("XCK_EXF011", value("EXF011"))
if (empty("XCK_EXF011") || equal("XCK_EXF011", "-1")) {
  add("XCK_EXF011", "0")
}

add("XCK_EXF013", value("EXF013"))
if (empty("XCK_EXF013") || equal("XCK_EXF013", "-1")) {
  add("XCK_EXF013", "0")
}

add("XCK_EXEB006", value("EXEB006"))
if (empty("XCK_EXEB006") || equal("XCK_EXEB006", "-1")) {
  add("XCK_EXEB006", "0")
}

add("XCK_EXG010", value("EXG010"))
if (empty("XCK_EXG010") || equal("XCK_EXG010", "-1")) {
  add("XCK_EXG010", "0")
}

add("XCK_EXG012", value("EXG012"))
if (empty("XCK_EXG012") || equal("XCK_EG012", "-1")) {
  add("XCK_EXG012", "0")
}

add("XCK_EXG014", value("EXG014"))
if (empty("XCK_EXG014") || equal("XCK_EXG014", "-1")) {
  add("XCK_EXG014", "0")
}

add("XCK_EXG016", value("EXG016"))
if (empty("XCK_EXG016") || equal("XCK_EXG016", "-1")) {
  add("XCK_EXG016", "0")
}
```

```
add("XCK_EXG017", value("EXG017"))
if (empty("XCK_EXG017") || equal("XCK_EXG017", "-1")) {
  add("XCK_EXG017", "0")
}

add("XEXGTotalValue", value("XCK_EXF005")+value("XCK_EXF007")+value("XCK_EXF009")+value("XCK_EXF011")+va
↪  lue("XCK_EXF013")+value("XCK_EXEB006")+value("XCK_EXG010")+value("XCK_EXG012")+value("XCK_EXG014")+v
↪  alue("XCK_EXG016")+value("XCK_EXG017"))
```

附录　函数说明

A. 题干中的函数

Hard Check 硬检查函数 hc(a,b,c) 用于规避数值型变量在逻辑上不可能的记录，如果对应问题的记录不满足 hc 中的条件，则要求访问员返回修改答案。

参数说明：

- a：取值区间
- b: *int* 表示只能填写整数，*real* 表示可以填写小数
- c：受访者拒绝回答或不知道对应问题时的记号，∅ 表示不允许这类记录

Soft Check 软检查函数 sc(a,b,c) 用于提醒数值型变量的异常值，如果对应问题的记录不满足 sc 中的条件，则要求访问员再次确认。

sc(a,b,c) 的参数与硬检查函数完全一致。

Unfolding Brackets 分级展开函数 ub(a,b) 用于在受访者拒绝回答或不太清楚某个具体金额的问题（比如收入）时获取该金额的范围（range）信息。

参数说明：

- a：数组，表示分级展开的触发条件，即只要对应问题的记录在该数组内，则触发分级展开
- b: 数组，表示分级展开的断点（breakpoints）

分级展开的问题中的拒绝回答或不知道的标记是“-1”。

Conflict 冲突检查函数 conflict(a,b,...,c) 用于检查输入答案是否存在逻辑错误，如何最后一个选项 c 与之前的任何一个选项被同时选中，则提醒错误，访问员必须重新确认并更新答案。

Transfer Picture 图片展示函数 transferPic() 用于展示图片。

B. 辅助变量中的函数

问卷中的辅助变量都是由原始变量基于预设的规则计算出来的，这些辅助变量的构建涉及到一些基础的逻辑控制结构（control structure），比如 if-else-if 和 for 循环，同时还包含一些自定义的函数。当前问卷涉及如下的自定义函数：

- add(x,y)：将变量 x 的值设为 y
- value(x)：返回变量 x 的值
- selected(x,y)：判断 y 是否在数组 x 中，一般用于判断某个选项是否在对应的多选题中被选中，选中返回 true，否则返回 false
- count(x)：返回数组 x 的大小（即元素个数），一般用于判断某个多选题选中的选项个数
- range(x,min,max,decimal,special)：判断变量 x 是否满足设定的范围，min 表示范围下限，max 表示范围上限，decimal 表示是否允许小数（true 表示允许小数，false 表示不允许小数），special 表示范围之外特别允许的数值（缺失表示没有）
- greater(x,y,equal)：判断变量 x 是否大于或大于等于 y，equal 用于控制比较的是大于还是大于等于（true 表示比较的是大于等于，缺失表示比较的是大于）
- equal(x,y)：判断变量 x 是否等于 y，等于返回 true
- empty(x)：判断变量 x 是否为空或不存在，为空或不存在返回 true
