# Supplementary material for: Association of internet use and health service utilization with self-rated health in middle-aged and older adults: findings from a nationally representative longitudinal survey
Source: Front Public Health. 2024 Oct 3;12:1429983. doi: 10.3389/fpubh.2024.1429983 (PMC11483889; doi:10.3389/fpubh.2024.1429983)
Supplement: Supplementary file 8 [file Data_Sheet_8.PDF]

中国健康与养老追踪调查

第五轮（2020）用户手册

赵耀辉　陈欣欣　王亚峰　孟琴琴
薄海　陈川　陈燕凤　龚金泉　贾媛　王格玮
吴晓阳　颜力　杨鹏　周红艳

版本号: 20231106

2023 年 11 月

引用格式: 赵耀辉，陈欣欣，王亚峰，孟琴琴，薄海，陈川，陈燕凤，龚金泉，贾媛，王格玮，吴晓阳，颜力，杨鹏，周红艳（2023），中国健康与养老追踪调查第五轮（2020）用户手册，北京大学国家发展研究院

北京大学国家发展研究院
北京大学中国社会科学调查中心

This page intentionally left blank

如何引用 CHARLS 数据

当你使用 CHARLS 数据开展研究时，在介绍数据的部分，请引用对 CHARLS 数据进行介绍的权威论文，这样可以佐证数据的权威性。

1. 如何引用本用户手册？
    - 赵耀辉，陈欣欣，王亚峰，孟琴琴，薄海，陈川，陈燕凤，龚金泉，贾媛，王格玮，吴晓阳，颜力，杨鹏，周红艳（2023），中国健康与养老追踪调查第五轮（2020）用户手册，北京大学国家发展研究院。
2. 使用 CHARLS 数据开展研究，一般性地介绍数据请引用以下文章
    - Zhao, Yaohui, Yisong Hu, James P Smith, John Strauss, Gonghuan Yang. (2014). Cohort Profile: The China Health and Retirement Longitudinal Study (CHARLS), *International Journal of Epidemiology*, 43 (1): 61–68.
    - 使用 CHARLS 基线数据以及介绍抽样信息，请引用以下文章
    - Zhao, Yaohui, John Strauss, Gonghuan Yang, John Giles, Peifeng (Perry) Hu, Yisong Hu, Xiaoyan Lei, Man Liu, Albert Park, James P. Smith, Yafeng Wang. (2013). China Health and Retirement Longitudinal Study: 2011-2012 National Baseline User's Guide, *National School of Development, Peking University*.
3. 使用 CHARLS 的生物样本数据，请在描述数据时引用以下论文：
    - Chen, Xinxin, Eileen Crimmins, Perry Hu, Jung Ki Kim, Qinqin Meng, John Strauss, Yafeng Wang, Yuan Zhang, Yaohui Zhao, 2019, Venous Blood-based Biomarkers in the China Health and Retirement Longitudinal Study: Rationale, Design, and Results of the 2015 Wave, *American Journal of Epidemiology*, 188(11): 1871–1877

前言

本手册介绍了中国健康与养老追踪调查项目（CHARLS）2020 年开展的第五轮全国调查的总体流程，包括设计、实施和数据发布，旨在帮助数据用户更好地理解和使用这套数据。

CHARLS 是一项具备中国大陆 45 岁及以上人群代表性的追踪调查，旨在建设一个高质量的公共微观数据库，采集的信息涵盖社会经济状况和健康状况等多维度的信息，以满足老龄科学研究的需要。

为利用国际上最佳的数据采集方式，并确保研究结果的国际可比性，CHARLS 参照包括美国的健康与退休研究（HRS）在内的系列国际老龄调查研究开展调查设计。其全国基线调查于 2011-12 年进行，于 2013 年、2015 年、2018 年和 2020 年分别开展了 4 轮常规问卷的追踪调查，并于 2014 年完成了中国中老年人生命历程调查。为确保样本的代表性，CHARLS 基线调查覆盖了全国 150 个国家/地区、450 个村庄/城市社区，涉及 10,257 户家庭的 17,708 人，反映了中国中老年人群的总体情况。2019 年底到 2020 年初，新冠疫情在中国爆发，为及时记录新冠疫情对中国中老年人生活和健康的影响，在 2020 年的第 5 轮调查中增加采集了疫情相关的信息。

CHARLS 项目所采集的所有数据均保存在北京大学中国社会科学调查中心。CHARLS 项目前四轮调查数据和中国中老年人生命历程调查数据已全部在 CHARLS 网站（https://charls.pku.edu.cn）上公开发布。截至 2023 年 10 月底，有 8.8 万余名用户注册并下载了数据。基于 CHARLS 的出版物增长迅速，据不完全统计，截止 2023 年 9 月底，以 CHARLS 数据为基础发表的论文有 4,587 篇，其中英文期刊论文有 2,079 篇，中文期刊论文 1,639 篇，学位论文有 697 篇。

致谢

中国健康与养老追踪调查（CHARLS）是一个庞大的科研项目，倾注了很多人的汗水。在此，我们向 CHARLS 研究团队、实地调查的组织和参与人员，每一位受访者和所有为访问提供支持的人员表示感谢。

CHARLS 第五轮调查的设计由赵耀辉教授总体负责，问卷和数据工作负责人是王亚峰博士，实地执行工作由陈欣欣博士负责。执行部成员包括贾媛、王永洁、胡倩煜和马钰在内的实地督导团队，她们招募、培训和管理了一支由 549 名实地访谈员、130 名质量核查人员和 15 名督导组成的团队。数据部团队成员包括孟琴琴、薄海、陈燕凤、龚金泉、王格玮、吴晓阳、颜力、杨鹏和周红艳负责设计问卷、利用并行数据开展实时的数据质量核查、清理数据并构建抽样权重。陈川和赵银霞负责为实地访问提供信息技术支持，他们开发了应用于安卓系统的 CAPI 系统，并为访员招聘、培训和实地访问期间的管理和多手段的质控核查开发了相应的支持系统。宋娜负责实地执行期间的所有后勤问题，金海玉负责 CHARLS 的财务事宜。王文津博士参与了问卷翻译工作。十多名学生参与了调查后的数据检查、清理、电话回访、开放式问题编码以及数据公开发布的准备工作。

CHARLS 调查的顺利开展，离不开所有受访户的理解和支持。他们所提供的数据不仅为中国老龄化问题的学术研究奠定了基础，也为我国政府未来社会保障政策的制定提供了参考。在此，我们表示衷心的感谢！

目录

如何引用 CHARLS 数据 **i**

前言 **iii**

致谢 **v**

1 CHARLS 基本情况介绍 **1**

1.1 研究背景和意义 1

1.2 伦理审批 2

1.3 本手册的结构 2

2 访问样本 **3**

2.1 基线抽样 3

2.2 备访样本 3

2.3 代理访问样本 4

3 问卷内容 **5**

4 实地访问和应答情况 **7**

4.1 问卷设计 7

4.2 CAPI 系统开发 7

4.3 访问员的招募与培训 7

4.4 实地访问的组织 8

4.5 实地访问使用的语言 8

4.6 访问质量控制 9

4.7 样本追踪 9

5 样本权重 **11**

6 数据集信息 **13**

7 表格附录 **15**

参考文献 **19**

1 CHARLS 基本情况介绍

1.1 研究背景和意义

中国是世界上老龄人口最多的国家，也是当今世界上老龄化速度最快的国家之一。到 2021 年底，中国 65 岁及以上老年人口占总人口的 14.2%，这意味着该比例从 7% 增至 14%，仅用时 21 年。相比之下，在多数发达国家，大概耗时大约半个世纪 (Kinsella, He, and Way, 2009)。预计到 2065 年，中国将成为世界上人口最多的 20 个国家中老龄化最严重的国家。到 2050 年，中国 65 岁及以上人口将达到 3.95 亿，相当于美国现有人口的 1.2 倍。高龄老人（80 岁及以上）的数量将达到 1.35 亿，超过日本目前的人口数量 (United Nations, 2022)。

与老龄化进程相关的是，中国正在经历快速的健康转型，从主要影响年轻人的传染病转变为影响老年人的慢性病 (Yang 等, 2013)。此外，与工业化国家相比，中国在经历老龄化过程时的收入水平要低得多。与其他大多数开展健康与退休研究的国家相比，中国的农村人口更多，老年人的受教育水平较低，可获得的公共服务水平较低，同时随着城市化的推进，传统的家庭养老面临更加严峻的挑战。如何解决老年人的赡养问题，是快速发展的中国社会在未来几十年面临的最重大挑战之一。

为了应对这一挑战，中国政府采取了强有力的措施来解决这一问题。近几十年来，中国实施了一系列社会保障政策。其中包括最低生活保障制度、新型农村合作医疗保险、城乡居民医疗保险、新型农村社会养老保险、城镇居民社会养老保险等。虽然其中一些政策并非专门针对老年人，但老年人口无疑是最重要的受益群体之一。与许多其他政策类似，这些政策由中央政府发起，但地方政府在执行过程中保持一定的自主权。地方政府可以决定试点和推广的时间表，也可以制定不同的实施计划。CHARLS 正试图从家庭和社区两个层面反映这些社会保障体系的实施状况，并希望通过进一步的分析，为政府进一步修订和完善现有政策提供更科学的依据。

在 2011-12 年 CHARLS 基线调查之前，对中国老龄相关问题的科学研究还处于起步阶段，最大的障碍是缺乏足够的微观追踪调查数据。当时已有的相关数据库往往样本规模较小，或者缺乏对老年健康开展社会科学研究所需的多维度的信息。例如，已有的与健康相关的数据库多关注健康指标的采集，而忽视社会经济指标；同时，社科学者建设的调查数据库往往缺乏与健康相关的指标。由于老年人的福利与他们的健康和社会经济状况密切相关，而且健康和社会经济水平本身也是相互关联的，因此中国老龄问题的研究亟需兼具广度和精度的微观数据。CHARLS 为填补这一空白做出了尝试。

CHARLS 是中国首个具有全国代表性的中老年人口调查，它以美国健康与退休研究（HRS）为蓝本，吸收和参考全球相关老龄调查的设计，比如英国老龄追踪调查（ELSA）、欧洲健康、老龄和退休调查（SHARE）、日本老龄和退休研究（JSTAR）、印度老龄追踪

调查（LASI）、印度尼西亚家庭生活调查（IFLS）和韩国老龄追踪调查（KLoSA）等，对中国老年人口健康状况进行研究。更为重要的是，该研究密切追踪中国近年来陆续推行的相关社保政策的实施情况和重大公共卫生事件（比如新冠疫情）对中国中老年人健康的影响。

1.2 伦理审批

CHARLS 各轮调查均获得了北京大学生物医学伦理委员会的批准。本轮家户问卷调查的实地工作方案已获得批准，批准号为：IRB00001052-11015。数据用户如在发表论文时需要该批件，请发邮件联系项目组：charls_info@pku.edu.cn，并提供具体的需求信息。

在实地调查期间，每位同意参与调查的受访者都被要求签署两份知情同意书。一份由受访者本人保存，另一份存放在 CHARLS 办公室，并以 PDF 格式扫描保存。

1.3 本手册的结构

第 2 部分介绍了 CHARLS 的抽样设计，主要是基线抽样程序、备选样本和代理访问。第 3 部分介绍了调查内容，包括第 5 轮调查内容的简要说明。第 4 部分介绍了实地调查的组织和受访者的追踪。第 5 部分介绍样本权重的构建，第 6 部分描述数据集的一些基本信息。

2 访问样本

CHARLS 全国基线调查于 2011-2012 年在全国 28 个省、150 个国家/地区、450 个村庄/城市社区开展，2013 年、2015 年、2018 年和 2020 年分别开展了第 2 轮调查，第 3 轮调查、第 4 轮调查和第 5 轮调查。CHARLS 基线调查对象为 45 岁及以上的住户人群，未涵盖入住养老机构的中老年人，但在之后几轮的追踪调查中对入住养老机构的基线调查对象进行了调查。受疫情防控政策的影响，在 2020 年第 5 轮调查中，新疆 3 个样本村居和内蒙古部分样本村居的访问未能开展。本节将介绍 CHARLS 样本的产生过程。

2.1 基线抽样

CHARLS 基线调查采用基于内隐分层（分层指标为区域、城乡属性以及人均 GDP）的多阶段（县/区-村/居-户）PPS 随机抽样方式。第一阶段，除了西藏以外，全国其他所有的县区在八个大区内分别按照城乡属性和人均 GDP 进行排序，再以与人口规模成比例的概率选取 150 个县或区 (Zhao 等, 2013)。在第二个阶段，在每个抽中的县级单位内，以与人口规模成比例的概率随机选取 3 个二级抽样单元（村委会或居委会）(Zhao 等, 2013)。因此，CHARLS 具有全国以及大区代表性。经上述抽样过程后，CHARLS 的基线样本分布于 28 个省、150 个区县的 450 个村居中。

考虑到村/居一级的住户列表因人口流动无法及时更新，CHARLS 开发了一个绘图/列表软件（Charls-GIS），利用谷歌地球的地图图像列出所有住宅楼的所有居住单元，以创建样本框。在每个抽中的家户中，访员使用一份简短的筛查表来确定该家户是否有符合年龄要求的成员。如果住户中有 45 岁以上且符合居住标准的人员，则随机抽取其中一人。如果被选中的人年龄在 45 岁或以上，那么他/她将成为主要受访者，其配偶也将接受访问。因此，CHARLS 的基线调查对象为每个家户中一名年满 45 岁的老人及其配偶，当年共计访问 17,708 人，他们居住在 450 个村庄/城市社区的 10,257 个家户中（表 1）。

在使用抽样权重加权后，CHARLS 基线样本的人口统计学特征与 2010 年人口普查的特征非常相似 (Zhao 等, 2014)，展示出对中国中老年人群良好的代表性。

2.2 备访样本

CHARLS 是一项针对 45 岁及以上人群的追踪研究。随着研究的持续，如果不补充新的样本，受访者的年龄会越来越大，将导致较年轻群体不再具备代表性。比如，在基线调查中，年龄在 45-46 岁之间的受访者在 2 年后的下一轮调查中将变为 47-48 岁, 这就使得 2013 年 45-46 岁的群体缺乏代表性。为了持续保持对 45 岁及以上人群的代表性，CHARLS 在基线调查中预留了 40-44 岁的人作为备访样本，供以后的调查使用。即，在基线调查时，如果随机抽中的家户中有 40 岁以上的人，我们会随机抽取其中一人。当他/她在后续的追踪调查中年满 45 岁，那么他/她将成为主要受访者，并对其配偶进行访

问。那就意味着：在 2013 年的第 2 轮追踪调查中，除了继续追踪访问基线调查中已抽中的 45 岁以上人群及其配偶外，还新增访问基线调查中年龄为 43-44 岁的受访者及其配偶，2015 年和 2018 年的追踪调查亦是如此。随着备访样本的耗竭，2018 年之后的追踪调查，CHARLS 样本对较年轻的群体不再具备全国代表性。

随着备访样本的加入，以及在后续每轮追踪调查中都会继续寻找并访问基线调查中未能联系上的家户，受访者总人数（主要受访者加上配偶）从基线调查的 17,708 人增加到第 5 轮调查的 19,395 人（表 1）。

表 1 还描述了基线调查和第五轮调查期间 CHARLS 样本的年龄/性别构成情况。在基线调查中，我们共采集了 17,708 位受访者的信息，其中 52.1% 为女性，60 岁以下的受访者有 58%，而在第 5 轮调查中，由于没有补充更年轻的样本，60 岁以下的受访者仅占 38%。

2.3 代理访问样本

在一些特殊情况下，例如当受访者因聋哑、精神失常、老年痴呆或者身体虚弱等健康原因无法完成调查，或者住在境外等无法追访的地方时，访员通常通过采用代理模式完成问卷访问。有的受访者本人强烈拒绝参加访问，但其家人愿意代为完成访谈，他们中的一小部分人也通过代理方式完成了访问。在进行代理访问之前，访谈员需要确定一名熟悉受访者并能提供其准确信息的代理人。在大多数情况下，代理人多由亲属（如配偶或子女）担任。

为了尽可能限制代理访问的使用，项目组要求采用代理模式访问的访员向队长汇报采用代理模式的原因，并申请代理码, 队长将在核实后向访员发放代理授权码。在 2011-2018 年的四轮调查中，当访员在平板中输入该代理码后，从第一个模块开始，系统就自动切换到代理模式，即每个模块的问卷题目都默认为是在代理模式下询问的题目。但在 2020 年的第五轮调查中，考虑到受访者有可能有能力回答部分模块的题目，因此，CAPI 系统做出了如下修改：当访问员在访问平板中输入该代理码后，在进入每一个访问模块时，访问系统都会首先确认该模块是否采用代理模式，如果选择不代理，那么受访者在该模块所被询问的题目和常规问卷完全相同；如果选择代理，那么代理人会被询问常规问卷中的部分题目（详见每个模块的逻辑图）。在第 5 轮调查中，共有 1,837 个样本申请了代理码。

3 问卷内容

在第 5 轮调查中，每个追踪家户都将首先完成（CV）过滤问卷。基于该模块采集的信息，对于在上次访问后已经去世的受访者，我们将邀请熟悉该受访者情况的人完成（EX）退出问卷；对于其他在世的受访者，将完成常规的家户问卷。

常规的家户问卷的核心内容主要由以下几个模块组成：(B) 基本信息，(C) 家庭信息，(D) 健康状况与功能，(F) 工作与退休，(G) 收入和支出（包含了住房情况），以及（V）疫情相关信息。表 2 概述了第五轮调查家户问卷的主要问卷内容。

除了丰富的个人社会、经济和行为数据外，CHARLS 数据的特点还包括丰富的受访者健康信息。健康部分从自我报告开始，包括受访者对一般健康状况的自我评估、询问医生对一系列慢性疾病诊断的问题、日常生活活动（ADLs）、工具性日常生活活动（IADLs）和身体功能障碍的问题。随后是有关抑郁症状和认知能力的部分。此外，还收集了几种健康行为的信息。其中包括有关吸烟、饮酒和体育活动的详细信息。

CHARLS 在第 2-5 轮的四次跟踪调查中，在 CAPI 中设计问卷时使用了预加载变量。从 2020 年的第 5 轮调查开始，我们将所有问题转换为逻辑图（该逻辑图和问卷一起发布），以展示每个问题的所有可能答案的路径模式，从而减少了问卷编程的错误。

为了了解新冠疫情对中国中老年人健康的影响，在第 5 轮调查中我们新增了一个专门关于新冠疫情的模块，以收集受访者在疫情爆发期间的预防行为、疾病预防意识、个人感染经历和隔离经历、疫情期间的个人活动和当地的防控举措等。此外，在健康模块、收入模块和工作模块中还分别收集了疫情爆发期间医疗服务利用、收入和就业可能受到的影响。

需要指出的是：由于新增的疫情模块增加了访问负担，和之前几轮调查相比，第 5 轮调查中其他部分模块的信息采集做了一些简化，比如家庭信息模块中未采集兄弟姐妹的相关信息，同时，和之前相比，健康状况、医疗服务利用状况、医疗保险、养老金以及资产的信息相对较少。

This page intentionally left blank

4　实地访问和应答情况

本轮调查的顺利开展，有赖于一系列的准备工作，包括：问卷设计、CAPI 系统的开发、访员的招聘和培训、实地访问的组织管理和质量控制。每轮调查的实地准备工作通常需要一年时间。CHARLS 研究团队于 2019 年 8 月开始修订问卷，并组织了几次预调查，以测试和改进问卷及调查流程。2020 年 4 月，在使用 CAPI 最终确定问卷之前，我们进行了几次正式的预调查。根据预调查的结果，研究团队修改了实地访问的问卷和流程。2020 年 3 月，项目组开始招募访问员，并准备培训材料。2020 年 4 月至 7 月，673 名大学生参加了约 60 个学时的培训课程，最终有 549 名学生通过了培训考试，参加实地访问。大部分实地访问工作于 2020 年 9 月底前完成。本节将介绍与实地访问相关的工作，包括样本追踪策略。

4.1　问卷设计

由于本轮调查是一项追踪调查，问卷设计中涉及到前几轮调查中已采集的受访者信息的预加载，以及在两轮调查之间的受访者状态变化的询问，要求比较复杂，因此研究团队花费大量精力绘制了问卷中每个模块的电子流程图，以理清每个问题间的逻辑关系，以便优化问卷设计流程。在此过程中，纠正了以前的 CAPI 错误和前几轮问卷设计中存在的逻辑错误。

此外，在常规调查内容之外，我们还增加了一个专门的疫情模块，以收集受访者在新冠疫情爆发初期的感染、隔离和个人活动经历。考虑到该模块的内容是全新的，在最终问卷定稿前，我们邀请北京的访员开展了几次预调查。

4.2　CAPI 系统开发

为了适应追踪调查的要求，我们持续开展了大量的程序开发工作，从 2018 年的第 4 轮调查开始，我们将调查终端从 Windows 笔记本电脑换成了安卓平板，这不仅方便收集 GPS、照片和录音等信息，同时，系统的运行速度也更快。经过优化后的 CAPI 系统，不仅能开展基于逻辑图的的问卷设计，自动生成问卷，也能自动开展问卷逻辑测试，此外也为访问的质量控制提供了更多维的数据。

4.3　访问员的招募与培训

受疫情防控政策影响，参与本轮调查的访员的招聘和培训工作主要通过线上方式举行。为了便于在短时间内招聘到 500 多名大学生访员，我们开发了网络招聘系统，通过 CHARLS 微信公众账号等渠道发布招聘广告，应聘的同学在线申请访员岗位后，我们基于应聘者的工作态度、以往的实地访问经验和沟通技巧等诸多因素，筛选出大学生参与访问培训。为了尽可能减少访员的大范围流动，在本轮调查中我们尽可能选择家乡为样本地的大学

生访员。在最后参与实地访问的 549 名访员中，来自京外高校的有 332 位，占 61%。因而当年的访员多数能说当地方言，便于和受访者开展更有效率的沟通。

我们的大部分访员都是本科生和研究生，这一点在历次采访中都是如此。由于严格的新冠疫情防控政策，不鼓励学生在学校间的流动。因此，当年的培训主要以在线培训为主。为了克服在线培训中课堂参与较少的弊端，我们专门增加了 25 人以内的脚本练习课程；并专门开发了访员自学和测试系统，用于检查和改进培训效果；培训过程中还安排了便利样本访问；对于最终确定参加实地访问的同学，我们在样本村居附近安排了 2-3 天左右的实地模拟，由具备丰富访问经验的督导带队观察，帮助访员改进访问规范，提升访谈技能。培训课程历时约 60 个小时，确保学生熟悉 CAPI 系统、掌握问卷内容、培养访谈技巧、了解实地工作规程和各自职责。通过最终培训考核的同学被确定为访员。

4.4 实地访问的组织

在第 5 轮调查中，共有 76 支访问队伍，549 名访员参与访问。一般而言，每支队伍负责访问 2 个样本区县内 6 个村居所有受访者，同时还访问从其他地方迁入该区县及其附近区县的受访者。每支队伍由 6-8 名访员构成，其中有一名队长，负责全队访问任务的管理。同时，有两人担任先遣员的角色，负责更新受访者的联系信息，以便及早发现外出的受访者并重新分配给其他队伍，同时负责安排团队的后勤工作，并完成社区问卷和区县的政策问卷。

在实地访问期间，每支伍都由一名具有实地访问经验的督导负责管理，每位督导管理 2-5 支队伍，负责向 CHARLS 总部的执行部门汇报工作，督导在管理队伍过程中无法解决的问题，由 CHARLS 总部负责解决。

在 2011-2018 的四轮调查中，所有访问都采用面对面的访问形式。然而，在第五轮访问中，有的受访者担心被新冠病毒感染而不愿意接受面访，有的受访者住在不允许探访的养老院，在这些情况下，访员向项目组提出申请，获准后通过视频方式开展访问。在这轮调查中，共有 267 位受访者通过视频方式完成访问。

4.5 实地访问使用的语言

绝大多数中国人（92%）是汉族，使用相同的书面语言。一些人口众多的少数民族也使用汉族语，如满族、回族和壮族。因此，我们估计只有不到 4% 的中国人使用不同的书面语言。尽管有相同的书面语言，但中国不同地区使用不同的方言。

为了减少沟通上的困难，我们在面试时，会优先考虑有方言优势的同学。在第 5 轮调查中，我们有 549 名来自全国各大专院校的学生访谈员。每个省份都有很多的大学生，因此我们在招募调查地的学生时没有遇到任何困难，对于维吾尔族和藏族等少数民族自治区县，我们招募的学生全部会说当地的方言。在极少数情况下，如果访员全都不会说受

访者的方言，或者需要使用普通话以外的少数民族语言，则会邀请其他会说该地方言的村居工作人员或当地居民全程翻译。

4.6 访问质量控制

在第 5 轮调查中，我们继续采用了之前行之有效的质量控制措施，包括对访员开展质量控制培训。为确保数据质量，我们仍采用了常规的质控措施，如数据核查和录音回听 (Zhao 等, 2013)。

计算机辅助访问系统（CAPI）的使用大大方便我们及时识别和纠正访员不规范的访问行为。当访员还在访问村居时，专门开发的质控系统就会立即将不规范的访问行为（如不规范的跳转提问、提问不完整或提问太快）告知访员。

其他的核查措施还包括：比较不同轮次的受访者照片，以确保受访者为同一人；核对录音；与受访者进行简短的电话回访。CAPI 系统允许各队伍在每天结束时通过互联网将数据传回总部，以便进行及时检查。所有访员的第一次访谈都将被核查。对于那些核查得分相对较低的访员，我们会更频繁地检查他们的后续访问。通常情况下，访员会在访问结束后 48 小时内收到质量控制团队的反馈。这样可以确保一些错误在队伍往下一个村居之前得到纠正。

在第 5 轮调查中，质量控制团队的 130 名成员每天都向访员提供详细的反馈。事实证明，这种反馈不仅是对实地调查前培训的有益补充，也是对实地调查监督的有益补充。

4.7 样本追踪

对于在基线调查中接受访问的每一位受访者，我们会在每轮追踪调查中再次访问他们。同时对于基线调查中未能找到的家庭或个人，我们仍将在后续每一轮的调查中继续联系他们。在任何一轮或多轮调查中未能参与访问的受访者，我们均不会放弃，仍继续联系他们开展追踪访问。

当受访者及其配偶离开原家庭时，访员仍将访问他们。基线调查中的主要受访者及其配偶在 CHARLS 的整个生命周期中都会被追踪访问，直到他们去世。如果主要受访者或配偶再婚，只要新配偶在特定轮次时仍与基线受访者处于婚姻存续期间，就会对其进行访问。

CHARLS 的目标是面对面地访问每一位受访者，无论他们搬到哪里。为了实现这一目标，我们借鉴了非常成功的印度尼西亚家庭生活调查（IFLS）的跟踪流程，并进行了创新，将该程序计算机化，以便将搬迁后的样本快速调配给其居住地附近的队伍。从上一轮调查中，我们掌握了每个受访者的联系信息，如当前的固定电话号码和手机号码。如果受访者搬家，我们通过已经采集的其亲戚或朋友的姓名、地址和电话号码等信息，联

系他们，来了解受访者的当前居住地址。当我们在上轮访问地没有找到受访者时，我们就会使用这些辅助信息。

通过这些努力，我们实现了很高的受访者应答率。基线调查的应答率为 80.5%（其中农村地区为 94%，城市地区为 69%，城市地区的应答率较低，这在发展中国家开展的大多数调查中很常见 (Zhao 等, 2013))。表 3 介绍了第 2、3、4 和 5 轮调查的样本数量和应答率。

目前面板样本的应答率在任何一轮追踪调查中都保持在 86% 以上。具体而言，在基线调查中接受过访谈的家庭中，约有 88% 的家庭在 2013 年的第二轮调查中至少完成了一个模块（农村地区为 92%，城市地区为 83%）。在第五轮调查中，约 87% 的受访家庭至少完成了一个模块（表 3）。与许多 HRS 系列的老龄调查相比，CHARLS 展示出了较高的应答率。

5　样本权重

我们建议用户在进行总体推断时使用权重，以最小化由于关键子群体中的应答差异而导致的代表性偏误。基于基线数据的抽样权重 (Zhao 等, 2013)，我们直接从第 5 轮家户和个体的抽样概率构建了横截面样本权重。我们提供了两组家户权重，一组调整了家户层面的无应答（HH_weight_ad1），另一组没有进行调整（HH_weight）。我们提供了两组个体权重，一组调整了家户层面和个体层面的无应答（INDV_weight_ad2），另一组没有进行任何无应答调整（INDV_weight），所有的家户和个体权重均考虑了死亡和离婚的因素。从第三期数据开始，我们不再提供面板权重。这是由于面板权重取决于分析目的，用户可能使用所有期的平衡面板，也可能只使用部分期的面板数据。用户可以根据任何合适的样本损耗调整方法构建面板权重。

横截面权重仅针对代表性样本，包括代理和部分访问。所有其他非代表性个体，如错误的基线受访者（例如，访问员去了错误的地址，在后续追访中得到确认）都没有权重。Sample_Infor 数据集中的变量 crosssection 可以用来识别横截面样本。

6 数据集信息

本次数据发布（版本号：20231106）一共发布了八个主要数据集以及两个辅助数据集（样本信息以及横截面权重）。PSU 信息与往期数据相同。下表给出了这十个数据集的详细说明。

问卷模块	数据集	说明
B. 基本信息	Demographic_Background	主要受访者及其配偶的基本人口学信息
C. 家庭信息	Family_Information	家庭以及家户成员信息
D. 健康状况与功能	Health_Status_and_Functioning	健康状态、健康行为以及认知能力
F. 工作与退休	Work_Retirement	工作以及退休状况
G1. 家户收入与支出	Household_Income	家户收入、支出以及住房情况
G2. 个人收入	Individual_Income	个人收入
V. 疫情	COVID_Module	受访者疫情期间的相关信息
EX. 退出问卷	EXIT_Module	去世受访者的信息
	Weights	横截面权重
	Sample_Infor	样本信息

所有的数据集均以 Stata 14 格式存储，用户可以通过随数据发布的“codebook”获取数据的基本统计描述。

样本识别相关的 ID（ID，householdID 以及 communityID）均可以与往期数据匹配，需要注意的是，如果要与基线数据匹配，需要根据第二期数据的发布说明调整基线 ID。

7　表格附录

表 1: 受访者年龄性别分布: 2011 - 2020

年龄组	基线，2011			第二轮，2013			第三轮，2015			第四轮，2018			第五轮，2020		
	合计	男性	女性	合计	男性	女性	合计	男性	女性	合计	男性	女性	合计	男性	女性
-44	483	74	409	435	76	359	718	137	581	255	31	224	87	11	76
45-49	3,575	1,643	1,932	3,153	1,398	1,755	3,175	1,503	1,672	1,960	820	1,140	526	120	406
50-54	2,707	1,310	1,397	2,827	1,348	1,479	3,551	1,694	1,857	3,500	1,664	1,836	2,727	1,255	1,472
55-59	3,520	1,721	1,799	3,406	1,655	1,751	3,095	1,532	1,563	3,045	1,429	1,616	3,934	1,856	2,078
60-64	2,823	1,432	1,391	3,152	1,581	1,571	3,594	1,723	1,871	3,375	1,665	1,710	2,687	1,303	1,384
65-69	1,836	928	908	2,084	1,051	1,033	2,537	1,297	1,240	3,162	1,512	1,650	3,495	1,698	1,797
70-74	1,291	681	610	1,466	756	710	1,679	823	856	1,996	1,002	994	2,701	1,325	1,376
75-79	850	427	423	981	511	470	1,083	577	506	1,330	656	674	1,598	779	819
80+	612	260	352	750	331	419	841	368	473	1,193	561	632	1,640	746	894
观测值	17,697	8,476	9,221	18,254	8,707	9,547	20,273	9,654	10,619	19,816	9,340	10,476	19,395	9,093	10,302

缺少合理年龄记录的受访者：2011 有 11 位，2013 有 10 位，2015 有 11 位。

表 2: 第五轮调查家户问卷主要内容

**B. 基本信息**<br>出生日期<br>居住地<br>户口、教育、婚姻<br>养老金、医疗保险	**F. 工作与退休**<br>工作概况<br>当前主要工作的详细信息<br>求职和就业<br>疫情期间工作状况<br>退休手续
**C. 家庭信息**<br>子女<br>人口学基本信息<br>和父母的联系及经济往来<br>疫情对收入以及与父母之间关系的影响<br>家户成员基本信息<br>春节期间的家庭联系	**G. 收入与支出**<br>家户收入与支出<br>个人收入<br>住房情况
**D. 健康状况与功能**<br>自报总体健康状况<br>医生诊断的慢性病<br>疼痛、意外伤害、摔倒和骨折<br>睡眠、运动、社交活动、抽烟和喝酒<br>身体功能障碍和辅助者<br>认知及抑郁<br>医疗服务利用状况及其受疫情影响的状况	**V. 疫情**<br>疾病防范意识<br>个人患病和隔离<br>疫情期间个人活动<br>疫情期间居住地管控

表 3: 应答率: 2011-2020

		基线	第二轮，2013		第三轮，2015		第四轮，2018		第五轮，2020	
		2011[a]	横截面[b]	面板[c]	横截面[b]	面板[c]	横截面[b]	面板[c]	横截面[b]	面板[c]
应答率 (%)	总体	80.51	82.63	88.30	82.13	87.15	83.84	86.46	84.31	86.81
	农村	94.15	91.74	92.18	91.32	93.13	91.40	92.79	92.54	93.80
	城市	68.63	72.20	82.61	71.64	78.45	74.55	77.24	74.45	76.76
家户数	总体	10,257	10,629	9,022	11,797	8,715	10,524	8,288	10,204	8,046
	农村	6,033	6,340	5,547	6,993	5,483	6,456	5,226	6,303	5,089
	城市	4,224	4,289	3,475	4,804	3,232	4,068	3,062	3,901	2,957
个人数	总体	17,708	18,264	15,196	20,284	14,522	17,970	13,567	17,364	13,095
	农村	10,537	10,950	9,439	12,075	9,200	11,017	8,622	10,739	8,352
	城市	7,171	7,314	5,757	8,209	5,322	6,953	4,945	6,625	4,743

[a] 基线应答率为应答家户数除以推算的适龄家户数。

[b] 追访轮次（2013-2020）横截面应答率的计算与基线相同。

[c] 追访轮次（2013-2020）面板应答率为基线应答受访者中本轮应答的人数除以基线应答人数。由于 CHARLS 在后续追访中不仅追访基线的应答样本，还会继续尝试基线无应答的样本，所以横截面样本不一定是面板样本。

参考文献

Kinsella, Kevin, Wan He, and Peter-O. Way. 2009. *An aging world: 2008, International Population Reports*. U.S. Governement Printing Office.

United Nations, Population Division, Department of Economic and Social Affairs. 2022. *World Population Prospects: The 2022 Revision*. UN.

Yang, Gonghuan, Yu Wang, Yixin Zeng, George F. Gao, Xiaofeng Liang, Maigeng Zhou, Xia Wan, Shicheng Yu, Yuhong Jiang, Mohsen Naghavi, Theo Vos, Haidong Wang, Alan D. Lopez, and Christopher JL Murray. 2013. "Rapid health transition in China, 1990–2010: findings from the Global Burden of Disease Study 2010." *The Lancet* 381 (9882):1987–2015.

Zhao, Yaohui, Yisong Hu, James P Smith, John Strauss, and Gonghuan Yang. 2014. "Cohort Profile: The China Health and Retirement Longitudinal Study (CHARLS)." *Int J Epidemiol* 43 (1):61–8.

Zhao, Yaohui, John Strauss, Gonghuan Yang, John Giles, Peifeng (Perry) Hu, Yisong Hu, Xiaoyan Lei, Man Liu, Albert Park, James P. Smith, and Yafeng Wang. 2013. "China Health and Retirement Longitudinal Study: 2011-2012 National Baseline User's Guide." Tech. rep., National School of Development, Peking University.
