# Supplementary material for: Association of internet use and health service utilization with self-rated health in middle-aged and older adults: findings from a nationally representative longitudinal survey
Source: Front Public Health. 2024 Oct 3;12:1429983. doi: 10.3389/fpubh.2024.1429983 (PMC11483889; doi:10.3389/fpubh.2024.1429983)
Supplement: Supplementary file 9 [file Table_1.docx]

**Supplementary Table S1 The definition of main variables**

| Variables | Explanation |  |  |  |
| --- | --- | --- | --- | --- |
|  |  | Definition |  |  |
| Explained variable (Y) | Self-rated health | What do you think of your health? Is it very good, good, average, bad or very bad? |  |  |
| Explanatory variable (X) | Internet use | Did you surf the Internet in the past month? Including chatting, watching news, watching videos, playing games, managing money and so on. |  |  |
| Control variable (C) | Sex | What's your gender? |  |  |
|  | Age | When were you born? |  |  |
|  | Marital status | What is your current marital status? |  |  |
|  | Education | What is your highest education level (excluding adult education)? |  |  |
|  | Current address | What is your current type of residence? |  |  |
|  | Medical insurance | Do you have medical insurance? |  |  |
|  | Endowment insurance | Do you have endowment insurance? |  |  |
|  | Duration of residence of children | How long (months) have you lived with your children in the past year? |  |  |
|  | Drinking | In the past year, did you drink alcohol, including beer, wine, rice wine, yellow wine or white wine, medicinal liquor, etc. |  |  |
|  | Exercise | Do you usually do this type of moderate-intensity physical activity consistently for at least 10 minutes per week? |  |  |
|  | Sleep duration | In the past month, on average, how many hours did you actually sleep each night? |  |  |
| Moderator and mediator variable | Outpatient | In the past month, have you visited a health care facility for outpatient or in-home medical care? |  |  |
|  | Inpatients | Have you been hospitalized in the past year? |  |  |

3.8.Analysis of the moderating effect of the action path

Adopting the framework outlined by Baron and Kenny(37), our study scrutinized the roles of independent, dependent, and moderating variables to elucidate the moderating effects of health service utilization on the relationship between Internet use and self-rated health. We constructed a model to analyze these moderating effects, designating self-rated health as the dependent variable, Internet use as the independent variable, and health service utilization as the moderating variable, while adjusting for other covariates. The findings indicated that both outpatient service utilization (β = -0.475, P <0.001) and inpatient service utilization (β = -0.584, P <0.001) significantly negatively impacted self-rated health, as detailed in Table S2 and illustrated in Figure S1.

**Table S2** Moderating role of health service utilization on Internet use – self-rated health. (N =17091)

|  | Variables | Coefficient | SE | t value | Bootstrap 95% CI | |
| --- | --- | --- | --- | --- | --- | --- |
|  |  |  |  |  | Lower | Upper |
| Self-rated health(Y) |  |  |  |  |  |  |
| Independent variable | Internet use | 0.197^***^ | 0.019 | 10.439 | 0.160 | 0.234 |
| Moderator variable | Outpatient(W) | -0.475^***^ | 0.020 | -24.296 | -0.513 | -0.436 |
| Interaction | X × W | -0.038 | 0.044 | -0.870 | -0.125 | 0.048 |
| Constant |  | 3.338^***^ | 0.066 | 50.514 | 3.208 | 3.467 |
| Control variable | Yes | | | | | |
| Pseudo R^2^ | 0.085 | | | | | |
| Prob>F | <0.001 | | | | | |
|  |  |  |  |  |  |  |
| Self-rated health(Y) |  |  |  |  |  |  |
| Independent variable | Internet use | 0.174^***^ | 0.019 | 9.266 | 0.137 | 0.210 |
| Moderator variable | Inpatients(W) | -0.584^***^ | 0.020 | -29.470 | -0.623 | -0.545 |
| Interaction | X × W | -0.005 | 0.047 | -0.112 | -0.097 | 0.087 |
| Constant |  | 3.272^***^ | 0.066 | 49.859 | 3.144 | 3.401 |
| Control variable | Yes | | | | | |
| Pseudo R^2^ | 0.099 | | | | | |
| Prob>F | <0.001 | | | | | |

***, **, and * indicate significance at the 1, 5, and 10% levels, respectively. The numbers in the brackets are the standard errors of coefficient robustness.

| **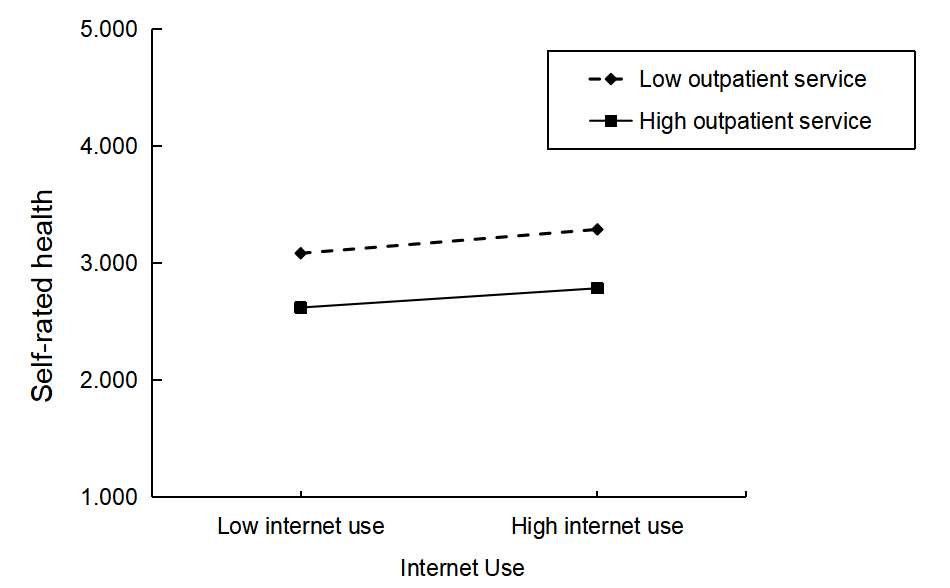** | **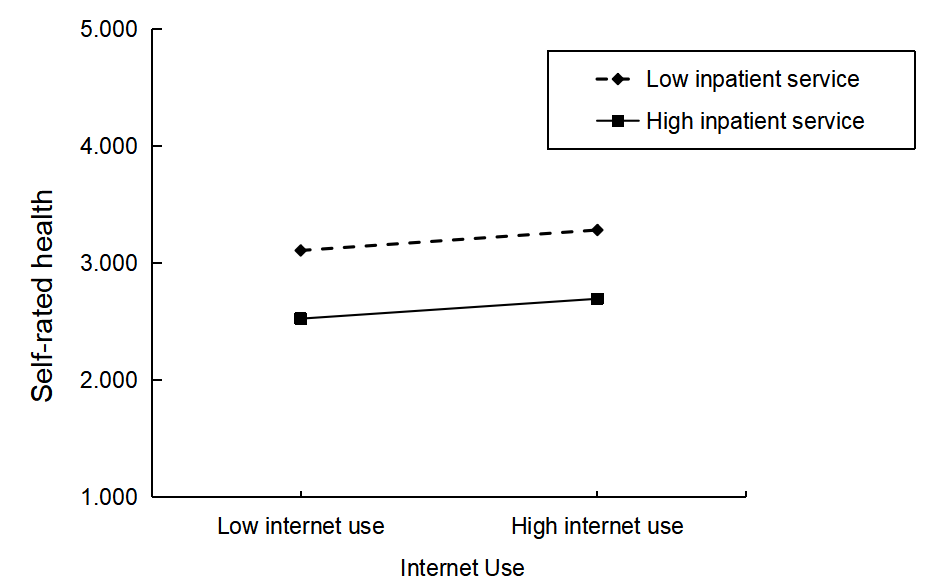** |
| --- | --- |

**Figure S1** Moderating effect of Internet use and health service utilization on self-rated health.
